# Dyotropic Rearrangement of Hypervalent Iodine Species: Migrative Heterofunctionalization of Quaternary Carbons

**DOI:** 10.1002/anie.7649039

**Published:** 2026-08-08

**Authors:** Chen‐Xu Liu, Qian Wang, Matthew D. Wodrich, Jieping Zhu

**Affiliations:** ^1^ Laboratory of Synthesis and Natural Products (LSPN), Institute of Chemical Sciences and Engineering Ecole Polytechnique Fédérale de Lausanne, EPFL‐SB‐ISIC‐LSPN, BCH5304 Lausanne Switzerland; ^2^ Laboratory For Computational Molecular Design (LCMD), Institute of Chemical Sciences and Engineering Ecole Polytechnique Fédérale de Lausanne Lausanne Switzerland

**Keywords:** dyotropic rearrangement, hypervalent iodine, phenonium ion, quaternary carbon editing, reaction mechanisms

## Abstract

Phenonium intermediates are almost universally invoked to account for 1,2‐aryl migration processes in reactions that exploit in situ‐generated hypervalent iodine(III) leaving groups. Here, we demonstrate that such transformations can instead proceed through a previously unrecognized 1,2‐C/I(III) dyotropic rearrangement, which represents a fundamentally different mechanistic manifold in iodine(III) chemistry. Treatment of ethyl 2‐alkyl‐2‐aryl‐3‐iodopropanoates with Selectfluor and Py·9HF enables migratory aryl fluorination across a C(sp^3^)─C(sp^3^) bond, installing fluorine regioselectively at the ester Cα position. The same dyotropic logic extends to migratory acyloxy arylation and alkoxy arylation reactions using hypervalent iodine reagents in combination with carboxylic acids or alcohols, respectively, and proceeds with inversion of absolute configuration at the Cα stereocenter. Although a phenonium intermediate is well‐established for many systems, this study demonstrates that a dyotropic rearrangement pathway can be energetically favored, offering a distinct rationale for the observed reaction outcome.

## Graphical abstract

1,2‐Aryl migration involving in situ‐generated alkyl iodine(III) species proceeds through a previously unrecognized 1,2‐C/I(III) dyotropic rearrangement. Migratory aryl fluorination, acetoxylation, and alkoxylation of 2‐alkyl‐2‐aryl‐3‐iodopropanoates provide α‐heterofunctionalized products, accompanied by aryl migration from the α‐ to the β‐position.

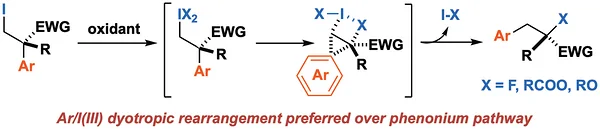

## 1 Introduction

Numerous 1,2‐aryl migration processes promoted by in situ‐generated I(III) species have been reported [1] and are almost invariably interpreted through a neighboring group participation framework, involving phenonium formation followed by regioselective ring opening [2]. This mechanistic picture has become the dominant paradigm for rationalizing rearrangements mediated by hypervalent iodine reagents. A representative example is the reaction of styrene derivatives **1** with Py∙(HF)_n_ and ArIF_2_, which affords 1,1‐difluorinated products **2** and is typically explained via l^3^‐iodane **A** and phenonium intermediate **B** (Scheme 1a) [3, 4, 5, 6, 7, 8, 9, 10, 11]. This reaction can also be rendered catalytic in aryl iodide, enabling the development of catalytic enantioselective variants [12]. The broad acceptance of this mechanistic interpretation is further reflected in its extension to alkyl [13, 14], halogen [15, 16], boron [17], hydride [18], azide [19], sulfonyloxy [20], and alkyne [21, 22] migrations (Scheme 1a, inset) [23], with alkyl shifts frequently described as Wagner–Meerwein‐type rearrangement [13, 14].

**SCHEME 1:**
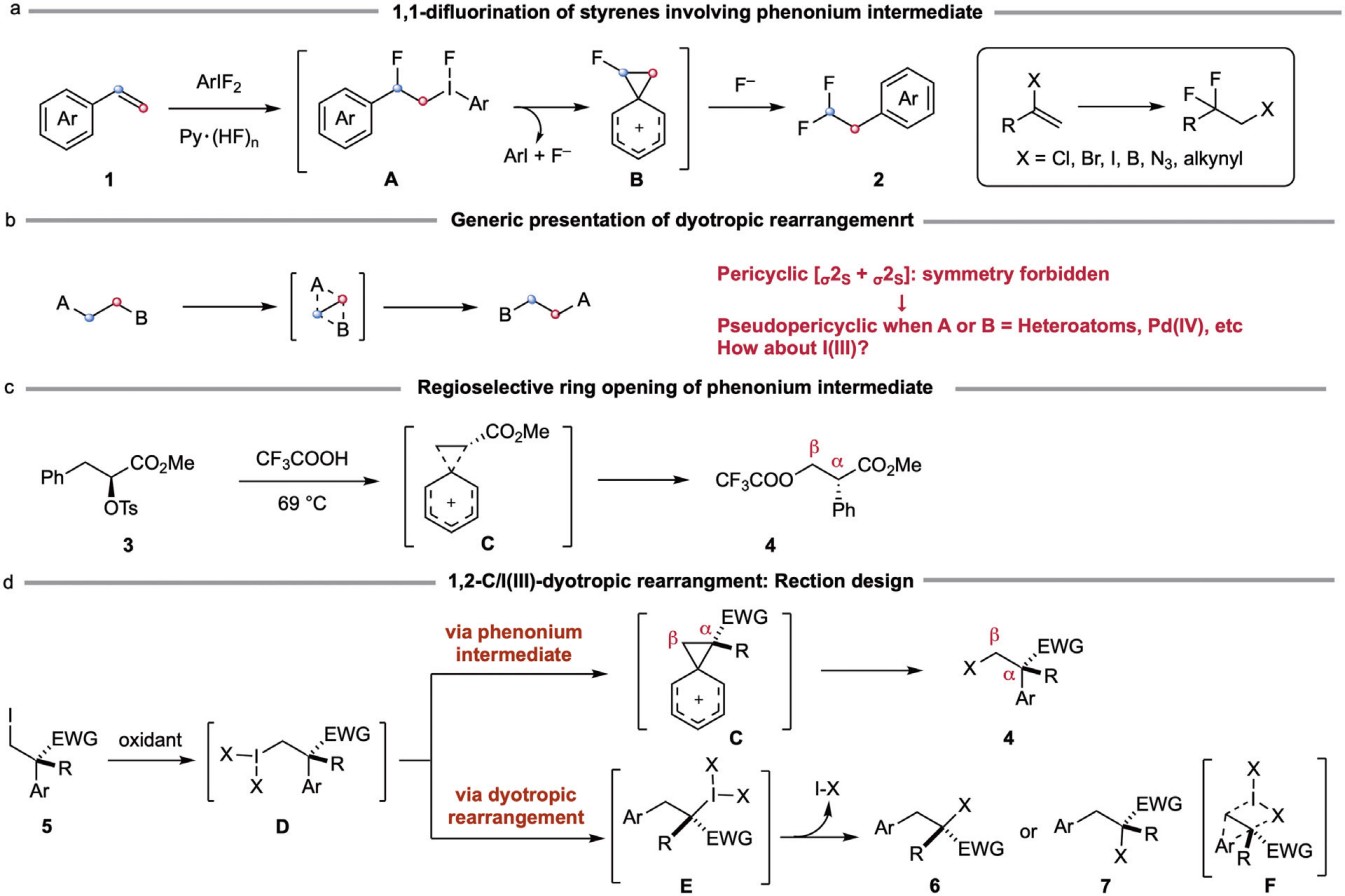
Exploiting I(III)‐based 1,2‐rearrangment. (a) *Gem*‐difluorination of styrenes: phenonium ions are the accepted intermediates, even when 4‐nitrophenyl acts as the migrating group. (b) Generic presentation of concerted type I dyotropic rearrangement. (c) Literature reports on the regioselective ring opening of phenonium intermediate. (d) I(III)‐based dyotropic rearrangement: reaction design. EWG, electron withdrawing group.

Despite substantial experimental and computational (DFT) [24, 25] support for the involvement of phenonium intermediates, alternative mechanistic manifolds have received comparatively little scrutiny. In particular, a 1,2‐C/I(III) dyotropic rearrangement (DR) has not been explicitly considered in prior computational analysis of I(III)‐mediated migrations. Given the unique electronic structure of l^3^‐iodane **A** and inspired by a recent report on a Cl/I(III)‐DR [26], we questioned whether such a pathway might be energetically accessible and potentially competitive with the classical phenonium mechanism. Formally, a type I DR corresponds to a four‐electron [_σ_2_S_ + _σ_2_S_] process and is therefore symmetry‐forbidden under the Woodward–Hoffmann (WH) rules (Scheme 1b) [27, 28]. Consistent with this restriction, reported DRs typically involve at least one heteroatom, whose lone pair participation renders the process pseudopericyclic [29] and circumvents orbital‐symmetry constraints. In this context, we recently demonstrated that Pd(IV) can serve as a migrating group [30] and developed domino processes incorporating a 1,2‐C/Pd(IV) DR reaction [31, 32, 33, 34, 35], illustrating that high‐valent elements can engage directly in concerted migration events. These precedents prompted us to re‐examine the mechanistic foundations of iodine(III)‐mediated rearrangements and to evaluate whether dyotropic pathways represent an unrecognized yet general mode of reactivity in hypervalent iodine chemistry.

To determine whether a 1,2‐C/I(III) dyotropic rearrangement can contribute to I(III)‐mediated migration processes, we sought a mechanistically diagnostic substrate capable of unambiguously distinguishing a phenonium pathway from a dyotropic rearrangement. It has been reported that methyl (*S*)‐3‐phenyl‐2‐(tosyloxy)propanoate (**3**) undergoes acid‐induced rearrangement to furnish (*R*)‐**4** via regioselective nucleophilic attack at the β carbon of the phenonium intermediate **C** (Scheme 1c) [36, 37]. Guided by this precedent, we identified readily accessible alkyl iodides **5** as ideal probes. If l^3^‐iodane **D**, generated in situ via oxidation of alkyliodide **5**, were to react through a phenonium intermediate, nucleophilic ring‐opening would be expected to occur preferentially at the β–rather than the α–carbon–for both steric and stereoelectronic reasons [36, 37], leading to product **4** (Scheme 1d). In contrast, a 1,2‐C/I(III) dyotropic rearrangement would generate intermediate **E**, from which reductive elimination would provide **6**, whereas an S_N_2 displacement would afford its enantiomer **7**. Alternatively, dyotropic rearrangement involving transition state (TS) **F** would directly furnish the arylfluorination product **6**. Herein, we show that alkyl iodide **5** is converted efficiently to **6** under mild oxidative conditions. A diverse set of nucleophiles, including fluoride, carboxylates, and alcohols, is installed regioselectively at the α‐carbon of carbonyl compounds. Notably, reactions of enantioenriched substrates proceed with net inversion of absolute configuration at the α stereocenter, consistent with C‐X bond formation via reductive elimination from **E** or direct F‐transfer via TS **F**. Both control experiments and computational studies support a 1,2‐C/I(III) dyotropic rearrangement as a viable, previously overlooked mechanistic pathway in iodine(III)‐mediated 1,2‐migrations. Beyond its mechanistic significance, this transformation provides a practical and modular approach for converting simple alkyl iodides into α‐heteroatom‐substituted carbonyl compounds of significant synthetic importance [38].

## 2 Results and Discussion

### 2.1 Reaction Development and Scope

Hypervalent iodoalkanes, generated by oxidation of alkyl iodide, are inherently unstable [39, 40] and prone to competing substitution, elimination, oxidation, and rearrangement pathways [41, 42, 43], which has limited their synthetic utility. Using ethyl 3‐iodo‐2‐methyl‐2‐phenylpropanoate (**5a**) as a test substrate, we evaluated conditions for a planned migratory fluorination reaction (See Supporting Information). A combination of Py∙9HF and Selectfluor {1‐chloromethyl‐4‐fluoro‐1,4‐diazoniabicyclo[2.2.2]octane bis(tetrafluoroborate)} in DCE (*c* 0,1) at room temperature proved optimal, affording ethyl 2‐fluoro‐2‐methyl‐3‐phenylpropanoate (**6a**) in 80% yield (Scheme 2a). *m*CPBA also served as a competent oxidant, converting **5a** to **6a** in 69% yield, whereas Oxone under otherwise identical conditions delivered only 10% yield of **6a**. Solvent screening revealed that dichloromethane and chloroform were also suitable (75%–76%), whereas more polar solvents, such as acetonitrile and *N*,*N*‐dimethylformamide, were ineffective. Although xenon difluoride also promoted the reaction (75% yield) [44], its toxicity and handling challenges led us to select Selectfluor for subsequent studies.

**SCHEME 2:**
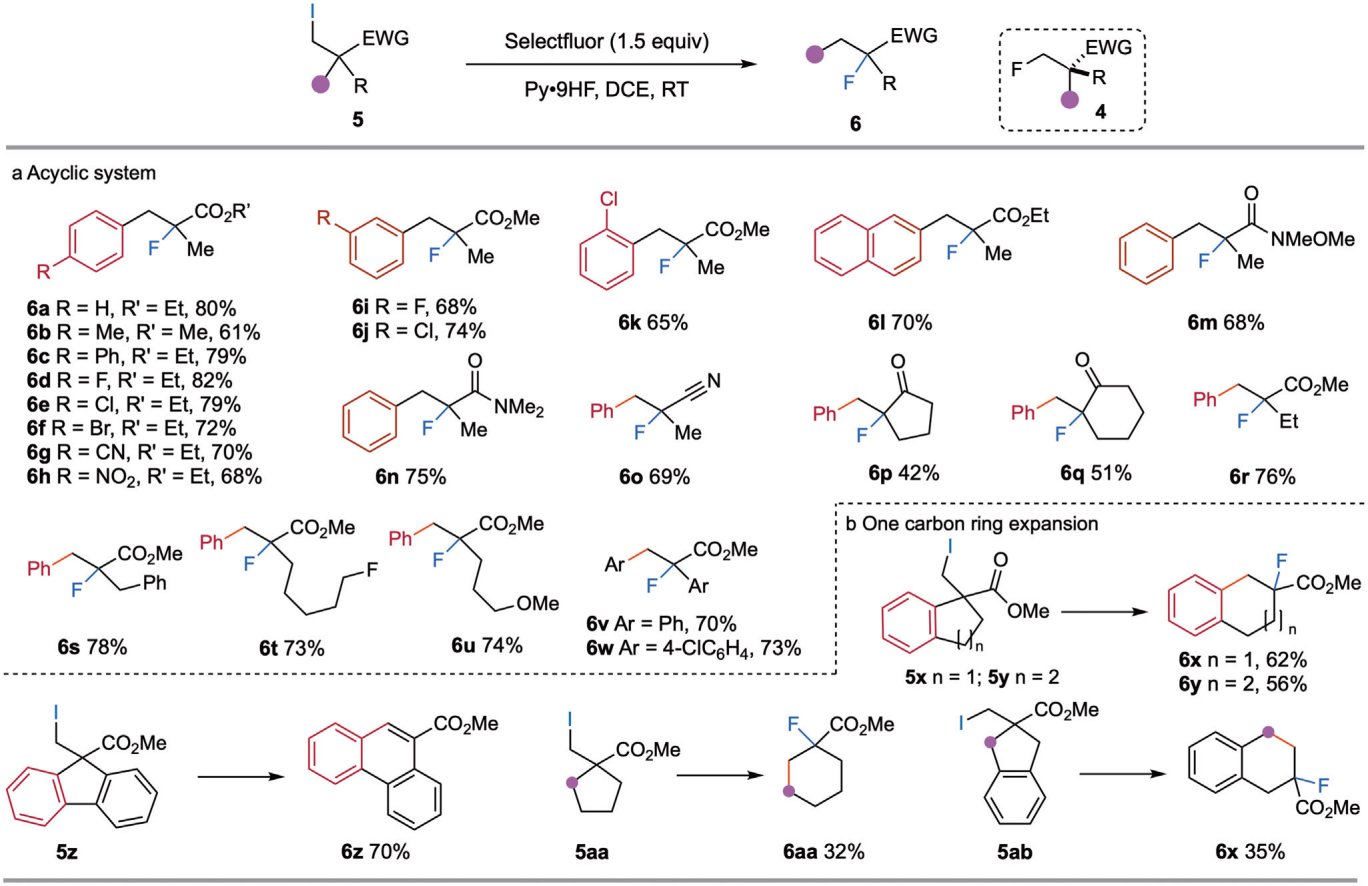
Editing the quaternary carbon enabled by in situ generated hypervalent iodine species: Migratory aryl fluorination. Reaction conditions: **5** (0.20 mmol), Selectfluor (1.5 equiv), Py∙9HF (0.2 mL), DCE (2.0 mL), RT. Yields refer to the isolated pure products. (a) Migratory aryl fluorination of an acyclic system. (b) Ring expansion of cycloalkanes. Selectfluor = 1‐chloromethyl‐4‐fluoro‐1,4‐diazoniabicyclo[2.2.2]octane bis(tetrafluoroborate), DCE = 1,2‐dichloroethane.

The generality of this rearrangement was then examined (Scheme 2a). Across all substrates, 1,2‐aryl migration consistently outcompeted alkyl migration, irrespective of the electronic nature or substitution pattern of the aromatic ring. Both electron‐donating (Me and Ph) and electron‐withdrawing substituents (F, Cl, Br, CN, and NO_2_) at various positions were well tolerated, affording **6d**−**6k** in high yields. Notably, the formation of **6h** via migration of a 4‐nitrophenyl group–an aryl substituent poorly predisposed to phenonium ion formation [37] provides a key mechanistic insight. A naphthyl substituent likewise underwent efficient migration to afford the corresponding product **6l**. Beyond ester substrates, amide (**6m** and **6n**), nitrile (**6o**), and ketone (**6p** and **6q**) were all compatible with the reaction conditions. Substrates bearing ethyl (**6r**), benzyl (**6s**), 5‐fluoropentyl (**6t**), 3‐methoxypropyl (**6u**), and aryl groups (**6v** and **6w**) at the α‐position were smoothly converted to the corresponding products in good yields.

Gratifyingly, this protocol could be extended to fluorinative one‐carbon ring expansion of cycloalkanes (Scheme 2b). Benzocyclopentane **5x** and benzocyclohexane **5y** underwent smooth fluorinative ring expansion to benzocyclohexane **6x** and benzocycloheptane **6y** in 62% and 56% yield, respectively [45]. Similarly, a fluorenyl substrate **5z** was transformed into a fully conjugated phenanthrene **6z**, resulting from a migration/elimination sequence, in 70% yield. Importantly, methyl 1‐(iodomethyl)cyclopentane‐1‐carboxylate (**5aa**) and methyl 2‐(iodomethyl)‐2,3‐dihydro‐1*H*‐indene‐2‐carboxylate (**5ab**) were converted efficiently into **6aa** and **6x**, respectively, via alkyl migration. These results are incompatible with a stepwise Wagner–Meerwein type rearrangement that would require carbocation formation α to an ester group and instead support a non‐cationic σ‐bonds metathesis pathway. It is also noteworthy that two distinct starting materials **5x** and **5ab** are converted to identical ring‐expanded product **6x**.

To further explore the scope of this transformation, we next examined the introduction of acyloxy groups at the α‐quaternary carbon of carbonyl compounds. Gratifyingly, stirring a solution of **5d** and [PIDA, phenyliodine(III) diacetate] [46] in a DCM/HFIP (*v/v* = 1:1) solvent mixture afforded the migratory acetoxylation product **8d** in 65% yield (Scheme 3a). These conditions proved to be broadly applicable. Aryl substituents bearing electron‐donating or electron‐withdrawing groups at *para*‐, *meta*‐, and *ortho*‐positions readily underwent migratory acetoxylation to furnish the corresponding products **8a**–**8m**. 3‐Iodo‐2‐methyl‐2‐phenylpropanenitrile was likewise converted smoothly to product **8n**. Finally, acetoxylative one‐carbon ring expansion of **5y** and **5aa** afforded **8o** and **8p**, albeit in moderate yields.

**SCHEME 3:**
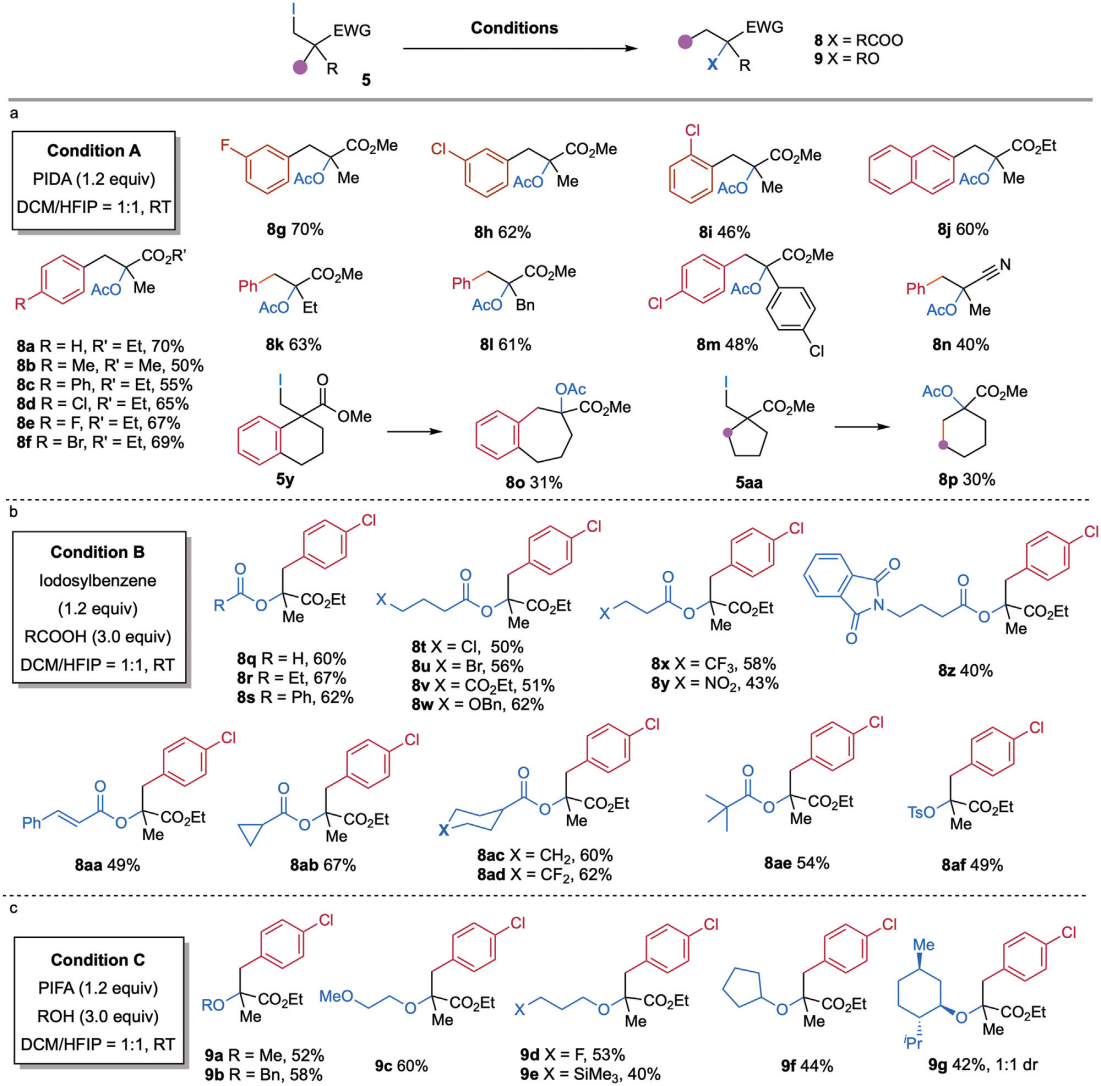
Editing the quaternary carbons enabled by in situ‐generated hypervalent iodine species. (a) Migratory aryl acetoxylation. (b) Migratory aryl acyloxylation. (c) Migratory aryl alkoxylation. All reactions were performed on a 0.2 mmol scale. PIDA = phenyliodine(III) diacetate, PIFA = phenyliodine(III) bis(trifluoroacetate). HFIP = 1,1,1,3,3,3‐hexafluoropropan‐2‐ol.

By generating phenyliodine dicarboxylates in situ, the migratory acyloxylation of **5** could be extended to a diverse set of carboxylic acids, enabling installation of different acyloxy groups at the α‐position (Scheme 3b). Reaction of ethyl 2‐(4‐chlorophenyl)‐3‐iodo‐2‐methylpropanoate (**5e**) with iodosylbenzene (1.2 equiv) and formic acid (3.0 equiv) in DCM/HFIP (*v/v* = 1:1) furnished the α‐formyloxylated product **8q** in 60% yield. Propionic acid (**8r**), benzoic acid (**8s**), and a range of functionalized carboxylic acids (**8t–8z**) bearing halogen (Cl and Br), ester, benzyl ether, nitro, and imido groups were also competent reaction partners. Notably, the olefinic functionality in cinnamic acid was well tolerated (**8aa**). α‐Secondary carboxylic acids, such as cyclopropane carboxylic acid and cyclohexane carboxylic acid, as well as the α‐tertiary carboxylic acid (pivalic acid), participated in the reaction to afford the corresponding acyloxylated products (**8ab**–**8ae**). In addition, *p*‐toluenesulfonic acid, despite its weak nucleophilicity, engaged in the reaction to deliver sulfonylated product **8af**.

Migratory alkoxylation also proceeds smoothly when alcohols are used as nucleophiles, with PIFA as the oxidant. A range of alcohols participated in the reaction to deliver the corresponding α‐alkoxylated products **9a**–**9g** in moderate to good yields (Scheme 3c). Together with the fluorination and acyloxylation reactions described above, these results highlight the generality of this rearrangement for installing diverse heteroatom functionalities at the α‐position of esters via a common migratory pathway.

We note that these reactions generally provide a clean crude mixture, even in cases where the rearrangement products are obtained in only moderate yields. This suggests that the remaining mass balance may be attributed to the partial decomposition of the hypervalent iodine intermediate into intractable materials [39, 40, 41, 42, 43].

Across all reactions examined, the β‐functionalized regioisomer (**4**, *cf* Scheme 1d) was not detected. This result is inconsistent with a phenonium pathway and instead supports a 1,2‐C/I(III) dyotropic rearrangement. Moreover, the observation that polar solvents such as DMF are ineffective for this reaction disfavors a cationic mechanism. Finally, we note that this transformation provides a unified platform for the introduction of a heteroatom at the quaternary carbon α to the carbonyl group, a transformation that is otherwise difficult to achieve [47, 48, 49].

### 2.2 Mechanistic Consideration and Computational Studies

To gain insight into the mechanism of the migratory aryl fluorination reaction of **5a**, a series of control experiments were performed. In the absence of Selectfluor, no reaction occurred. Notably, omission of Py·9HF still afforded **6a** in 10% yield after 30 h. Since tetrafluoroborate (BF_4_^−^), the counteranion of Selectfluor, has previously been reported to serve as a potential fluoride donor [6, 50], this result suggests that BF_4_^−^ may contribute to product formation. However, the low yield indicates that BF_4_^−^ is not an efficient fluoride donor for the present rearrangement, and Py·9HF remains the primary fluoride source.

In the migratory fluorination of **5** to **6**, iodine monofluoride (IF), a highly reactive iodination reagent, is expected to be generated in situ (*cf* Scheme 1d) [51]. Surprisingly, no iodinated products were initially detected. This observation may be attributed to the inherent instability of IF (b.p. 1°C), which is known to decompose at 0°C to molecular iodine, a significantly weaker electrophile. To understand the reaction, the conversion of **5i** to **6i** was investigated in detail (Scheme 4a). Consistent with our hypothesis, the reaction mixture gradually turned deep purple, indicative of iodine formation. Upon extending the reaction time to 9 h, the iodinated product **10** was isolated in 50% yield (Scheme 4a). Moreover, addition of an excess of *o*‐xylene (5.0 equiv) to the reaction mixture under otherwise standard conditions afforded **6i** and 4‐iodo‐1,2‐dimethylbenzene (**11**, Scheme 4a inset) in yields of 50% and 20%, respectively, supporting the transient generation of electrophilic iodine species during the reaction.

**SCHEME 4:**
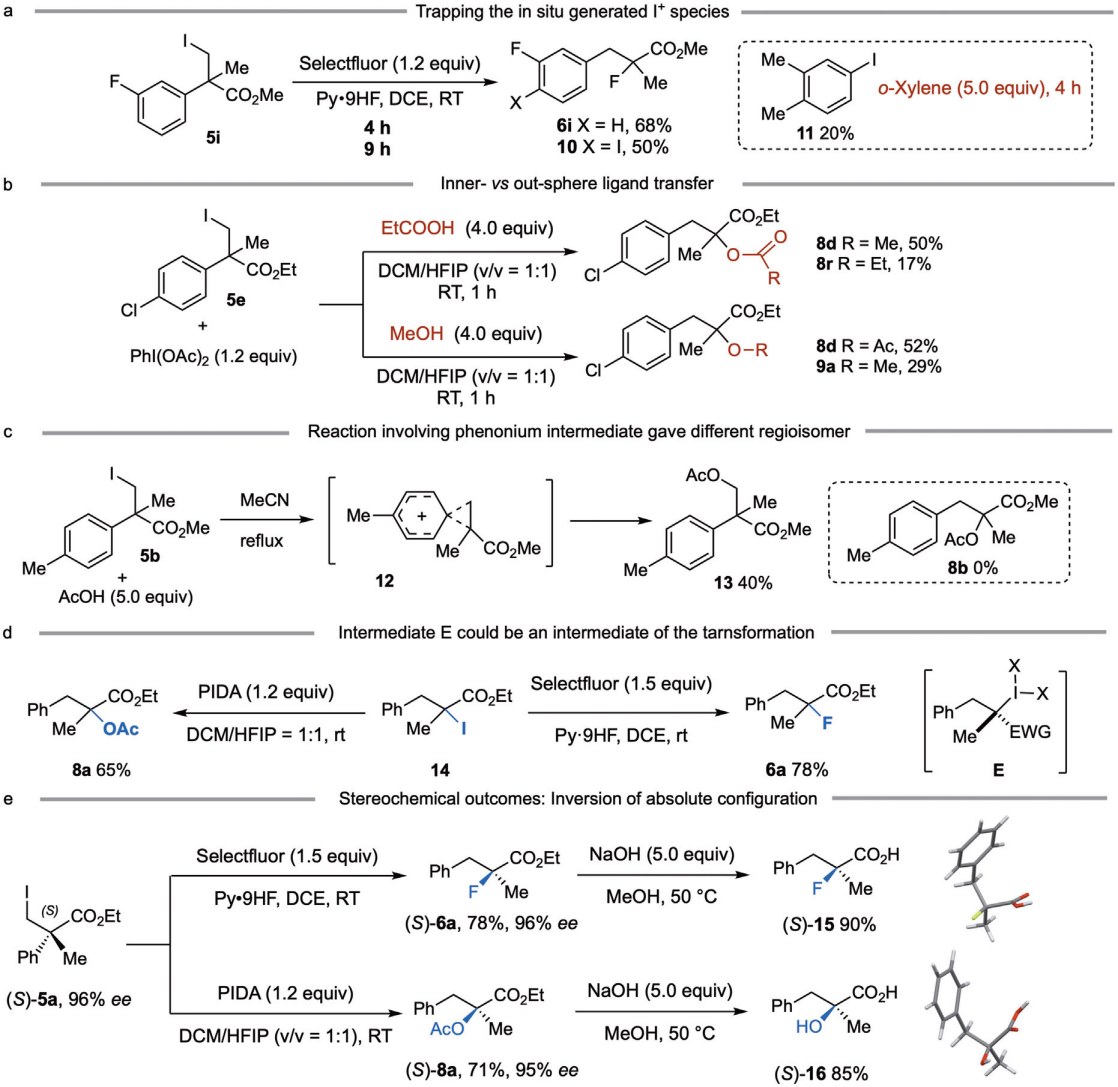
Control experiments and stereochemical outcome of the rearrangement reactions. (a) Trapping the in situ‐generated iodinating reagent. (b) Competition experiments indicating that ligand transfer from the I(III) intermediate is a major reaction pathway. (c) Ring‐opening of the phenonium intermediate affords different regioisomers, indicating that this intermediate might not be involved in the conversion shown in Schemes 2 and 3. (d) Intermediate **E** could be a possible intermediate of the transformation. (e) The absolute configuration of the quaternary stereocenter is inverted during the reaction.

The mode of C─O bond formation in the migratory acyloxylation was next examined. Treatment of **5e** with PIDA (1.2 equiv) in the presence of propanoic acid (4.0 equiv) afforded the acetoxylated product **8d** (*R* = Me, 50%), along with a minor amount of propionyloxylated product **8r** (*R* = Et, 17% yield). When MeOH (4.0 equiv), a stronger nucleophile than AcOH, was employed under the same conditions, **8d** and **9a** were isolated in 52% and 29% yields, respectively (Scheme 4b). Since both carboxylic acid and alcohols are known to undergo ligand‐exchange with *λ* ^3^‐iodanes [43, 52], we hypothesized that C─O bond formation most likely proceeds via ligand transfer followed by reductive elimination, although a direct nucleophilic substitution pathway cannot be completely ruled out.

In line with literature precedents [36, 37], acetolysis of methyl 2‐methyl‐3‐iodo‐2‐(*p*‐tolyl)propanoate (**5b**) in acetonitrile gave methyl 3‐acetoxy‐2‐ethyl‐2‐(*p*‐tolyl)propanoate (**12**) in 40% yield, with no detectable formation of **8b** (Scheme 4c). Given the severe steric hindrance associated with S_N_2 displacement at a neopentyl iodide, this transformation is best rationalized by β‐selective ring opening of phenonium intermediate **13** [53]. In contrast, our protocol delivers **8b** in 50% yield with complementary regioselectivity, underscoring its distinct mechanistic pathway and synthetic utility.

We next prepared 2‐iodo‐2‐methyl‐3‐phenylpropanoate (**14**). Subjecting **14** to the fluorination (Selectfluor and Py·9HF, RT) and acetoxylation conditions (PIDA, DCM/HFIP, RT) afforded products **6a** (78% yield) and **8a** (65% yield), respectively (Scheme 4d). These results suggest that **E** is a viable intermediate in the conversion of **5** to **6** and to **8**, although direct transfer of ligand X via transition state **F** (*cf* Scheme 1d) could well be a competitive pathway.

The stereochemical course of the reaction was next investigated. Enantioenriched (*S*)‐ethyl 3‐iodo‐2‐methyl‐2‐phenylpropanoate [(S)‐**5a**, 96% ee] was converted to product (*S*)‐**6a** (96% ee) and (*S*)‐**8a** (95% ee) under the standard migratory aryl fluorination and acyloxylation conditions, respectively, without erosion of enantiopurity (Scheme 4d). The absolute configurations of (*S*)‐**6a** and (*S*)‐**8a** were determined by x‐ray crystallographic analysis of their corresponding carboxylic acid (*S*)‐**14** and (*S*)‐**15** [54], confirming net inversion at the quaternary stereocenter during the transformation.

To gain deeper insight into the reaction mechanism, we performed DFT computations at the M06‐2X/def2‐TZVPPD (SMD, DCM)//M06‐2X/def2‐SVPD level of theory (see Computational Details for full protocol). The computed reaction profiles for the conversion of **5** to **6h** (Figure 1) show two plausible but mechanistically distinct pathways for the rearrangement. The dyotropic pathway (blue) proceeds through a single, concerted transition state (TS1_d_), directly connecting reactants and products along a relatively smooth potential energy surface, with an overall barrier of 21.4 kcal/mol. In contrast, the alternative phenonium ion pathway (red) proceeds in a stepwise manner, involving I─F bond cleavage (TS1_p_), formation of the phenonium ion (TS2_p_), and subsequent C─F bond formation (TS3_p_). Notably, the first step leading to int2_p_ is reversible, with the equilibrium largely favoring the Int1 (∆*G* = 3.6 kcal/mol). The highest energy point along this pathway, corresponding to the formation of the phenonium ion intermediate, is calculated to be 25.5 kcal/mol, which is 4.1 kcal/mol higher than the corresponding barrier for the dyotropic rearrangement pathway. These results establish the dyotropic pathway as the energetically preferred mechanism.

**FIGURE 1:**
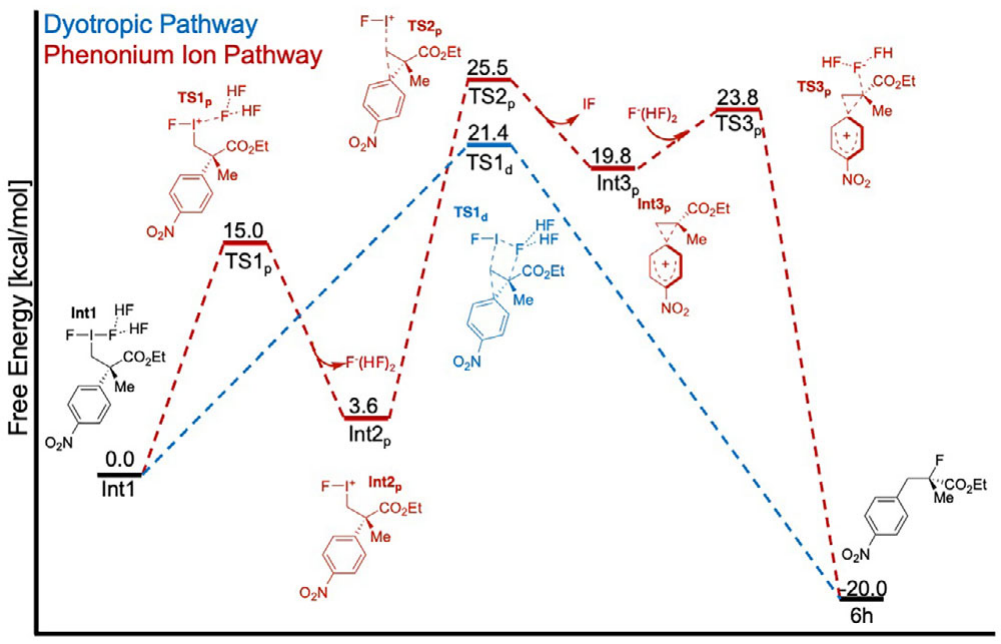
Conversion of **5** to **6h**. Computed free energy profiles of the dyotropic (blue) and phenonium ion (red) pathways, obtained at the M06‐2X/def2‐TZVPPD(SMD, DCM)//M06‐2X/def2‐SVPD level of theory.

Next, the two mechanistic pathways were calculated for the conversion of **5a** to **6a** (phenyl migration, Figure 2). Again, the concerted dyotropic rearrangement via the [2,1,0] bicyclic transition state is favored (E^≠^ = 15.7 kcal/mol) over the stepwise phenonium pathway, which features a peak energy barrier of 21.3 kcal/mol. Interestingly, the energy barrier difference between the two pathways is larger for the phenyl‐substituted substrate **5a** (5.6 kcal/mol) than for the 4‐nitrophenyl‐substituted substrate **5h** (4.1 kcal/mol). Comparing the two energy diagrams, the activation energy for **5a** via the phenonium intermediate is clearly lower than that of **5h**, which is consistent with the electronic properties of the respective aromatic rings. However, the activation energy for the dyotropic pathway decreases even more significantly when moving from the 4‐nitrophenyl group (21.4 kcal/mol) to the phenyl group (15.7 kcal/mol). Taken together, these findings indicate that the phenyl group has a much higher propensity to undergo dyotropic rearrangement compared to the electron‐deficient 4‐nitrophenyl group.

**FIGURE 2:**
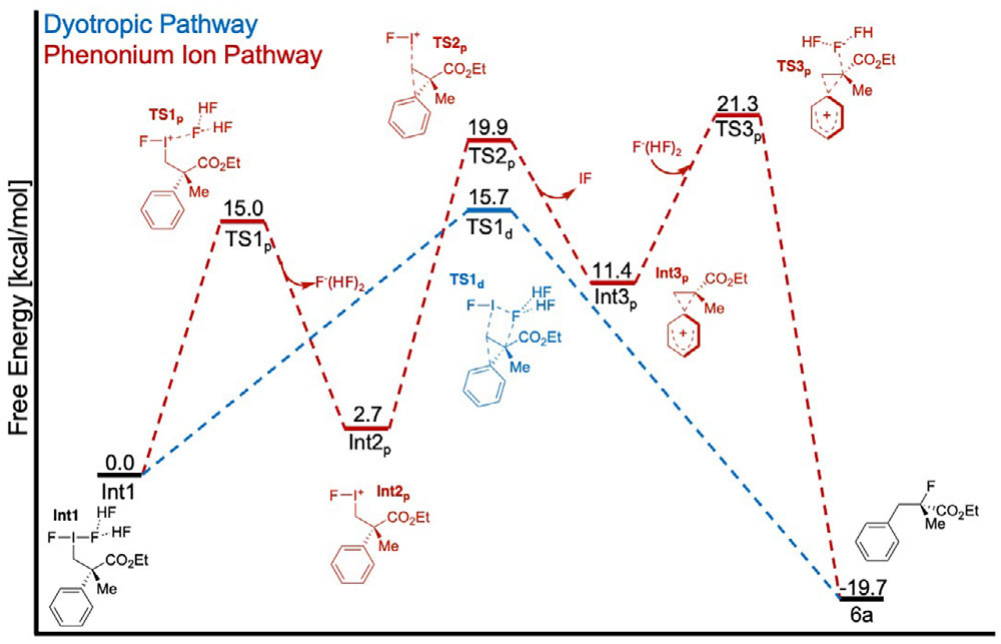
Conversion of **5a** to **6a**. Computed free energy profiles of the dyotropic (blue) and phenonium ion (red) pathways, obtained at the M06‐2X/def2‐TZVPPD(SMD, DCM)//M06‐2X/def2‐SVPD level of theory.

We also modeled the two potential pathways for the migratory acyloxy arylation reaction. In agreement with our previous findings, calculations indicate that the dyotropic mechanism is energetically much more favorable than the phenonium pathway. Specifically, the dyotropic pathway is favored by values of 4.1 and 20.3, kcal/mol for fluoroarylation and acetyloxyarylation, respectively (see Supporting Information).

To confirm that the pathways shown in Figure 1 indeed correspond to distinct dyotropic and phenonium ion mechanisms, we analyzed the intrinsic reaction coordinates (IRC) in the vicinity of the transition state (Figure 3). For the dyotropic pathway (Figure 3, left panels), progression toward the transition state (panel a) reveals a concerted bond reorganization. As shown in panel c, formation of the new C_β_─C_ipso_ bond (orange trace) occurs simultaneously with cleavage of the C_β_─I bond (blue trace). Concurrently, the C_α_─F bond begins to form (green trace), consistent with fluorine transfer from iodine, assisted by two HF molecules that stabilize the developing charge. The concerted process is further reflected in the charge distribution (CM5 charges, panel e), where positive charge is transferred from iodine to the aryl ring, reaching a maximum near the transition state before dissipating while moving toward the products along the reaction coordinate. Importantly, the aryl ring does not acquire a net positive charge at any point, supporting a concerted, non‐phenonium (dyotropic) pathway.

**FIGURE 3:**
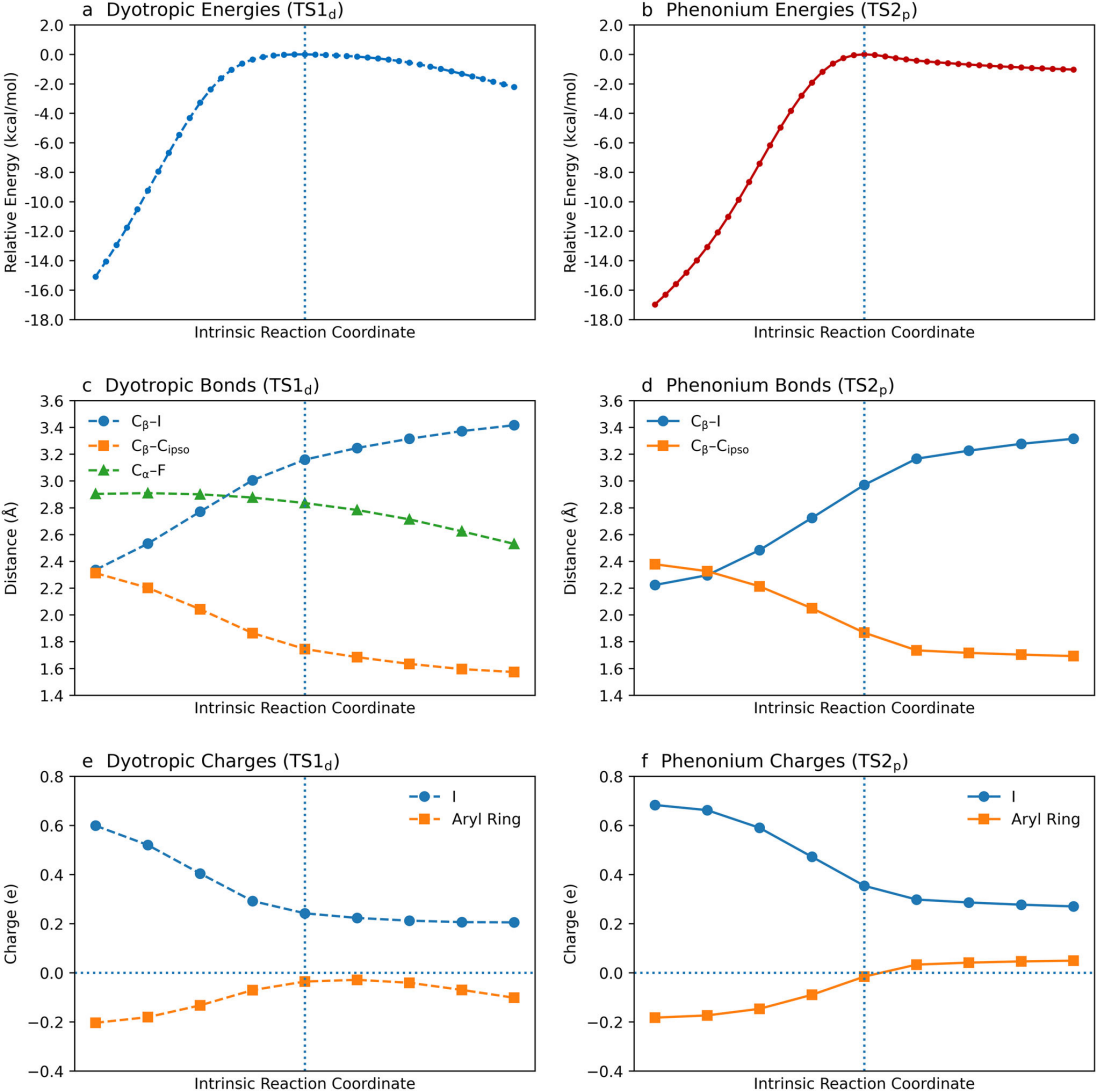
Intrinsic reaction coordinate (IRC) analysis in the vicinity of the transition states TS1_d_ and TS2_p_. (a,b) Relative electronic energies along the IRC of TS1_d_ and TS2_p_, respectively, computed at the M06‐2X/def2‐SVPD level, with the transition state set to zero in each case. (c,d) Selected bond distances for bonds being formed and cleaved along the IRC. (e,f) CM5 charges on the I and aryl ring (sum over carbon atoms), illustrating charge redistribution along the IRC.

In contrast, the phenonium pathway (Figure 3, right panels) exhibits markedly different behavior. As shown in panel d, formation of the C_β_─C_ipso_ bond (orange line) and cleavage of the C_β_─I bond (blue line) also occur in a concerted fashion near the transition state. However, the associated charge distribution (panel f) differs significantly: positive charge is transferred from the iodine (blue line) to the aryl ring (orange line) and continues to accumulate beyond the transition state. This sustained buildup of positive charge on the aryl ring is consistent with the formation of a discrete phenonium ion intermediate, which clearly distinguishes this pathway from the dyotropic mechanism. Taken together, these results provide clear electronic and structural evidence that the two pathways are mechanistically different, with only the phenonium pathway exhibiting true carbocationic character.

## 3 Conclusion

We demonstrate that 1,2‐aryl migration in reactions involving in situ‐generated hypervalent iodine(III) leaving groups proceeds via a previously unrecognized 1,2‐C/I(III) dyotropic rearrangement. Migratory aryl fluorination, acetoxylation, and alkoxylation of 2‐alkyl‐2‐aryl‐3‐iodopropanoates **5** furnish α‐heterofunctionalized products with concurrent aryl migration from the α‐ to the β‐position. In contrast, reactions of **5** with nucleophiles in the absence of oxidants proceed via a phenonium intermediate with complementary regioselectivity. These findings reveal a novel mechanistic manifold in hypervalent iodine chemistry. While a phenonium intermediate remains highly viable in many systems, the present study demonstrates that a dyotropic rearrangement pathway can be energetically favored, providing an alternative rationale for the reaction outcome.

## Author Contributions

**Chen‐Xu Liu**: investigation, writing – original draft, writing – review and editing, formal analysis, conceptualization. **Qian Wang**: conceptualization, investigation, writing – original draft, writing – review and editing, formal analysis. **Matthew D. Wodrich**: investigation, formal analysis, writing – review and editing. **Jieping Zhu**: conceptualization, funding acquisition, writing – original draft, writing – review and editing, supervision, resources.

## Conflicts of Interest

The authors declare no conflicts of interest.

## Supporting information




**Supporting File 1**: anie74083‐sup‐0001‐Data.zip.

**Supporting File 2**: anie74083‐sup‐0002‐SuppMat.pdf.

## Data Availability

The data that support the findings of this study are available in the Supporting Information of this article.
