# Supplementary material for: Dyotropic Rearrangement of Hypervalent Iodine Species: Migrative Heterofunctionalization of Quaternary Carbons
Source: Angew Chem Int Ed Engl. 2026 Aug 8;65(41):e7649039. doi: 10.1002/anie.7649039 (PMC13638110; doi:10.1002/anie.7649039)

Supplementary Information for

Dyotropic Rearrangement of Hypervalent Iodine Species: Migrative Heterofunctionalization of Quaternary Carbons

Chen-Xu Liu,[1] Qian Wang,[1] Matthew D. Wodrich,[2,*] Jieping Zhu[1,*]

1. Laboratory of Synthesis and Natural Products (LSPN), Institute of Chemical Sciences and Engineering, Ecole Polytechnique Fédérale de Lausanne, EPFL-SB-ISIC-LSPN, CH-1015 Lausanne, Switzerland.

2. Laboratory for Computational Molecular Design (LCMD), Institute of Chemical Sciences and Engineering, Ecole Polytechnique Fédérale de Lausanne, CH-1015 Lausanne, Switzerland.

* Email: matthew.wodrich@epfl.ch, jieping.zhu@epfl.ch

Table of Contents

General methods S3

Optimization of dyotropic rearrangement of hypervalent iodine species S4

General procedure for the synthesis of substrates..S6

General procedure for hypervalent iodine based dyotropic rearrangement reactions: migrative fluorination S24

General procedure for hypervalent iodine based dyotropic rearrangement reactions: migrative acyloxylation S37

General procedure for hypervalent iodine based dyotropic rearrangement reactions: migrative alkoxylation S53

Mechanism Study..S57

X-Ray crystallographic data S70

Computational studies...S82

References...S86

Copies of the NMR spectra...S87

General methods

Reagents and solvents were purchased from commercial sources (Aldrich, Acros, Merck, Fluka and VWR international) and used without further purification. Flash column chromatography was performed using Silicycle P60 silica: 230-400 mesh (40-63 μm) silica. Reactions were monitored using Merck Kieselgel 60F254 aluminium plates. TLC was visualized by UV fluorescence (254 nm) then $KMnO_4$ or phosphomolybdic acid. NMR spectra were recorded on a Brüker Avance III-400, Brüker Avance-400 or Brüker DPX-400 spectrometer at room temperature. Chemical shifts (δ) were reported in parts per million (ppm) relative to residual solvent peaks rounded to the nearest 0.01 for proton and 0.1 for carbon (ref: $CHCl_3$ [^{1}H: 7.26, ^{13}C: 77.0]). Coupling constants (J) were reported in Hz to the nearest 0.1 Hz. Peak multiplicity was indicated as follows s (singlet), d (doublet), t (triplet), q (quartet), p (pentet), sext (sextet), m (multiplet) and br (broad). Attribution of peaks was done using the multiplicities and integrals of the peaks. HRMS measurements were performed by the mass spectrometry service of the EPFL by ESI-TOF using a QTOF Ultima from Waters, or APPIFT-ICR using a linear ion trap Fourier transform ion cyclotron resonance mass spectrometer from Thermo Scientific. High resolution mass values are given in m/z. Melting points were measured using a Stuart SMP30. Infrared (IR) data were recorded on an Alpha-P Bruker FT-IR Spectrometer. Absorbance frequencies are reported in reciprocal centimeters (cm^{-1}).

Optimization of dyotropic rearrangement of hypervalent iodine species

Table S1. Survey of reaction conditions of migrative fluorination

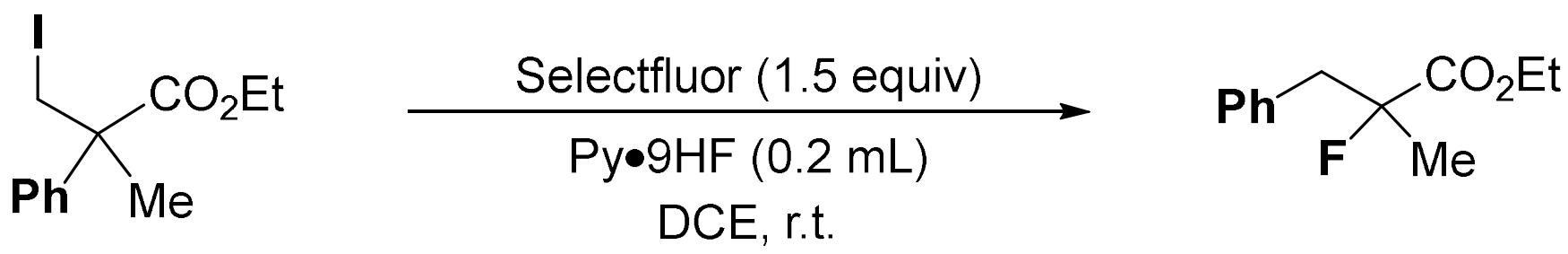

5a **6a**

[a]Entry	Modified conditions	Conversion	Isolated yield of **6a**
1	none	>95%	80%
2	Oxone as oxidant	>95%	10%
3	*m*-CPBA as oxidant	>95%	69%
4	PIDA as oxidant	>95%	54%
5	$Et_3N{\cdot}3HF$	>95%	19%
6	$Et_3N{\cdot}5HF$	>95%	34%
7	$CHCl_3$ as solvent	>95%	76%
8	CH_2Cl_2 as solvent	>95%	75%
9	MeCN as solvent	52%	24%
10	DMF as solvent	30%	--

[a]Conditions: **5a** (0.2 mmol), Selectfluor (0.3 mmol), Py·9HF (0.2 mL), DCE (2.0 mL), rt.

Table S2. Survey of reaction conditions of migrative acetoxylation

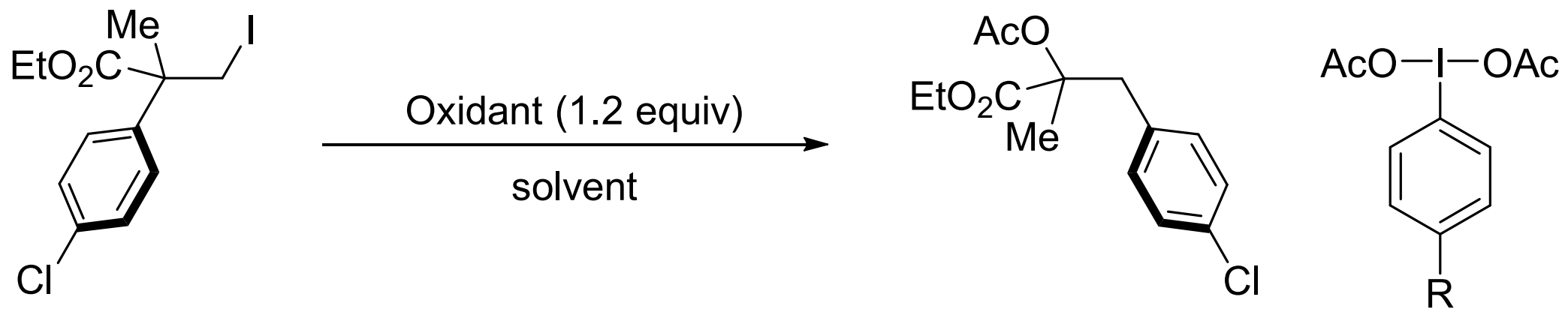

5e

8d

[O]-1, R = OMe
[O]-2, R = CO_2Me
[O]-3, R = CN

[a]entry	oxidant	solvent	conversion	yield
1	PIDA	DCM	12%	5%
2	PIDA	THF	<5%	--
3	PIDA	MeCN	<5%	--
4	PIDA	MeOH	<5%	--
5	PIDA	HFIP	>95%	53%
6	PIDA	DMF	<5%	--
7	PIDA	DCM/HFIP = 1 : 5	>95%	58%
8	PIDA	DCM/HFIP = 1 : 1	>95%	65%
9	PIDA	DCM/HFIP = 5 : 1	>95%	63%
10	PIDA	DCE/HFIP = 1 : 1	>95%	60%
11	**[O]-1**	DCM/HFIP = 1 : 1	>95%	60%
12	**[O]-2**	DCM/HFIP = 1 : 1	>95%	58%
13	**[O]-3**	DCM/HFIP = 1 : 1	>95%	55%

[a]Conditions: **5e** (0.2 mmol), Oxidant (0.24 mmol), Solvent (2.0 mL), rt.

General procedure for the synthesis of substrates

Substrates **5p**[1] and **5q**[2] are known compounds whose characterization has been reported in the literature.

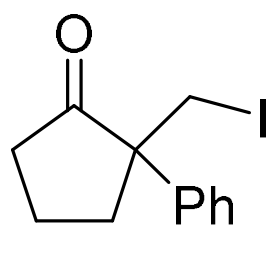

5p

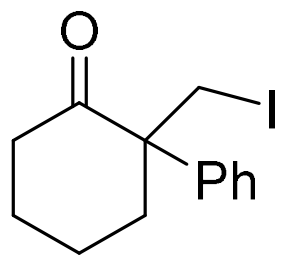

5q

All others were synthesized according to the following procedures.

General Procedure A:

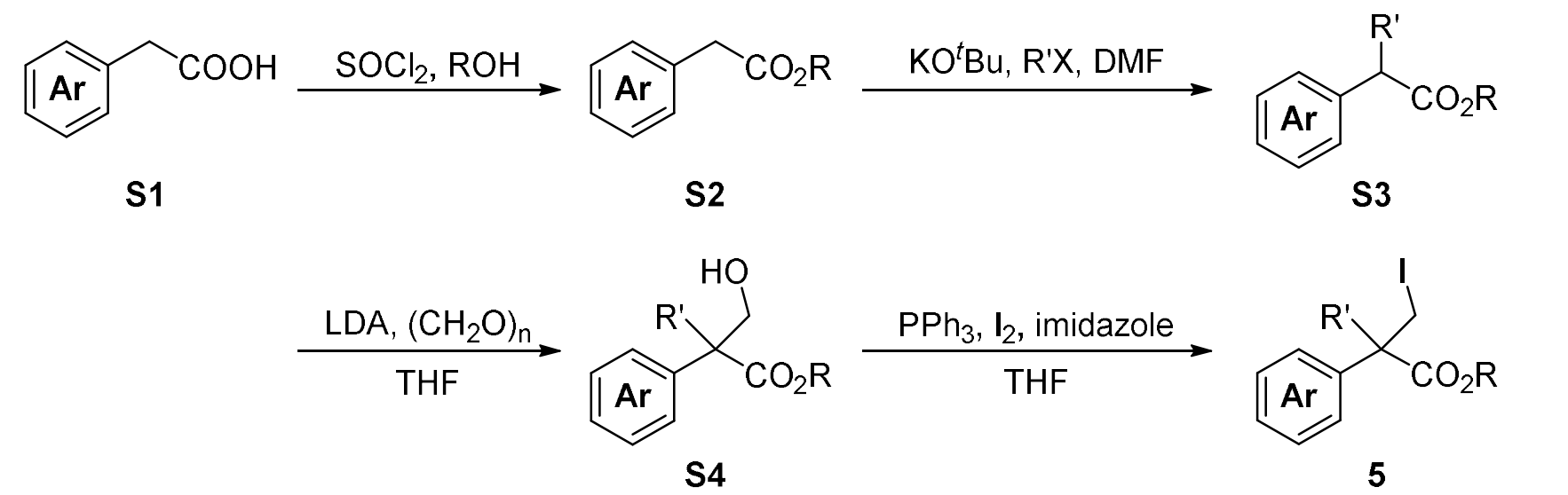

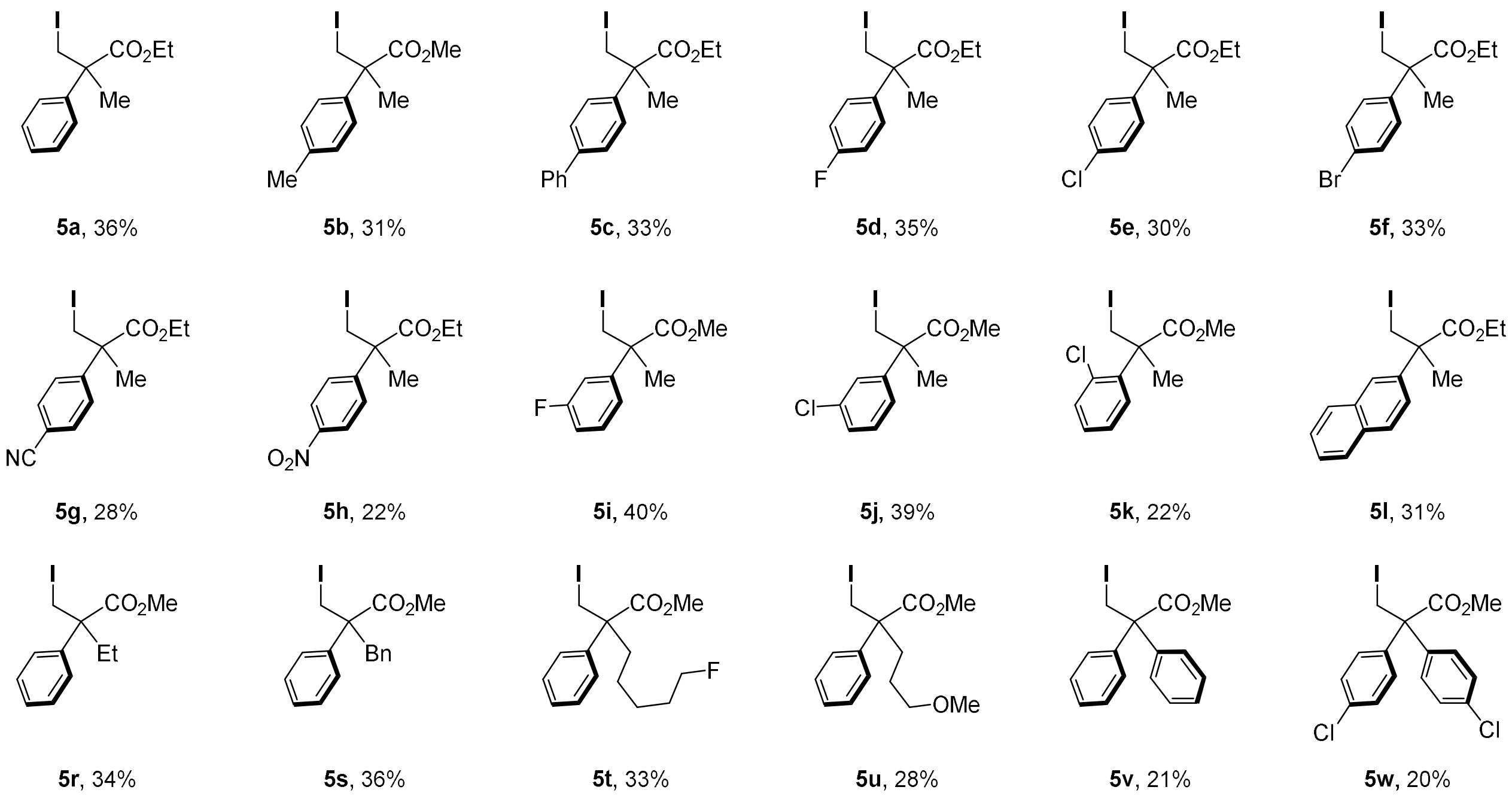

Step 1: To a solution of acid **S1** in ROH (1 M) at 0 ºC was added $SOCl_2$ (2.5 equiv) slowly. The mixture was stirred at 0 ºC for 30 min, then at room temperature for 2 h. Then, the mixture was concentrated under reduced pressure to afford **S2** which was directly used for the next step without further purification.

Step 2: To a solution of ester **S2** in DMF (1 M) at 0 ºC was added KO*^t*Bu (2.0 equiv) slowly. The mixture was stirred at 0 ºC for 30 min and the alkyl halide (1.2 equiv) was added dropwise. The reaction mixture was allowed to warm to room temperature and stirred overnight. Then, the mixture was quenched with dilute HCl (2 M) and extracted with ethyl acetate (twice). The extracts were combined, dried over Na_2SO_4, filtered and concentrated under reduced pressure to give **S3** which was used for the next step without further purification.

Step 3: To a solution of **S3** in THF (1 M) at -78 ºC was added LDA (1.0 M solution in THF, 1.3 equiv) dropwise. The mixture was stirred at -78 ºC for 1 h and the paraformaldehyde (1.5 equiv) was added slowly. The reaction mixture was allowed to warm to room temperature and stirred overnight. Then, the mixture was quenched with dilute HCl (2 M) and extracted with ethyl acetate (twice). The extracts were combined, dried over Na_2SO_4, filtered and concentrated under reduced pressure to give **S4** which was used for the next step without further purification.

Step 4: To a solution of triphenylphosphine (1.3 equiv), imidazole (1.3 equiv) and iodine (1.3 equiv) in THF (1 M) at 0 ºC was added **S4** (1.0 M solution in THF) dropwise. The reaction mixture was allowed to warm to 60 ºC and stirred overnight. Then, the mixture was quenched with $Na_2S_2O_3$ solution (2 M) and extracted with ethyl acetate (twice). The extracts were combined, dried over Na_2SO_4, filtered and concentrated under reduced pressure. The residue was purified by silica gel flash column chromatography (eluent: hexanes / ethyl acetate = 20:1 → 10:1) to give the desired product **5**.

General Procedure B:

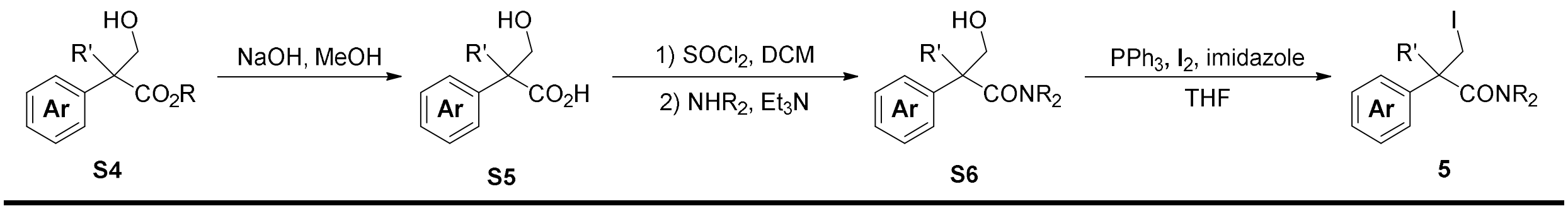

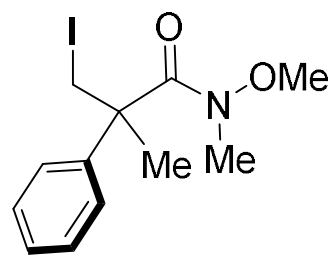

5m, 42%

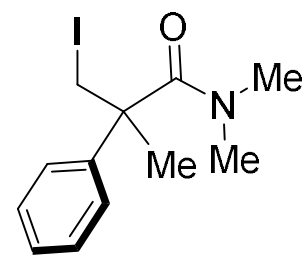

5n, 45%

Step 1: To a solution of the ester **S4** (1.0 equiv) in MeOH (1 M) was added NaOH (2.0 equiv) at room temperature. The reaction mixture was allowed to warm to 60 ºC and stirred overnight. Then, the mixture was quenched with dilute HCl (2 M) and extracted with ethyl acetate (twice). The extracts were combined, dried over Na_2SO_4, filtered and concentrated under reduced pressure to give **S5** which was used for the next step without further purification.

Step 2: To a solution of the acid **S5** (1.0 equiv) in DCM (1 M) was added dropwise $SOCl_2$ (1.5 equiv) followed by 3 drops of DMF at 0 °C. The reaction mixture was stirred at rt for 1 h. The reaction mixture was then concentrated. The resulting acid chloride was redissolved in dichloromethane (0.5 M) and amine (1.1 equiv) and triethylamine (1.5 equiv) were added. The resulting mixture was stirred at rt overnight. Then, the mixture was quenched with H_2O (5 mL) and extracted with DCM (twice). The extracts were combined, dried over Na_2SO_4, filtered and concentrated under reduced pressure to give **S6** which was used for the next step without further purification.

Step 3: To a solution of triphenylphosphine (1.3 equiv), imidazole (1.3 equiv) and iodine (1.3 equiv) in THF (1 M) at 0 ºC was added **S6** (1.0 M solution in THF) dropwise. The reaction mixture was allowed to warm to 60 ºC and stirred overnight. Then, the mixture was quenched with $Na_2S_2O_3$ solution (2 M) and extracted with ethyl acetate (twice). The extracts were combined, dried over Na_2SO_4, filtered and concentrated under reduced pressure. The residue was purified by silica gel flash column chromatography (eluent: hexanes / ethyl acetate = 20:1 → 10:1) to give the desired product **5**.

General Procedure C:

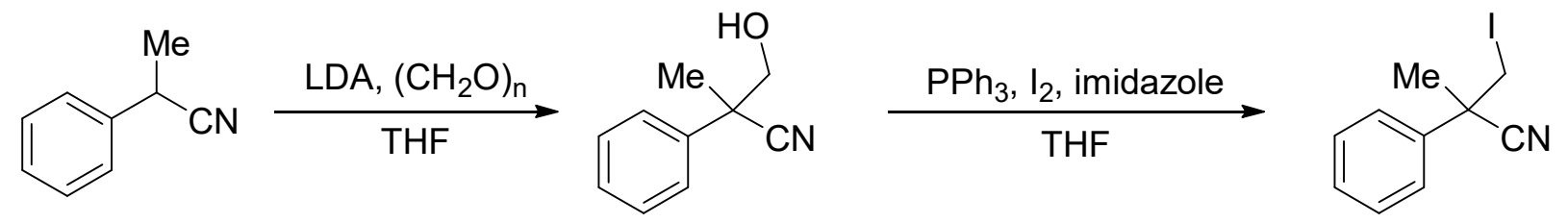

S7 **S8** **5o**, 50%

Step 1: To a solution of **S7** in THF (1 M) at -78 ºC was added LDA (1.0 M solution in THF, 1.3 equiv) dropwise. The mixture was stirred at -78 ºC for 1 h and the paraformaldehyde (1.5 equiv) was added slowly. The reaction mixture was allowed to warm to room temperature and stirred overnight. Then, the mixture was quenched with dilute HCl (2 M) and extracted with ethyl acetate (twice). The extracts were combined, dried over Na_2SO_4, filtered and concentrated under reduced pressure to give **S8** which was used for the next step without further purification.

Step 2: To a solution of triphenylphosphine (1.3 equiv), imidazole (1.3 equiv) and iodine (1.3 equiv) in THF (1 M) at 0 ºC was added **S8** (1.0 M solution in THF) dropwise. The reaction mixture was allowed to warm to 60 ºC and stirred overnight. Then, the mixture was quenched with $Na_2S_2O_3$ solution (2 M) and extracted with ethyl acetate (twice). The extracts were combined, dried over Na_2SO_4, filtered and concentrated under reduced pressure. The residue was purified by silica gel flash column chromatography (eluent: hexanes / ethyl acetate = 20:1 → 10:1) to give the desired product **5o**.

General Procedure D:

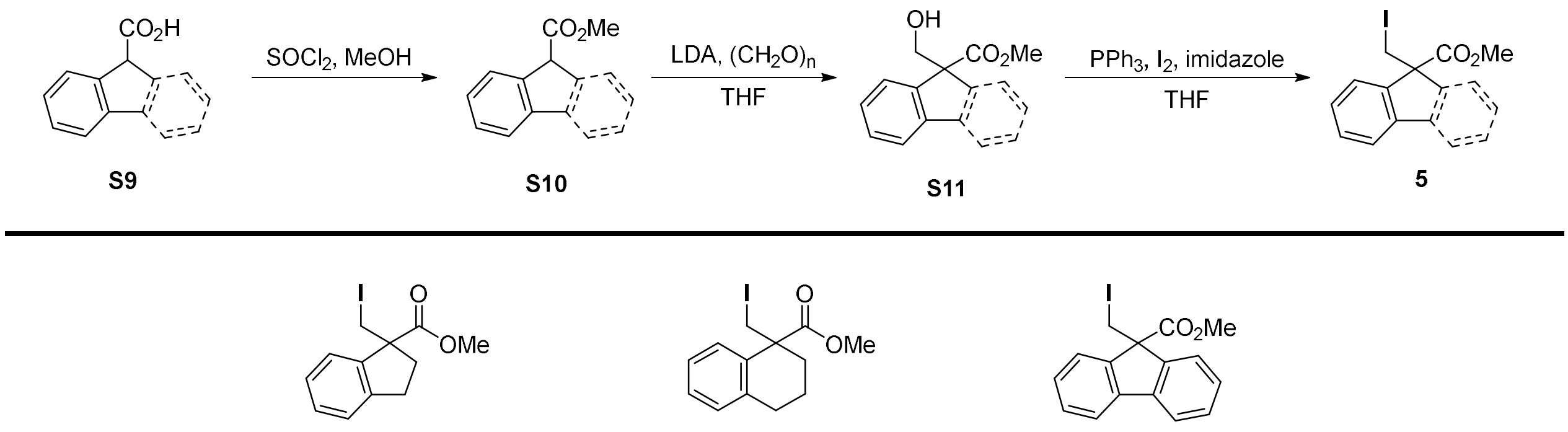

S9 **S10** **S11** **5**

5x, 50% **5y**, 52% **5z**, 48%

Step 1: To a solution of acid **S9** in MeOH (1 M) at 0 ºC was added $SOCl_2$ (2.5 equiv) slowly. The mixture was stirred at 0 ºC for 30 min, then at room temperature for 2 h. Then, the mixture was concentrated under reduced pressure to generate **S10** which was directly used for the next step without further purification.

Step 2: To a solution of **S10** in THF (1 M) at -78 ºC was added LDA (1.0 M solution in THF, 1.3 equiv) dropwise. The mixture was stirred at -78 ºC for 1 h and the paraformaldehyde (1.5 equiv) was added slowly.

The reaction mixture was allowed to warm to room temperature and stirred overnight. Then, the mixture was quenched with dilute HCl (2 M) and extracted with ethyl acetate (twice). The extracts were combined, dried over Na_2SO_4, filtered and concentrated under reduced pressure to give **S11** which was used for the next step without further purification.

Step 3: To a solution of triphenylphosphine (1.3 equiv), imidazole (1.3 equiv) and iodine (1.3 equiv) in THF (1 M) at 0 ºC was added **S11** (1.0 M solution in THF) dropwise. The reaction mixture was allowed to warm to 60 ºC and stirred overnight. Then, the mixture was quenched with $Na_2S_2O_3$ solution (2 M) and extracted with ethyl acetate (twice). The extracts were combined, dried over Na_2SO_4, filtered and concentrated under reduced pressure. The residue was purified by silica gel flash column chromatography (eluent: hexanes / ethyl acetate = 20:1 → 10:1) to give the desired product **5**.

General Procedure E:

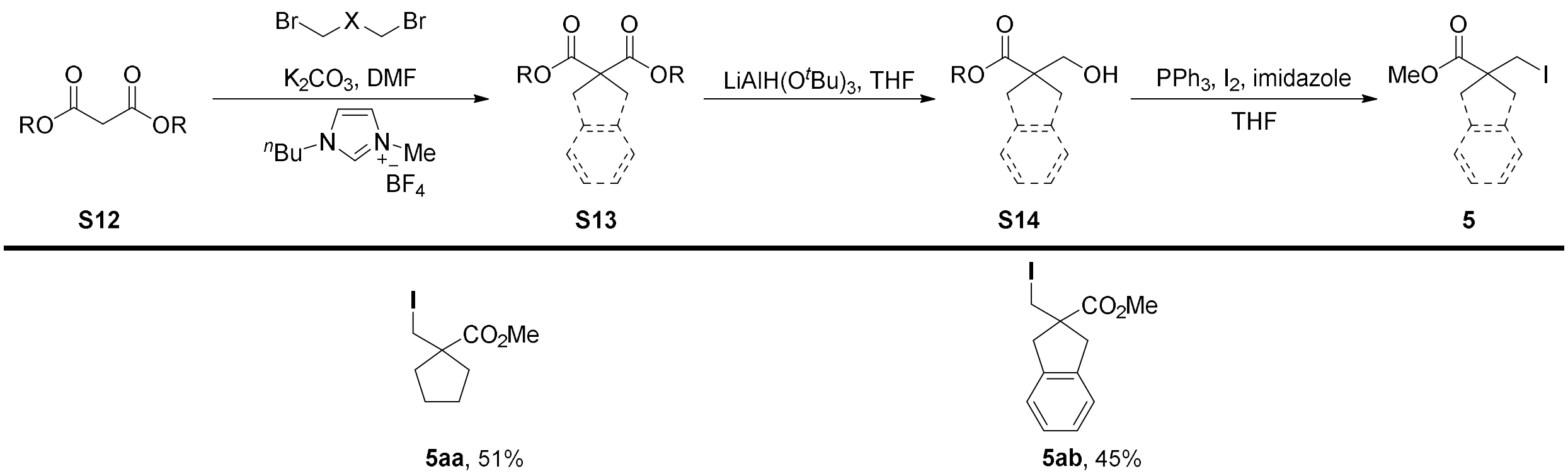

Step 1: To a solution of ester **S12,** alkyl dibromide (1.2 equiv) and K_2CO_3 (2.0 equiv) in DMF (1 M) at 0 ºC was added 1-butyl-3-methylimidazolium tetrafluoroborate (10 mol%) slowly. The mixture was stirred at room temperature for 15 h. Then, the mixture was quenched with H_2O and extracted with ethyl acetate (twice). The extracts were combined, dried over Na_2SO_4, filtered and concentrated under reduced pressure to give **S13** which was used for the next step without further purification.

Step 2: To a solution of **S13** in THF (1 M) at -78 ºC was added $LiAlH(O^tBu)_3$ (1.0 M solution in THF, 1.2 equiv) dropwise. The mixture was stirred at -78 ºC for 2 h and allowed to warm to room temperature and stirred overnight. Then, the mixture was quenched with dilute HCl (2 M) and extracted with ethyl acetate (twice). The extracts were combined, dried over Na_2SO_4, filtered and concentrated under reduced pressure to give **S14** which was used for the next step without further purification.

Step 3: To a solution of triphenylphosphine (1.3 equiv), imidazole (1.3 equiv) and iodine (1.3 equiv) in THF (1 M) at 0 ºC was added **S14** (1.0 M solution in THF) dropwise. The reaction mixture was allowed to warm to 60 ºC and stirred overnight. Then, the mixture was quenched with $Na_2S_2O_3$ solution (2 M) and extracted with ethyl acetate (twice). The extracts were combined, dried over Na_2SO_4, filtered and concentrated under

reduced pressure. The residue was purified by silica gel flash column chromatography (eluent: hexanes / ethyl acetate = 20:1 → 10:1) to give the desired product **5**.

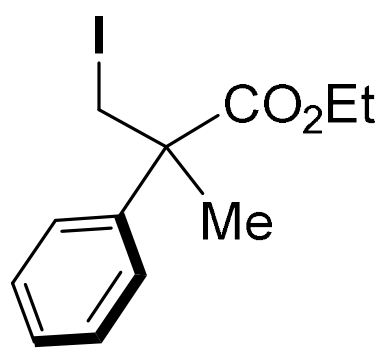

5a. Colorless oil.

Analytical data for **5a**:

^{1}H NMR (400 MHz, $CDCl_3$) δ 7.28 – 7.26 (m, 4H), 7.23 – 7.18 (m, 1H), 4.12 (q, *J* = 7.1 Hz, 2H), 3.80 (d, *J* = 9.8 Hz, 1H), 3.47 (d, *J* = 9.9 Hz, 1H), 1.66 (s, 3H), 1.16 (t, *J* = 7.1 Hz, 3H).

^{13}C NMR (101 MHz, $CDCl_3$) δ 172.8, 140.9, 128.6, 127.6, 125.8, 61.5, 50.7, 24.4, 16.1, 14.0.

IR (neat) ν_{max} (cm^{-1}) = 2979, 2870, 1728, 1559, 1506, 1496, 1457, 1445, 1419, 1377, 1290, 1266, 1223, 1152, 1092, 1070, 1045, 1017, 941, 887, 860, 763, 728, 696, 647.

HRMS (Sicrit plasma/LTQ-Orbitrap) m/z: $[M + H]^+$ Calcd for $C_{12}H_{16}IO_2^+$ 319.0190; Found 319.0188.

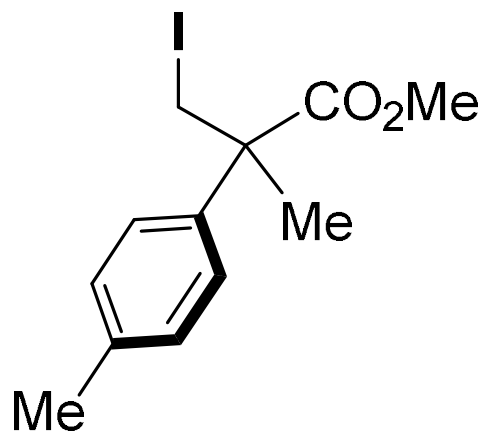

5b. Colorless oil.

Analytical data for **5b**:

^{1}H NMR (800 MHz, $CDCl_3$) δ 7.22 (d, *J* = 8.0 Hz, 2H), 7.15 (d, *J* = 8.1 Hz, 2H), 3.86 (d, *J* = 9.9 Hz, 1H), 3.71 (s, 3H), 3.52 (d, *J* = 9.9 Hz, 1H), 2.33 (s, 3H), 1.73 (s, 3H).

^{13}C NMR (201 MHz, $CDCl_3$) δ 173.5, 137.7, 137.5, 129.4, 125.6, 52.6, 50.5, 24.4, 21.0, 16.2.

IR (neat) ν_{max} (cm^{-1}) = 2949, 1734, 1511, 1433, 1376, 1290, 1263, 1200, 1155, 1116, 1088, 1072, 1043, 1019, 984, 922, 874, 815, 772, 731, 612.

HRMS (ESI/QTOF) m/z: $[M + Na]^+$ Calcd for $C_{12}H_{15}INaO_2^+$ 341.0009; Found 341.0012.

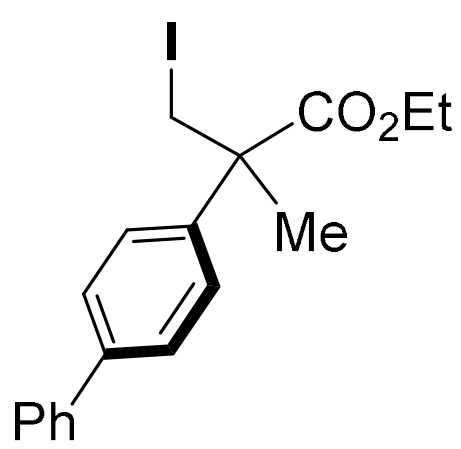

5c. Colorless oil.

Analytical data for **5c**:

^{1}H NMR (400 MHz, $CDCl_3$) δ 7.62 – 7.55 (m, 4H), 7.48 – 7.39 (m, 4H), 7.39 – 7.31 (m, 1H), 4.23 (q, *J* = 7.1 Hz, 2H), 3.92 (d, *J* = 9.8 Hz, 1H), 3.58 (d, *J* = 9.8 Hz, 1H), 1.78 (s, 3H), 1.27 (t, *J* = 7.1 Hz, 3H).

^{13}C NMR (101 MHz, $CDCl_3$) δ 172.8, 140.4, 140.3, 139.8, 128.8, 127.5, 127.3, 127.0, 126.3, 61.6, 50.6, 24.5, 16.0, 14.1.

IR (neat) ν_{max} (cm^{-1}) = 2977, 2869, 1730, 1487, 1457, 1378, 1289, 1275, 1258, 1225, 1151, 1117, 1087, 1074, 1045, 1020, 1007, 860, 839, 759, 734, 697, 643, 635.

HRMS (ESI/QTOF) m/z: $[M + Na]^+$ Calcd for $C_{18}H_{19}INaO_2^+$ 417.0322; Found 417.0324.

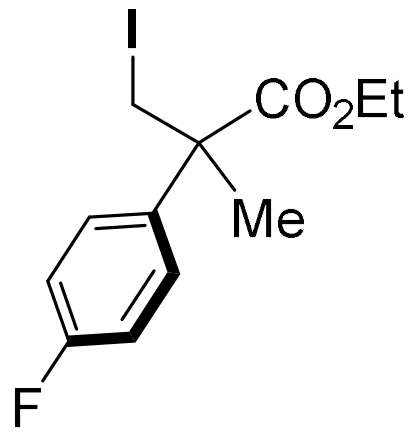

5d. Colorless oil.

Analytical data for **5d**:

^{1}H NMR (400 MHz, $CDCl_3$) δ 7.37 – 7.28 (m, 2H), 7.08 – 6.97 (m, 2H), 4.18 (q, *J* = 7.1 Hz, 2H), 3.83 (d, *J* = 10.0 Hz, 1H), 3.53 (d, *J* = 9.9 Hz, 1H), 1.73 (s, 3H), 1.23 (t, *J* = 7.1 Hz, 3H).

^{13}C NMR (101 MHz, $CDCl_3$) δ 172.6, 162.0 (d, *J* = 247.1 Hz), 136.6 (d, *J* = 3.2 Hz), 127.6 (d, *J* = 8.1 Hz), 115.5 (d, *J* = 21.4 Hz), 61.6, 50.1, 24.4, 16.0, 14.0.

^{19}F{^{1}H} NMR (377 MHz, $CDCl_3$) δ -114.69.

^{19}F NMR (377 MHz, $CDCl_3$) δ -114.65 – -114.72 (m).

IR (neat) ν_{max} (cm^{-1}) = 2988, 2869, 1727, 1602, 1508, 1458, 1377, 1295, 1264, 1235, 1166, 1153, 1105, 1084, 1070, 1044, 1014, 860, 834, 817, 772, 736, 611.

HRMS (nanochip-ESI/LTQ-Orbitrap) m/z: $[M + Na]^+$ Calcd for $C_{12}H_{14}FINaO_2^+$ 358.9915; Found 358.9907.

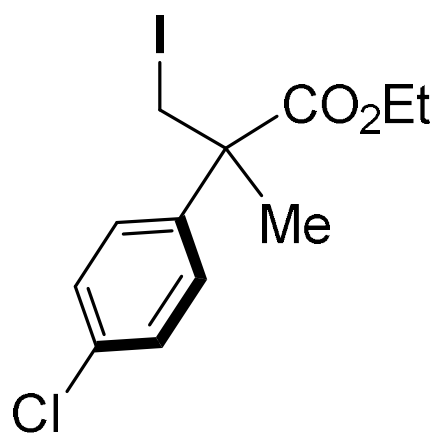

5e. Colorless oil.

Analytical data for **5e**:

^{1}H NMR (400 MHz, $CDCl_3$) δ 7.26 – 7.22 (m, 2H), 7.22 – 7.17 (m, 2H), 4.11 (q, *J* = 7.1 Hz, 2H), 3.74 (d, *J* = 10.0 Hz,1H), 3.46 (d, *J* = 10.0 Hz, 1H), 1.65 (s, 3H), 1.16 (t, *J* = 7.1 Hz, 3H).

^{13}C NMR (101 MHz, $CDCl_3$) δ 172.3, 139.3, 133.5, 128.8, 127.3, 61.7, 50.3, 24.3, 15.6, 14.0.

IR (neat) ν_{max} (cm^{-1}) = 2980, 2869, 1729, 1492, 1458, 1376, 1286, 1226, 1153, 1095, 1044, 1012, 860, 827, 753, 722.

HRMS (Sicrit plasma/LTQ-Orbitrap) m/z: $[M + H]^+$ Calcd for $C_{12}H_{15}ClIO_2^+$ 352.9800; Found 352.9800.

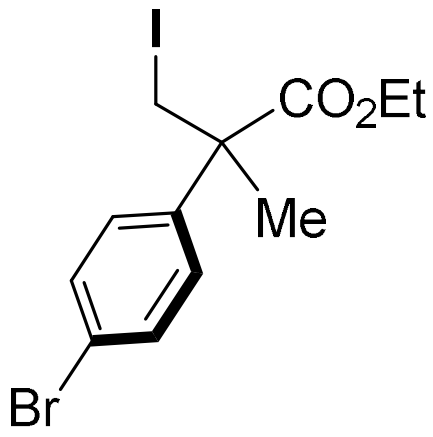

5f. Colorless oil.

Analytical data for **5f**:

^{1}H NMR (400 MHz, $CDCl_3$) δ 7.47 (d, *J* = 8.6 Hz, 2H), 7.22 (d, *J* = 8.7 Hz, 2H), 4.18 (q, *J* = 7.1 Hz, 2H), 3.81 (d, *J* = 10.0 Hz, 1H), 3.53 (d, *J* = 10.0 Hz, 1H), 1.71 (s, 3H), 1.23 (t, *J* = 7.1 Hz, 3H).

^{13}C NMR (101 MHz, $CDCl_3$) δ 172.3, 139.9, 131.7, 127.7, 121.7, 61.7, 50.3, 24.2, 15.5, 14.0.

IR (neat) ν_{max} (cm^{-1}) = 2978, 1726, 1489, 1462, 1397, 1376, 1285, 1258, 1224, 1183, 1153, 1128, 1112, 1081, 1043, 1007, 934, 860, 823, 770, 747, 716, 666.

HRMS (ESI/QTOF) m/z: $[M + H]^+$ Calcd for $C_{12}H_{15}BrIO_2^+$ 396.9295; Found 396.9281.

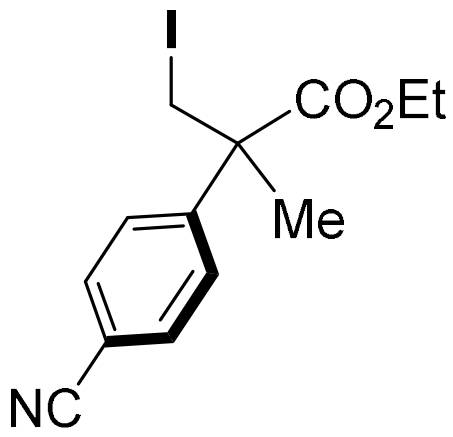

5g. White solid.

Analytical data for **5g**:

m.p. = 90 ~ 92 °C.

^{1}H NMR (400 MHz, $CDCl_3$) δ 7.64 (d, *J* = 8.5 Hz, 2H), 7.45 (d, *J* = 8.6 Hz, 2H), 4.18 (q, *J* = 7.1 Hz, 2H), 3.78 (d, *J* = 10.1 Hz, 1H), 3.59 (d, *J* = 10.1 Hz, 1H), 1.74 (s, 3H), 1.22 (t, *J* = 7.1 Hz, 3H).

^{13}C NMR (101 MHz, $CDCl_3$) δ 171.7, 146.1, 132.4, 126.9, 118.4, 111.6, 62.0, 50.9, 24.1, 14.6, 14.0.

IR (neat) ν_{max} (cm^{-1}) = 2980, 2229, 1726, 1606, 1504, 1458, 1407, 1378, 1291, 1265, 1229, 1155, 1123, 1087, 1071, 1045, 1017, 888, 839, 769, 740, 715, 640.

HRMS (ESI/QTOF) m/z: $[M + Na]^+$ Calcd for $C_{13}H_{14}INNaO_2^+$ 365.9961; Found 365.9967.

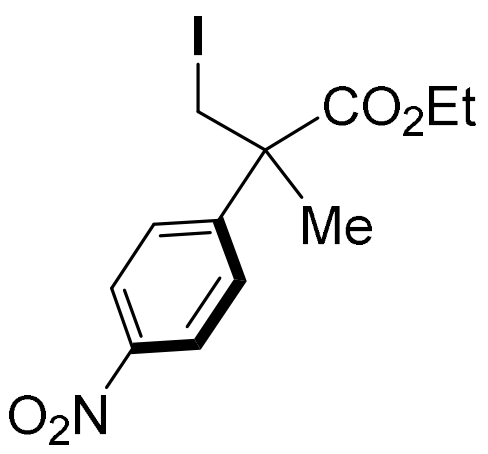

5h. White solid.

Analytical data for **5h**:

m.p. = 102 ~ 104 °C.

^{1}H NMR (400 MHz, $CDCl_3$) δ 8.22 – 8.19 (m, 2H), 7.53 – 7.49 (m, 2H), 4.20 (q, *J* = 7.1 Hz, 2H), 3.80 (d, *J* = 10.2 Hz, 1H), 3.63 (d, *J* = 10.2 Hz, 1H), 1.78 (s, 3H), 1.23 (t, *J* = 7.1 Hz, 3H).

^{13}C NMR (101 MHz, $CDCl_3$) δ 171.6, 148.1, 147.2, 127.2, 123.7, 62.1, 50.9, 24.3, 14.6, 14.0.

IR (neat) ν_{max} (cm^{-1}) = 2988, 2869, 1724, 1603, 1517, 1464, 1427, 1379, 1360, 1343, 1299, 1229, 1204, 1192, 1171, 1143, 1130, 1086, 1045, 1012, 933, 894, 853, 831, 805, 762, 737, 702, 681, 639.

HRMS (Sicrit plasma/LTQ-Orbitrap) m/z: $[M + H]^+$ Calcd for $C_{12}H_{15}INO_4^+$ 364.0040; Found 364.0040.

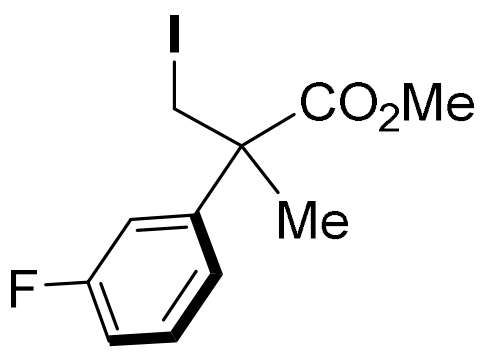

5i. Colorless oil.

Analytical data for **5i**:

^{1}H NMR (400 MHz, $CDCl_3$) δ 7.31 (td, *J* = 8.1, 6.1 Hz, 1H), 7.11 (ddd, *J* = 7.9, 1.9, 0.9 Hz, 1H), 7.05 (dt, *J* = 10.5, 2.2 Hz, 1H), 6.99 (tdd, *J* = 8.3, 2.5, 0.9 Hz, 1H), 3.83 (d, *J* = 10.0 Hz, 1H), 3.73 (s, 3H), 3.54 (d, *J* = 10.0 Hz, 1H), 1.73 (s, 3H).

^{13}C NMR (101 MHz, $CDCl_3$) δ 172.8, 162.8 (d, *J* = 246.5 Hz), 143.2 (d, *J* = 7.0 Hz), 130.1 (d, *J* = 8.2 Hz), 121.6 (d, *J* = 3.0 Hz), 114.6 (d, *J* = 21.0 Hz), 113.3 (d, *J* = 22.9 Hz), 52.8, 50.7 (d, *J* = 1.6 Hz), 24.3, 15.2.

^{19}F{^{1}H} NMR (377 MHz, $CDCl_3$) δ -111.85.

^{19}F NMR (377 MHz, $CDCl_3$) δ -111.82 – -111.88 (m).

IR (neat) ν_{max} (cm^{-1}) = 2951, 1730, 1612, 1588, 1488, 1434, 1378, 1264, 1243, 1211, 1157, 1121, 1096, 982, 913, 868, 816, 785, 772, 726, 693, 653.

HRMS (ESI/QTOF) m/z: $[M + Na]^+$ Calcd for $C_{11}H_{12}FINaO_2^+$ 344.9758; Found 344.9760.

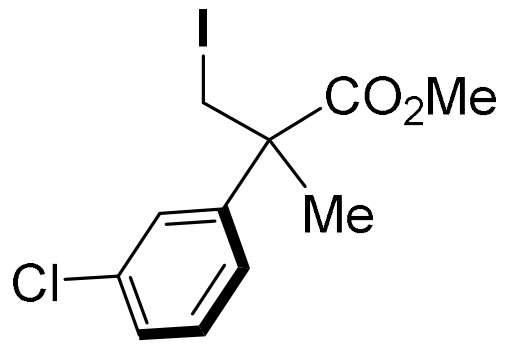

5j. Colorless oil.

Analytical data for **5j**:

¹H NMR (400 MHz, $CDCl_3$) δ 7.24 (brs, 1H), 7.22 – 7.18 (m, 2H), 7.16 – 7.12 (m, 1H), 3.75 (d, *J* = 10.0 Hz, 1H), 3.65 (s, 3H), 3.46 (d, *J* = 10.0 Hz, 1H), 1.66 (s, 3H).

¹³C NMR (101 MHz, $CDCl_3$) δ 172.8, 142.7, 134.6, 129.9, 127.9, 126.2, 124.1, 52.8, 50.7, 24.3, 15.2.

IR (neat) ν_{max} (cm^{-1}) = 2988, 2869, 1731, 1593, 1571, 1458, 1433, 1420, 1378, 1284, 1228, 1197, 1155, 1091, 1073, 1046, 998, 982, 928, 884, 817, 786, 766, 705, 688, 649.

HRMS (ESI/QTOF) m/z: $[M + Na]^+$ Calcd for $C_{11}H_{12}ClINaO_2^+$ 360.9463; Found 360.9472.

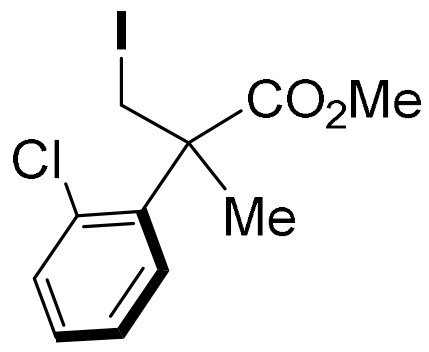

5k. Colorless oil.

Analytical data for **5k**:

¹H NMR (400 MHz, $CDCl_3$) δ 7.32 – 7.15 (m, 4H), 4.14 (dq, *J* = 10.5, 1.0 Hz, 1H), 3.72 (d, *J* = 10.5 Hz, 1H), 3.64 (s, 3H), 1.68 (s, 3H).

¹³C NMR (101 MHz, $CDCl_3$) δ 173.3, 138.7, 133.2, 130.6, 128.99, 128.95, 126.5, 52.9, 50.0, 25.8, 14.5.

IR (neat) ν_{max} (cm^{-1}) = 2988, 2869, 1731, 1593, 1570, 1458, 1433, 1420, 1378, 1284, 1228, 1197, 1144, 1091, 1074, 1046, 998, 982, 928, 884, 817, 786, 766, 705, 688, 649.

HRMS (ESI/QTOF) m/z: $[M + H]^+$ Calcd for $C_{11}H_{13}ClIO_2^+$ 338.9643; Found 338.9650.

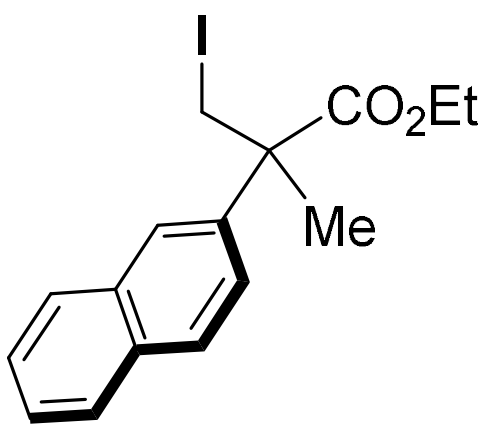

5l. Colorless oil.

Analytical data for **5l**:

¹H NMR (400 MHz, $CDCl_3$) δ 7.85 – 7.78 (m, 4H), 7.52 – 7.44 (m, 3H), 4.21 (q, *J* = 7.1 Hz, 2H), 3.98 (d, *J* = 9.9 Hz, 1H), 3.67 (d, *J* = 9.9 Hz, 1H), 1.85 (s, 3H), 1.23 (t, *J* = 7.1 Hz, 3H).

^{13}C NMR (101 MHz, $CDCl_3$) δ 172.8, 138.2, 133.1, 132.5, 128.4, 128.2, 127.5, 126.4, 126.3, 124.8, 123.8, 61.6, 50.8, 24.5, 15.9, 14.0.

IR (neat) ν_{max} (cm^{-1}) = 2978, 1727, 1506, 1460, 1377, 1271, 1222, 1152, 1128, 1112, 1092, 1065, 1044, 1017, 949, 894, 857, 814, 775, 747, 712, 657, 637, 632, 623, 603.

HRMS (ESI/QTOF) m/z: $[M + Na]^+$ Calcd for $C_{16}H_{17}INaO_2^+$ 391.0165; Found 391.0169.

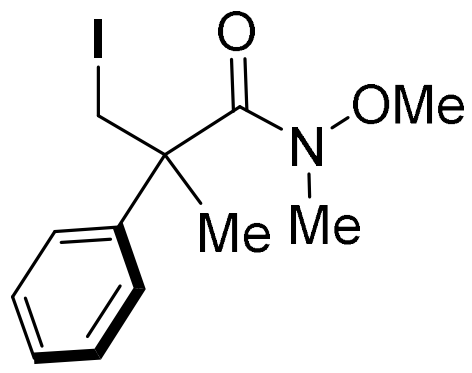

5m. Colorless oil.

Analytical data for **5m**:

^{1}H NMR (400 MHz, $CDCl_3$) δ 7.41 – 7.34 (m, 2H), 7.31 – 7.25 (m, 3H), 4.04 (d, J = 9.8 Hz, 1H), 3.65 (d, J = 9.8 Hz, 1H), 3.17 (s, 3H), 2.68 (s, 3H), 1.73 (s, 3H).

^{13}C NMR (101 MHz, $CDCl_3$) δ 174.0, 142.4, 128.4, 127.0, 125.9, 59.1, 50.2, 33.3, 24.0, 18.4.

IR (neat) ν_{max} (cm^{-1}) = 2977, 1642, 1497, 1446, 1361, 1142, 1069, 992, 766, 732, 699, 637.

HRMS (ESI/QTOF) m/z: $[M + Na]^+$ Calcd for $C_{12}H_{16}INNaO_2^+$ 356.0118; Found 356.0123.

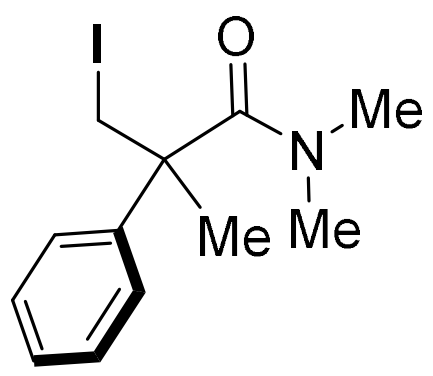

5n. Colorless oil.

Analytical data for **5n**:

^{1}H NMR (400 MHz, $CDCl_3$) δ 7.31 – 7.24 (m, 2H), 7.23 – 7.17 (m, 1H), 7.16 – 7.10 (m, 2H), 3.79 (dd, J = 10.0, 1.4 Hz, 1H), 3.52 (d, J = 10.0 Hz, 1H), 2.87 (br, 3H), 2.44 (br, 3H), 1.65 (d, J = 1.4 Hz, 3H).

^{13}C NMR (101 MHz, $CDCl_3$) δ 172.6, 141.9, 128.7, 127.2, 125.6, 49.5, 38.1, 37.1, 23.4, 22.0.

IR (neat) ν_{max} (cm^{-1}) = 2979, 2932, 2858, 1666, 1489, 1400, 1364, 1273, 1209, 1178, 1142, 1088, 1075, 1014, 949, 932, 882, 839, 805, 730, 678.

HRMS (ESI/QTOF) m/z: $[M + H]^+$ Calcd for $C_{12}H_{17}INO^+$ 318.0349; Found 318.0360.

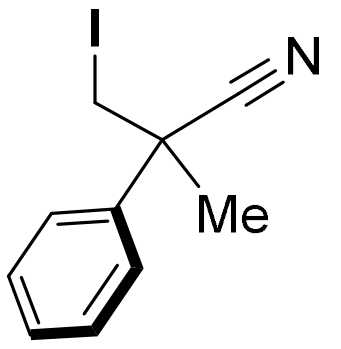

5o. Colorless oil.

Analytical data for **5o**:

^{1}H NMR (400 MHz, $CDCl_3$) δ 7.50 – 7.35 (m, 5H), 3.57 (d, *J* = 10.5 Hz, 1H), 3.53 (d, *J* = 10.5 Hz, 1H), 1.90 (s, 3H).

^{13}C NMR (101 MHz, $CDCl_3$) δ 138.0, 129.1, 128.7, 125.5, 121.6, 43.8, 26.9, 13.6.

IR (neat) ν_{max} (cm^{-1}) = 2988, 2869, 1733, 1495, 1446, 1378, 1242, 1213, 1181, 1143, 1072, 1044, 760, 696, 628, 613, 603.

HRMS (Sicrit plasma/LTQ-Orbitrap) m/z: $[M + H]^+$ Calcd for $C_{10}H_{11}IN^+$ 271.9931; Found 271.9931.

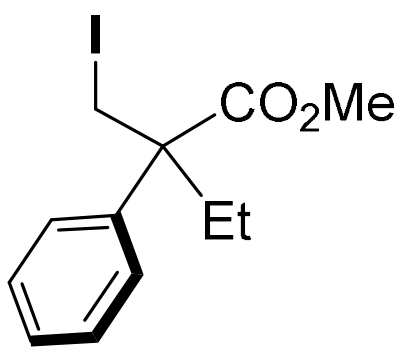

5r. Colorless oil.

Analytical data for **5r**:

^{1}H NMR (400 MHz, $CDCl_3$) δ 7.29-7.24 (m, 2H), 7.22 – 7.15 (m, 3H), 3.81 (d, *J* = 10.2 Hz, 1H), 3.65 (d, *J* = 10.1 Hz, 1H), 3.60 (s, 3H), 2.28 – 2.06 (m, 2H), 0.69 (t, *J* = 7.4 Hz, 3H).

^{13}C NMR (101 MHz, $CDCl_3$) δ 173.0, 140.2, 128.5, 127.4, 126.3, 54.4, 52.5, 28.7, 13.1, 8.2.

IR (neat) ν_{max} (cm^{-1}) = 2971, 2875, 1725, 1495, 1446, 1433, 1381, 1299, 1252, 1222, 1147, 1127, 1098, 1032, 1017, 1002, 819, 805, 764, 734, 696.

HRMS (Sicrit plasma/LTQ-Orbitrap) m/z: $[M + H]^+$ Calcd for $C_{12}H_{16}IO_2^+$ 319.0190; Found 319.0189.

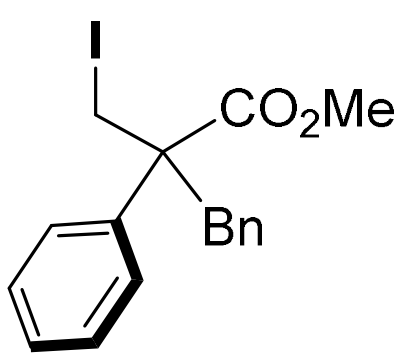

5s. Colorless oil.

Analytical data for **5s**:

^{1}H NMR (400 MHz, $CDCl_3$) δ 7.38 – 7.27 (m, 3H), 7.24 – 7.10 (m, 5H), 6.98 – 6.94 (m, 2H), 3.73 (d, *J* = 9.9 Hz, 1H), 3.72 (s, 3H), 3.60 (d, *J* = 9.9 Hz, 1H), 3.58 (d, *J* = 13.5 Hz, 1H), 3.40 (d, *J* = 13.5 Hz, 1H).

^{13}C NMR (101 MHz, $CDCl_3$) δ 172.7, 140.2, 135.9, 130.0, 128.5, 128.1, 127.6, 127.0, 126.3, 55.6, 52.6, 40.9, 13.1.

IR (neat) ν_{max} (cm^{-1}) = 2988, 2869, 1734, 1496, 1446, 1255, 1178, 1154, 1079, 1031, 1002, 874, 817, 789, 763, 734, 696, 623, 608.

HRMS (Sicrit plasma/LTQ-Orbitrap) m/z: $[M + H]^+$ Calcd for $C_{17}H_{18}IO_2^+$ 381.0346; Found 381.0346.

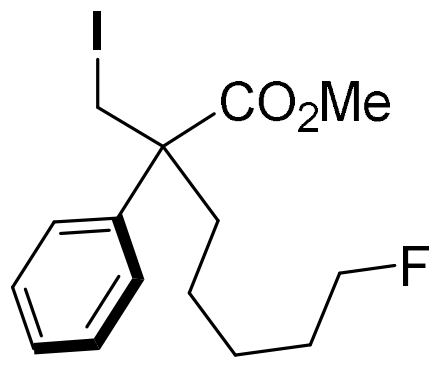

5t. Colorless oil.

Analytical data for **5t**:

^{1}H NMR (400 MHz, $CDCl_3$) δ 7.31 – 7.24 (m, 2H), 7.24 – 7.16 (m, 3H), 4.40 (t, *J* = 6.1 Hz, 1H), 4.28 (t, *J* = 6.1 Hz, 1H), 3.82 (d, *J* = 10.1 Hz, 1H), 3.65 (d, *J* = 10.2 Hz, 1H), 3.61 (s, 3H), 2.19 – 2.07 (m, 2H), 1.69 – 1.54 (m, 2H), 1.38 (dq, *J* = 8.6, 6.9 Hz, 2H), 1.15 – 0.95 (m, 2H).

^{13}C NMR (101 MHz, $CDCl_3$) δ 172.9, 140.1, 128.6, 127.5, 126.1, 83.9 (d, *J* = 164.5 Hz), 54.0, 52.6, 35.7, 30.1 (d, *J* = 19.6 Hz), 25.3 (d, *J* = 5.4 Hz), 23.3, 13.5.

^{19}F{^{1}H} NMR (377 MHz, $CDCl_3$, decoupling) δ -218.37.

^{19}F NMR (377 MHz, $CDCl_3$, no decoupling) δ -218.37 (tt, *J* = 46.8, 25.1 Hz).

IR (neat) ν_{max} (cm^{-1}) = 2950, 2868, 1734, 1496, 1457, 1447, 1435, 1251, 1229, 1157, 1063, 1013, 940, 903, 822, 777, 756, 731, 697, 669.

HRMS (Sicrit plasma/LTQ-Orbitrap) m/z: $[M + H]^+$ Calcd for $C_{15}H_{21}FIO_2^+$ 379.0565; Found 379.0565.

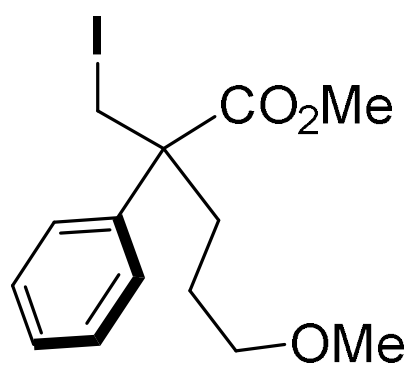

5u. Colorless oil.

Analytical data for **5u**:

^{1}H NMR (400 MHz, $CDCl_3$) δ 7.30 – 7.24 (m, 2H), 7.23 – 7.16 (m, 3H), 3.83 (d, *J* = 10.2 Hz, 1H), 3.66 (d, *J* = 10.1 Hz, 1H), 3.62 (s, 3H), 3.31 (t, *J* = 6.6 Hz, 2H), 3.24 (s, 3H), 2.17 (dd, *J* = 9.2, 7.5 Hz, 2H), 1.40 – 1.20 (m, 2H).

^{13}C NMR (100 MHz, $CDCl_3$) δ 172.9, 140.0, 128.6, 127.5, 126.2, 72.5, 58.5, 54.0, 52.6, 32.6, 24.1, 13.3.

IR (neat) ν_{max} (cm^{-1}) = 2988, 2979, 2869, 1727, 1489, 1459, 1396, 1377, 1364, 1285, 1258, 1224, 1183, 1148, 1112, 1081, 1043, 1007, 860, 823, 747, 716.

HRMS (ESI/QTOF) m/z: $[M + Na]^+$ Calcd for $C_{14}H_{19}INaO_3^+$ 385.0271; Found 385.0270.

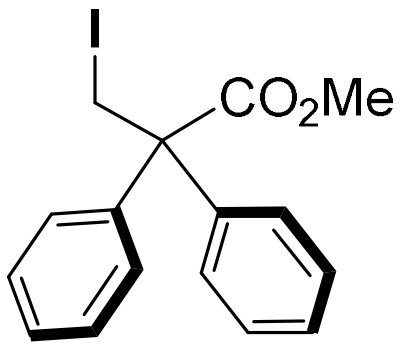

5v. Colorless oil.

Analytical data for **5v**:

^{1}H NMR (400 MHz, $CDCl_3$) δ 7.34 – 7.31 (m, 10H), 4.11 (s, 2H), 3.75 (s, 3H).

^{13}C NMR (101 MHz, $CDCl_3$) δ 172.1, 141.1, 128.9, 128.0, 127.5, 60.4, 52.8, 15.1.

IR (neat) ν_{max} (cm^{-1}) = 2979, 2931, 2868, 2856, 1637, 1578, 1532, 1490, 1451, 1282, 1271, 1143, 913, 888, 743, 714, 692.

HRMS (Sicrit plasma/LTQ-Orbitrap) m/z: $[M + H]^+$ Calcd for $C_{16}H_{16}IO_2^+$ 367.0190; Found 367.0189.

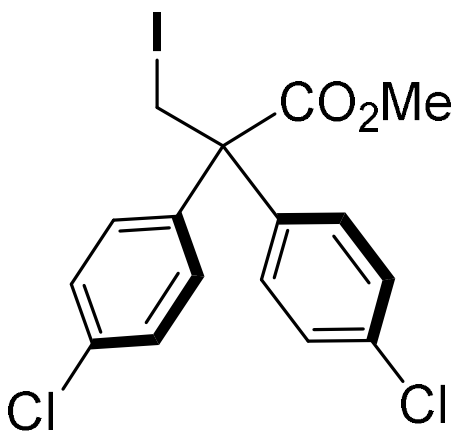

5w. Colorless oil.

Analytical data for **5w**:

^{1}H NMR (800 MHz, $CDCl_3$) δ 7.32 – 7.29 (m, 4H), 7.25 – 7.21 (m, 4H), 4.02 (s, 2H), 3.74 (s, 3H).

^{13}C NMR (201 MHz, $CDCl_3$) δ 171.4, 139.1, 133.8, 130.2, 128.3, 59.7, 53.1, 14.1.

IR (neat) ν_{max} (cm^{-1}) = 2951, 1730, 1612, 1588, 1488, 1434, 1378, 1264, 1243, 1211, 1157, 1120, 1096, 982, 913, 868, 815, 785, 772, 726, 693, 653.

HRMS (ESI/QTOF) m/z: $[M + Na]^+$ Calcd for $C_{16}H_{13}Cl_2INaO_2^+$ 456.9230; Found 456.9234.

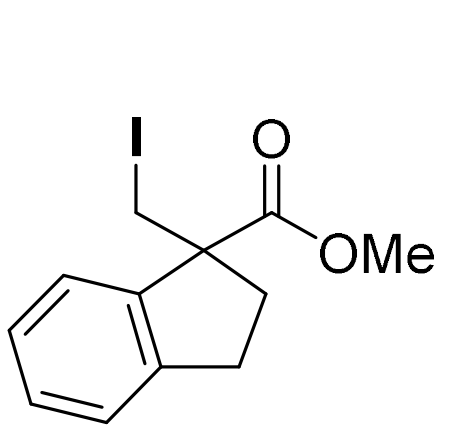

5x. Colorless oil.

Analytical data for **5x**:

^{1}H NMR (400 MHz, $CDCl_3$) δ 7.28 (dd, *J* = 7.4, 1.5 Hz, 1H), 7.22 – 7.09 (m, 3H), 3.89 (d, *J* = 9.6 Hz, 1H), 3.66 (s, 3H), 3.22 (d, *J* = 9.6 Hz, 1H), 2.99 (ddd, *J* = 16.6, 9.2, 6.8 Hz, 1H), 2.85 (tdd, *J* = 13.8, 8.6, 5.0 Hz, 2H), 2.05 (ddd, *J* = 12.9, 8.9, 6.7 Hz, 1H).

^{13}C NMR (101 MHz, $CDCl_3$) δ 172.9, 144.2, 142.0, 128.6, 126.8, 125.3, 123.9, 60.1, 52.6, 36.3, 30.2, 13.2.

IR (neat) ν_{max} (cm^{-1}) = 2988, 2932, 2869, 2853, 1627, 1605, 1540, 1503, 1462, 1451, 1441, 1330, 1314, 1252, 1177, 1143, 1031, 885, 848, 770, 618, 604.

HRMS (ESI/QTOF) m/z: $[M + Na]^+$ Calcd for $C_{12}H_{13}INaO_2^+$ 338.9852; Found 338.9847.

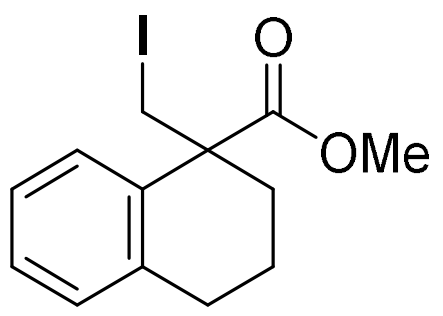

5y. Colorless oil.

Analytical data for **5y**:

^{1}H NMR (400 MHz, $CDCl_3$) δ 7.36 (dd, *J* = 7.8, 1.5 Hz, 1H), 7.23 – 7.08 (m, 3H), 3.96 (d, *J* = 10.1 Hz, 1H), 3.73 (s, 3H), 3.42 (d, *J* = 10.1 Hz, 1H), 2.86 – 2.71 (m, 2H), 2.46 (ddd, *J* = 12.8, 9.3, 2.9 Hz, 1H), 2.08 (ddd, *J* = 13.6, 8.5, 2.9 Hz, 1H), 2.04 – 1.89 (m, 1H), 1.81 – 1.71 (m, 1H).

^{13}C NMR (101 MHz, $CDCl_3$) δ 173.5, 137.3, 134.8, 129.8, 127.6, 127.3, 126.4, 52.5, 49.6, 32.8, 29.8, 19.2, 16.2.

IR (neat) ν_{max} (cm^{-1}) = 2979, 2929, 2869, 2853, 1636, 1586, 1526, 1487, 1450, 1333, 1281, 1211, 1141, 886, 809, 742, 690, 664.

HRMS (ESI/QTOF) m/z: $[M + Na]^+$ Calcd for $C_{13}H_{15}INaO_2^+$ 353.0009; Found 353.0013.

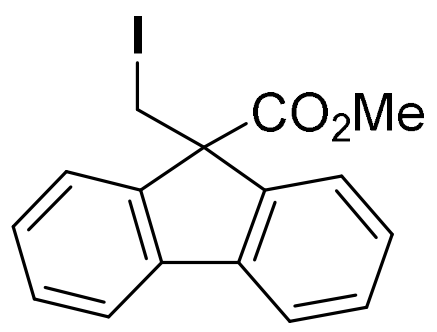

5z. White solid.

Analytical data for **5z**:

m.p. = 114 ~ 116 °C.

^{1}H NMR (400 MHz, $CDCl_3$) δ 7.75 (dt, *J* = 7.5, 0.9 Hz, 2H), 7.68 (dt, *J* = 7.6, 1.0 Hz, 2H), 7.46 (td, *J* = 7.5, 1.1 Hz, 2H), 7.37 (td, *J* = 7.5, 1.0 Hz, 2H), 3.88 (s, 2H), 3.67 (s, 3H).

^{13}C NMR (101 MHz, $CDCl_3$) δ 171.3, 144.3, 140.9, 129.0, 127.6, 124.6, 120.1, 60.8, 53.1, 11.2.

IR (neat) ν_{max} (cm^{-1}) = 2988, 2869, 1717, 1447, 1431, 1250, 1230, 1213, 1188, 1143, 1065, 1003, 920, 764, 747, 732, 688, 619, 604.

HRMS (ESI/QTOF) m/z: $[M + Na]^+$ Calcd for $C_{16}H_{13}INaO_2^+$ 386.9852; Found 386.9853.

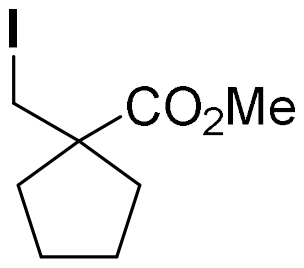

5aa. Colorless oil.

Analytical data for **5aa**:

^{1}H NMR (400 MHz, $CDCl_3$) δ 3.73 (s, 3H), 3.42 (s, 2H), 2.27 – 2.14 (m, 2H), 1.80 – 1.60 (m, 6H).

^{13}C NMR (101 MHz, $CDCl_3$) δ 175.6, 55.2, 52.3, 37.1, 25.5, 14.7.

IR (neat) ν_{max} (cm^{-1}) = 2926, 1639, 1586, 1528, 1483, 1450, 1332, 1283, 1214, 890, 744, 689.

HRMS (ESI/QTOF) m/z: $[M + Na]^+$ Calcd for $C_8H_{13}INaO_2^+$ 290.9852; Found 290.9859.

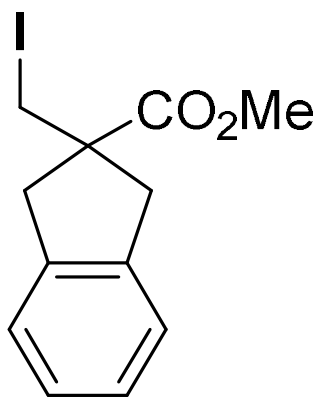

5ab. Colorless oil.

Analytical data for **5ab**:

^{1}H NMR (400 MHz, $CDCl_3$) δ 7.21 – 7.15 (m, 4H), 3.77 (d, J = 1.0 Hz, 3H), 3.55 (d, J = 16.5 Hz, 2H), 3.51 (s, 2H), 3.06 (d, J = 16.5 Hz, 2H).

^{13}C NMR (101 MHz, $CDCl_3$) δ 174.5, 140.1, 126.9, 124.7, 55.2, 52.6, 42.9, 13.8.

IR (neat) ν_{max} (cm^{-1}) = 2988, 2980, 2933, 2869, 1640, 1595, 1540, 1484, 1450, 1393, 1380, 1361, 1348, 1294, 1279, 1142, 1092, 1015, 913, 744.

HRMS (LTQ-Orbitrap) m/z: $[M + H]^+$ Calcd for $C_{12}H_{14}IO_2^+$ 317.0033; Found 317.0043.

General Procedure F:

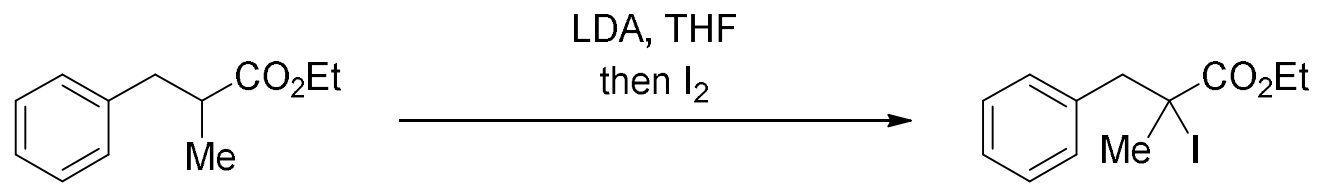

S15 **14**

To a solution of **S15** (5 mmol) in THF (1 M) at -78 ºC was added LDA (1.0 M solution in THF, 1.3 equiv) dropwise. The mixture was stirred at -78 ºC for 1 h and iodine (1.5 equiv) was added slowly. The reaction mixture was allowed to warm to room temperature and stirred overnight. Then, the mixture was quenched with $Na_2S_2O_3$ solution (2 M) and extracted with ethyl acetate (twice). The extracts were combined, dried over Na_2SO_4, filtered and concentrated under reduced pressure. The residue was purified by silica gel flash column chromatography (eluent: hexanes / ethyl acetate = 50:1 → 20:1) to give the desired product **14**.

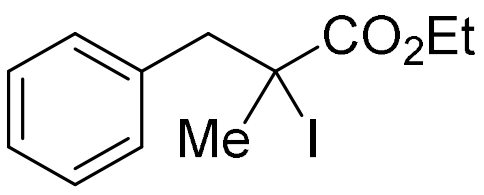

14. Colorless oil (1.13 g, 71% yield).

Analytical data for **14**:

^{1}H NMR (500 MHz, $CDCl_3$) δ 7.30 – 7.26 (m, 3H), 7.24 – 7.19 (m, 2H), 4.25 (q, *J* = 7.1 Hz, 2H), 3.75 (d, *J* = 13.7 Hz, 1H), 3.45 (d, *J* = 13.7 Hz, 1H), 2.00 (s, 3H), 1.32 (t, *J* = 7.1 Hz, 3H).

^{13}C NMR (126 MHz, $CDCl_3$) δ 172.6, 136.9, 130.4, 128.3, 127.2, 62.0, 50.2, 40.1, 29.8, 13.7.

IR (neat) ν_{max} (cm^{-1}) = 2979, 1732, 1522, 1488, 1421, 1402, 1328, 1333, 1271, 1266, 1154, 1071, 1037, 943, 862, 826, 755, 701, 694.

HRMS (ESI/QTOF) m/z: $[M + Na]^+$ Calcd for $C_{12}H_{15}INaO_2^+$ 341.0009; Found 341.0020.

General procedure for hypervalent iodine based dyotropic rearrangement reactions: migrative fluorination

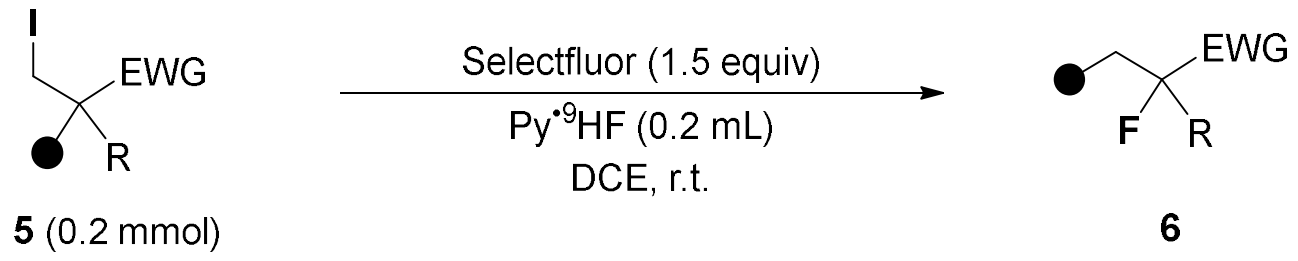

At room temperature, to a 10-mL test tube charged with a magnetic stirring bar, were added substrate **5** (0.2 mmol, 1.0 equiv), Selectfluor (106 mg, 0.3 mmol, 1.5 equiv) and Py·9HF (0.2 mL) in anhydrous DCE (2.0 mL). The reaction mixture was allowed to stir at room temperature. After the reaction was complete (monitored by TLC), the mixture was quenched with $NaHCO_3$ solution (2 M) and $Na_2S_2O_3$ solution (2 M). Then, the mixture was extracted twice with ethyl acetate. The combined organic extracts were dried over Na_2SO_4 and concentrated under reduced pressure. The residue was purified by silica gel flash column chromatography to give the desired product **6**.

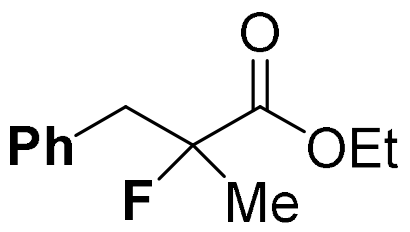

6a. Colorless oil (33.6 mg, 80% yield).

Analytical data for **6a**:

^{1}H NMR (400 MHz, $CDCl_3$) δ 7.25 – 7.17 (m, 3H), 7.16 – 7.11 (m, 2H), 4.12 – 4.04 (m, 2H), 3.14 (dd, J = 26.3, 14.3 Hz, 1H), 3.02 (dd, J = 21.4, 14.3 Hz, 1H),1.49 (d, J = 21.1 Hz, 3H), 1.12 (t, J = 7.1 Hz, 3H).

^{13}C NMR (101 MHz, $CDCl_3$) δ 171.6 (d, J = 24.8 Hz), 134.6 (br), 130.22 (d, J = 1.3 Hz), 128.2, 127.1, 94.9 (d, J = 187.8 Hz), 61.5, 44.1 (d, J = 21.8 Hz), 23.3 (d, J = 24.2 Hz), 14.0.

^{19}F{^{1}H} NMR (377 MHz, $CDCl_3$) δ -154.99.

^{19}F NMR (377 MHz, $CDCl_3$) δ -154.98 (dp, J = 26.2, 21.0 Hz).

IR (neat) ν_{max} (cm^{-1}) = 2988, 2870, 1757, 1735, 1700, 1684, 1653, 1559, 1540, 1507, 1497, 1472, 1456, 1375, 1334, 1279, 1188, 1121, 1099, 1020, 946, 861, 833, 768, 745, 700, 677.

HRMS (Sicrit plasma/LTQ-Orbitrap) m/z: $[M + H]^+$ Calcd for $C_{12}H_{16}FO_2^+$ 211.1129; Found 211.1127.

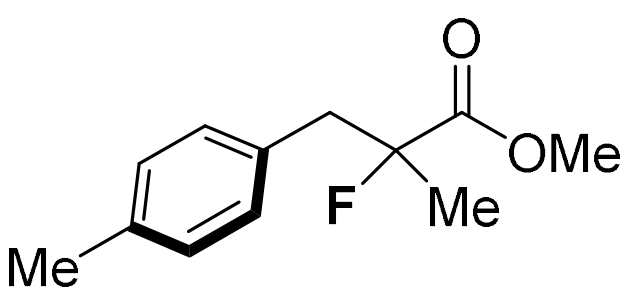

6b. Colorless oil (25.6 mg, 61% yield).

Analytical data for **6b**:

^{1}H NMR (500 MHz, $CDCl_3$) δ 7.12 – 7.07 (m, 4H), 3.71 (s, 3H), 3.18 (dd, J = 25.9, 14.3 Hz, 1H), 3.05 (dd, J = 21.4, 14.3 Hz, 1H), 2.32 (s, 3H), 1.56 (d, J = 21.2 Hz, 3H).

^{13}C NMR (126 MHz, $CDCl_3$) δ 172.2 (d, J = 25.0 Hz), 136.7 (br), 131.4, 130.0, 129.0, 95.1 (d, J = 187.5 Hz), 52.4, 43.8 (d, J = 21.9 Hz), 23.1 (d, J = 24.2 Hz), 21.1.

^{19}F{^{1}H} NMR (377 MHz, $CDCl_3$) δ -155.17.

^{19}F NMR (377 MHz, $CDCl_3$) δ -155.17 (dp, J = 25.5, 21.0 Hz).

IR (neat) ν_{max} (cm^{-1}) = 2931, 2860, 1732, 1511, 1433, 1376, 1290, 1263, 1155, 1152, 1116, 1088, 1072, 1043, 1019, 984, 922, 874, 815, 772, 731, 612.

HRMS (ESI/QTOF) m/z: $[M + Na]^+$ Calcd for $C_{12}H_{15}FNaO_2^+$ 233.0948; Found 233.0952.

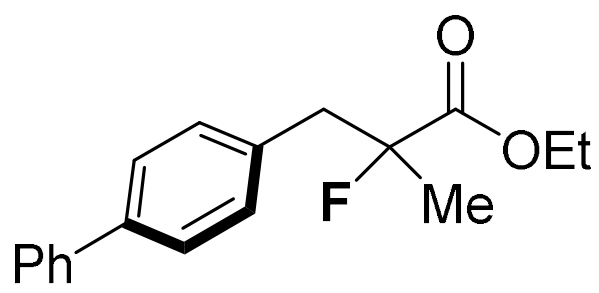

6c. Colorless oil (45.2 mg, 79% yield).

Analytical data for **6c**:

^{1}H NMR (400 MHz, $CDCl_3$) δ 7.61 – 7.56 (m, 2H), 7.55 – 7.50 (m, 2H), 7.46 – 7.40 (m, 2H), 7.37 – 7.32 (m, 1H), 7.31 – 7.27 (m, 2H), 4.19 (qd, J = 7.1, 3.1 Hz, 2H), 3.26 (dd, J = 26.5, 14.3 Hz, 1H), 3.14 (dd, J = 21.2, 14.3 Hz, 1H), 1.61 (d, J = 21.2 Hz, 3H), 1.22 (t, J = 7.1 Hz, 3H).

^{13}C NMR (101 MHz, $CDCl_3$) δ 171.6 (d, J = 25.0 Hz), 140.8, 140.0, 133.7, 130.7 (br), 128.7, 127.2, 127.02, 126.97, 94.9 (d, J = 187.9 Hz), 61.6, 43.8 (d, J = 21.8 Hz), 23.3 (d, J = 24.1 Hz), 14.1.

^{19}F{^{1}H} NMR (377 MHz, $CDCl_3$) δ -155.05.

^{19}F NMR (377 MHz, $CDCl_3$) δ -155.05 (dp, J = 26.4, 21.0 Hz).

IR (neat) ν_{max} (cm^{-1}) = 2988, 2979, 2869, 1727, 1489, 1459, 1396, 1377, 1364, 1285, 1258, 1224, 1183, 1148, 1112, 1081, 1043, 1007, 860, 823, 747, 716.

HRMS (ESI/QTOF) m/z: $[M + Na]^+$ Calcd for $C_{18}H_{19}FNaO_2^+$ 309.1261; Found 309.1257.

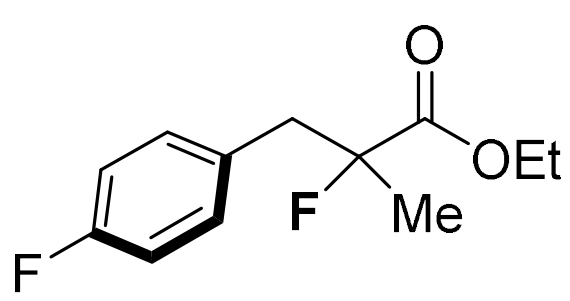

6d. Colorless oil (37.4 mg, 82% yield).

Analytical data for **6d**:

^{1}H NMR (400 MHz, $CDCl_3$) δ 7.22 – 7.15 (m, 2H), 7.01 – 6.92 (m, 2H), 4.19 – 4.12 (m, 2H), 3.18 (dd, *J* = 27.1, 14.5 Hz, 1H), 3.06 (dd, *J* = 20.6, 14.5 Hz, 1H), 1.56 (d, *J* = 21.1 Hz, 3H), 1.20 (t, *J* = 7.1 Hz, 3H).

^{13}C NMR (101 MHz, $CDCl_3$) δ 171.4 (d, *J* = 25.0 Hz), 162.1 (d, *J* = 245.3 Hz), 131.7 (dd, *J* = 8.0, 1.4 Hz), 130.3 (d, *J* = 3.3 Hz), 115.1 (d, *J* = 21.2 Hz), 94.8 (d, *J* = 188.0 Hz), 61.6, 43.3 (d, *J* = 21.8 Hz), 23.3 (d, *J* = 24.1 Hz), 14.0.

^{19}F{^{1}H} NMR (377 MHz, $CDCl_3$) δ -115.77, -155.58.

^{19}F NMR (377 MHz, $CDCl_3$) δ -115.72 – 115.80 (m), -155.58 (dp, *J* = 27.1, 20.9 Hz).

IR (neat) ν_{max} (cm^{-1}) = 2986, 1757, 1735, 1607, 1510, 1448, 1418, 1376, 1336, 1281, 1221, 1190, 1159, 1121, 1106, 1086, 1017, 942, 885, 843, 818, 780, 754, 669, 609.

HRMS (nanochip-ESI/LTQ-Orbitrap) m/z: $[M + Na]^+$ Calcd for $C_{12}H_{14}F_2NaO_2^+$ 251.0854; Found 251.0847.

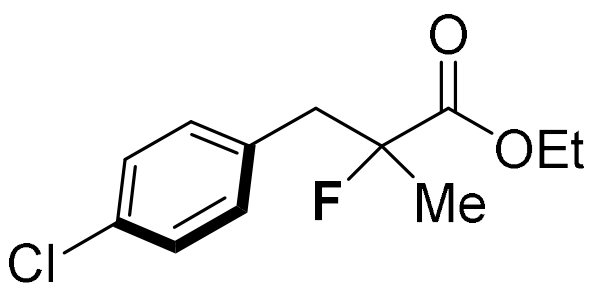

6e. Colorless oil (38.5 mg, 79% yield).

Analytical data for **6e**:

^{1}H NMR (400 MHz, $CDCl_3$) δ 7.27 – 7.24 (m, 2H), 7.17 – 7.12 (m, 2H), 4.16 (qd, *J* = 7.1, 1.4 Hz, 2H), 3.18 (dd, *J* = 26.9, 14.4 Hz, 1H), 3.05 (dd, *J* = 20.5, 14.4 Hz, 1H), 1.56 (d, *J* = 21.2 Hz, 3H), 1.21 (t, *J* = 7.1 Hz, 3H).

^{13}C NMR (101 MHz, $CDCl_3$) δ 171.3 (d, *J* = 24.8 Hz), 133.11, 133.08, 131.5 (d, *J* = 1.4 Hz), 128.4, 94.7 (d, *J* = 188.1 Hz), 61.6, 43.4 (d, *J* = 21.7 Hz), 23.3 (d, *J* = 24.2 Hz), 14.0.

^{19}F{^{1}H} NMR (377 MHz, $CDCl_3$) δ -155.43.

^{19}F NMR (377 MHz, $CDCl_3$) δ -155.36 (dp, *J* = 27.2, 20.7 Hz).

IR (neat) ν_{max} (cm^{-1}) = 2988, 1757, 1735, 1684, 1653, 1559, 1540, 1507, 1492, 1457, 1375, 1275, 1192, 1112, 1092, 1017, 941, 847, 802, 735, 719, 669.

HRMS (Sicrit plasma/LTQ-Orbitrap) m/z: $[M + H]^+$ Calcd for $C_{12}H_{15}ClFO_2^+$ 245.0739; Found 245.0739.

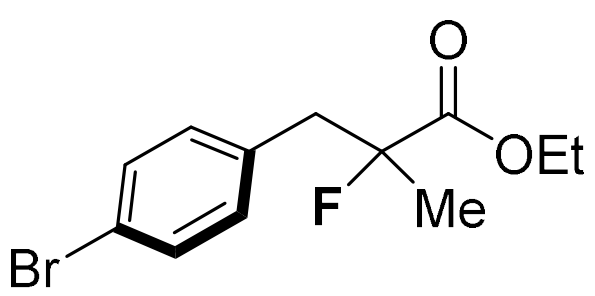

6f. Colorless oil (41.5 mg, 72% yield).

Analytical data for **6f**:

^{1}H NMR (400 MHz, $CDCl_3$) δ 7.43 – 7.40 (m, 2H), 7.13 – 7.06 (m, 2H), 4.16 (qd, *J* = 7.2, 1.3 Hz, 2H), 3.17 (dd, *J* = 26.9, 14.4 Hz, 1H), 3.04 (dd, *J* = 20.6, 14.4 Hz, 1H), 1.56 (d, *J* = 21.2 Hz, 3H), 1.21 (t, *J* = 7.1 Hz, 3H).

^{13}C NMR (101 MHz, $CDCl_3$) δ 171.3 (d, *J* = 24.9 Hz), 133.6, 131.9 (d, *J* = 1.3 Hz), 131.4, 121.3, 94.6 (d, *J* = 188.1 Hz), 61.7, 43.5 (d, *J* = 21.8 Hz), 23.3 (d, *J* = 24.3 Hz), 14.1.

^{19}F{^{1}H} NMR (377 MHz, $CDCl_3$) δ -155.44.

^{19}F NMR (377 MHz, $CDCl_3$) δ -155.44 (dp, *J* = 26.9, 20.9 Hz).

IR (neat) ν_{max} (cm^{-1}) = 2927, 1735, 1653, 1559, 1540, 1507, 1489, 1447, 1406, 1375, 1275, 1191, 1111, 1088, 1072, 1013, 941, 846, 799, 730, 713, 669, 644.

HRMS (ESI/QTOF) m/z: $[M + Na]^+$ Calcd for $C_{12}H_{14}BrFNaO_2^+$ 311.0053; Found 311.0062.

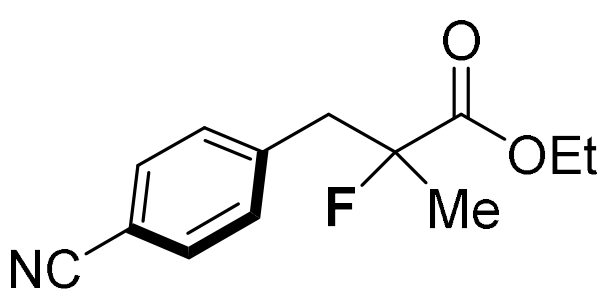

6g. Colorless oil (32.9 mg, 70% yield).

Analytical data for **6g**:

^{1}H NMR (400 MHz, $CDCl_3$) δ 7.58 (d, *J* = 8.3 Hz, 2H), 7.34 (d, *J* = 7.6 Hz, 2H), 4.15 (q, *J* = 7.1 Hz, 2H), 3.27 (dd, *J* = 27.4, 14.3 Hz, 1H), 3.14 (dd, *J* = 19.6, 14.3 Hz, 1H), 1.59 (d, *J* = 21.1 Hz, 3H), 1.20 (t, *J* = 7.1 Hz, 3H).

^{13}C NMR (101 MHz, $CDCl_3$) δ 170.9 (d, *J* = 24.8 Hz), 140.2, 132.0, 131.0 (d, *J* = 1.5 Hz), 118.7, 111.2, 94.4 (d, *J* = 189.3 Hz), 61.8, 44.0 (d, *J* = 21.7 Hz), 23.5 (d, *J* = 24.1 Hz), 14.0.

^{19}F{^{1}H} NMR (377 MHz, $CDCl_3$) δ -155.39.

^{19}F NMR (377 MHz, $CDCl_3$) δ -155.38 (dp, *J* = 27.5, 20.8 Hz).

IR (neat) ν_{max} (cm^{-1}) = 2988, 2228, 1756, 1735, 1610, 1507, 1457, 1417, 1377, 1335, 1282, 1192, 1120, 1092, 1020, 945, 856, 837, 816, 771, 669, 649, 612.

HRMS (ESI/QTOF) m/z: $[M + Na]^+$ Calcd for $C_{13}H_{14}FNNaO_2^+$ 258.0901; Found 258.0906.

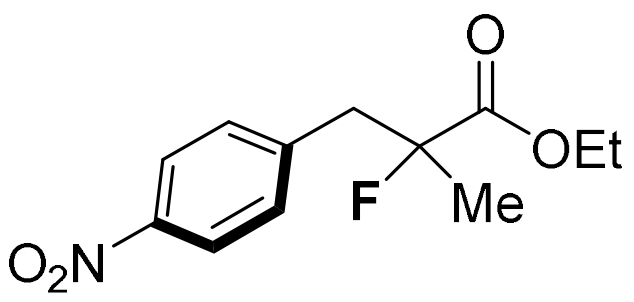

6h. Colorless oil (34.7 mg, 68% yield).

Analytical data for **6h**:

¹H NMR (400 MHz, $CDCl_3$) δ 8.20 – 8.11 (m, 2H), 7.47 – 7.35 (m, 2H), 4.17 (q, *J* = 7.2 Hz, 2H), 3.33 (dd, *J* = 27.4, 14.3 Hz, 1H), 3.19 (dd, *J* = 19.3, 14.3 Hz, 1H), 1.61 (d, *J* = 21.1 Hz, 3H), 1.21 (t, *J* = 7.1 Hz, 3H).

¹³C NMR (101 MHz, $CDCl_3$) δ 170.9 (d, *J* = 24.9 Hz), 147.3, 142.2, 131.1 (d, *J* = 1.6 Hz), 123.4, 94.4 (d, *J* = 189.5 Hz), 61.9, 43.7 (d, *J* = 21.5 Hz), 23.6 (d, *J* = 24.1 Hz), 14.1.

¹⁹F{¹H} NMR (377 MHz, $CDCl_3$) δ -155.37.

¹⁹F NMR (377 MHz, $CDCl_3$) δ -155.37 (dp, *J* = 27.3, 20.9 Hz).

IR (neat) ν_{max} (cm^{-1}) = 2988, 2869, 1755, 1735, 1606, 1518, 1448, 1377, 1345, 1282, 1195, 1111, 1091, 1017, 945, 888, 857, 831, 808, 764, 746, 712, 700, 654, 608.

HRMS (Sicrit plasma/LTQ-Orbitrap) m/z: $[M + H]^+$ Calcd for $C_{12}H_{15}FNO_4^+$ 256.0980; Found 256.0980.

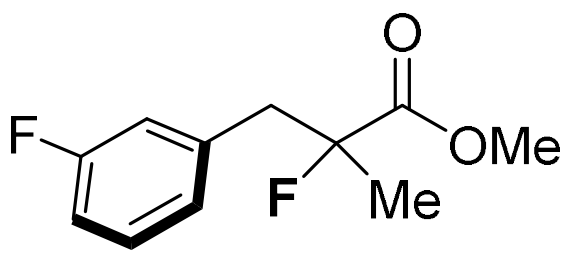

6i. White solid (29.1 mg, 68% yield).

Analytical data for **6i**:

¹H NMR (500 MHz, $CDCl_3$) δ 7.27 (td, *J* = 8.1, 6.2 Hz, 1H), 7.02 – 6.91 (m, 3H), 3.73 (s, 3H), 3.23 (dd, *J* = 26.4, 14.3 Hz, 1H), 3.09 (dd, *J* = 20.7, 14.3 Hz, 1H), 1.59 (d, *J* = 21.2 Hz, 3H).

¹³C NMR (126 MHz, $CDCl_3$) δ 171.8 (d, *J* = 25.1 Hz), 162.6 (d, *J* = 245.6 Hz), 136.9 (d, *J* = 7.6 Hz), 129.7 (d, *J* = 8.2 Hz), 125.9 (d, *J* = 2.9 Hz), 117.1 (d, *J* = 21.4 Hz), 114.2 (d, *J* = 21.0 Hz), 94.8 (d, *J* = 188.5 Hz), 52.5, 43.8 (dd, *J* = 21.8, 1.8 Hz), 23.3 (d, *J* = 24.0 Hz).

¹⁹F{¹H} NMR (377 MHz, $CDCl_3$) δ -113.46, -155.32.

¹⁹F NMR (377 MHz, $CDCl_3$) δ -113.46 (app td, *J* = 9.2, 5.9 Hz), -155.32 (dp, *J* = 26.3, 21.1 Hz).

IR (neat) ν_{max} (cm^{-1}) = 2951, 1741, 1617, 1589, 1488, 1449, 1376, 1253, 1200, 1145, 1120, 1101, 985, 871, 790, 772, 745, 693, 616.

HRMS (ESI/QTOF) m/z: $[M + Na]^+$ Calcd for $C_{11}H_{12}F_2NaO_2^+$ 237.0698; Found 237.0699.

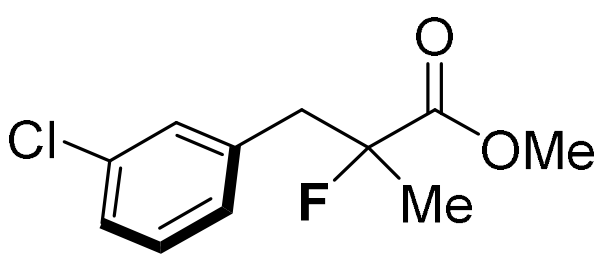

6j. Colorless oil (34.1 mg, 74% yield).

Analytical data for **6j**:

¹H NMR (400 MHz, $CDCl_3$) δ 7.29 – 7.21 (m, 3H), 7.13 – 7.09 (m, 1H), 3.74 (s, 3H), 3.21 (dd, *J* = 26.3, 14.3 Hz, 1H), 3.07 (dd, *J* = 20.8, 14.3 Hz, 1H), 1.59 (d, *J* = 21.2 Hz, 3H).

^{13}C NMR (101 MHz, $CDCl_3$) δ 171.7 (d, J = 25.0 Hz), 136.5, 134.1, 130.3 (d, J = 1.5 Hz), 129.5, 128.4 (d, J = 1.3 Hz), 127.4, 94.8 (d, J = 188.5 Hz), 52.5, 43.7 (d, J = 21.8 Hz), 23.3 (d, J = 24.0 Hz).

^{19}F{^{1}H} NMR (377 MHz, $CDCl_3$) δ -155.34.

^{19}F NMR (377 MHz, $CDCl_3$) δ -155.34 (dp, J = 26.2, 21.1 Hz).

IR (neat) ν_{max} (cm^{-1}) = 2954, 1762, 1740, 1598, 1574, 1559, 1540, 1507, 1477, 1434, 1376, 1338, 1277, 1201, 1122, 1088, 985, 944, 862, 816, 786, 768, 725, 703, 685, 617.

HRMS (ESI/QTOF) m/z: $[M + Na]^+$ Calcd for $C_{11}H_{12}ClFNaO_2^+$ 253.0402; Found 253.0406.

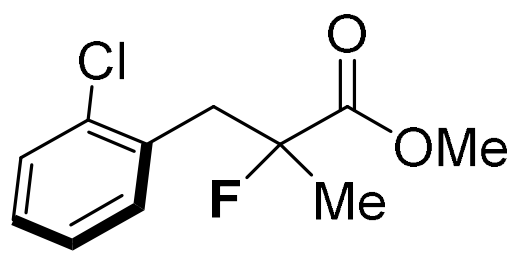

6k. Colorless oil (29.9 mg, 65% yield).

Analytical data for **6k**:

^{1}H NMR (400 MHz, $CDCl_3$) δ 7.41 – 7.35 (m, 1H), 7.34 – 7.28 (m, 1H), 7.24 – 7.18 (m, 2H), 3.77 (s, 3H), 3.43 (dd, J = 23.2, 14.6 Hz, 1H), 3.34 (dd, J = 22.5, 14.6 Hz, 1H), 1.58 (d, J = 21.6 Hz, 3H).

^{13}C NMR (101 MHz, $CDCl_3$) δ 171.9 (d, J = 25.2 Hz), 135.0, 132.6, 132.3 (d, J = 2.4 Hz), 129.6, 128.6, 126.7, 94.6 (d, J = 188.1 Hz), 52.6, 40.2 (d, J = 21.8 Hz), 22.8 (d, J = 24.0 Hz).

^{19}F{^{1}H} NMR (377 MHz, $CDCl_3$) δ -153.48.

^{19}F NMR (377 MHz, $CDCl_3$) δ -153.48 (h, J = 21.9 Hz).

IR (neat) ν_{max} (cm^{-1}) = 2954, 1761, 1743, 1684, 1653, 1559, 1540, 1507, 1475, 1436, 1376, 1339, 1286, 1204, 1122, 1098, 1053, 984, 942, 754, 682, 649, 617.

HRMS (ESI/QTOF) m/z: $[M + Na]^+$ Calcd for $C_{11}H_{12}ClFNaO_2^+$ 253.0402; Found 253.0409.

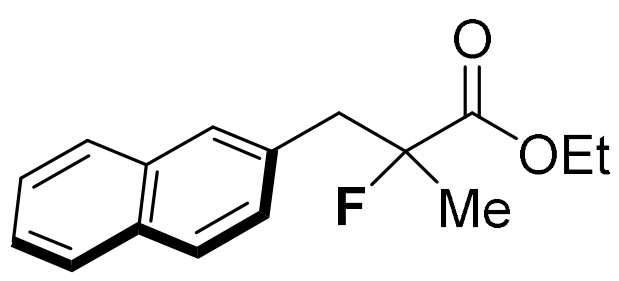

6l. Colorless oil (36.4 mg, 70% yield).

Analytical data for **6l**:

^{1}H NMR (400 MHz, $CDCl_3$) δ 7.83 – 7.77 (m, 3H), 7.69 (s, 1H), 7.50 – 7.43 (m, 2H), 7.38 (dt, J = 8.3, 1.6 Hz, 1H), 4.16 (q, J = 7.1 Hz, 2H), 3.39 (dd, J = 26.1, 14.3 Hz, 1H), 3.27 (dd, J = 21.5, 14.3 Hz, 1H), 1.62 (d, J = 21.2 Hz, 3H), 1.18 (t, J = 7.1 Hz, 3H).

^{13}C NMR (101 MHz, $CDCl_3$) δ 171.6 (d, J = 24.8 Hz), 133.3, 132.5, 132.2, 129.1, 128.4 (d, J = 1.5 Hz), 127.8, 127.7, 127.6, 126.0, 125.7, 95.0 (d, J = 187.9 Hz), 61.6, 44.2 (d, J = 21.8 Hz), 23.3 (d, J = 24.2 Hz), 14.0.

^{19}F{^{1}H} NMR (377 MHz, $CDCl_3$) δ -154.62.

^{19}F NMR (377 MHz, $CDCl_3$) δ -154.62 (dp, *J* = 26.1, 21.4 Hz).

IR (neat) ν_{max} (cm^{-1}) = 2979, 2869, 1727, 1506, 1457, 1378, 1271, 1222, 1146, 1129, 1092, 1065, 1044, 1017, 949, 894, 857, 814, 774, 747, 657, 648, 637, 628, 603.

HRMS (ESI/QTOF) m/z: $[M + Na]^+$ Calcd for $C_{16}H_{17}FNaO_2^+$ 283.1105; Found 283.1108.

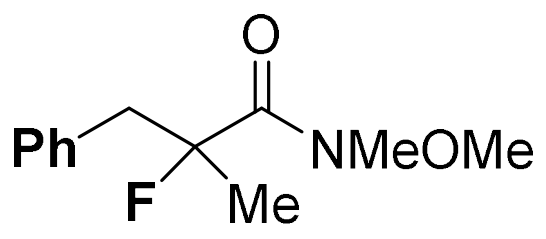

6m. Colorless oil (30.6 mg, 68% yield).

Analytical data for **6m**:

^{1}H NMR (400 MHz, $CDCl_3$) δ 7.32 – 7.21 (m, 5H), 3.66 (s, 3H), 3.37 (dd, *J* = 22.9, 14.2 Hz, 1H), 3.13 (s, 3H), 3.10 (dd, *J* = 24.2, 14.2 Hz, 1H), 1.56 (d, *J* = 22.1 Hz, 3H).

^{19}F{^{1}H} NMR (377 MHz, $CDCl_3$) δ -153.20.

^{19}F NMR (377 MHz, $CDCl_3$) δ -153.20 (br).

This is a known compound. The spectroscopic data matches those reported in the literature.[3]

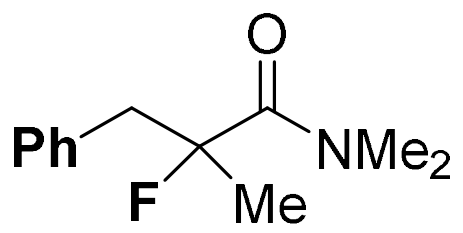

6n. Colorless oil (31.4 mg, 75% yield).

Analytical data for **6n**:

^{1}H NMR (500 MHz, $CDCl_3$) δ 7.29 (ddt, *J* = 11.4, 7.1, 3.8 Hz, 3H), 7.25 – 7.20 (m, 2H), 3.35 (dd, *J* = 27.4, 14.2 Hz, 1H), 3.07 (dd, *J* = 21.2, 14.2 Hz, 1H), 2.90 (s, 3H), 2.85 (d, *J* = 4.5 Hz, 3H), 1.62 (d, *J* = 21.8 Hz, 3H).

^{13}C NMR (126 MHz, $CDCl_3$) δ 171.1 (d, *J* = 20.5 Hz), 135.5, 130.6, 128.1, 126.9, 99.2 (d, *J* = 190.7 Hz), 44.9 (d, *J* = 21.5 Hz), 37.8, 37.5 (d, *J* = 18.9 Hz), 24.6 (d, *J* = 24.3 Hz).

^{19}F{^{1}H} NMR (377 MHz, $CDCl_3$) δ -150.07.

^{19}F NMR (377 MHz, $CDCl_3$) δ -149.91 – -150 23 (m).

IR (neat) ν_{max} (cm^{-1}) = 2934, 2869, 1600, 1446, 1423, 1405, 1374, 1361, 1200, 1173, 1142, 1069, 1026, 992, 766, 732, 699, 601.

HRMS (ESI/QTOF) m/z: $[M + Na]^+$ Calcd for $C_{12}H_{16}FNNaO^+$ 232.1108; Found 232.1111.

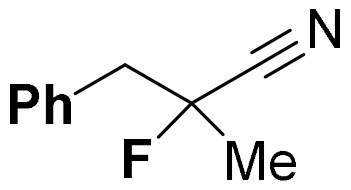

6o. Colorless oil (22.5 mg, 69% yield).

Analytical data for **6o**:

^{1}H NMR (400 MHz, $CDCl_3$) δ 7.40 – 7.33 (m, 3H), 7.32 – 7.27 (m, 2H), 3.24 (dd, *J* = 17.6, 14.2 Hz, 1H), 3.19 (dd, *J* = 20.6, 14.2 Hz, 1H), 1.73 (d, *J* = 21.1 Hz, 3H).

^{13}C NMR (101 MHz, $CDCl_3$) δ 132.4 (d, *J* = 4.0 Hz), 130.4, 128.7, 128.0, 118.0 (d, *J* = 34.3 Hz), 88.1 (d, *J* = 182.3 Hz), 45.7 (d, *J* = 23.3 Hz), 25.1 (d, *J* = 24.4 Hz).

^{19}F{^{1}H} NMR (377 MHz, $CDCl_3$) δ -143.74.

^{19}F NMR (377 MHz, $CDCl_3$) δ -143.74 (pd, *J* = 21.0, 17.7 Hz).

IR (neat) ν_{max} (cm^{-1}) = 2988, 2869, 1734, 1495, 1446, 1378, 1242, 1213, 1182, 1143, 1072, 1044, 1023, 760, 696, 642, 608, 603.

HRMS (Sicrit plasma/LTQ-Orbitrap) m/z: $[M + H]^+$ Calcd for $C_{10}H_{11}FN^+$ 164.0870; Found 164.0870.

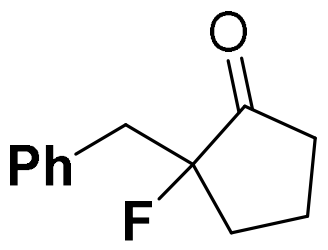

6p. Colorless oil (16.1 mg, 42% yield).

Analytical data for **6p**:

^{1}H NMR (400 MHz, $CDCl_3$) δ 7.35 – 7.24 (m, 3H), 7.23 – 7.18 (m, 2H), 3.16 (t, *J* = 14.1 Hz, 1H), 2.90 (dd, *J* = 28.0, 14.1 Hz, 1H), 2.50 – 2.40 (m, 1H), 2.25 – 2.13 (m, 1H), 2.08 – 1.93 (m, 3H), 1.78 – 1.67 (m, 1H).

^{13}C NMR (101 MHz, $CDCl_3$) δ 212.9 (d, *J* = 16.9 Hz), 134.6 (d, *J* = 4.9 Hz), 130.2, 128.5, 127.1, 97.3 (d, *J* = 184.7 Hz), 38.6 (d, *J* = 24.6 Hz), 35.6, 32.8 (d, *J* = 21.6 Hz), 17.3 (d, *J* = 3.7 Hz).

^{19}F{^{1}H} NMR (377 MHz, $CDCl_3$) δ -153.49.

^{19}F NMR (377 MHz, $CDCl_3$) δ -153.30 – -153.53 (m).

IR (neat) ν_{max} (cm^{-1}) = 2931, 2855, 1664, 1559, 1540, 1507, 1457, 1446, 1364, 1277, 1228, 1193, 1181, 1126, 1073, 944, 932, 912, 881, 862, 822, 753.

HRMS (Sicrit plasma/LTQ-Orbitrap) m/z: $[M + H]^+$ Calcd for $C_{12}H_{14}FO^+$ 193.1023; Found 193.1023.

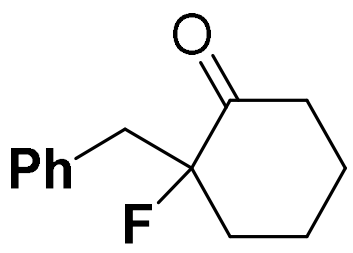

6q. Colorless oil (21.0 mg, 51% yield).

Analytical data for **6q**:

^{1}H NMR (600 MHz, $CDCl_3$) δ 7.32 – 7.28 (m, 2H), 7.27 – 7.25 (m, 1H), 7.24 – 7.21 (m, 2H), 3.23 (dd, *J* = 20.2, 14.8 Hz, 1H), 3.02 (dd, *J* = 30.5, 14.8 Hz, 1H), 2.78 – 2.72 (m, 1H), 2.46 (ddd, *J* = 12.9, 7.5, 4.7 Hz, 1H), 2.02 – 1.90 (m, 2H), 1.89 – 1.67 (m, 4H).

^{13}C NMR (151 MHz, $CDCl_3$) δ 207.6 (d, *J* = 21.0 Hz), 134.7, 130.5, 128.2, 126.9, 97.8 (d, *J* = 184.3 Hz), 39.8 (d, *J* = 22.1 Hz), 39.5, 37.0 (d, *J* = 22.6 Hz), 27.5, 21.5 (d, *J* = 6.1 Hz).

^{19}F{^{1}H} NMR (377 MHz, $CDCl_3$) δ -156.32.

^{19}F NMR (377 MHz, $CDCl_3$) δ -156.32 (app dddt, *J* = 32.8, 21.2, 15.1, 7.4 Hz).

IR (neat) ν_{max} (cm^{-1}) = 2979, 2869, 1669, 1559, 1540, 1521, 1507, 1491, 1448, 1362, 1315, 1287, 1235, 1200, 1145, 1079, 1027, 954, 875, 807, 780, 756, 697.

HRMS (ESI/QTOF) m/z: $[M + Na]^+$ Calcd for $C_{13}H_{15}FNaO^+$ 229.0999; Found 229.1001.

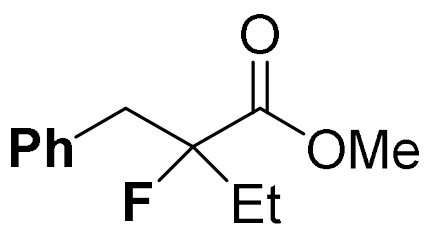

6r. Colorless oil (31.9 mg, 76% yield).

Analytical data for **6r**:

^{1}H NMR (400 MHz, $CDCl_3$) δ 7.24 – 7.16 (m, 3H), 7.16 – 7.09 (m, 2H), 3.60 (s, 3H), 3.11 (dd, *J* = 28.5, 14.3 Hz, 1H), 3.03 (dd, *J* = 19.9, 14.3 Hz, 1H), 2.00 – 1.72 (m, 2H), 0.89 (t, *J* = 7.5 Hz, 3H).

^{13}C NMR (101 MHz, $CDCl_3$) δ 171.5 (d, *J* = 25.5 Hz), 134.6, 130.1, 128.2, 127.1, 98.2 (d, *J* = 191.2 Hz), 52.2, 43.2 (d, *J* = 21.5 Hz), 30.2 (d, *J* = 22.8 Hz), 7.6 (d, *J* = 4.3 Hz).

^{19}F{^{1}H} NMR (377 MHz, $CDCl_3$) δ -166.95.

^{19}F NMR (377 MHz, $CDCl_3$) δ -166.83 – -167.07 (m).

IR (neat) ν_{max} (cm^{-1}) = 2975, 2953, 1762, 1735, 1718, 1700, 1507, 1496, 1456, 1438, 1386, 1354, 1259, 1245, 1199, 1155, 1142, 1124, 1101, 1030, 1007, 973, 878, 816, 744, 700, 670, 663, 649, 605.

HRMS (Sicrit plasma/LTQ-Orbitrap) m/z: $[M + H]^+$ Calcd for $C_{12}H_{16}FO_2^+$ 211.1129; Found 211.1128.

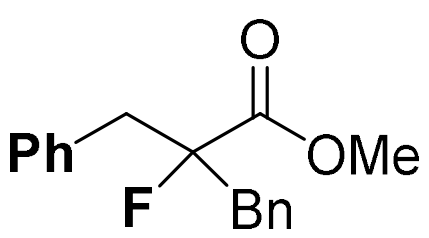

6s. Colorless oil (42.5 mg, 78% yield).

Analytical data for **6s**:

^{1}H NMR (400 MHz, $CDCl_3$) δ 7.33 – 7.25 (m, 6H), 7.24 – 7.21 (m, 4H), 3.54 (s, 3H), 3.27 (dd, *J* = 30.2, 14.3 Hz, 2H), 3.14 (dd, *J* = 18.1, 14.3 Hz, 2H).

^{13}C NMR (101 MHz, $CDCl_3$) δ 170.9 (d, *J* = 25.2 Hz), 134.4, 130.2 (d, *J* = 1.4 Hz), 128.3, 127.2, 97.7 (d, *J* = 194.7 Hz), 52.1, 43.2 (d, *J* = 21.4 Hz).

^{19}F{^{1}H} NMR (377 MHz, $CDCl_3$) δ -163.86.

^{19}F NMR (377 MHz, $CDCl_3$) δ -163.85 (tt, *J* = 30.0, 18.1 Hz).

IR (neat) ν_{max} (cm^{-1}) = 3031, 2989, 2869, 1763, 1735, 1521, 1496, 1455, 1437, 1349, 1279, 1236, 1199, 1129, 1085, 1039, 963, 818, 769, 748, 699, 670.

HRMS (Sicrit plasma/LTQ-Orbitrap) m/z: $[M + H]^+$ Calcd for $C_{17}H_{18}FO_2^+$ 273.1285; Found 273.1286.

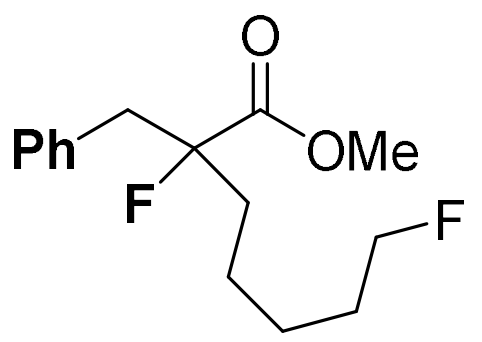

6t. Colorless oil (39.4 mg, 73% yield).

Analytical data for **6t**:

^{1}H NMR (400 MHz, $CDCl_3$) δ 7.33 – 7.22 (m, 3H), 7.21 – 7.15 (m, 2H), 4.48 (t, *J* = 6.0 Hz, 1H), 4.36 (t, *J* = 6.1 Hz, 1H), 3.68 (s, 3H), 3.25 – 3.14 (m, 1H), 3.13 – 3.04 (m, 1H), 2.07 – 1.90 (m, 1H), 1.88 – 1.76 (m, 1H), 1.75 – 1.68 (m, 1H), 1.67 – 1.61 (m, 1H), 1.61 – 1.48 (m, 1H), 1.46 – 1.37 (m, 2H), 1.35 – 1.22 (m, 1H).

^{13}C NMR (101 MHz, $CDCl_3$) δ 171.5 (d, *J* = 25.7 Hz), 134.5, 130.1, 128.3, 127.1, 97.7 (d, *J* = 191.2 Hz), 83.8 (d, *J* = 164.5 Hz), 52.3, 43.5 (d, *J* = 21.7 Hz), 36.9 (d, *J* = 22.3 Hz), 30.1 (d, *J* = 19.6 Hz), 25.1 (d, *J* = 5.3 Hz), 22.9 (d, *J* = 3.2 Hz).

^{19}F{^{1}H} NMR (377 MHz, $CDCl_3$) δ -165.07, -218.57.

^{19}F NMR (377 MHz, $CDCl_3$) δ -165.07 (dddd, *J* = 31.8, 28.2, 20.0, 12.5 Hz), -218.56 (tt, *J* = 47.9, 25.2 Hz).

IR (neat) ν_{max} (cm^{-1}) = 2954, 2869, 1735, 1724, 1718, 1559, 1507, 1496, 1456, 1437, 1275, 1199, 1141, 1029, 817, 746, 700, 669.

HRMS (Sicrit plasma/LTQ-Orbitrap) m/z: $[M + H]^+$ Calcd for $C_{15}H_{21}F_2O_2^+$ 271.1504; Found 271.1505.

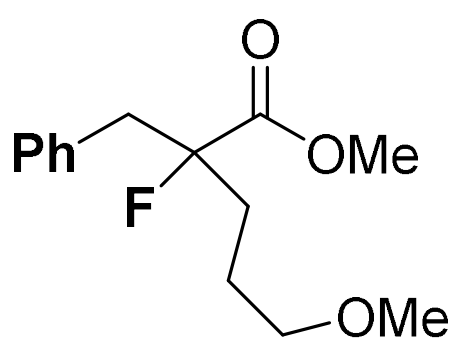

6u. Colorless oil (37.6 mg, 74% yield).

Analytical data for **6u**:

^{1}H NMR (400 MHz, $CDCl_3$) δ 7.33 – 7.24 (m, 3H), 7.24 – 7.19 (m, 2H), 3.68 (s, 3H), 3.43 – 3.35 (m, 2H), 3.33 (s, 3H), 3.20 (dd, *J* = 28.8, 14.3 Hz, 1H), 3.13 (dd, *J* = 19.5, 14.3 Hz, 1H), 2.18 – 1.99 (m, 1H), 1.98 – 1.86 (m, 1H), 1.86 – 1.72 (m, 1H), 1.62 – 1.50 (m, 1H).

^{13}C NMR (101 MHz, $CDCl_3$) δ 171.4 (d, *J* = 25.7 Hz), 134.5, 130.1, 128.3, 127.1, 97.7 (d, *J* = 191.2 Hz), 72.0, 58.5, 52.2, 43.5 (d, *J* = 21.5 Hz), 33.9 (d, *J* = 22.3 Hz), 23.6 (d, *J* = 3.1 Hz).

$^{19}F\{^{1}H\}$ NMR (377 MHz, $CDCl_3$) δ -165.10.

^{19}F NMR (377 MHz, $CDCl_3$) δ -165.10 (dddd, *J* = 32.2, 28.8, 19.5, 12.7 Hz).

IR (neat) ν_{max} (cm^{-1}) = 2952, 2873, 2870, 1761, 1736, 1497, 1456, 1445, 1348, 1277, 1248, 1214, 1200, 1117, 1030, 999, 958, 818, 746, 701, 677, 669, 602.

HRMS (ESI/QTOF) m/z: $[M + Na]^+$ Calcd for $C_{14}H_{19}FNaO_3^+$ 277.1210; Found 277.1215.

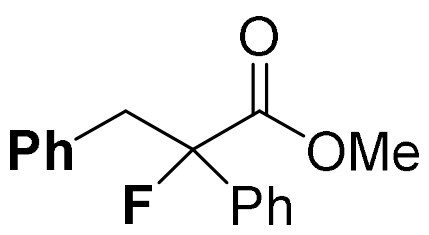

6v. Colorless oil (36.1 mg, 70% yield).

Analytical data for **6v**:

^{1}H NMR (500 MHz, $CDCl_3$) δ 7.59 – 7.56 (m, 2H), 7.43 – 7.37 (m, 3H), 7.30 – 7.25 (m, 3H), 7.25 – 7.20 (m, 2H), 3.72 (s, 3H), 3.69 (dd, *J* = 30.5, 14.5 Hz, 1H), 3.42 (dd, *J* = 20.0, 14.4 Hz, 1H).

^{13}C NMR (126 MHz, $CDCl_3$) δ 170.3 (d, *J* = 26.4 Hz), 137.9 (d, *J* = 22.5 Hz), 134.1, 130.5 (br), 128.6, 128.4 (d, *J* = 1.7 Hz), 128.1, 127.1, 124.7 (d, *J* = 9.4 Hz), 96.9 (d, *J* = 193.6 Hz), 52.8, 44.7 (d, *J* = 21.5 Hz).

$^{19}F\{^{1}H\}$ NMR (377 MHz, $CDCl_3$) δ -163.35.

^{19}F NMR (377 MHz, $CDCl_3$) δ -163.35 (dd, *J* = 30.3, 20.0 Hz).

IR (neat) ν_{max} (cm^{-1}) = 2988, 2932, 2868, 1684, 1669, 1653, 1559, 1507, 1457, 1380, 1330, 1164, 1143, 1096, 1081, 1063, 1008, 828, 728.

HRMS (ESI/QTOF) m/z: $[M + Na]^+$ Calcd for $C_{16}H_{15}FNaO_2^+$ 281.0948; Found 281.0956.

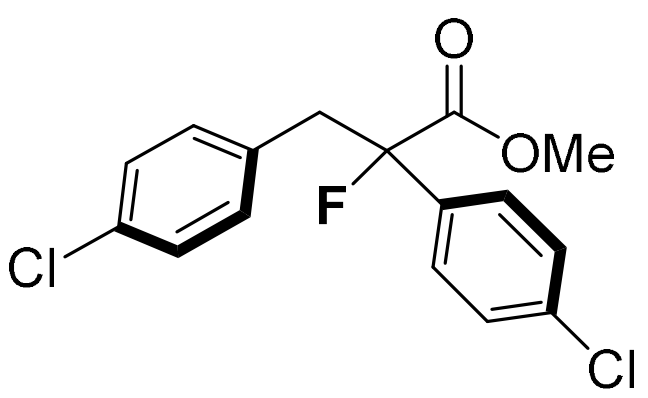

6w. Colorless oil (47.6 mg, 73% yield).

Analytical data for **6w**:

^{1}H NMR (500 MHz, $CDCl_3$) δ 7.45 (d, *J* = 8.5 Hz, 2H), 7.35 (d, *J* = 8.4 Hz, 2H), 7.22 (d, *J* = 8.2 Hz, 2H), 7.09 (d, *J* = 8.1 Hz, 2H), 3.71 (s, 3H), 3.58 (dd, *J* = 28.5, 14.5 Hz, 1H), 3.33 (dd, *J* = 21.5, 14.5 Hz, 1H).

^{13}C NMR (126 MHz, $CDCl_3$) δ 169.9 (d, J = 26.1 Hz), 135.9 (d, J = 22.9 Hz), 134.8 (br), 133.3, 132.2, 131.8 (br), 128.7 (d, J = 1.7 Hz), 128.4, 126.2 (d, J = 9.5 Hz), 96.4 (d, J = 194.5 Hz), 53.0, 44.0 (d, J = 21.5 Hz).

^{19}F{^{1}H} NMR (377 MHz, $CDCl_3$) δ -163.90.

^{19}F NMR (377 MHz, $CDCl_3$) δ -163.90 (dd, J = 28.5, 21.4 Hz).

IR (neat) ν_{max} (cm^{-1}) = 2988, 2869, 1762, 1740, 1491, 1437, 1401, 1271, 1205, 1142, 1092, 1046, 1016, 835, 803, 782, 761, 746, 717.

HRMS (ESI/QTOF) m/z: $[M + Na]^+$ Calcd for $C_{16}H_{13}Cl_2FNaO_2^+$ 349.0169; Found 349.0165.

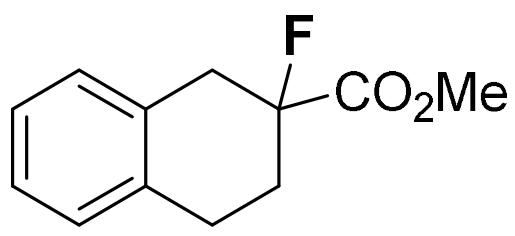

6x. Colorless oil (25.8 mg, 62% yield).

Analytical data for **6x**:

^{1}H NMR (600 MHz, $CDCl_3$) δ 7.20 – 7.12 (m, 3H), 7.11 – 7.07 (m, 1H), 3.85 (s, 3H), 3.38 (dd, J = 37.7, 17.7 Hz, 1H), 3.17 (td, J = 17.5, 2.6 Hz, 1H), 3.06 (ddd, J = 17.4, 11.8, 6.2 Hz, 1H), 2.90 – 2.81 (m, 1H), 2.30 (ddddd, J = 13.6, 7.9, 5.6, 3.2, 2.1 Hz, 1H), 2.26 – 2.09 (m, 1H).

^{13}C NMR (151 MHz, $CDCl_3$) δ 172.2 (d, J = 25.3 Hz), 134.5, 131.5, 129.1, 128.6, 126.4, 126.3, 93.2 (d, J = 186.1 Hz), 52.8, 36.5 (d, J = 24.4 Hz), 29.8 (d, J = 22.5 Hz), 24.6 (d, J = 4.7 Hz).

^{19}F{^{1}H} NMR (377 MHz, $CDCl_3$) δ -162.90.

^{19}F NMR (377 MHz, $CDCl_3$) δ -162.90 (tdd, J = 36.0, 16.9, 8.3 Hz).

IR (neat) ν_{max} (cm^{-1}) = 2930, 2863, 1669, 1507, 1457, 1380, 1330, 1300, 1276, 1164, 1143, 1096, 1081, 1063, 1008, 912, 828, 728.

HRMS (ESI/QTOF) m/z: $[M + Na]^+$ Calcd for $C_{12}H_{13}FNaO_2^+$ 231.0792; Found 231.0791.

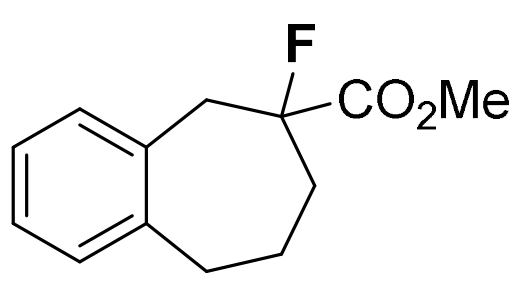

6y. Colorless oil (24.9 mg, 56% yield).

Analytical data for **6y**:

^{1}H NMR (400 MHz, $CDCl_3$) δ 7.21 – 7.09 (m, 4H), 3.78 (s, 3H), 3.45 (dd, J = 29.6, 14.5 Hz, 1H), 3.20 (ddd, J = 14.5, 3.9, 1.1 Hz, 1H), 2.90 – 2.80 (m, 2H), 2.33 – 2.13 (m, 2H), 1.93 – 1.84 (m, 1H), 1.82 – 1.71 (m, 1H).

^{13}C NMR (101 MHz, $CDCl_3$) δ 172.2 (d, J = 24.7 Hz), 142.4, 133.6 (d, J = 6.2 Hz), 131.1, 128.7, 127.4, 126.4, 93.0 (d, J = 187.8 Hz), 52.5, 42.4 (d, J = 24.9 Hz), 37.9 (d, J = 22.9 Hz), 34.9, 22.1 (d, J = 4.1 Hz).

^{19}**F{^{1}H} NMR** (377 MHz, $CDCl_3$) δ -156.43.

^{19}**F NMR** (377 MHz, $CDCl_3$) δ -156.34 (br).

IR (neat) ν_{max} (cm^{-1}) = 2929, 2856, 1665, 1486, 1445, 1365, 1299, 1275, 1183, 1156, 1139, 1072, 991, 951, 932, 913, 881, 819, 796, 714.

HRMS (ESI/QTOF) m/z: $[M + Na]^+$ Calcd for $C_{13}H_{15}FNaO_2^+$ 245.0948; Found 245.0948.

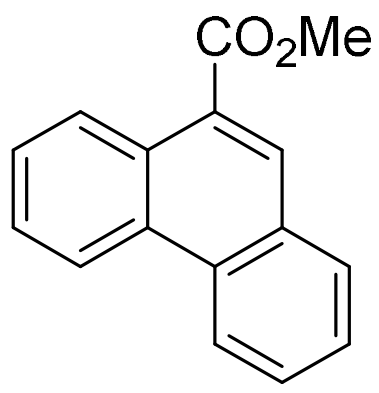

6z. Colorless oil (33.1 mg, 70% yield).

Analytical data for **6z**:

^{1}**H NMR** (400 MHz, $CDCl_3$) δ 8.96 – 8.89 (m, 1H), 8.77 – 8.72 (m, 1H), 8.69 (d, *J* = 8.4 Hz, 1H), 8.48 (s, 1H), 7.97 (d, *J* = 7.9 Hz, 1H), 7.78 – 7.61 (m, 4H), 4.05 (s, 3H).

^{13}**C NMR** (101 MHz, $CDCl_3$) δ 168.1, 132.4, 132.2, 130.7, 130.1, 129.9, 129.0, 128.9, 127.4, 127.0, 126.9, 126.6, 126.2, 122.8, 122.6, 52.3.

IR (neat) ν_{max} (cm^{-1}) = 2950, 1717, 1528, 1496, 1445, 1433, 1304, 1252, 1237, 1210, 1194, 1157, 1143, 1102, 1027, 804, 785, 743, 721, 619, 609.

HRMS (ESI/QTOF) m/z: $[M + Na]^+$ Calcd for $C_{16}H_{12}NaO_2^+$ 259.0730; Found 259.0731.

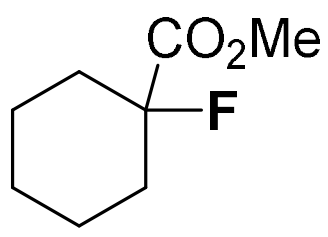

6aa. Colorless oil (10.2 mg, 32% yield).

Analytical data for **6aa**:

^{1}**H NMR** (500 MHz, $CDCl_3$) δ 3.76 (s, 3H), 1.98 – 1.90 (m, 2H), 1.88 – 1.73 (m, 2H), 1.71 – 1.57 (m, 5H), 1.35 – 1.26 (m, 1H).

^{13}**C NMR** (101 MHz, $CDCl_3$) δ 172.8 (d, *J* = 24.9 Hz), 94.0 (d, *J* = 185.7 Hz), 52.4, 32.5 (d, *J* = 22.6 Hz), 24.5, 20.9 (d, *J* = 1.8 Hz).

^{19}**F{^{1}H} NMR** (377 MHz, $CDCl_3$) δ -165.11.

^{19}**F NMR** (377 MHz, $CDCl_3$) δ -165.03 (br).

IR (neat) ν_{max} (cm^{-1}) = 2931, 1664, 1609, 1511, 1249, 1169, 1141, 1075, 1032, 950, 881, 839, 807, 685.

HRMS (ESI/QTOF) m/z: $[M + Na]^+$ Calcd for $C_8H_{13}FNaO_2^+$ 183.0792; Found 183.0798.

General procedure for hypervalent iodine based dyotropic rearrangement reactions: migrative acyloxylation

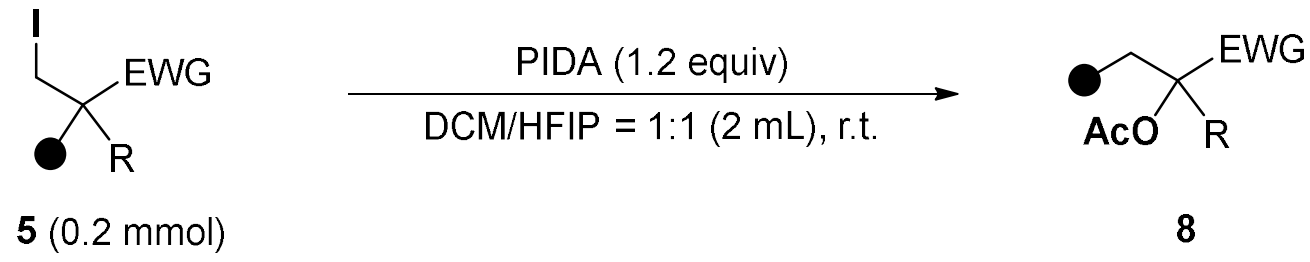

At room temperature, to a 10-mL test tube charged with a magnetic stirring bar were added substrates **5** (0.2 mmol, 1.0 equiv) and PIDA (77.3 mg, 0.24 mmol, 1.2 equiv) in anhydrous DCM (1.0 mL) and HFIP (1.0 mL). The reaction mixture was allowed to stir at room temperature. After the reaction was complete (monitored by TLC), the mixture was quenched with H_2O. Then, the mixture was extracted twice with DCM. The combined organic extracts were dried over Na_2SO_4 and concentrated under reduced pressure. The residue was purified by silica gel flash column chromatography to give the desired products **8**.

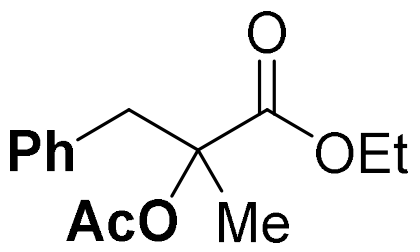

8a. Colorless oil (35.1 mg, 70% yield).

Analytical data for **8a**:

^{1}H NMR (500 MHz, $CDCl_3$) δ 7.25 – 7.17 (m, 3H), 7.12 – 7.08 (m, 2H), 4.09 (q, J = 7.1 Hz, 2H), 3.22 (d, J = 13.8 Hz, 1H), 2.96 (d, J = 13.8 Hz, 1H), 2.00 (s, 3H), 1.42 (s, 3H), 1.14 (t, J = 7.1 Hz, 3H).

^{13}C NMR (126 MHz, $CDCl_3$) δ 172.0, 170.0, 135.1, 130.5, 128.1, 127.0, 80.6, 61.3, 43.4, 21.23, 21.17, 14.0.

IR (neat) ν_{max} (cm^{-1}) = 2967, 2853, 1753, 1635, 1621, 1580, 1525, 1484, 1450, 1343, 1281, 1210, 1186, 1143, 1079, 975, 883, 810, 750, 692, 621.

HRMS (nanochip-ESI/LTQ-Orbitrap) m/z: $[M + Na]^+$ Calcd for $C_{14}H_{18}NaO_4^+$ 273.1097; Found 273.1099.

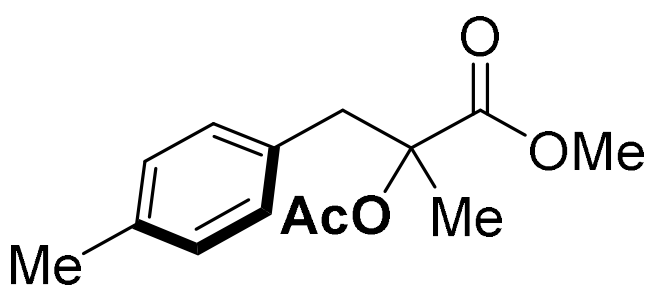

8b. Colorless oil (25.2 mg, 50% yield).

Analytical data for **8b**:

^{1}H NMR (600 MHz, $CDCl_3$) δ 7.10 (d, J = 7.8 Hz, 2H), 7.06 – 7.02 (m, 2H), 3.70 (s, 3H), 3.24 (d, J = 13.8 Hz, 1H), 3.00 (d, J = 13.8 Hz, 1H), 2.33 (s, 3H), 2.07 (s, 3H), 1.49 (s, 3H).

^{13}C NMR (151 MHz, $CDCl_3$) δ 172.6, 170.0, 136.7, 131.8, 130.3, 128.9, 80.7, 52.3, 43.0, 21.2, 21.09, 21.06.

IR (neat) ν_{max} (cm^{-1}) = 2998, 2943, 1752, 1627, 1605, 1533, 1512, 1431, 1329, 1242, 1187, 1149, 1038, 886, 858, 769, 614.

HRMS (ESI/QTOF) m/z: $[M + Na]^+$ Calcd for $C_{14}H_{18}NaO_4^+$ 273.1097; Found 273.1103.

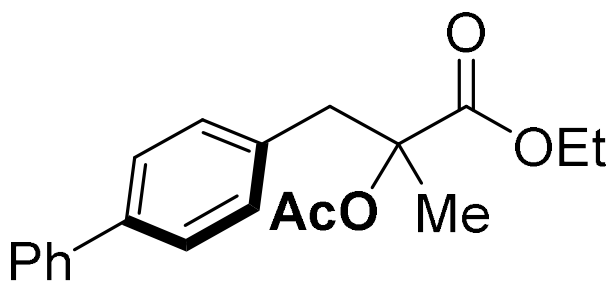

8c. Colorless oil (35.9 mg, 55% yield).

Analytical data for **8c**:

^{1}H NMR (500 MHz, $CDCl_3$) δ 7.61 – 7.57 (m, 2H), 7.55 – 7.50 (m, 2H), 7.47 – 7.41 (m, 2H), 7.37 – 7.32 (m, 1H), 7.26 – 7.23 (m, 2H), 4.18 (q, *J* = 7.1 Hz, 2H), 3.34 (d, *J* = 13.8 Hz, 1H), 3.08 (d, *J* = 13.8 Hz, 1H), 2.09 (s, 3H), 1.53 (s, 3H), 1.23 (t, *J* = 7.2 Hz, 3H).

^{13}C NMR (126 MHz, $CDCl_3$) δ 172.0, 170.0, 140.8, 139.9, 134.2, 131.0, 128.8, 127.3, 127.0, 126.8, 80.7, 61.4, 43.0, 21.27, 21.25, 14.0.

IR (neat) ν_{max} (cm^{-1}) = 2988, 2953, 2838, 2825, 1764, 1638, 1563, 1542, 1495, 1450, 1325, 1342, 1180, 1145, 910, 879, 750, 710, 691.

HRMS (nanochip-ESI/LTQ-Orbitrap) m/z: $[M + H]^+$ Calcd for $C_{20}H_{23}O_4^+$ 327.1591; Found 327.1586.

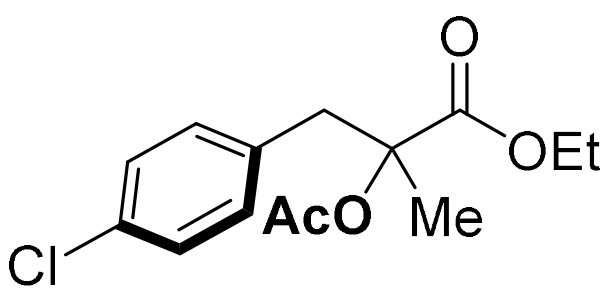

8d. Colorless oil (36.9 mg, 65% yield).

Analytical data for **8d**:

^{1}H NMR (600 MHz, $CDCl_3$) δ 7.21 – 7.19 (m, 2H), 7.04 – 7.02 (m, 2H), 4.09 (q, *J* = 7.1 Hz, 2H), 3.19 (d, *J* = 13.9 Hz, 1H), 2.93 (d, *J* = 13.9 Hz, 1H), 1.99 (s, 3H), 1.41 (s, 3H), 1.15 (t, *J* = 7.1 Hz, 3H).

^{13}C NMR (151 MHz, $CDCl_3$) δ 171.7, 169.8, 133.6, 133.0, 131.8, 128.3, 80.4, 61.4, 42.7, 21.24, 21.19, 14.0.

IR (neat) ν_{max} (cm^{-1}) = 2990, 2981, 1754, 1725, 1680, 1657, 1549, 1538, 1492, 1488, 1456, 1375, 1270, 1198, 1115, 1090, 1023, 951, 836, 801, 734, 714, 660.

HRMS (nanochip-ESI/LTQ-Orbitrap) m/z: $[M + Na]^+$ Calcd for $C_{14}H_{17}ClNaO_4^+$ 307.0708; Found 307.0708.

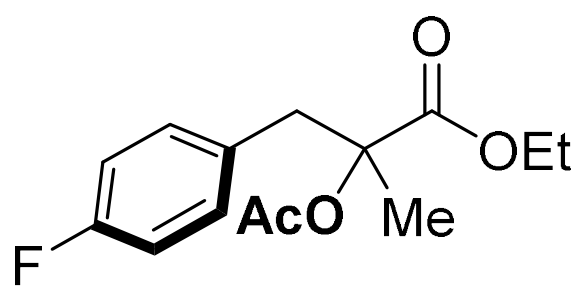

8e. Colorless oil (36.0 mg, 67% yield).

Analytical data for **8e**:

^{1}H NMR (400 MHz, $CDCl_3$) δ 7.16 – 7.10 (m, 2H), 7.01 – 6.96 (m, 2H), 4.15 (q, *J* = 7.1 Hz, 2H), 3.24 (d, *J* = 14.0 Hz, 1H), 3.00 (d, *J* = 14.0 Hz, 1H), 2.06 (s, 3H), 1.48 (s, 3H), 1.21 (t, *J* = 7.1 Hz, 3H).

^{13}C NMR (101 MHz, $CDCl_3$) δ 171.8, 169.9, 162.1 (d, *J* = 245.3 Hz), 131.9 (d, *J* = 7.8 Hz), 130.8 (d, *J* = 3.4 Hz), 115.0 (d, *J* = 21.3 Hz), 80.5, 61.3, 42.6, 21.167, 21.161, 14.0.

^{19}F{^{1}H} NMR (377 MHz, $CDCl_3$) δ -115.87.

^{19}F NMR (377 MHz, $CDCl_3$) δ -115.83 – -115.90 (m).

IR (neat) ν_{max} (cm^{-1}) = 2984, 2953, 2942, 2860, 1743, 1652, 1615, 1483, 1344, 1283, 1254, 1261, 1132, 895, 858, 778, 618, 623.

HRMS (ESI/QTOF) m/z: $[M + Na]^+$ Calcd for $C_{14}H_{17}FNaO_4^+$ 291.1003; Found 291.1005.

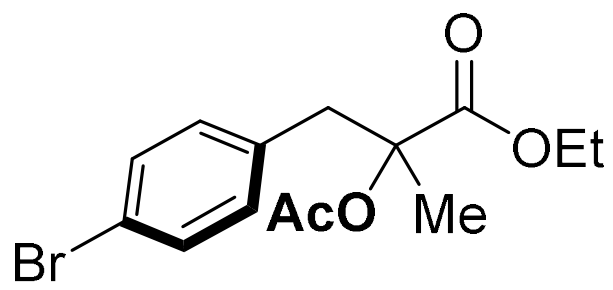

8f. Colorless oil (45.3 mg, 69% yield).

Analytical data for **8f**:

^{1}H NMR (400 MHz, $CDCl_3$) δ 7.45 – 7.38 (m, 2H), 7.07 – 7.01 (m, 2H), 4.16 (q, *J* = 7.1 Hz, 2H), 3.24 (d, *J* = 13.9 Hz, 1H), 2.98 (d, *J* = 13.9 Hz, 1H), 2.06 (s, 3H), 1.48 (s, 3H), 1.21 (t, *J* = 7.1 Hz, 3H).

^{13}C NMR (101 MHz, $CDCl_3$) δ 171.7, 169.8, 134.1, 132.2, 131.2, 121.1, 80.3, 61.4, 42.7, 21.24, 21.17, 14.0.

IR (neat) ν_{max} (cm^{-1}) = 2922, 2913, 1655, 1644, 1575, 1533, 1520, 1494, 1456, 1390, 1363, 1140, 1091, 918, 745, 662.

HRMS (ESI/QTOF) m/z: $[M + Na]^+$ Calcd for $C_{14}H_{17}BrNaO_4^+$ 351.0202; Found 351.0203.

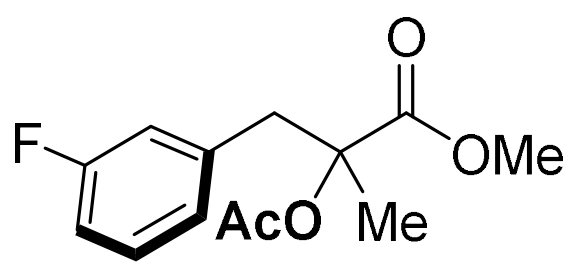

8g. Colorless oil (35.6 mg, 70% yield).

Analytical data for **8g**:

^{1}**H NMR** (400 MHz, $CDCl_3$) δ 7.19 (td, J = 8.4, 6.1 Hz, 1H), 6.94 – 6.87 (m, 1H), 6.86 (dt, J = 7.6, 1.4 Hz, 1H), 6.81 (dt, J = 9.9, 2.3 Hz, 1H), 3.63 (s, 3H), 3.21 (d, J = 13.8 Hz, 1H), 2.96 (d, J = 13.8 Hz, 1H), 2.00 (s, 3H), 1.43 (s, 3H).

^{13}**C NMR** (101 MHz, $CDCl_3$) δ 172.2, 169.9, 162.6 (d, J = 245.6 Hz), 137.5 (d, J = 7.5 Hz), 129.5 (d, J = 8.3 Hz), 126.2 (d, J = 2.9 Hz), 117.3 (d, J = 21.2 Hz), 114.0 (d, J = 21.0 Hz), 80.4, 52.4, 43.1 (d, J = 1.8 Hz), 21.3, 21.2.

^{19}**F{^{1}H} NMR** (377 MHz, $CDCl_3$) δ -113.60.

^{19}**F NMR** (377 MHz, $CDCl_3$) δ -113.60 (app td, J = 9.4, 6.0 Hz).

IR (neat) ν_{max} (cm^{-1}) = 2966, 2879, 1735, 1646, 1657, 1583, 1522, 1433, 1263, 1218, 1183, 1146, 1130, 1081, 884, 828, 813, 776, 746, 732, 632.

HRMS (ESI/QTOF) m/z: $[M + Na]^+$ Calcd for $C_{13}H_{15}FNaO_4^+$ 277.0847; Found 277.0853.

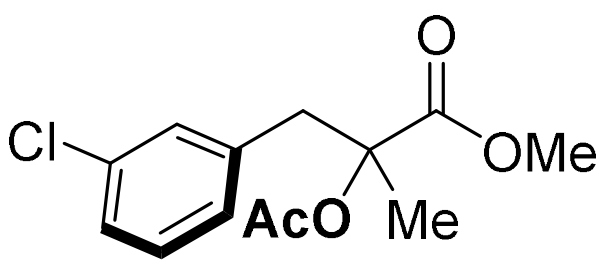

8h. Colorless oil (33.5 mg, 62% yield).

Analytical data for **8h**:

^{1}**H NMR** (600 MHz, $CDCl_3$) δ 7.32 – 7.26 (m, 2H), 7.20 (t, J = 2.0 Hz, 1H), 7.08 (dt, J = 7.1, 1.6 Hz, 1H), 3.75 (s, 3H), 3.31 (d, J = 13.9 Hz, 1H), 3.05 (d, J = 13.9 Hz, 1H), 2.12 (s, 3H), 1.54 (s, 3H).

^{13}**C NMR** (151 MHz, $CDCl_3$) δ 172.2, 169.9, 137.0, 134.0, 130.5, 129.4, 128.6, 127.3, 80.3, 52.4, 43.0, 21.3, 21.2.

IR (neat) ν_{max} (cm^{-1}) = 2956, 2929, 2854, 1634, 1611, 1539, 1486, 1479, 1435, 1382, 1380, 1360, 1348, 1293, 1157, 1093, 1088, 897, 748, 710, 663.

HRMS (ESI/QTOF) m/z: $[M + Na]^+$ Calcd for $C_{13}H_{15}ClNaO_4^+$ 293.0551; Found 293.0557.

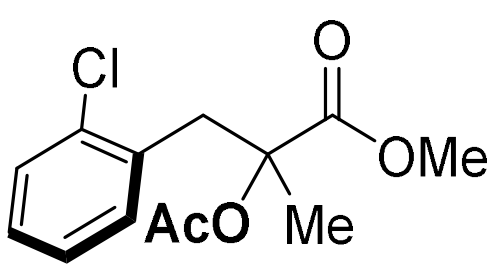

8i. Colorless oil (24.8 mg, 46% yield).

Analytical data for **8i**:

^{1}**H NMR** (500 MHz, $CDCl_3$) δ 7.40 – 7.35 (m, 1H), 7.25 – 7.18 (m, 3H), 3.73 (s, 3H), 3.43 (d, J = 14.2 Hz, 1H), 3.34 (d, J = 14.2 Hz, 1H), 2.08 (s, 3H), 1.54 (s, 3H).

^{13}**C NMR** (126 MHz, $CDCl_3$) δ 172.2, 170.0, 135.3, 133.2, 132.7, 129.7, 128.5, 126.4, 80.7, 52.5, 39.6, 21.3,

21.1.

IR (neat) ν_{max} (cm^{-1}) = 2998, 2931, 2859, 1688, 1676, 1642, 1569, 1563, 1542, 1497, 1436, 1411, 1375, 1333, 1290, 1141, 903, 887, 742, 710, 682.

HRMS (ESI/QTOF) m/z: $[M + Na]^+$ Calcd for $C_{13}H_{15}ClNaO_4^+$ 293.0551; Found 293.0557.

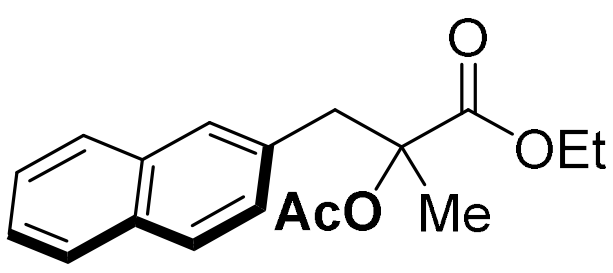

8j. Colorless oil (36.0 mg, 60% yield).

Analytical data for **8j**:

^{1}H NMR (500 MHz, $CDCl_3$) δ 7.84 – 7.78 (m, 2H), 7.78 (d, *J* = 8.4 Hz, 1H), 7.63 (brs, 1H), 7.50 – 7.43 (m, 2H), 7.32 (dd, *J* = 8.5, 1.7 Hz, 1H), 4.18 (q, *J* = 7.1 Hz, 2H), 3.47 (d, *J* = 13.9 Hz, 1H), 3.21 (d, *J* = 13.9 Hz, 1H), 2.09 (s, 3H), 1.54 (s, 3H), 1.20 (t, *J* = 7.1 Hz, 3H).

^{13}C NMR (126 MHz, $CDCl_3$) δ 172.0, 170.0, 133.2, 132.7, 132.4, 129.4, 128.7, 127.59, 127.57, 126.0, 125.7, 80.8, 61.4, 43.5, 21.32, 21.25, 14.0.

IR (neat) ν_{max} (cm^{-1}) = 2998, 2956, 2879, 1756, 1626, 1549, 1481, 1438, 1375, 1382, 1352, 1338, 1311, 1298, 1240, 1152, 1096, 886, 754, 694.

HRMS (nanochip-ESI/LTQ-Orbitrap) m/z: $[M + H]^+$ Calcd for $C_{18}H_{21}O_4^+$ 301.1434; Found 301.1431.

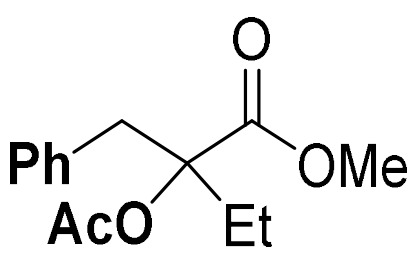

8k. Colorless oil (31.5 mg, 63% yield).

Analytical data for **8k**:

^{1}H NMR (500 MHz, $CDCl_3$) δ 7.24 – 7.17 (m, 3H), 7.05 – 7.01 (m, 2H), 3.63 (s, 3H), 3.40 (d, *J* = 14.3 Hz, 1H), 3.11 (d, *J* = 14.3 Hz, 1H), 2.00 (s, 3H), 1.89 (dt, *J* = 15.1, 7.5 Hz, 1H), 1.76 (dq, *J* = 14.8, 7.5 Hz, 1H), 0.84 (t, *J* = 7.6 Hz, 3H).

^{13}C NMR (126 MHz, $CDCl_3$) δ 171.9, 170.1, 135.3, 130.2, 128.2, 126.9, 83.6, 52.1, 38.5, 27.0, 21.2, 7.7.

IR (neat) ν_{max} (cm^{-1}) = 2975, 2941, 1755, 1745, 1720, 1567, 1488, 1458, 1384, 1255, 1195, 1144, 1131, 1037, 978, 868, 824, 708, 671, 666, 605.

HRMS (nanochip-ESI/LTQ-Orbitrap) m/z: $[M + Na]^+$ Calcd for $C_{14}H_{18}NaO_4^+$ 273.1097; Found 273.1100.

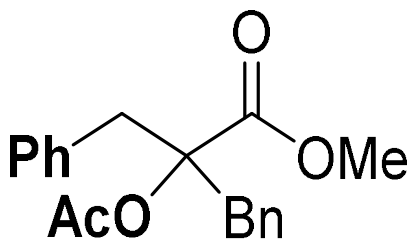

8l. Colorless oil (38.1 mg, 61% yield).

Analytical data for **8l**:

^{1}H NMR (500 MHz, $CDCl_3$) δ 7.25 – 7.17 (m, 6H), 7.10 – 7.06 (m, 4H), 3.46 (s, 3H), 3.36 (d, *J* = 14.2 Hz, 2H), 3.14 (d, *J* = 14.2 Hz, 2H), 1.98 (s, 3H).

^{13}C NMR (126 MHz, $CDCl_3$) δ 171.1, 170.3, 135.2, 130.3, 128.2, 127.0, 83.8, 52.0, 40.5, 21.5.

IR (neat) ν_{max} (cm^{-1}) = 2986, 2876, 1762, 1734, 1581, 1566, 1467, 1389, 1299, 1676, 1189, 1185, 1069, 973, 822, 757, 738, 629, 610.

HRMS (nanochip-ESI/LTQ-Orbitrap) m/z: $[M + Na]^+$ Calcd for $C_{19}H_{20}NaO_4^+$ 335.1254; Found 335.1256.

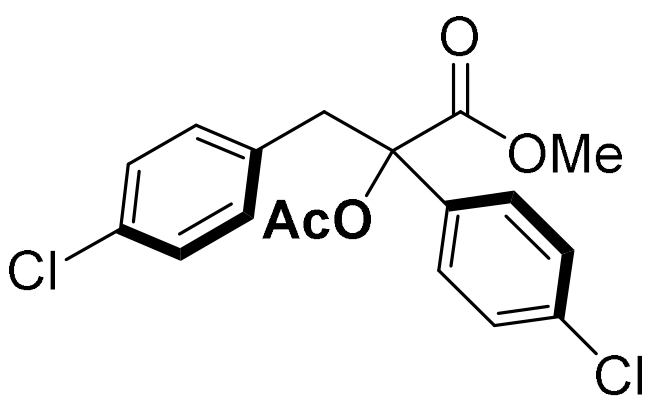

8m. Colorless oil (35.1 mg, 48% yield).

Analytical data for **8m**:

^{1}H NMR (600 MHz, $CDCl_3$) δ 7.33 – 7.29 (m, 2H), 7.29 – 7.26 (m, 2H), 7.10 – 7.07 (m, 2H), 6.59 – 6.55 (m, 2H), 3.94 (d, *J* = 14.5 Hz, 1H), 3.72 (s, 3H), 3.47 (d, *J* = 14.5 Hz, 1H), 2.15 (s, 3H).

^{13}C NMR (151 MHz, $CDCl_3$) δ 170.7, 169.7, 135.8, 134.3, 133.0, 132.9, 131.4, 128.7, 128.1, 126.6, 83.1, 53.0, 41.2, 21.2.

IR (neat) ν_{max} (cm^{-1}) = 2983, 2951, 1751, 1717, 1700, 1653, 1599, 1567, 1488, 1349, 1336, 1250, 1135, 1121, 1111, 1097, 987, 881, 770, 745, 653, 636.

HRMS (ESI/QTOF) m/z: $[M + Na]^+$ Calcd for $C_{18}H_{16}Cl_2NaO_4^+$ 389.0318; Found 389.0323.

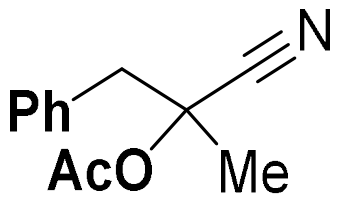

8n. Colorless oil (16.2 mg, 40% yield).

Analytical data for **8n**:

^{1}H NMR (400 MHz, $CDCl_3$) δ 7.39 – 7.32 (m, 3H), 7.31-7.27 (m, 2H), 3.32 (d, *J* = 13.7 Hz, 1H), 3.20 (d, *J* = 13.8 Hz, 1H), 2.10 (s, 3H), 1.71 (s, 3H).

^{13}C NMR (101 MHz, $CDCl_3$) δ 168.8, 133.0, 130.7, 128.5, 127.8, 118.5, 71.7, 44.8, 24.2, 21.2.

IR (neat) ν_{max} (cm^{-1}) = 2968, 2234, 1766, 1725, 1600, 1588, 1537, 1437, 1406, 1401, 1378, 1325, 1282, 1122, 1020, 985, 854, 827, 778, 667, 632.

HRMS (ESI/QTOF) m/z: $[M + Na]^+$ Calcd for $C_{12}H_{13}NNaO_2^+$ 226.0838; Found 226.0842.

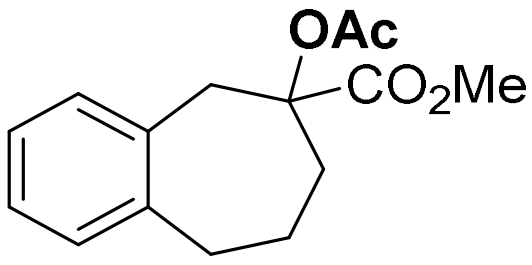

8o. Colorless oil (16.2 mg, 31% yield).

Analytical data for **8o**:

^{1}H NMR (600 MHz, $CDCl_3$) δ 7.14 (td, *J* = 7.3, 1.4 Hz, 1H), 7.10-7.07 (m, 2H), 7.02 – 6.99 (m, 1H), 3.75 (s, 3H), 3.67 (dd, *J* = 14.5, 1.9 Hz, 1H), 3.36 (d, *J* = 14.5 Hz, 1H), 2.88 (ddd, *J* = 13.9, 11.5, 2.0 Hz, 1H), 2.79 (ddd, *J* = 14.4, 7.1, 2.0 Hz, 1H), 2.24 (dp, *J* = 13.7, 2.4 Hz, 1H), 2.01 (ddd, *J* = 13.8, 12.3, 3.9 Hz, 1H), 1.94 – 1.87 (m, 1H), 1.76 (s, 3H), 1.77-1.70 (m, 1H).

^{13}C NMR (151 MHz, $CDCl_3$) δ 173.3, 169.9, 142.6, 135.1, 130.7, 128.5, 127.0, 126.1, 78.9, 52.4, 39.6, 39.5, 35.1, 22.3, 20.6.

IR (neat) ν_{max} (cm^{-1}) = 2988, 2896, 1742, 1740, 1666, 1588, 1564, 1539, 1522, 1457, 1430, 1371, 1332, 1271, 1251, 1132, 1098, 954, 866, 788, 723, 685, 617.

HRMS (ESI/QTOF) m/z: $[M + Na]^+$ Calcd for $C_{15}H_{18}NaO_4^+$ 285.1097; Found 285.1104.

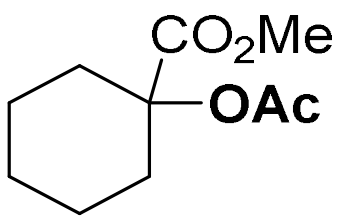

8p. Colorless oil (11.8 mg, 30% yield).

Analytical data for **8p**:

^{1}H NMR (600 MHz, $CDCl_3$) δ 3.70 (s, 3H), 2.13 – 2.09 (m, 2H), 2.08 (s, 3H), 1.79 – 1.74 (m, 2H), 1.64 – 1.59 (m, 3H), 1.55 – 1.48 (m, 2H), 1.32 – 1.25 (m, 1H).

^{13}C NMR (151 MHz, $CDCl_3$) δ 173.2, 169.9, 79.9, 52.2, 32.1, 25.0, 21.2, 21.1.

IR (neat) ν_{max} (cm^{-1}) = 2956, 2914, 1722, 1684, 1629, 1531, 1269, 1179, 1175, 1052, 954, 883, 869, 837, 754, 732, 675.

HRMS (ESI/QTOF) m/z: $[M + Na]^+$ Calcd for $C_{10}H_{16}NaO_4^+$ 223.0941; Found 223.0931.

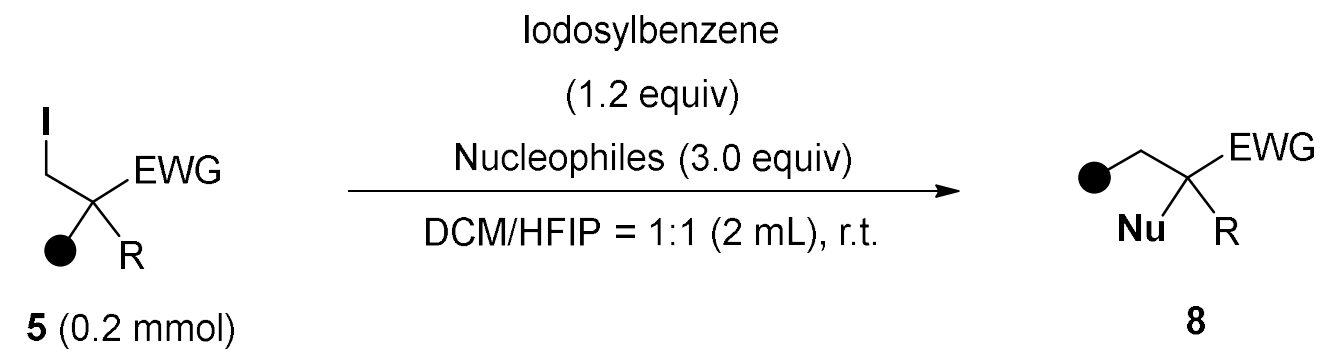

At room temperature, to a 10-mL test tube charged with a magnetic stirring bar were added substrates **5** (0.2 mmol, 1.0 equiv), iodosylbenzene (52.8 mg, 0.24 mmol, 1.2 equiv) and the nucleophiles (0.6 mmol, 3.0 equiv) in anhydrous DCM (1.0 mL) and HFIP (1.0 mL). The reaction mixture was allowed to stir at room temperature. After the reaction was complete (monitored by TLC), the mixture was quenched with H_2O. Then, the mixture was extracted twice with DCM. The combined organic extracts were dried over Na_2SO_4 and concentrated under reduced pressure. The residue was purified by silica gel flash column chromatography to give the desired products **8**.

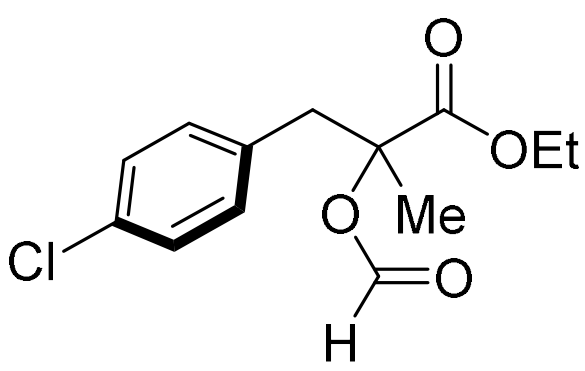

8q. Colorless oil (32.4 mg, 60% yield).

Analytical data for **8q**:

^{1}H NMR (400 MHz, $CDCl_3$) δ 7.91 (s, 1H), 7.23 – 7.17 (m, 2H), 7.09 – 7.02 (m, 2H), 4.10 (q, *J* = 7.1 Hz, 2H), 3.18 (d, *J* = 14.0 Hz, 1H), 2.98 (d, *J* = 14.0 Hz, 1H), 1.48 (s, 3H), 1.15 (t, *J* = 7.1 Hz, 3H).

^{13}C NMR (101 MHz, $CDCl_3$) δ 171.0, 159.5, 133.2, 133.1, 131.8, 128.4, 81.1, 61.7, 43.0, 21.3, 13.9.

IR (neat) ν_{max} (cm^{-1}) = 2967, 2874, 1763, 1675, 1498, 1331, 1256, 1179, 1095, 1052, 1036, 997, 883, 781, 746, 727, 658, 629.

HRMS (APCI/QTOF) m/z: $[M + Na]^+$ Calcd for $C_{13}H_{15}ClNaO_4^+$ 293.0551; Found 293.0550.

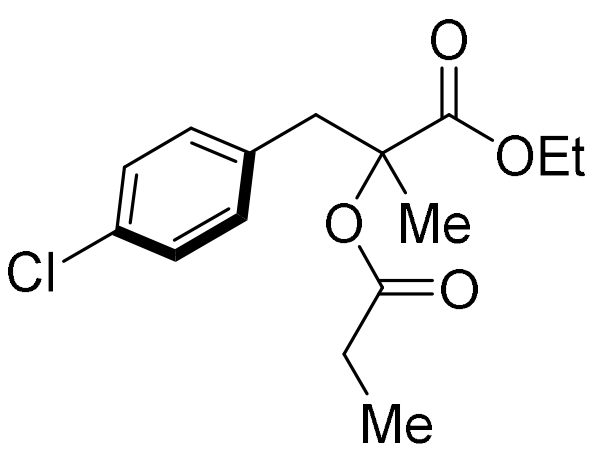

8r. Colorless oil (40.0 mg, 67% yield).

Analytical data for **8r**:

^{1}H NMR (400 MHz, $CDCl_3$) δ 7.28 – 7.24 (m, 2H), 7.12 – 7.07 (m, 2H), 4.15 (q, *J* = 7.1 Hz, 2H), 3.26 (d, *J*

= 13.9 Hz, 1H), 3.00 (d, J = 13.9 Hz, 1H), 2.33 (q, J = 7.5 Hz, 2H), 1.48 (s, 3H), 1.21 (t, J = 7.1 Hz, 3H), 1.13 (t, J = 7.6 Hz, 3H).

^{13}C NMR (101 MHz, $CDCl_3$) δ 173.3, 171.8, 133.7, 133.0, 131.8, 128.2, 80.2, 61.4, 42.8, 27.8, 21.3, 14.0, 9.0.

IR (neat) ν_{max} (cm^{-1}) = 2969, 2846, 1756, 1685, 1567, 1457, 1385, 1295, 1186, 1159, 971, 921, 891, 812, 786, 704, 655.

HRMS (ESI/QTOF) m/z: $[M + Na]^+$ Calcd for $C_{15}H_{19}ClNaO_4^+$ 321.0864; Found 321.0872.

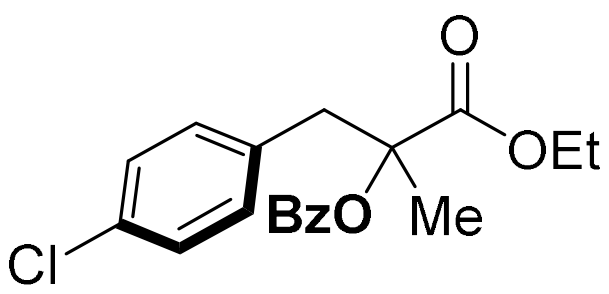

8s. Colorless oil (42.9 mg, 62% yield).

Analytical data for **8s**:

^{1}H NMR (500 MHz, $CDCl_3$) δ 7.94 – 7.91 (m, 2H), 7.53 – 7.49 (m, 1H), 7.41 – 7.34 (m, 2H), 7.22 – 7.17 (m, 2H), 7.11 – 7.05 (m, 2H), 4.13 (q, J = 7.1 Hz, 2H), 3.37 (d, J = 14.0 Hz, 1H), 3.08 (d, J = 14.0 Hz, 1H), 1.54 (s, 3H), 1.13 (t, J = 7.1 Hz, 3H).

^{13}C NMR (126 MHz, $CDCl_3$) δ 171.8, 165.4, 133.6, 133.2, 133.1, 131.9, 130.0, 129.7, 128.4, 128.3, 80.8, 61.5, 42.7, 21.4, 14.0.

IR (neat) ν_{max} (cm^{-1}) = 2983, 2879, 1732, 1654, 1542, 1491, 1451, 1344, 1265, 1148, 1087, 1042, 934, 883, 768, 721, 695, 621.

HRMS (ESI/QTOF) m/z: $[M + Na]^+$ Calcd for $C_{19}H_{19}ClNaO_4^+$ 369.0864; Found 369.0868.

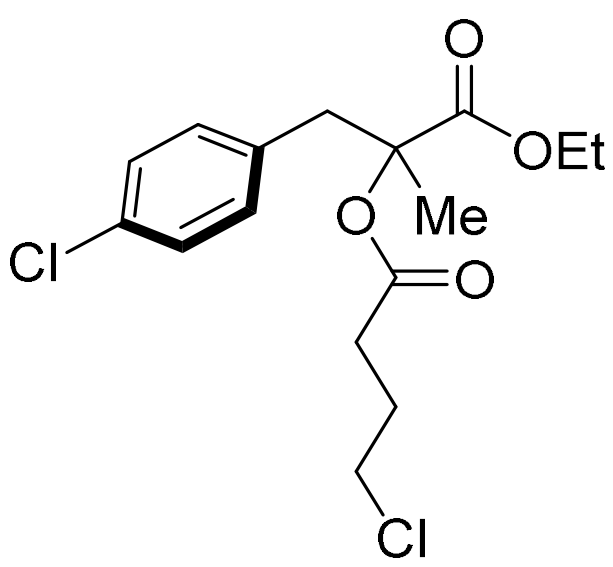

8t. Colorless oil (34.6 mg, 50% yield).

Analytical data for **8t**:

^{1}H NMR (500 MHz, $CDCl_3$) δ 7.28 – 7.25 (m, 2H), 7.11 – 7.07 (m, 2H), 4.14 (qd, J = 7.2, 0.8 Hz, 2H), 3.58 (td, J = 6.5, 1.1 Hz, 2H), 3.24 (d, J = 13.9 Hz, 1H), 3.00 (d, J = 13.9 Hz, 1H), 2.57 – 2.44 (m, 2H), 2.09 – 2.04 (m, 2H), 1.49 (s, 3H), 1.20 (t, J = 7.2 Hz, 3H).

^{13}C NMR (126 MHz, $CDCl_3$) δ 171.6, 171.5, 133.5, 133.1, 131.8, 128.3, 80.7, 61.5, 43.8, 42.9, 31.3, 27.6, 21.3, 14.0.

IR (neat) ν_{max} (cm^{-1}) = 2988, 2816, 1752, 1654, 1619, 1501, 1469, 1365, 1279, 1238, 1179, 1065, 1009, 952, 869, 827, 771, 704, 695.

HRMS (ESI/QTOF) m/z: $[M + Na]^+$ Calcd for $C_{16}H_{20}Cl_2NaO_4^+$ 369.0631; Found 369.0634.

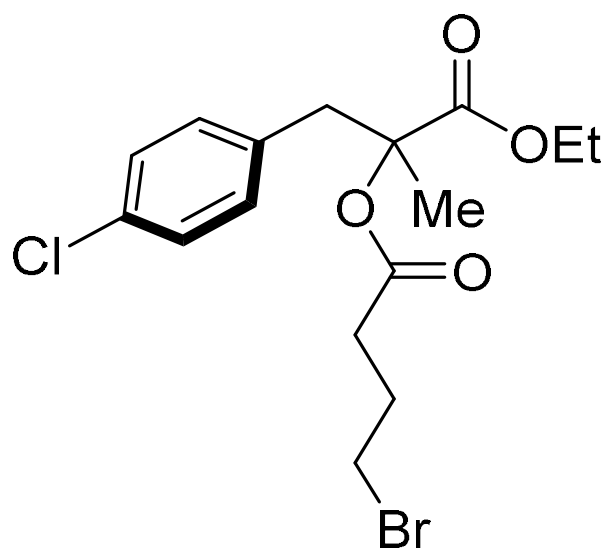

8u. Colorless oil (43.7 mg, 56% yield).

Analytical data for **8u**:

^{1}H NMR (600 MHz, $CDCl_3$) δ 7.29 – 7.26 (m, 2H), 7.12 – 7.08 (m, 2H), 4.15 (qd, *J* = 7.1, 1.0 Hz, 2H), 3.46 (td, *J* = 6.5, 1.6 Hz, 2H), 3.25 (d, *J* = 14.0 Hz, 1H), 3.01 (d, *J* = 14.0 Hz, 1H), 2.56 – 2.47 (m, 2H), 2.18 – 2.13 (m, 2H), 1.50 (s, 3H), 1.21 (t, *J* = 7.1 Hz, 3H).

^{13}C NMR (151 MHz, $CDCl_3$) δ 171.6, 171.4, 133.5, 133.1, 131.8, 128.3, 80.7, 61.5, 42.9, 32.6, 32.4, 27.7, 21.4, 14.0.

IR (neat) ν_{max} (cm^{-1}) = 2981, 2848, 1762, 1676, 1458, 1365, 1293, 1228, 1172, 1088, 1045, 992, 938, 942, 862, 837, 855, 768, 645.

HRMS (ESI/QTOF) m/z: $[M + Na]^+$ Calcd for $C_{16}H_{20}BrClNaO_4^+$ 413.0126; Found 413.0134.

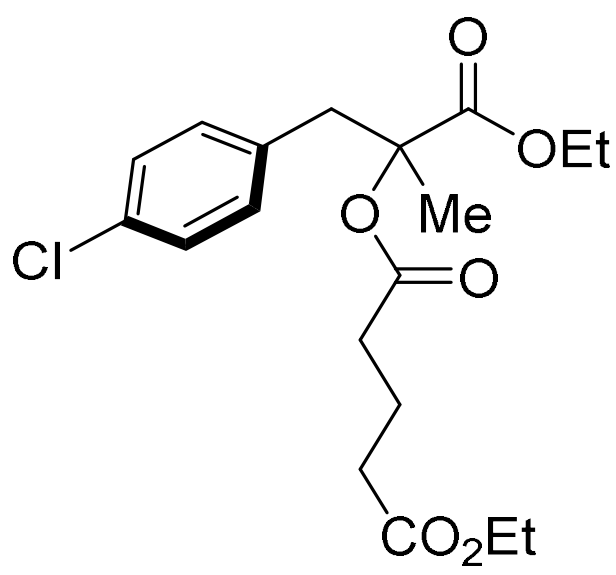

8v. Colorless oil (39.2 mg, 51% yield).

Analytical data for **8v**:

^{1}H NMR (600 MHz, $CDCl_3$) δ 7.28 – 7.24 (m, 2H), 7.11 – 7.07 (m, 2H), 4.18 – 4.10 (m, 4H), 3.24 (d, *J* = 13.9 Hz, 1H), 3.00 (d, *J* = 14.0 Hz, 1H), 2.40 – 2.34 (m, 4H), 1.93 (p, *J* = 7.1 Hz, 2H), 1.49 (s, 3H), 1.25 (t, *J* = 7.1 Hz, 3H), 1.21 (t, *J* = 7.1 Hz, 3H).

^{13}C NMR (151 MHz, $CDCl_3$) δ 172.9, 171.8, 171.6, 133.6, 133.1, 131.8, 128.3, 80.5, 61.4, 60.4, 42.9, 33.5, 33.1, 21.3, 20.1, 14.2, 14.0.

IR (neat) ν_{max} (cm^{-1}) = 2979, 2913, 2859, 1755, 1685, 1633, 1582, 1456, 1328, 1265, 1211, 1172, 1074, 956, 867, 843, 824, 735.

HRMS (ESI/QTOF) m/z: $[M + Na]^+$ Calcd for $C_{19}H_{25}ClNaO_6^+$ 407.1232; Found 407.1226.

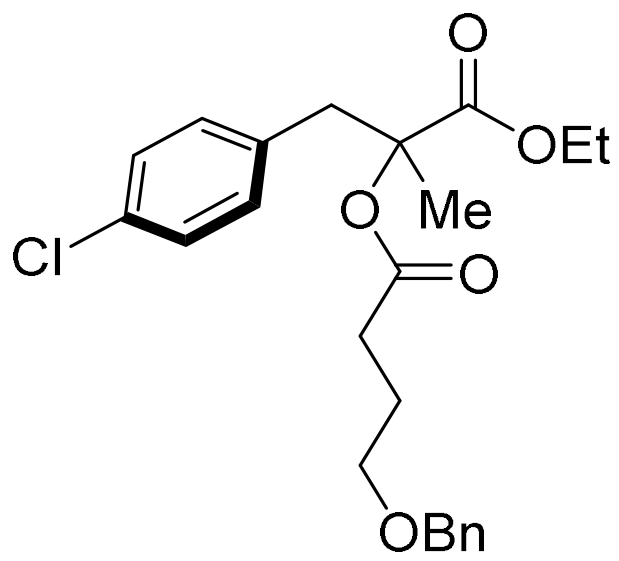

8w. Colorless oil (51.9 mg, 62% yield).

Analytical data for **8w**:

^{1}H NMR (600 MHz, $CDCl_3$) δ 7.36 – 7.31 (m, 4H), 7.30 – 7.27 (m, 1H), 7.26 – 7.23 (m, 2H), 7.11 – 7.07 (m, 2H), 4.49 (s, 2H), 4.14 (q, *J* = 7.1 Hz, 2H), 3.50 (t, *J* = 6.2 Hz, 2H), 3.23 (d, *J* = 13.9 Hz, 1H), 2.99 (d, *J* = 13.9 Hz, 1H), 2.44 (td, *J* = 7.3, 1.2 Hz, 2H), 1.92 (tt, *J* = 7.4, 6.2 Hz, 2H), 1.47 (s, 3H), 1.20 (t, *J* = 7.1 Hz, 3H).

^{13}C NMR (151 MHz, $CDCl_3$) δ 172.3, 171.7, 138.3, 133.6, 133.0, 131.8, 128.4, 128.3, 127.6, 80.3, 72.9, 69.0, 61.4, 42.9, 31.2, 25.0, 21.3, 14.0.

IR (neat) ν_{max} (cm^{-1}) = 2986, 2964, 2839, 1754, 1656, 1433, 1352, 1284, 1162, 1150, 1082, 1047, 1021, 933, 852, 768, 685.

HRMS (nanochip-ESI/LTQ-Orbitrap) m/z: $[M + Na]^+$ Calcd for $C_{23}H_{27}ClNaO_5^+$ 441.1439; Found 441.1456.

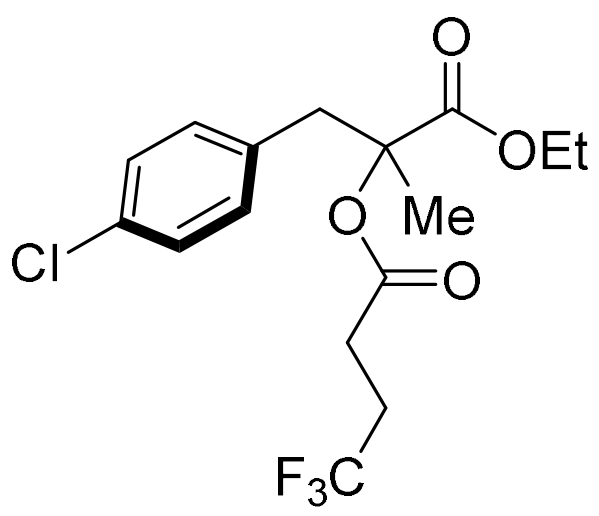

8x. Colorless oil (42.5 mg, 58% yield).

Analytical data for **8x**:

^{1}H NMR (600 MHz, $CDCl_3$) δ 7.29 – 7.26 (m, 2H), 7.10 – 7.07 (m, 2H), 4.16 (q, *J* = 7.1 Hz, 2H), 3.24 (d, *J* = 14.0 Hz, 1H), 3.02 (d, *J* = 13.9 Hz, 1H), 2.59 (td, *J* = 7.3, 2.0 Hz, 2H), 2.48 – 2.38 (m, 2H), 1.51 (s, 3H),

1.21 (t, J = 7.2 Hz, 3H).

^{13}C NMR (151 MHz, $CDCl_3$) δ 171.3, 169.8, 133.3, 133.2, 131.7, 128.4, 126.4 (d, J = 276.0 Hz), 81.3, 61.6, 43.0, 29.1 (q, J = 30.2 Hz), 27.3 (q, J = 3.2 Hz), 21.2, 13.9.

^{19}F{^{1}H} NMR (377 MHz, $CDCl_3$) δ -66.95.

^{19}F NMR (377 MHz, $CDCl_3$) δ -66.95 (t, J = 10.4 Hz).

IR (neat) ν_{max} (cm^{-1}) = 2995, 2878, 1763, 1686, 1564, 1477, 1430, 1360, 1270, 1239, 1177, 1108, 1062, 1114, 941, 881, 831, 732, 676.

HRMS (nanochip-ESI/LTQ-Orbitrap) m/z: $[M + Na]^+$ Calcd for $C_{16}H_{18}ClF_3NaO_4^+$ 389.0738; Found 389.0756.

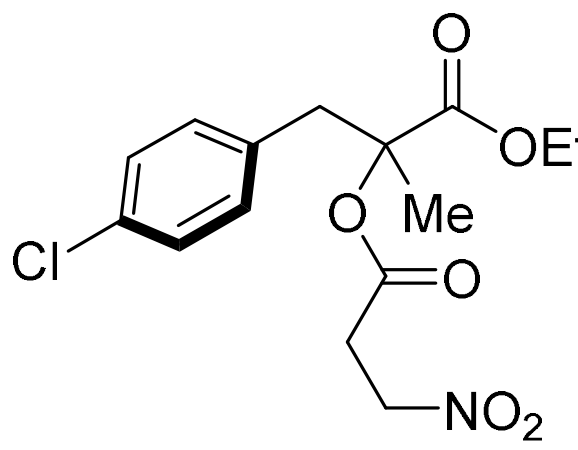

8y. Colorless oil (29.5 mg, 43% yield).

Analytical data for **8y**:

^{1}H NMR (600 MHz, $CDCl_3$) δ 7.30 – 7.27 (m, 2H), 7.11 – 7.07 (m, 2H), 4.67 – 4.58 (m, 2H), 4.15 (q, J = 7.1 Hz, 2H), 3.27 (d, J = 14.0 Hz, 1H), 3.05 (d, J = 14.1 Hz, 1H), 3.04 – 2.94 (m, 2H), 1.52 (s, 3H), 1.21 (t, J = 7.1 Hz, 3H).

^{13}C NMR (151 MHz, $CDCl_3$) δ 171.1, 168.3, 133.3, 133.1, 131.8, 128.4, 81.8, 69.4, 61.7, 42.7, 31.2, 21.3, 13.9.

IR (neat) ν_{max} (cm^{-1}) = 2984, 2953, 2859, 1766, 1667, 1543, 1478, 1361, 1244, 1184, 1123, 1048, 1037, 920, 857, 781, 742, 683.

HRMS (ESI/QTOF) m/z: $[M + Na]^+$ Calcd for $C_{15}H_{18}ClNNaO_6^+$ 366.0715; Found 366.0721.

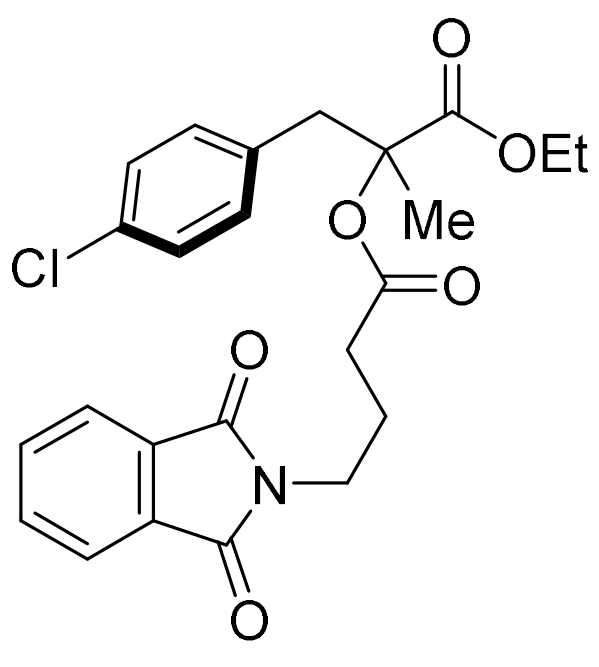

8z. Colorless oil (36.5 mg, 40% yield).

Analytical data for **8z**:

^{1}H NMR (600 MHz, $CDCl_3$) δ 7.85 (dd, *J* = 5.4, 3.0 Hz, 2H), 7.72 (dd, *J* = 5.5, 3.0 Hz, 2H), 7.26 – 7.23 (m, 2H), 7.11 – 7.07 (m, 2H), 4.14 (q, *J* = 7.1 Hz, 2H), 3.74 (t, *J* = 7.0 Hz, 2H), 3.23 (d, *J* = 13.9 Hz, 1H), 3.00 (d, *J* = 13.9 Hz, 1H), 2.38 (ddd, *J* = 8.1, 6.8, 1.5 Hz, 2H), 2.04 – 1.97 (m, 2H), 1.48 (s, 3H), 1.19 (t, *J* = 7.2 Hz, 3H).

^{13}C NMR (151 MHz, $CDCl_3$) δ 171.6, 171.4, 168.3, 134.0, 133.5, 133.0, 132.0, 131.8, 128.3, 123.3, 80.6, 61.4, 42.9, 37.1, 31.8, 23.9, 21.2, 14.0.

IR (neat) ν_{max} (cm^{-1}) = 2947, 2893, 1754, 1721, 1689, 1666, 1567, 1440, 1382, 1285, 1187, 1063, 982, 943, 898, 829, 786, 704, 665.

HRMS (nanochip-ESI/LTQ-Orbitrap) m/z: $[M + Na]^+$ Calcd for $C_{24}H_{24}ClNNaO_6^+$ 480.1184; Found 480.1204.

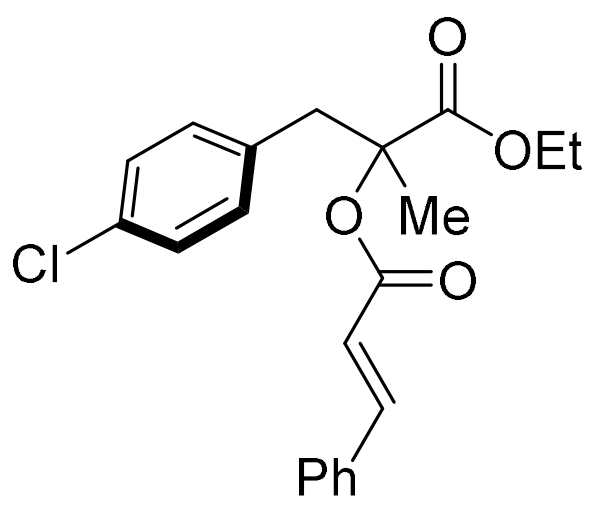

8aa. Colorless oil (36.5 mg, 49% yield).

Analytical data for **8aa**:

^{1}H NMR (400 MHz, $CDCl_3$) δ 7.68 (d, *J* = 16.0 Hz, 1H), 7.56 – 7.50 (m, 2H), 7.42 – 7.37 (m, 3H), 7.31 – 7.25 (m, 2H), 7.18 – 7.12 (m, 2H), 6.43 (d, *J* = 16.0 Hz, 1H), 4.20 (q, *J* = 7.1 Hz, 2H), 3.39 (d, *J* = 13.9 Hz, 1H), 3.10 (d, *J* = 14.0 Hz, 1H), 1.55 (s, 3H), 1.23 (t, *J* = 7.1 Hz, 3H).

^{13}C NMR (101 MHz, $CDCl_3$) δ 171.8, 165.7, 145.7, 134.2, 133.7, 133.0, 131.9, 130.5, 128.9, 128.3, 128.2, 117.6, 80.4, 61.5, 42.5, 21.4, 14.0.

IR (neat) ν_{max} (cm^{-1}) = 2987, 2855, 1724, 1653, 1542, 1517, 1481, 1325, 1270, 1164, 1067, 957, 877, 781, 754, 697.

HRMS (ESI/QTOF) m/z: $[M + Na]^+$ Calcd for $C_{21}H_{21}ClNaO_4^+$ 395.1021; Found 395.1028.

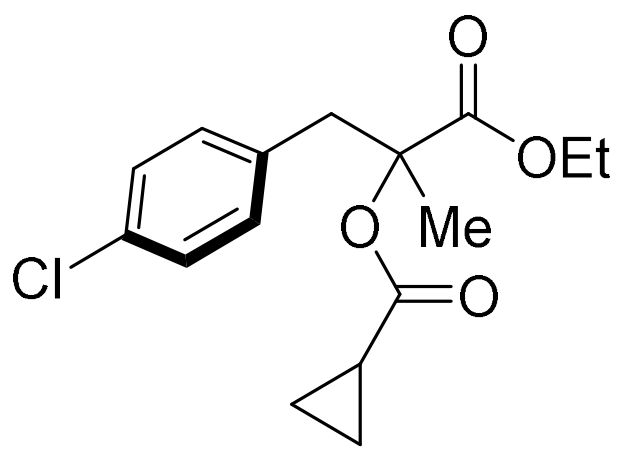

8ab. Colorless oil (41.6 mg, 67% yield).

Analytical data for **8ab**:

^{1}H NMR (600 MHz, $CDCl_3$) δ 7.29 – 7.26 (m, 2H), 7.13 – 7.09 (m, 2H), 4.15 (qd, *J* = 7.2, 2.1 Hz, 2H), 3.28 (d, *J* = 13.9 Hz, 1H), 3.00 (d, *J* = 13.9 Hz, 1H), 1.61 (tt, *J* = 8.1, 4.8 Hz, 1H), 1.47 (s, 3H), 1.21 (t, *J* = 7.1 Hz, 3H), 1.02 – 0.94 (m, 2H), 0.92 – 0.82 (m, 2H).

^{13}C NMR (151 MHz, $CDCl_3$) δ 173.6, 171.8, 133.7, 133.0, 131.9, 128.3, 80.1, 61.3, 42.6, 21.3, 14.0, 13.1, 8.6, 8.5.

IR (neat) ν_{max} (cm^{-1}) = 2981, 2874, 1721, 1662, 1523, 1443, 1372, 1263, 1173, 1079, 1014, 972, 843, 776, 742, 685.

HRMS (ESI/QTOF) m/z: $[M + Na]^+$ Calcd for $C_{16}H_{19}ClNaO_4^+$ 333.0864; Found 333.0863.

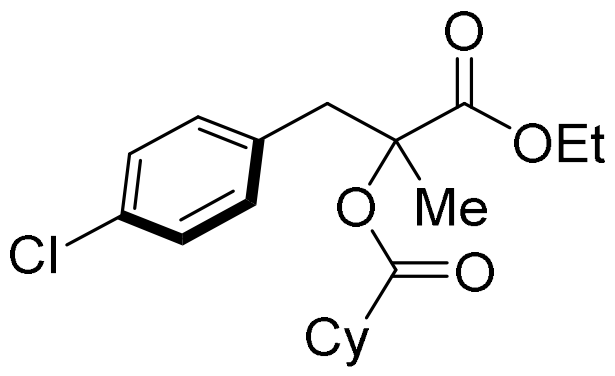

8ac. Colorless oil (42.2 mg, 60% yield).

Analytical data for **8ac**:

^{1}H NMR (500 MHz, $CDCl_3$) δ 7.28 – 7.24 (m, 2H), 7.12 – 7.08 (m, 2H), 4.14 (qd, *J* = 7.2, 1.5 Hz, 2H), 3.27 (d, *J* = 13.9 Hz, 1H), 3.00 (d, *J* = 13.9 Hz, 1H), 2.29 (tt, *J* = 11.1, 3.6 Hz, 1H), 1.91 – 1.83 (m, 2H), 1.78 – 1.70 (m, 2H), 1.66 – 1.61 (m, 1H), 1.47 (s, 3H), 1.46 – 1.38 (m, 2H), 1.33 – 1.23 (m, 3H), 1.20 (t, *J* = 7.1 Hz, 3H).

^{13}C NMR (126 MHz, $CDCl_3$) δ 174.8, 171.9, 133.7, 133.0, 131.9, 128.2, 79.9, 61.3, 43.1, 42.8, 28.81, 28.79, 25.7, 25.3, 21.3, 14.0.

IR (neat) ν_{max} (cm^{-1}) = 2987, 2862, 1754, 1667, 1546, 1448, 1354, 1263, 1233, 1164, 1102, 1076, 1022, 950, 881, 857, 823, 789, 691.

HRMS (ESI/QTOF) m/z: $[M + Na]^+$ Calcd for $C_{19}H_{25}ClNaO_4^+$ 375.1334; Found 375.1337.

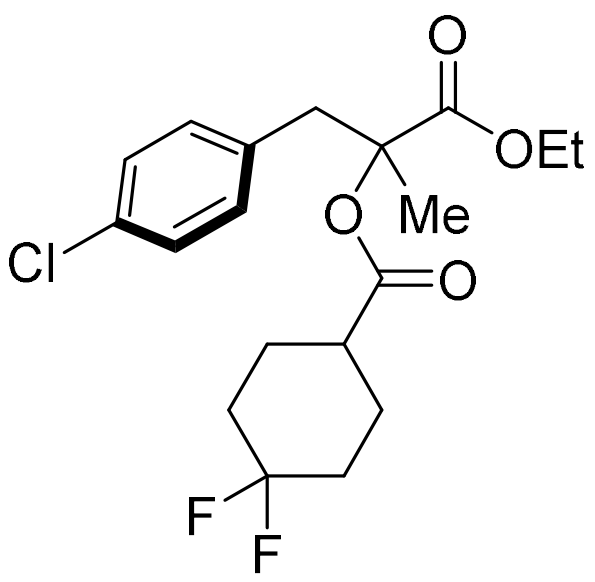

8ad. Colorless oil (48.1 mg, 62% yield).

Analytical data for **8ad**:

^{1}**H NMR** (600 MHz, $CDCl_3$) δ 7.27 (dd, *J* = 8.4, 1.9 Hz, 2H), 7.10 – 7.07 (m, 2H), 4.15 (qd, *J* = 7.1, 1.1 Hz, 2H), 3.26 (d, *J* = 14.0 Hz, 1H), 3.02 (d, *J* = 14.0 Hz, 1H), 2.45 – 2.40 (m, 1H), 2.13 – 2.03 (m, 2H), 1.97 – 1.90 (m, 2H), 1.90 – 1.72 (m, 4H), 1.51 (s, 3H), 1.20 (t, *J* = 7.1 Hz, 3H).

^{13}**C NMR** (151 MHz, $CDCl_3$) δ 173.0, 171.5, 133.4, 133.2, 131.8, 128.3, 122.6 (t, *J* = 241.1 Hz), 80.6, 61.5, 42.9, 40.4, 32.3 (td, *J* = 24.6, 2.6 Hz), 24.9 (dt, *J* = 10.2, 5.1 Hz), 21.4, 14.0.

^{19}**F{^{1}H} NMR** (377 MHz, $CDCl_3$) δ -95.11 (d, *J* = 237.8 Hz), -98.66 (d, *J* = 235.3 Hz).

^{19}**F NMR** (377 MHz, $CDCl_3$) δ -95.11 (brd, *J* = 237.6 Hz, 1F), -98.66 (brd, *J* = 239.6 Hz, 1F).

IR (neat) ν_{max} (cm^{-1}) = 2987, 2951, 2879, 1732, 1663, 1594, 1449, 1372, 1274, 1263, 1162, 1076, 943, 832, 774, 696.

HRMS (nanochip-ESI/LTQ-Orbitrap) m/z: $[M + Na]^+$ Calcd for $C_{19}H_{23}ClF_2NaO_4^+$ 411.1145; Found 411.1161.

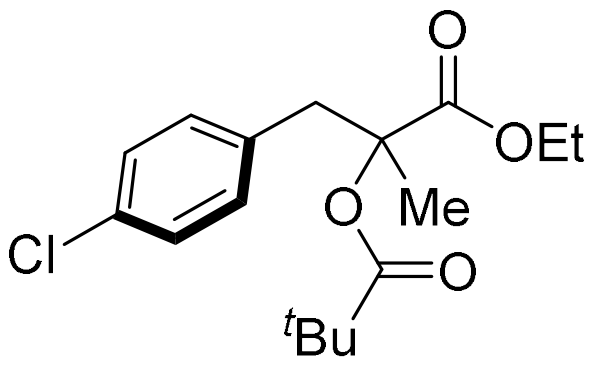

8ae. Colorless oil (35.2 mg, 54% yield).

Analytical data for **8ae**:

^{1}**H NMR** (600 MHz, $CDCl_3$) δ 7.28 – 7.25 (m, 2H), 7.13 – 7.09 (m, 2H), 4.18 – 4.09 (m, 2H), 3.26 (d, *J* = 13.9 Hz, 1H), 2.99 (d, *J* = 13.9 Hz, 1H), 1.48 (s, 3H), 1.19 (t, *J* = 7.1 Hz, 3H), 1.18 (s, 9H).

^{13}**C NMR** (151 MHz, $CDCl_3$) δ 177.2, 171.9, 133.7, 133.0, 131.9, 128.1, 80.0, 61.2, 43.0, 38.7, 27.0, 21.2, 14.0.

IR (neat) ν_{max} (cm^{-1}) = 2998, 2965, 2847, 1753, 1668, 1553, 1494, 1461, 1384, 1281, 1166, 1073, 1028, 847, 747, 695.

HRMS (nanochip-ESI/LTQ-Orbitrap) m/z: $[M + H]^+$ Calcd for $C_{17}H_{24}ClO_4^+$ 327.1358; Found 327.1372.

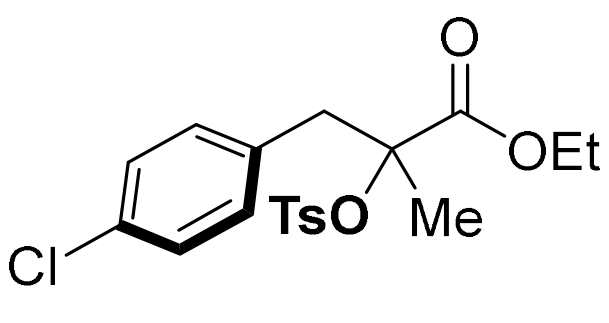

8af. Colorless oil (38.8 mg, 49% yield).

Analytical data for **8af**:

^{1}**H NMR** (500 MHz, $CDCl_3$) δ 7.79 – 7.74 (m, 2H), 7.32 – 7.28 (m, 2H), 7.25 – 7.21 (m, 2H), 7.11 – 7.05 (m, 2H), 4.21 (q, *J* = 7.2 Hz, 2H), 3.16 (d, *J* = 13.9 Hz, 1H), 3.08 (d, *J* = 13.9 Hz, 1H), 2.43 (s, 3H), 1.69 (s, 3H), 1.25 (t, *J* = 7.1 Hz, 3H).

^{13}**C NMR** (126 MHz, $CDCl_3$) δ 170.5, 144.4, 135.7, 133.4, 132.4, 131.8, 129.5, 128.4, 127.3, 88.0, 62.1, 45.0, 21.9, 21.6, 13.9.

IR (neat) ν_{max} (cm^{-1}) = 2974, 1734, 1653, 1543, 1470, 1345, 1251, 1162, 1076, 1031, 955, 871, 762, 696, 621.

HRMS (nanochip-ESI/LTQ-Orbitrap) m/z: $[M + Na]^+$ Calcd for $C_{19}H_{21}ClNaO_5S^+$ 419.0690; Found 419.0685.

General procedure for hypervalent iodine based dyotropic rearrangement reactions: migrative alkoxylation

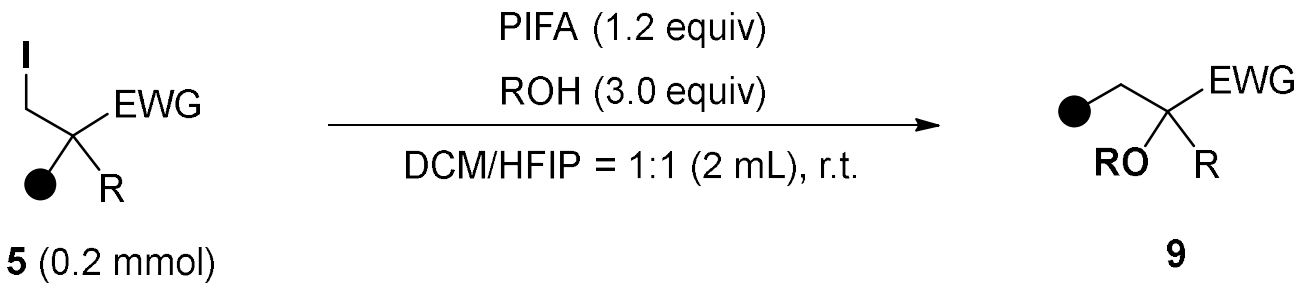

At room temperature, to a 10-mL test tube charged with a magnetic stirring bar were added substrates **5** (0.2 mmol, 1.0 equiv), PIFA (103.2 mg, 0.24 mmol, 1.2 equiv) and ROH (0.6 mmol, 3.0 equiv) in anhydrous DCM (1.0 mL) and HFIP (1.0 mL). The reaction mixture was allowed to stir at room temperature. After the reaction was complete (monitored by TLC), the mixture was quenched with H_2O. Then, the mixture was extracted twice with DCM. The combined organic extracts were dried over Na_2SO_4 and concentrated under reduced pressure. The residue was purified by silica gel flash column chromatography to give the desired products **9**

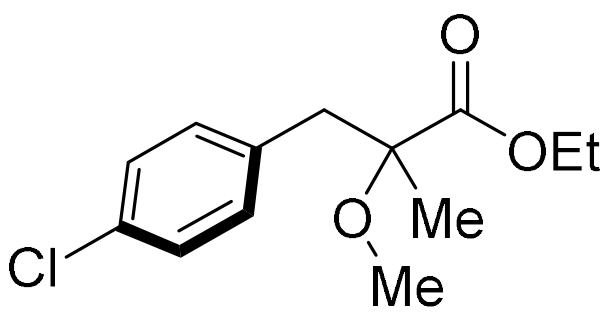

9a. Colorless oil (26.6 mg, 52% yield).

Analytical data for **9a**:

^{1}H NMR (500 MHz, $CDCl_3$) δ 7.25 – 7.21 (m, 2H), 7.15 – 7.11 (m, 2H), 4.17 (q, *J* = 7.1 Hz, 2H), 3.30 (s, 3H), 3.01 (d, *J* = 13.8 Hz, 1H), 2.95 (d, *J* = 13.8 Hz, 1H), 1.33 (s, 3H), 1.25 (t, *J* = 7.1 Hz, 3H).

^{13}C NMR (126 MHz, $CDCl_3$) δ 173.6, 134.6, 132.6, 131.7, 128.1, 80.6, 61.1, 52.1, 44.0, 19.9, 14.2.

IR (neat) ν_{max} (cm^{-1}) = 2981, 2868, 1772, 1654, 1543, 1433, 1321, 1316, 1279, 1147, 1125, 1066, 951, 887, 791, 697, 657.

HRMS (ESI/QTOF) m/z: $[M + Na]^+$ Calcd for $C_{13}H_{17}ClNaO_3^+$ 279.0758; Found 279.0767.

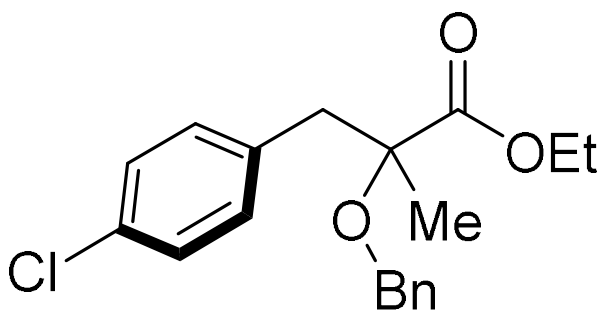

9b. Colorless oil (38.5 mg, 58% yield).

Analytical data for **9b**:

^{1}H NMR (400 MHz, $CDCl_3$) δ 7.37-7.25 (m, 5H), 7.25 – 7.21 (m, 2H), 7.19 – 7.15 (m, 2H), 4.52 (d, *J* = 11.0 Hz, 1H), 4.48 (d, *J* = 11.0 Hz, 1H), 4.16 (qd, *J* = 7.1, 1.8 Hz, 2H), 3.10 (d, *J* = 13.6 Hz, 1H), 3.03 (d, *J* = 13.7 Hz, 1H), 1.44 (s, 3H), 1.24 (t, *J* = 7.1 Hz, 3H).

^{13}C NMR (101 MHz, $CDCl_3$) δ 173.7, 138.5, 134.7, 132.6, 131.9, 128.3, 128.0, 127.44, 127.43, 80.8, 66.9, 61.1, 44.8, 20.6, 14.2.

IR (neat) ν_{max} (cm^{-1}) = 2865, 1721, 1654, 1532, 1491, 1489, 1333, 1311, 1269, 1172, 1143, 1036, 981, 857, 780, 715, 696, 643.

HRMS (APCI/QTOF) m/z: $[M + Na]^+$ Calcd for $C_{19}H_{21}ClNaO_3^+$ 355.1071; Found 355.1076.

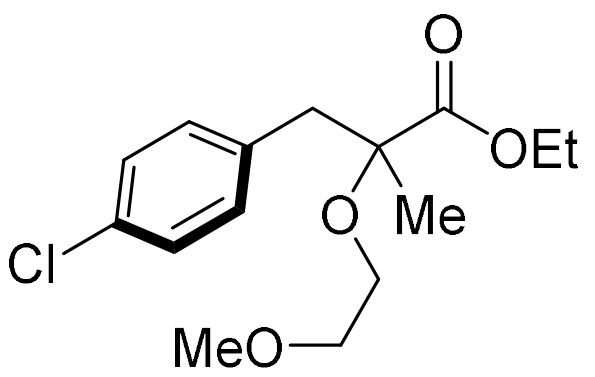

9c. Colorless oil (36.0 mg, 60% yield).

Analytical data for **9c**:

^{1}H NMR (600 MHz, $CDCl_3$) δ 7.25 – 7.20 (m, 2H), 7.17 – 7.13 (m, 2H), 4.16-4.11 (m, 2H), 3.61 – 3.53 (m, 4H), 3.39 (s, 3H), 3.03 (d, *J* = 13.7 Hz, 1H), 2.99 (d, *J* = 13.7 Hz, 1H), 1.34 (s, 3H), 1.23 (t, *J* = 7.1 Hz, 3H).

^{13}C NMR (151 MHz, $CDCl_3$) δ 173.5, 134.7, 132.6, 131.8, 128.0, 80.5, 72.0, 63.9, 61.0, 59.1, 44.4, 20.4, 14.2.

IR (neat) ν_{max} (cm^{-1}) = 2978, 2921, 2879, 1771, 1678, 1683, 1522, 1502, 1488, 1412, 1391, 1377, 1281, 1162, 1097, 929, 641.

HRMS (ESI/QTOF) m/z: $[M + Na]^+$ Calcd for $C_{15}H_{21}ClNaO_4^+$ 323.1021; Found 323.1025.

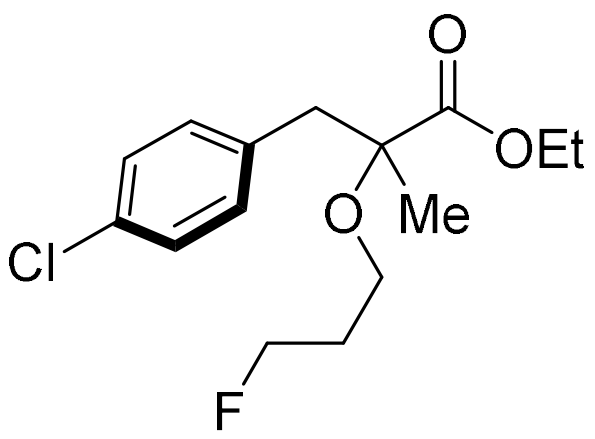

9d. Colorless oil (32.0 mg, 53% yield).

Analytical data for **9d**:

^{1}H NMR (600 MHz, $CDCl_3$) δ 7.24 – 7.21 (m, 2H), 7.14 – 7.10 (m, 2H), 4.62 – 4.55 (m, 1H), 4.54 – 4.47 (m, 1H), 4.16-4.11 (m, 2H), 3.54 (dt, *J* = 8.8, 5.9 Hz, 1H), 3.49 (dt, *J* = 8.8, 6.0 Hz, 1H), 3.00 (d, *J* = 13.7 Hz, 1H), 2.93 (d, *J* = 13.7 Hz, 1H), 1.99 – 1.90 (m, 2H), 1.34 (s, 3H), 1.23 (t, *J* = 7.1 Hz, 3H).

^{13}C NMR (151 MHz, $CDCl_3$) δ 173.7, 134.7, 132.6, 131.8, 128.0, 81.1 (d, *J* = 163.3 Hz), 80.3, 61.0, 60.0 (d,

J = 5.4 Hz), 44.7, 31.2 (d, J = 19.9 Hz), 20.3, 14.1.

^{19}F{^{1}H} NMR (377 MHz, $CDCl_3$) δ -221.59.

^{19}F NMR (377 MHz, $CDCl_3$) δ -221.59 (tt, J = 47.2, 25.9 Hz).

IR (neat) ν_{max} (cm^{-1}) = 2982, 2911, 2863, 1771, 1654, 1533, 1517, 1471, 1340, 1322, 1276, 1152, 1088, 1038, 912, 828, 738, 651.

HRMS (ESI/QTOF) m/z: $[M + Na]^+$ Calcd for $C_{15}H_{20}ClFNaO_3^+$ 325.0977; Found 325.0984.

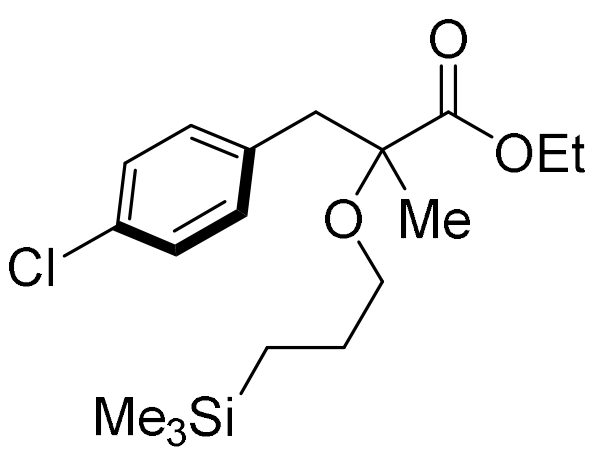

9e. Colorless oil (28.5 mg, 40% yield).

Analytical data for **9e**:

^{1}H NMR (600 MHz, $CDCl_3$) δ 7.24 – 7.21 (m, 2H), 7.16 – 7.13 (m, 2H), 4.18-4.12 (m, 2H), 3.33 (td, J = 7.0, 1.8 Hz, 2H), 3.01 (d, J = 13.7 Hz, 1H), 2.95 (d, J = 13.7 Hz, 1H), 1.60-1.54 (m, 2H), 1.31 (s, 3H), 1.24 (t, J = 7.1 Hz, 3H), 0.49 – 0.44 (m, 2H), -0.01 (s, 9H).

^{13}C NMR (151 MHz, $CDCl_3$) δ 174.0, 134.8, 132.5, 131.9, 128.0, 80.2, 67.5, 60.9, 44.5, 24.6, 20.3, 14.2, 12.6, -1.8.

IR (neat) ν_{max} (cm^{-1}) = 2984, 2912, 2879, 1711, 1651, 1533, 1434, 1421, 1372, 1266, 1241, 1191, 1152, 1079, 1044, 964, 873, 797, 697.

HRMS (nanochip-ESI/LTQ-Orbitrap) m/z: $[M + Na]^+$ Calcd for $C_{18}H_{29}ClNaO_3Si^+$ 379.1467; Found 379.1481.

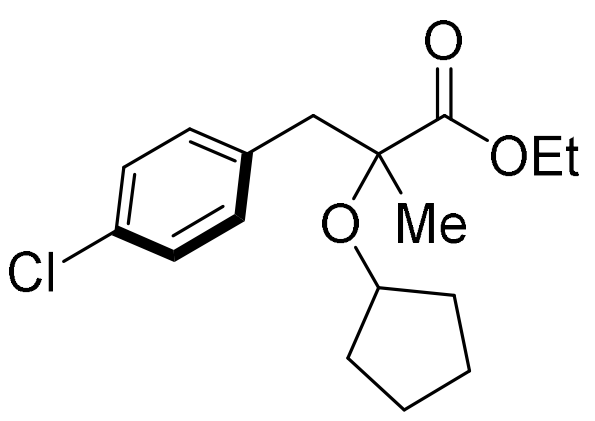

9f. Colorless oil (27.3 mg, 44% yield).

Analytical data for **9f**:

^{1}H NMR (600 MHz, $CDCl_3$) δ 7.23 – 7.20 (m, 2H), 7.16 – 7.12 (m, 2H), 4.18 – 4.07 (m, 2H), 3.93-3.90 (m, 1H), 2.95 (d, J = 13.5 Hz, 1H), 2.89 (d, J = 13.5 Hz, 1H), 1.82 – 1.74 (m, 1H), 1.73 – 1.65 (m, 4H), 1.49-1.42 (m, 3H), 1.31 (s, 3H), 1.23 (t, J = 7.1 Hz, 3H).

^{13}C NMR (151 MHz, $CDCl_3$) δ 174.6, 135.0, 132.4, 131.9, 127.9, 80.6, 77.4, 60.8, 45.5, 34.6, 33.9, 23.7, 23.5,

21.2, 14.1.

IR (neat) ν_{max} (cm^{-1}) = 2978, 2923, 1721, 1654, 1497, 1488, 1371, 1321, 1291, 1222, 1134, 1090, 1032, 952, 874, 783, 721, 691, 622.

HRMS (nanochip-ESI/LTQ-Orbitrap) m/z: $[M + Na]^+$ Calcd for $C_{17}H_{23}ClNaO_3^+$ 333.1228; Found 333.1240.

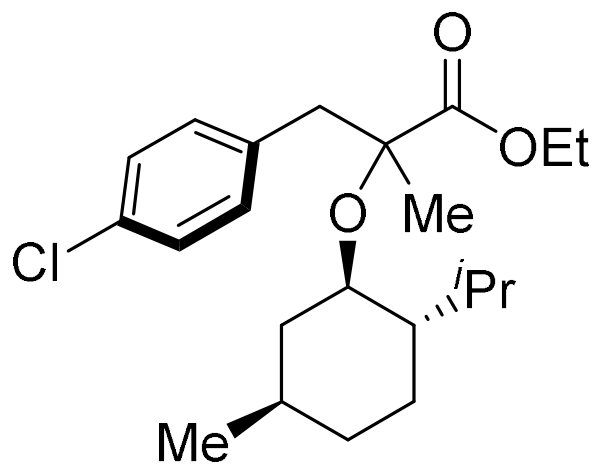

9g. Colorless oil (31.9 mg, 42% yield, dr = 1:1).

Analytical data for **9g**:

^{1}H NMR (600 MHz, $CDCl_3$) δ 7.23 – 7.19 (m, 4H), 7.16 – 7.13 (m, 2H), 7.13 – 7.10 (m, 2H), 4.19 – 4.07 (m, 4H), 3.40 (td, *J* = 10.3, 4.2 Hz, 1H), 3.34 (td, *J* = 10.5, 4.2 Hz, 1H), 3.10 (d, *J* = 13.3 Hz, 1H), 2.97 (d, *J* = 13.3 Hz, 1H), 2.93 (d, *J* = 13.3 Hz, 1H), 2.925 (d, *J* = 13.3 Hz, 1H), 2.30 (dtd, *J* = 14.2, 7.1, 2.4 Hz, 1H), 2.15 (pd, *J* = 7.0, 2.7 Hz, 1H), 1.85 (dtd, *J* = 12.5, 3.8, 1.8 Hz, 1H), 1.71 (dtd, *J* = 12.2, 3.9, 1.6 Hz, 1H), 1.64 – 1.59 (m, 4H), 1.34 (s, 3H), 1.31 (s, 3H), 1.29 – 1.25 (m, 8H), 1.21 – 1.17 (m, 1H), 0.99 – 0.88 (m, 5H), 0.90 (d, *J* = 7.1 Hz, 3H), 0.86 (d, *J* = 7.5 Hz, 3H), 0.852 (d, *J* = 6.5 Hz, 3H), 0.85 (d, *J* = 7.0 Hz, 3H), 0.83 – 0.77 (m, 2H), 0.68 (d, *J* = 6.9 Hz, 3H), 0.56 (d, *J* = 6.9 Hz, 3H).

^{13}C NMR (151 MHz, $CDCl_3$) δ 175.3, 173.0, 135.3, 134.6, 132.6, 132.4, 132.0, 127.99, 127.95, 80.1, 78.1, 74.5, 73.7, 61.0, 60.9, 49.0, 48.0, 46.6, 45.4, 44.5, 41.7, 34.3, 34.2, 31.6, 31.5, 24.8, 24.7, 23.1, 22.9, 22.4, 22.3, 22.0, 21.6, 21.0, 18.8, 16.2, 15.5, 14.1.

IR (neat) ν_{max} (cm^{-1}) = 2962, 2855, 1741, 1654, 1622, 1516, 1477, 1439, 1361, 1320, 1212, 1171, 1165, 1013, 981, 837, 750, 693, 626.

HRMS (ESI/QTOF) m/z: $[M + Na]^+$ Calcd for $C_{22}H_{33}ClNaO_3^+$ 403.2010; Found 403.2014.

Mechanism Study

Control experiments

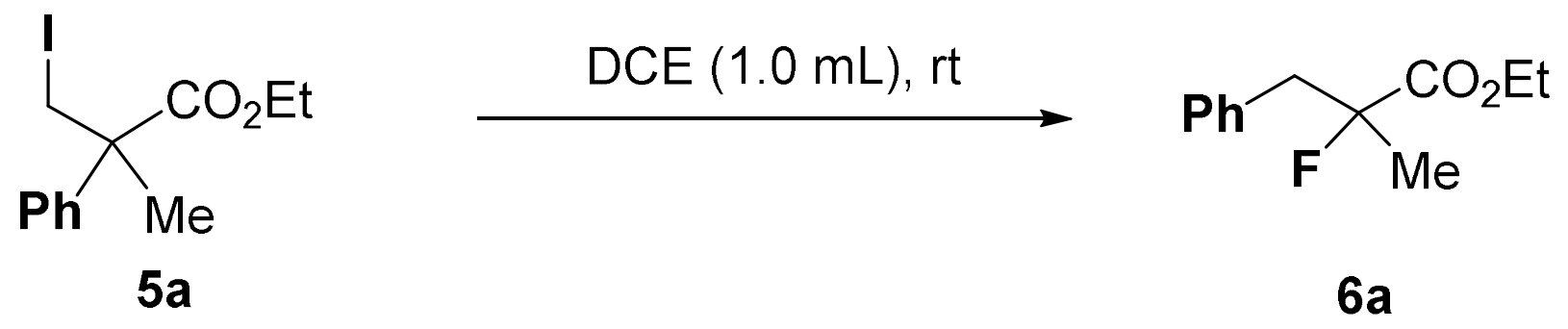

XeF_2 (1.2 equiv)	8 h, 60% conv., 40%
XeF_2 (1.2 equiv), Py•9HF (0.2 mL)	1 min, >95% conv., 75%
Selectfluor (1.2 equiv), Py•9HF (0.2 mL)	4 h, >95% conv., 74%
Py•9HF (0.2 mL)	24 h, <10% conv., <5%
Et_4NF (5.0 equiv), Py•9HF (0.2 mL)	24 h, <10% conv., <5%
Et_4NF (5.0 equiv)	24 h, <10% conv., <5%
Selectfluor (1.2 equiv)	30 h, 50% conv., 10%

Trapping the in situ generated I^+ species

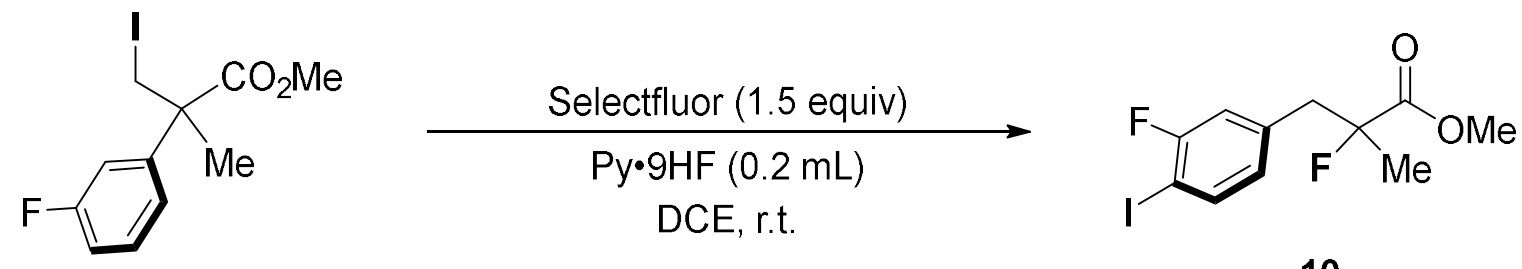

5i 10

At room temperature, to a 10-mL test tube charged with a magnetic stirring bar, were added substrate **5i** (64.4 mg, 0.2 mmol, 1.0 equiv), Selectfluor (106 mg, 0.3 mmol, 1.5 equiv) and Py·9HF (0.2 mL) in anhydrous DCE (2.0 mL). The reaction mixture was allowed to stir at room temperature. After the reaction was complete (monitored by TLC), the mixture was quenched with $NaHCO_3$ solution (2 M) and $Na_2S_2O_3$ solution (2 M). Then, the mixture was extracted twice with ethyl acetate. The combined organic extracts were dried over Na_2SO_4 and concentrated under reduced pressure. The residue was purified by silica gel flash column chromatography to give the desired product **10**.

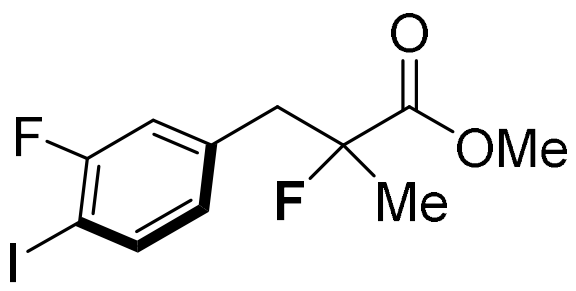

10. Colorless oil (34.0 mg, 50% yield).

Analytical data for **10**:

^{1}H NMR (500 MHz, $CDCl_3$) δ 7.66 (t, J = 7.3 Hz, 1H), 6.94 (dd, J = 8.9, 1.8 Hz, 1H), 6.76 (dd, J = 7.9, 1.8 Hz, 1H), 3.73 (s, 3H), 3.19 (dd, J = 26.8, 14.4 Hz, 1H), 3.04 (dd, J = 20.0, 14.4 Hz, 1H), 1.58 (d, J = 21.4 Hz, 3H).

^{13}C NMR (126 MHz, $CDCl_3$) δ 171.6 (d, J = 24.9 Hz), 161.5 (d, J = 245.6 Hz), 139.1 (d, J = 2.1 Hz), 137.3 (d, J = 7.1 Hz), 127.6 (d, J = 3.2 Hz), 117.5 (d, J = 23.6 Hz), 94.6 (d, J = 188.8 Hz), 79.8 (d, J = 25.5 Hz), 52.6, 43.4 (dd, J = 21.5, 1.8 Hz), 23.4 (d, J = 23.9 Hz).

^{19}F{^{1}H} NMR (377 MHz, $CDCl_3$) δ -94.05, -155.59.

^{19}F NMR (377 MHz, $CDCl_3$) δ -94.02 – -94.07 (m), -155.58 (dp, J = 27.0, 20.9 Hz).

IR (neat) ν_{max} (cm^{-1}) = 2989, 2947, 2870, 1723, 1495, 1448, 1431, 1252, 1226, 1173, 1149, 1090, 1032, 1016, 996, 781, 756, 737, 725, 700, 654, 609.

HRMS (ESI/QTOF) m/z: $[M + Na]^+$ Calcd for $C_{11}H_{11}F_2INaO_2^+$ 362.9664; Found 362.9668.

Reaction involving phenonium intermediate

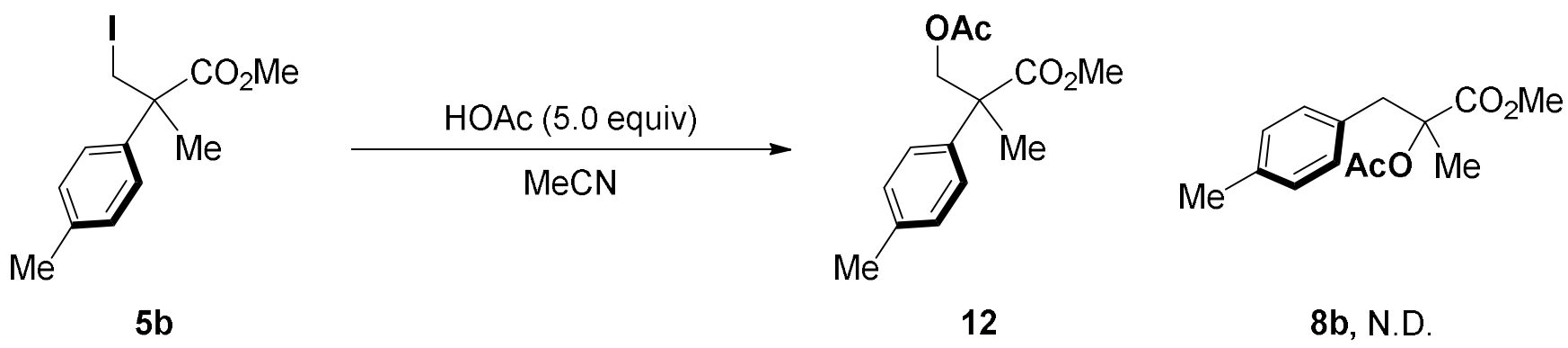

To a solution of **5b** (31.8 mg, 0.1 mmol) in MeCN (0.1 M) at room temperature was added HOAc (29 µL, 5.0 equiv) dropwise. The reaction mixture was allowed to warm to reflux. After the reaction was complete (monitored by TLC), the mixture was quenched with $NaHCO_3$ solution (2 M). Then, the mixture was extracted twice with ethyl acetate. The combined organic extracts were dried over Na_2SO_4 and concentrated under reduced pressure. The residue was purified by silica gel flash column chromatography to give the desired product **12**.

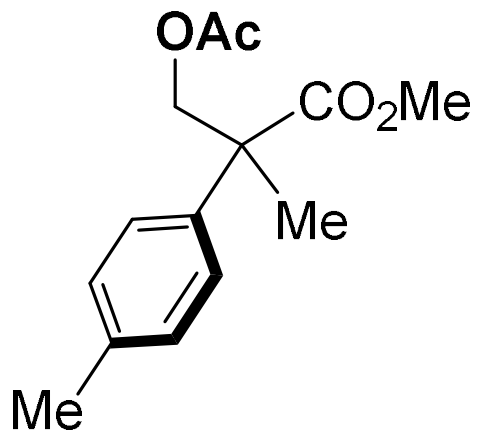

12. Colorless oil (10.0 mg, 40% yield).

Analytical data for **12**:

^{1}H NMR (400 MHz, $CDCl_3$) δ 7.23 – 7.18 (m, 2H), 7.15 (d, J = 8.3 Hz, 2H), 4.58 (d, J = 10.8 Hz, 1H), 4.35 (d, J = 10.8 Hz, 1H), 3.70 (s, 3H), 2.33 (s, 3H), 2.04 (s, 3H), 1.63 (s, 3H).

^{13}C NMR (101 MHz, $CDCl_3$) δ 174.5, 170.8, 137.2, 136.9, 129.3, 125.8, 69.2, 52.3, 50.0, 20.9, 20.8, 20.8.

IR (neat) ν_{max} (cm^{-1}) = 2983, 2922, 2913, 2844, 1756, 1733, 1655, 1537, 1502, 1487, 1455, 1336, 1277, 1261, 1144, 1102, 1077, 988, 883, 774, 722, 712, 633, 612.

HRMS (ESI/QTOF) m/z: [M + Na]$^+$ Calcd for $C_{14}H_{18}NaO_4^+$ 273.1097; Found 273.1105.

Study of stereochemistry

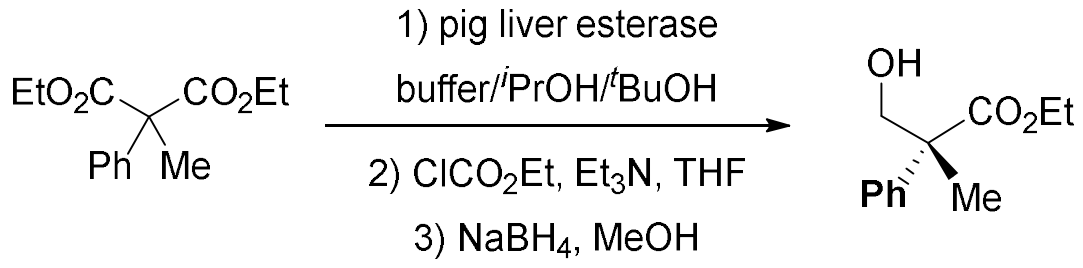

rac-**S16** (*R*)-**S17**, 74%

Step 1: The ester *rac*-**16** (1.25 g, 5.0 mmol) was suspended in mixed solvents (0.2 M, containing 20 mL phosphate buffer (pH = 7.0), 2.5 mL ^{i}PrOH and 2.5 mL ^{t}BuOH). Then, pig liver esterase was added and the reaction mixture was stirred at room temperature. The pH was maintained at 7.0 by addition of 0.2 M NaOH solution. The reaction was monitored by TLC. After full conversion of the starting material, the suspension became a homogeneous solution. The resulting mixture was washed with diethyl ether at pH 7.0 then acidified to pH 2.0 with 6.0 N HCl solution. The acidified solution was extracted with diethyl ether. The combined organic extracts were dried over Na_2SO_4 and concentrated under reduced pressure to give the product which was used for the next step without further purification.[4]

Step 2: To a solution of the chiral acid and Et_3N (1.1 mL, 7.5 mmol, 1.5 equiv) in THF (1 M) at 0 ºC was added $ClCO_2Et$ (0.57 mL, 6.0 mmol, 1.2 equiv) slowly. The reaction mixture was allowed to stir at room temperature. After the reaction was complete (monitored by TLC), diethyl ether (2 mL) was added and the reaction mixture was filtered through a short pad of silica gel with diethyl ether. The filtrate was concentrated under reduced pressure to give the product which was used for the next step without further purification.

Step 3: To a solution of the above product in MeOH (1 M) at 0 ºC was added $NaBH_4$ (246 mg, 6.5 mmol, 1.3 equiv) slowly. The reaction mixture was allowed to stir at room temperature. After the reaction was complete (monitored by TLC), the mixture was quenched with dilute HCl (2 M) and extracted with ethyl acetate (twice). The combined organic extracts were dried over Na_2SO_4 and concentrated under reduced pressure. Flash chromatography on silica gel (hexanes:ethyl acetate 3:1) afforded (*R*)-**S17**.

The absolute configuration was determined by comparing the specific optical rotation value with that reported in the literature.[5]

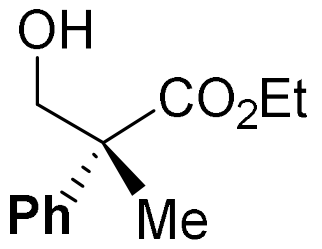

(*R*)-**S17**. Colorless oil (770 mg, 74% yield).

Analytical data for (*R*)-**S17**:

$[\alpha]_D^{25}$ = +20.5 (c = 1.0 $CHCl_3$).

^{1}H NMR (400 MHz, $CDCl_3$) δ 7.30 – 7.23 (m, 2H), 7.23 – 7.15 (m, 3H), 4.17 – 4.05 (m, 2H), 3.97 (d, J = 11.4 Hz, 1H), 3.54 (d, J = 11.4 Hz, 1H), 2.43 (brs, 1H), 1.57 (s, 3H), 1.13 (t, J = 7.1 Hz, 3H).

^{13}C NMR (101 MHz, $CDCl_3$) δ 176.0, 140.5, 128.5, 127.2, 126.1, 69.6, 61.0, 52.5, 19.9, 13.9.

IR (neat) ν_{max} (cm^{-1}) = 2934, 1642, 1497, 1446, 1361, 1173, 1069, 992, 766, 733, 699, 636.

HRMS (nanochip-ESI/LTQ-Orbitrap) m/z: $[M + H]^+$ Calcd for $C_{12}H_{17}O_3^+$ 209.1172; Found 209.1179.

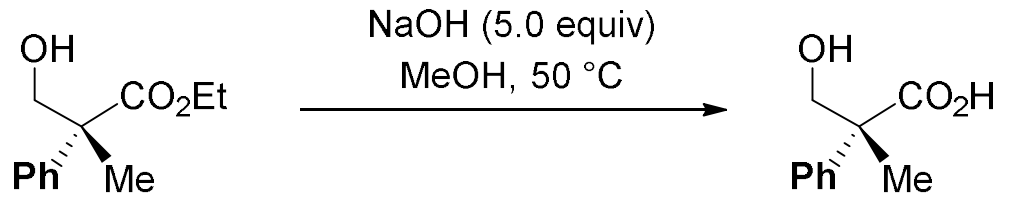

(*R*)-**S17** (*R*)-**S18**, 91%

To a solution of the ester (*R*)-**S17** (20.8 mg, 0.1 mmol, 1.0 equiv) in MeOH (1 mL) was added NaOH (20.0 mg, 5.0 equiv) and the reaction mixture was allowed to warm to 50 ºC. After the reaction was complete (monitored by TLC), the residue was diluted with water, acidified until pH = 1 with HCl (3 M), and extracted twice with ethyl acetate. The combined organic extracts were dried over Na_2SO_4 and concentrated under reduced pressure. Flash chromatography on silica gel (hexanes:ethyl acetate 3:1) afforded (*R*)-**S18** as colorless crystals. The absolute configuration was determined by X-ray single crystal analysis.

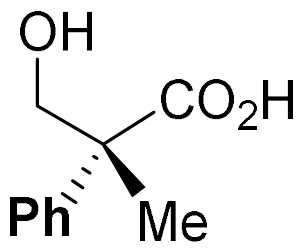

(*R*)-**S18**. Colorless solid (16.4 mg, 91% yield).

Analytical data for (*R*)-**S18**:

m.p. = 110 ~ 112 ºC.

$[\alpha]_D^{25}$ = +16.8 (c = 1.0 $CHCl_3$).

^{1}H NMR (400 MHz, $CDCl_3$) δ 7.41 – 7.37 (m, 4H), 7.34 – 7.28 (m, 1H), 4.12 (d, *J* = 11.5 Hz, 1H), 3.69 (d, *J* = 11.4 Hz, 1H), 1.70 (s, 3H).

^{13}C NMR (101 MHz, $CDCl_3$) δ 180.8, 139.5, 128.8, 127.7, 126.3, 69.1, 52.4, 20.1.

IR (neat) ν_{max} (cm^{-1}) = 2989, 2869, 1719, 1497, 1454, 1376, 1284, 1182, 1134, 1098, 1031, 946, 911, 868, 787, 761, 739, 698, 669, 664.

HRMS (nanochip-ESI/LTQ-Orbitrap) m/z: $[M + H]^+$ Calcd for $C_{10}H_{13}O_3^+$ 181.0859; Found 181.0863.

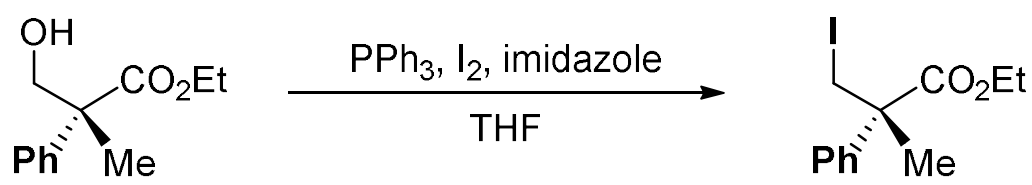

(*R*)-**S17** (*S*)-**5a**, 78%
96% ee

To a solution of triphenylphosphine (682 mg, 2.6 mmol, 1.3 equiv), imidazole (177 mg, 2.6 mmol 1.3 equiv) and iodine (660 mg, 2.6 mmol, 1.3 equiv) in THF (1 M) at 0 ºC was added (*R*)-**S17** (416 mg, 2.0 mmol, 1.0

M solution in THF) dropwise. The reaction mixture was allowed to warm to 60 ºC and stirred overnight. Then, the mixture was quenched with $Na_2S_2O_3$ solution (2 M) and extracted twice with ethyl acetate. The extracts were combined, dried over Na_2SO_4, filtered and concentrated under reduced pressure. The residue was purified by silica gel flash column chromatography (eluent: hexanes / ethyl acetate = 20:1 → 10:1) to give the desired product (*S*)-**5a**.

The ee of (*S*)-**5a** was determined to be 96%.

[α]$_D^{25}$ = +21.2 (*c* 1.0, $CHCl_3$).

HPLC: The enantiomeric excess was determined by Daicel Chiralpack IB (SFC:3.0 mm, I.D.:150 mm), CO_2 / MeOH = 100 / 0, 1.0 mL/min, λ = 220 nm, t (minor) = 2.19 min, t (major) = 2.38 min.

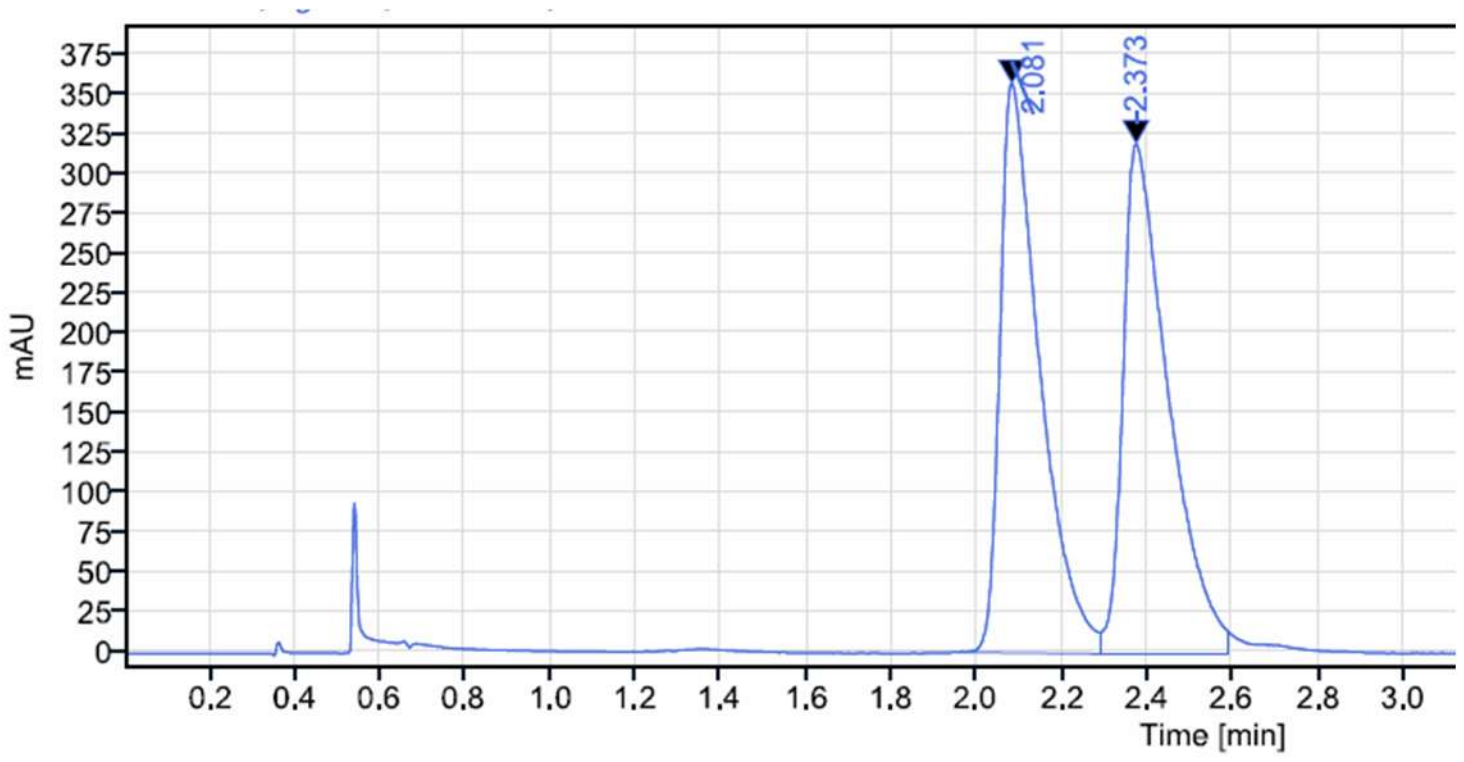

RT [min]	Type	Width [min]	Area	Height	Area%
2.081	MM m	0.35	2316.47	356.77	49.49
2.373	MM m	0.30	2363.90	319.44	50.51
		**Sum**	**4680.37**		

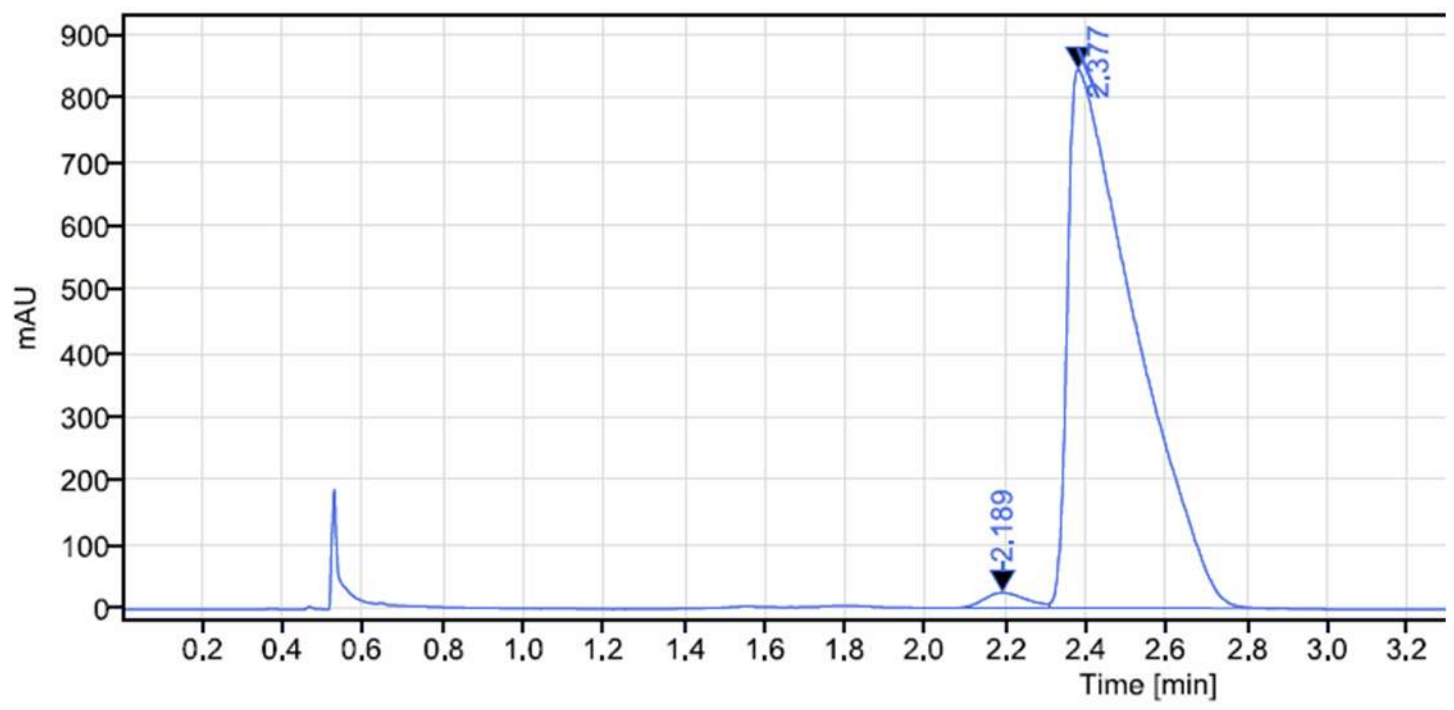

RT [min]	Type	Width [min]	Area	Height	Area%
2.189	MM m	0.25	178.75	24.16	1.79
2.377	MM m	0.57	9781.19	844.97	98.21
		**Sum**	**9959.93**		

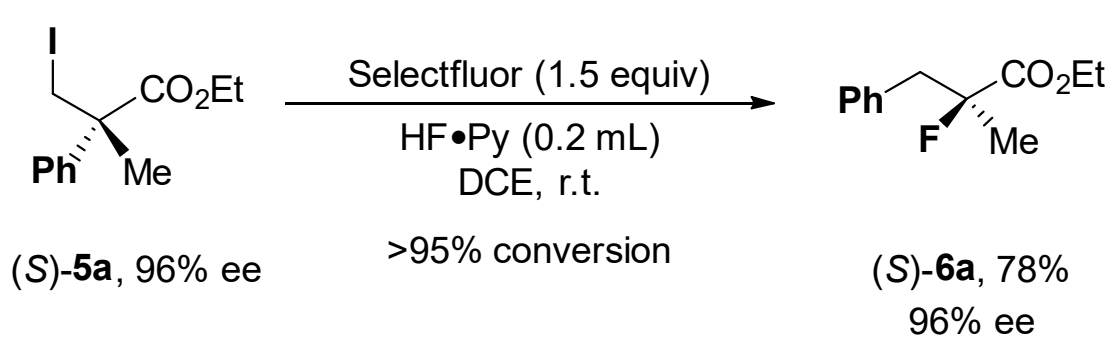

At room temperature, to a 10-mL test tube charged with a magnetic stirring bar, were added substrate (*S*)-**5a** (63.6 mg, 0.2 mmol, 1.0 equiv), Selectfluor (106 mg, 0.3 mmol, 1.5 equiv) and Py·9HF (0.2 mL) in anhydrous

DCE (2.0 mL). The reaction mixture was allowed to stir at room temperature. After the reaction was complete (monitored by TLC), the mixture was quenched with $NaHCO_3$ solution (2 M) and $Na_2S_2O_3$ solution (2 M). Then, the mixture was extracted twice with ethyl acetate. The combined organic extracts were dried over Na_2SO_4 and concentrated under reduced pressure. The residue was purified by silica gel flash column chromatography to give the desired product (*S*)-**6a**.

The absolute configuration was determined by X-ray single crystal analysis of the hydrolyzed product (*S*)-**6a**. The ee of (*S*)-**6a** was determined to be 96%.

$[\alpha]_D^{25}$ = -21.6 (*c* 1.0, $CHCl_3$).

HPLC: The enantiomeric excess was determined by Daicel Chiralpack IA (0.46 cm x 25 cm), Hexanes / IPA = 85 / 15, 1.0 mL/min, λ = 210 nm, t (major) = 8.28 min, t (minor) = 9.20 min.

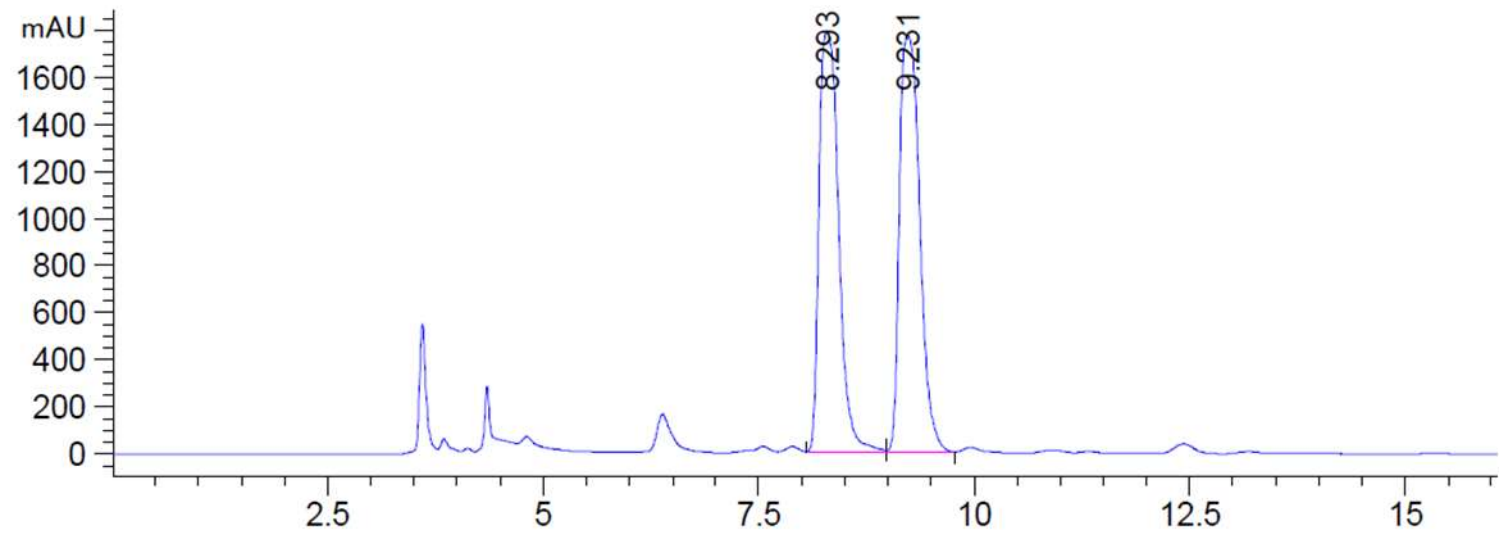

Peak #	RetTime [min]	Type	Width [min]	Area [mAU*s]	Height [mAU]	Area %
1	8.293	BV	0.2523	2.82371e4	1786.99902	49.4157
2	9.231	VB	0.2604	2.89048e4	1771.44861	50.5843

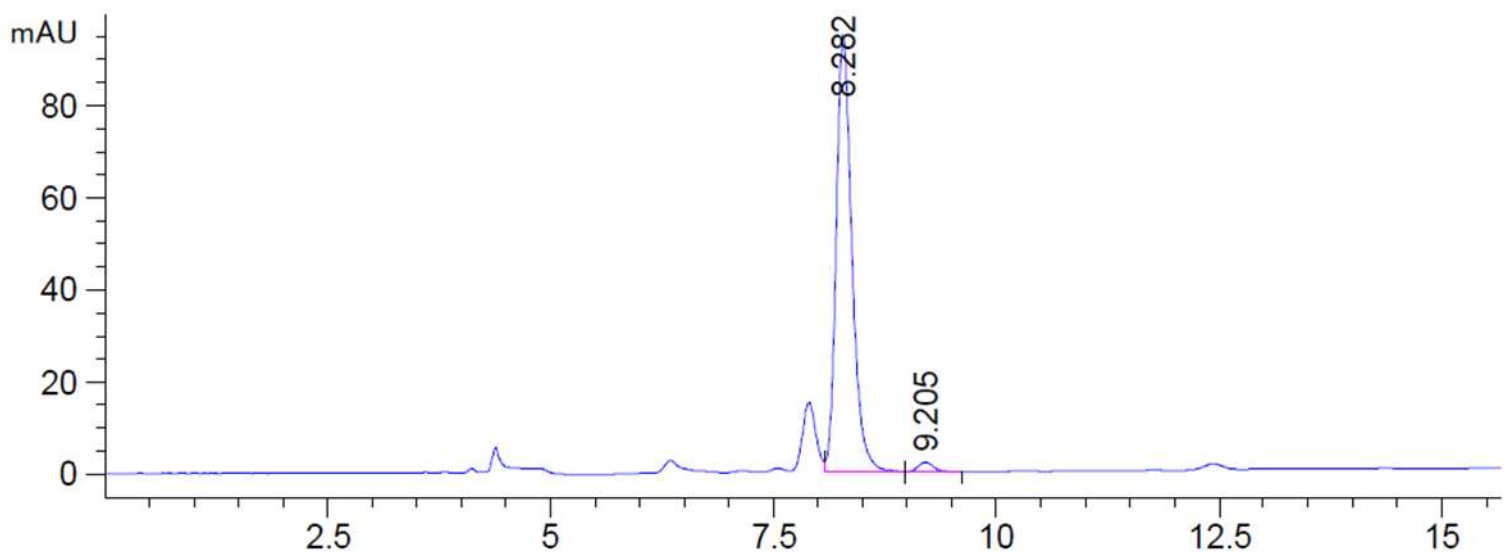

Peak #	RetTime [min]	Type	Width [min]	Area [mAU*s]	Height [mAU]	Area %
1	8.282	VB	0.1852	1162.36523	94.68412	97.8729
2	9.205	BB	0.1853	25.26245	2.11604	2.1271

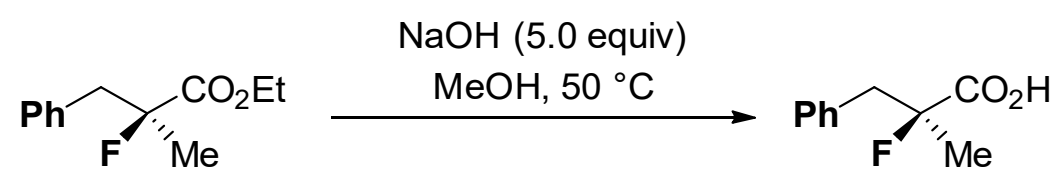

(*S*)-**6a** (*S*)-**15**, 90%

To a solution of the ester (*S*)-**6a** (21.0 mg, 0.1 mmol, 1.0 equiv) in MeOH (1 mL) was added NaOH (20.0 mg, 5.0 equiv) and the reaction mixture was allowed to warm to 50 ºC. After the reaction was complete (monitored by TLC), the residue was diluted with water, acidified until pH = 1 with HCl (3 M), and extracted twice with

ethyl acetate. The combined organic extracts were dried over Na_2SO_4 and concentrated under reduced pressure. Flash chromatography on silica gel (hexanes:ethyl acetate 3:1) afforded (*R*)-**14** as colorless crystals.
The absolute configuration was determined by X-ray single crystal analysis.

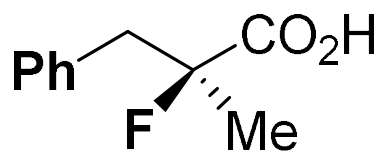

(*S*)-**15**. Colorless solid (16.4 mg, 90% yield).

Analytical data for (*S*)-**15**:

m.p. = 101 ~ 103 °C.

$[\alpha]_D^{25}$ = -9.5 (c = 1.0 $CHCl_3$).

^{1}H NMR (400 MHz, $CDCl_3$) δ 9.95 (s, 1H), 7.26 – 7.19 (m, 3H), 7.19 – 7.13 (m, 2H), 3.18 (dd, *J* = 26.0, 14.4 Hz, 1H), 3.03 (dd, *J* = 21.8, 14.4 Hz, 1H), 1.52 (d, *J* = 21.1 Hz, 3H).

^{13}C NMR (101 MHz, $CDCl_3$) δ 177.3 (d, *J* = 26.0 Hz), 134.0, 130.3, 128.4, 127.3, 94.5 (d, *J* = 187.5 Hz), 43.7 (d, *J* = 21.6 Hz), 23.0 (d, *J* = 24.0 Hz).

^{19}F{^{1}H} NMR (377 MHz, $CDCl_3$) δ -155.31.

^{19}F NMR (377 MHz, $CDCl_3$) δ -155.30 (dq, *J* = 25.5, 20.8 Hz).

IR (neat) ν_{max} (cm^{-1}) = 2989, 2869, 1729, 1497, 1454, 1377, 1287, 1181, 1134, 1097, 946, 912, 869, 784, 736, 699, 669, 664.

HRMS (LTQ-Orbitrap) m/z: $[M + H]^+$ Calcd for $C_{10}H_{12}FO_2^+$ 183.0816; Found 183.0823.

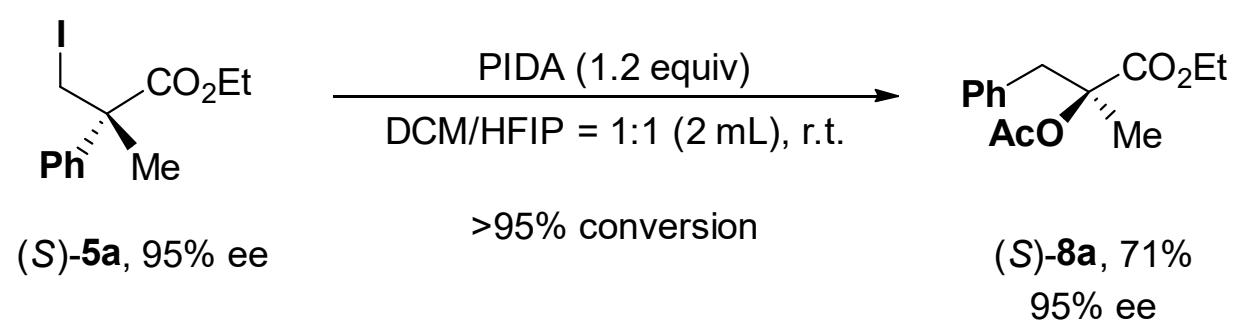

At room temperature, to a 10-mL test tube charged with a magnetic stirring bar were added substrates (*S*)-**5a** (63.6 mg, 0.2 mmol, 1.0 equiv) and PIDA (77.3 mg, 0.24 mmol, 1.2 equiv) in anhydrous DCM (1.0 mL) and HFIP (1.0 mL). The reaction mixture was allowed to stir at room temperature. After the reaction was complete (monitored by TLC), the mixture was quenched with H_2O. Then, the mixture was extracted twice with DCM. The combined organic extracts were dried over Na_2SO_4 and concentrated under reduced pressure. The residue was purified by silica gel flash column chromatography to give the desired product (*S*)-**8a**.

The absolute configuration was determined by X-ray single crystal analysis of the hydrolyzed product (*S*)-**8a**. The *ee* of (*S*)-**8a** was determined to be 95%.
$[\alpha]_D^{25}$ = -16.7 (c = 1.0, $CHCl_3$).
HPLC: The enantiomeric excess was determined by Daicel Chiralpack IG (0.46 cm x 25 cm), Hexanes / IPA = 99.5 / 0.5, 1.0 mL/min, λ = 254 nm, t (major) = 12.47 min, t (minor) = 14.57 min.

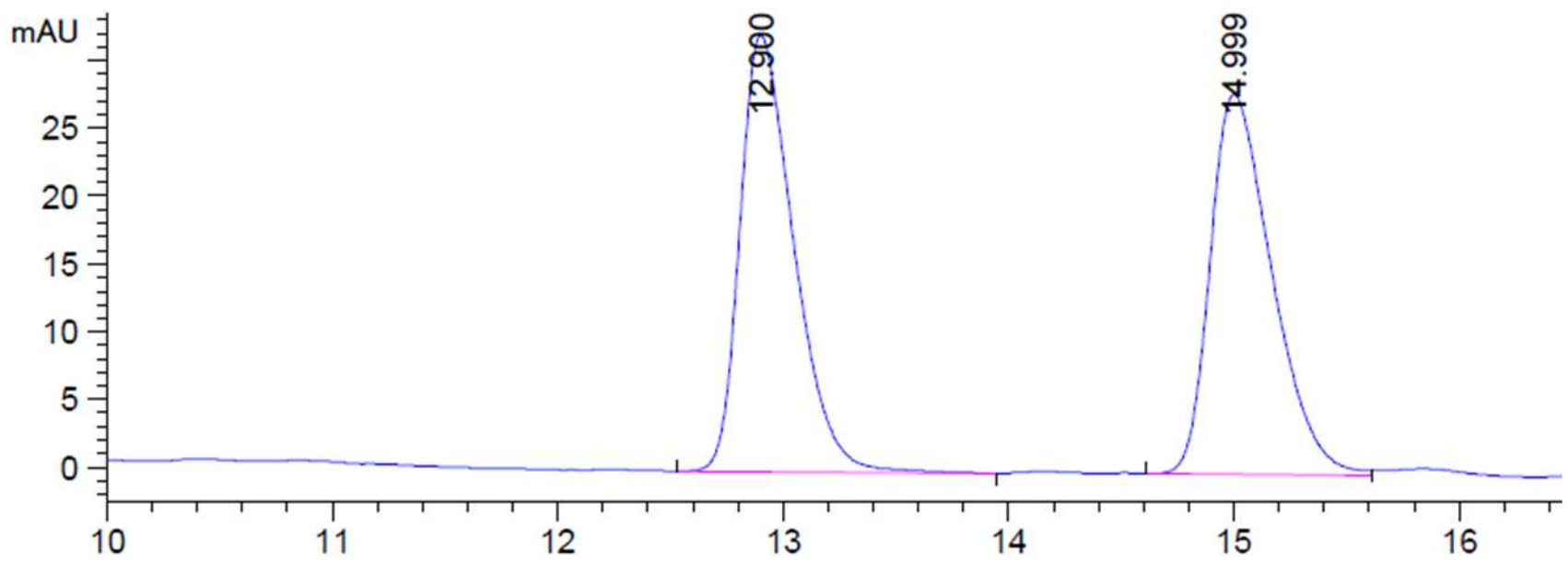

Peak #	RetTime [min]	Type	Width [min]	Area [mAU*s]	Height [mAU]	Area %
1	12.900	BB	0.2544	536.45807	32.19931	50.1668
2	14.999	BV	0.2898	532.89044	28.03298	49.8332

Totals : 1069.34851 60.23229

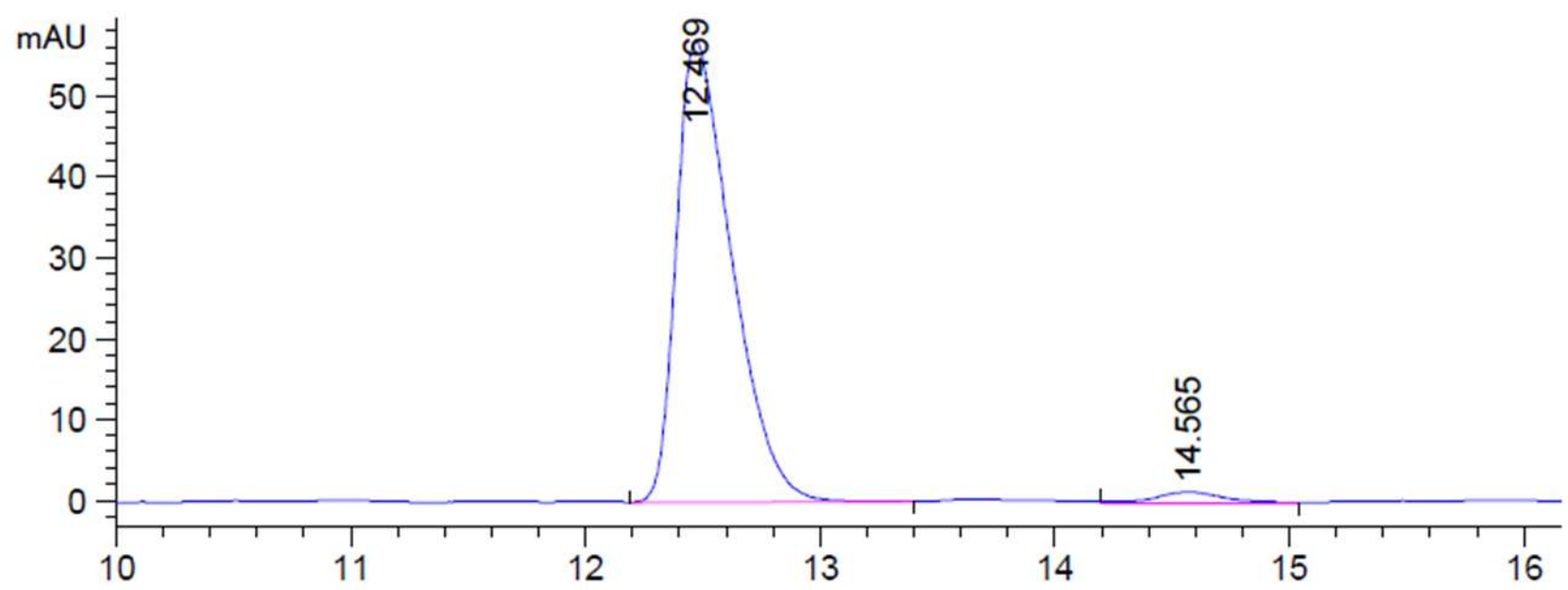

Peak #	RetTime [min]	Type	Width [min]	Area [mAU*s]	Height [mAU]	Area %
1	12.469	BB	0.2498	936.98950	57.00078	97.6717
2	14.565	VB	0.2646	22.33605	1.28624	2.3283
Totals :				959.32555	58.28702	

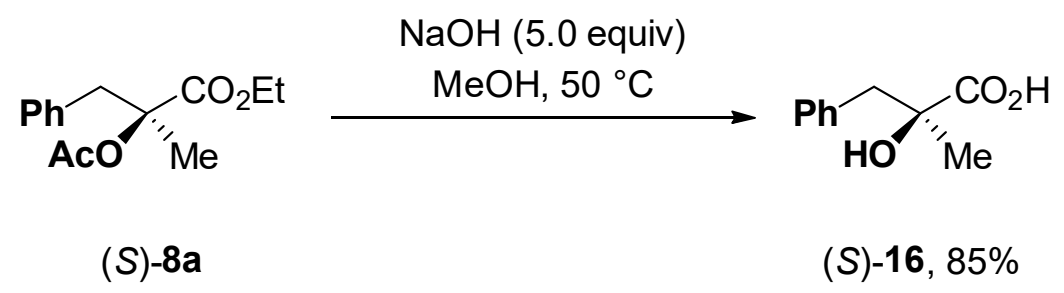

To a solution of the ester (*S*)-**8a** (25.0 mg, 0.1 mmol, 1.0 equiv) in MeOH (1 mL) was added NaOH (20.0 mg, 5.0 equiv) and the reaction mixture was allowed to warm to 50 ºC. After the reaction was complete (monitored by TLC), the residue was diluted with water, acidified until pH = 1 with HCl (3 M), and extracted twice with ethyl acetate. The combined organic extracts were dried over Na_2SO_4 and concentrated under reduced pressure. Flash chromatography on silica gel (hexanes:ethyl acetate 3:1) afforded (*R*)-**16** as colorless crystals.

The absolute configuration was determined by X-ray single crystal analysis.

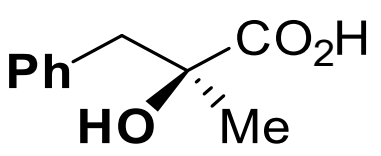

(*S*)-**16**. Colorless solid (15.3 mg, 85% yield).

Analytical data for (*S*)-**16**:

m.p. = 110 ~ 113 ºC.

$[\alpha]_D^{25}$ = -33.2 (c = 1.0, $CHCl_3$).

^{1}H NMR (400 MHz, $CDCl_3$) δ 7.34 – 7.27 (m, 3H), 7.25 – 7.20 (m, 2H), 3.15 (d, *J* = 13.6 Hz, 1H), 2.94 (d, *J* = 13.6 Hz, 1H), 1.54 (s, 3H).

^{13}C NMR (101 MHz, $CDCl_3$) δ 180.7, 135.3, 130.1, 128.4, 127.2, 75.1, 46.0, 25.7.

IR (neat) ν_{max} (cm^{-1}) = 2921, 2833, 1721, 1644, 1633, 1543, 1462, 1312, 1271, 1163, 1016, 954, 832, 812, 731, 693, 652, 636.

HRMS (ESI/QTOF) m/z: $[M + Na]^+$ Calcd for $C_{10}H_{12}NaO_3^+$ 203.0679; Found 203.0681.

General procedure for the measurement of reaction orders

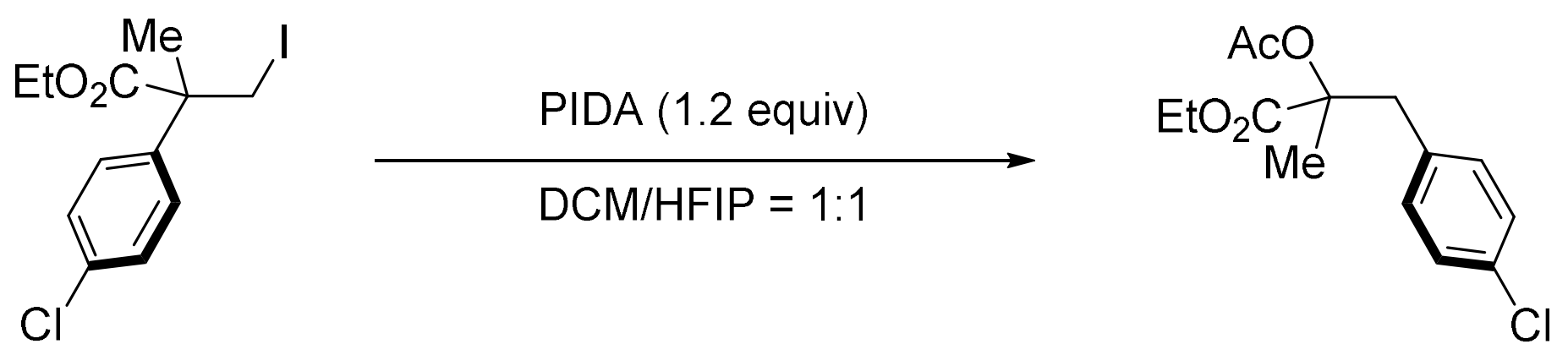

5e **8d**

At room temperature, a 10-mL test tube charged with a magnetic stirring bar were added substrates **5e** and PIDA in anhydrous DCM (1.0 mL) and HFIP (1.0 mL). The mixture was stirred at room temperature (at a rate of 500 rpm), and aliquots of the reaction mixture (100 μL) were withdrawn via syringe at specified time intervals. Each aliquot was evaporated under reduced pressure, dissovled in $CDCl_3$ (600 μL), passed through a small plug of cotton into an NMR tube. Quantitative ^1H NMR analysis was performed using mesitylene as an internal standard to monitor the reaction progress.

Reaction conditions for kinetic study of 5e

The concentrations of PIDA: 0.12 M

The concentrations of **5e**: 0.050 M, 0.075 M, 0.100 M, 0.150 M.

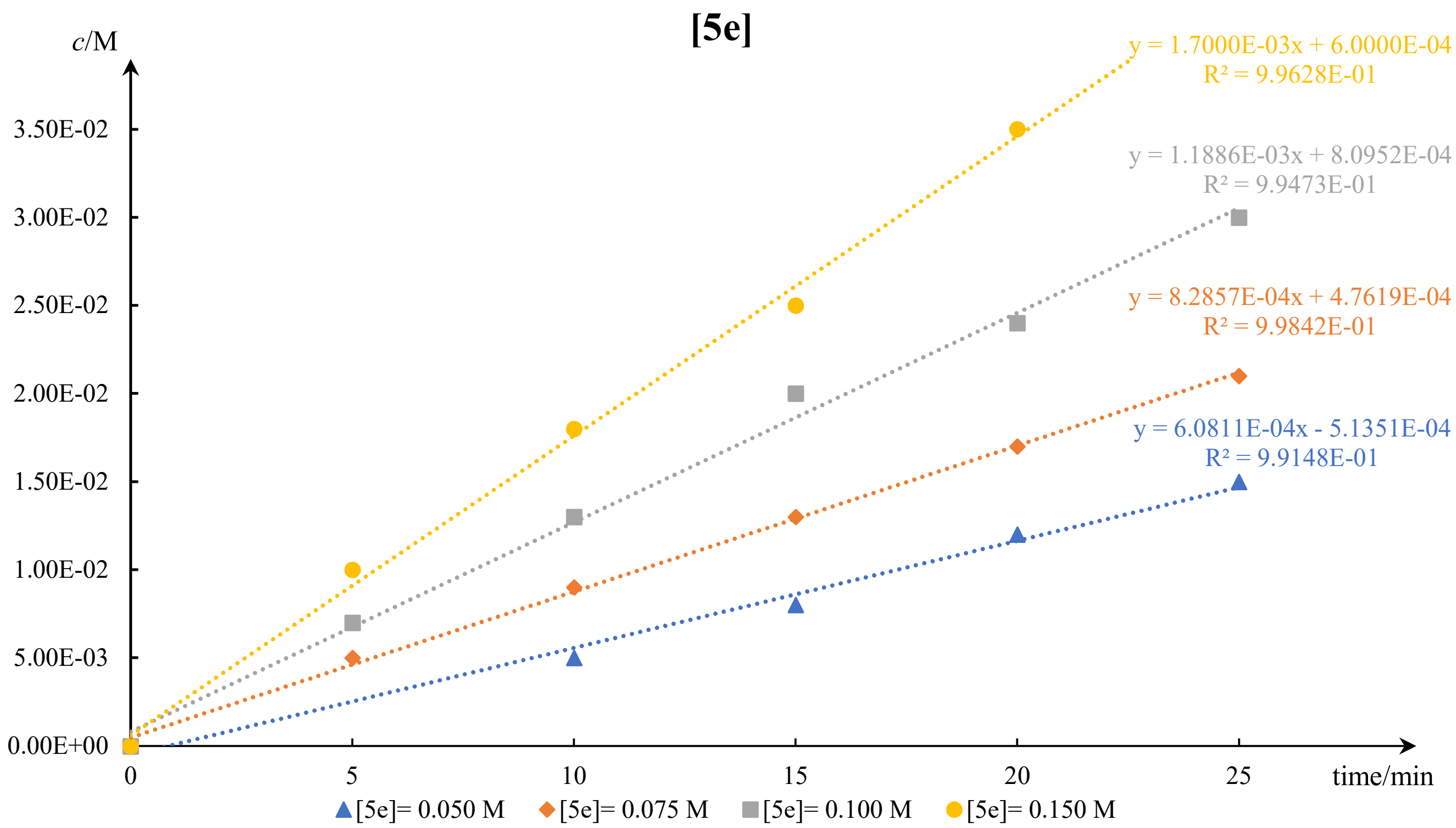

Figure S1. Time-dependent concentration of **8d** in reactions containing different concentrations of **5e**.

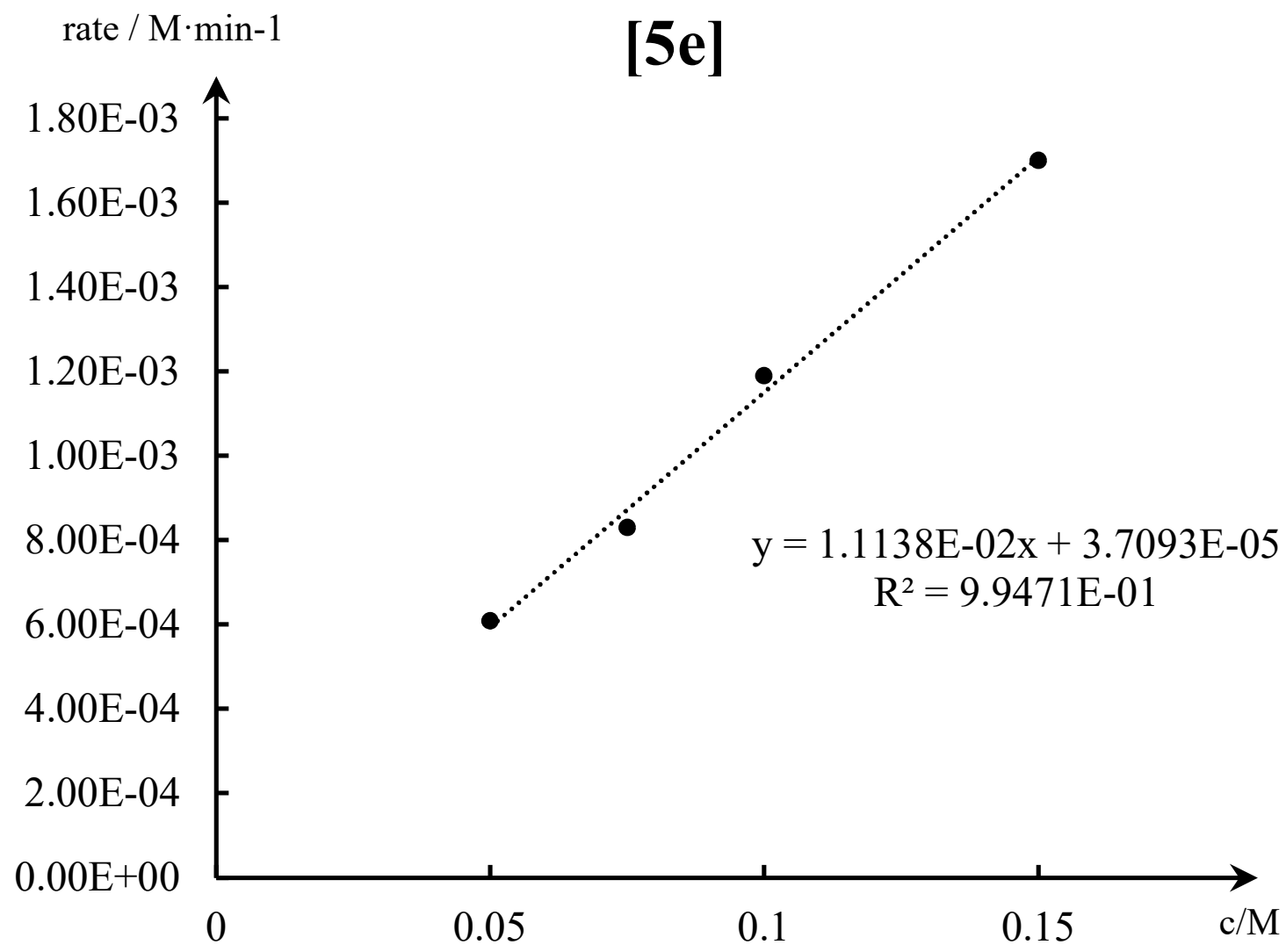

Figure S2. First-order dependence of the reaction rate on **5e** concentration.

Reaction conditions for kinetic study of PIDA

The concentrations of **5e**: 0.1 M

The concentrations of PIDA: 0.050 M, 0.120 M, 0.200 M, 0.250 M.

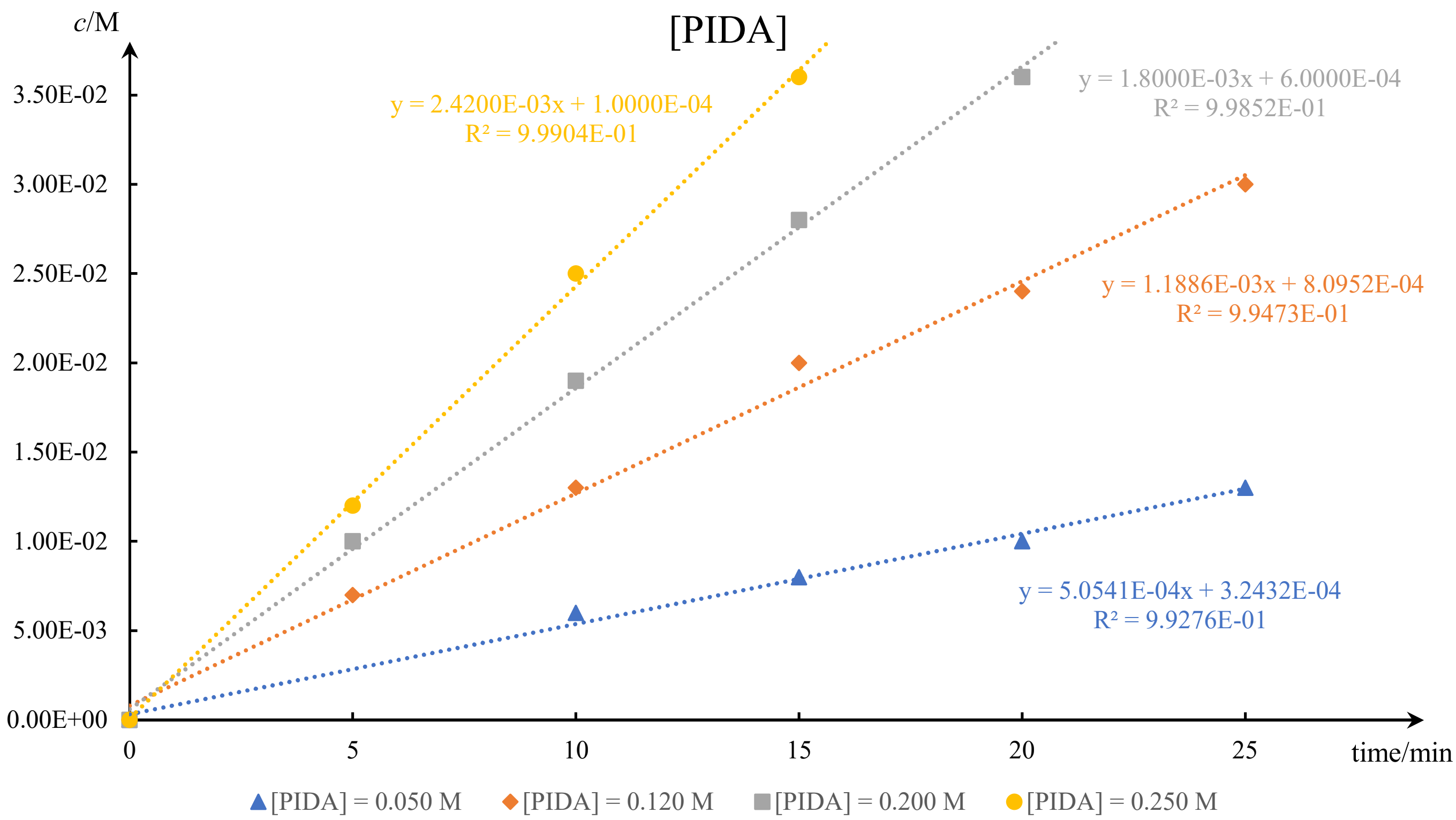

Figure S3. Time-dependent concentration of **8d** in reactions containing different concentrations of PIDA.

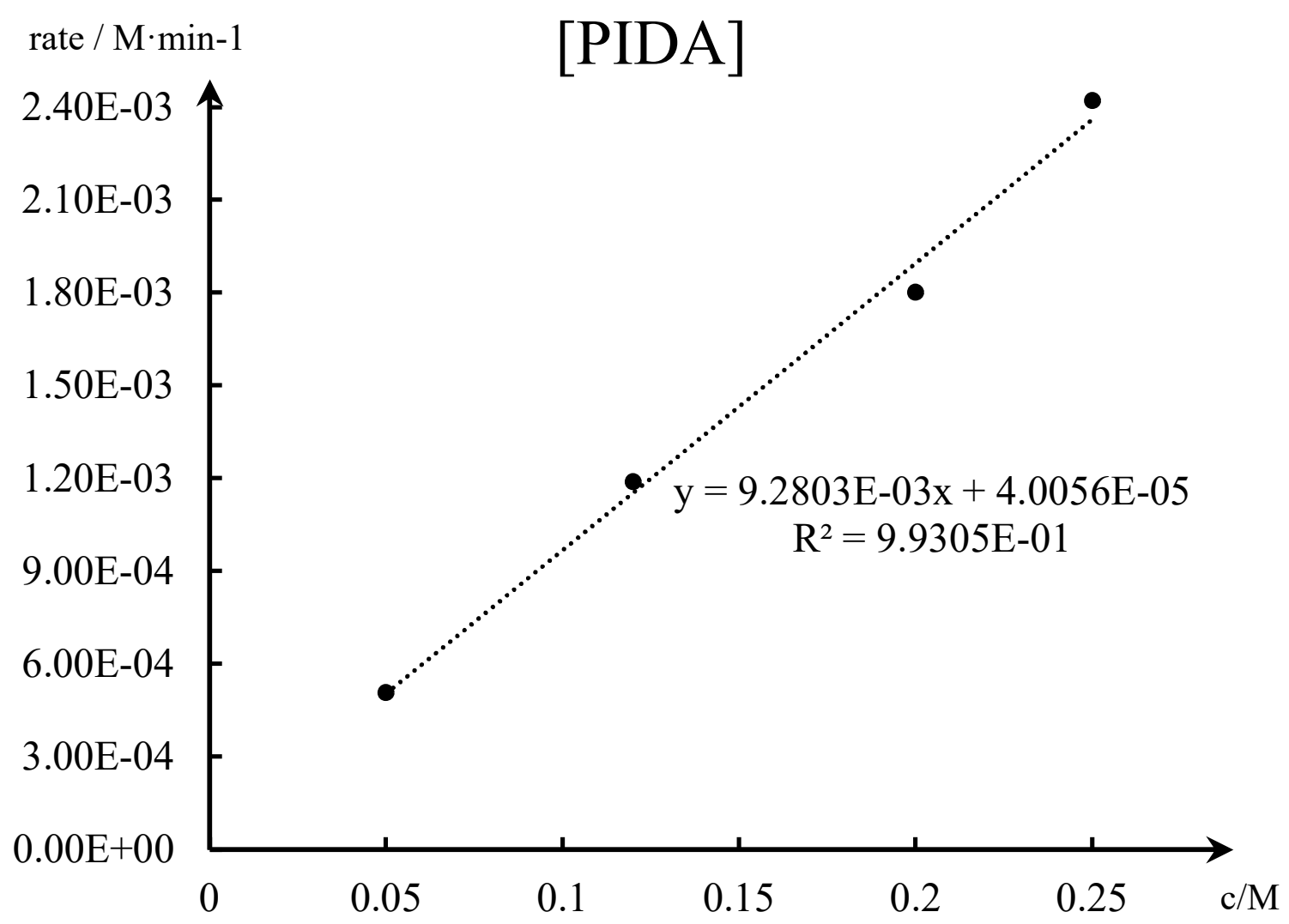

Figure S4. First-order dependence of the reaction rate on PIDA concentration.

X-Ray crystallographic data

X-Ray crystallographic data of (*R*)-**S18** (CCDC 2341813):

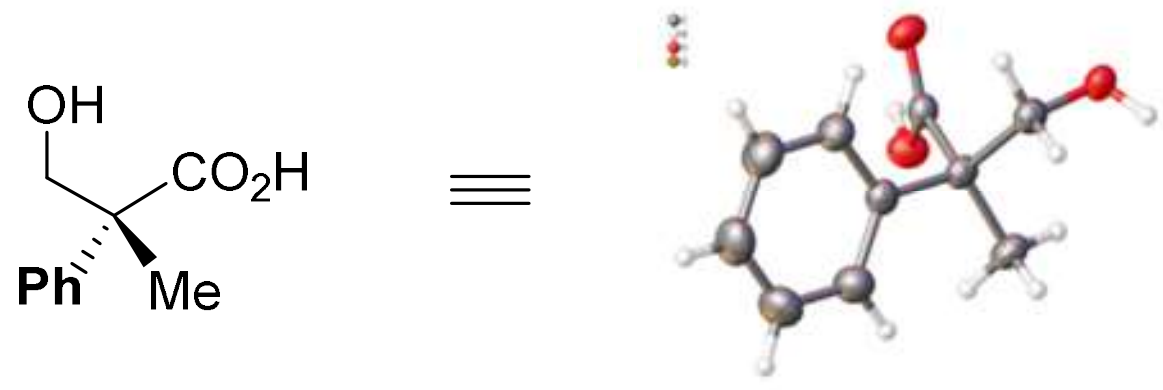

CCDC 2341813

Experimental. Single clear pale colourless plate-shaped crystals of (*R*)-**S18** were used as supplied. A suitable crystal with dimensions 0.29 × 0.13 × 0.06 mm^3 was selected and mounted on a SuperNova, Dual, Cu at home/near, Atlas diffractometer. The crystal was kept at a steady T = 200.00(10) K during data collection. The structure was solved with the ShelXT (Sheldrick, 2015) solution program using dual methods and by using Olex2 1.5 (Dolomanov et al., 2009) as the graphical interface. The model was refined with ShelXL 2019/3 (Sheldrick, 2015) using full matrix least squares minimisation on $\boldsymbol{F^2}$.

Crystal Data. $C_{10}H_{12}O_3$, M_r = 180.20, monoclinic, $P2_1$ (No. 4), a = 5.67736(19) Å, b = 5.9763(3) Å, c = 13.7316(5) Å, β = 96.905(3)°, $\alpha = \gamma$ = 90°, V = 462.53(3) Å^3, T = 200.00(10) K, Z = 2, Z' = 1, μ(Cu K_α) = 0.786, 4303 reflections measured, 1825 unique (R_{int} = 0.0229) which were used in all calculations. The final wR_2 was 0.0732 (all data) and R_1 was 0.0286 (I≥2 σ(I)).

Table S3. Crystal data and structure refinement for (*R*)-**S18**.

**Compound**	(*R*)-**S18**
Formula	$C_{10}H_{12}O_3$
$D_{calc.}$/ g cm^{-3}	1.294
μ/mm^{-1}	0.786
Formula Weight	180.20
Colour	clear pale colourless
Shape	plate
Size/mm^3	0.29×0.13×0.06
T/K	200.00(10)
Crystal System	monoclinic
Flack Parameter	0.0(2)
Hooft Parameter	0.05(12)
Space Group	$P2_1$
a/Å	5.67736(19)
b/Å	5.9763(3)
c/Å	13.7316(5)
α/°	90
β/°	96.905(3)
γ/°	90
V/Å^3	462.53(3)
Z	2
Z'	1
Wavelength/Å	1.54184
Radiation type	Cu K_α
Θ_{min}/°	3.242
Θ_{max}/°	76.600
Measured Refl's.	4303
Indep't Refl's	1825
Refl's I≥2 σ(I)	1722
R_{int}	0.0229
Parameters	167
Restraints	1
Largest Peak	0.154
Deepest Hole	-0.139
GooF	1.058
wR_2 (all data)	0.0732
wR_2	0.0702

Structure Quality Indicators

**Reflections:**	d min (CuKα) 2Θ=153.2° 0.79	I/σ(I) 33.8	Rint m=2.36 2.29%	Full 135.4° 99% to 153.2° 100
**Refinement:**	Shift 0.000	Max Peak 0.1	Min Peak -0.1	GooF 1.058

A clear pale colourless plate-shaped crystal with dimensions 0.29 × 0.13 × 0.06 mm^3 was mounted. Data were collected using a SuperNova, Dual, Cu at home/near, Atlas diffractometer operating at T = 200.00(10) K.

Data were measured using ω scans with Cu K_α radiation. The diffraction pattern was indexed and the total number of runs and images was based on the strategy calculation from the program CrysAlisPro system (CCD 43.112a 64-bit (release 01-03-2024)). The maximum resolution that was achieved was Θ = 76.600° (0.79 Å).

The unit cell was refined using CrysAlisPro 1.171.43.115a (Rigaku OD, 2024) on 2728 reflections, 63% of the observed reflections.

Data reduction, scaling and absorption corrections were performed using CrysAlisPro 1.171.43.115a (Rigaku OD, 2024). The final completeness is 100.00 % out to 76.600° in Θ. An analytical absorption correction was performed using CrysAlisPro 1.171.43.115a (Rigaku Oxford Diffraction, 2024). The analytical numeric absorption correction was done using a multifaceted crystal model based on expressions derived by R.C. Clark & J.S. Reid. (Clark, R. C. & Reid, J. S. (1995). Acta Cryst. A51, 887-897). The empirical absorption correction was done using spherical harmonics, implemented in SCALE3 ABSPACK scaling algorithm. The absorption coefficient μ of this crystal is 0.786 mm^{-1} at this wavelength (λ = 1.54184Å) and the minimum and maximum transmissions are 0.896 and 0.964.

The structure was solved and the space group $P2_1$ (# 4) determined by the ShelXT (Sheldrick, 2015) structure solution program using dual methods and refined by full matrix least squares minimisation on $\boldsymbol{F}^2$ using version 2019/3 of ShelXL 2019/3 (Sheldrick, 2015). All non-hydrogen atoms were refined anisotropically. Hydrogen atom positions were calculated geometrically and refined freely.

_refine_special_details: Refined as a 2-component inversion twin.

There is a single formula unit in the asymmetric unit, which is represented by the reported sum formula. In other words: Z is 2 and Z' is 1. The moiety formula is C10 H12 O3.

The Flack parameter was refined to 0.0(2). Determination of absolute structure using Bayesian statistics on Bijvoet differences using the Olex2 results in 0.05(12). The chiral atoms in this structure are: C2(R). Note: The Flack parameter is used to determine chirality of the crystal studied, the value should be near 0, a value of 1 means that the stereochemistry is wrong and the model should be inverted. A value of 0.5 means that the crystal consists of a racemic mixture of the two enantiomers.

X-Ray crystallographic data of (*S*)-**15** (CCDC 2331075):

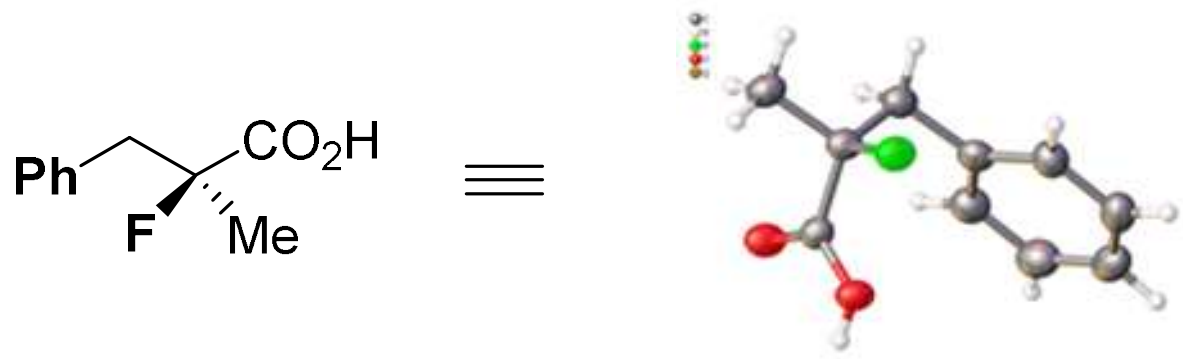

CCDC 2331075

Experimental. Single clear pale colourless irregular-shaped crystals of (*S*)-**15** were used as supplied. A suitable crystal with dimensions 0.41 × 0.13 × 0.07 mm^3 was selected and mounted on a XtaLAB Synergy R, DW system, HyPix-Arc 150 diffractometer. The crystal was kept at a steady T = 140.00(10) K during data collection. The structure was solved with the ShelXT (Sheldrick, 2015) solution program using dual methods and by using Olex2 1.5 (Dolomanov et al., 2009) as the graphical interface. The model was refined with ShelXL 2019/3 (Sheldrick, 2015) using full matrix least squares minimisation on $\boldsymbol{F}^2$.

Crystal Data. $C_{10}H_{11}O_2F$, M_r = 182.19, monoclinic, $P2_1$ (No. 4), a = 8.1805(2) Å, b = 5.15956(18) Å, c = 11.1860(3) Å, β = 100.004(3)°, $\alpha = \gamma$ = 90°, V = 464.96(2) Å^3, T = 140.00(10) K, Z = 2, Z' = 1, μ(Cu K_α) = 0.866, 10248 reflections measured, 1763 unique (R_{int} = 0.0343) which were used in all calculations. The final wR_2 was 0.0820 (all data) and R_1 was 0.0304 (I≥2 σ(I)).

Table S4. Crystal data and structure refinement for (*S*)-**15**.

**Compound**	(*S*)-**15**
Formula	$C_{10}H_{11}O_2F$
$D_{calc.}$/ g cm^{-3}	1.301
μ/mm^{-1}	0.866
Formula Weight	182.19
Colour	clear pale colourless
Shape	irregular
Size/mm^3	0.41×0.13×0.07
T/K	140.00(10)
Crystal System	monoclinic
Flack Parameter	0.05(17)
Hooft Parameter	0.05(6)
Space Group	$P2_1$
a/Å	8.1805(2)
b/Å	5.15956(18)
c/Å	11.1860(3)
α/°	90
β/°	100.004(3)
γ/°	90
V/Å^3	464.96(2)
Z	2
Z'	1
Wavelength/Å	1.54184
Radiation type	Cu K_α
Θ_{min}/°	4.013
Θ_{max}/°	75.041
Measured Refl's.	10248
Indep't Refl's	1763
Refl's I≥2 σ(I)	1685
R_{int}	0.0343
Parameters	124
Restraints	1
Largest Peak	0.186
Deepest Hole	-0.181
GooF	1.119
wR_2 (all data)	0.0820

Structure Quality Indicators

**Reflections:**	d min (CuKα) 2Θ=150.1° 0.80	I/σ(I) 52.9	Rint m=5.82 3.43%	Full 135.4° 96% to 150.1° 98.4
**Refinement:**	Shift 0.000	Max Peak 0.2	Min Peak -0.2	GooF 1.119

A clear pale colourless irregular-shaped crystal with dimensions 0.41 × 0.13 × 0.07 mm^3 was mounted. Data were collected using a XtaLAB Synergy R, DW system, HyPix-Arc 150 diffractometer operating at T = 140.00(10) K.

Data were measured using ω scans with Cu K_α radiation. The diffraction pattern was indexed and the total number of runs and images was based on the strategy calculation from the program CrysAlisPro system (CCD 43.105a 64-bit (release 11-01-2024)). The maximum resolution that was achieved was Θ = 75.041° (0.80 Å).

The unit cell was refined using CrysAlisPro 1.171.43.106a (Rigaku OD, 2024) on 7614 reflections, 74% of the observed reflections.

Data reduction, scaling and absorption corrections were performed using CrysAlisPro 1.171.43.106a (Rigaku OD, 2024). The final completeness is 98.40 % out to 75.041° in Θ. A gaussian absorption correction was performed using CrysAlisPro 1.171.43.106a (Rigaku Oxford Diffraction, 2024). The numerical absorption correction was based on gaussian integration over a multifaceted crystal model. The empirical absorption correction was done using spherical harmonics, implemented in SCALE3 ABSPACK scaling algorithm. The absorption coefficient μ of this crystal is 0.866 mm^{-1} at this wavelength (λ = 1.54184Å) and the minimum and maximum transmissions are 0.621 and 1.000.

The structure was solved and the space group $P2_1$ (# 4) determined by the ShelXT (Sheldrick, 2015) structure solution program using dual methods and refined by full matrix least squares minimisation on $\boldsymbol{F}^2$ using version 2019/3 of ShelXL (Sheldrick, 2015). All non-hydrogen atoms were refined anisotropically. Most hydrogen atom positions were calculated geometrically and refined using the riding model, but some hydrogen atoms were refined freely.

_refine_special_details: Refined as a 2-component inversion twin.

There is a single formula unit in the asymmetric unit, which is represented by the reported sum formula. In other words: Z is 2 and Z' is 1. The moiety formula is C10 H11 F O2.

The Flack parameter was refined to 0.05(17). Determination of absolute structure using Bayesian statistics on Bijvoet differences using the Olex2 results in 0.05(6). The chiral atoms in this structure are: C2(S). Note: The Flack parameter is used to determine chirality of the crystal studied, the value should be near 0, a value of 1 means that the stereochemistry is wrong and the model should be inverted. A value of 0.5 means that the crystal consists of a racemic mixture of the two enantiomers.

X-Ray crystallographic data of (*S*)-**16** (CCDC 2391886):

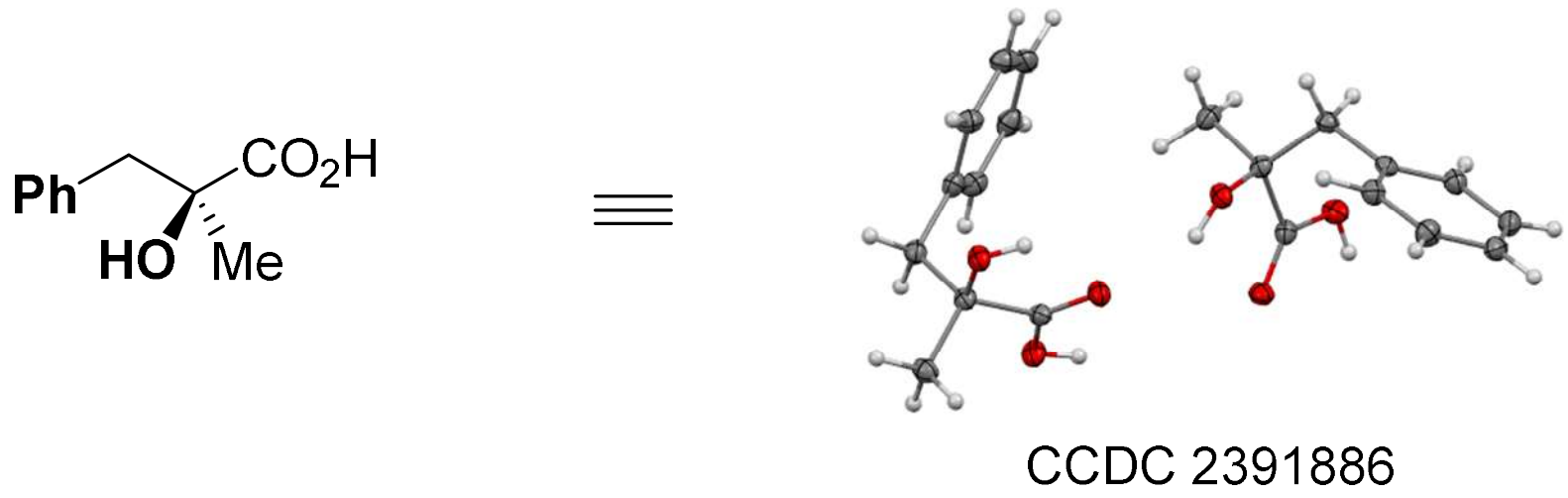

Experimental. Single colourless needle-shaped crystals of (*S*)-**16** were used as supplied. A suitable crystal with dimensions 0.37 × 0.03 × 0.03 mm^3 was selected and mounted on a SuperNova, Dual, Cu at home/near, AtlasS2 diffractometer. The crystal was kept at a steady T = 140.00(10) K during data collection. The structure was solved with the ShelXS (Sheldrick, 2008) solution program using direct methods and by using Olex2 1.5 (Dolomanov et al., 2009) as the graphical interface. The model was refined with ShelXL 2019/3 (Sheldrick, 2015) using full matrix least squares minimisation on $\boldsymbol{F}^2$.

Crystal Data. $C_{10}H_{12}O_3$, M_r = 180.20, monoclinic, $P2_1$ (No. 4), a = 5.81404(17) Å, b = 12.2255(3) Å, c = 13.3087(4) Å, β = 93.433(3)°, $\alpha = \gamma$ = 90°, V = 944.28(5) Å^3, T = 140.00(10) K, Z = 4, Z' = 2, μ(Cu K_α) = 0.770, 6860 reflections measured, 3602 unique (R_{int} = 0.0388) which were used in all calculations. The final wR_2 was 0.0823 (all data) and R_1 was 0.0352 (I≥2 σ(I)).

Table S5. Crystal data and structure refinement for (*S*)-**16**.

**Compound**	(*S*)-**16**
Formula	$C_{10}H_{12}O_3$
$D_{calc.}$/ g cm^{-3}	1.268
μ/mm^{-1}	0.770
Formula Weight	180.20
Colour	colourless
Shape	needle
Size/mm^3	0.37×0.03×0.03
T/K	140.00(10)
Crystal System	monoclinic
Flack Parameter	-0.1(3)
Hooft Parameter	-0.04(16)
Space Group	$P2_1$
a/Å	5.81404(17)
b/Å	12.2255(3)
c/Å	13.3087(4)
α/°	90
β/°	93.433(3)
γ/°	90
V/Å^3	944.28(5)
Z	4
Z'	2
Wavelength/Å	1.54184
Radiation type	Cu K$_\alpha$
Θ_{min}/°	3.327
Θ_{max}/°	72.358
Measured Refl's.	6860
Indep't Refl's	3602
Refl's I≥2 σ(I)	3209
R_{int}	0.0388
Parameters	333
Restraints	7
Largest Peak	0.169
Deepest Hole	-0.142
GooF	1.008
wR_2 (all data)	0.0823

Structure Quality Indicators

**Reflections:**	d min (CuKα) 2Θ=144.7° 0.81	I/σ(I) 18.6	Rint m=1.91 3.88%	Full 135.4° 98% to 144.7° 99.9
**Refinement:**	Shift 0.000	Max Peak 0.2	Min Peak -0.1	GooF 1.008

A colourless needle-shaped crystal with dimensions 0.37 × 0.03 × 0.03 mm^3 was mounted. Data were collected using a SuperNova, Dual, Cu at home/near, AtlasS2 diffractometer operating at T = 140.00(10) K.

Data were measured using ω scans with Cu K_α radiation. The diffraction pattern was indexed and the total number of runs and images was based on the strategy calculation from the program CrysAlisPro system (CCD 43.141a 64-bit (release 04-10-2024)). The maximum resolution that was achieved was Θ = 72.358° (0.81 Å).

The unit cell was refined using CrysAlisPro 1.171.43.141a (Rigaku OD, 2024) on 2948 reflections, 43% of the observed reflections.

Data reduction, scaling and absorption corrections were performed using CrysAlisPro 1.171.43.141a (Rigaku OD, 2024). The final completeness is 99.90 % out to 72.358° in Θ. A gaussian absorption correction was performed using CrysAlisPro 1.171.43.141a (Rigaku Oxford Diffraction, 2024). The numerical absorption correction was based on gaussian integration over a multifaceted crystal model. The empirical absorption correction was done using spherical harmonics, implemented in SCALE3 ABSPACK scaling algorithm. The absorption coefficient μ of this crystal is 0.770 mm^{-1} at this wavelength (λ = 1.54184Å) and the minimum and maximum transmissions are 0.780 and 1.000.

The structure was solved and the space group $P2_1$ (# 4) determined by the ShelXS (Sheldrick, 2008) structure solution program using direct methods and refined by full matrix least squares minimisation on $\boldsymbol{F}^2$ using version 2019/3 of ShelXL (Sheldrick, 2015). All non-hydrogen atoms were refined anisotropically. Hydrogen atom positions were calculated geometrically and refined freely.

_refine_special_details: Refined as a 2-component inversion twin.

The value of Z' is 2. This means that there are two independent molecules in the asymmetric unit. The moiety formula is C10 H12 O3.

The Flack parameter was refined to -0.1(3). Determination of absolute structure using Bayesian statistics on Bijvoet differences using the Olex2 results in -0.04(16). The chiral atoms in this structure are: C1(S), C11(S). Note: The Flack parameter is used to determine chirality of the crystal studied, the value should be near 0, a value of 1 means that the stereochemistry is wrong and the model should be inverted. A value of 0.5 means that the crystal consists of a racemic mixture of the two enantiomers.

Computational studies

Computational Details: The geometries of all species were optimized in the gas phase using the M06-2X[6,7] functional appended along with the def2-SVPD basis set.[8] Species were characterized as either minimum (zero imaginary frequencies) or transition states (one imaginary frequency) based on analysis of the vibrational frequencies. Refined energies were then obtained through single point computations on the optimized M06-2X/def2-SVPD geometries at the M06-2X/def2-TZVPPD level including implicit solvation (in dichloromethane) using the SMD model.[9] Free energy corrections were determined using the quasi rigid-rotor harmonic oscillator model[10] and were corrected for translational entropy in solution using the method proposed by Martin, Hay, and Pratt[11] as implemented in Goodvibes[12,13]. Reported free energies are obtained by summing the electronic energies at the M06-2X/def2-TZVPPD level and the free energy corrections at the M06-2X/def2-SVPD level. Charge distribution analysis employed CM5 charges[14]. All computations employed Gaussian16.[15]

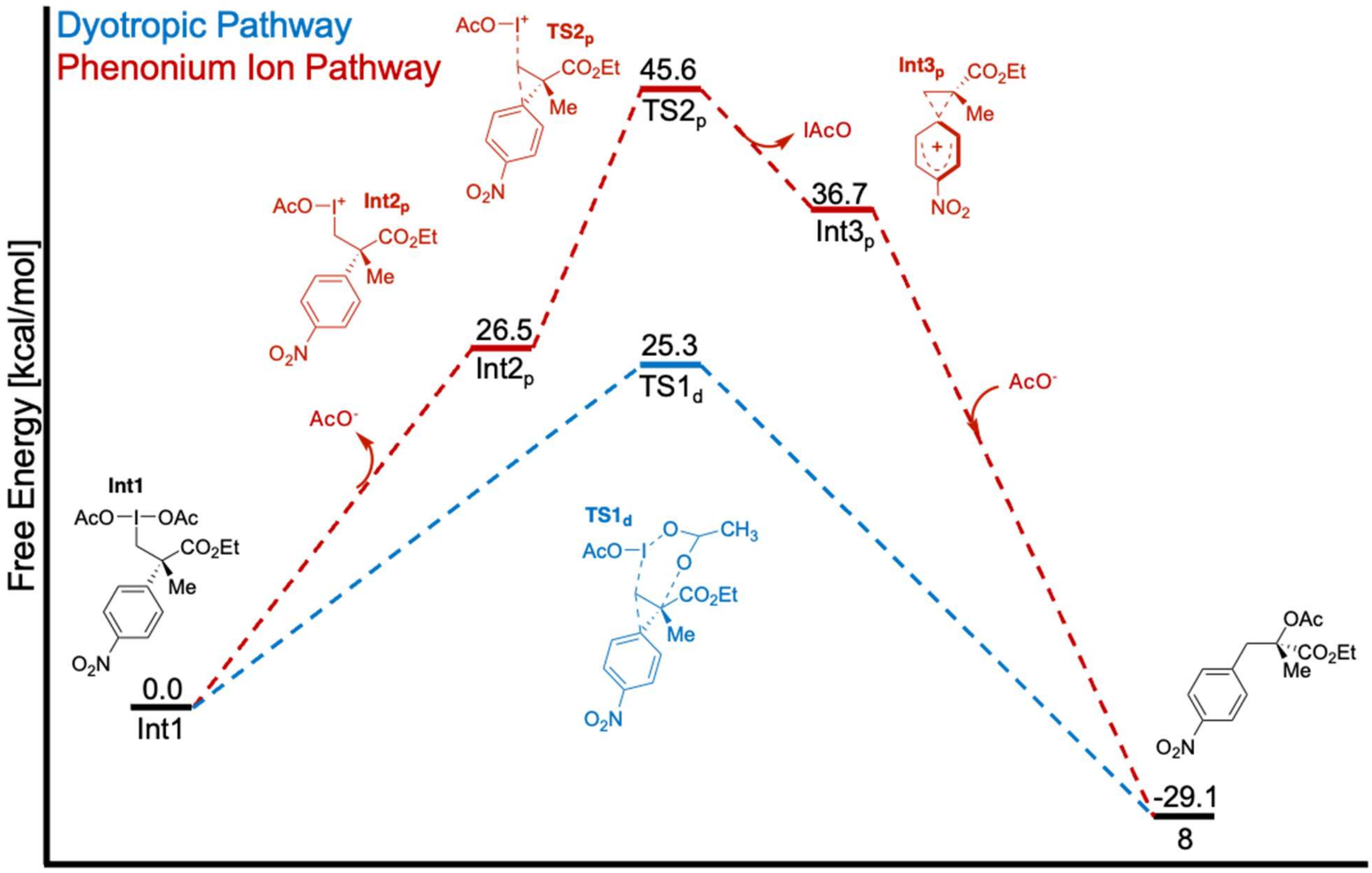

Figure S5. Computed free energy profiles of the dyotropic (blue) and phenonium ion (red) pathways for the migratory acyloxy arylation reaction, obtained at the M06-2X/def2-TZVPPD(SMD, DCM)//M06-2X/def2-SVPD level of theory.

Table S6. Electronic energies, free energy corrections, singlet point energies (each in Hartree) and relative Gibbs free energies (relative to Int1, in kcal/mol) of relevant species.

	M06-2X/def2-SVPD Electronic Energy	M06-2X/def2-SVPD Free Energy Correction	M06-2X/def2-TZVPPD Single Point Energy	Total FE	Relative FE
**Reference Molecules**					
HF	-100.337217	-0.003863	-100.462310	-100.466173	--
IF	-397.313303	-0.018855	-397.449449	-397.468304	--
F-(HF)2	-300.537013	-0.000618	-300.972664	-300.973282	--
AcO-	-228.270545	0.025641	-228.616015	-228.590374	--
IAcO	-525.816897	0.022226	-526.094140	-526.071914	--
**R = NO_2**					
**Dyotropic Pathway**					
Int1	-1518.118136	0.226106	-1519.527959	-1519.301853	0.0
$TS1_d$	-1518.072471	0.222292	-1519.489993	-1519.267701	21.4
6h	-920.113405	0.209757	-921.142907	-920.933150	-20.0
**Phenonium Ion Pathway**					
Int1	-1518.118136	0.226106	-1519.527959	-1519.301853	0.0
$TS1_p$	-1518.093636	0.226947	-1519.504864	-1519.277917	15.0
$Int2_p$	-1217.401728	0.207303	-1218.530158	-1218.322855	3.6
$TS2_p$	-1217.367658	0.202658	-1218.490568	-1218.287910	25.5
$Int3_p$	-820.048372	0.207236	-821.035875	-820.828639	19.8
$TS3_p$	-1120.743283	0.224799	-1122.020471	-1121.795672	23.8
6h	-920.113405	0.209757	-921.142907	-920.933150	-20.0
**R = H**					
**Dyotropic Pathway**					
Int1	-1313.832559	0.226419	-1315.010393	-1314.783974	0.0
$TS1_d$	-1313.796755	0.222893	-1314.981836	-1314.758943	15.7
6a	-715.826736	0.211337	-716.626007	-716.414670	-19.7
**Phenonium Ion Pathway**					
Int1	-1313.832559	0.226419	-1315.010393	-1314.783974	0.0
$TS1_p$	-1313.808087	0.227397	-1314.987421	-1314.760024	15.0
$Int2_p$	-1013.125016	0.207761	-1014.014101	-1013.806340	2.7
$TS2_p$	-1013.099073	0.203885	-1013.982911	-1013.779026	19.9
$Int3_p$	-615.782745	0.208615	-616.532786	-616.324171	11.4
$TS3_p$	-916.458758	0.225414	-917.507111	-917.281697	21.3
6a	-715.826736	0.211337	-716.626007	-716.414670	-19.7
**R = AcO**					
**Dyotropic Pathway**					
Int1	-1574.404122	0.298067	-1575.847470	-1575.549403	0.0
$TS1_d$	-1574.350370	0.292273	-1575.801376	-1575.509103	25.3
8	-1048.610699	0.255081	-1049.778989	-1049.523908	-29.1
**Phenonium Ion Pathway**					
Int1	-1574.404122	0.298067	-1575.847470	-1575.549403	0.0
$Int2_p$	-1345.909506	0.251133	-1347.167933	-1346.916800	26.5
$TS2_p$	-1345.871766	0.244931	-1347.131268	-1346.886337	45.6
$Int3_p$	-820.048372	0.207237	-821.035875	-820.828638	36.7
8	-1048.610699	0.255081	-1049.778989	-1049.523908	-29.1

Table S7. Intrinsic reaction coordinate energies (in hartree), relative energies (in kcal/mol), bond distances (in Å), and charges (in e-) for the dyotropic pathway ($TS1_d$).

IRC	Elec. Energy	Relative Energy	C_b-I	C_b-C_{ipso}	C_a-F	I-Charge	C6-Charge	C12-Charge	C15-Charge	C13-Charge	C16-Charge	C14-Charge	Ring-Charge
Step: -20	-1518.096519	-15.09	2.336	2.312	2.903	0.599	0.009	-0.079	-0.080	-0.065	-0.068	0.079	-0.204
Step: -19	-1518.094873	-14.06											
Step: -18	-1518.093101	-12.95											
Step: -17	-1518.091213	-11.76											
Step: -16	-1518.089236	-10.52											
Step: -15	-1518.087202	-9.24	2.532	2.202	2.909	0.520	0.014	-0.073	-0.075	-0.063	-0.066	0.083	-0.181
Step: -14	-1518.085148	-7.96											
Step: -13	-1518.083124	-6.68											
Step: -12	-1518.081176	-5.46											
Step: -11	-1518.079353	-4.32											
Step: -10	-1518.077698	-3.28	2.771	2.042	2.900	0.404	0.021	-0.061	-0.063	-0.060	-0.063	0.093	-0.133
Step: -9	-1518.076255	-2.37											
Step: -8	-1518.075060	-1.62											
Step: -7	-1518.074135	-1.04											
Step: -6	-1518.073471	-0.63											
Step: -5	-1518.073027	-0.35	3.005	1.864	2.876	0.292	0.024	-0.041	-0.045	-0.056	-0.060	0.107	-0.071
Step: -4	-1518.072755	-0.18											
Step: -3	-1518.072600	-0.08											
Step: -2	-1518.072518	-0.03											
Step: -1	-1518.072481	-0.01											
Step: TS	-1518.072471	0.00	3.159	1.745	2.835	0.242	0.024	-0.028	-0.033	-0.055	-0.058	0.115	-0.036
Step: 1	-1518.072503	-0.02											
Step: 2	-1518.072538	-0.04											
Step: 3	-1518.072586	-0.07											
Step: 4	-1518.072645	-0.11											
Step: 5	-1518.072719	-0.16	3.246	1.684	2.783	0.223	0.023	-0.025	-0.031	-0.054	-0.058	0.116	-0.029
Step: 6	-1518.072807	-0.21											
Step: 7	-1518.072913	-0.28											
Step: 8	-1518.073041	-0.36											
Step: 9	-1518.073192	-0.45											
Step: 10	-1518.073369	-0.56	3.315	1.634	2.713	0.212	0.023	-0.030	-0.035	-0.055	-0.058	0.113	-0.041
Step: 11	-1518.073572	-0.69											
Step: 12	-1518.073799	-0.83											
Step: 13	-1518.074045	-0.99											
Step: 14	-1518.074305	-1.15											
Step: 15	-1518.074575	-1.32	3.372	1.595	2.624	0.206	0.022	-0.040	-0.045	-0.055	-0.059	0.107	-0.070
Step: 16	-1518.074851	-1.49											
Step: 17	-1518.075134	-1.67											
Step: 18	-1518.075421	-1.85											
Step: 19	-1518.075713	-2.03											
Step: 20	-1518.076009	-2.22	3.416	1.573	2.530	0.205	0.020	-0.050	-0.055	-0.056	-0.060	0.100	-0.102

Table S8. Intrinsic reaction coordinate energies (in hartree), relative energies (in kcal/mol), bond distances (in Å), and charges (in e-) for the phenonium ion pathway ($TS2_p$).

IRC	Elec. Energy	Relative Energy	C_b-I	C_b-C_{ipso}	I-Charge	C6-Charge	C12-Charge	C15-Charge	C13-Charge	C16-Charge	C14-Charge	Ring-Charge
Step: -20	-1217.394714	-16.98	2.223	2.378	0.683	-0.006	-0.083	-0.074	-0.057	-0.053	0.091	-0.183
Step: -19	-1217.393659	-16.32										
Step: -18	-1217.392513	-15.60										
Step: -17	-1217.391274	-14.82										
Step: -16	-1217.389937	-13.98										
Step: -15	-1217.388491	-13.07	2.296	2.326	0.662	-0.004	-0.081	-0.073	-0.056	-0.053	0.093	-0.174
Step: -14	-1217.386921	-12.09										
Step: -13	-1217.385218	-11.02										
Step: -12	-1217.383386	-9.87										
Step: -11	-1217.381455	-8.66										
Step: -10	-1217.379474	-7.41	2.484	2.213	0.590	0.002	-0.074	-0.068	-0.054	-0.051	0.098	-0.147
Step: -9	-1217.377492	-6.17										
Step: -8	-1217.375568	-4.96										
Step: -7	-1217.373762	-3.83										
Step: -6	-1217.372127	-2.80										
Step: -5	-1217.370712	-1.92	2.724	2.049	0.472	0.011	-0.059	-0.053	-0.050	-0.048	0.110	-0.090
Step: -4	-1217.369548	-1.19										
Step: -3	-1217.368662	-0.63										
Step: -2	-1217.368067	-0.26										
Step: -1	-1217.367749	-0.06										
Step: TS	-1217.367658	0.00	2.970	1.867	0.354	0.017	-0.035	-0.031	-0.047	-0.046	0.127	-0.016
Step: 1	-1217.367725	-0.04										
Step: 2	-1217.367878	-0.14										
Step: 3	-1217.368053	-0.25										
Step: 4	-1217.368207	-0.34										
Step: 5	-1217.368332	-0.42	3.166	1.735	0.298	0.016	-0.017	-0.015	-0.045	-0.044	0.139	0.033
Step: 6	-1217.368434	-0.49										
Step: 7	-1217.368526	-0.54										
Step: 8	-1217.368606	-0.59										
Step: 9	-1217.368688	-0.65										
Step: 10	-1217.368764	-0.69	3.226	1.716	0.286	0.016	-0.014	-0.012	-0.045	-0.044	0.140	0.041
Step: 11	-1217.368835	-0.74										
Step: 12	-1217.368901	-0.78										
Step: 13	-1217.368963	-0.82										
Step: 14	-1217.369021	-0.86										
Step: 15	-1217.369075	-0.89	3.277	1.703	0.277	0.016	-0.013	-0.011	-0.045	-0.044	0.142	0.046
Step: 16	-1217.369126	-0.92										
Step: 17	-1217.369174	-0.95										
Step: 18	-1217.369219	-0.98										
Step: 19	-1217.369263	-1.01										
Step: 20	-1217.369305	-1.03	3.315	1.692	0.270	0.016	-0.012	-0.010	-0.044	-0.044	0.142	0.049

References

1. L. Song, Y. Zhou, H. Liang, H. Li, Y. Lai, H. Yao, R. Lin, and R. Tong, "Two Green Protocols for Halogenative Semipinacol Rearrangement," *Journal of Organic Chemistry* 88 (2023): 504−512.
2. T. Singha, G. A. Kadam, and D. P. Hari, "Photocatalyzed Dowd−Beckwith Radical-polar Crossover Reaction for the Synthesis of Medium-sized Carbocyclic Compounds," *Chemical Science* 14 (2023), 6930−6935.
3. G. Yang, H. Wu, S. Gallarati, C. Corminboeuf, Q. Wang, and J. Zhu, "Migrative Carbofluorination of Saturated Amides Enabled by Pd-Based Dyotropic Rearrangement," *Journal of the American Chemical Society* 144 (2022): 14047−14052.
4. P. D. María, B. Kossmann, N. Potgrave, S. Buchholz, H. Trauthwein, O. May, and H. Gröger, "Improved Process for the Enantioselective Hydrolysis of Prochiral Diethyl Malonates Catalyzed by Pig Liver Esterase," *Synlett* 11 (2005): 1746−1748.
5. P. Xu, and Z. Huang, "Catalytic Reductive Desymmetrization of Malonic Esters." *Nature Chemistry* 13 (2021): 634−642.
6. Y. Zhao, and D. G. Truhlar, "The M06 Suite of Density Functionals for Main Group Thermochemistry, Thermochemical Kinetics, Noncovalent Interactions, Excited States, and Transition Elements: Two New Functionals and Systematic Testing of Four M06-Class Functionals and 12 Other Functionals," *Theoretical Chemistry Accounts* 120 (2008): 215−241.
7. Y. Zhao, and D. G. Truhlar, "Density Functionals with Broad Applicability in Chemistry," *Accounts of Chemical Research* 41 (2008): 157−167.
8. F. Weigend, and R. Ahlrichs, "Balanced Basis Sets of Split Valence, Triple Zeta Valence and Quadruple Zeta Valence Quality for H to Rn: Design and Assessment of Accuracy," *Physical Chemistry Chemical Physics* 7 (2005): 3297−3305.
9. A. V. Marenich, C. J. Cramer, and D. G. Truhlar, "Universal Solvation Model Based on Solute Electron Density and on a Continuum Model of the Solvent Defined by the Bulk Dielectric Constant and Atomic Surface Tensions," *The Journal of Physical Chemistry B* 113 (2009): 6378−6396.
10. S. Grimme, "Supramolecular Binding Thermodynamics by Dispersion-Corrected Density Functional Theory," *Chemistry – A European Journal* 18 (2012): 9955−9964.
11. R. L. Martin, P. J. Hay, and L. R. Pratt, "Hydrolysis of Ferric Ion in Water and Conformational Equilibrium," *The Journal of Physical Chemistry A* 102 (1998): 3565−3573.
12. G. Luchini, J. V. Alegre-Requena, Y. Guan, I. Funes-Ardoiz, and R. S. Paton, GoodVibes v3.0.1, 2019. http://doi.org/10.5281/zenodo.595246.
13. G. Luchini, J. V. Alegre-Requena, I. Funes-Ardoiz, and R. S. Paton, "GoodVibes: Automated Thermochemistry for Heterogeneous Computational Chemistry Data," *F1000Research* 9 (2020): 291.
14. A. V. Marenich, S. V. Jerome, C. J. Cramer, and D. G. Truhlar, "Charge Model 5: An Extension of Hirshfeld Population Analysis for the Accurate Description of Molecular Interactions in Gaseous and Condensed Phases," *Journal of Chemical Theory and Computation* 8 (2012): 527−541.
15. Gaussian 16, Revision C.01, M. J. Frisch, G. W. Trucks, H. B. Schlegel, G. E. Scuseria, M. A. Robb, J. R. Cheeseman, G. Scalmani, V. Barone, G. A. Petersson, H. Nakatsuji, X. Li, M. Caricato, A. V. Marenich, J. Bloino, B. G. Janesko, R. Gomperts, B. Mennucci, H. P. Hratchian, J. V. Ortiz, A. F. Izmaylov, J. L. Sonnenberg, D. Williams-Young, F. Ding, F. Lipparini, F. Egidi, J. Goings, B. Peng, A. Petrone, T. Henderson, D. Ranasinghe, V. G. Zakrzewski, J. Gao, N. Rega, G. Zheng, W. Liang, M. Hada, M. Ehara, K. Toyota, R. Fukuda, J. Hasegawa, M. Ishida, T. Nakajima, Y. Honda, O. Kitao, H. Nakai, T. Vreven, K. Throssell, J. A. Jr. Montgomery, J. E. Peralta, F. Ogliaro, M. J. Bearpark, J. J. Heyd, E. N. Brothers, K. N. Kudin, V. N. Staroverov, T. A. Keith, R. Kobayashi, J. Normand, K. Raghavachari, A. P. Rendell, J. C. Burant, S. S. Iyengar, J. Tomasi, M. Cossi, J. M. Millam, M. Klene, C. Adamo, R. Cammi, J. W. Ochterski, R. L. Martin, K. Morokuma, O. Farkas, J. B. Foresman, and Fox, D. J., Gaussian, Inc., Wallingford CT, 2016.

Copies of the NMR spectra

^{1}H NMR spectrum of **5a** (400 MHz, $CDCl_3$)

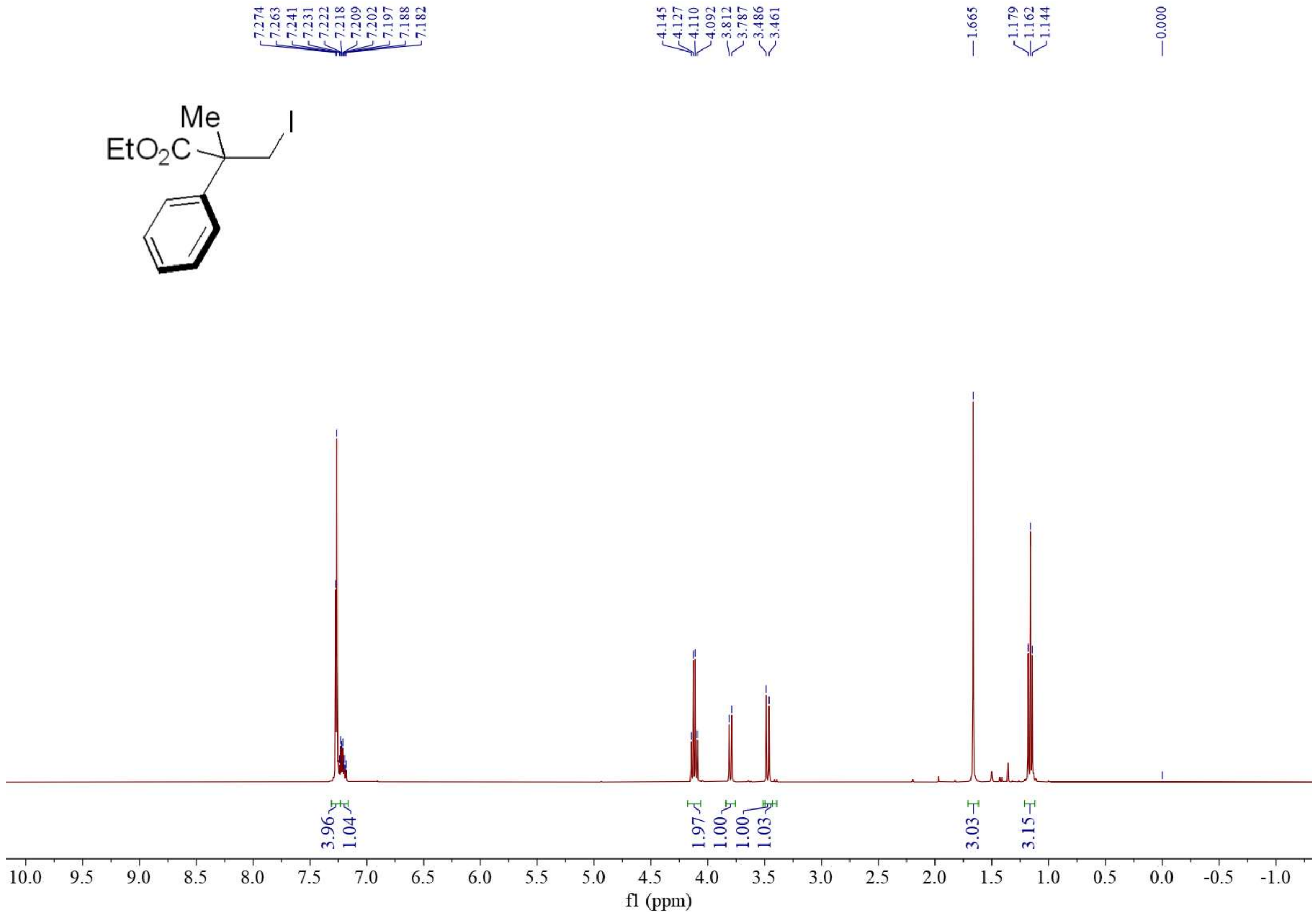

^{13}C NMR spectrum of **5a** (101 MHz, $CDCl_3$)

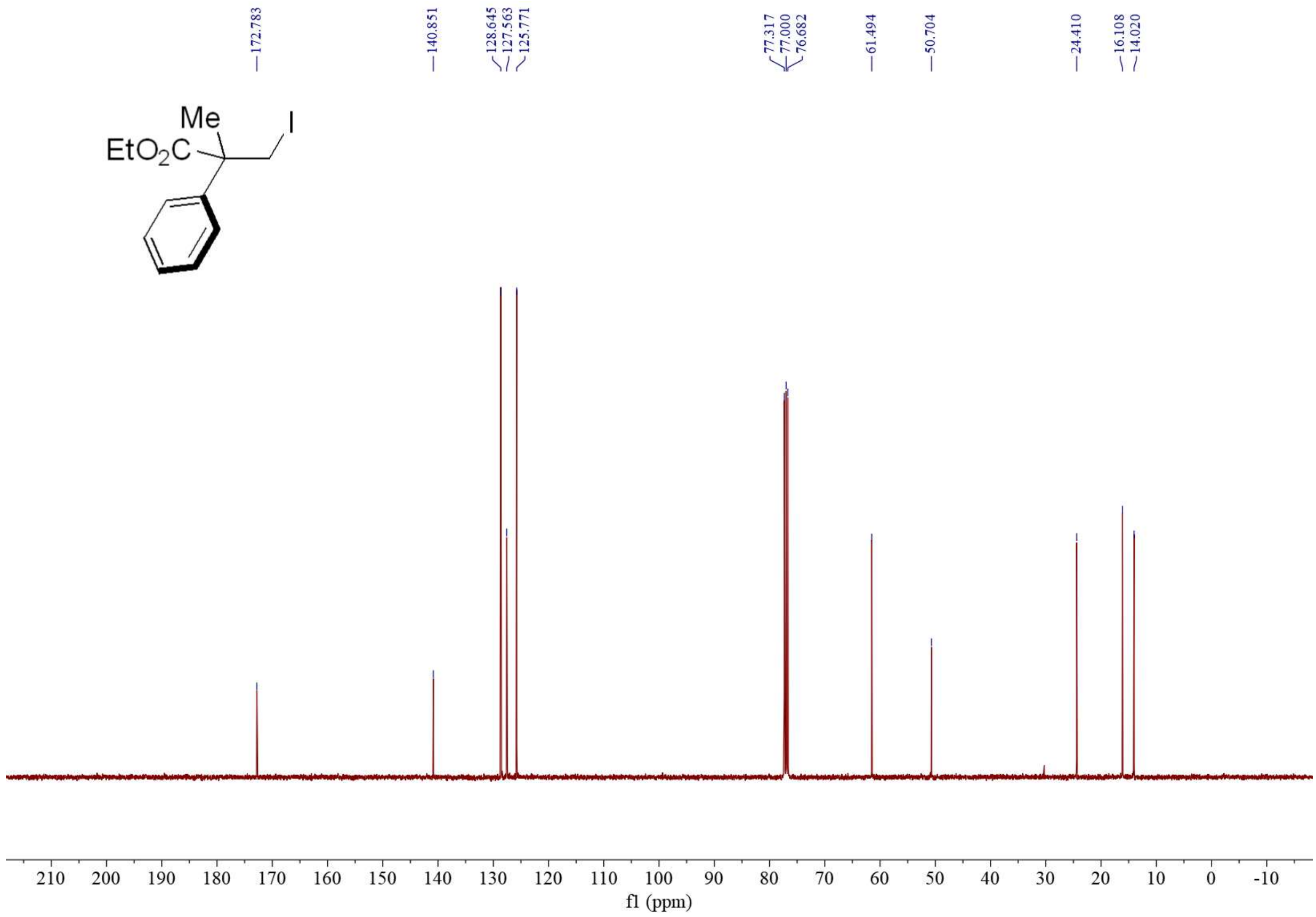

^{1}H NMR spectrum of **5b** (800 MHz, $CDCl_3$)

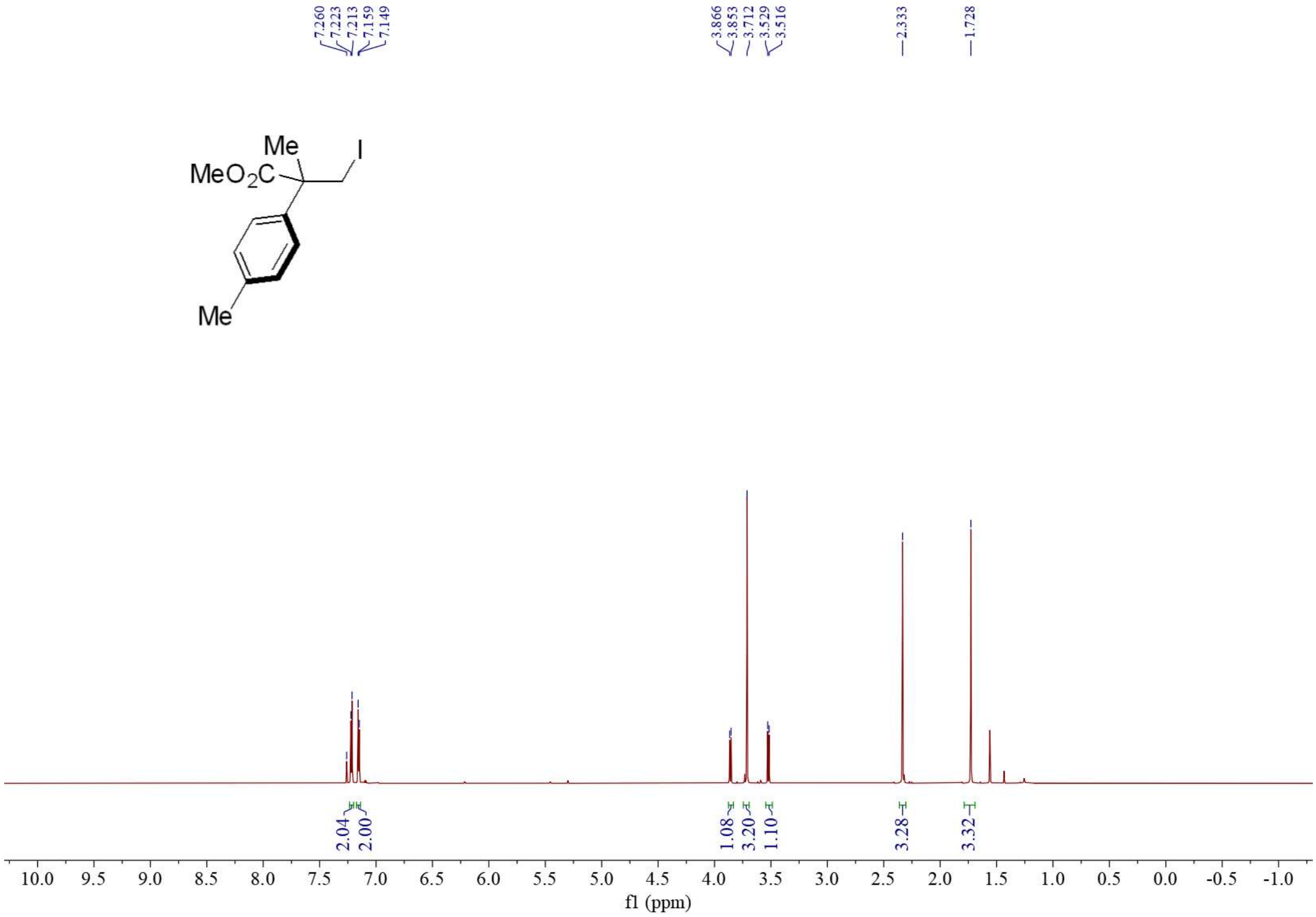

^{13}C NMR spectrum of **5b** (201 MHz, $CDCl_3$)

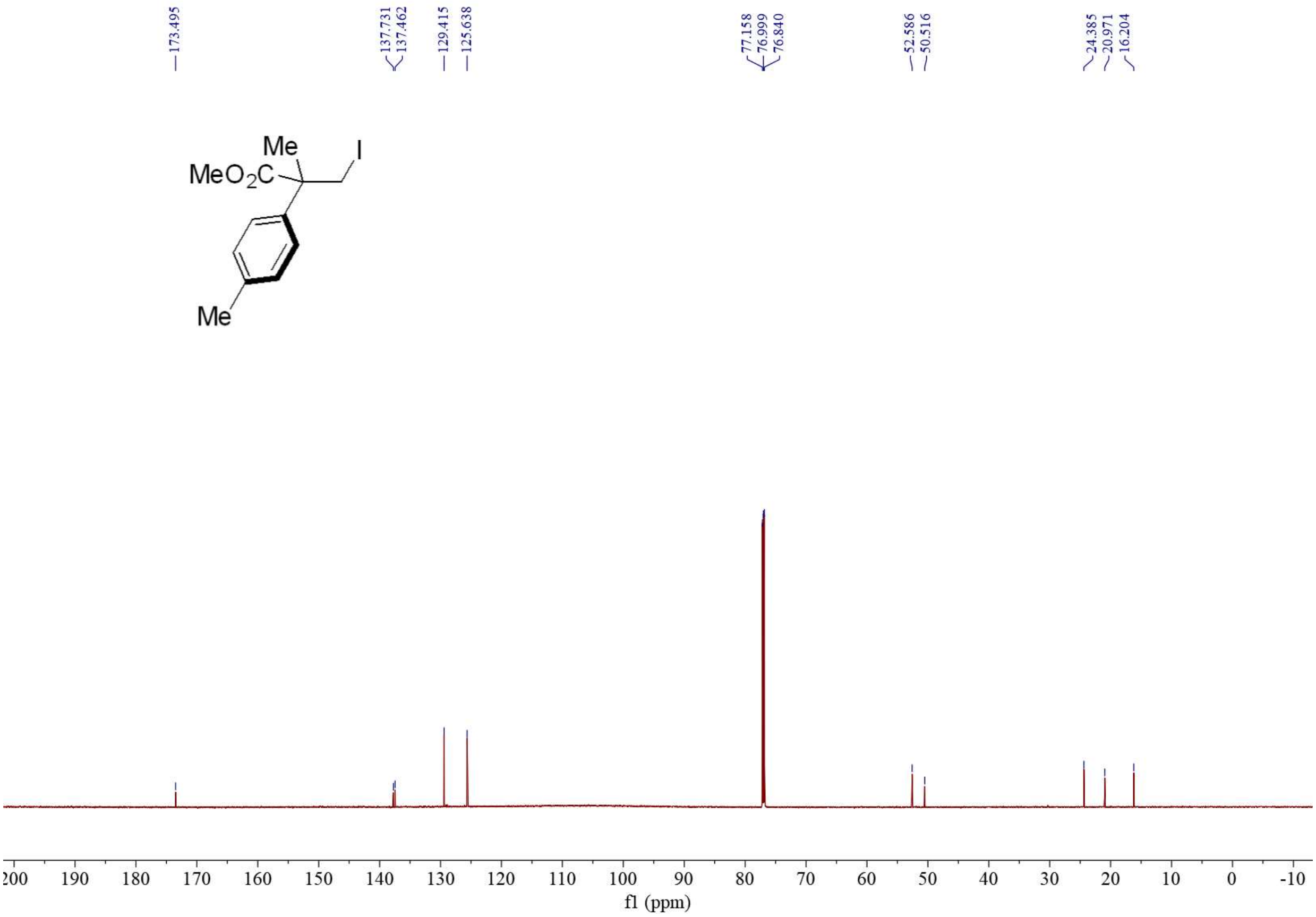

^{1}H NMR spectrum of **5c** (400 MHz, $CDCl_3$)

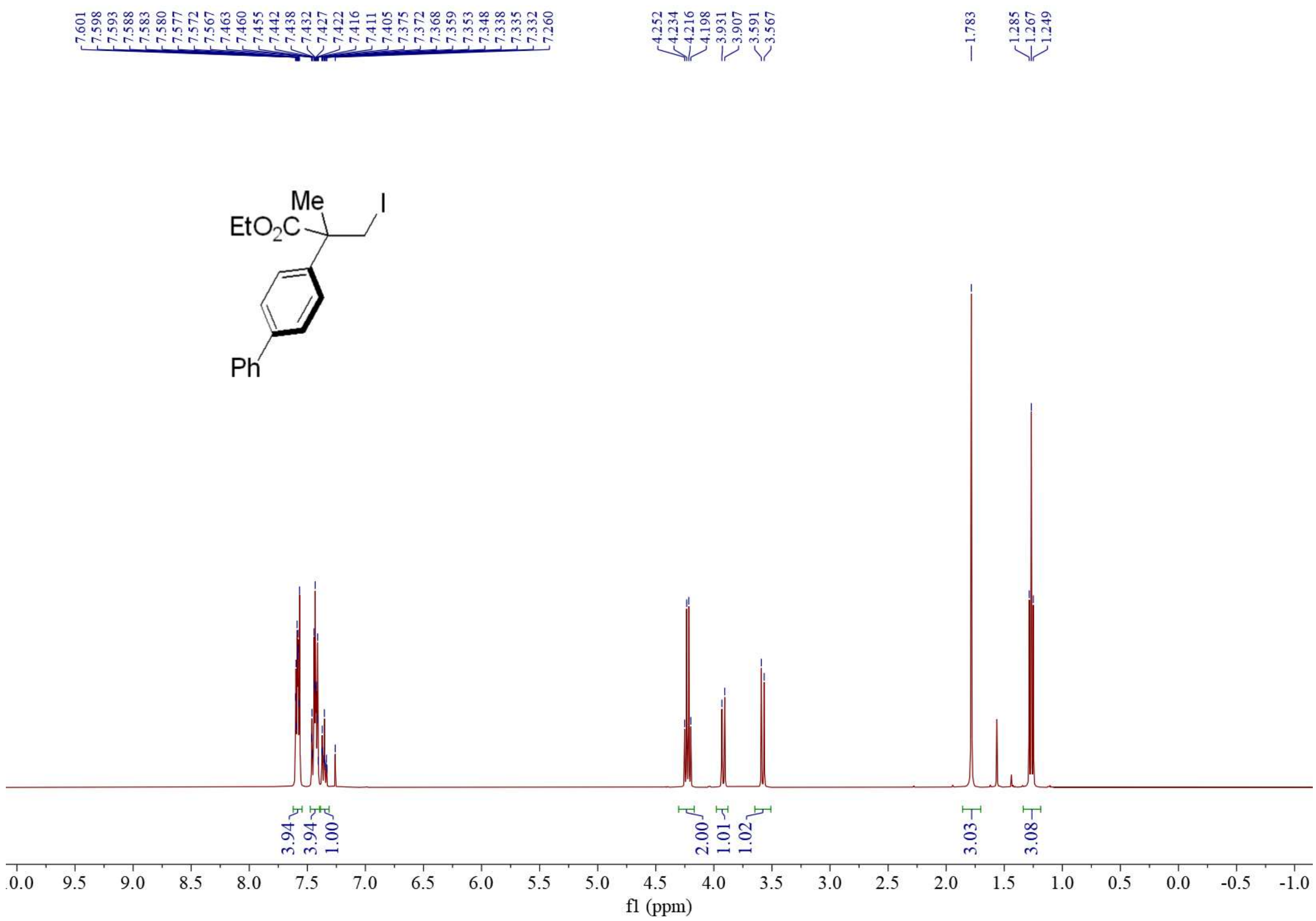

^{13}C NMR spectrum of **5c** (101 MHz, $CDCl_3$)

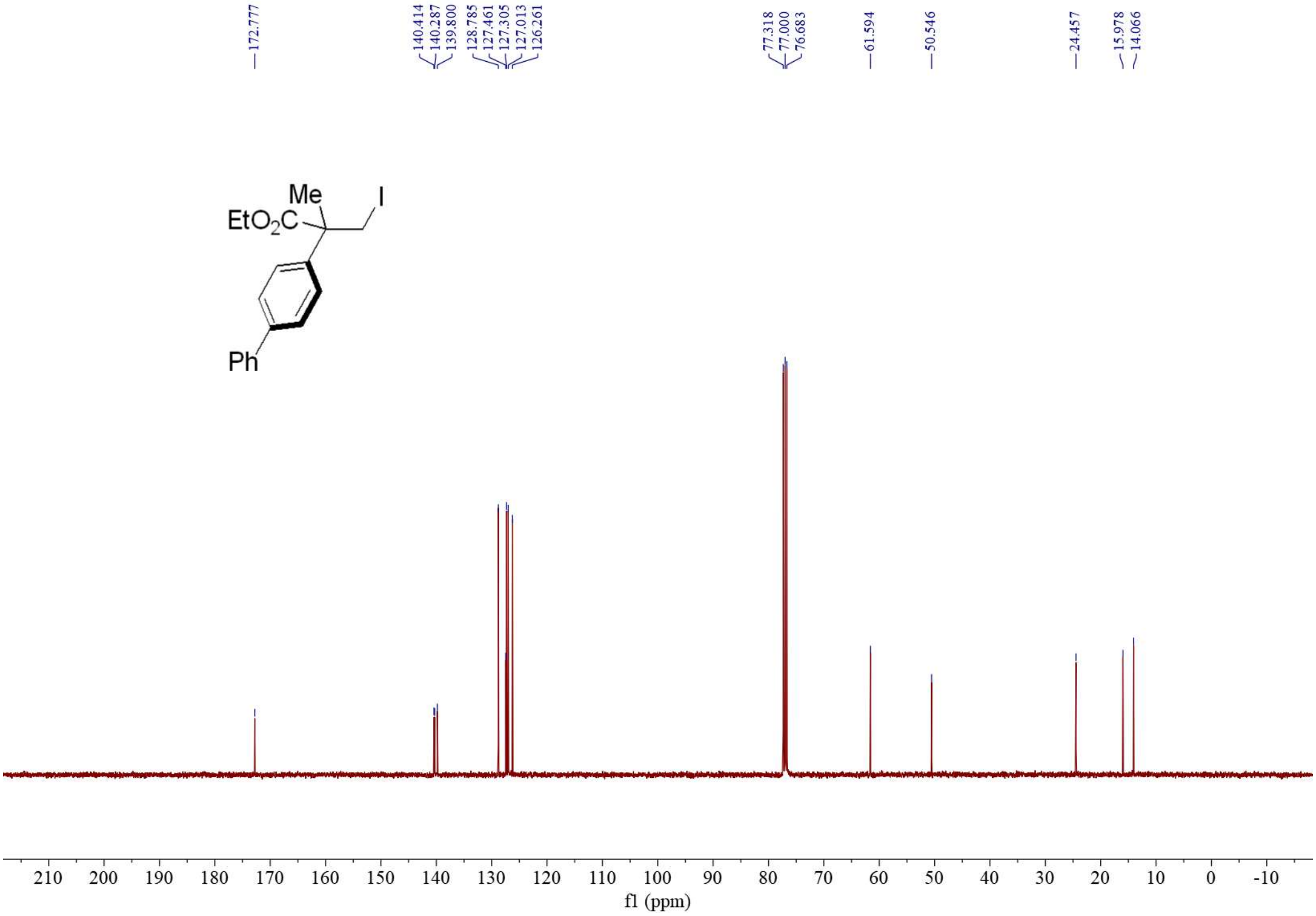

^{1}H NMR spectrum of **5d** (400 MHz, $CDCl_3$)

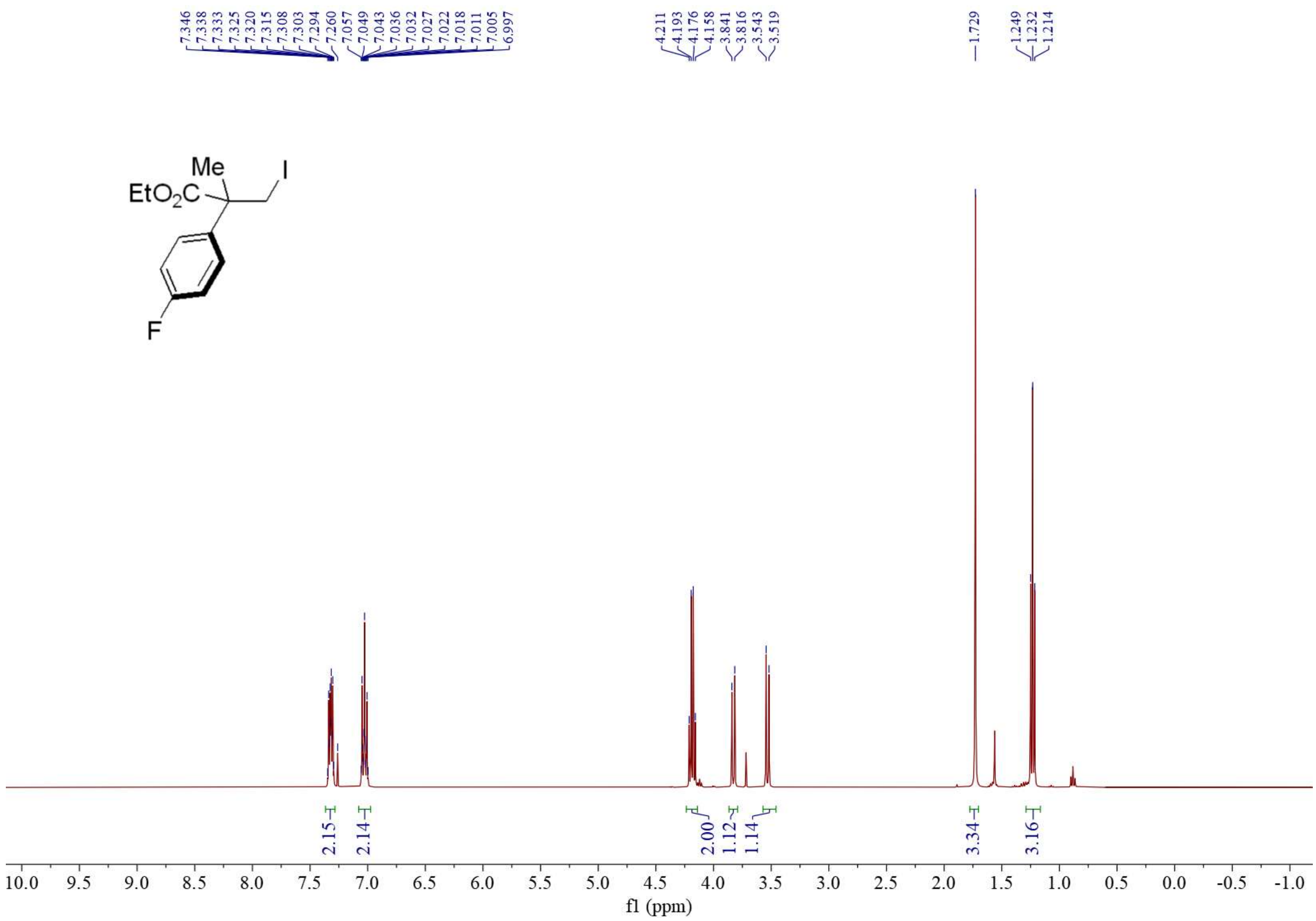

^{13}C NMR spectrum of **5d** (101 MHz, $CDCl_3$)

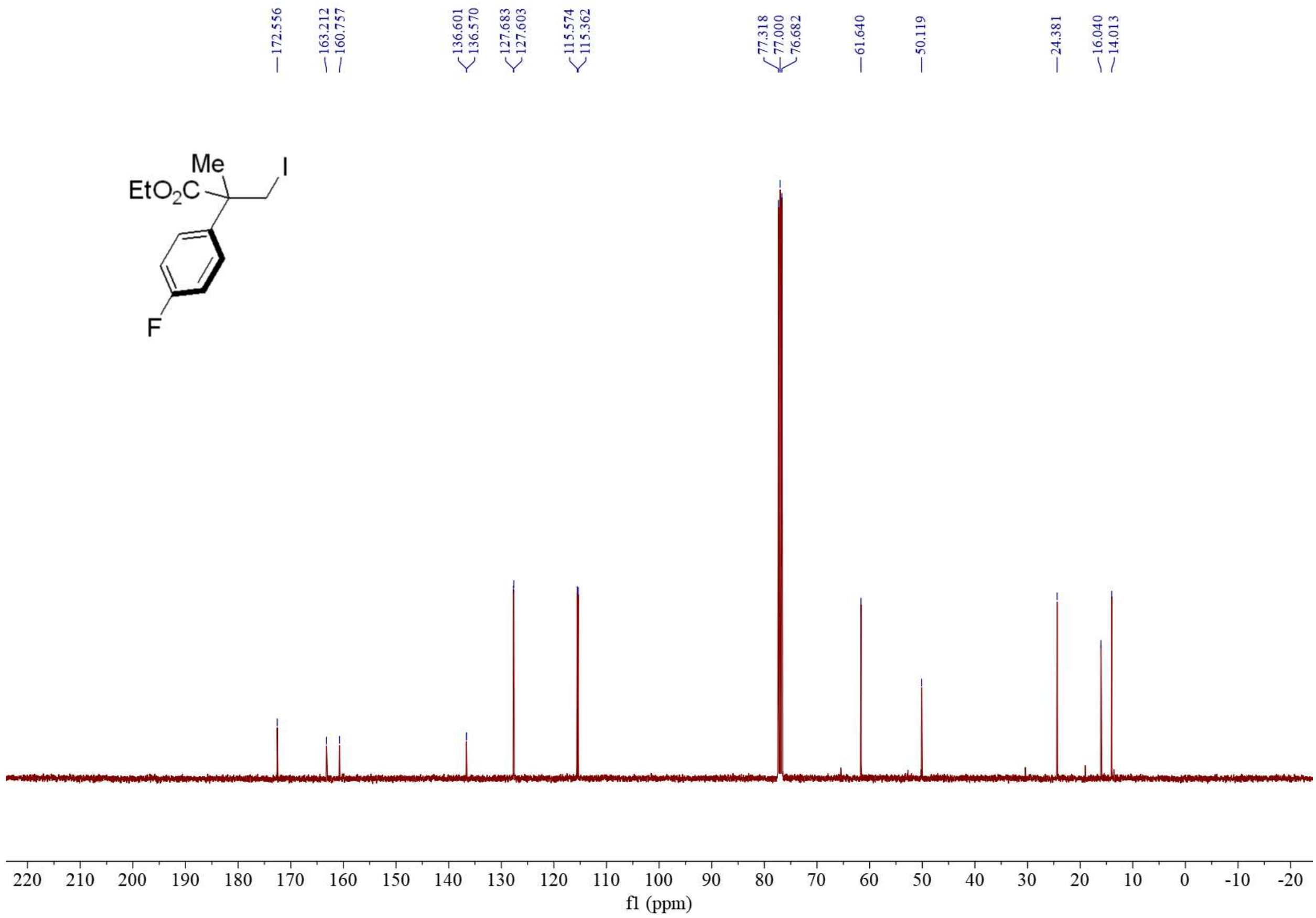

$^{19}F\{^1H\}$ NMR spectrum of **5d** (377 MHz, $CDCl_3$)

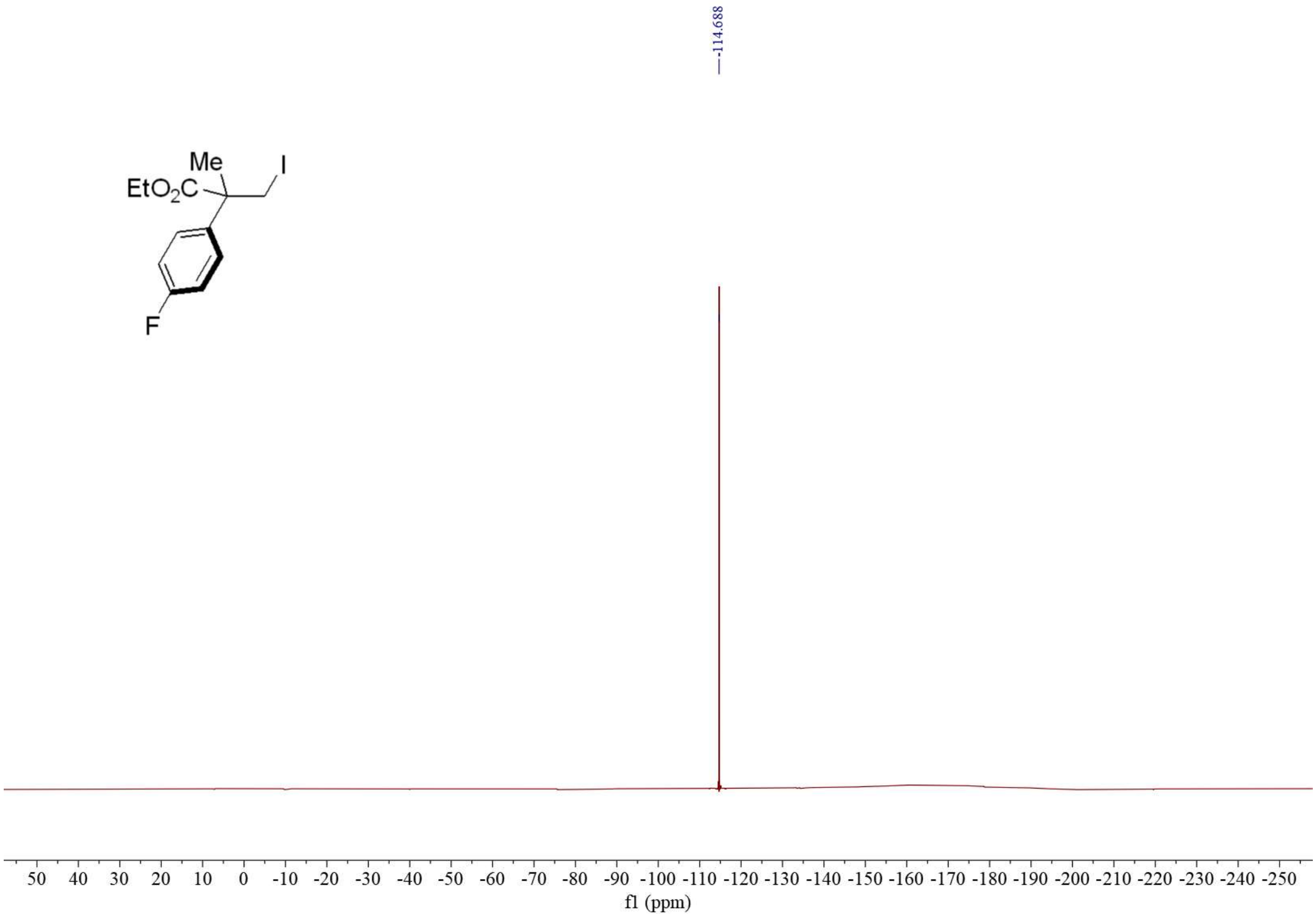

^{19}F NMR spectrum of **5d** (377 MHz, $CDCl_3$)

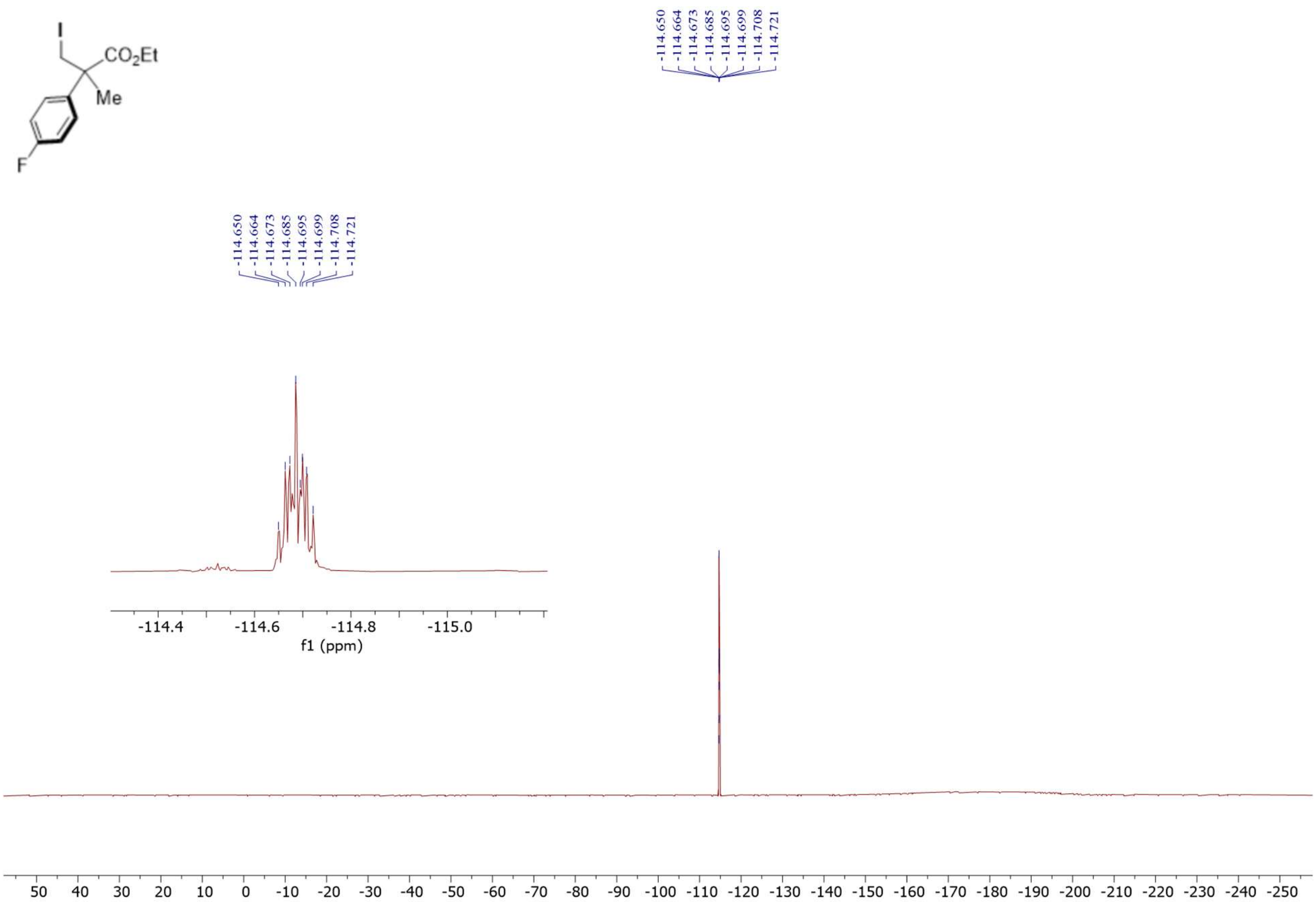

^{1}H NMR spectrum of **5e** (400 MHz, $CDCl_3$)

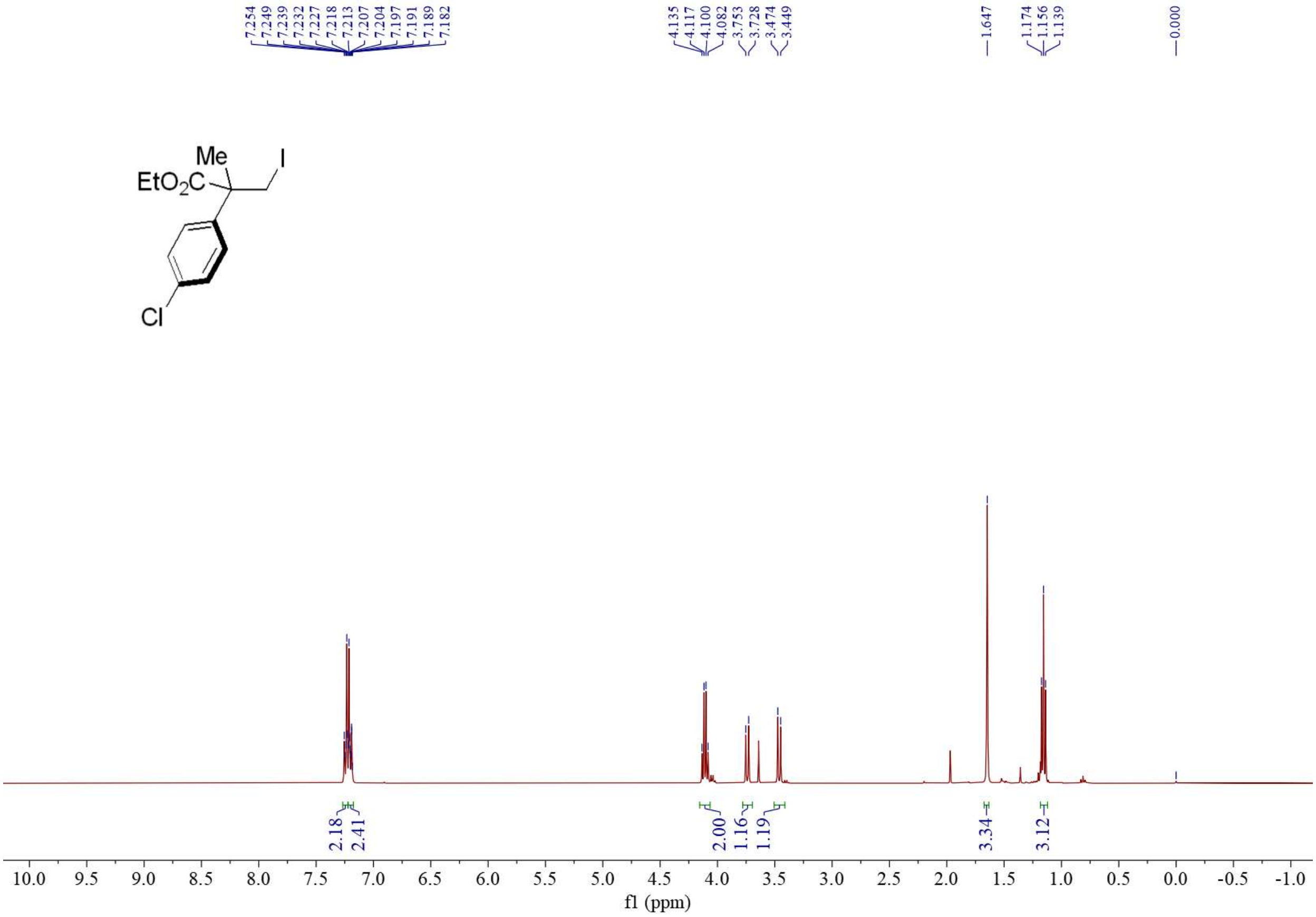

^{13}C NMR spectrum of **5e** (101 MHz, $CDCl_3$)

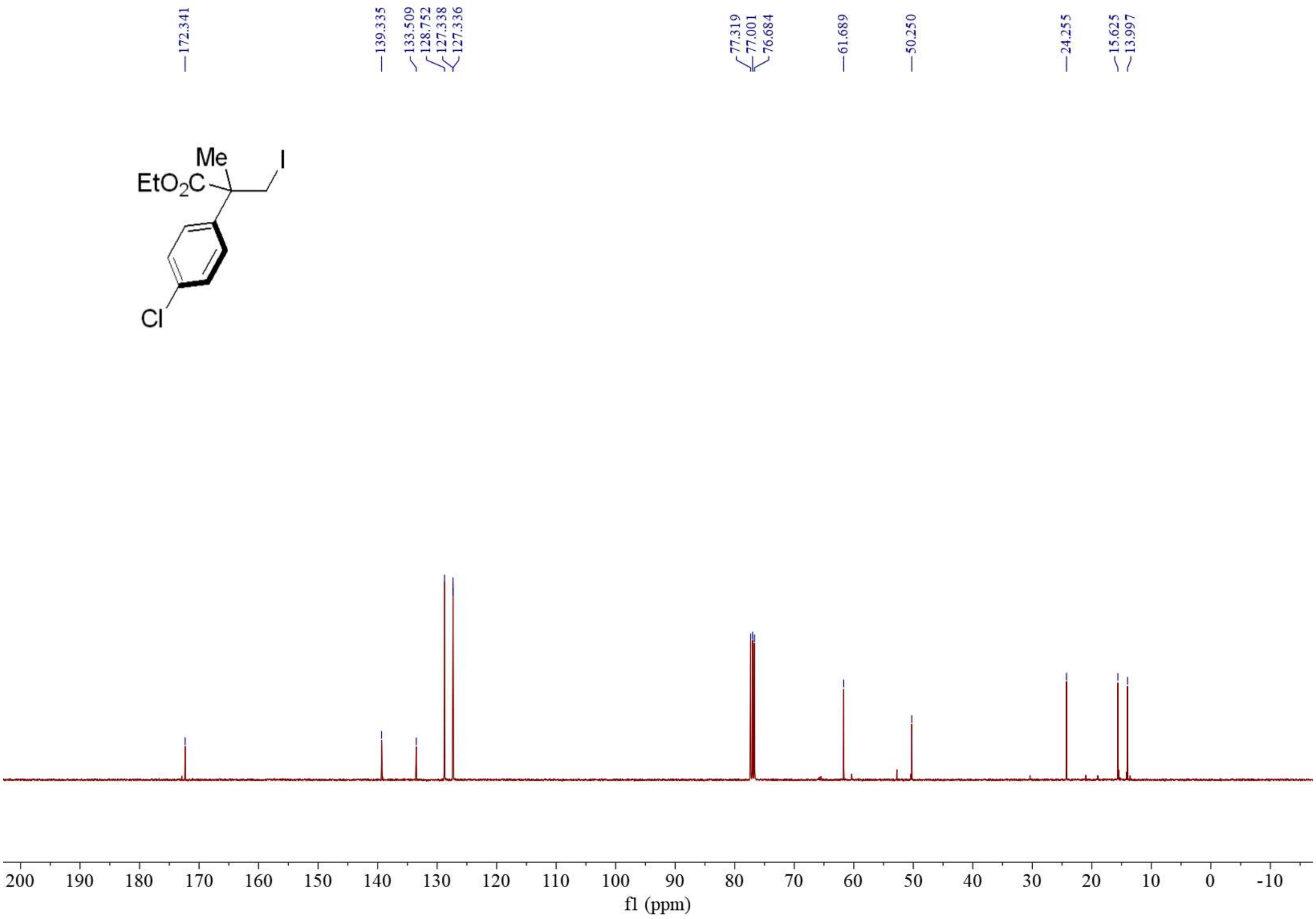

^{1}H NMR spectrum of **5f** (400 MHz, $CDCl_3$)

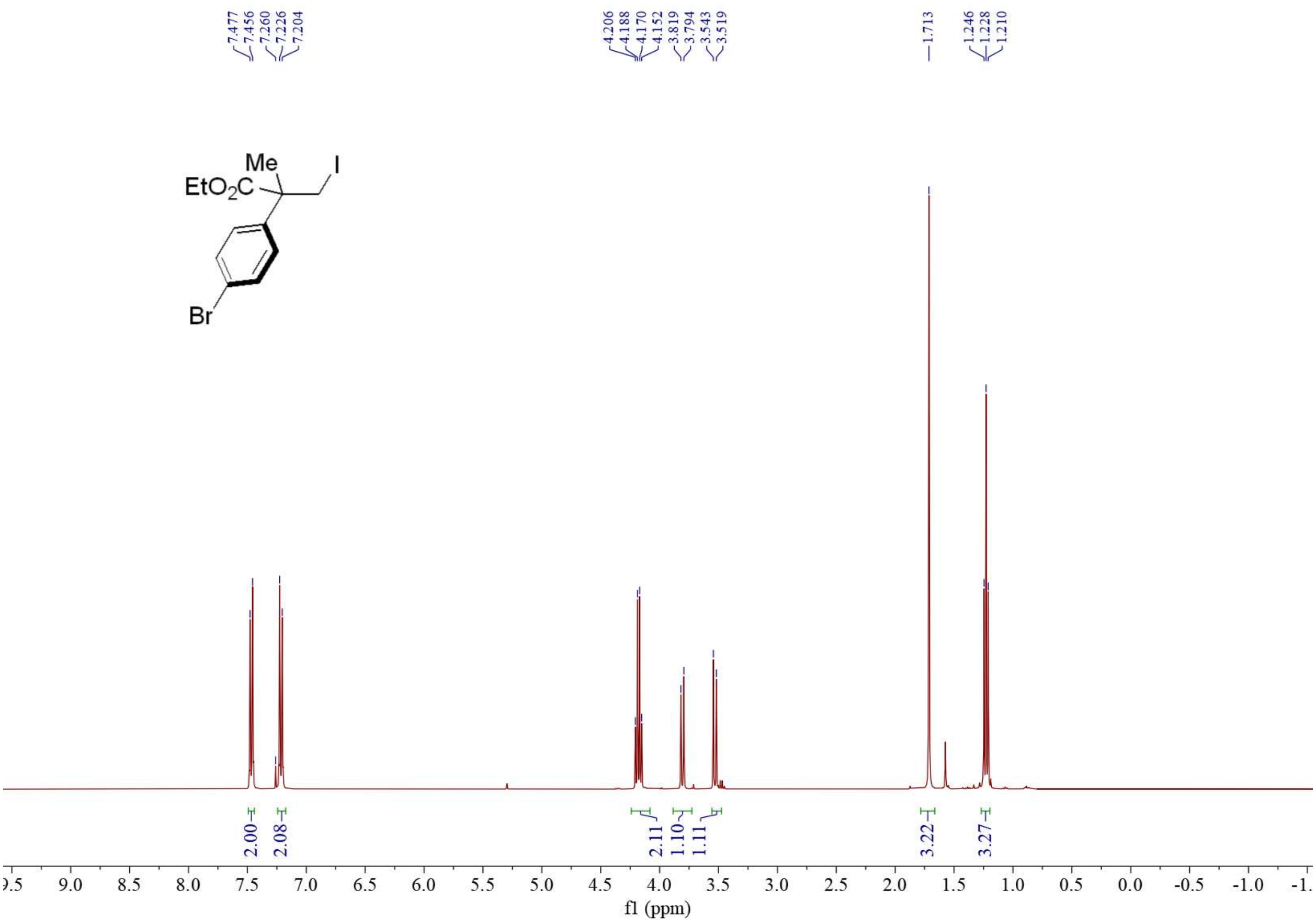

^{13}C NMR spectrum of **5f** (101 MHz, $CDCl_3$)

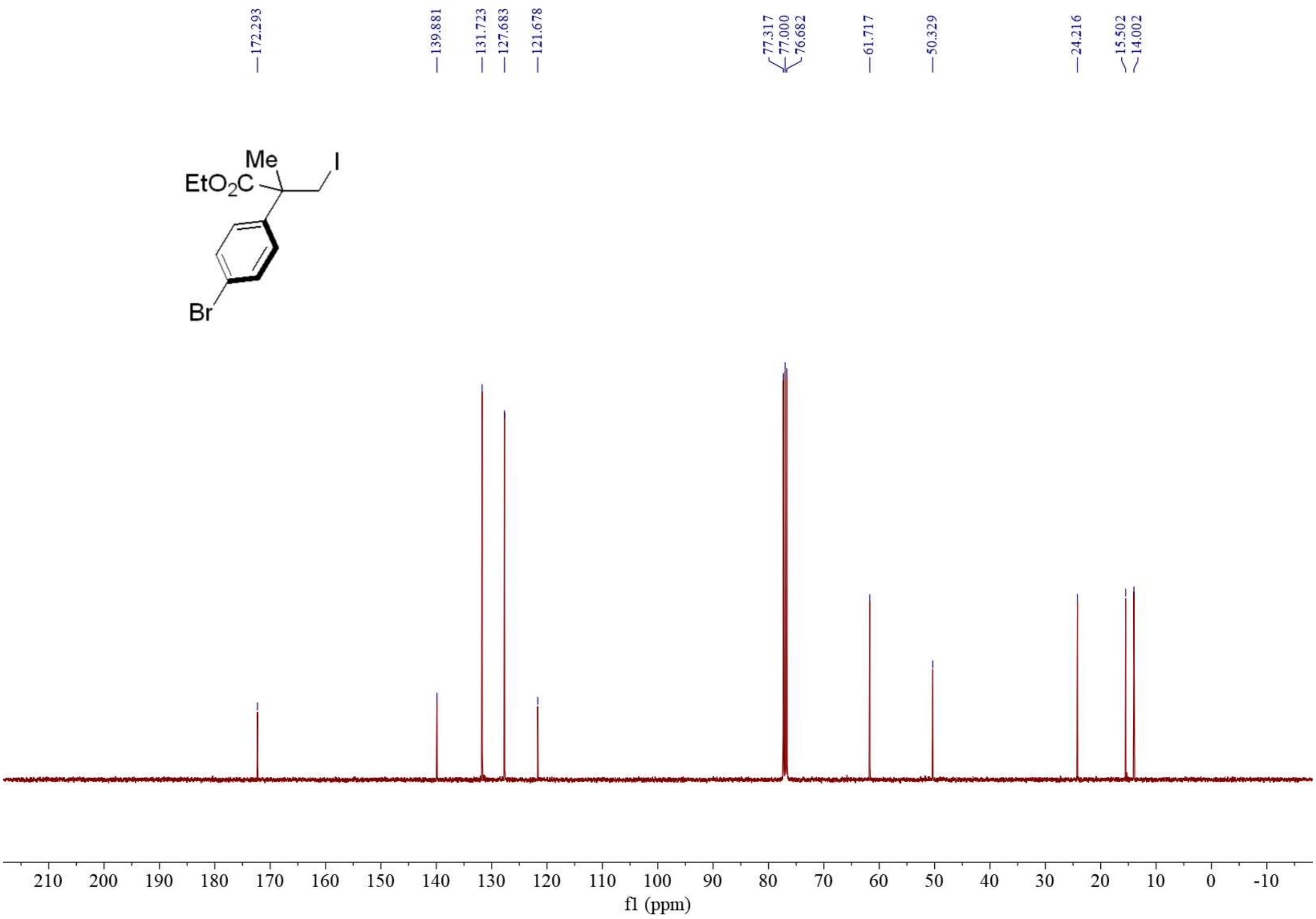

^{1}H NMR spectrum of **5g** (400 MHz, $CDCl_3$)

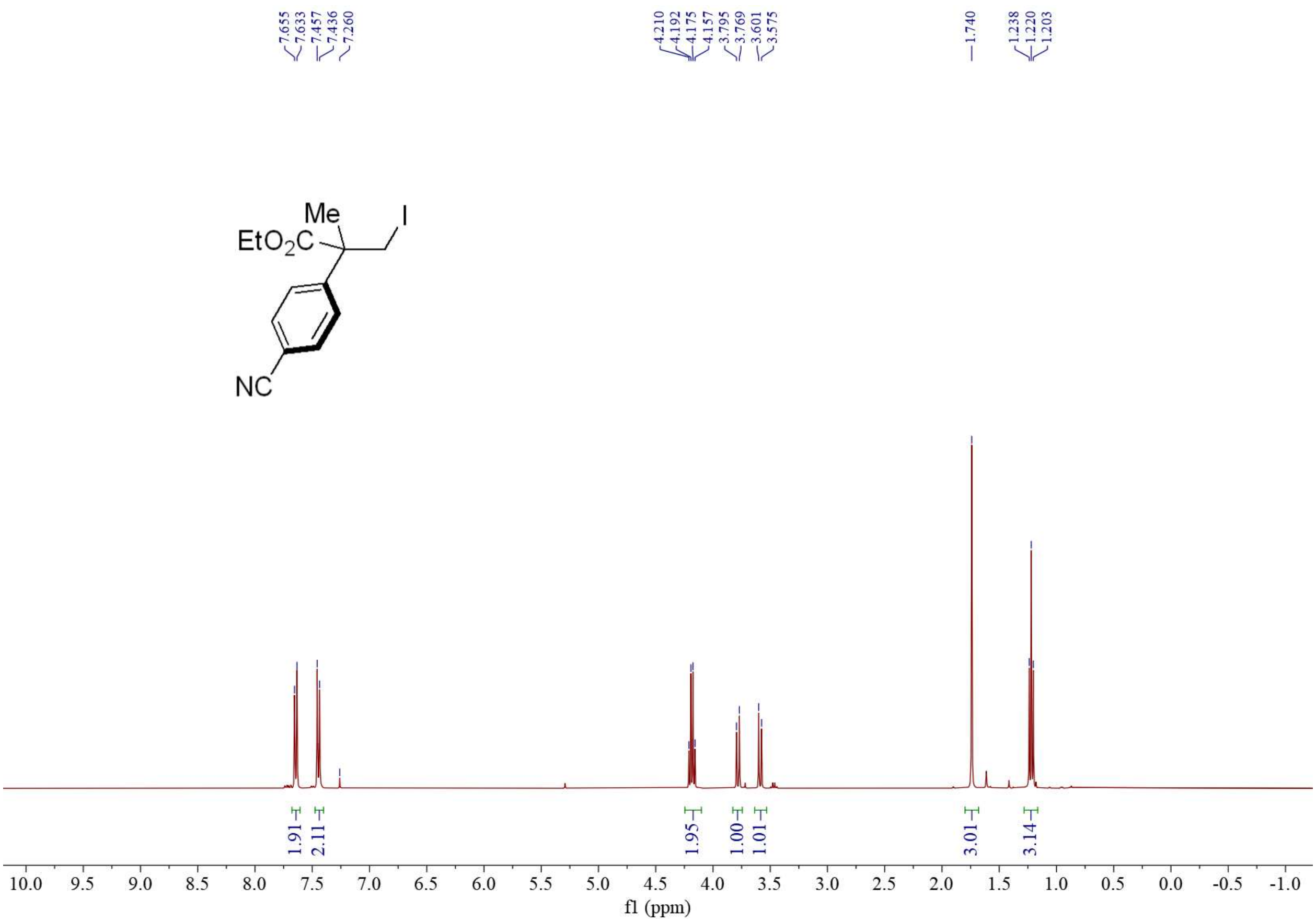

^{13}C NMR spectrum of **5g** (101 MHz, $CDCl_3$)

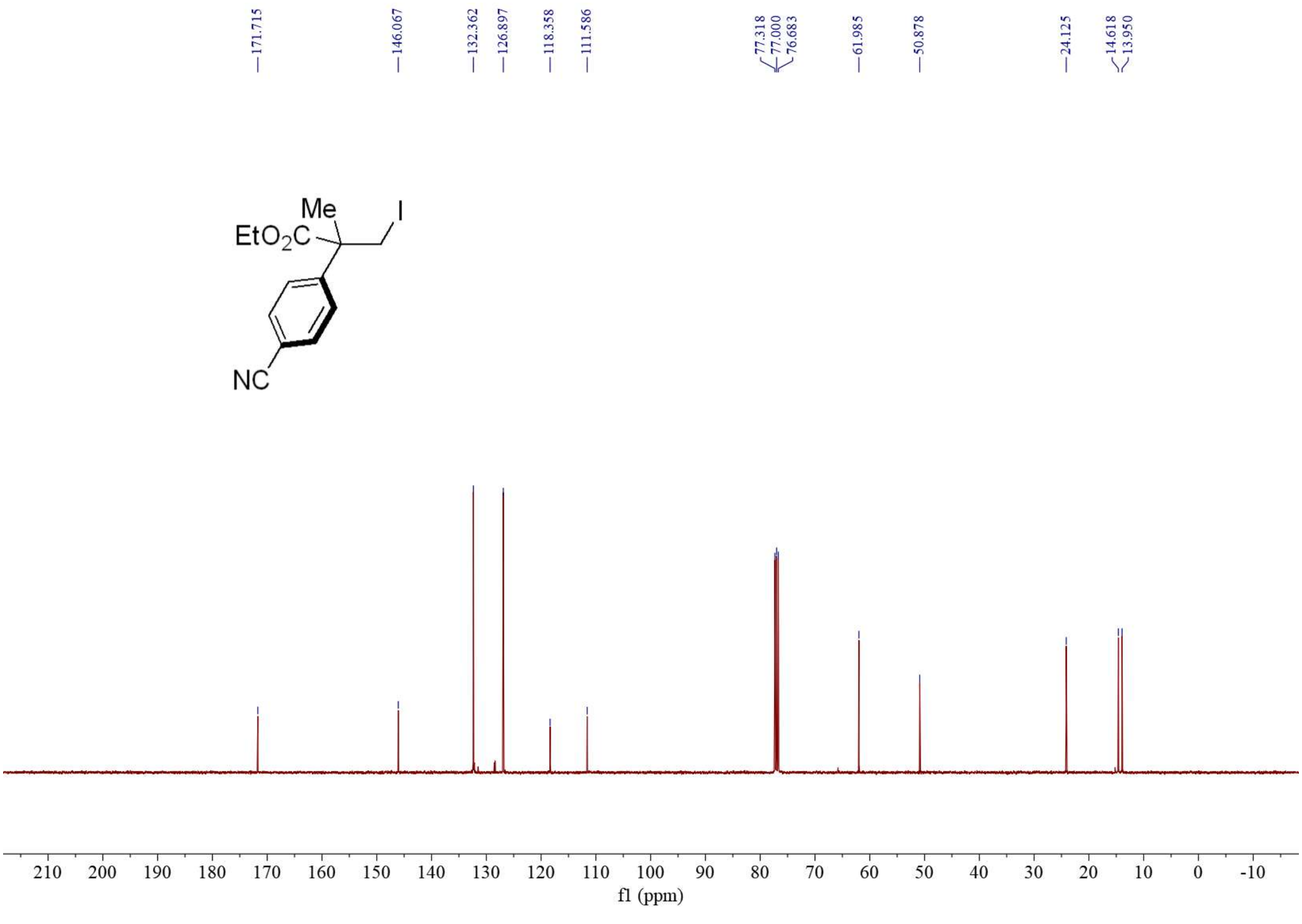

^{1}H NMR spectrum of **5h** (400 MHz, $CDCl_3$)

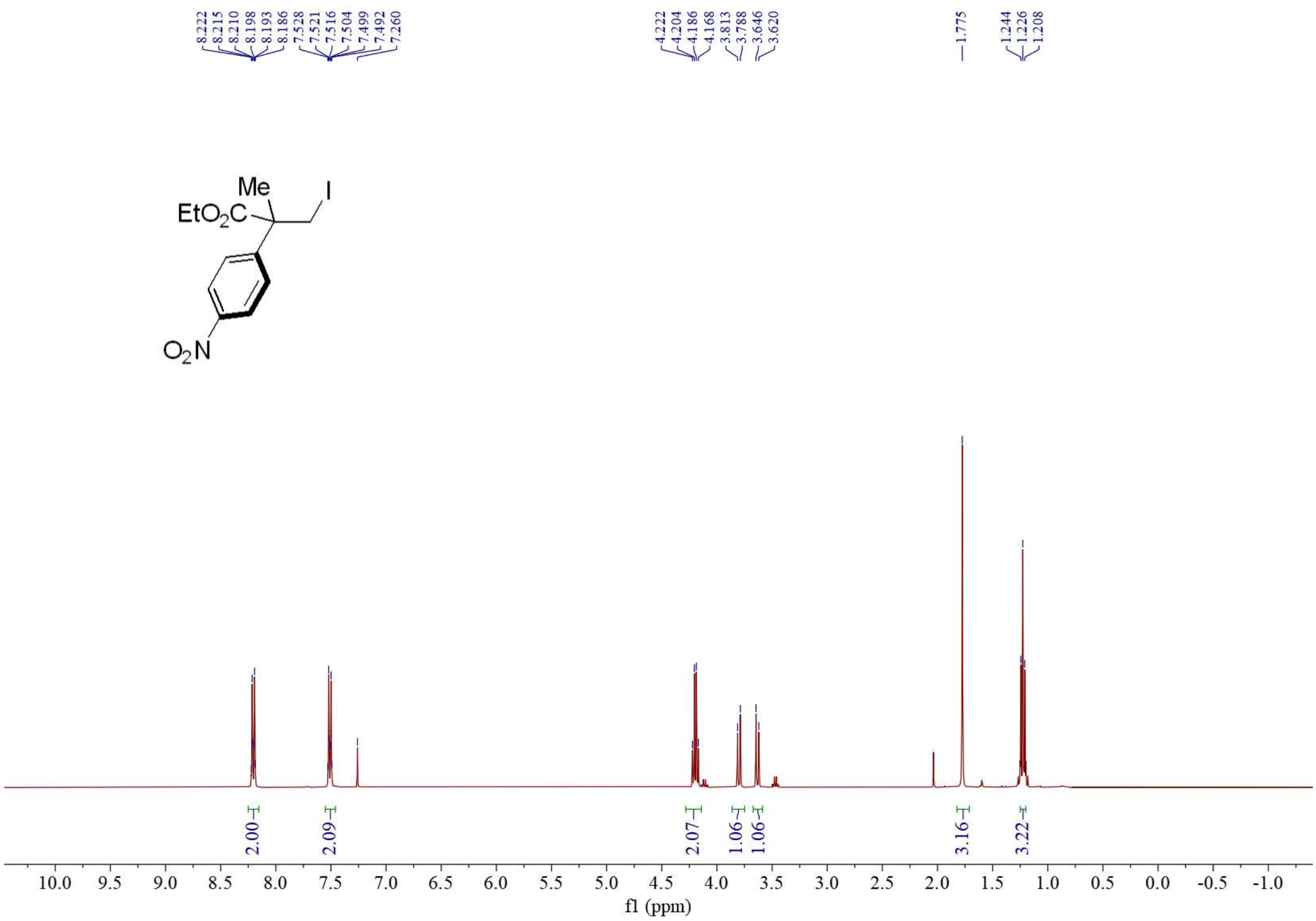

^{13}C NMR spectrum of **5h** (101 MHz, $CDCl_3$)

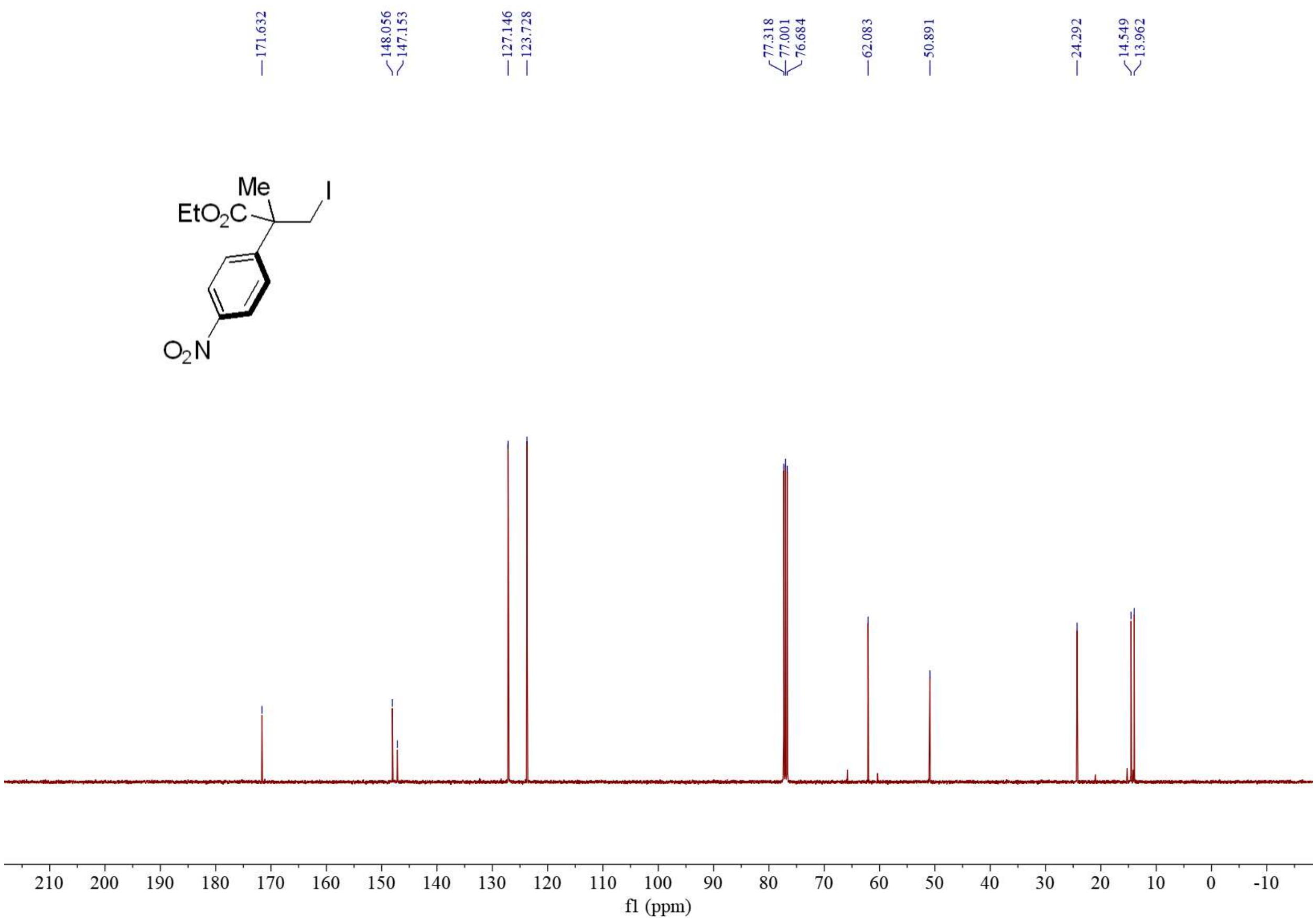

^{1}H NMR spectrum of **5i** (400 MHz, $CDCl_3$)

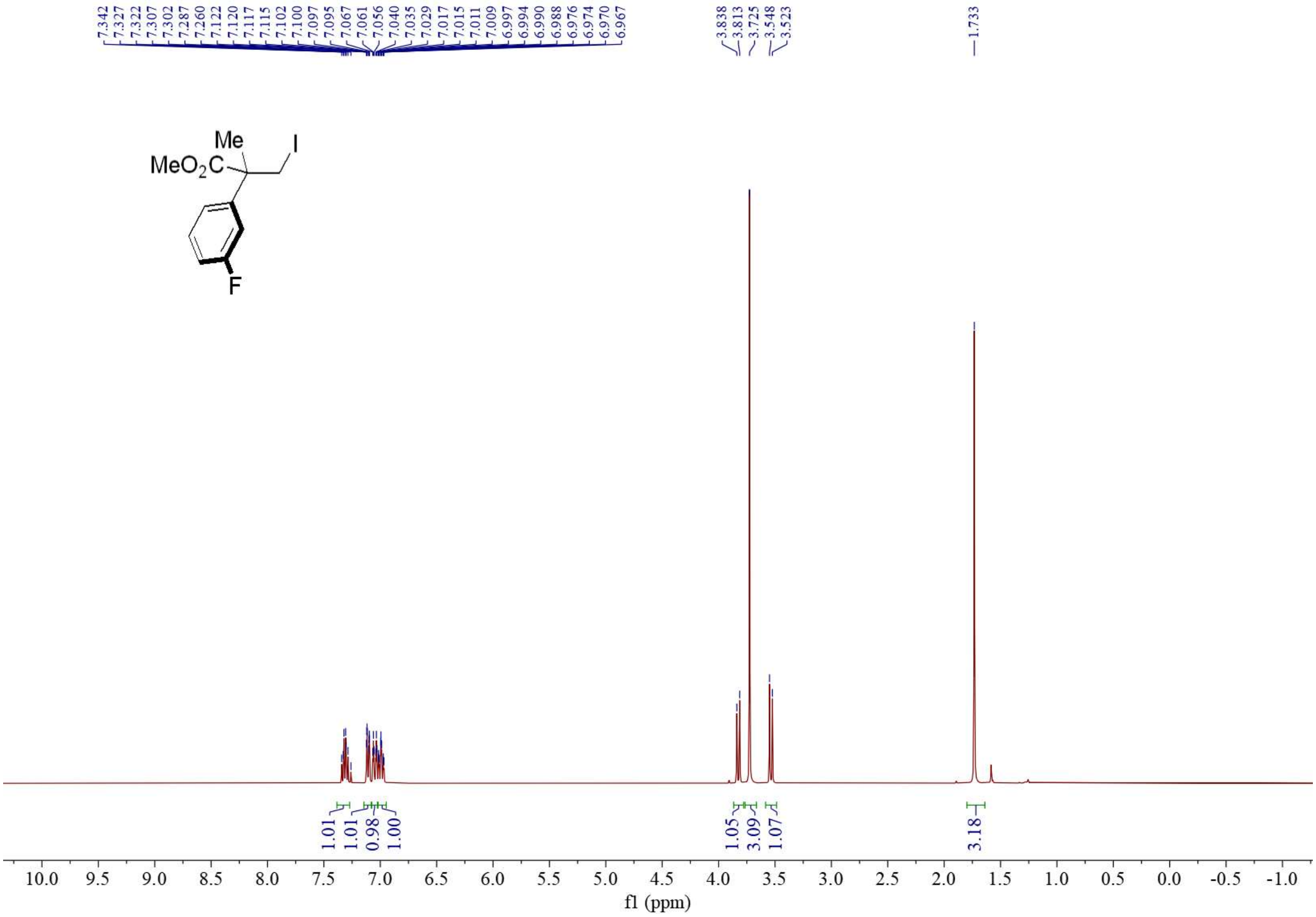

^{13}C NMR spectrum of **5i** (101 MHz, $CDCl_3$)

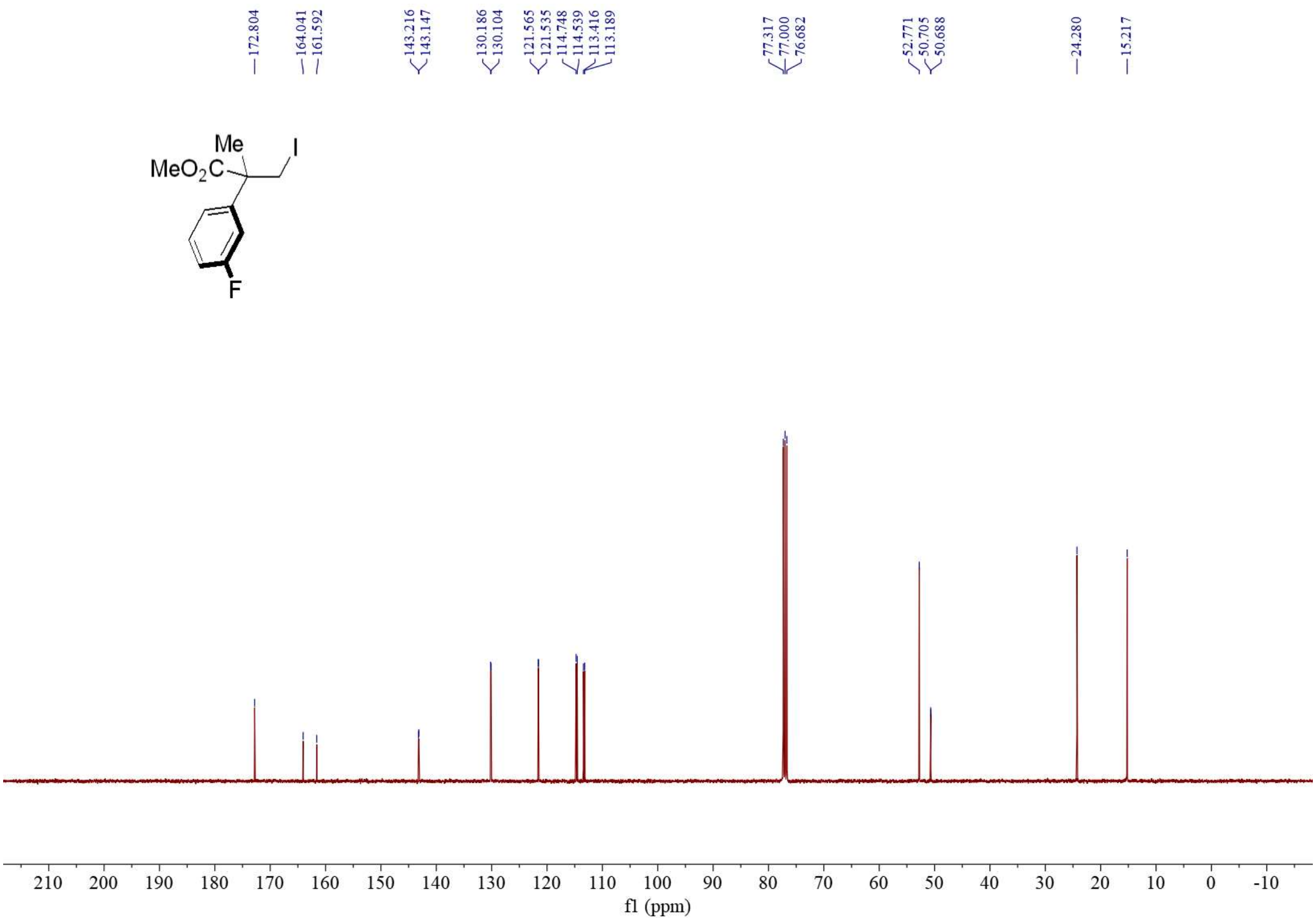

$^{19}F\{^{1}H\}$ NMR spectrum of **5i** (377 MHz, $CDCl_3$)

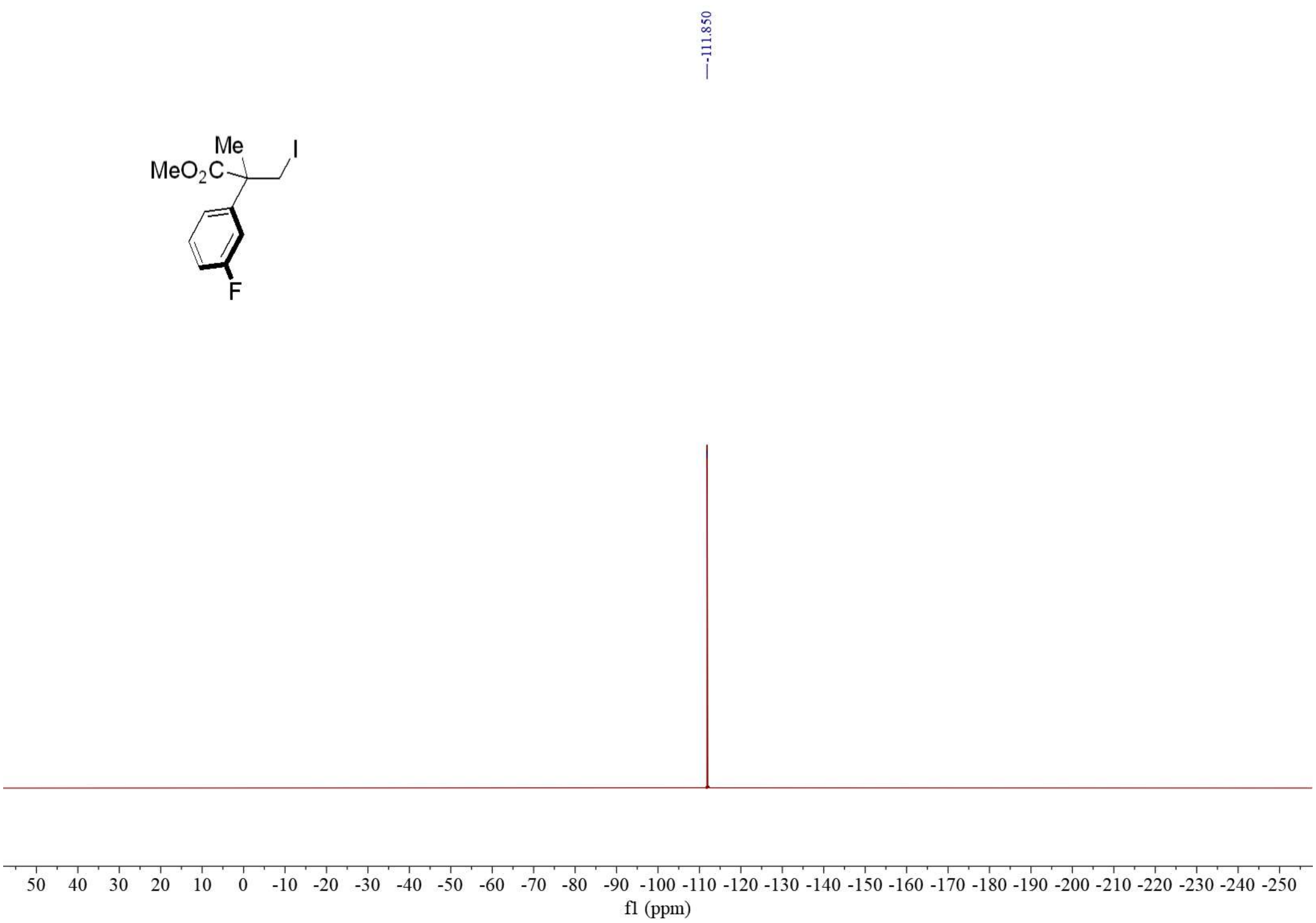

^{19}F NMR spectrum of **5i** (377 MHz, $CDCl_3$)

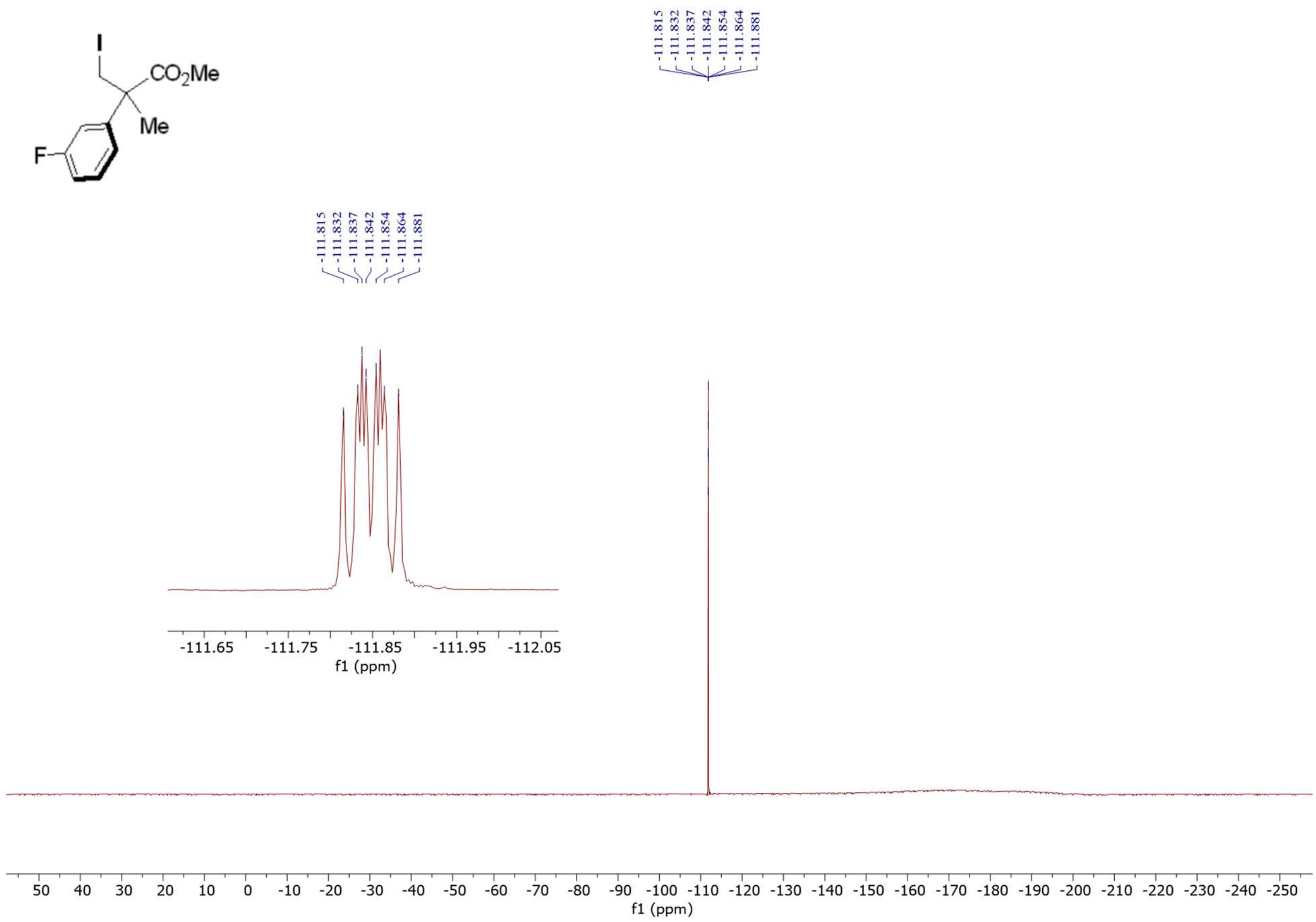

^{1}H NMR spectrum of **5j** (400 MHz, $CDCl_3$)

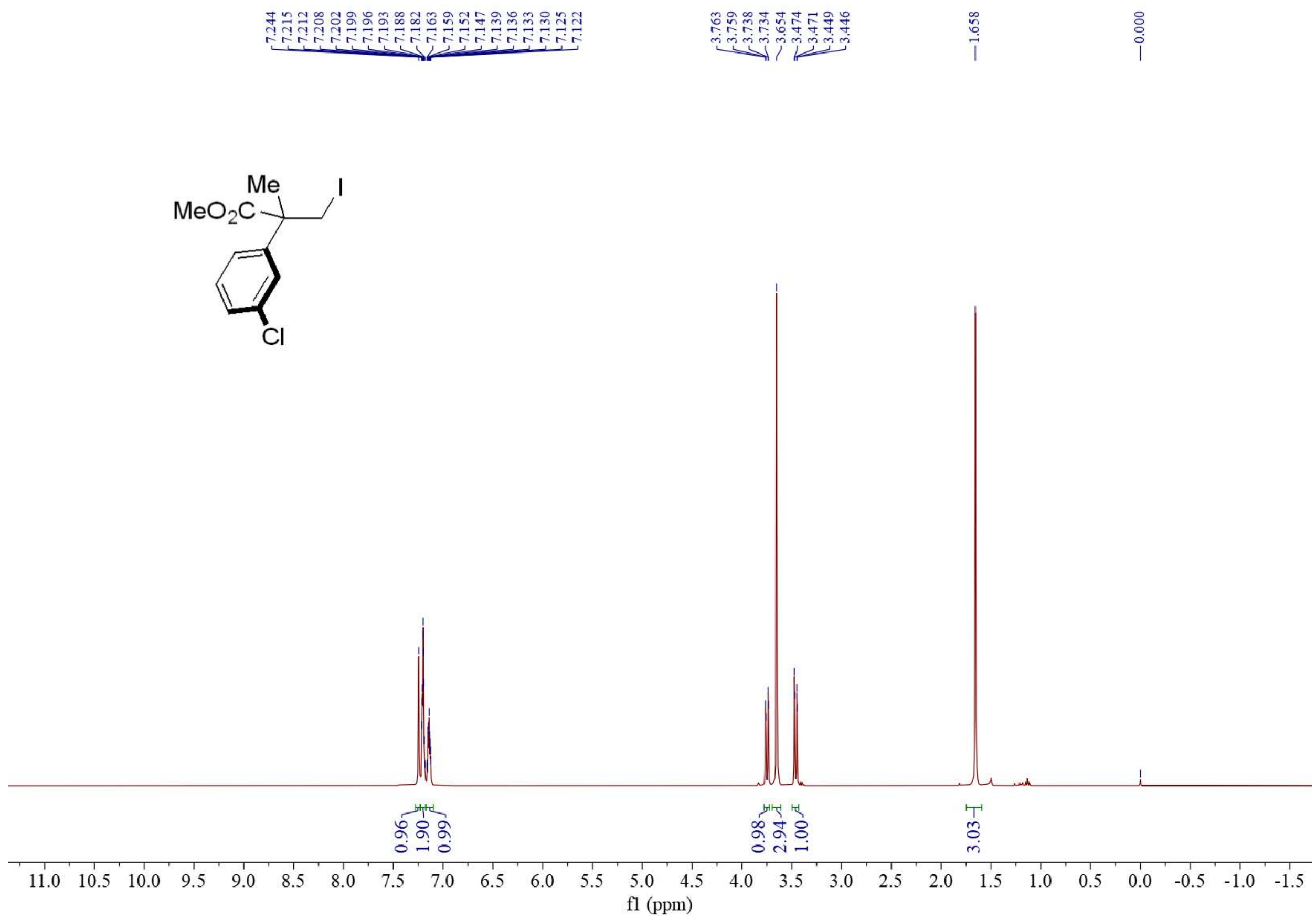

^{13}C NMR spectrum of **5j** (101 MHz, $CDCl_3$)

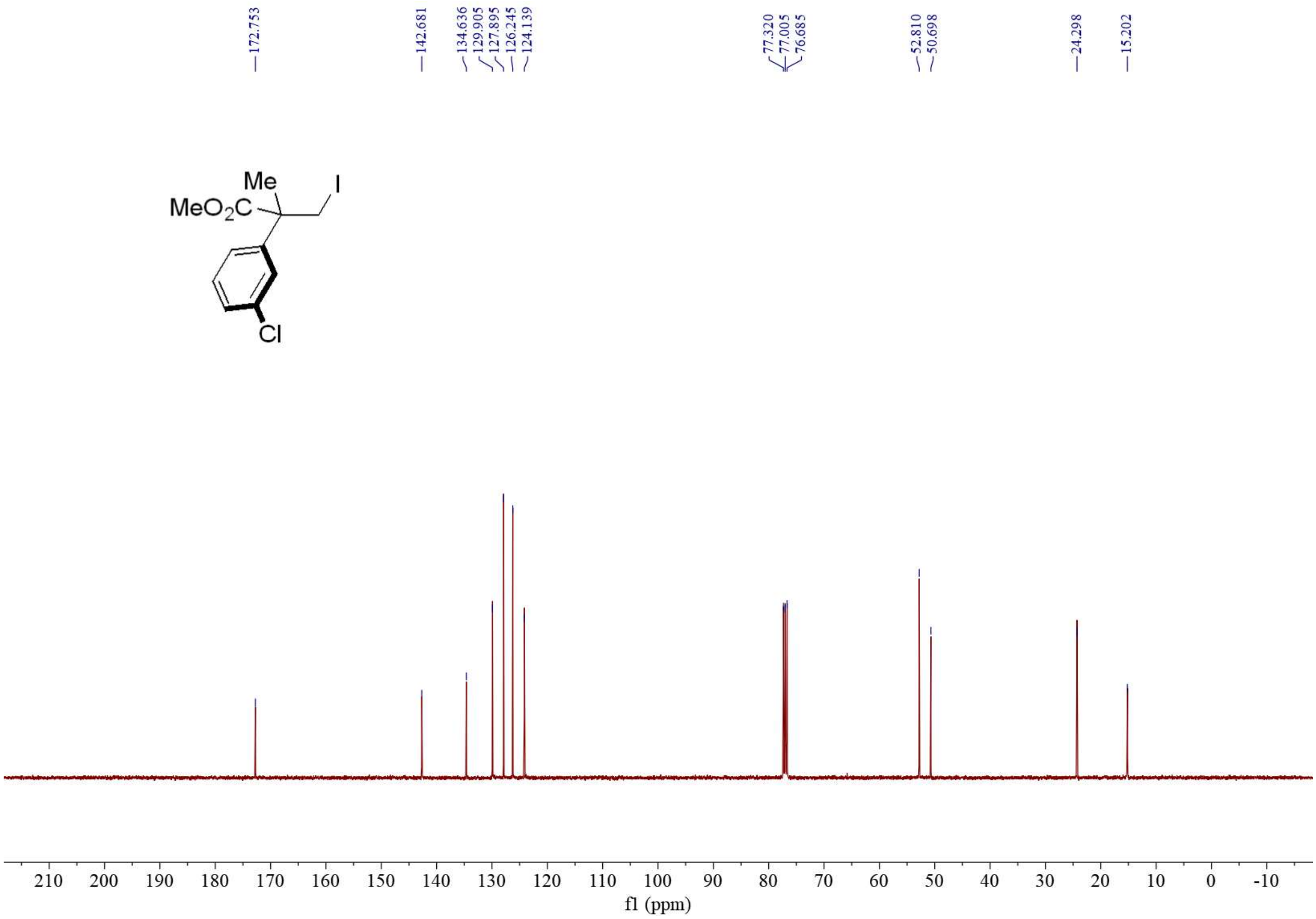

^{1}H NMR spectrum of **5k** (400 MHz, $CDCl_3$)

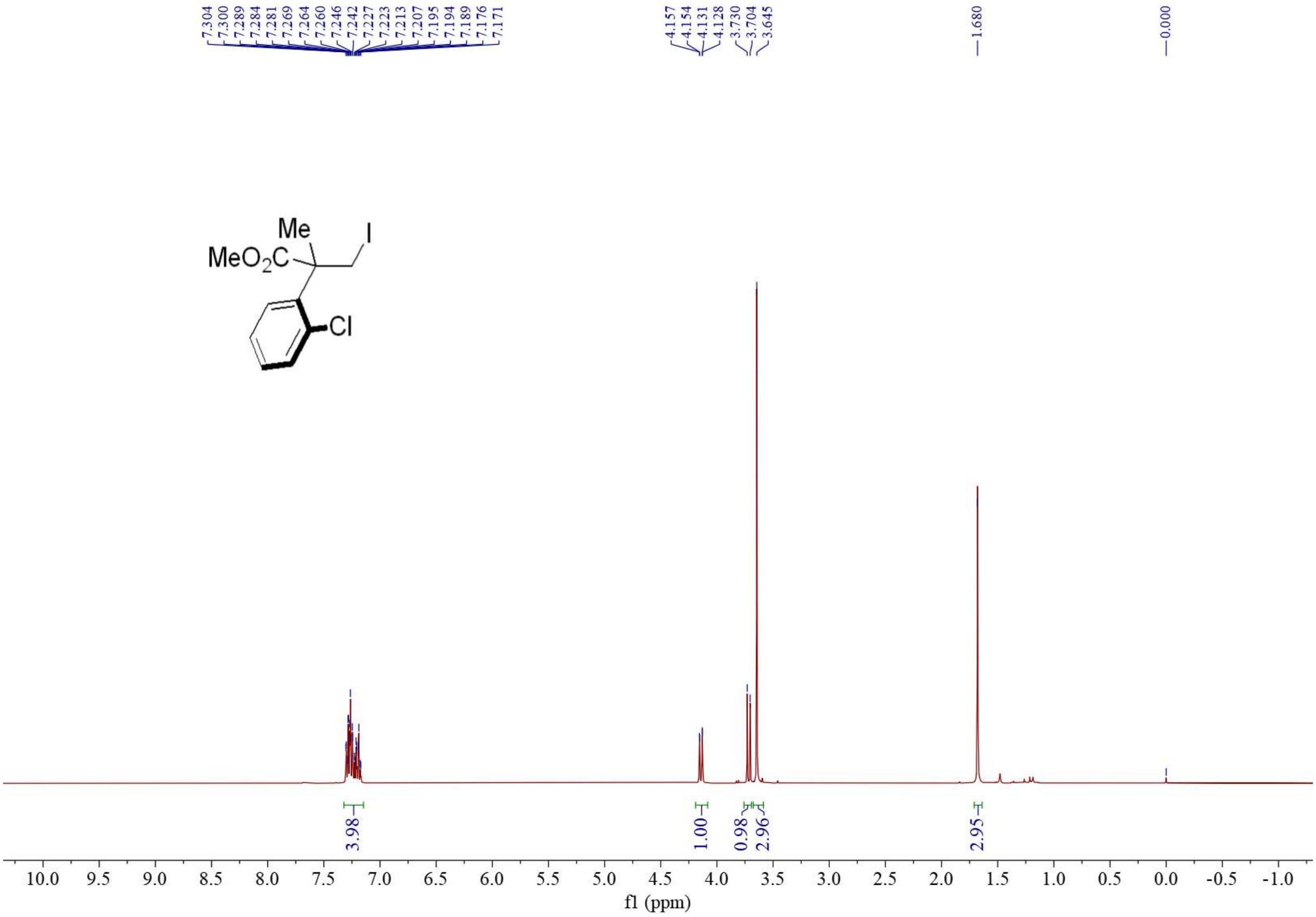

^{13}C NMR spectrum of **5k** (101 MHz, $CDCl_3$)

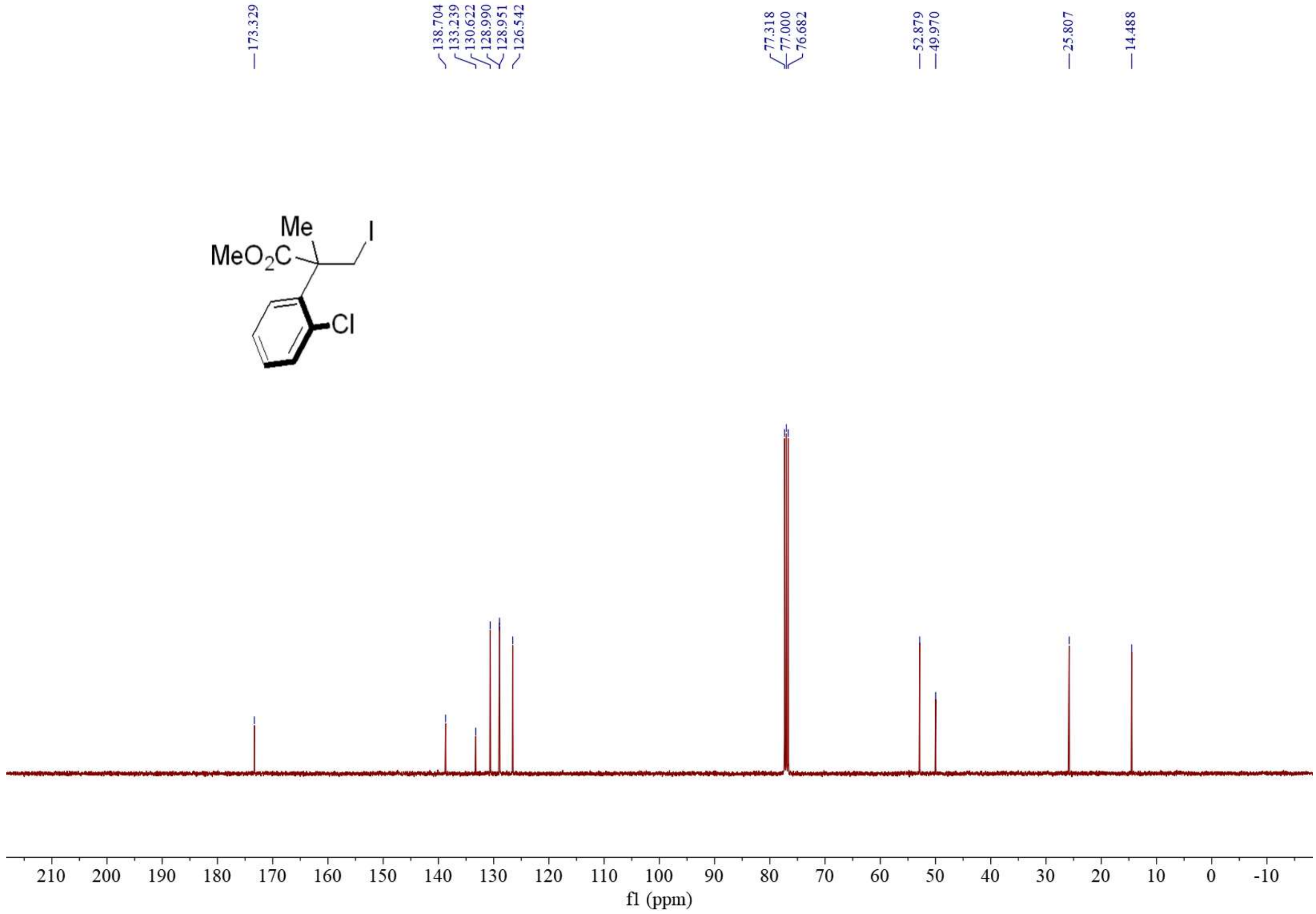

^{1}H NMR spectrum of **5l** (400 MHz, $CDCl_3$)

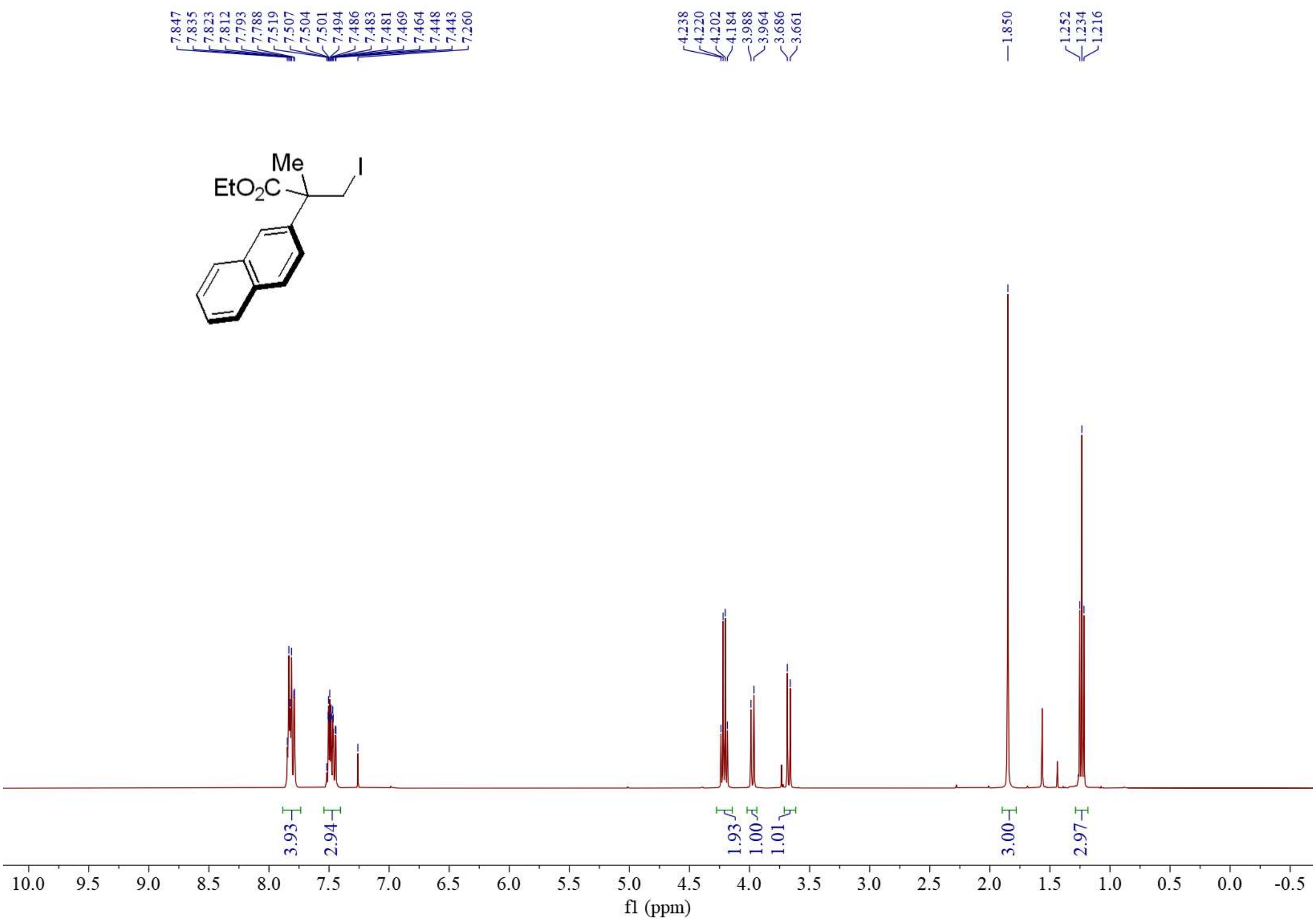

^{13}C NMR spectrum of **5l** (101 MHz, $CDCl_3$)

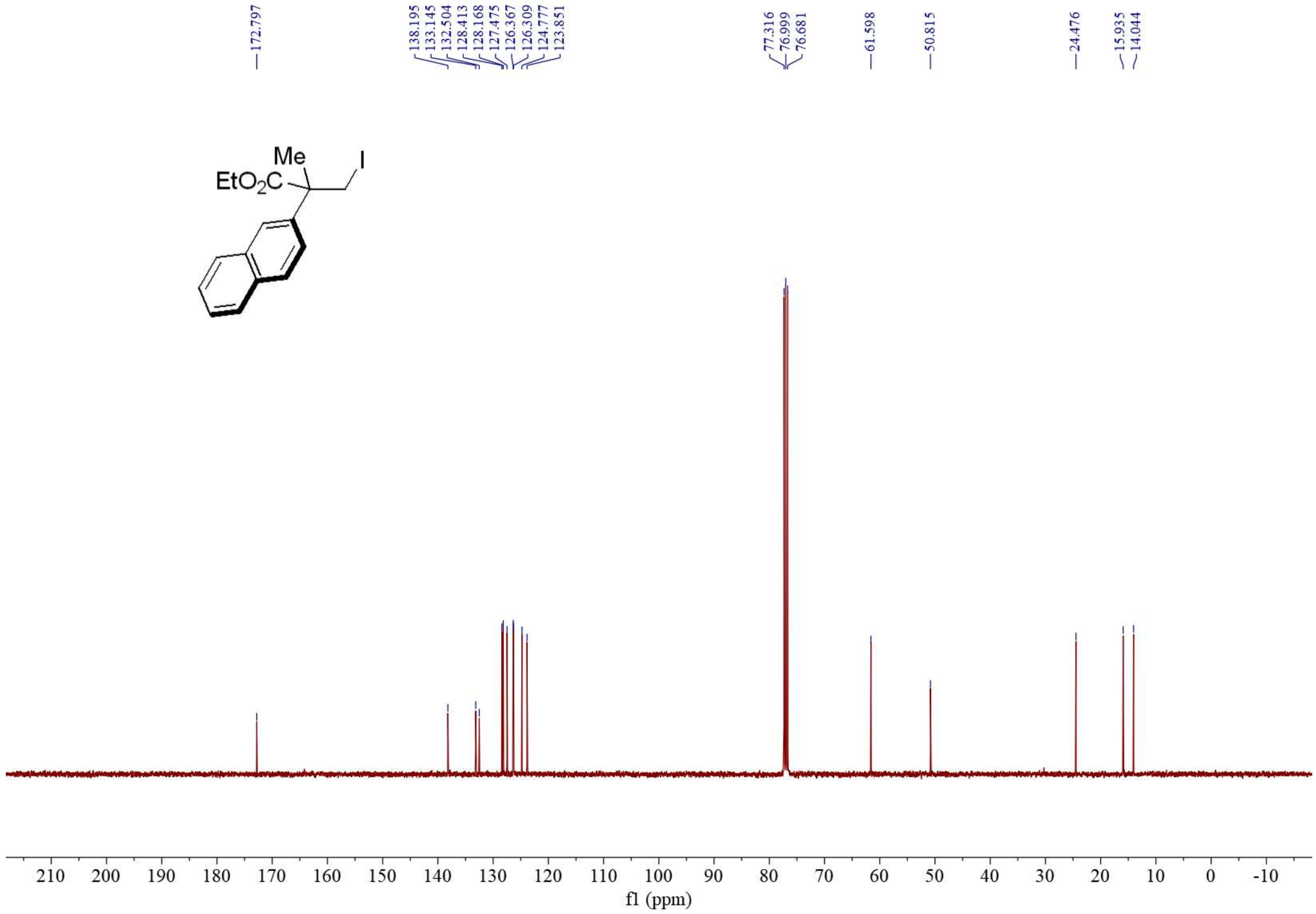

^{1}H NMR spectrum of **5m** (400 MHz, $CDCl_3$)

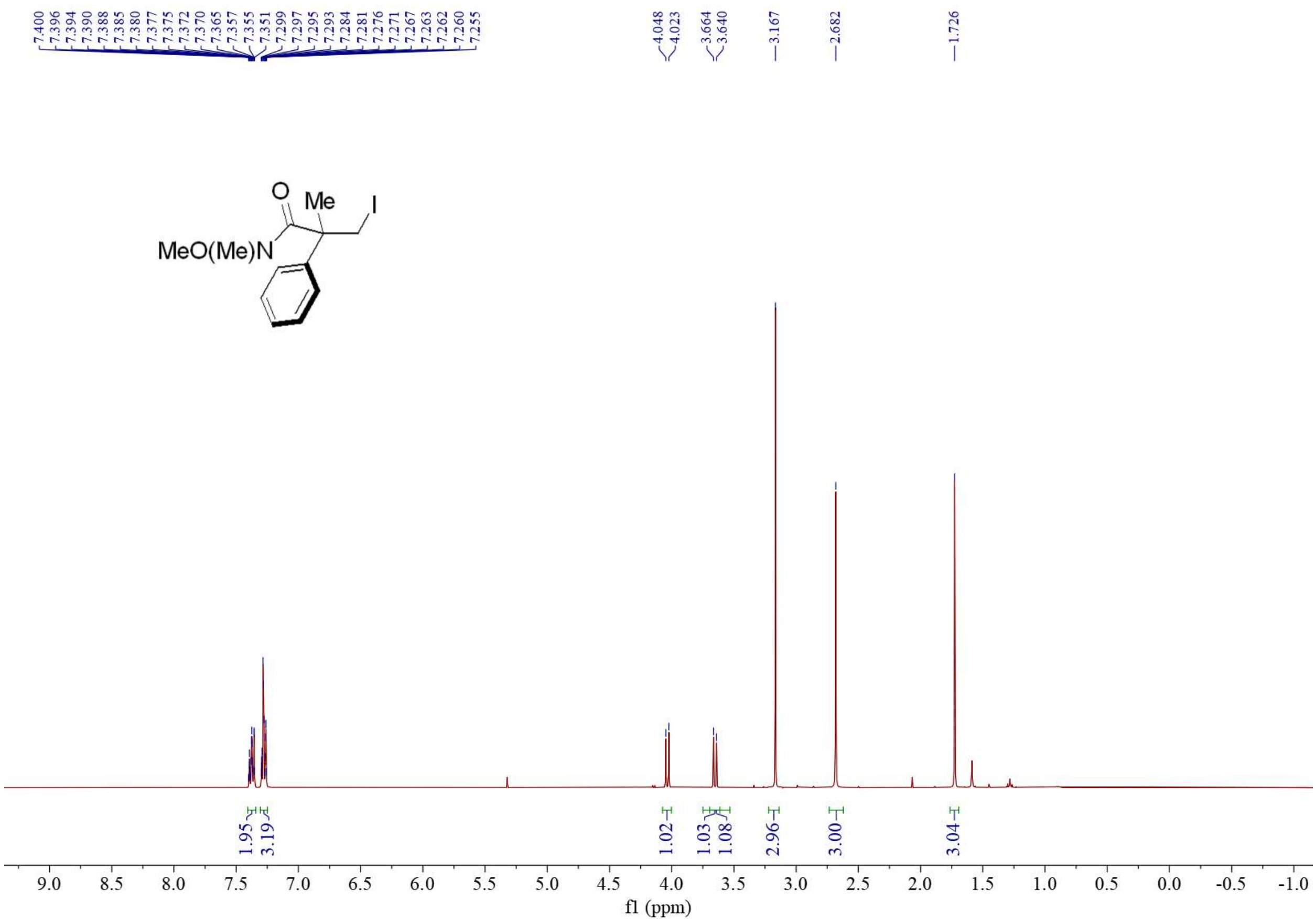

^{13}C NMR spectrum of **5m** (101 MHz, $CDCl_3$)

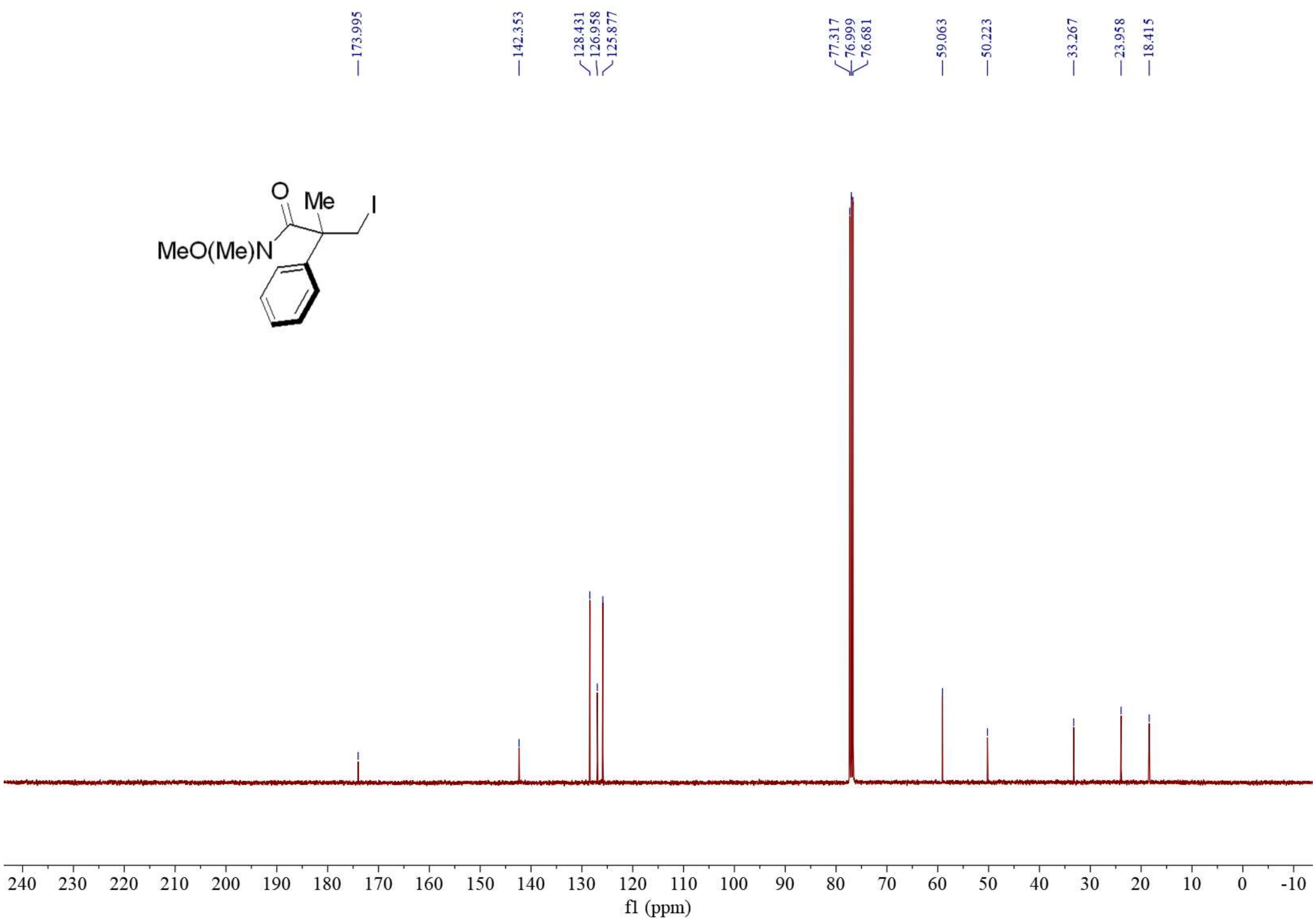

^{1}H NMR spectrum of **5n** (400 MHz, $CDCl_3$)

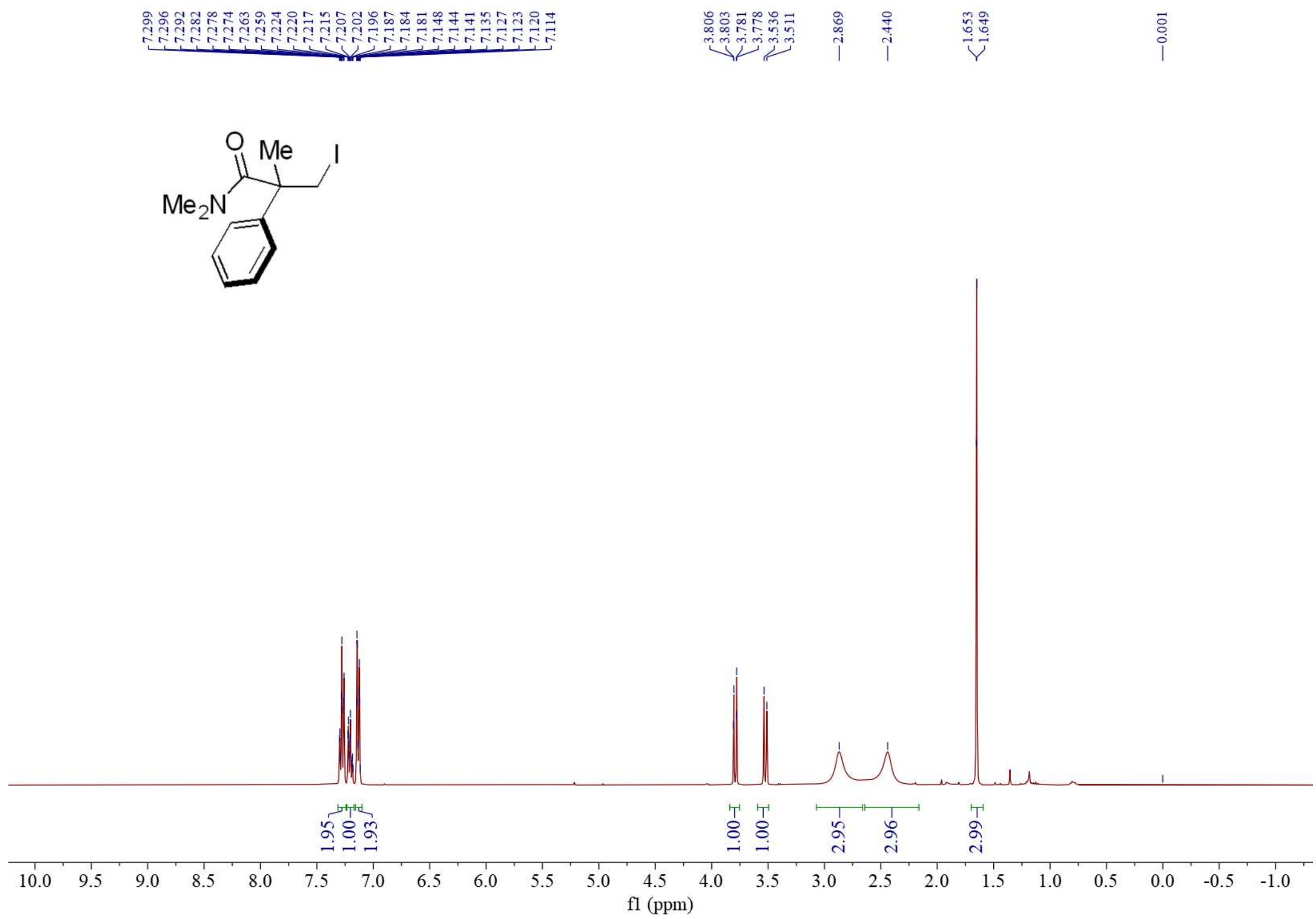

^{13}C NMR spectrum of **5n** (101 MHz, $CDCl_3$)

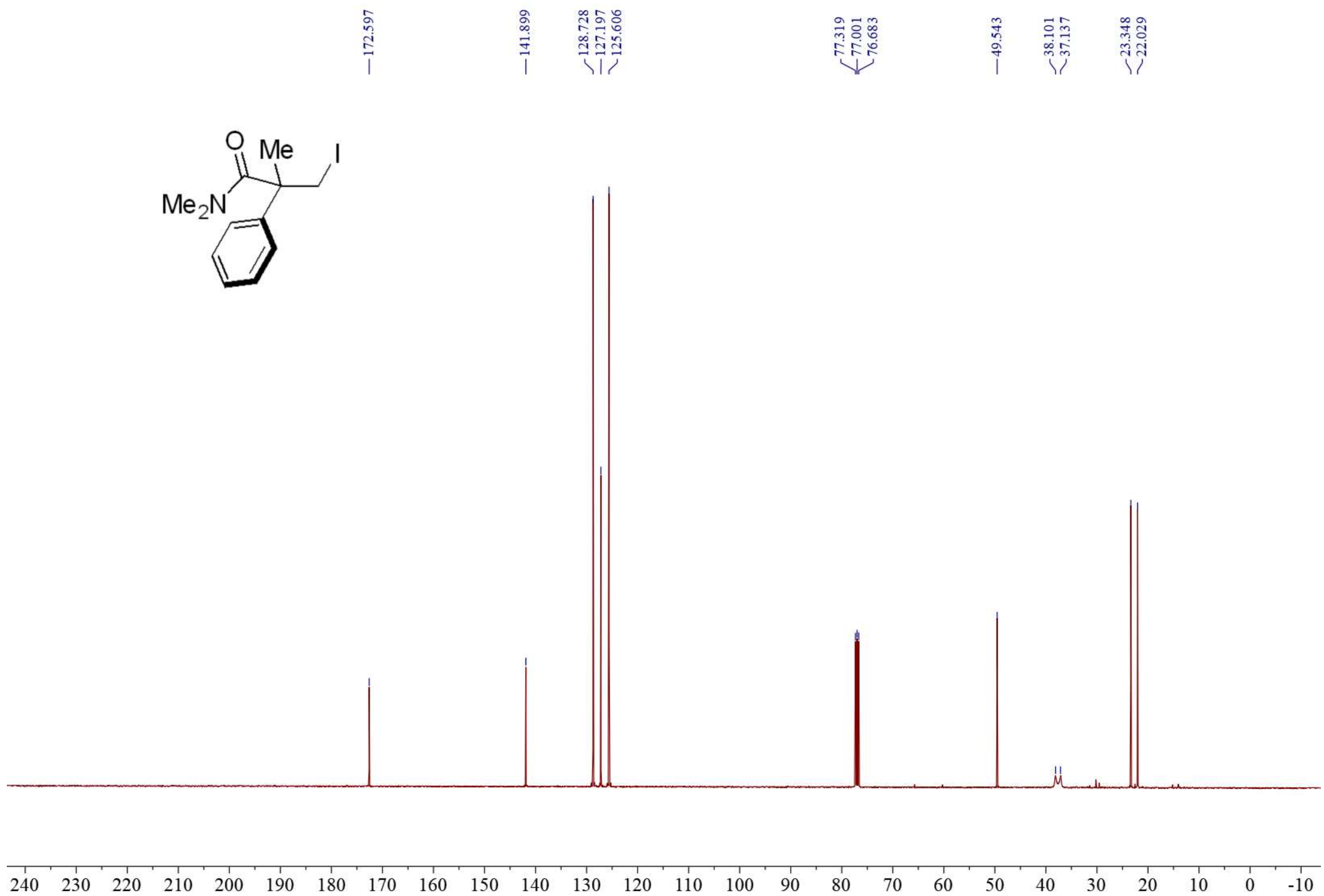

^{1}H NMR spectrum of **5o** (400 MHz, $CDCl_3$)

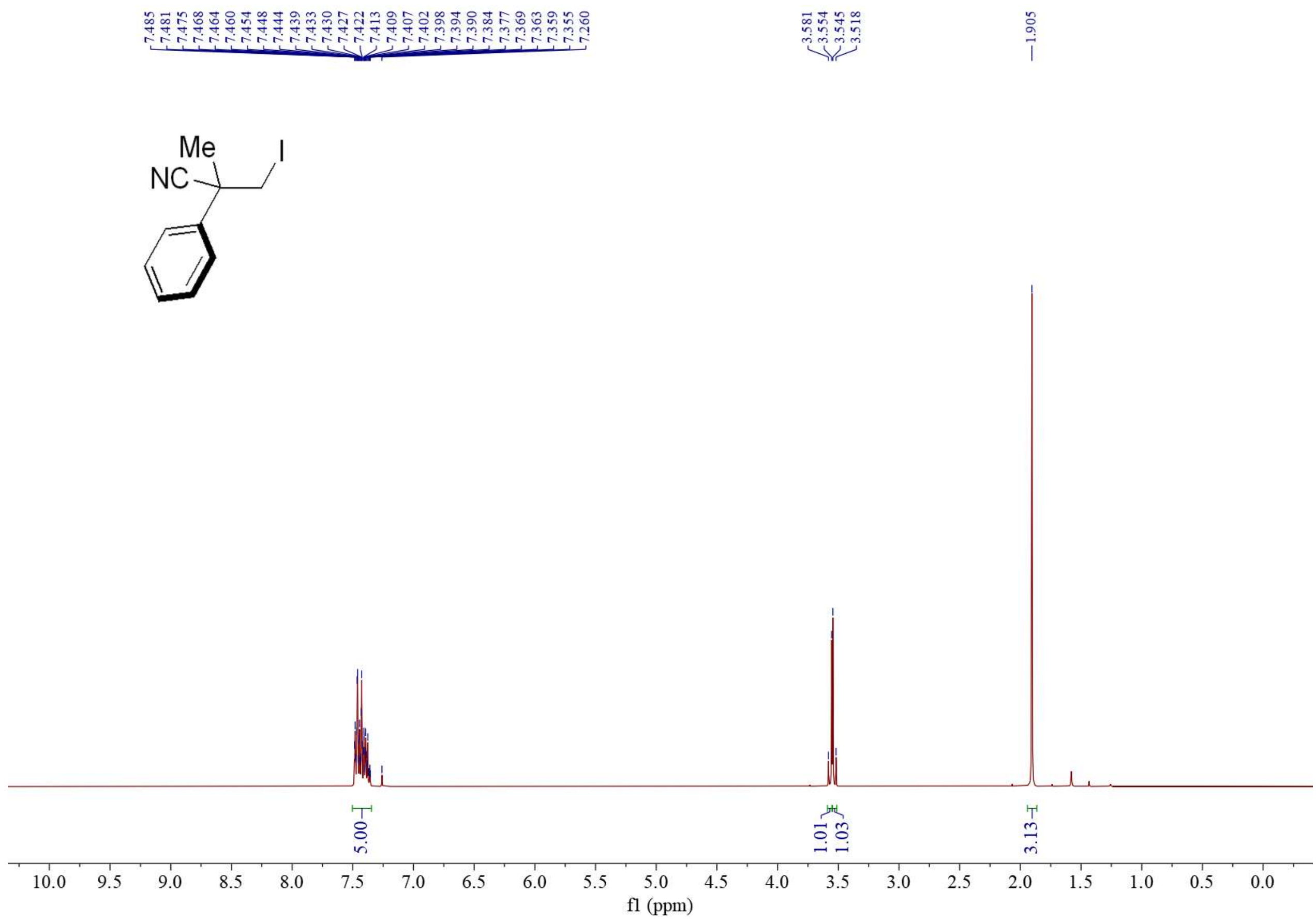

^{13}C NMR spectrum of **5o** (101 MHz, $CDCl_3$)

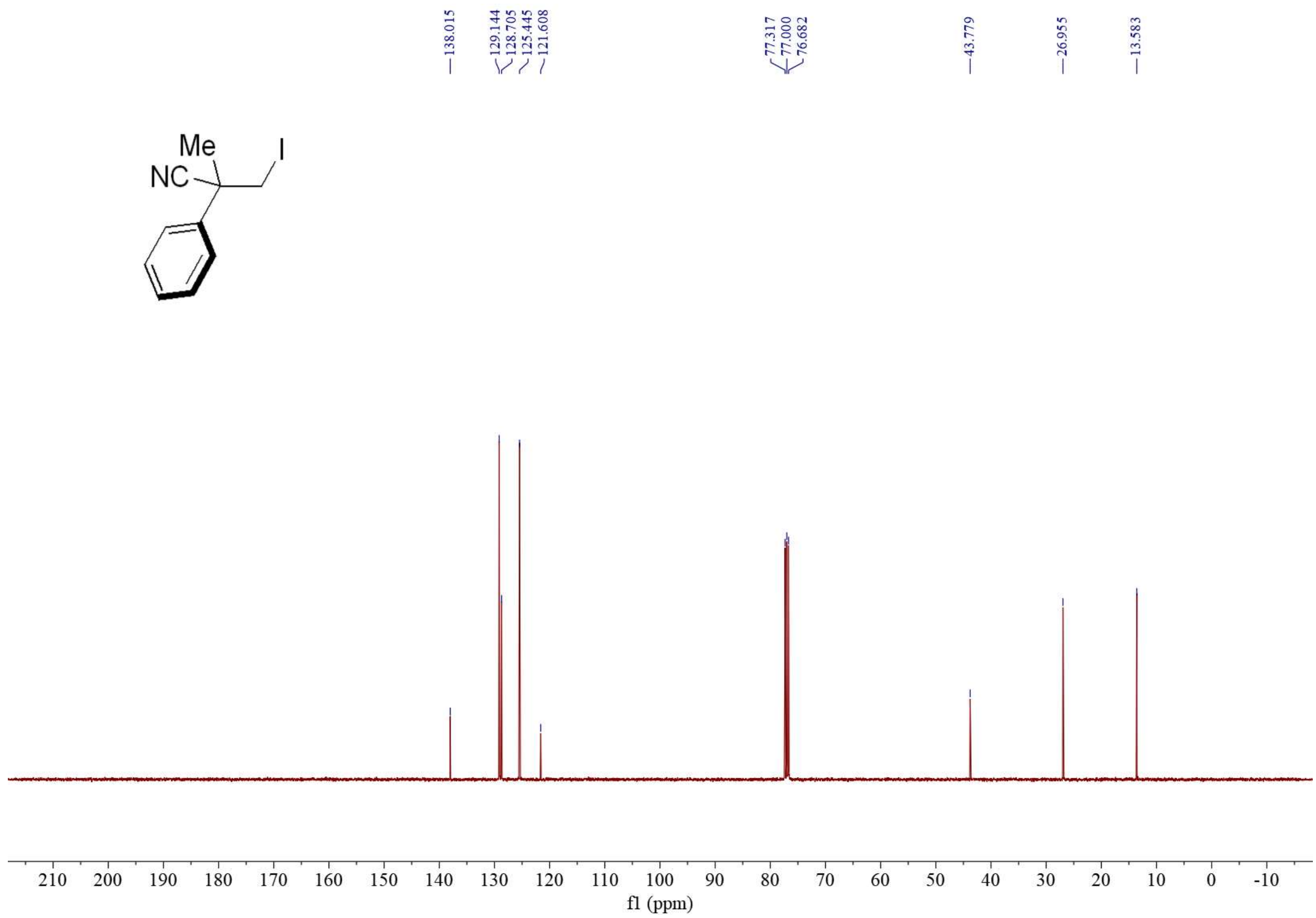

^{1}H NMR spectrum of **5r** (400 MHz, $CDCl_3$)

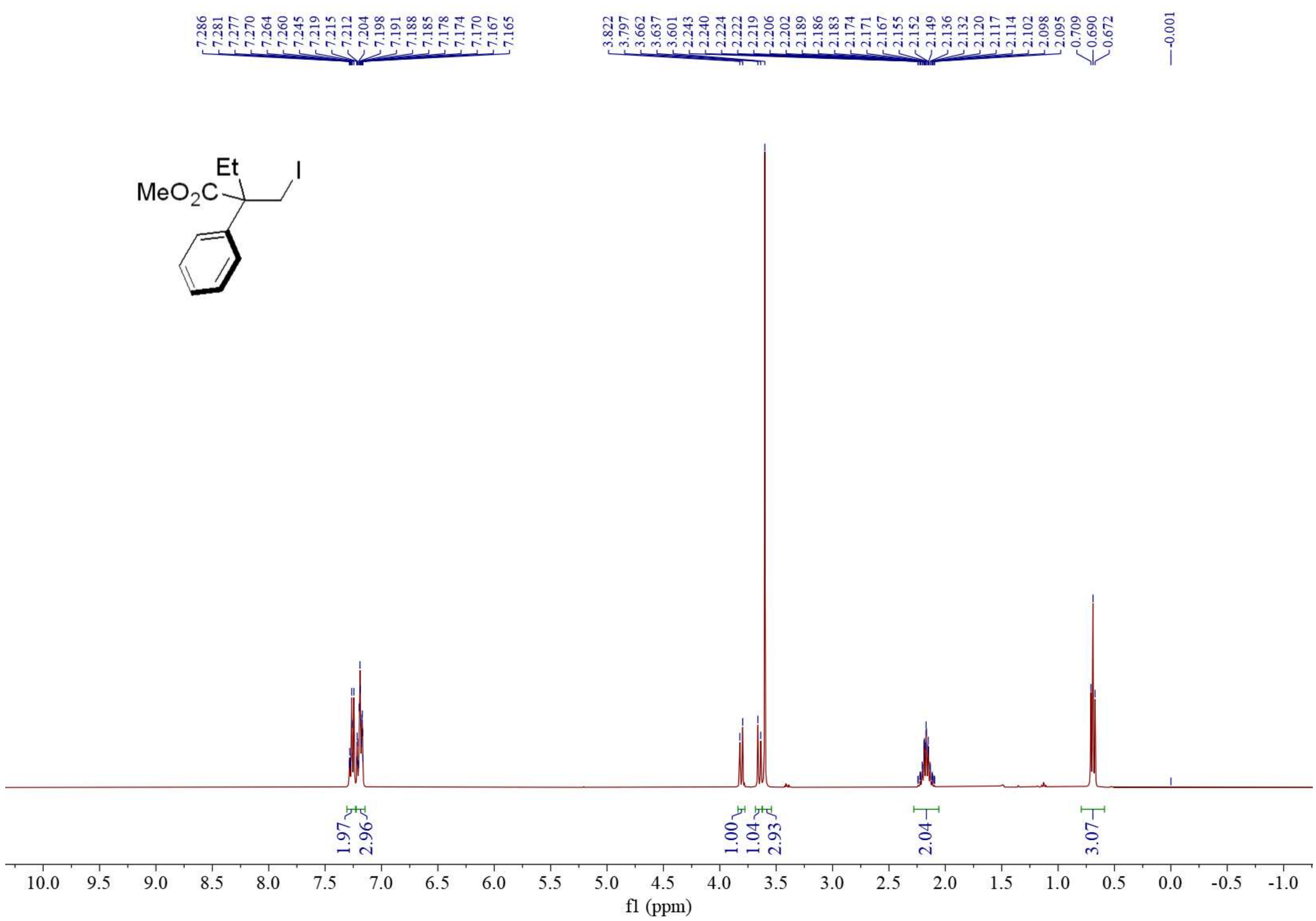

^{13}C NMR spectrum of **5r** (101 MHz, $CDCl_3$)

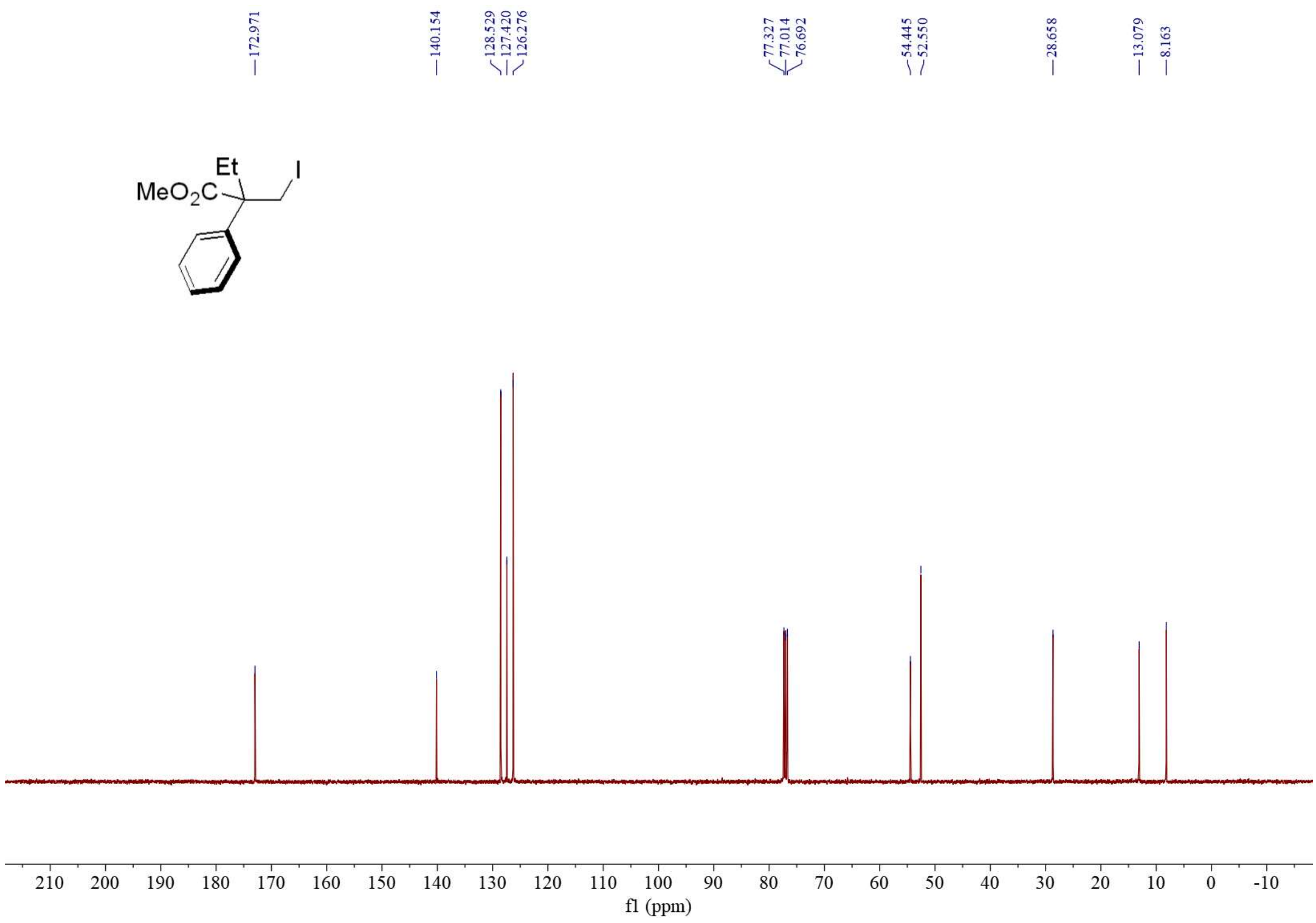

^{1}H NMR spectrum of **5s** (400 MHz, $CDCl_3$)

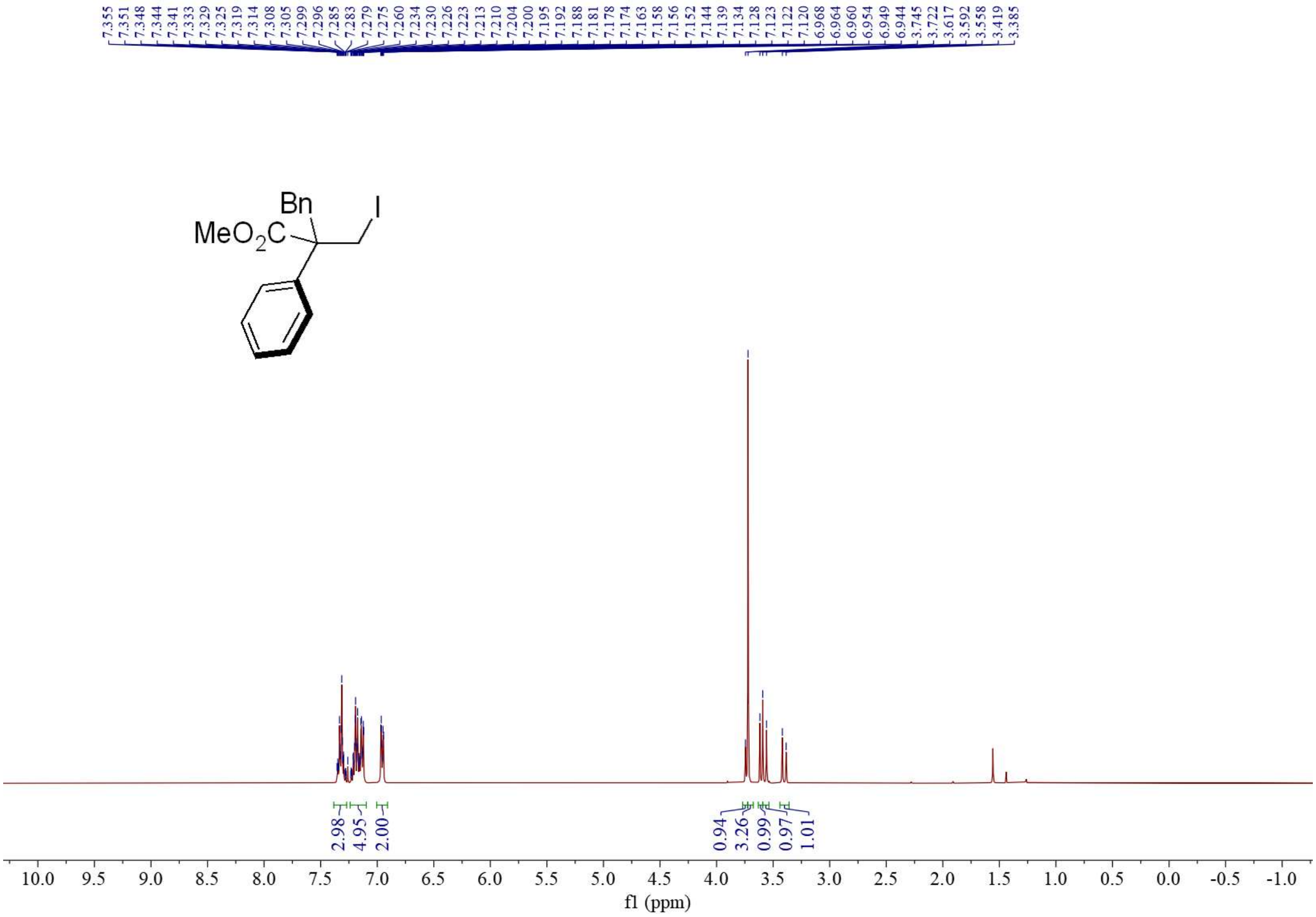

^{13}C NMR spectrum of **5s** (101 MHz, $CDCl_3$)

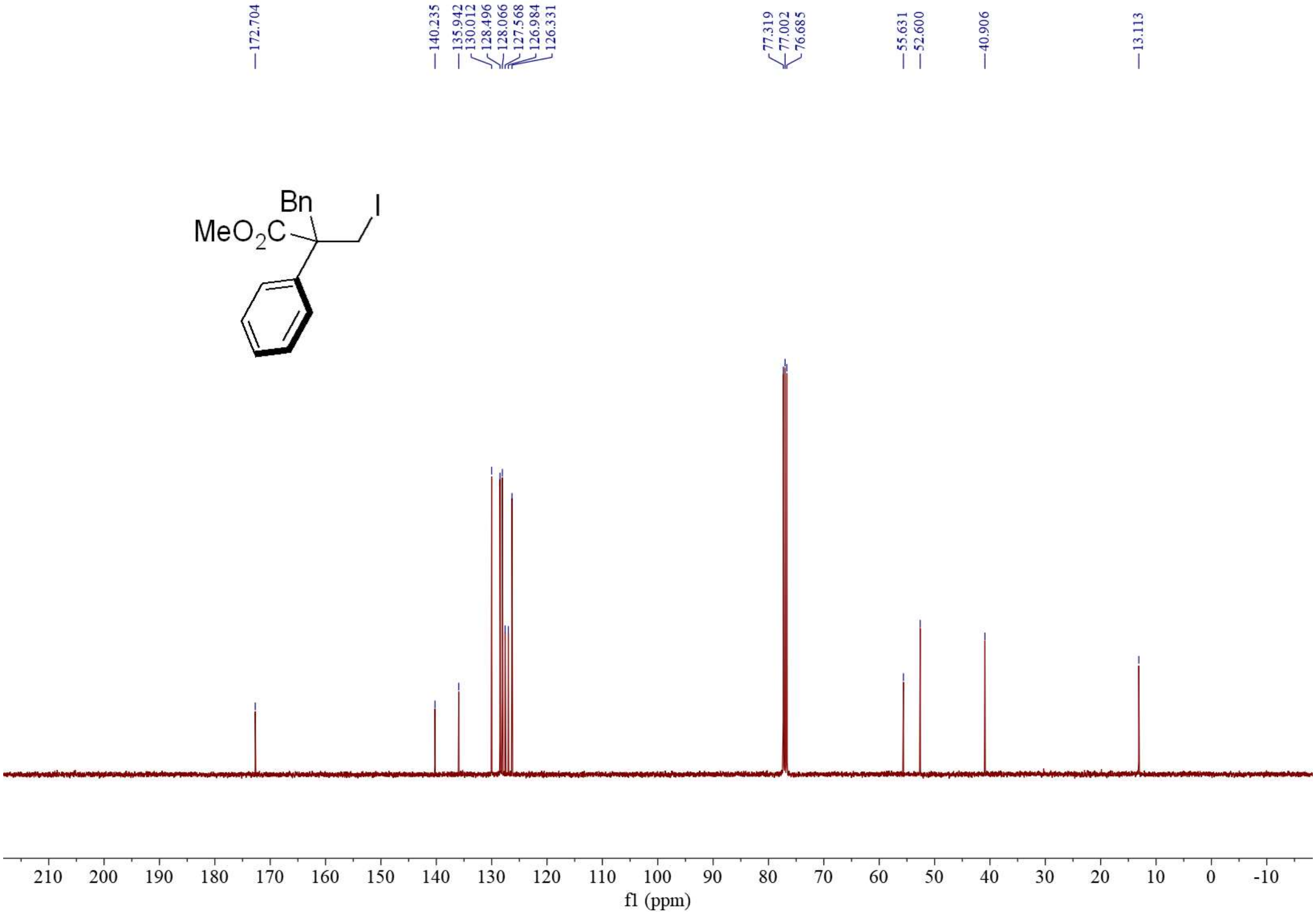

^{1}H NMR spectrum of **5t** (400 MHz, $CDCl_3$)

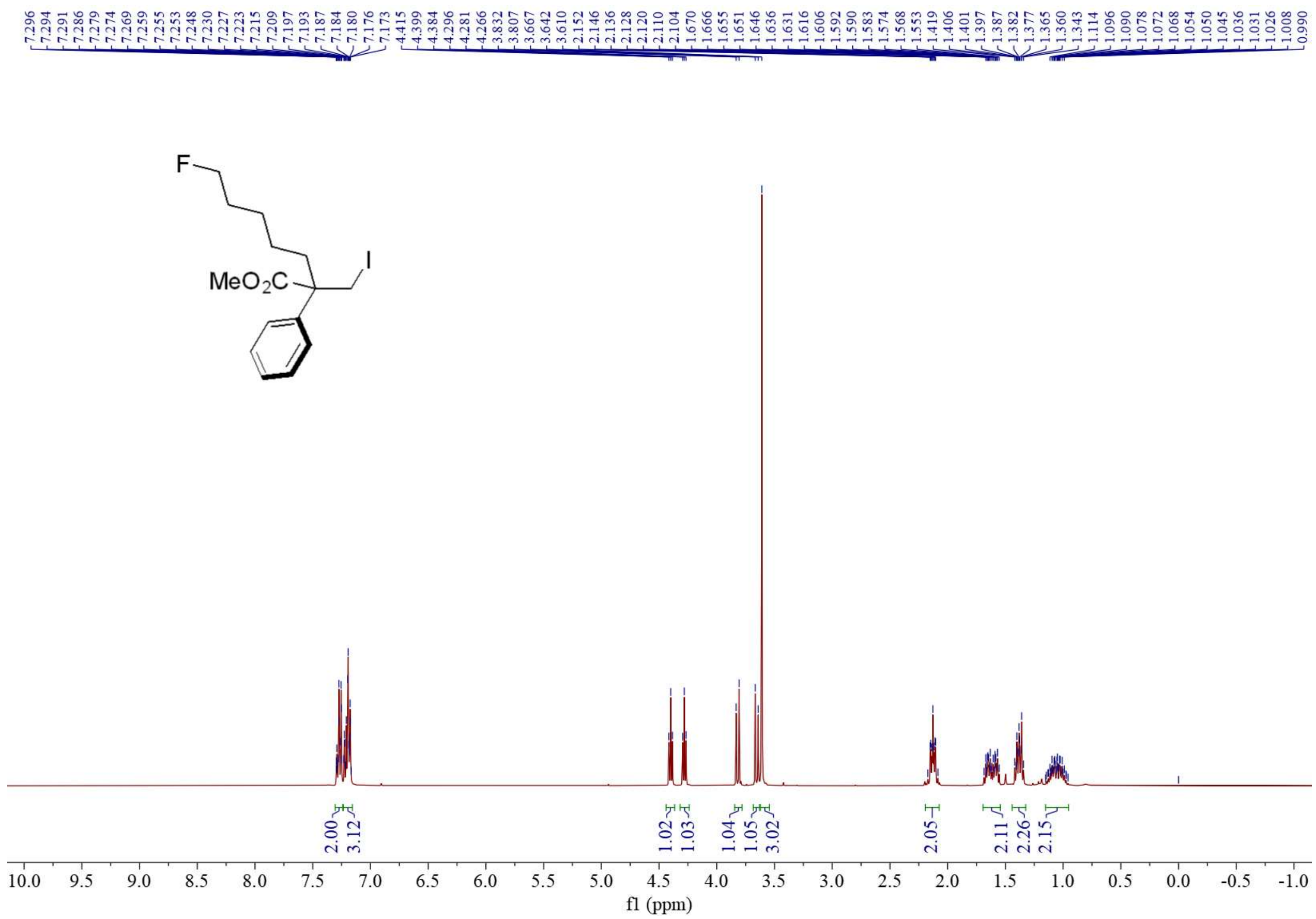

^{13}C NMR spectrum of **5t** (101 MHz, $CDCl_3$)

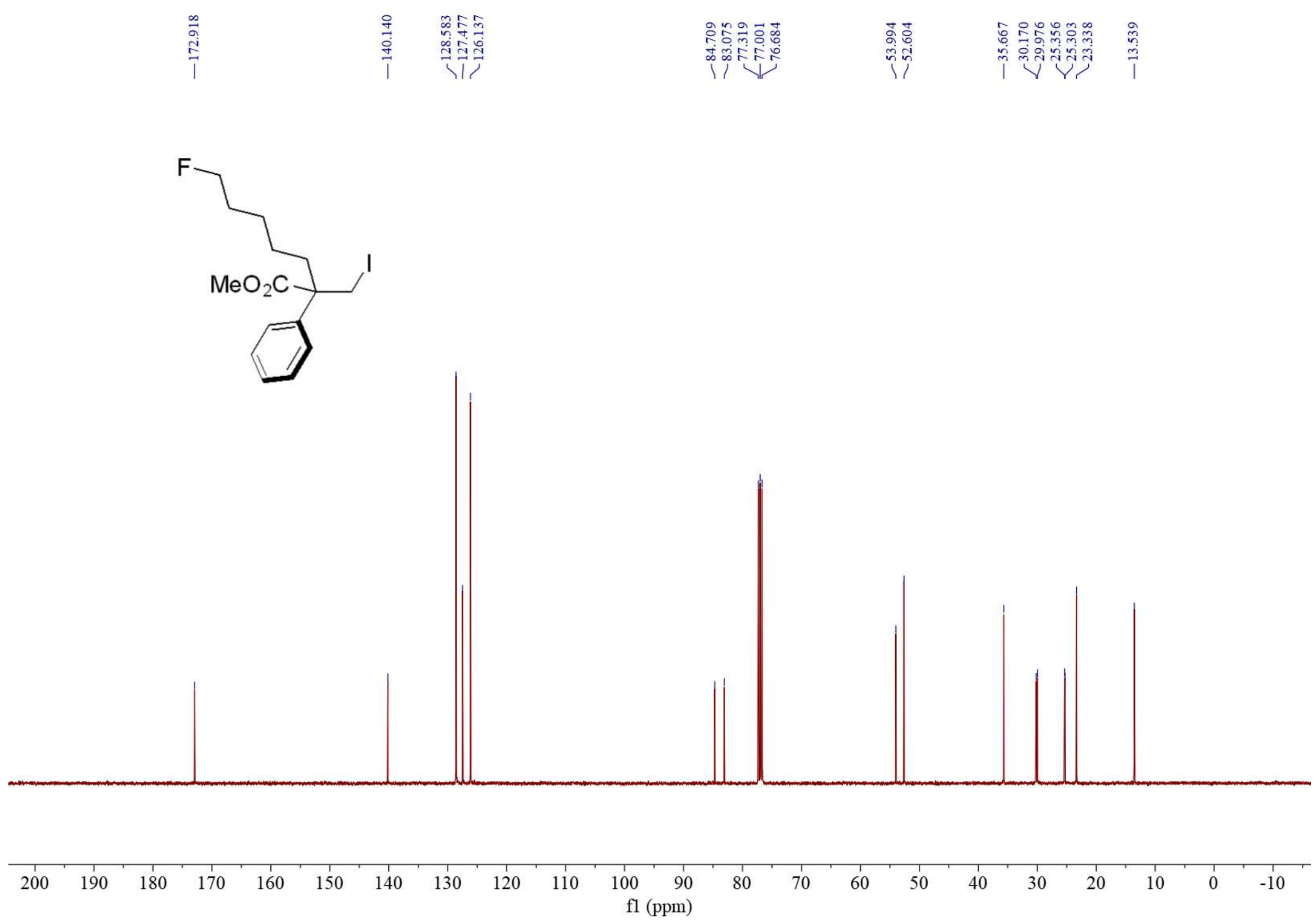

$^{19}F\{^1H\}$ NMR spectrum of **5t** (377 MHz, $CDCl_3$)

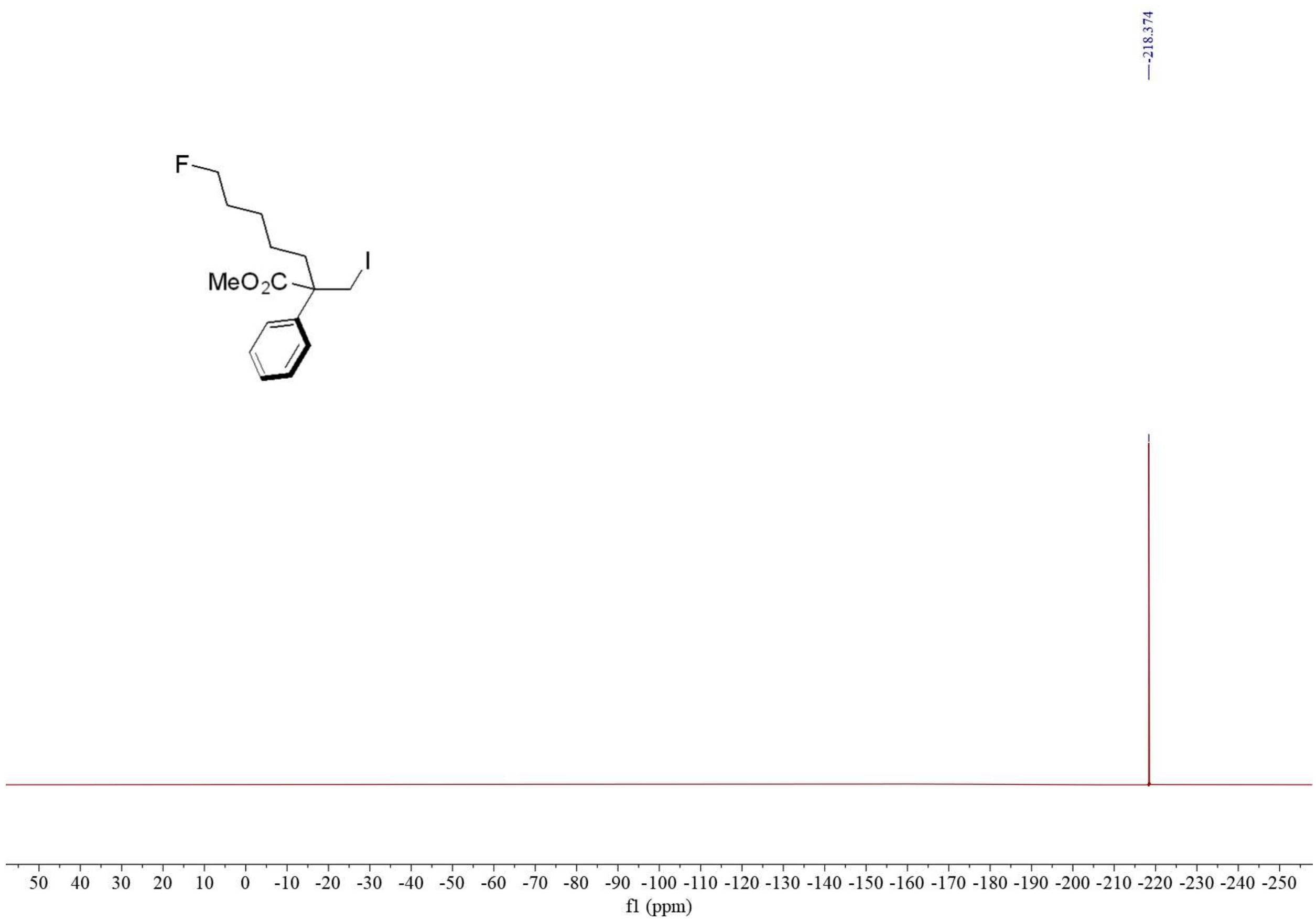

^{19}F NMR spectrum of **5t** (377 MHz, $CDCl_3$)

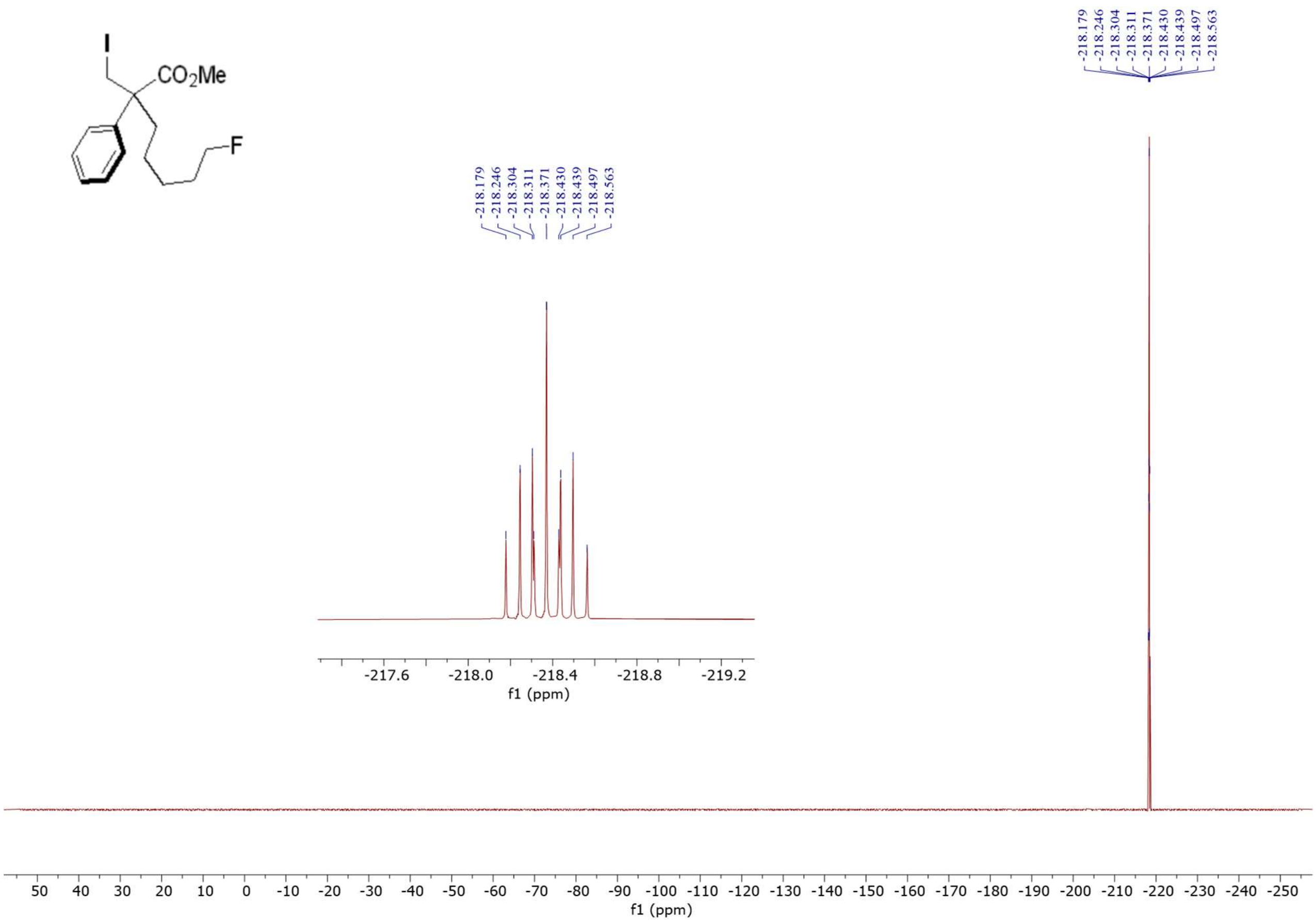

^{1}H NMR spectrum of **5u** (400 MHz, $CDCl_3$)

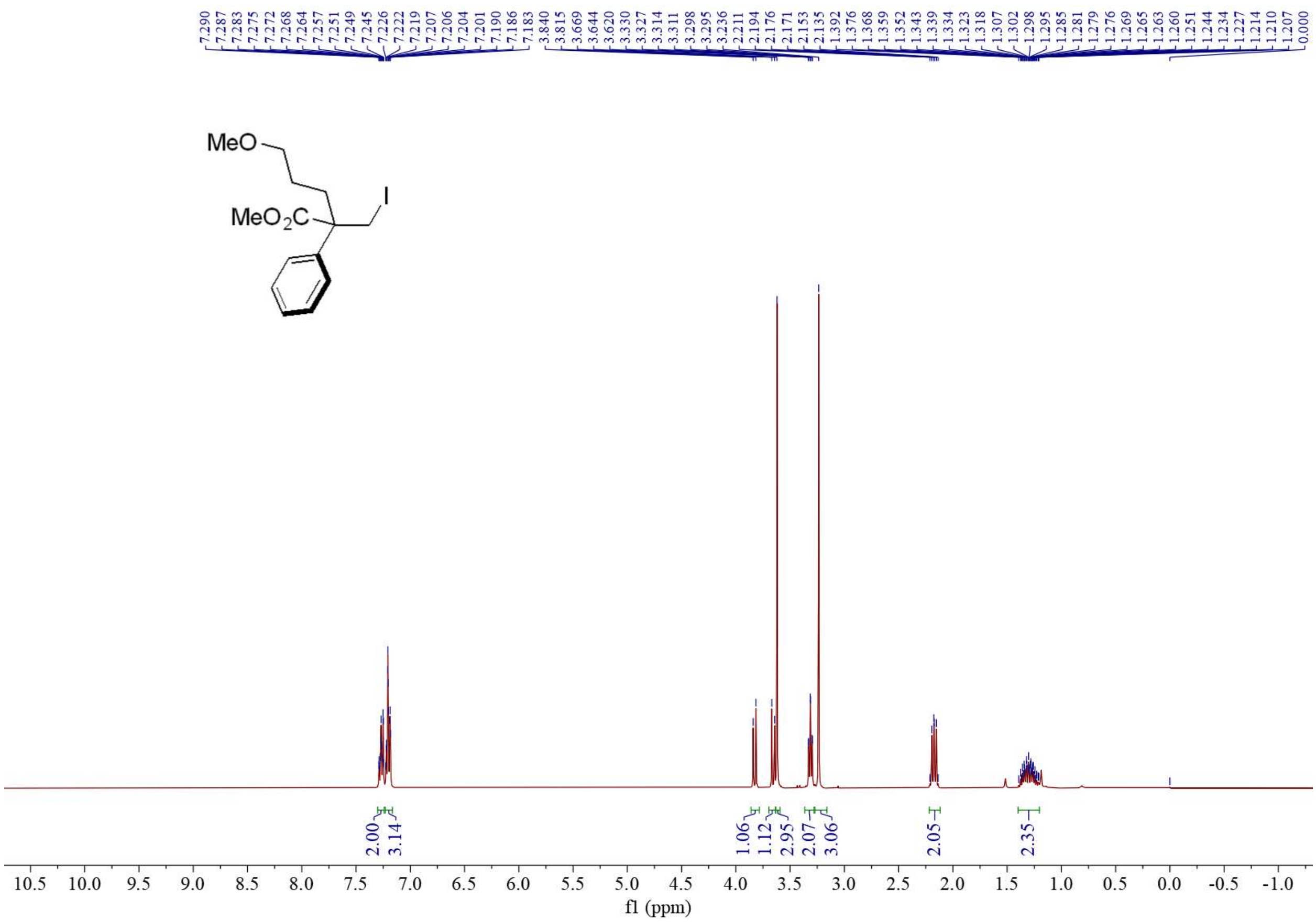

^{13}C NMR spectrum of **5u** (101 MHz, $CDCl_3$)

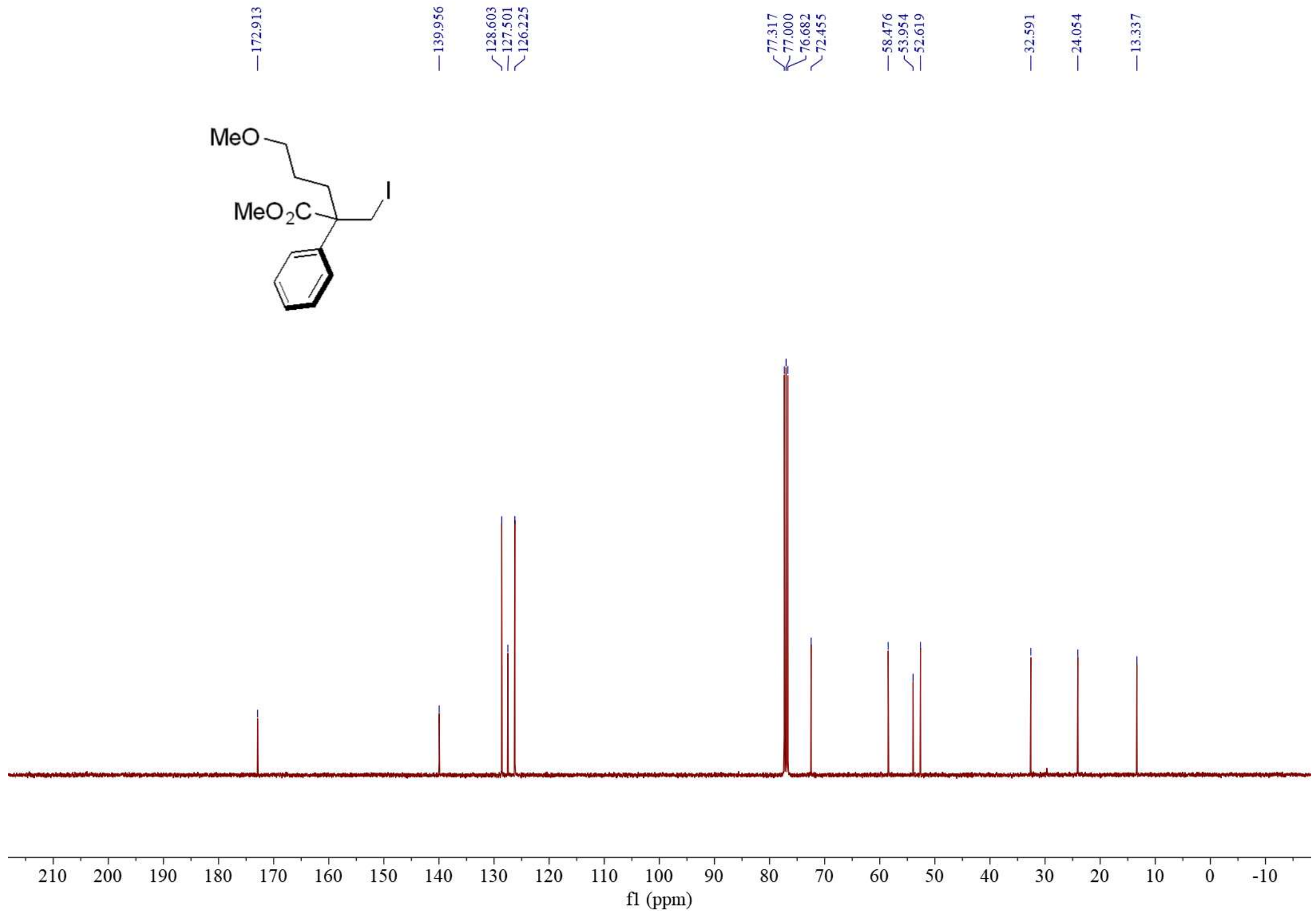

^{1}H NMR spectrum of **5v** (400 MHz, $CDCl_3$)

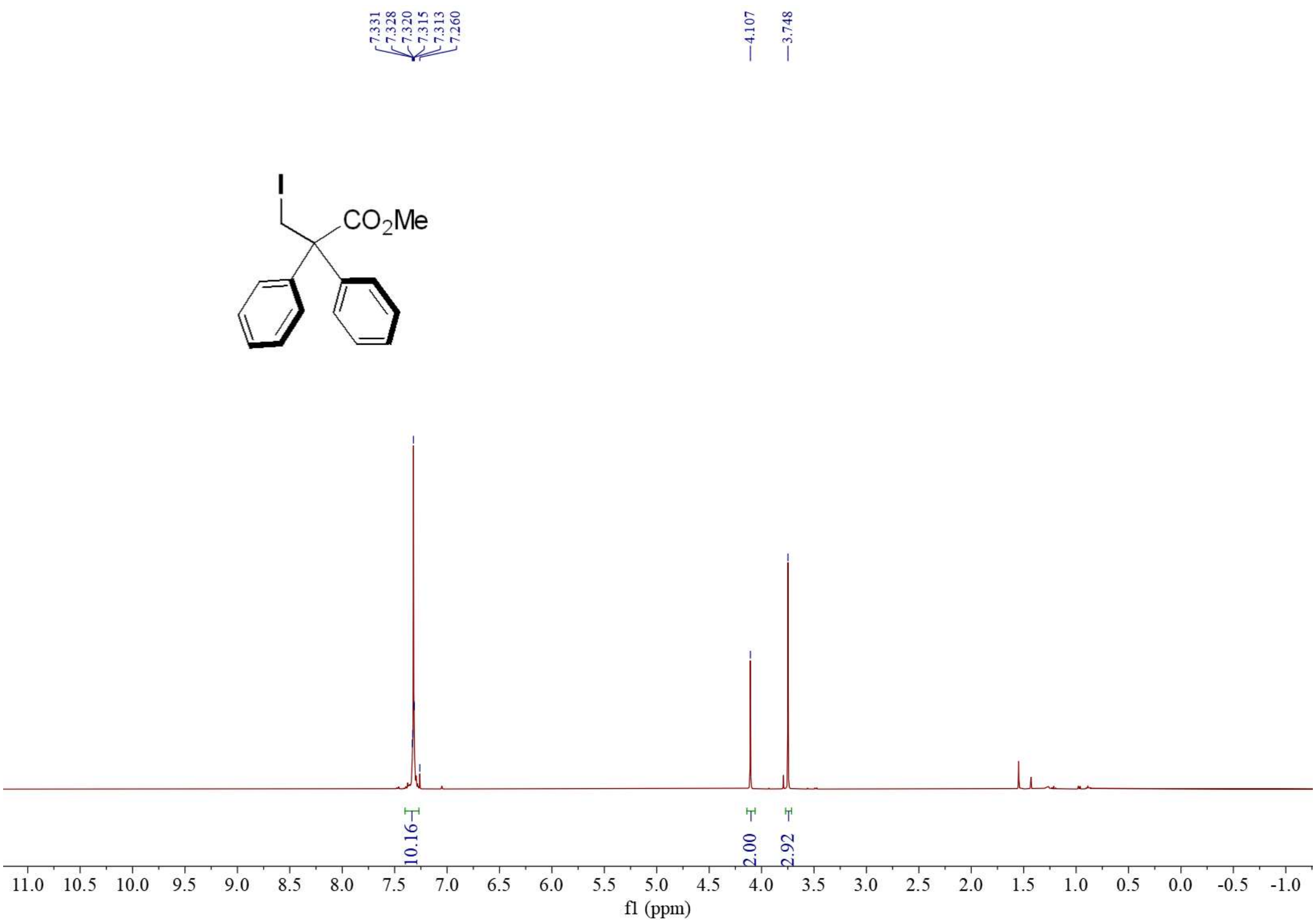

^{13}C NMR spectrum of **5v** (101 MHz, $CDCl_3$)

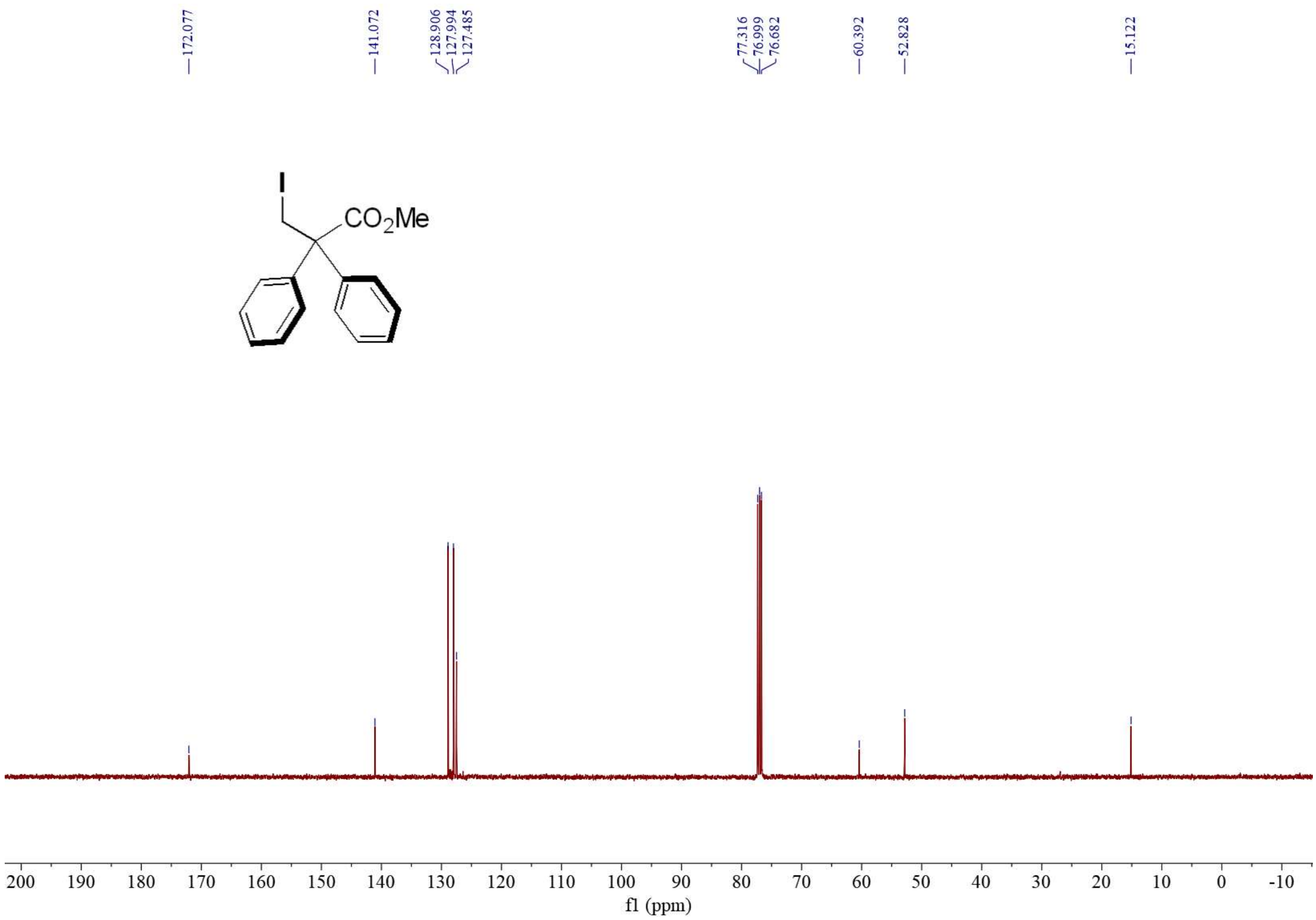

^{1}H NMR spectrum of **5w** (800 MHz, $CDCl_3$)

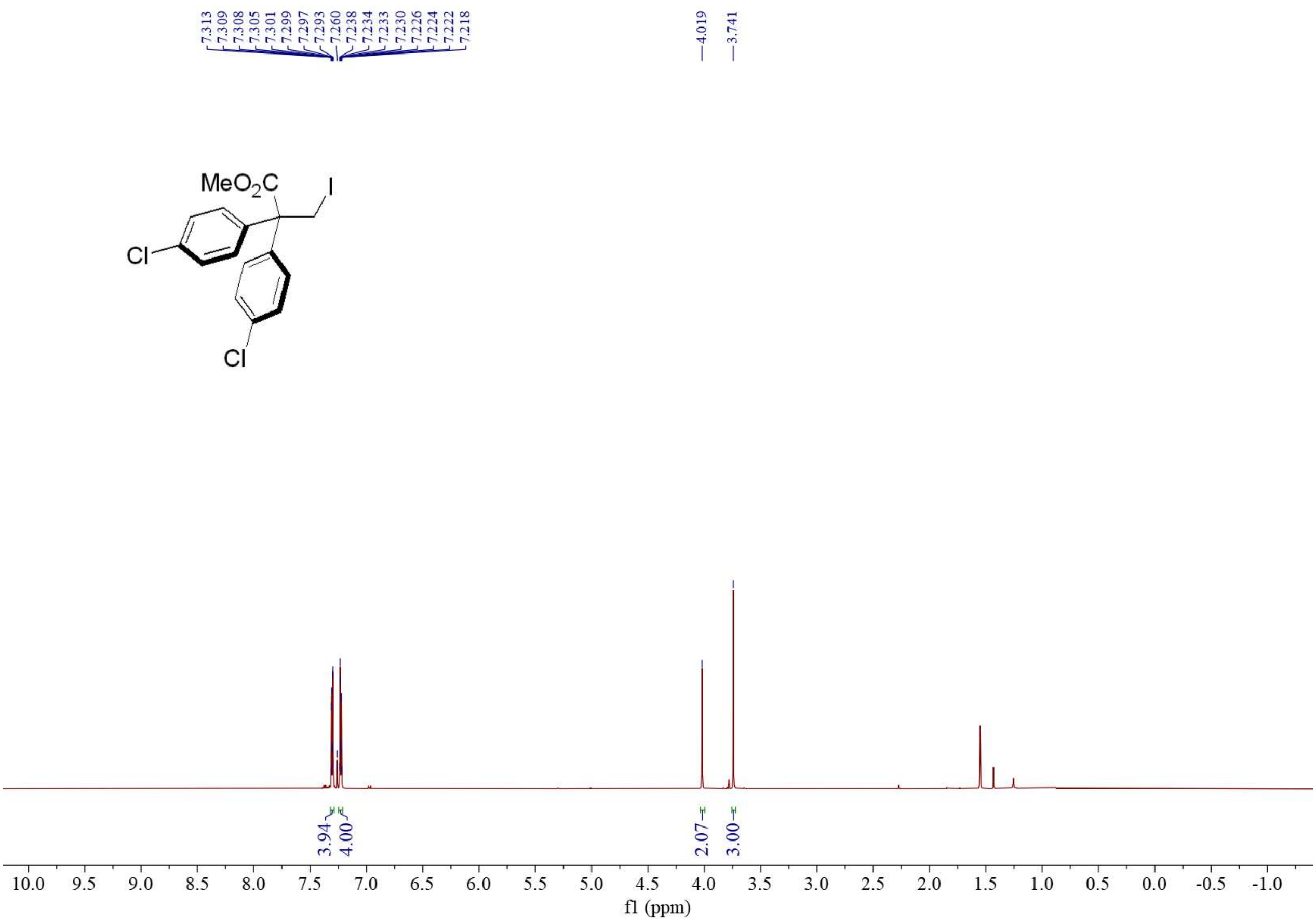

^{13}C NMR spectrum of **5w** (201 MHz, $CDCl_3$)

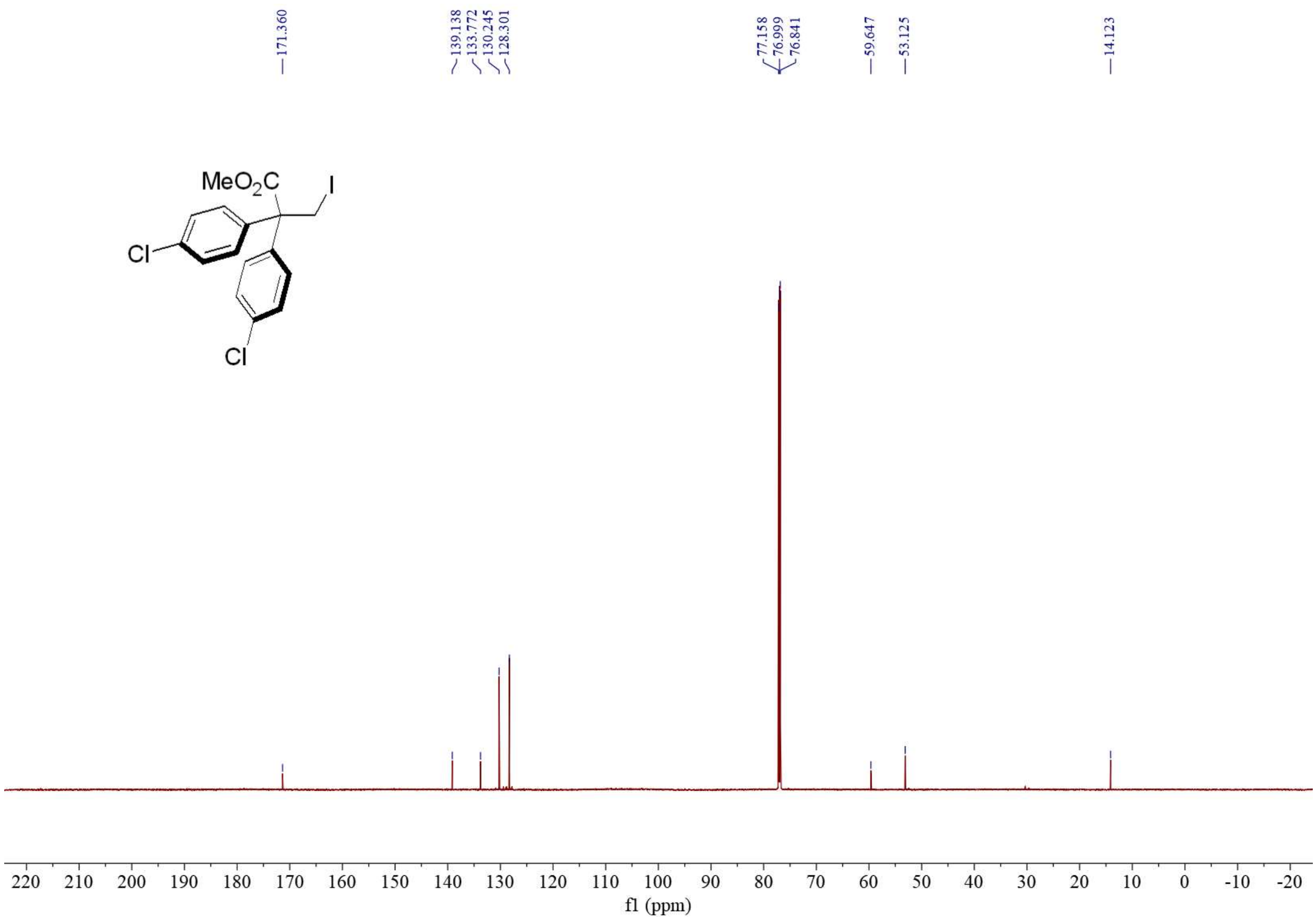

^{1}H NMR spectrum of **5x** (400 MHz, $CDCl_3$)

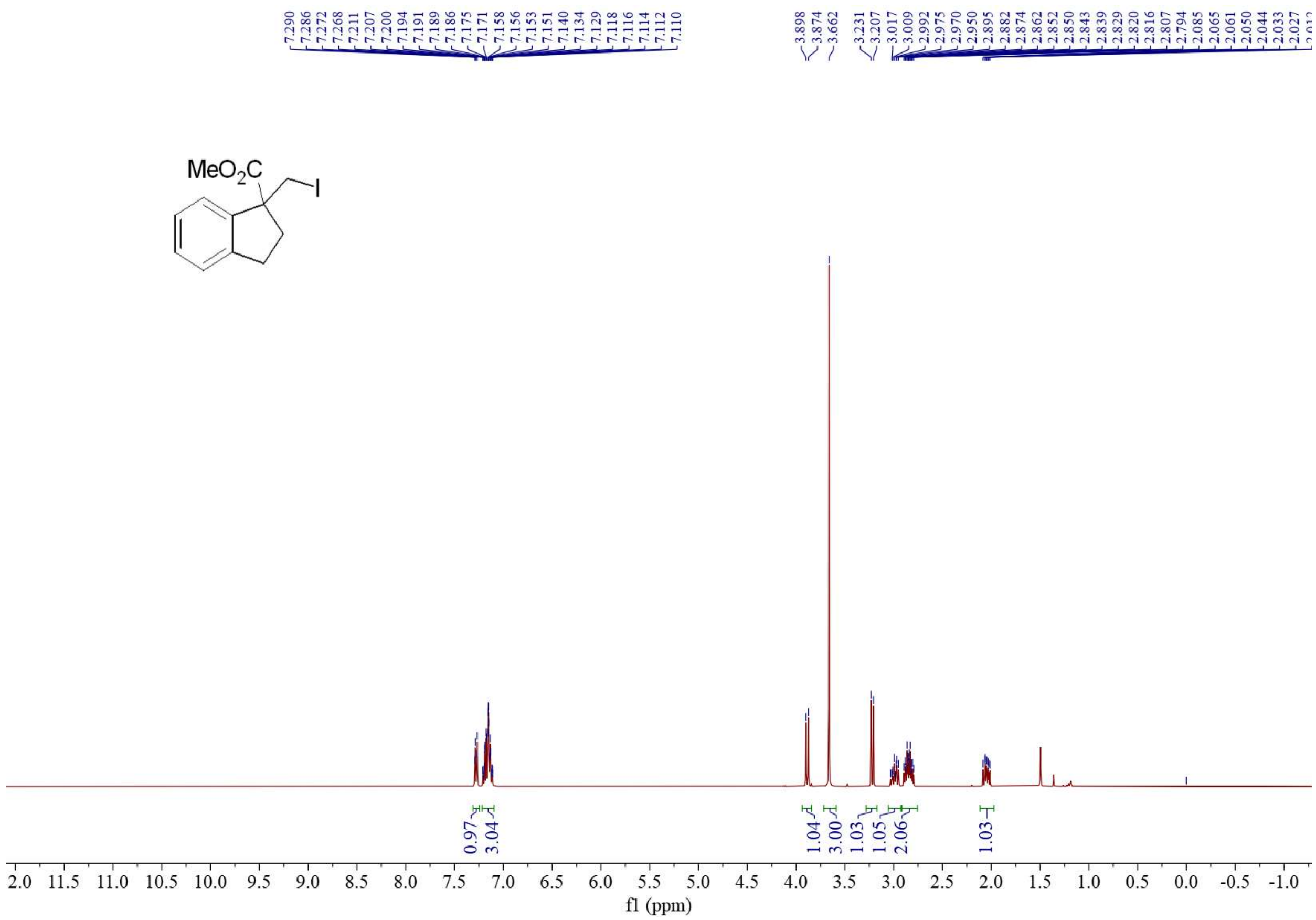

^{13}C NMR spectrum of **5x** (101 MHz, $CDCl_3$)

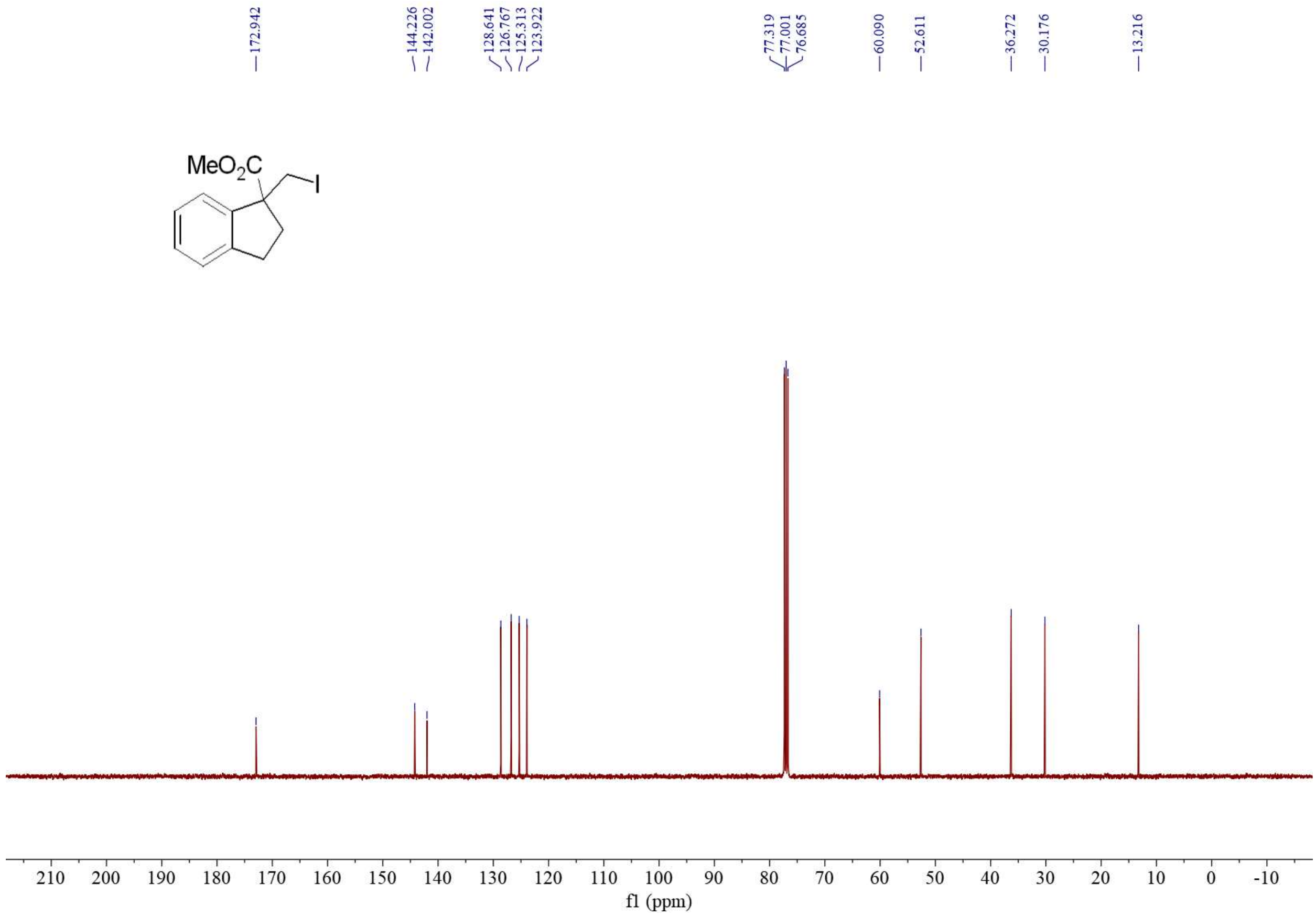

^{1}H NMR spectrum of **5y** (400 MHz, $CDCl_3$)

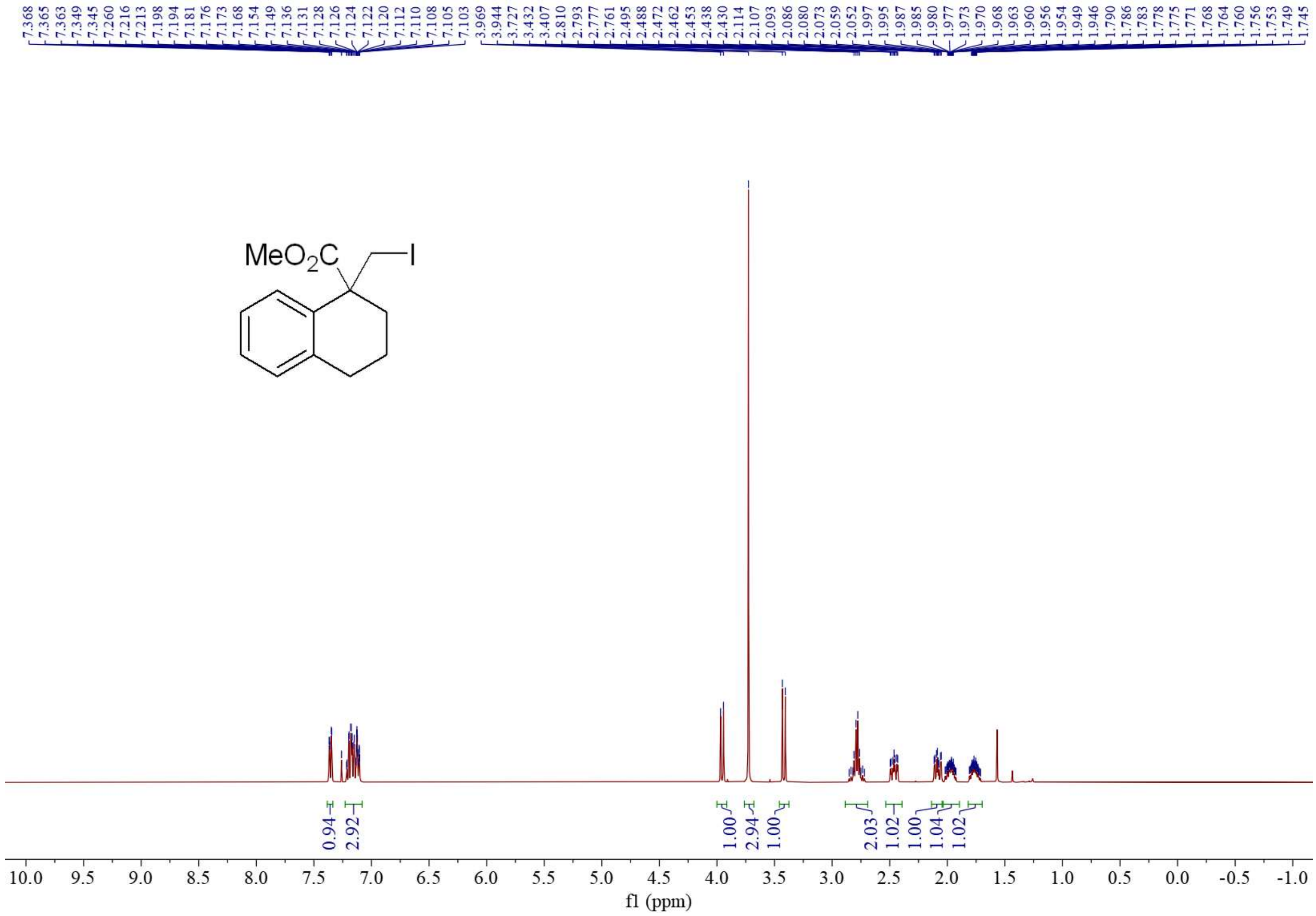

^{13}C NMR spectrum of **5y** (101 MHz, $CDCl_3$)

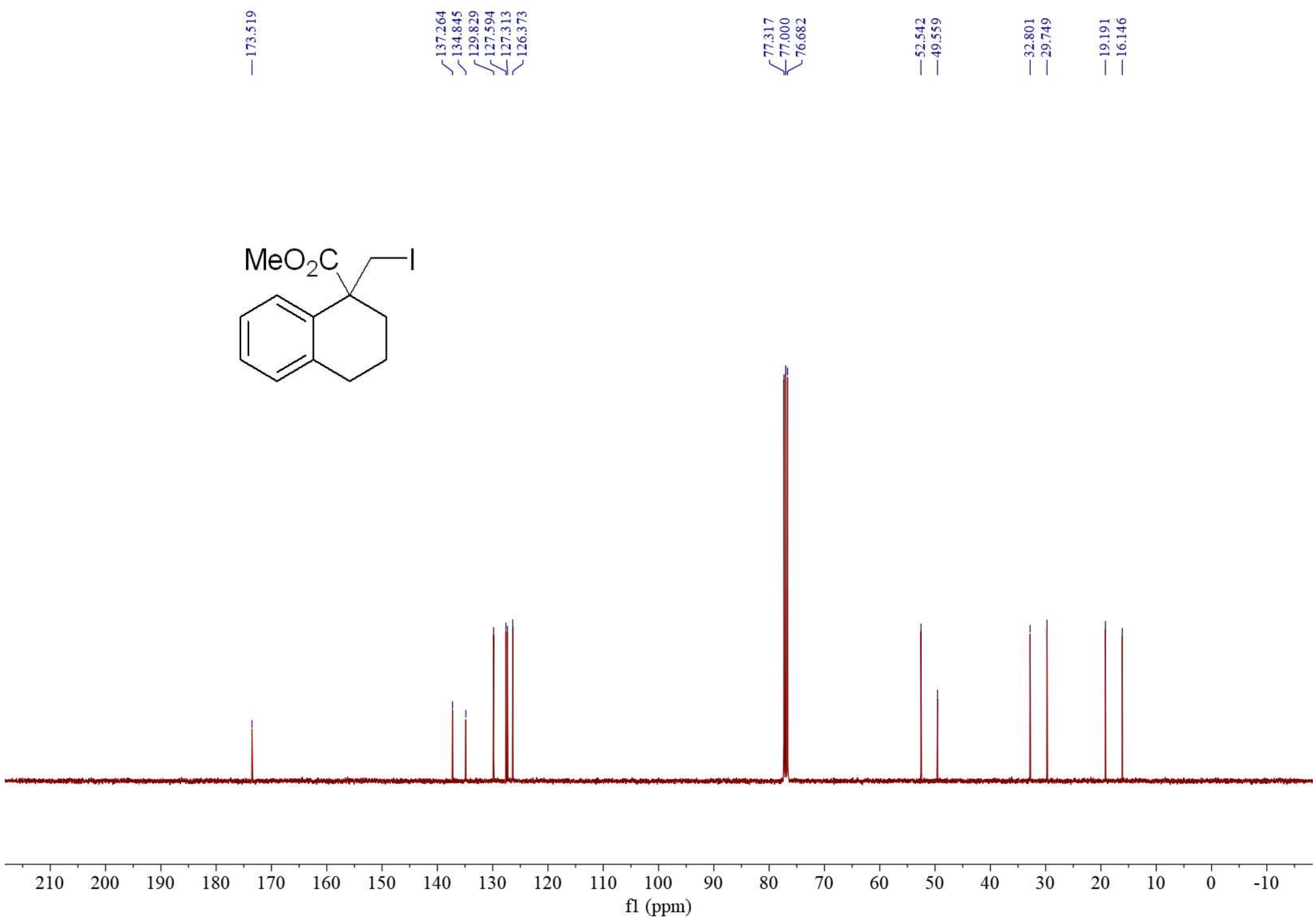

^{1}H NMR spectrum of **5z** (400 MHz, $CDCl_3$)

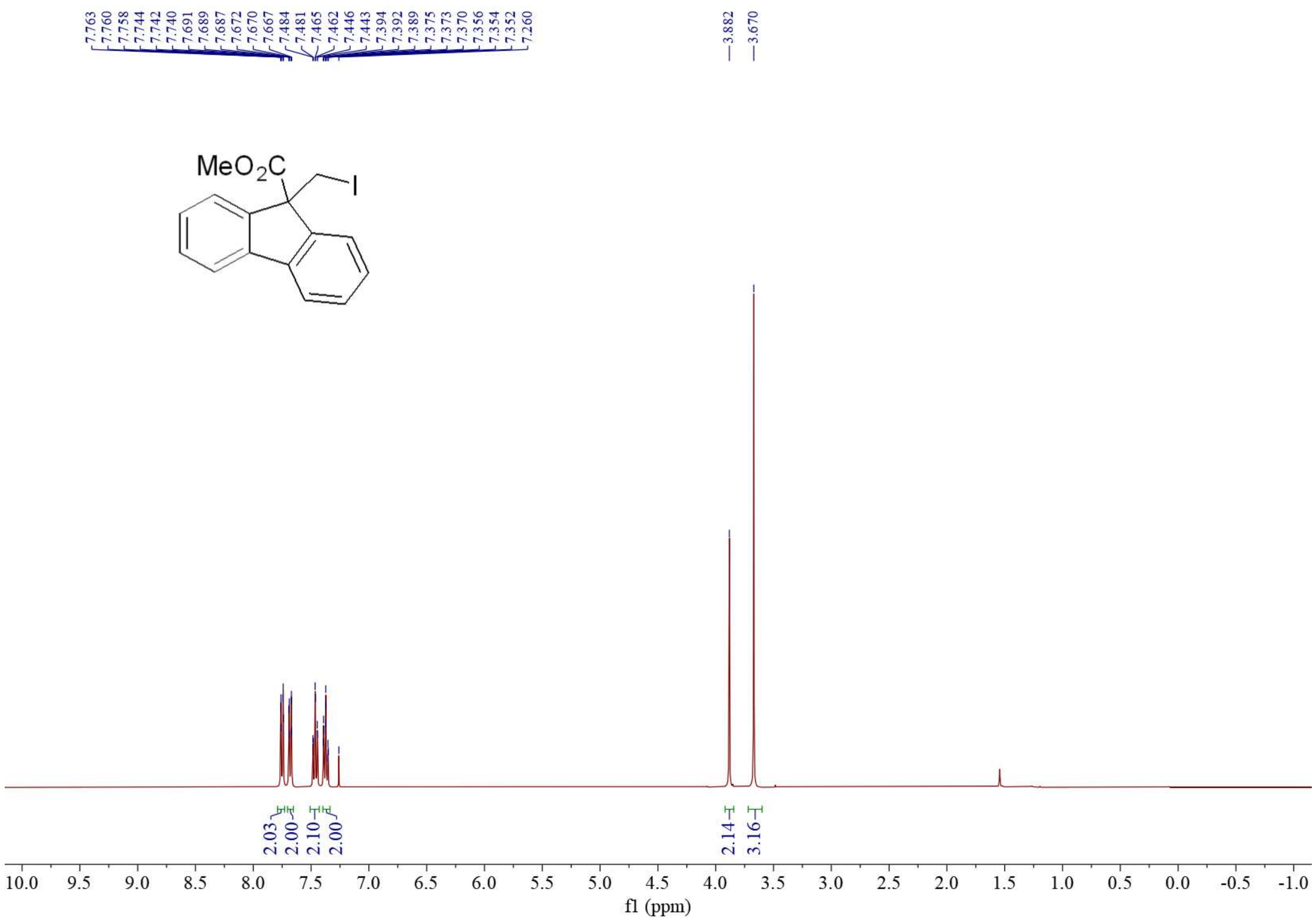

^{13}C NMR spectrum of **5z** (101 MHz, $CDCl_3$)

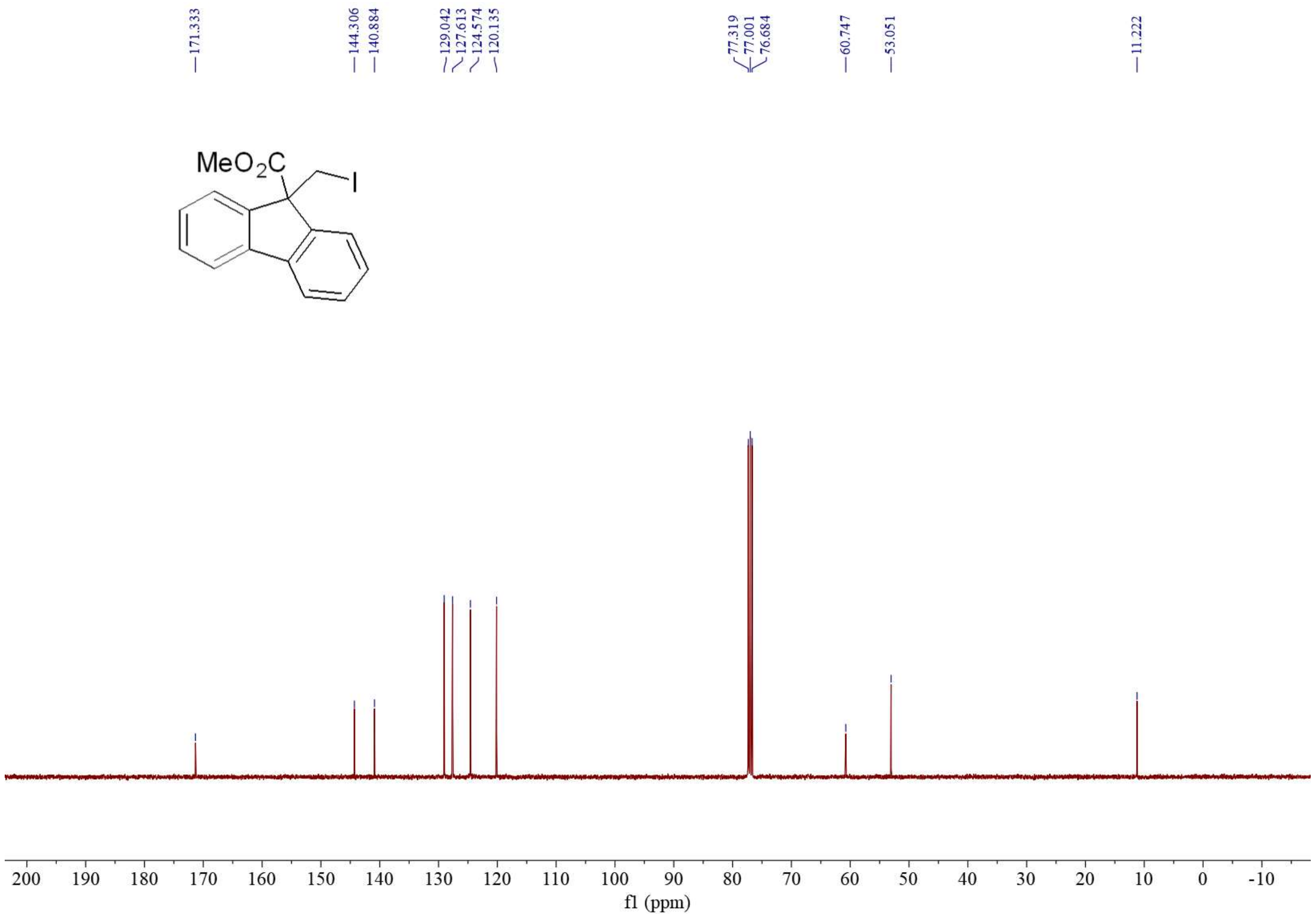

^{1}H NMR spectrum of **5aa** (400 MHz, $CDCl_3$)

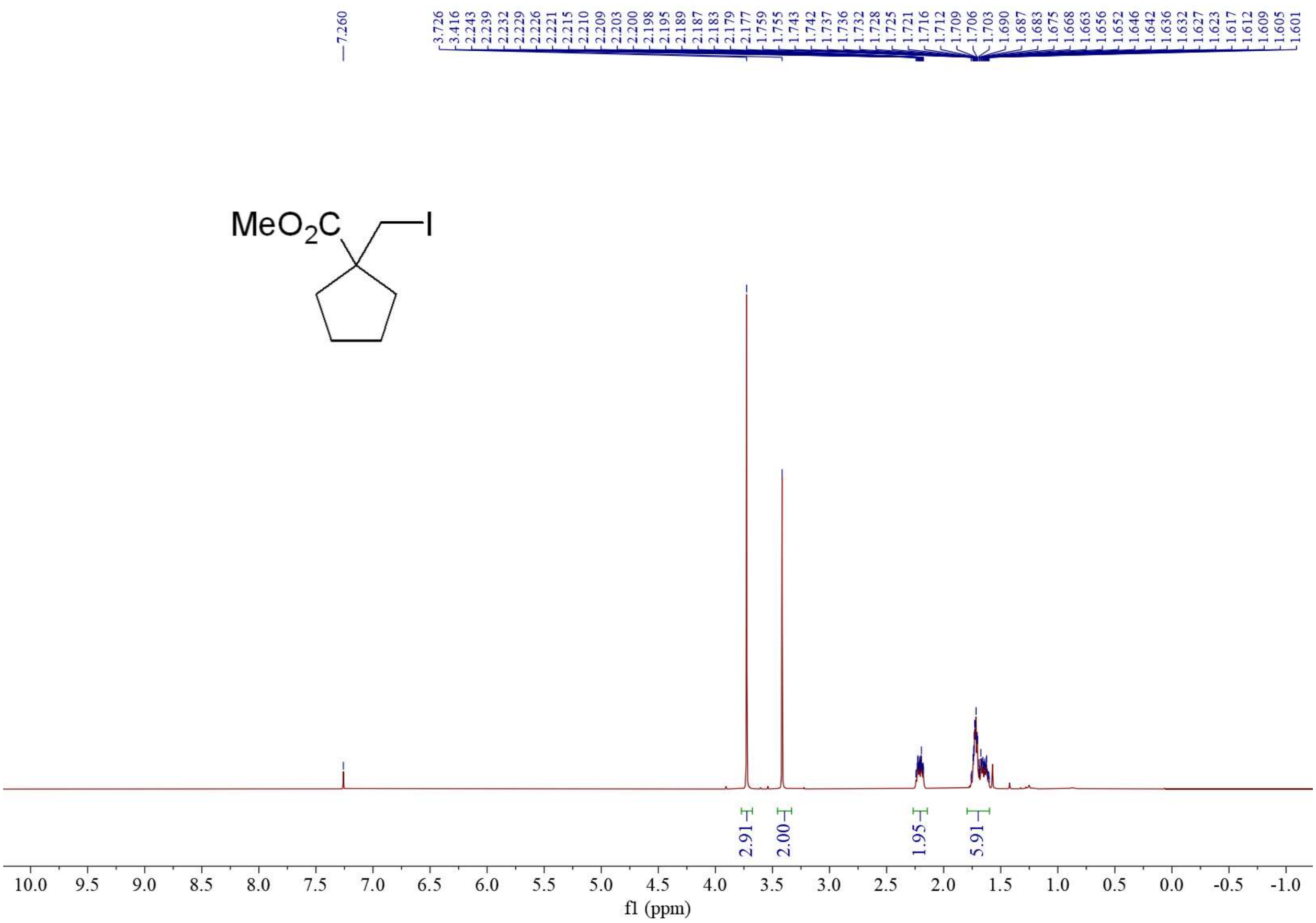

^{13}C NMR spectrum of **5aa** (101 MHz, $CDCl_3$)

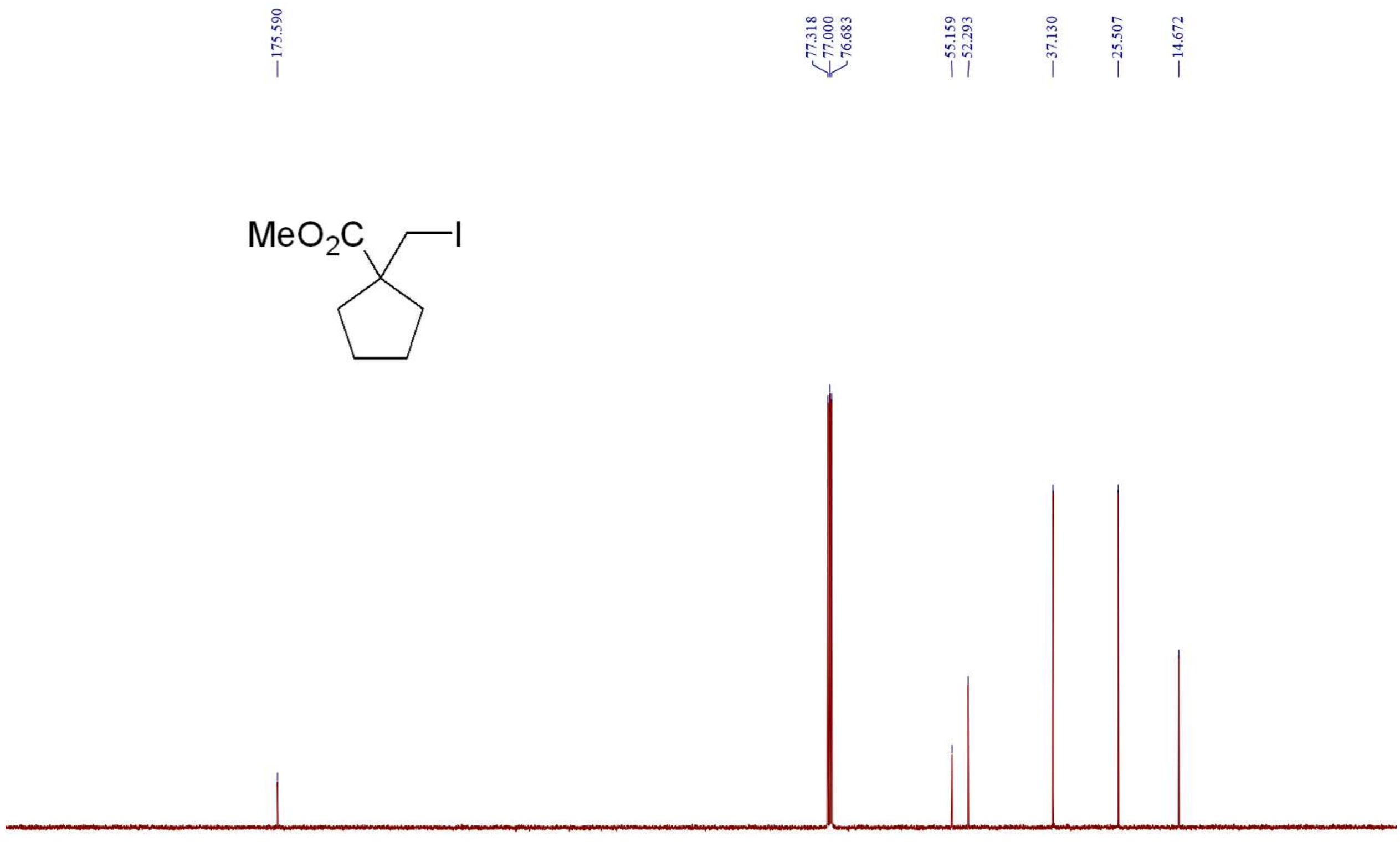

^{1}H NMR spectrum of **5ab** (400 MHz, $CDCl_3$)

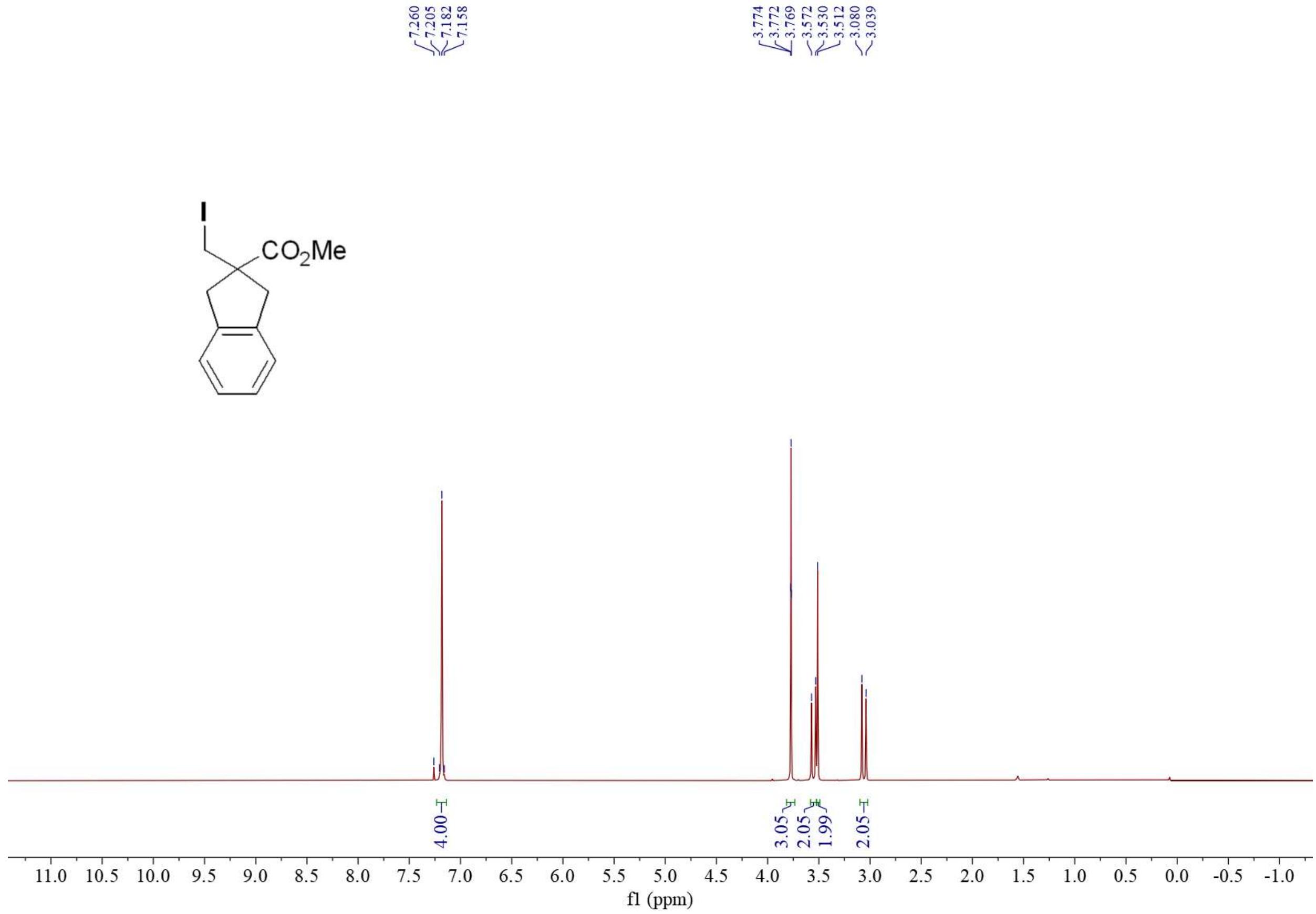

^{13}C NMR spectrum of **5ab** (101 MHz, $CDCl_3$)

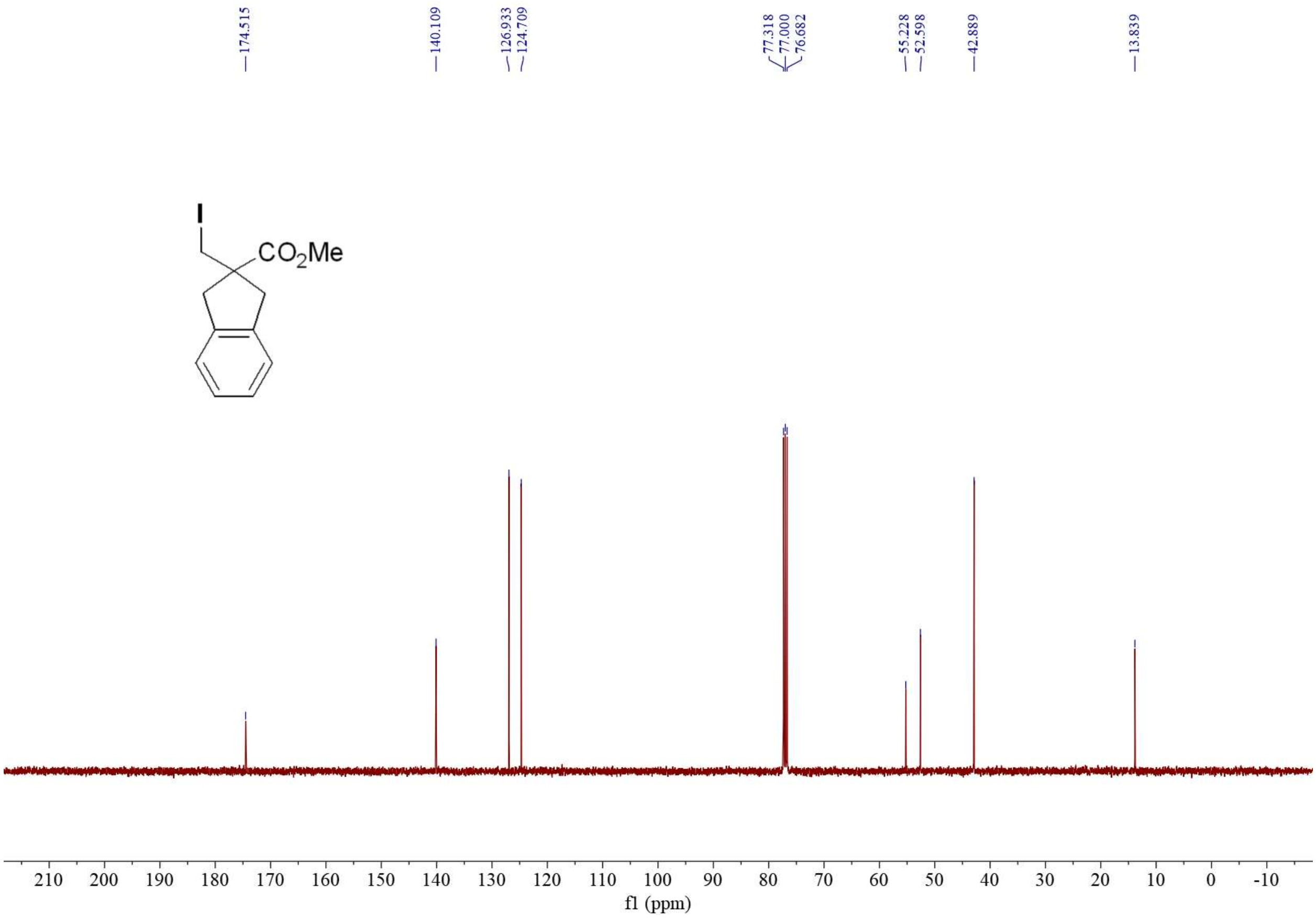

^{1}H NMR spectrum of **6a** (400 MHz, $CDCl_3$)

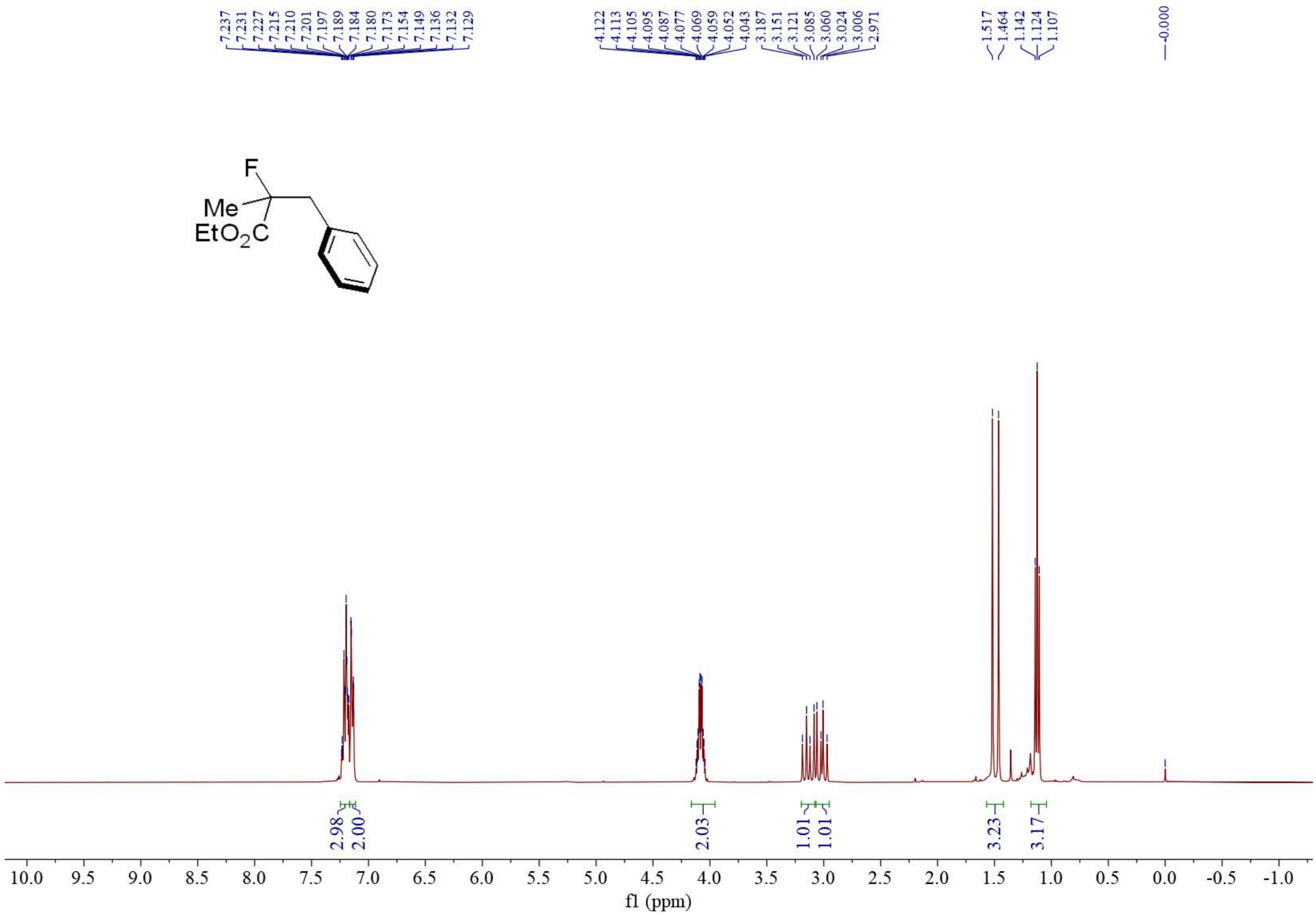

^{13}C NMR spectrum of **6a** (101 MHz, $CDCl_3$)

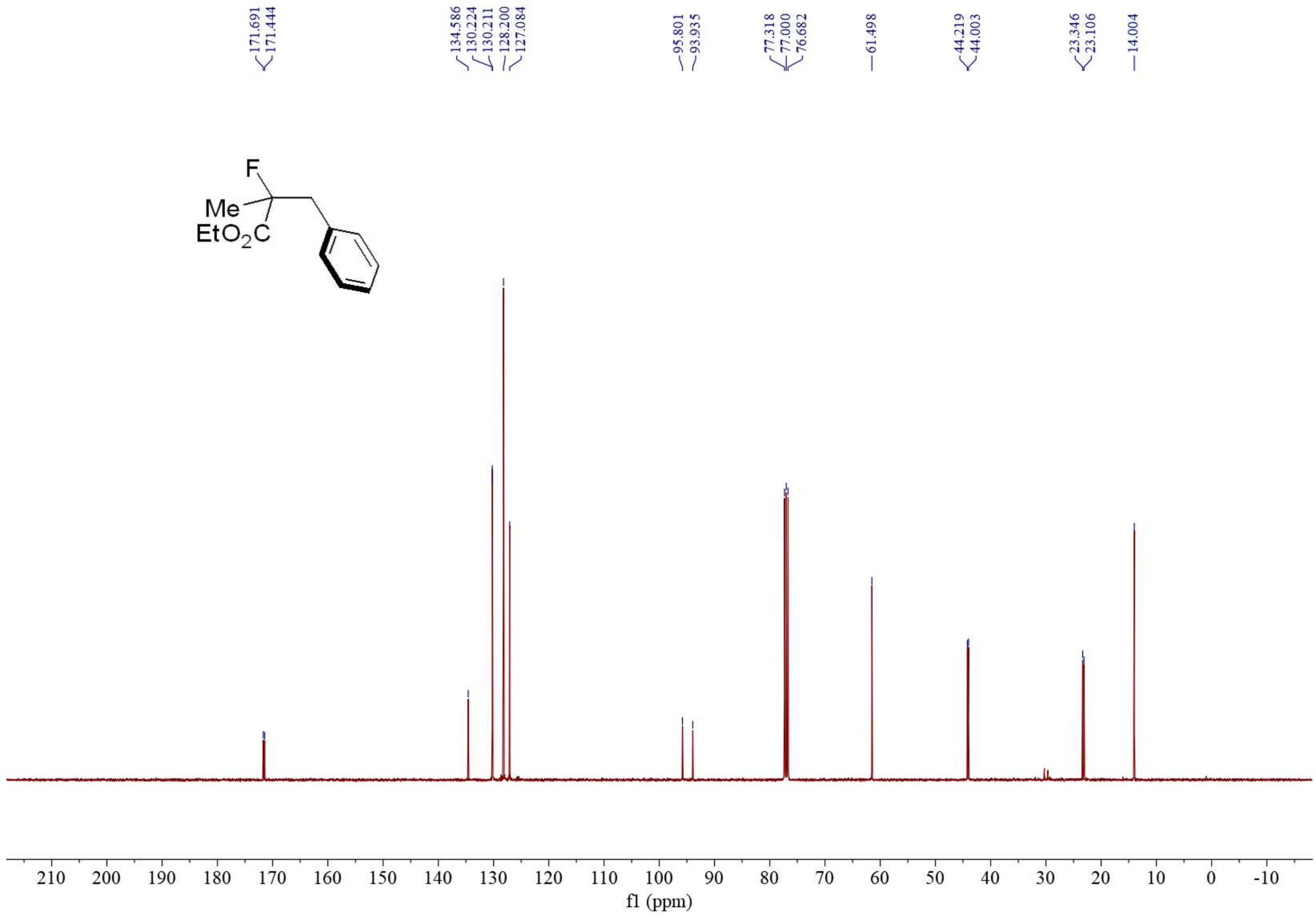

$^{19}F\{^1H\}$ NMR spectrum of **6a** (377 MHz, $CDCl_3$)

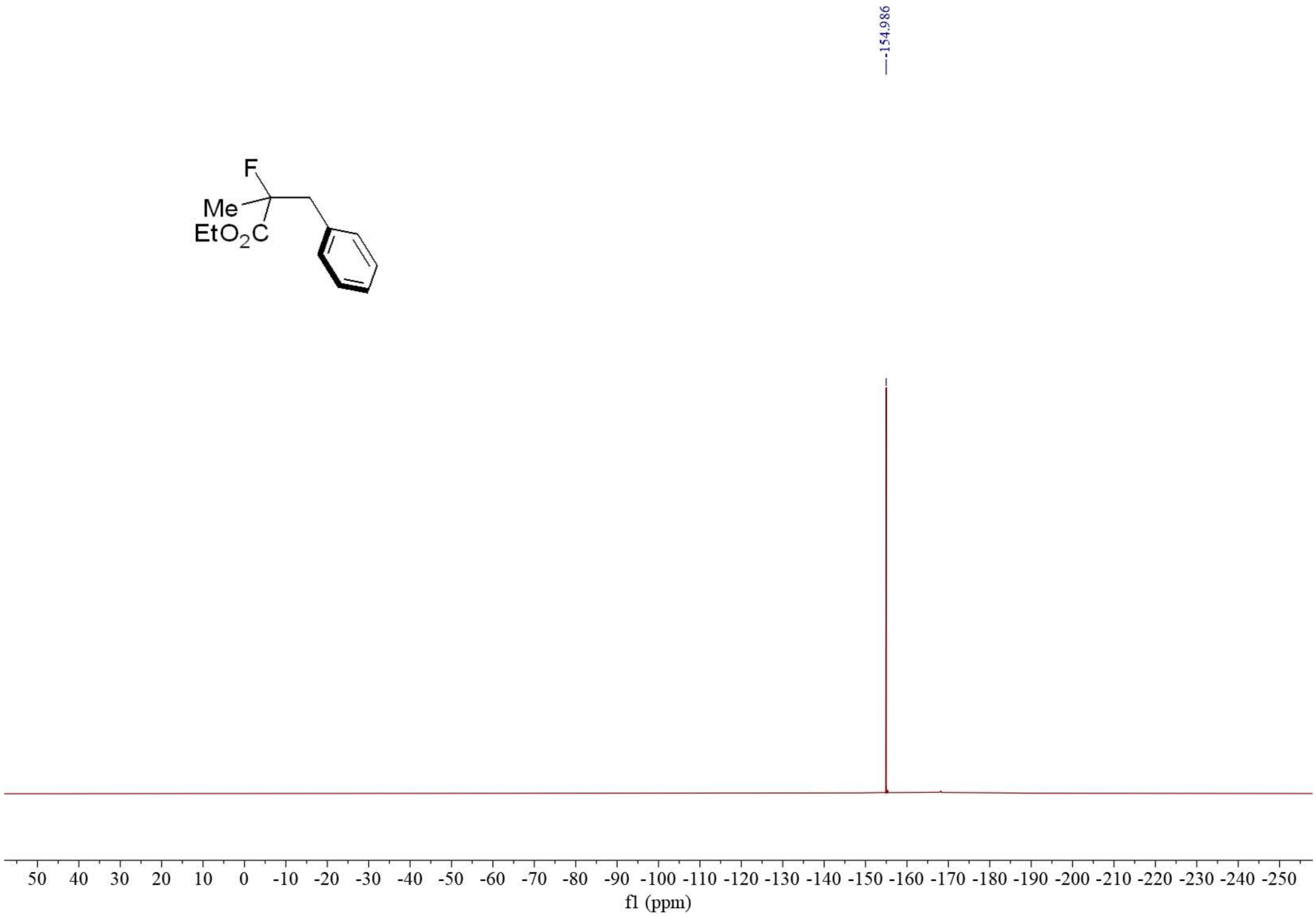

^{19}F NMR spectrum of **6a** (377 MHz, $CDCl_3$)

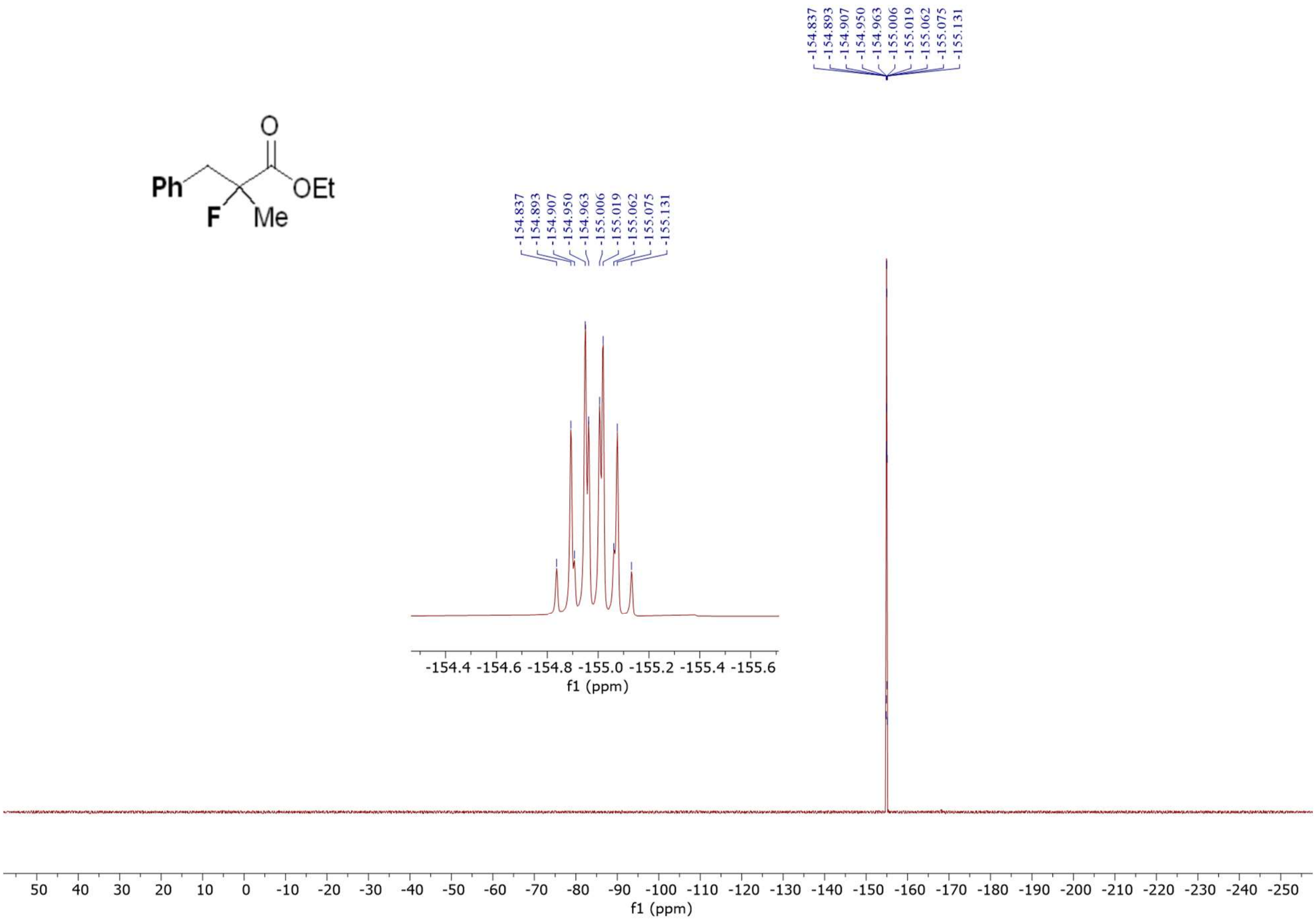

^{1}H NMR spectrum of **6b** (500 MHz, $CDCl_3$)

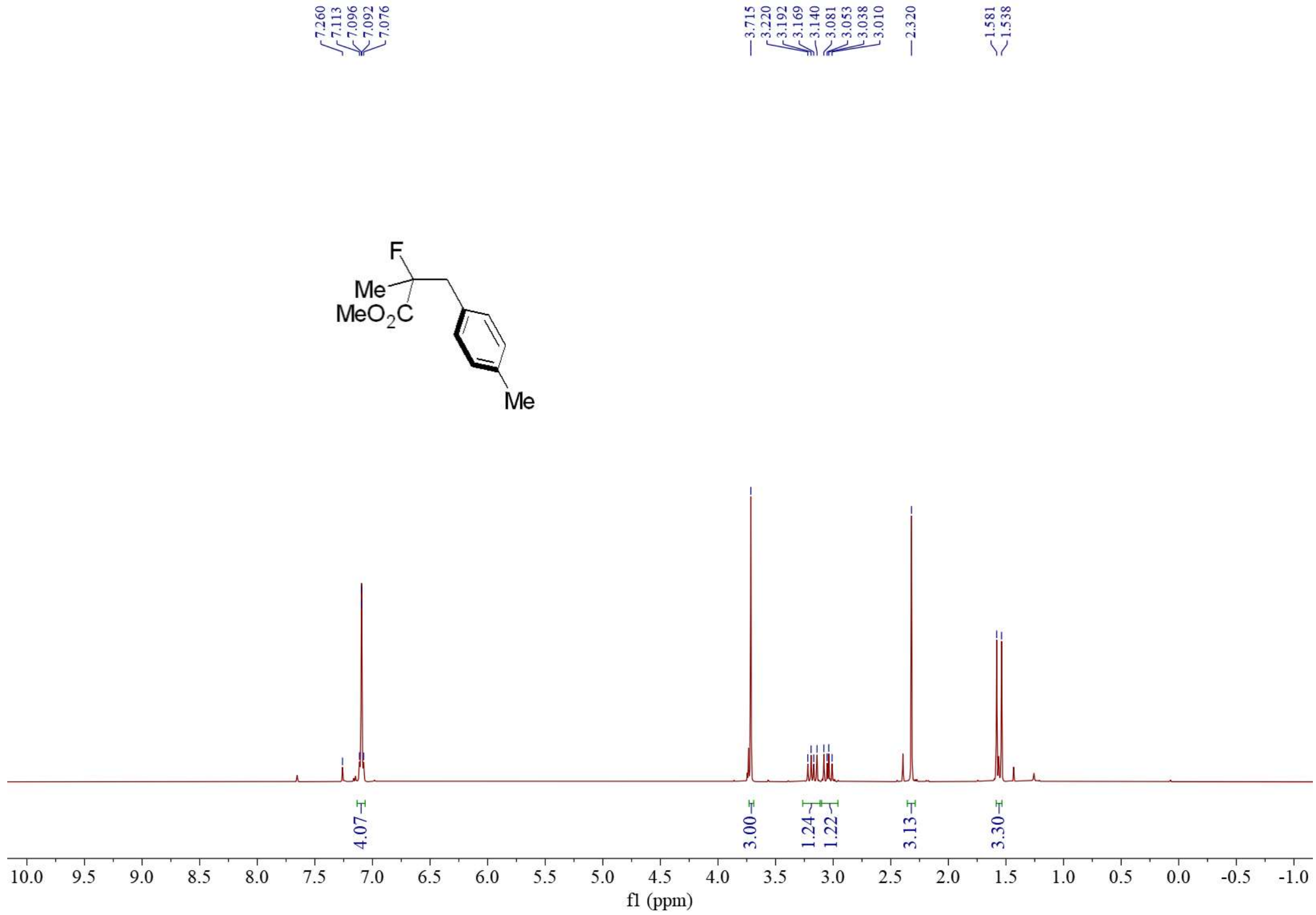

^{13}C NMR spectrum of **6b** (126 MHz, $CDCl_3$)

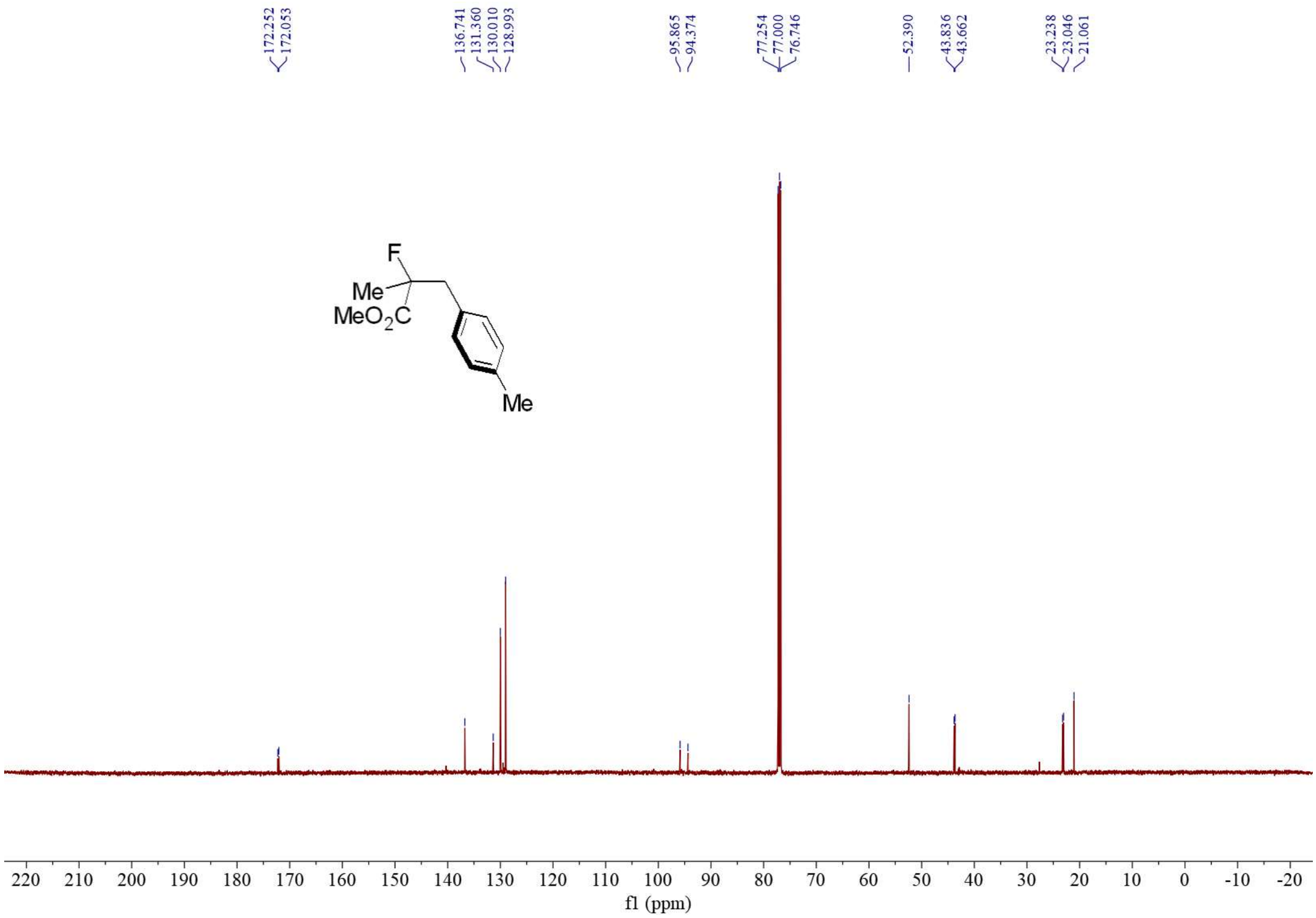

$^{19}F\{^{1}H\}$ NMR spectrum of **6b** (377 MHz, $CDCl_3$)

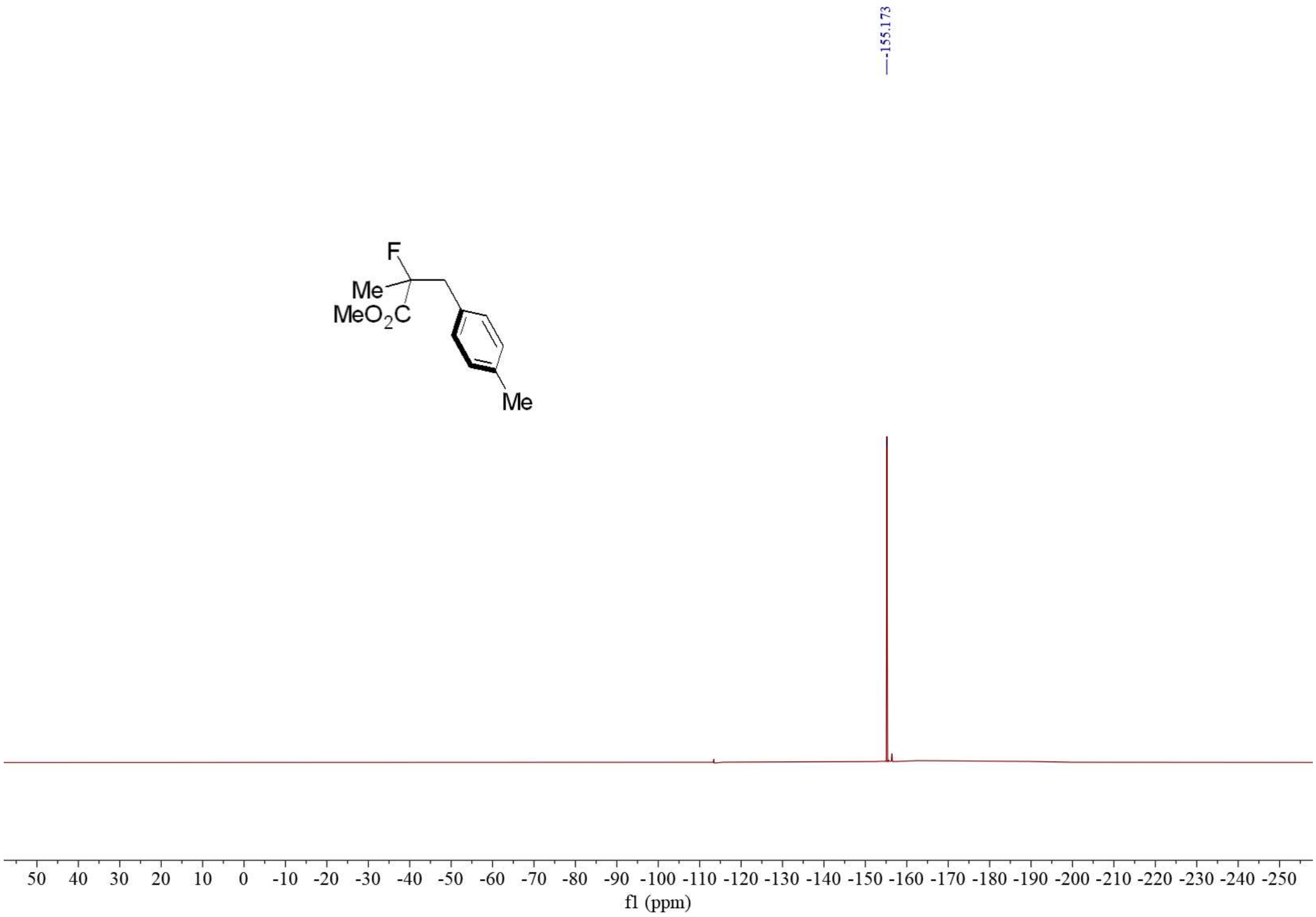

^{19}F NMR spectrum of **6b** (377 MHz, $CDCl_3$)

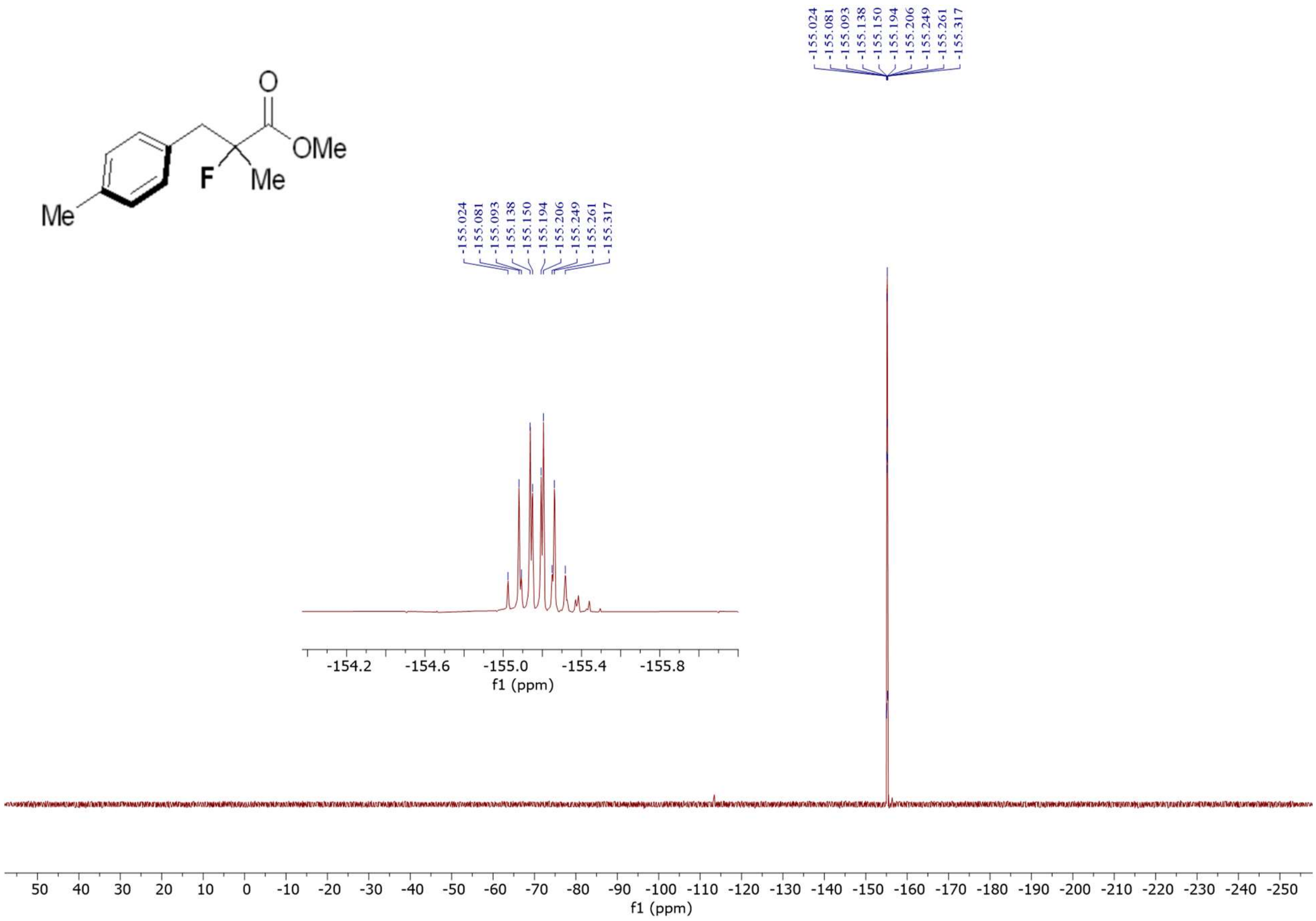

^{1}H NMR spectrum of **6c** (400 MHz, $CDCl_3$)

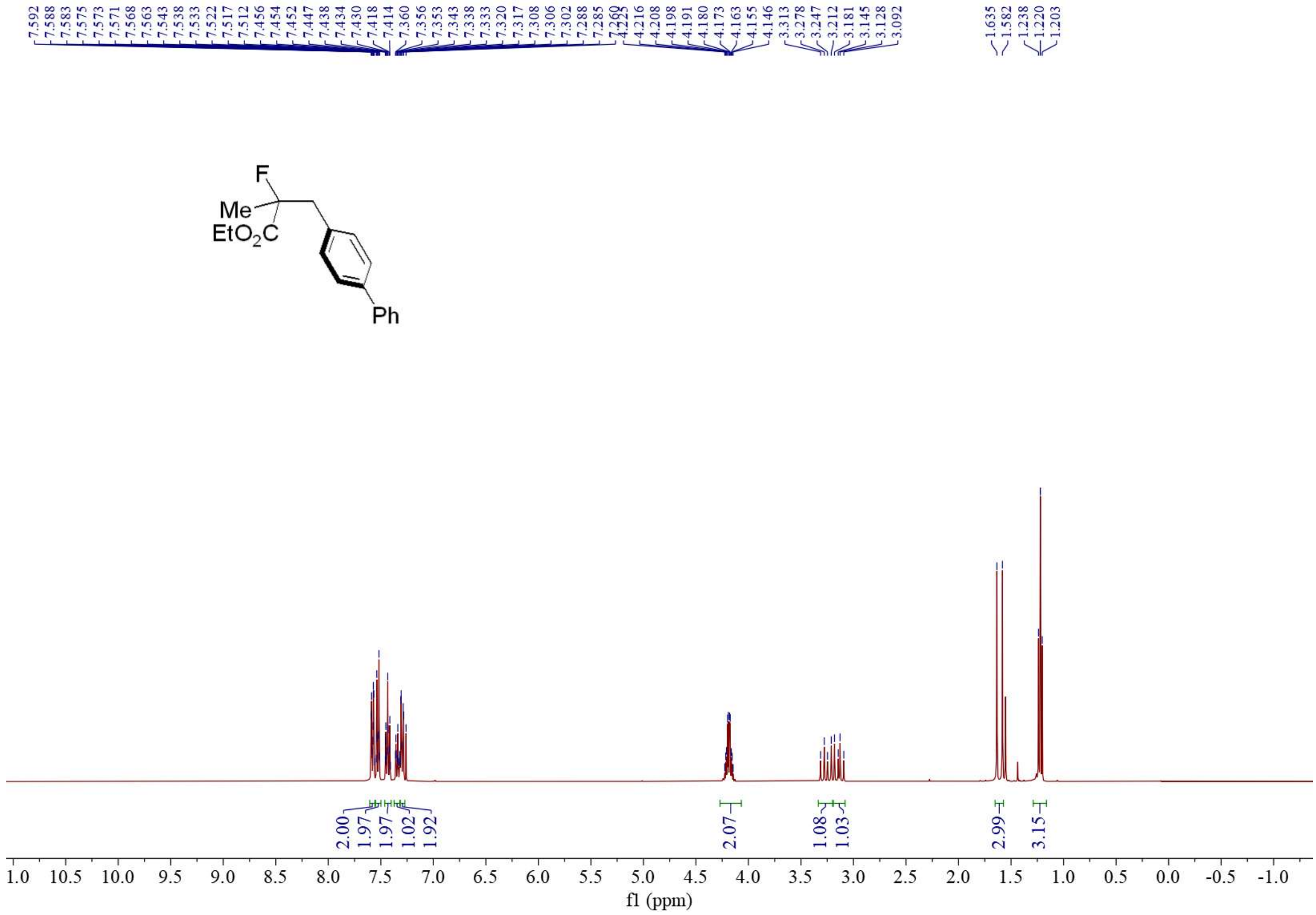

^{13}C NMR spectrum of **6c** (101 MHz, $CDCl_3$)

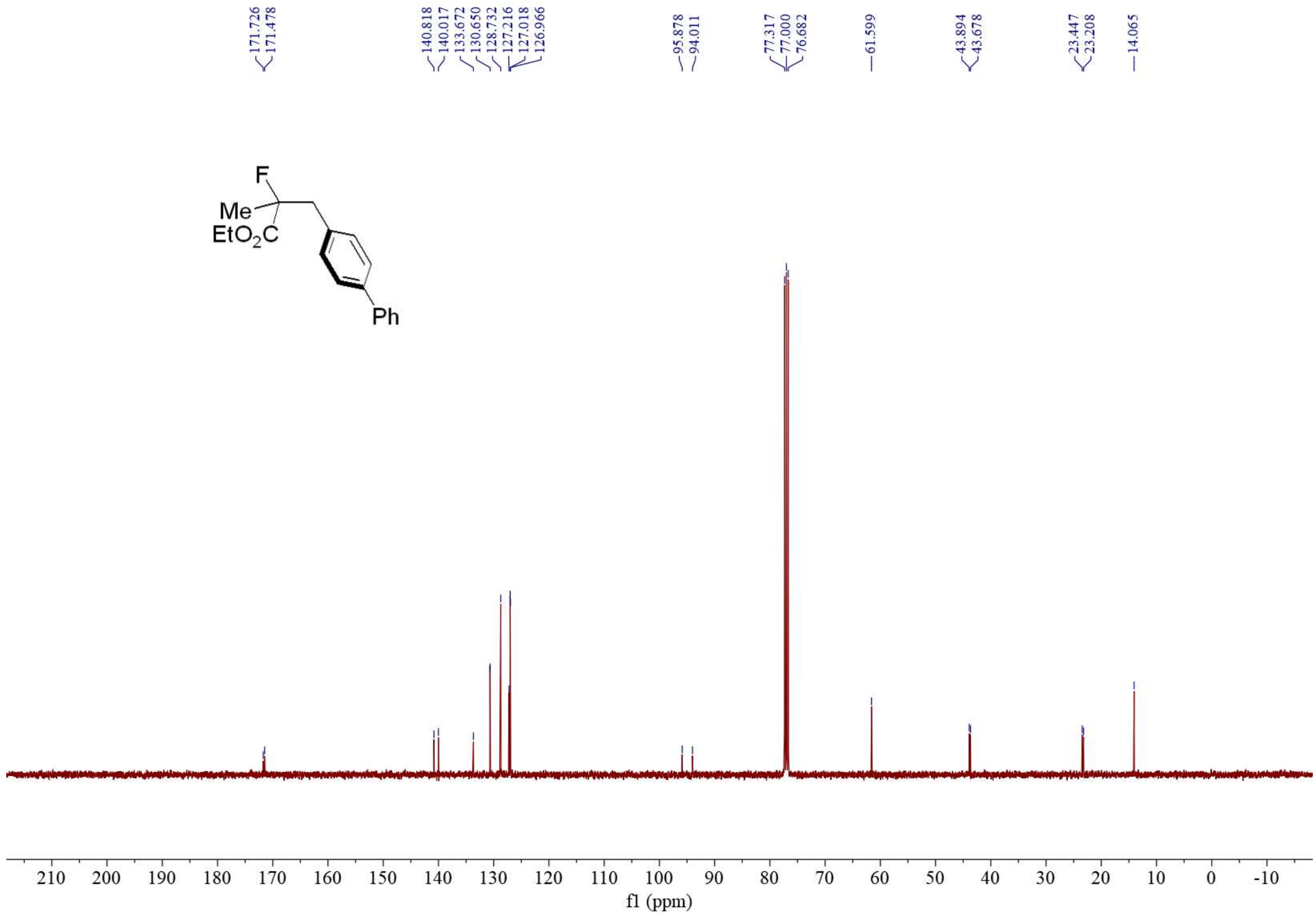

$^{19}F\{^{1}H\}$ NMR spectrum of **6c** (377 MHz, $CDCl_3$)

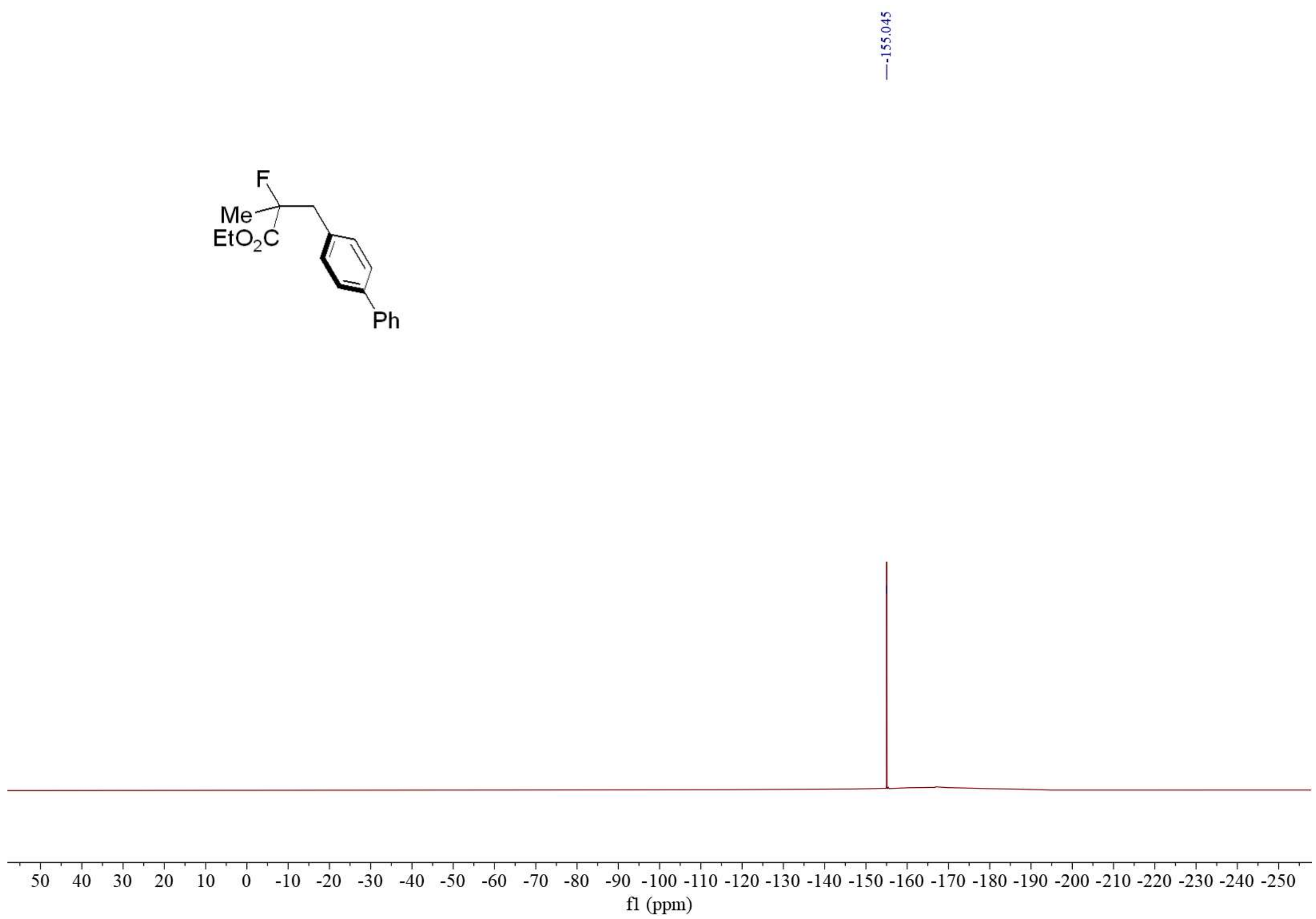

^{19}F NMR spectrum of **6c** (377 MHz, $CDCl_3$)

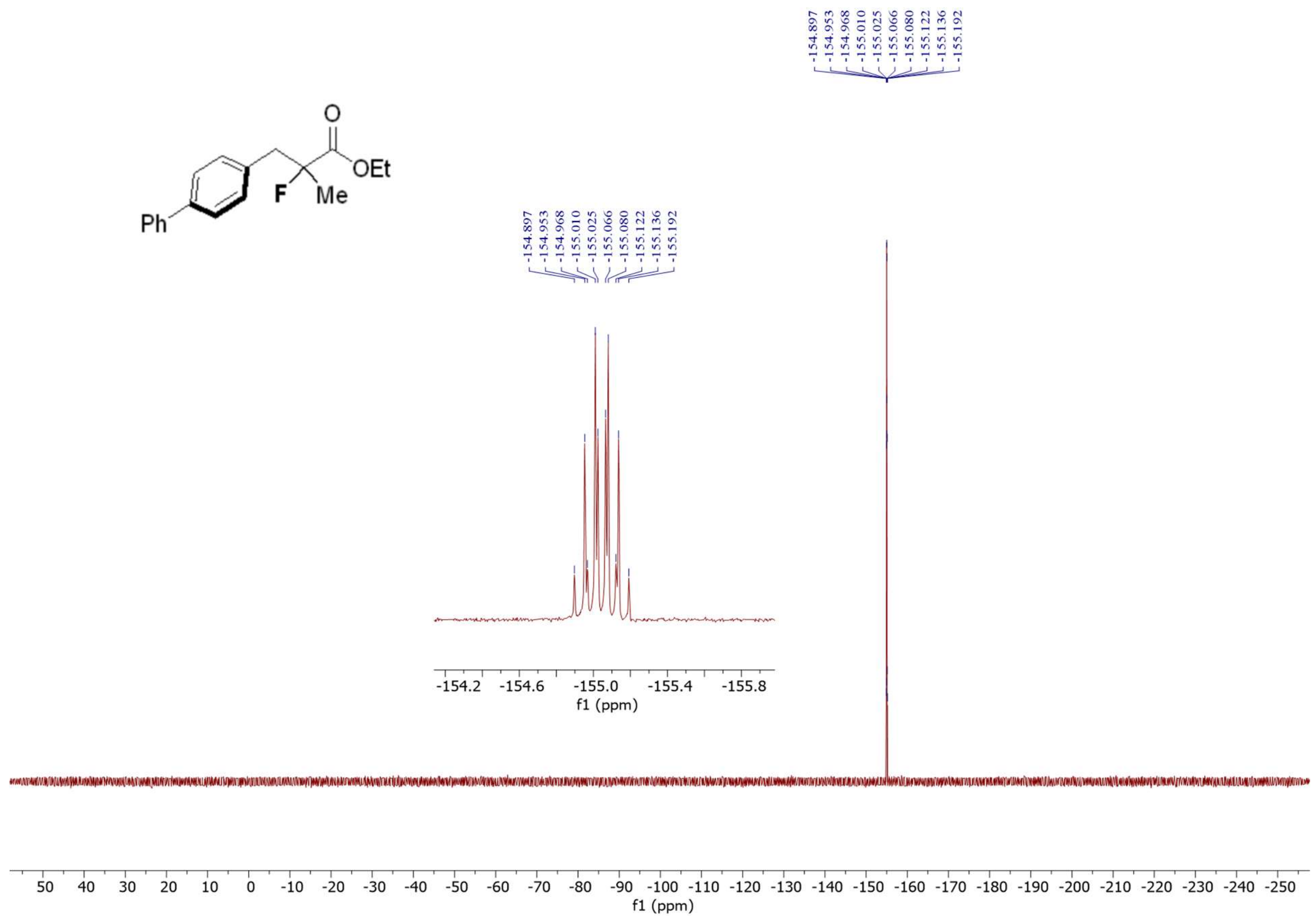

^{1}H NMR spectrum of **6d** (400 MHz, $CDCl_3$)

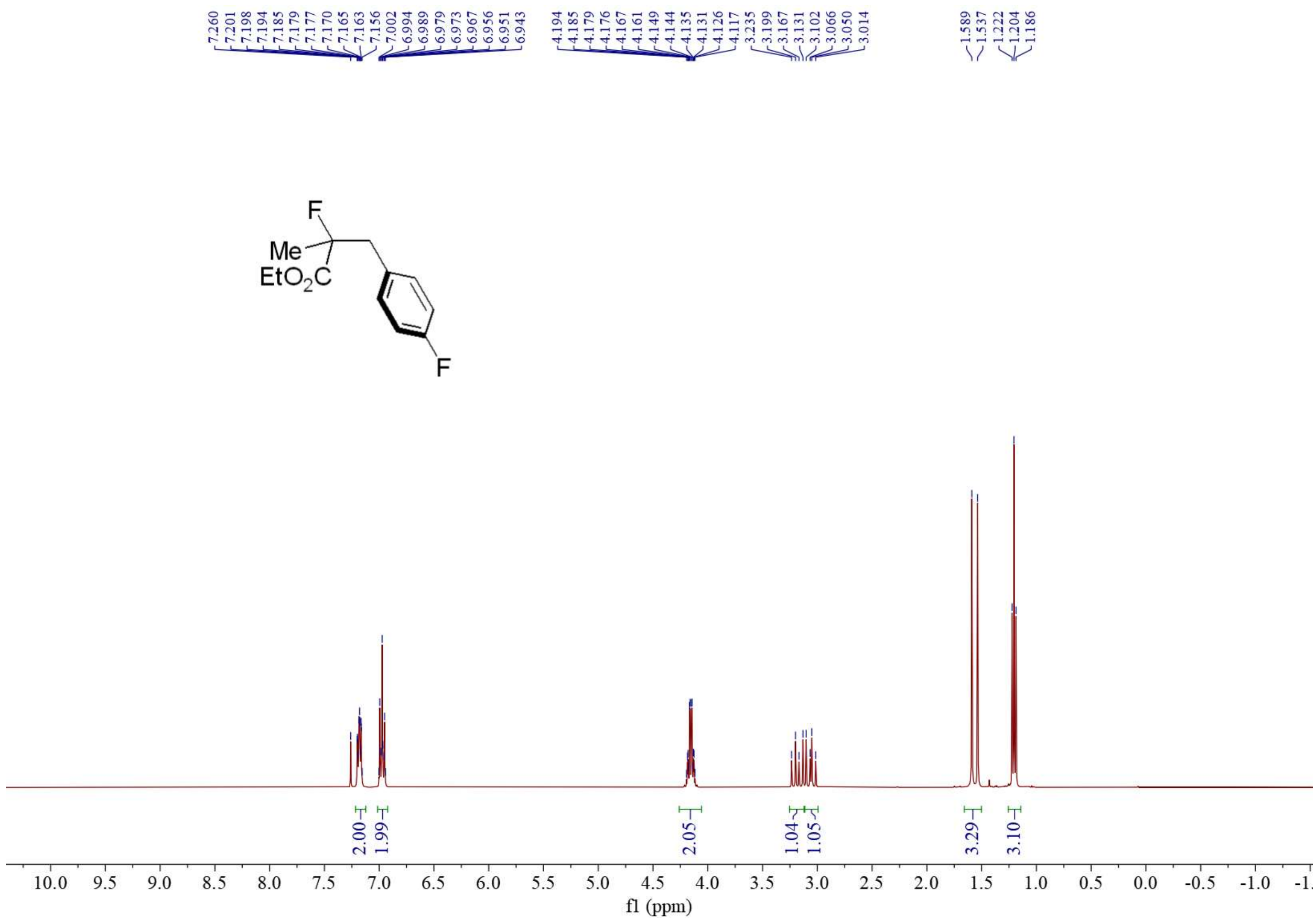

^{13}C NMR spectrum of **6d** (101 MHz, $CDCl_3$)

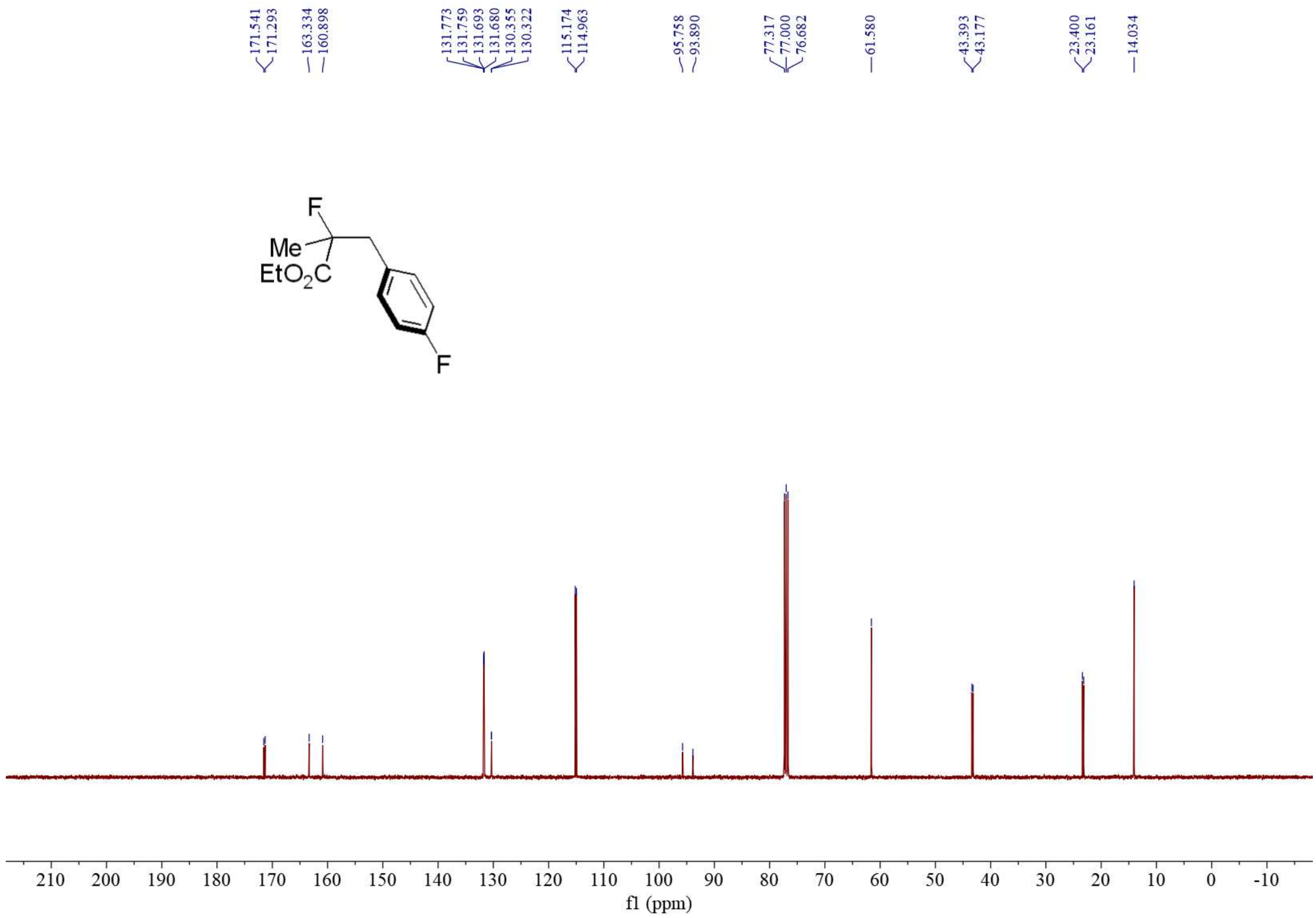

$^{19}F\{^{1}H\}$ NMR spectrum of **6d** (377 MHz, $CDCl_3$)

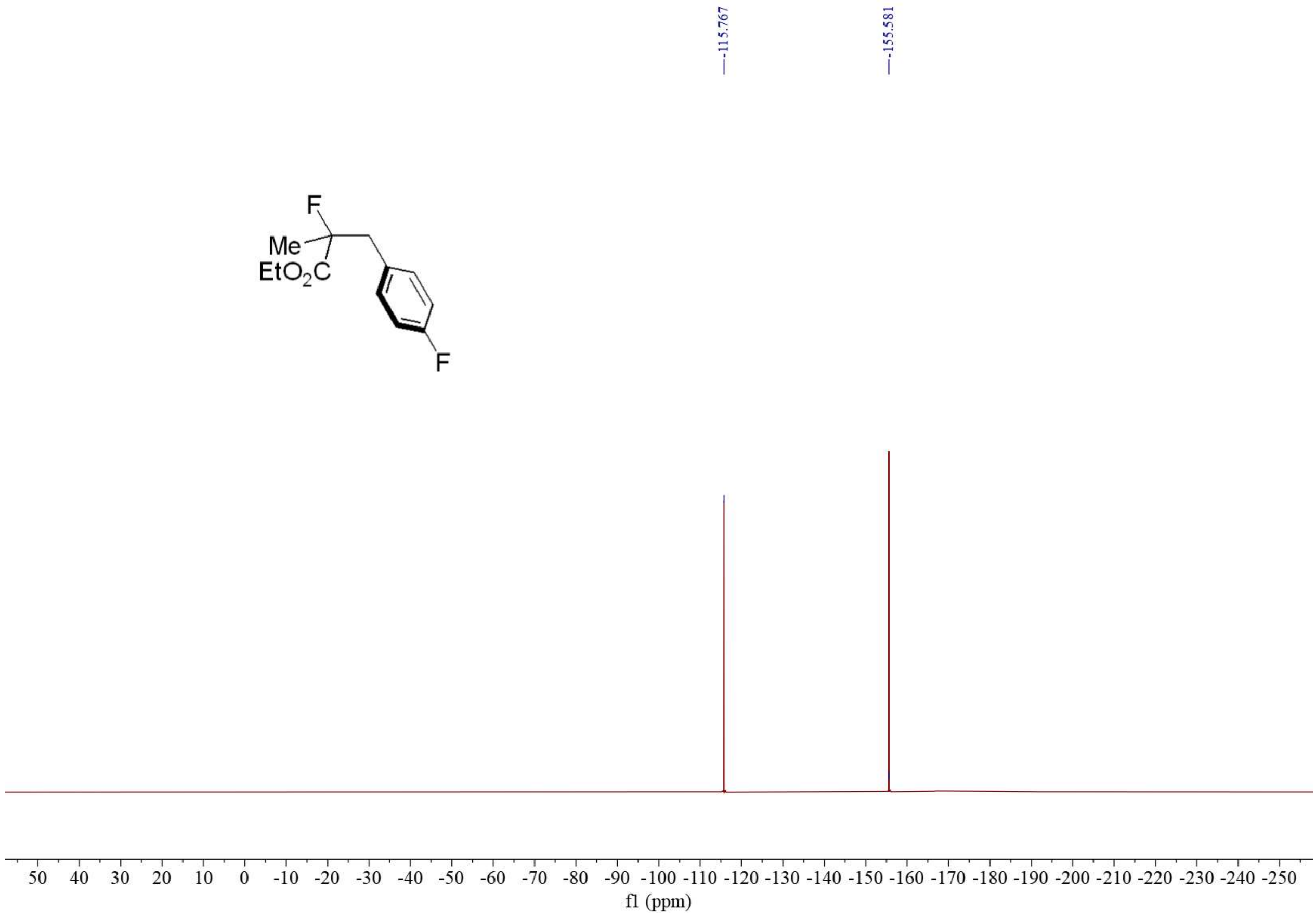

^{19}F NMR spectrum of **6d** (377 MHz, $CDCl_3$)

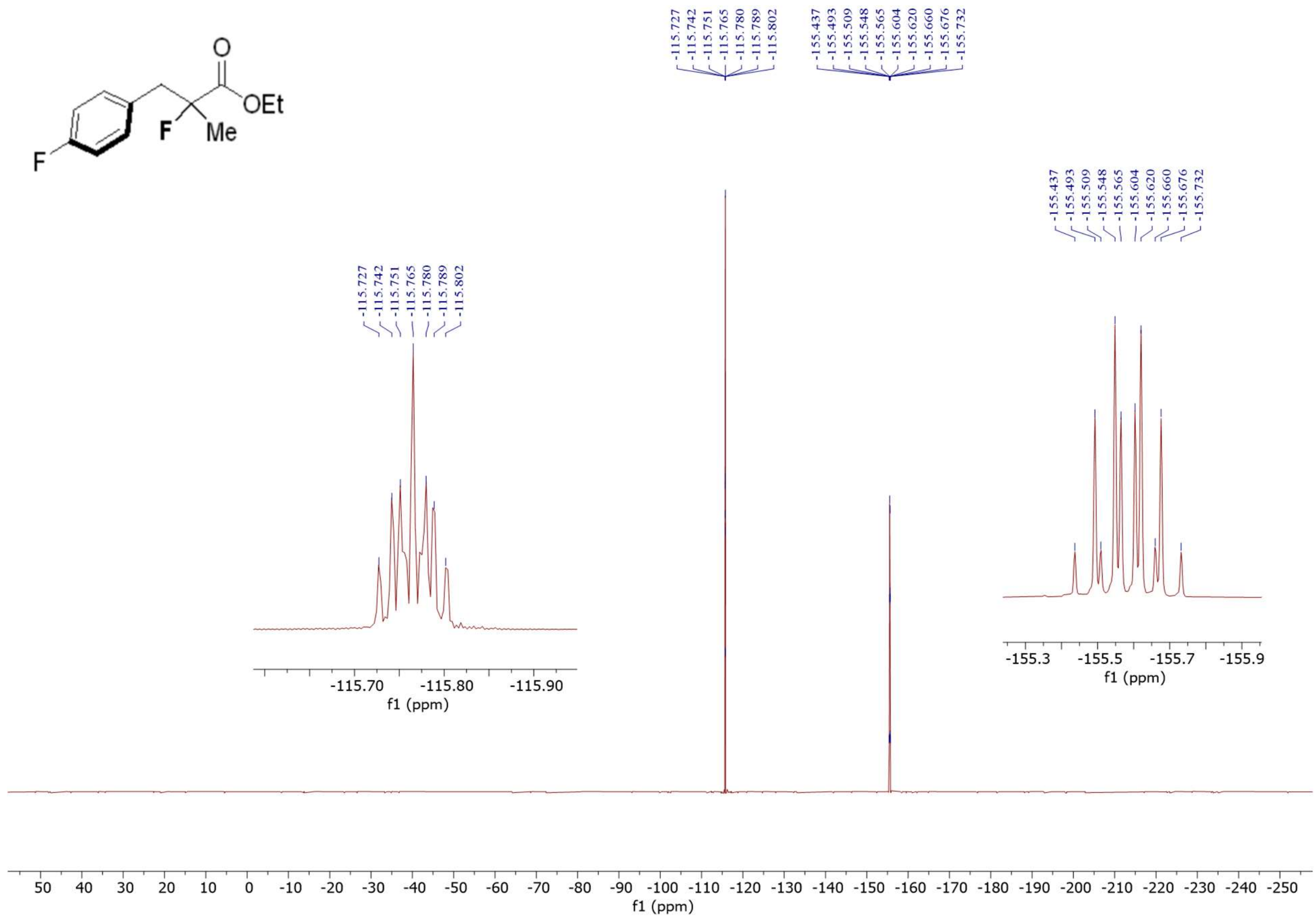

^{1}H NMR spectrum of **6e** (400 MHz, $CDCl_3$)

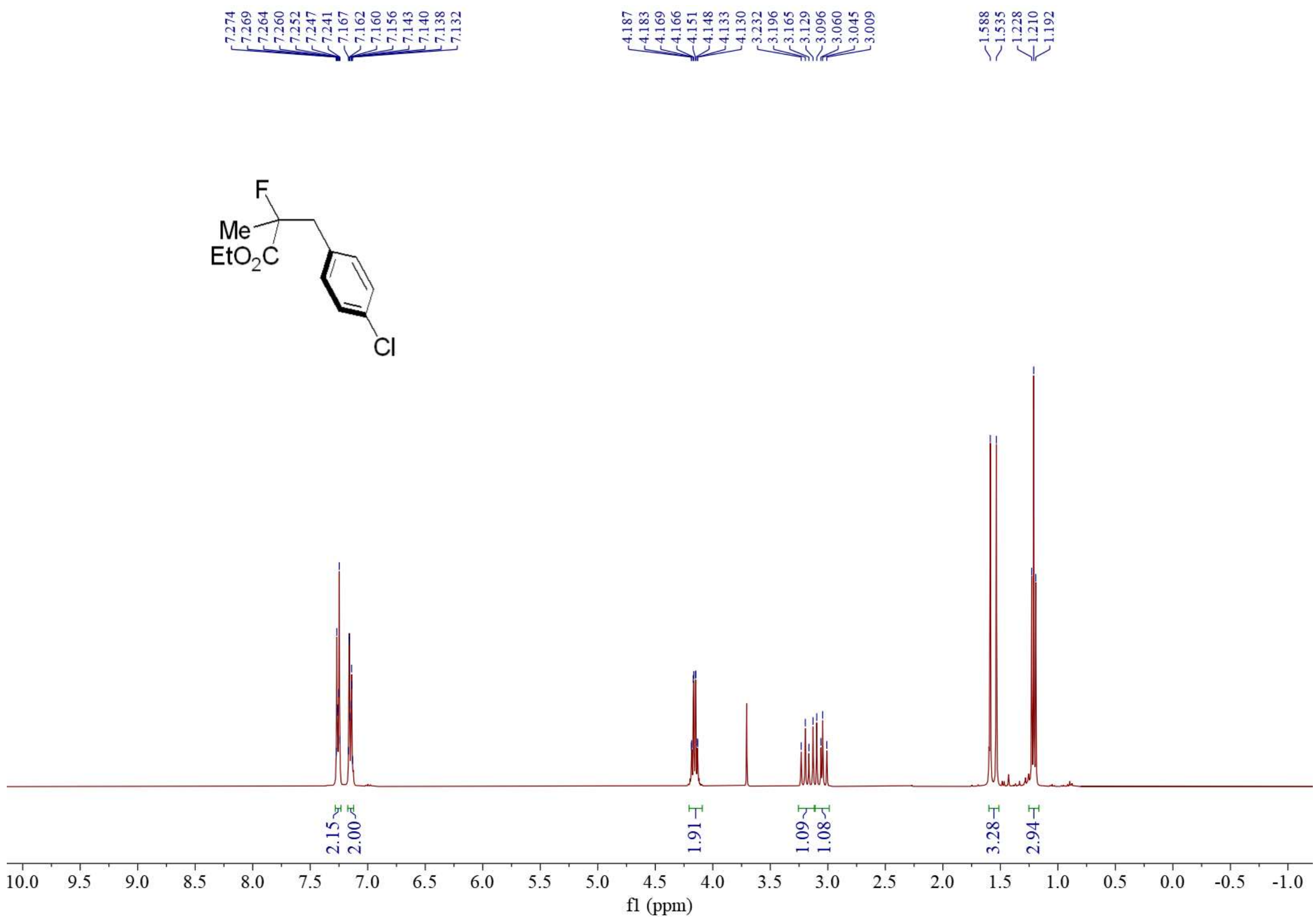

^{13}C NMR spectrum of **6e** (101 MHz, $CDCl_3$)

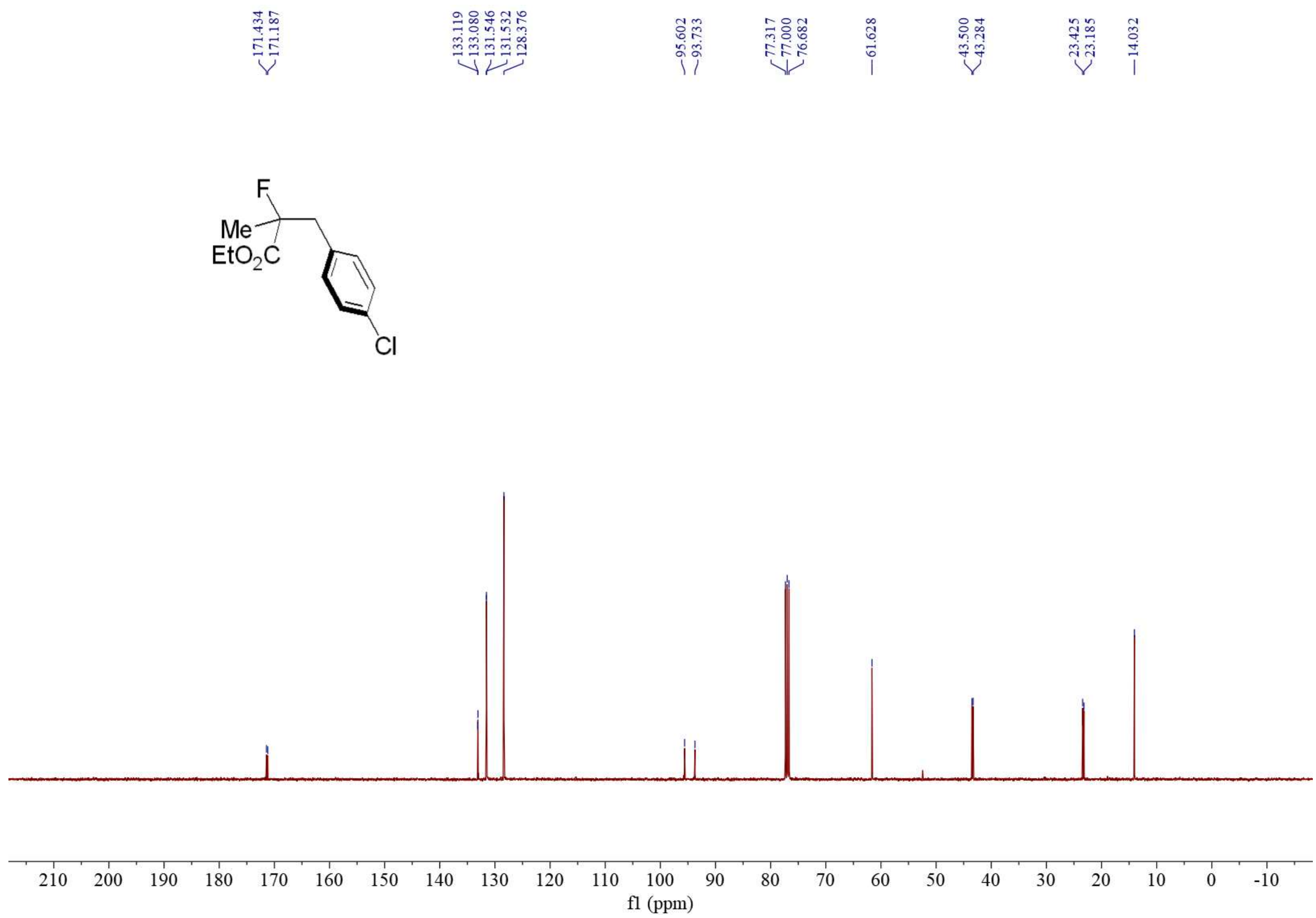

$^{19}F\{^{1}H\}$ NMR spectrum of **6e** (377 MHz, $CDCl_3$)

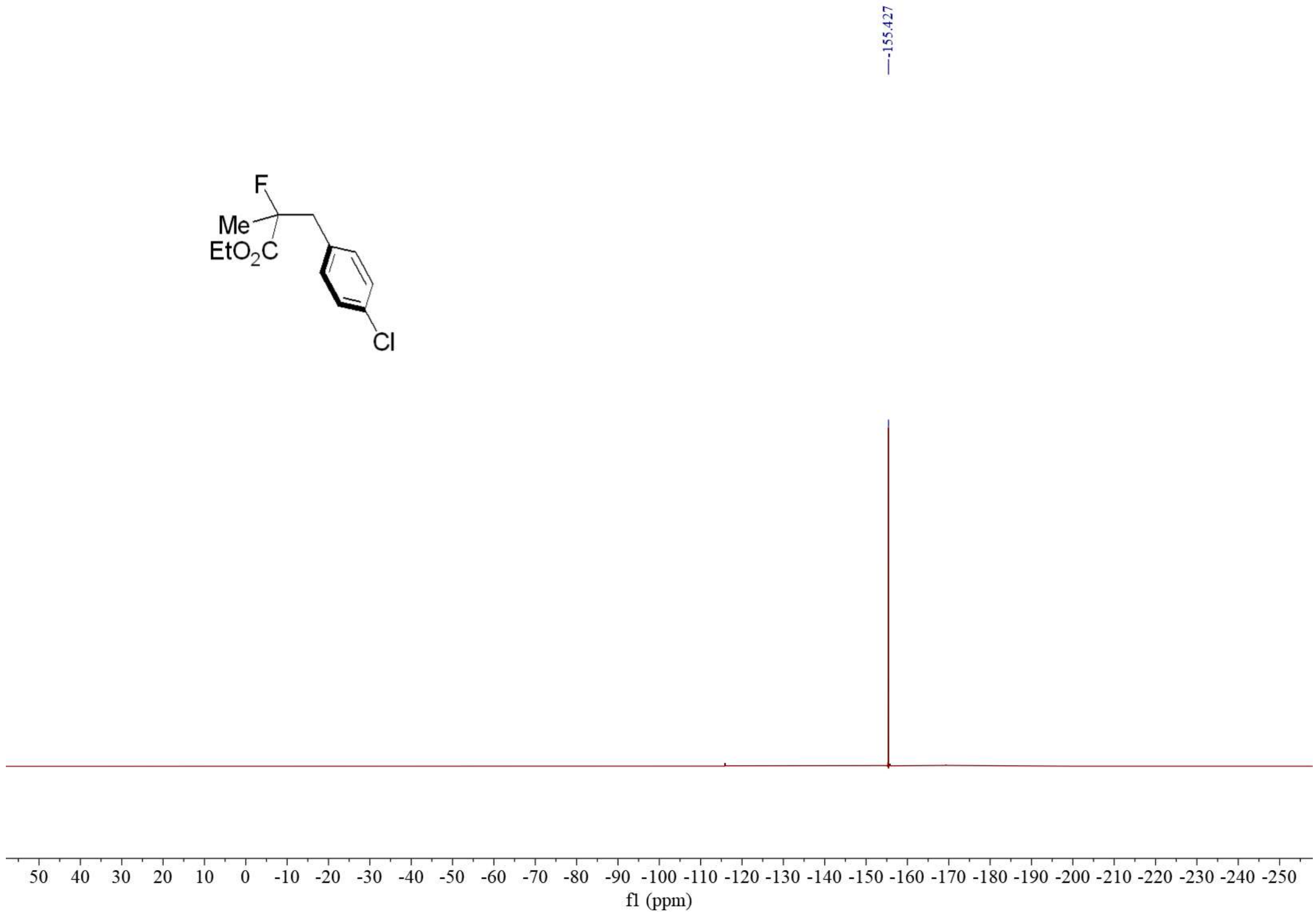

^{19}F NMR spectrum of **6e** (377 MHz, $CDCl_3$)

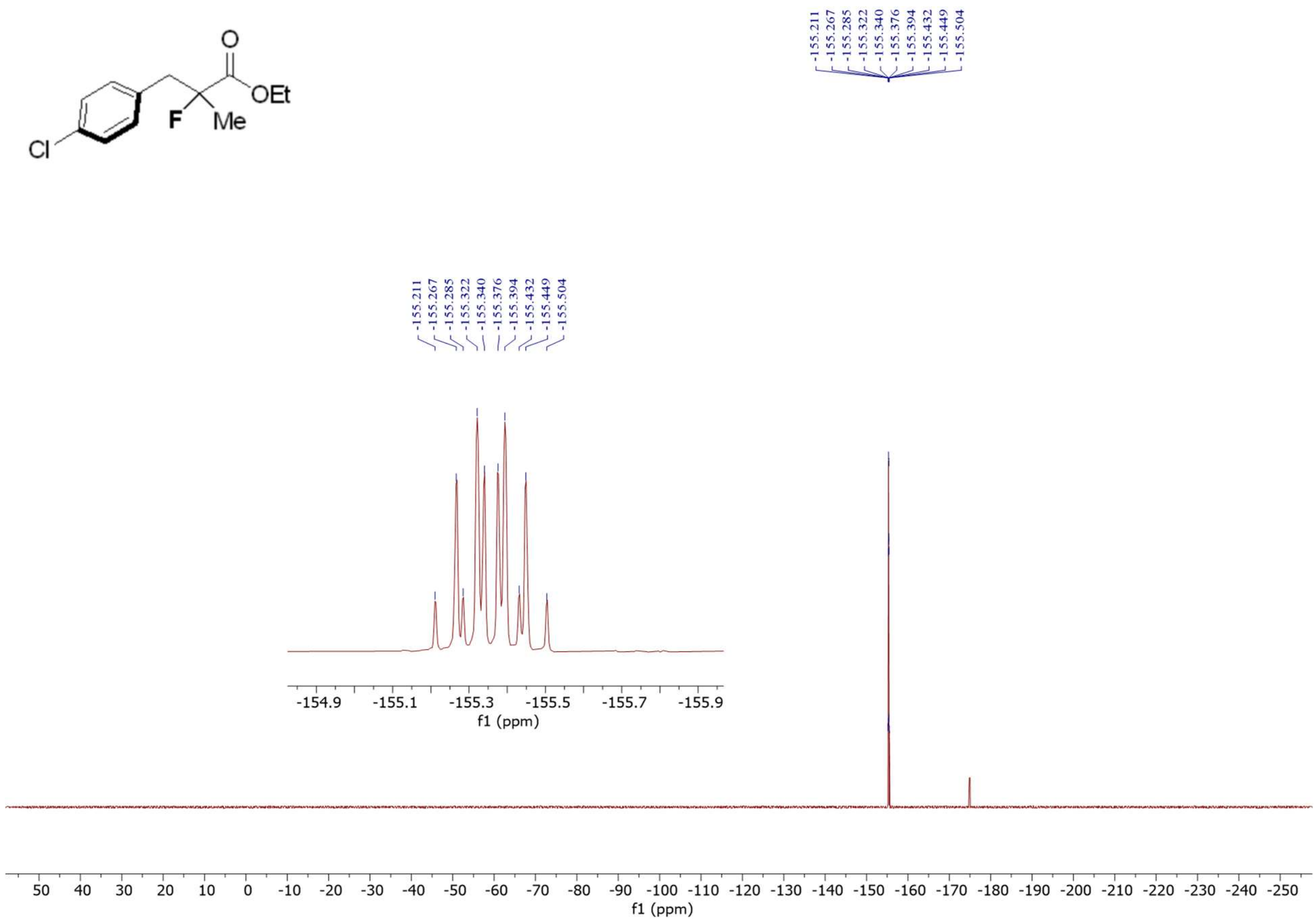

^{1}H NMR spectrum of **6f** (400 MHz, $CDCl_3$)

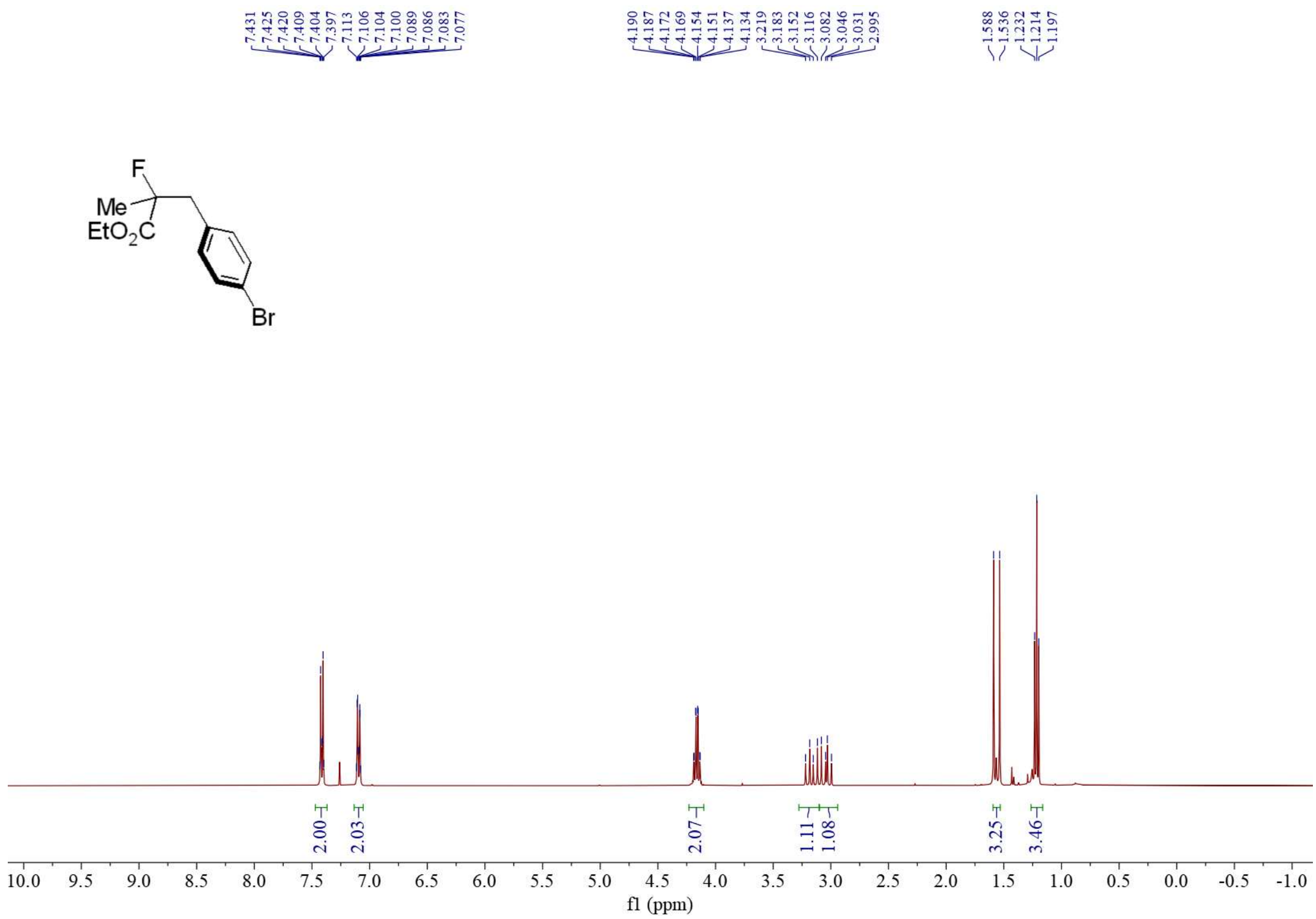

^{13}C NMR spectrum of **6f** (101 MHz, $CDCl_3$)

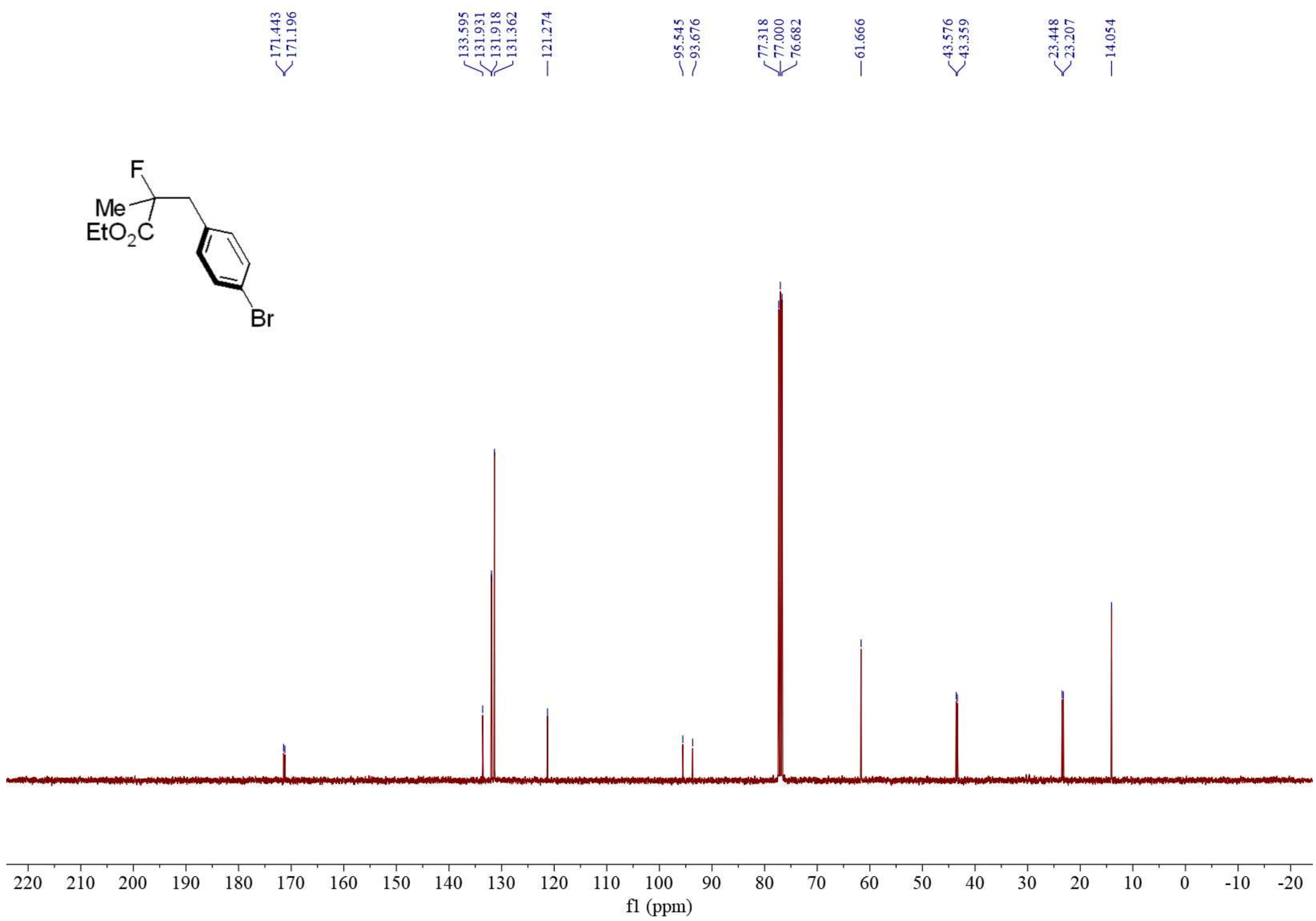

$^{19}F\{^1H\}$ NMR spectrum of **6f** (377 MHz, $CDCl_3$)

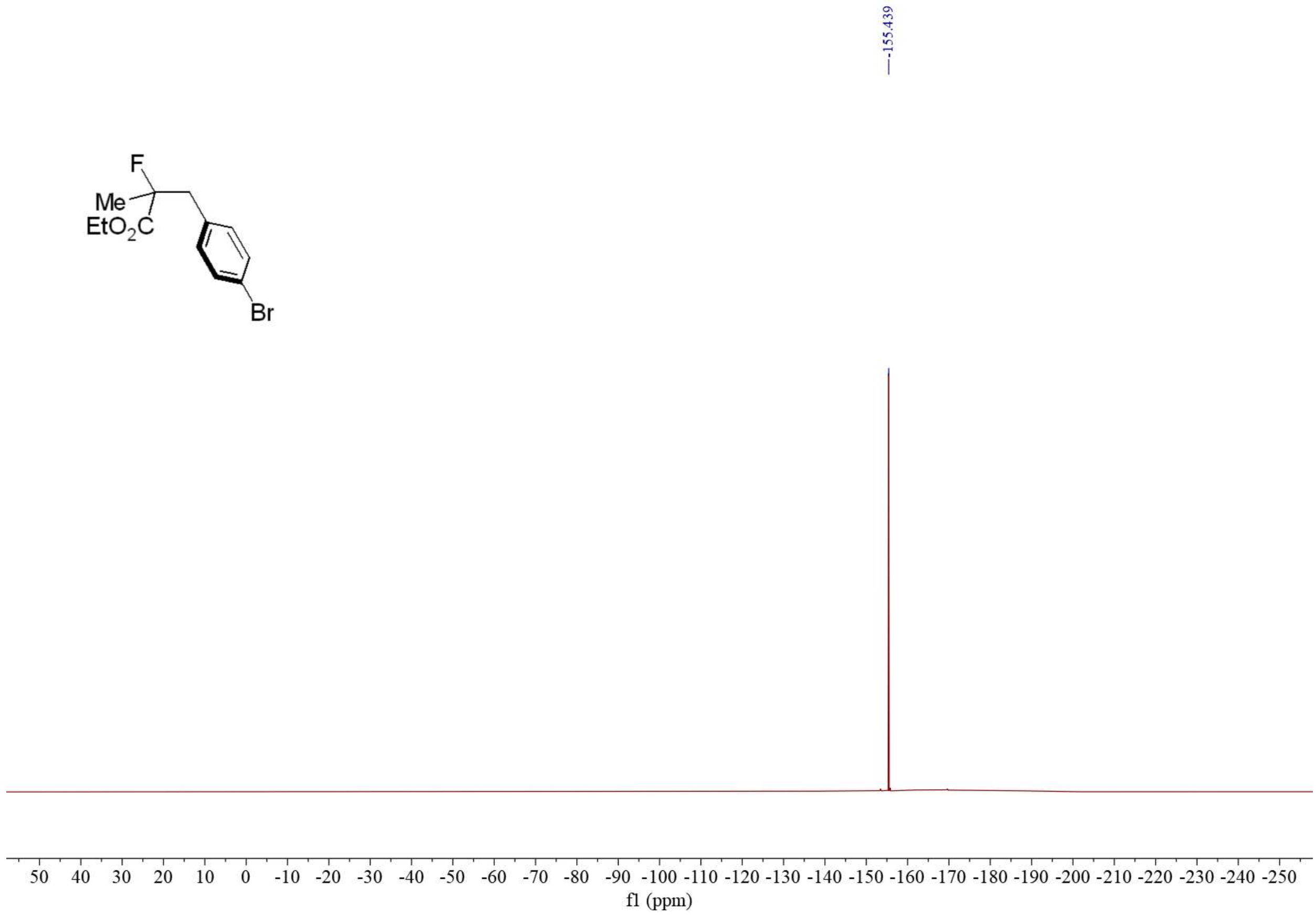

^{19}F NMR spectrum of **6f** (377 MHz, $CDCl_3$)

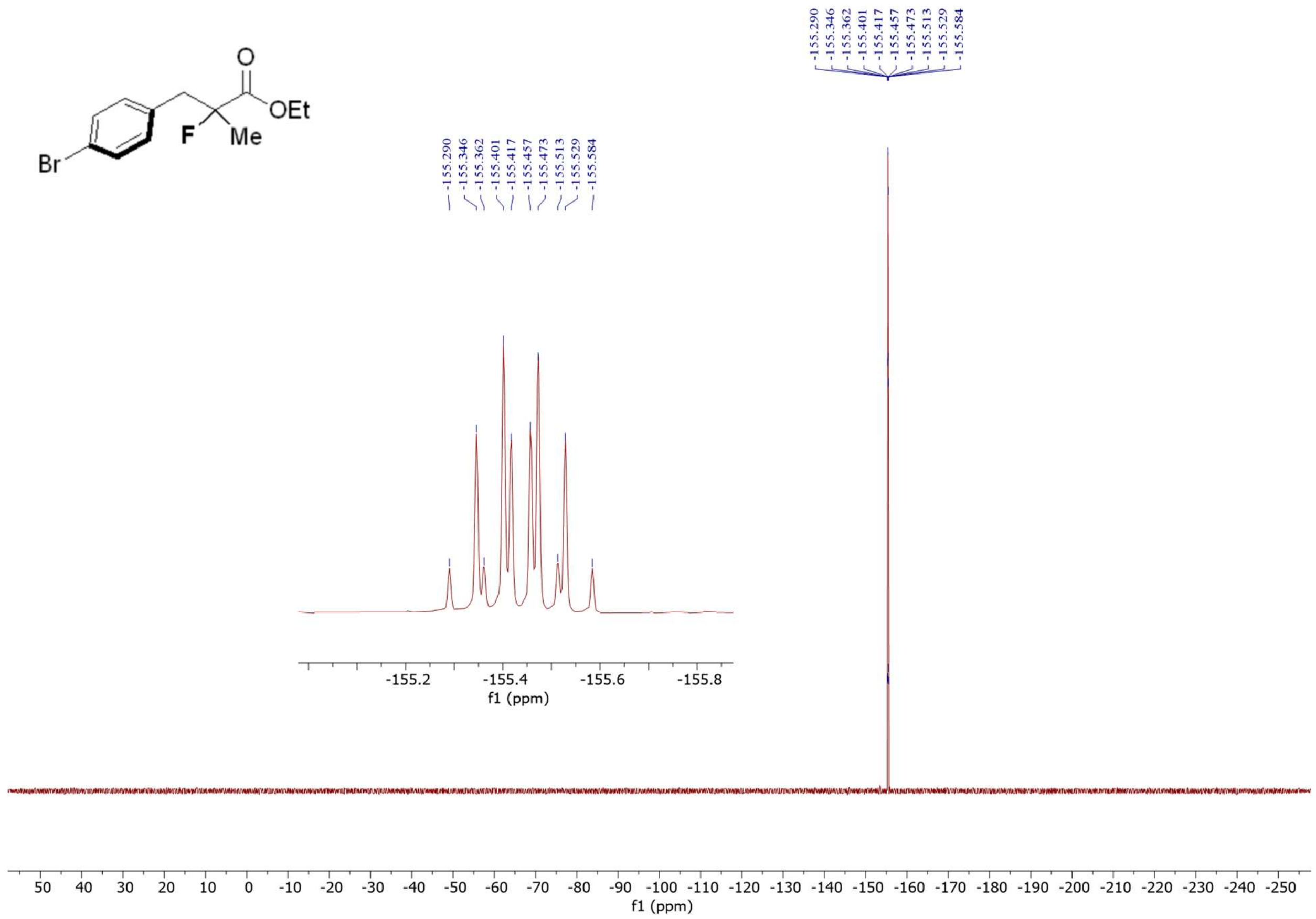

^{1}H NMR spectrum of **6g** (400 MHz, $CDCl_3$)

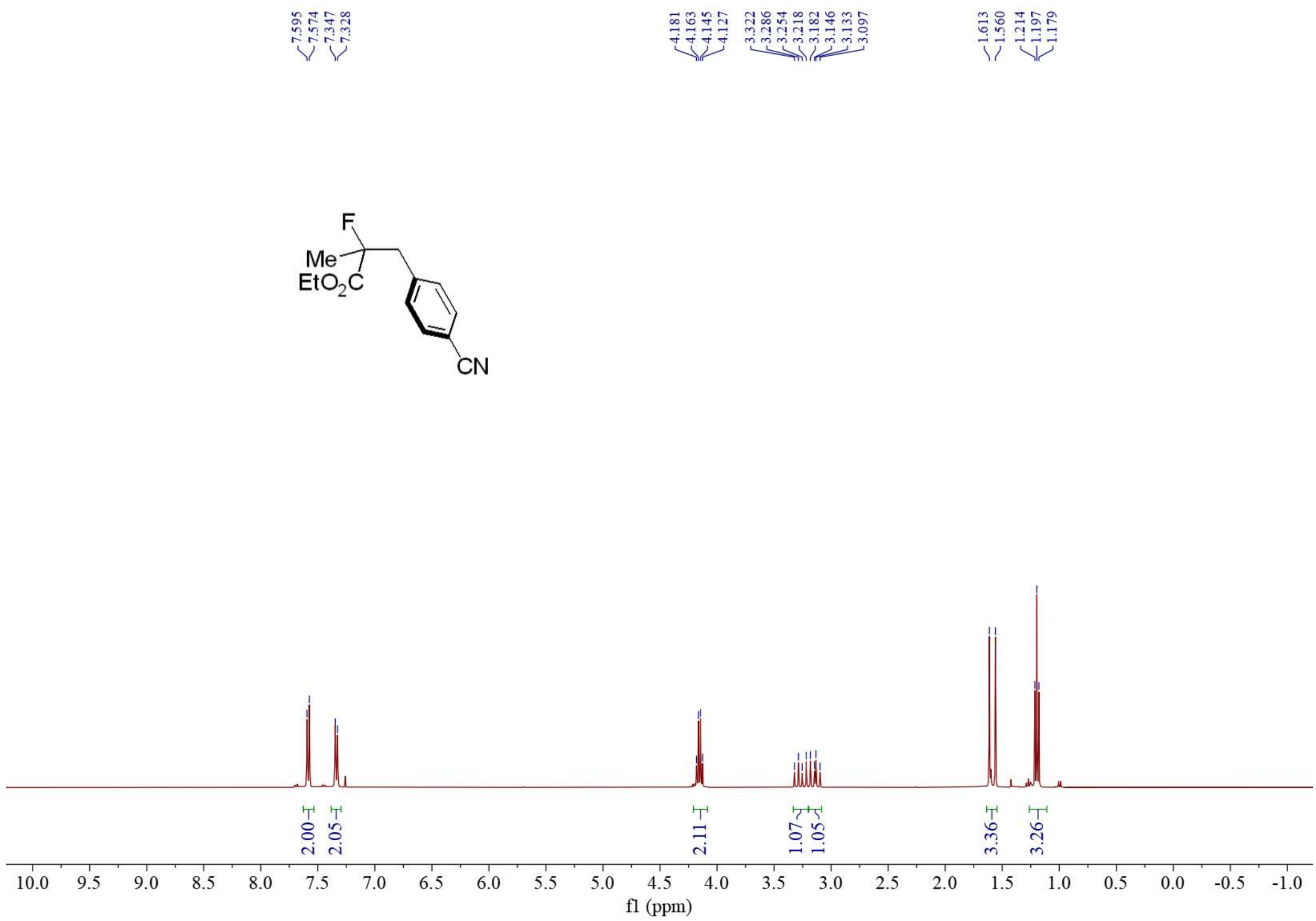

^{13}C NMR spectrum of **6g** (101 MHz, $CDCl_3$)

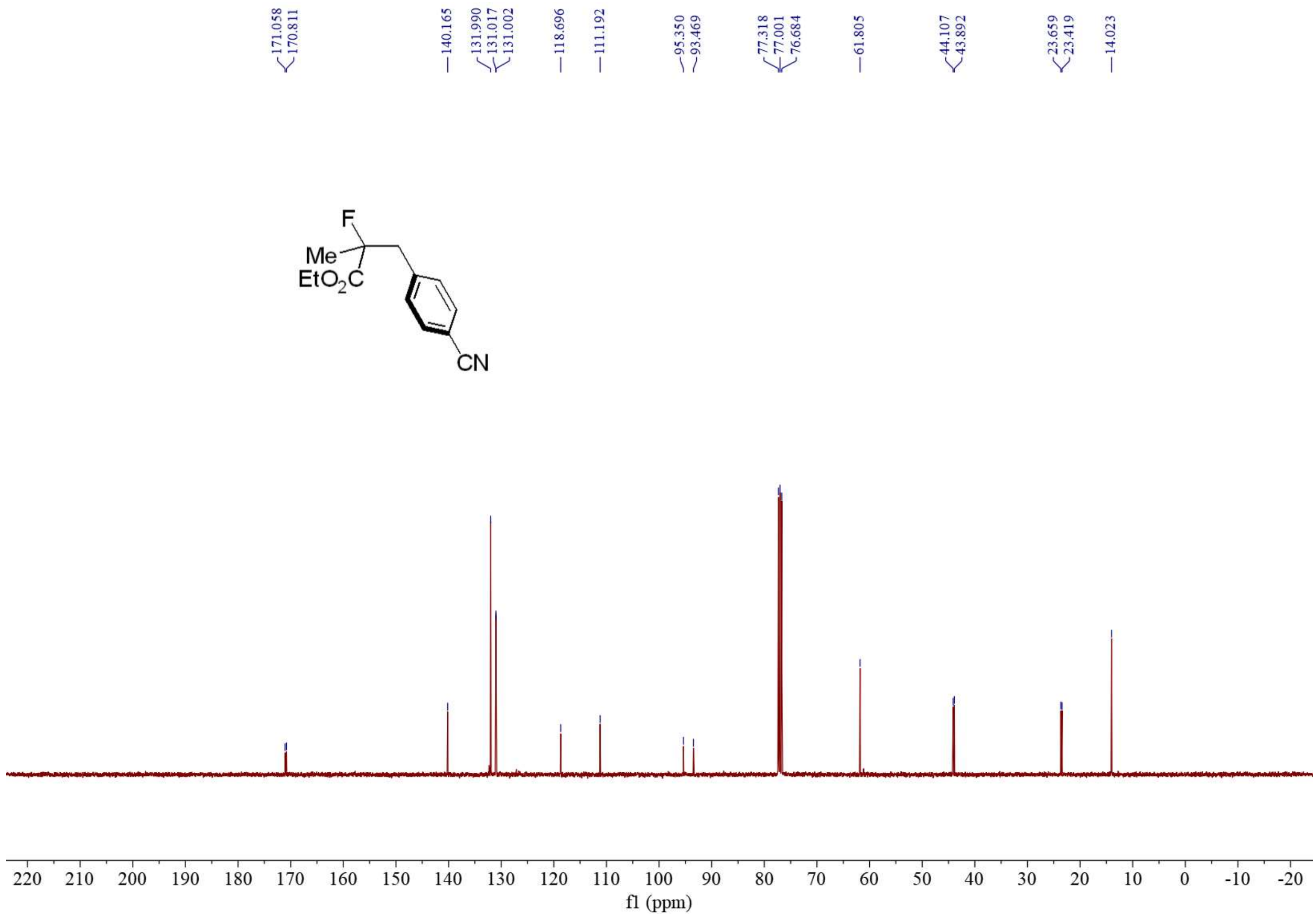

$^{19}F\{^1H\}$ NMR spectrum of **6g** (377 MHz, $CDCl_3$)

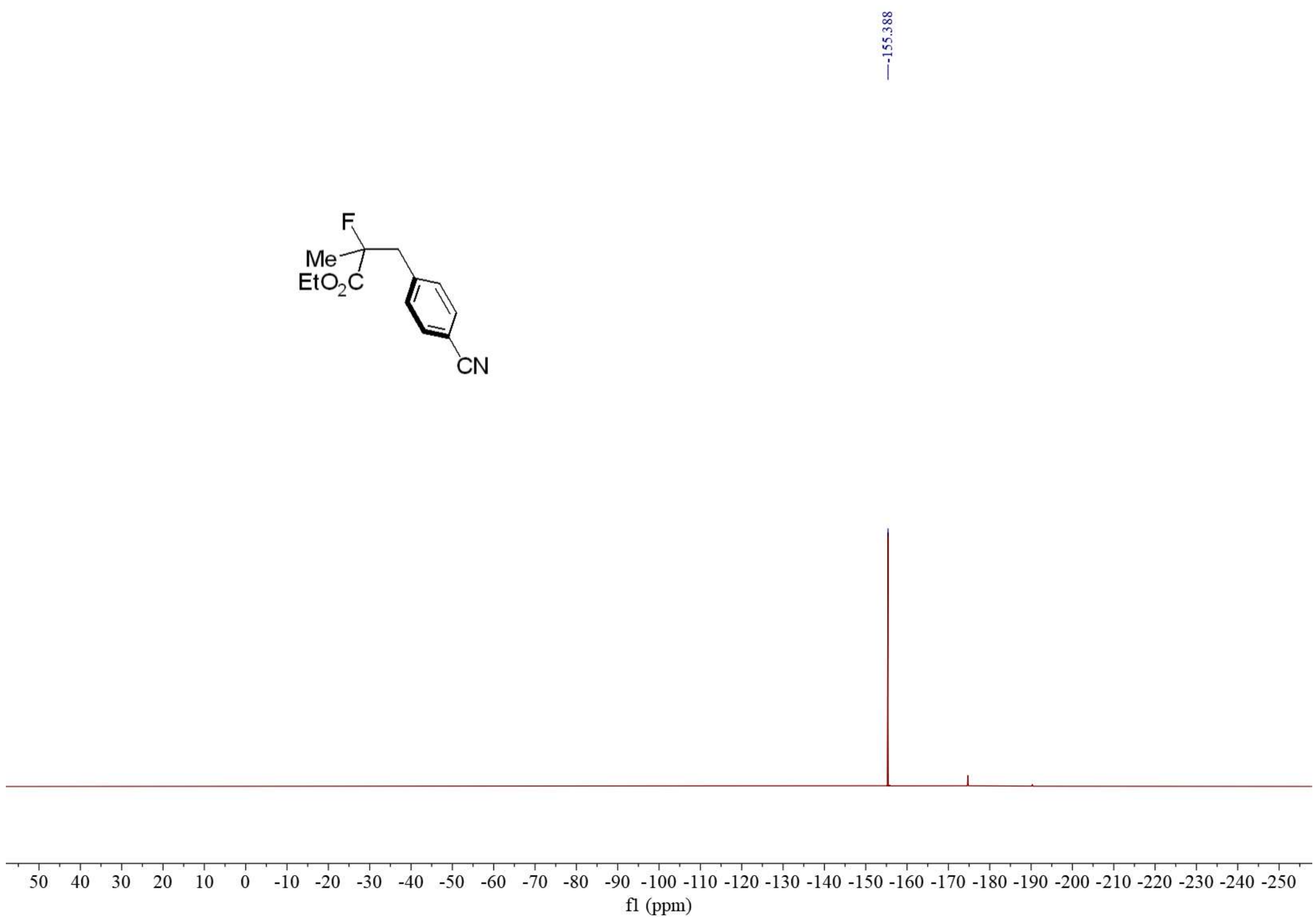

^{19}F NMR spectrum of **6g** (377 MHz, $CDCl_3$)

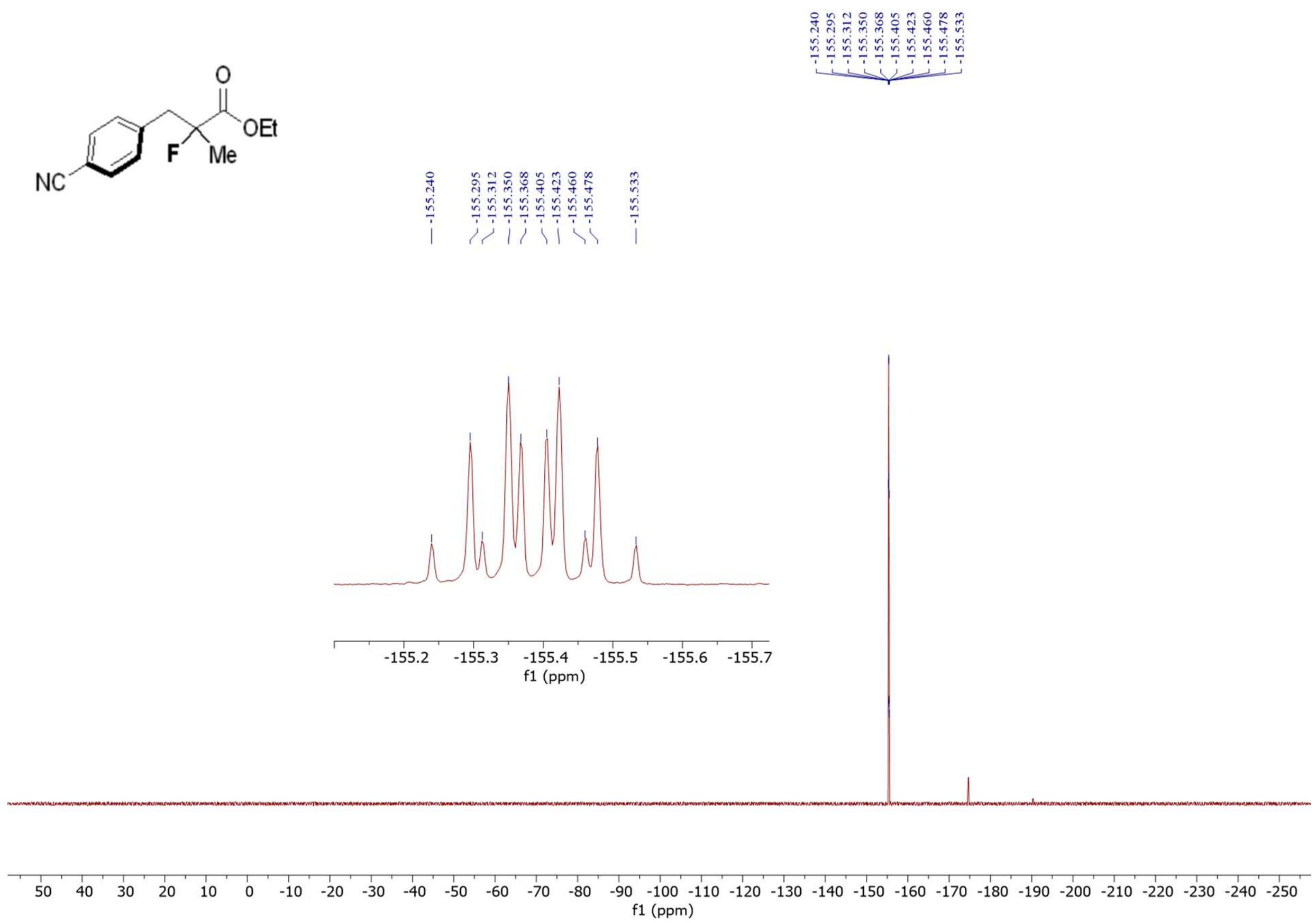

^{1}H NMR spectrum of **6h** (400 MHz, $CDCl_3$)

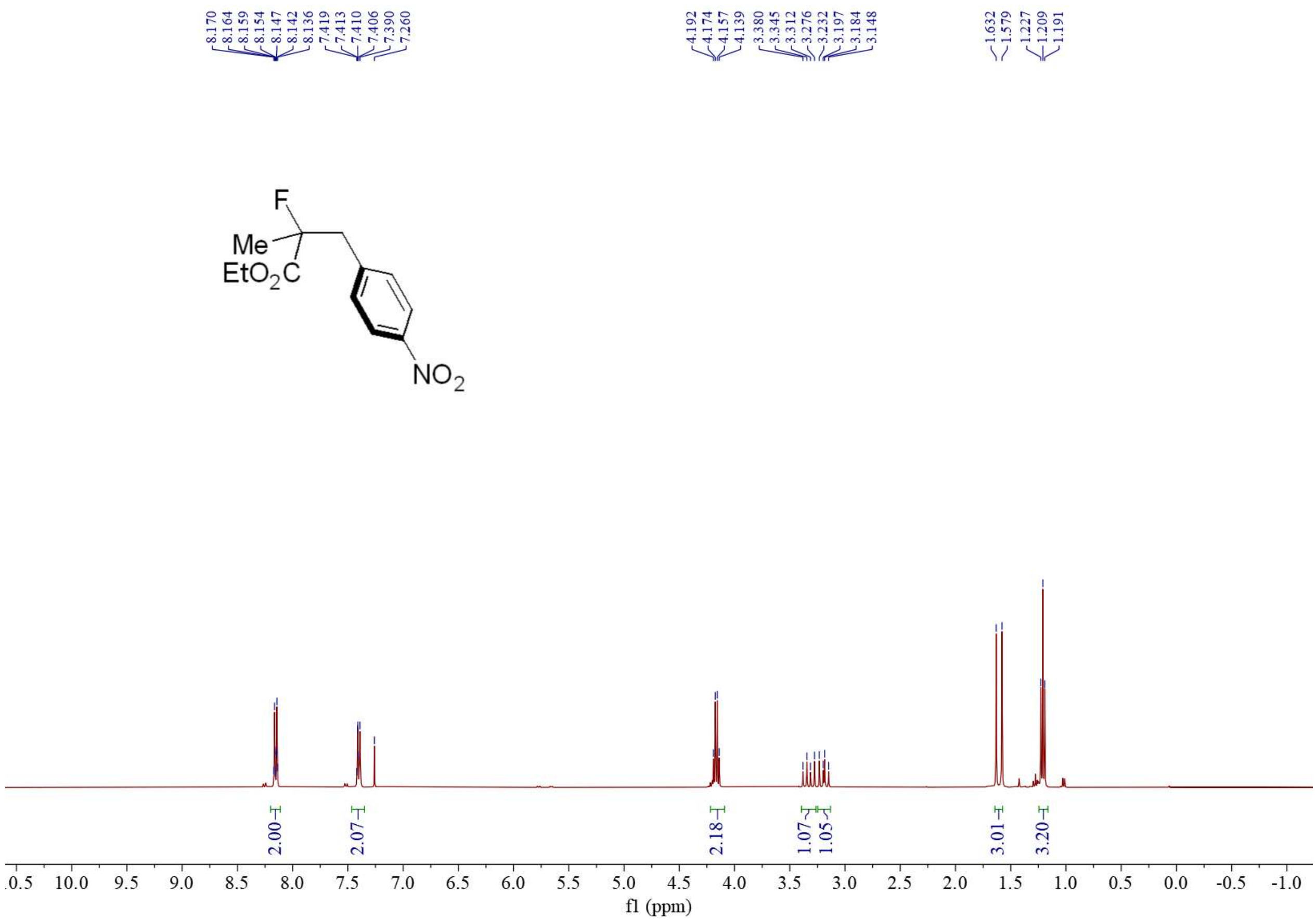

^{13}C NMR spectrum of **6h** (101 MHz, $CDCl_3$)

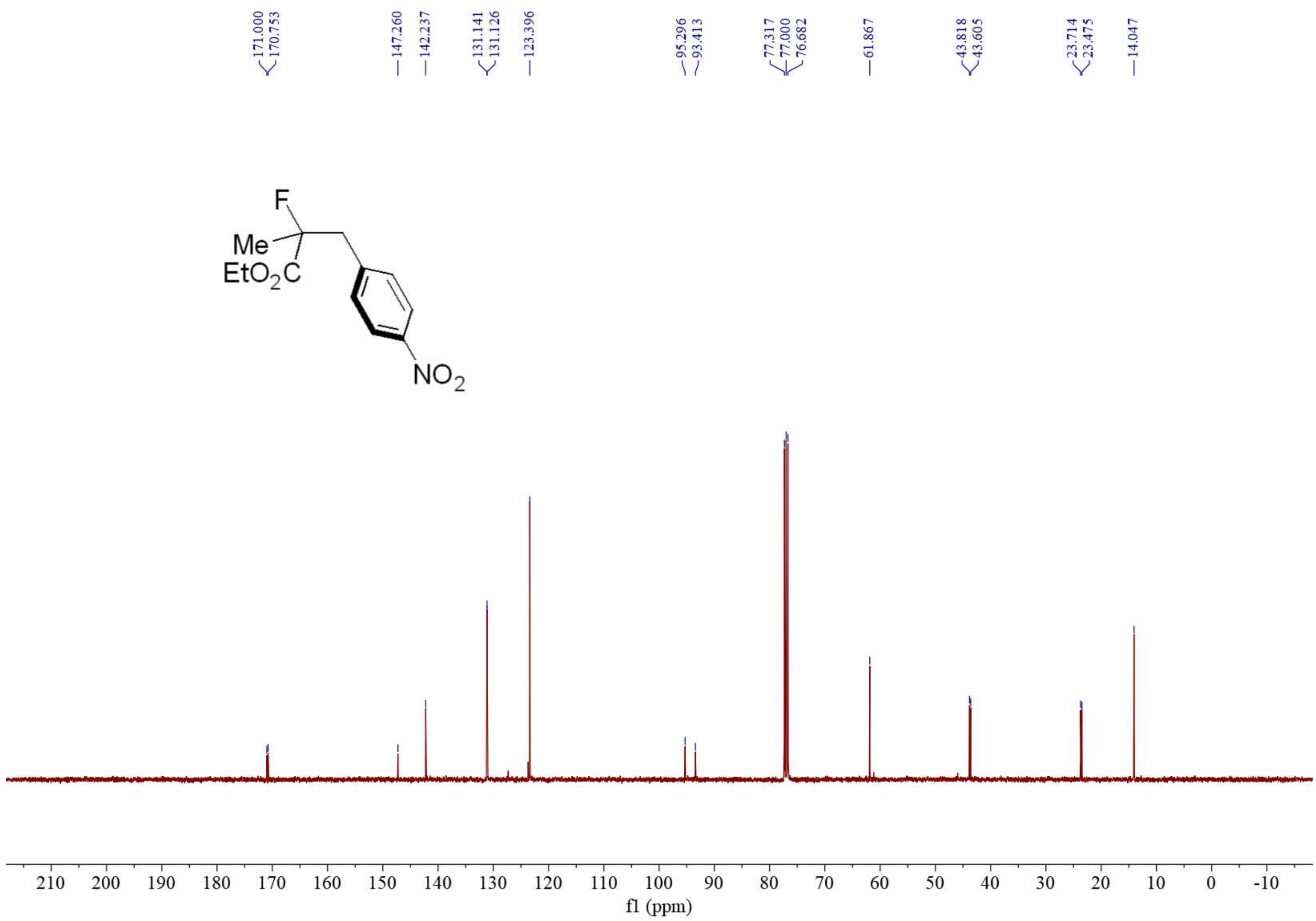

$^{19}F\{^1H\}$ NMR spectrum of **6h** (377 MHz, $CDCl_3$)

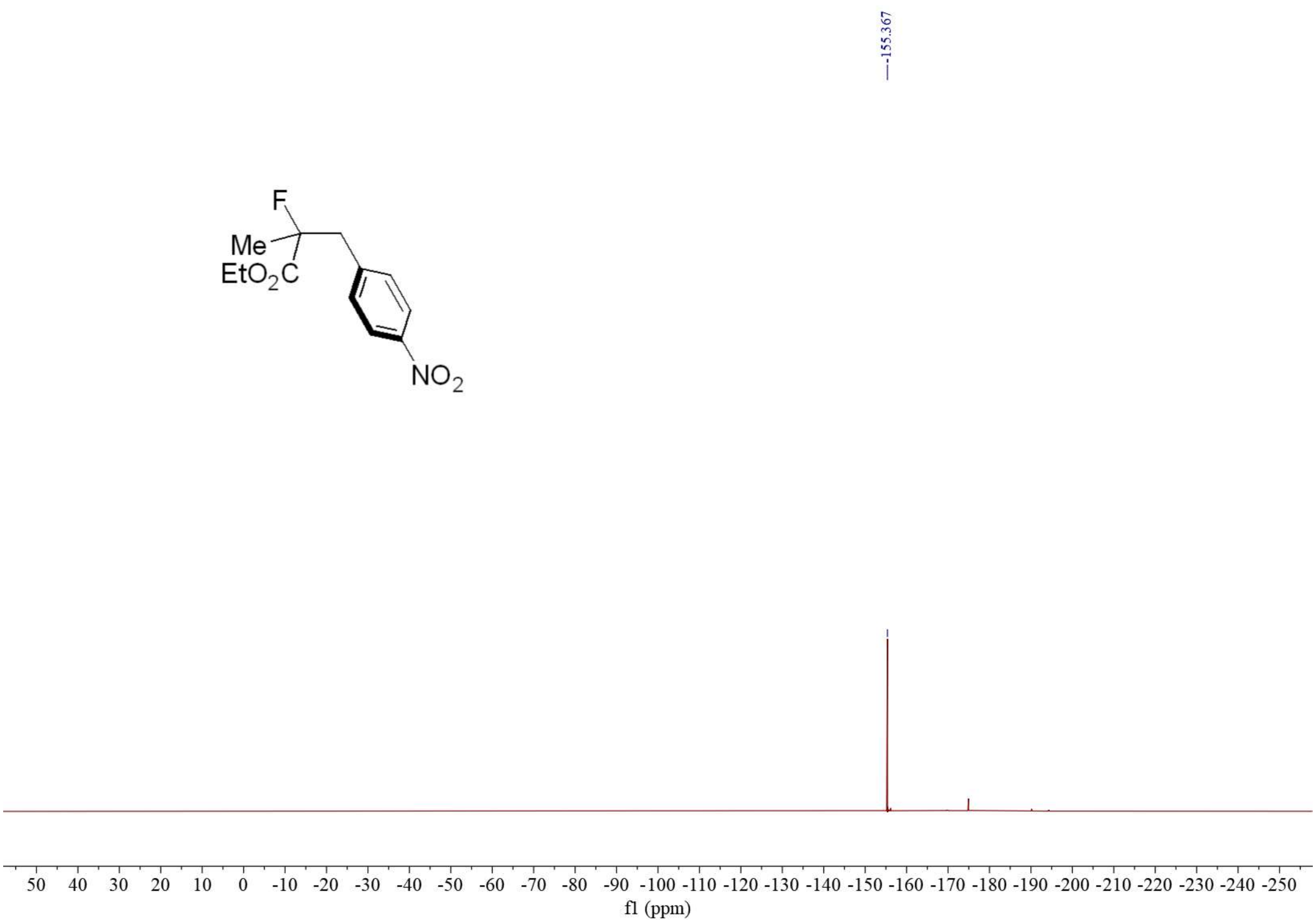

^{19}F NMR spectrum of **6h** (377 MHz, $CDCl_3$)

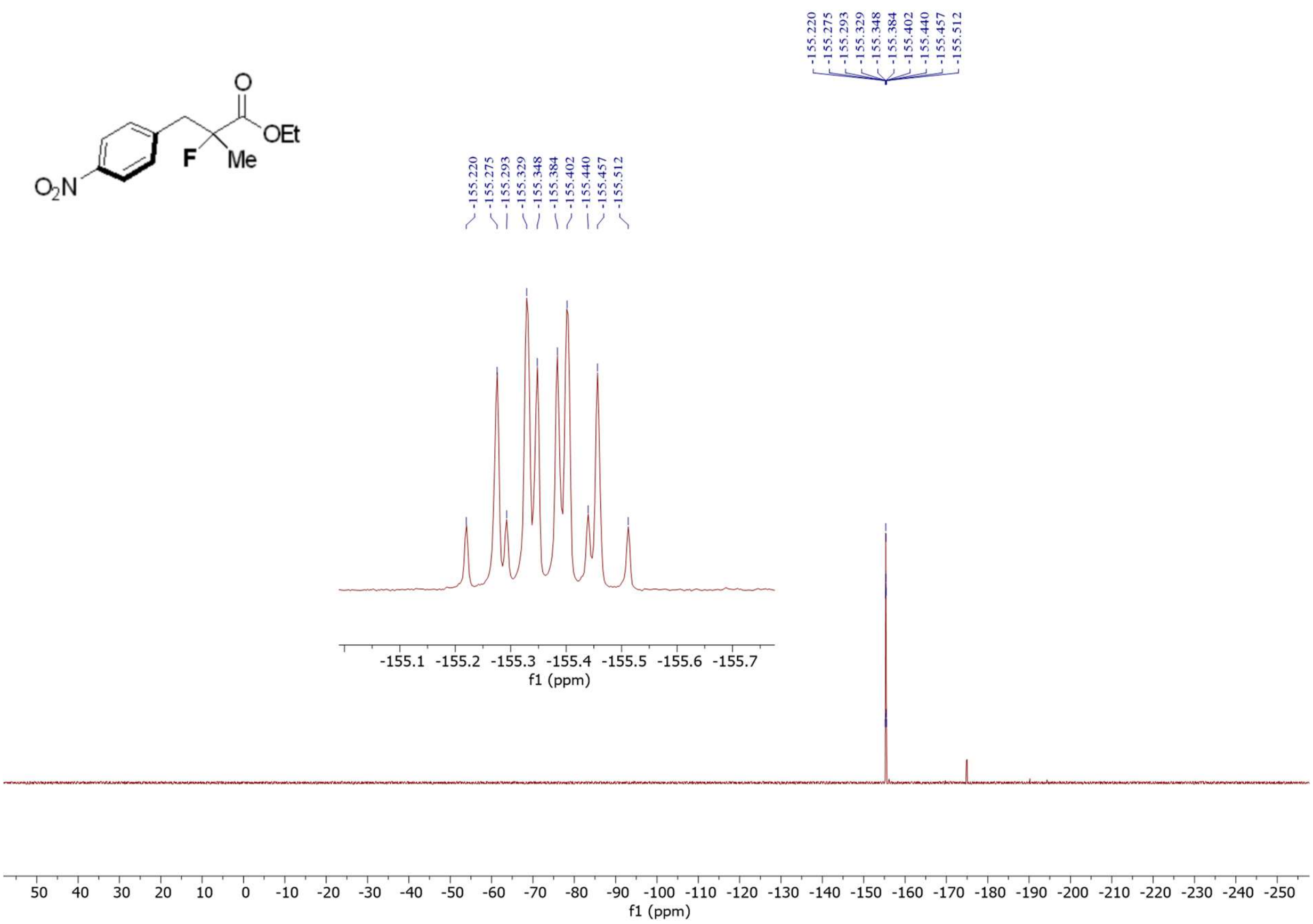

^{1}H NMR spectrum of **6i** (500 MHz, $CDCl_3$)

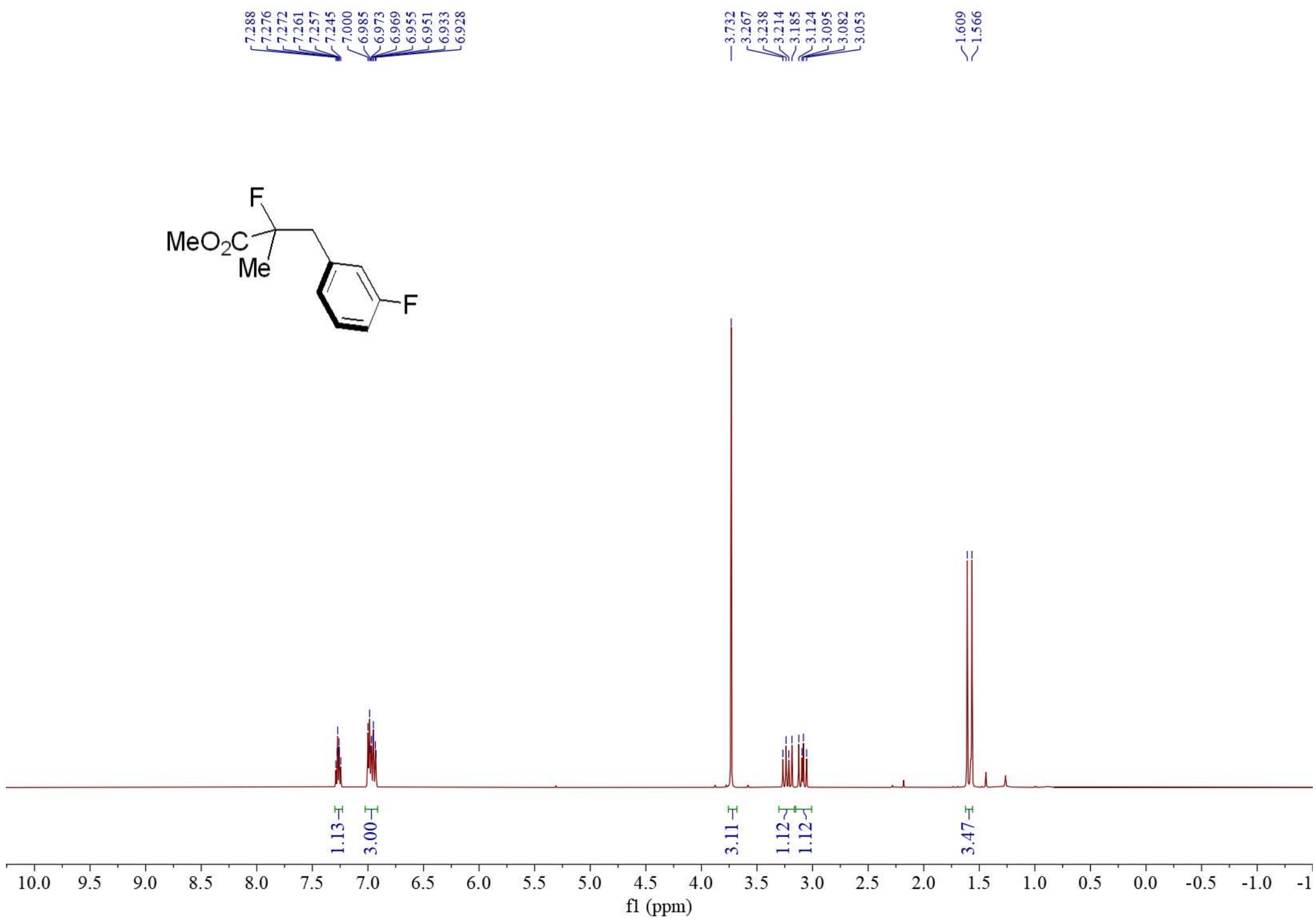

^{13}C NMR spectrum of **6i** (126 MHz, $CDCl_3$)

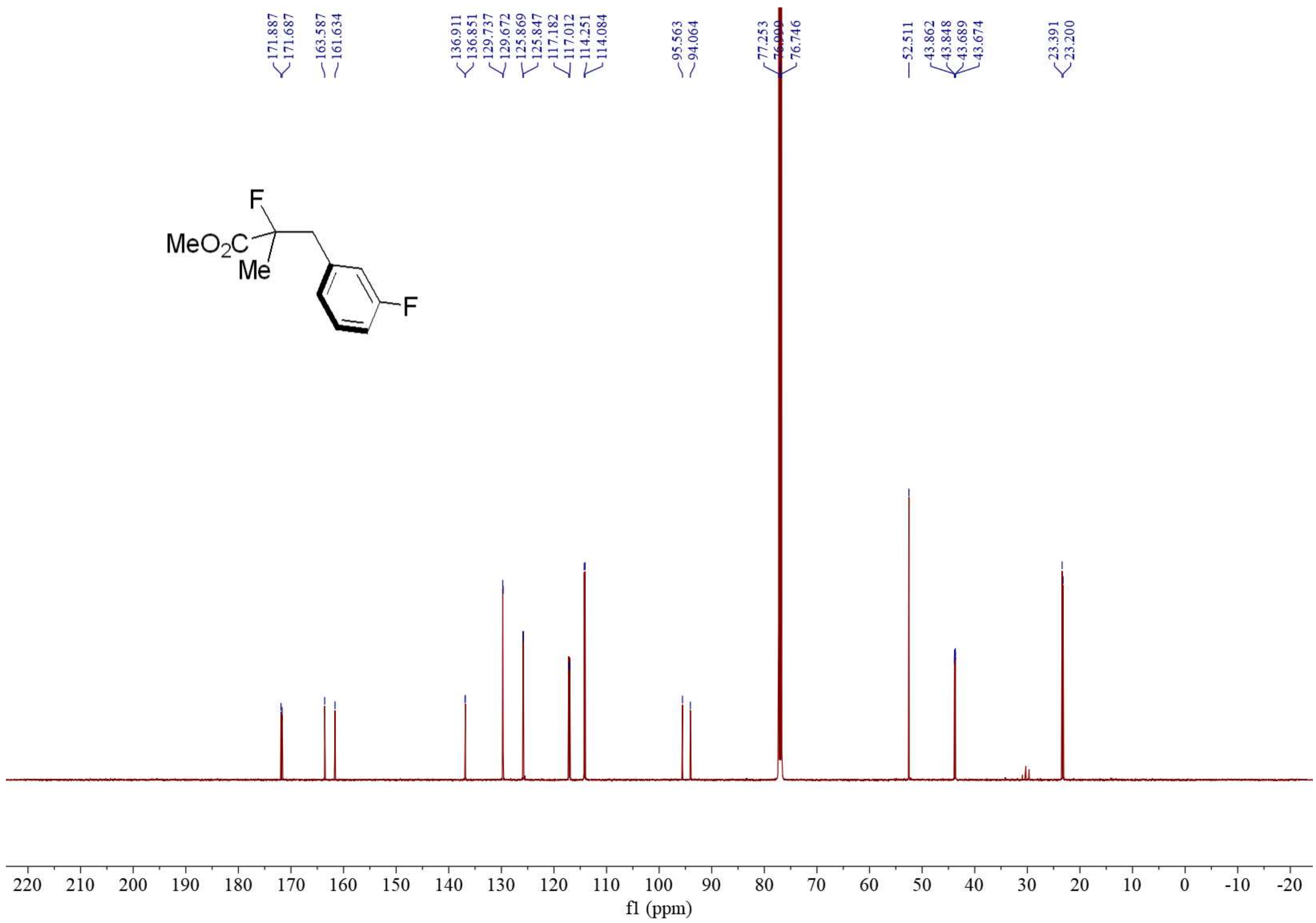

$^{19}F\{^{1}H\}$ NMR spectrum of **6i** (377 MHz, $CDCl_3$)

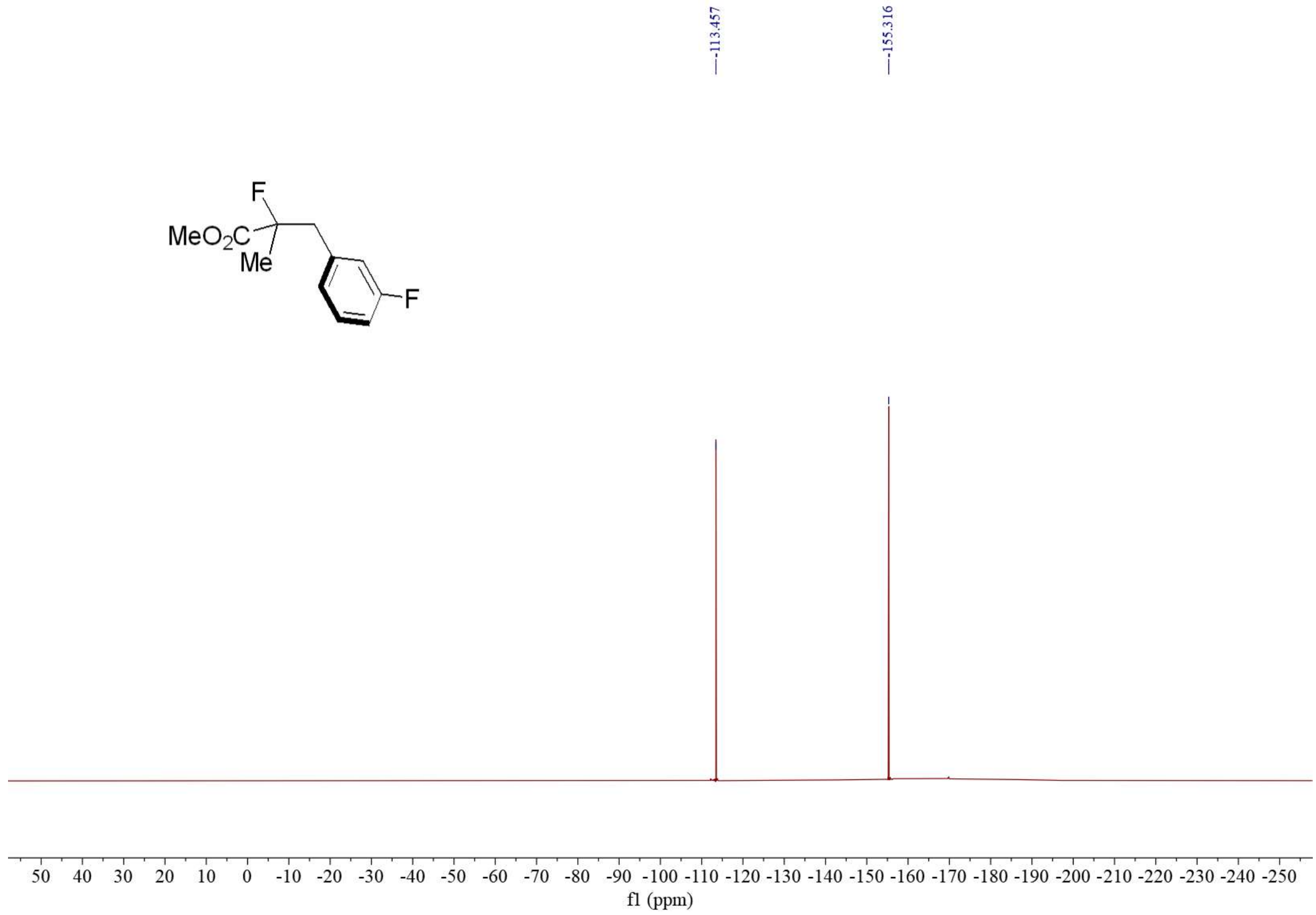

^{19}F NMR spectrum of **6i** (377 MHz, $CDCl_3$)

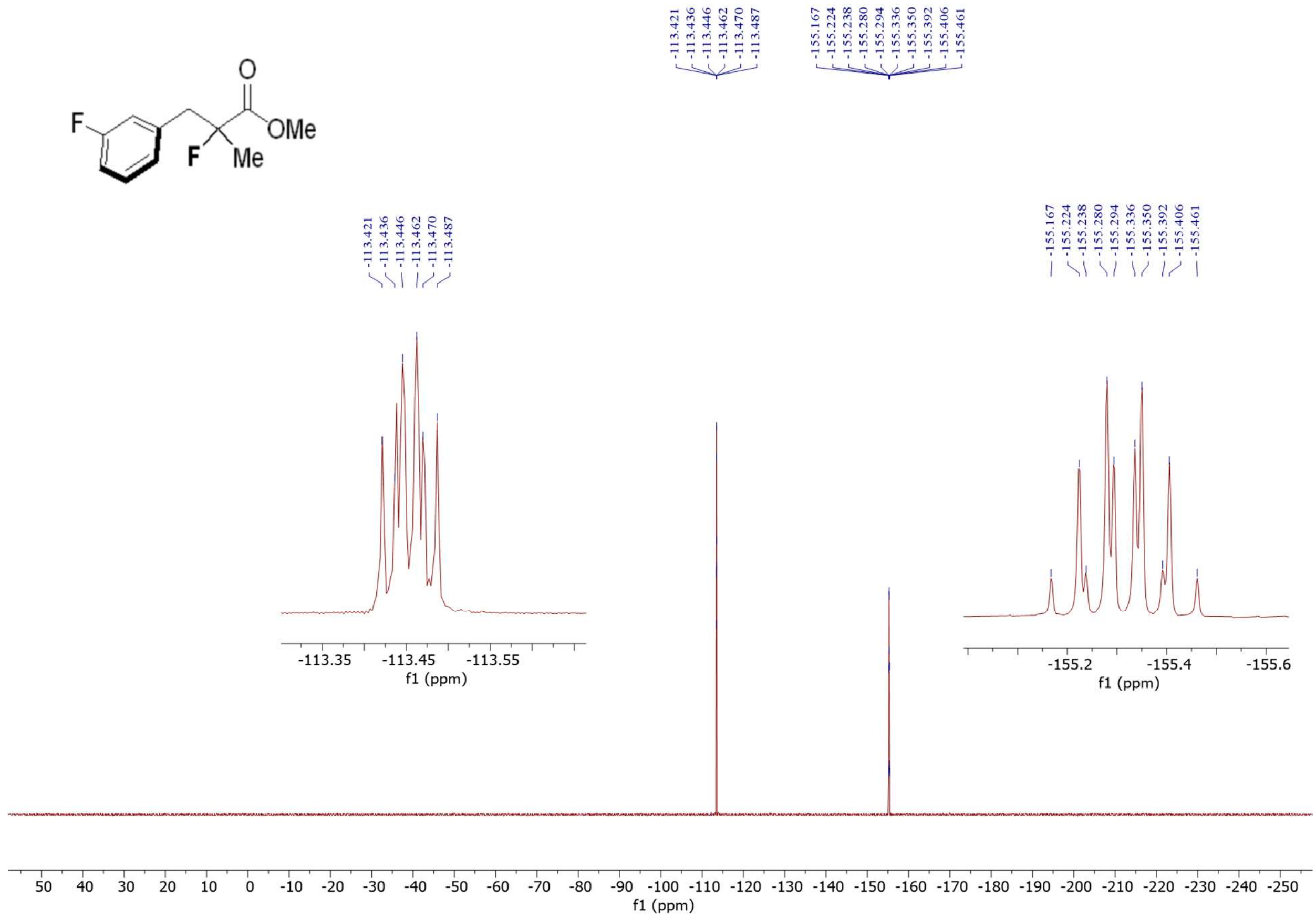

^{1}H NMR spectrum of **6j** (400 MHz, $CDCl_3$)

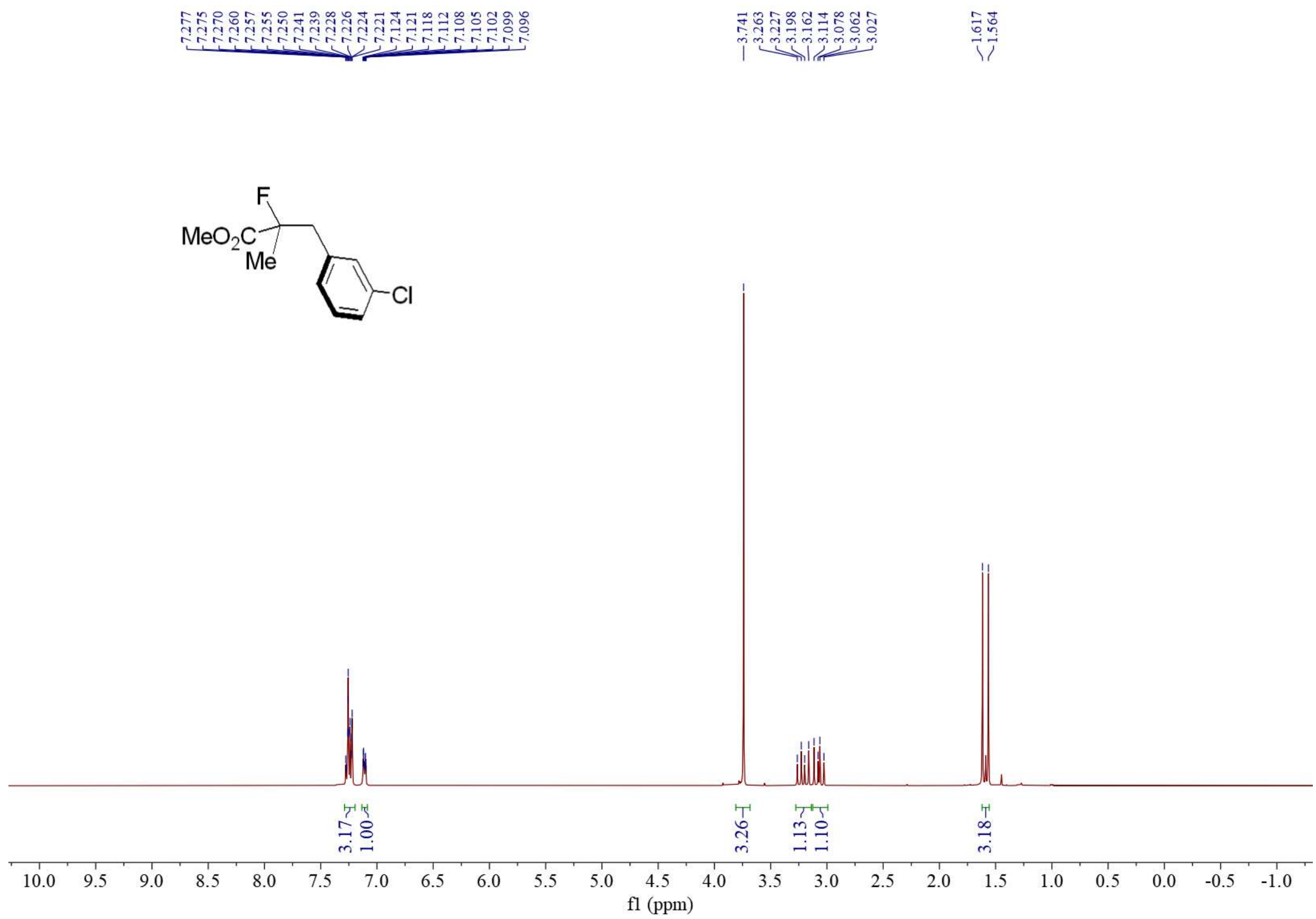

^{13}C NMR spectrum of **6j** (101 MHz, $CDCl_3$)

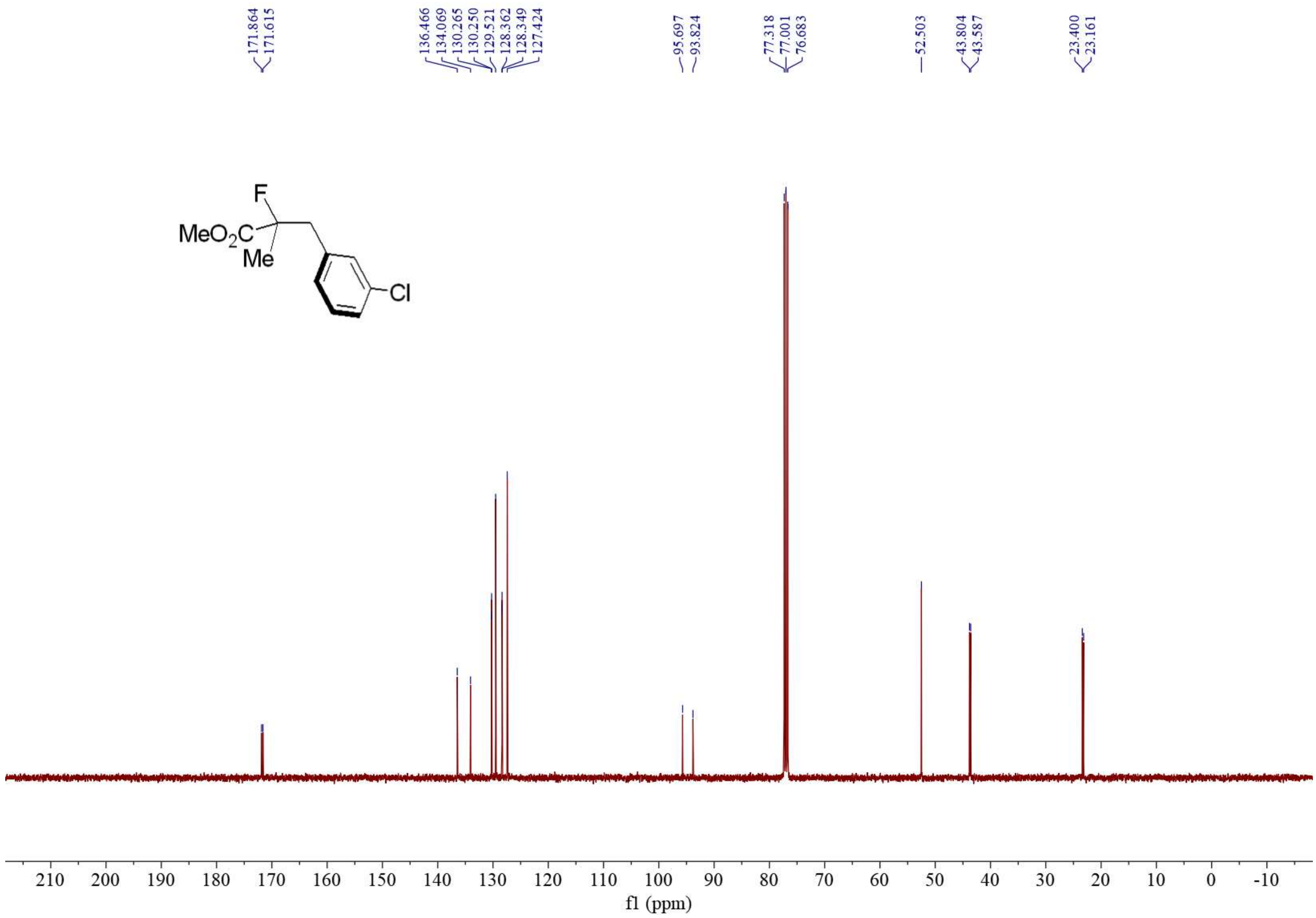

$^{19}F\{^{1}H\}$ NMR spectrum of **6j** (377 MHz, $CDCl_3$)

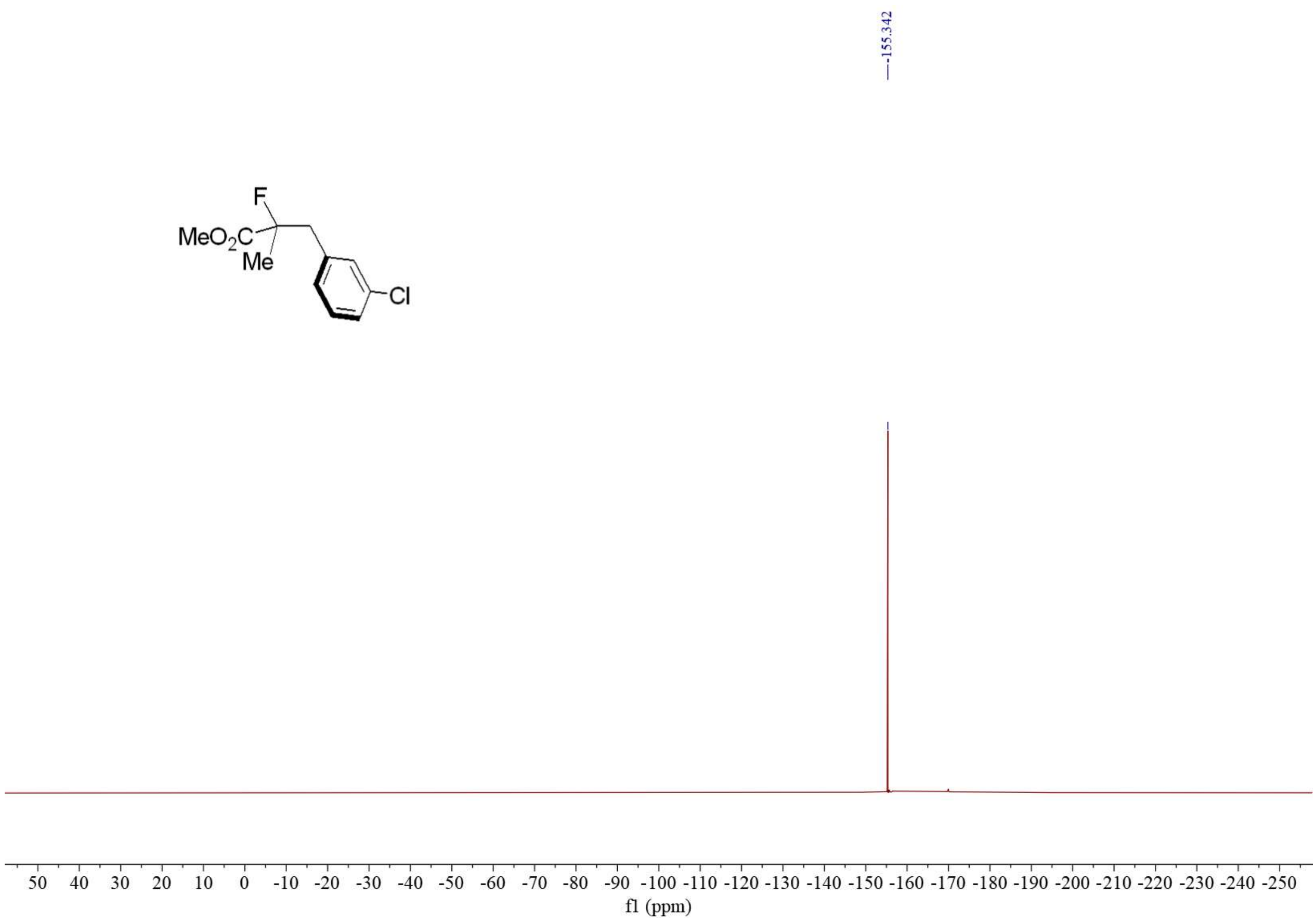

^{19}F NMR spectrum of **6j** (377 MHz, $CDCl_3$)

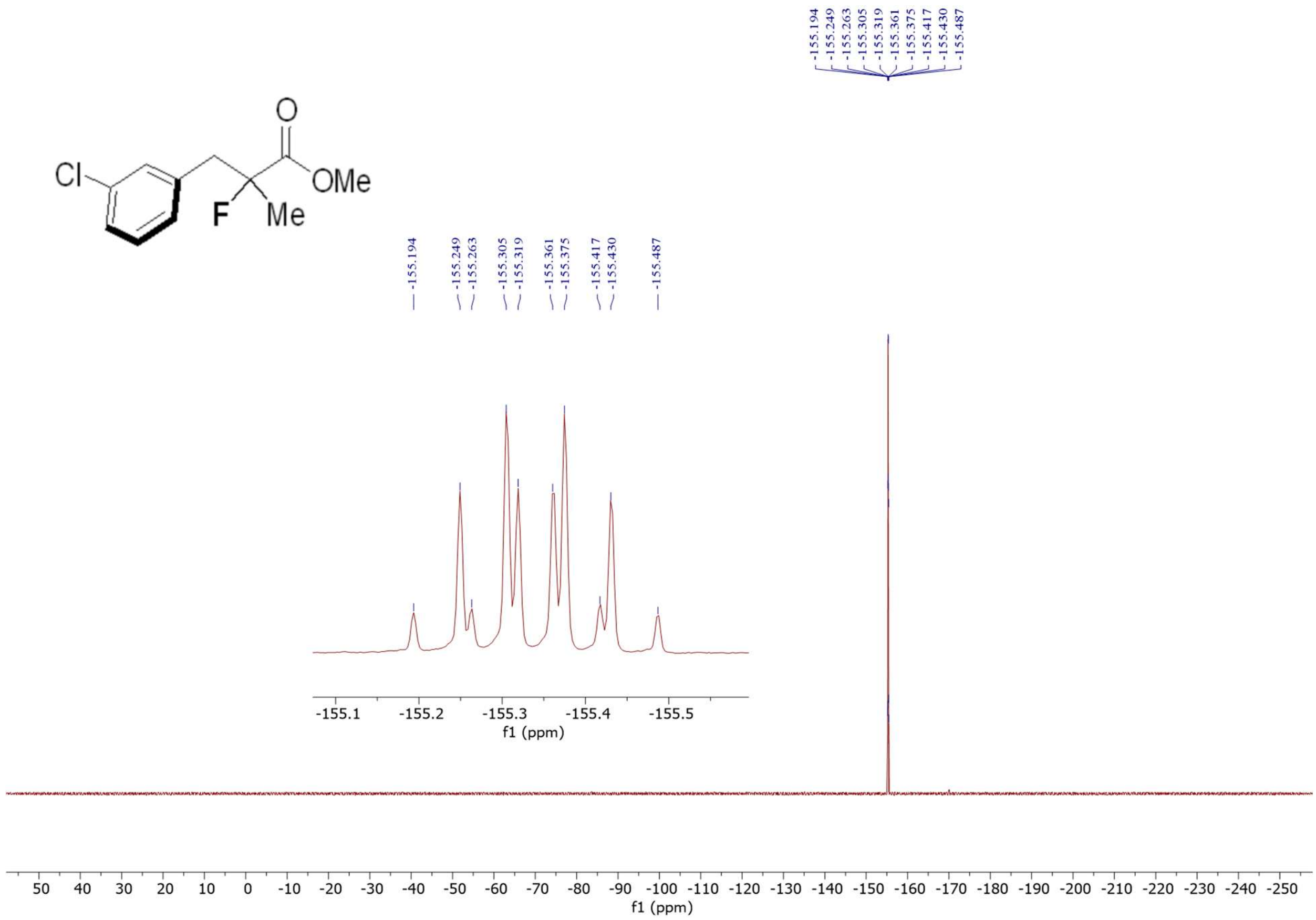

^{1}H NMR spectrum of **6k** (400 MHz, $CDCl_3$)

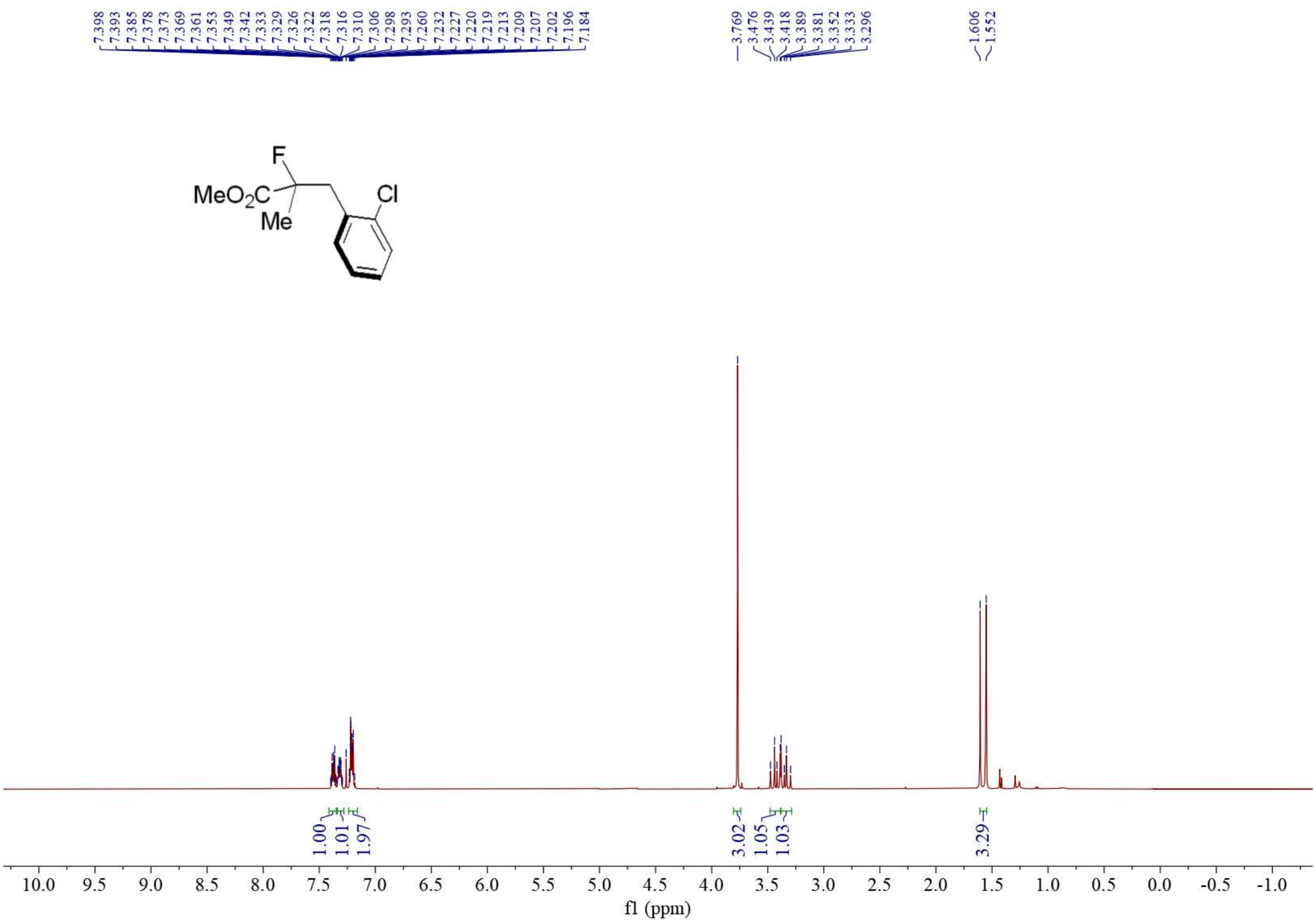

^{13}C NMR spectrum of **6k** (101 MHz, $CDCl_3$)

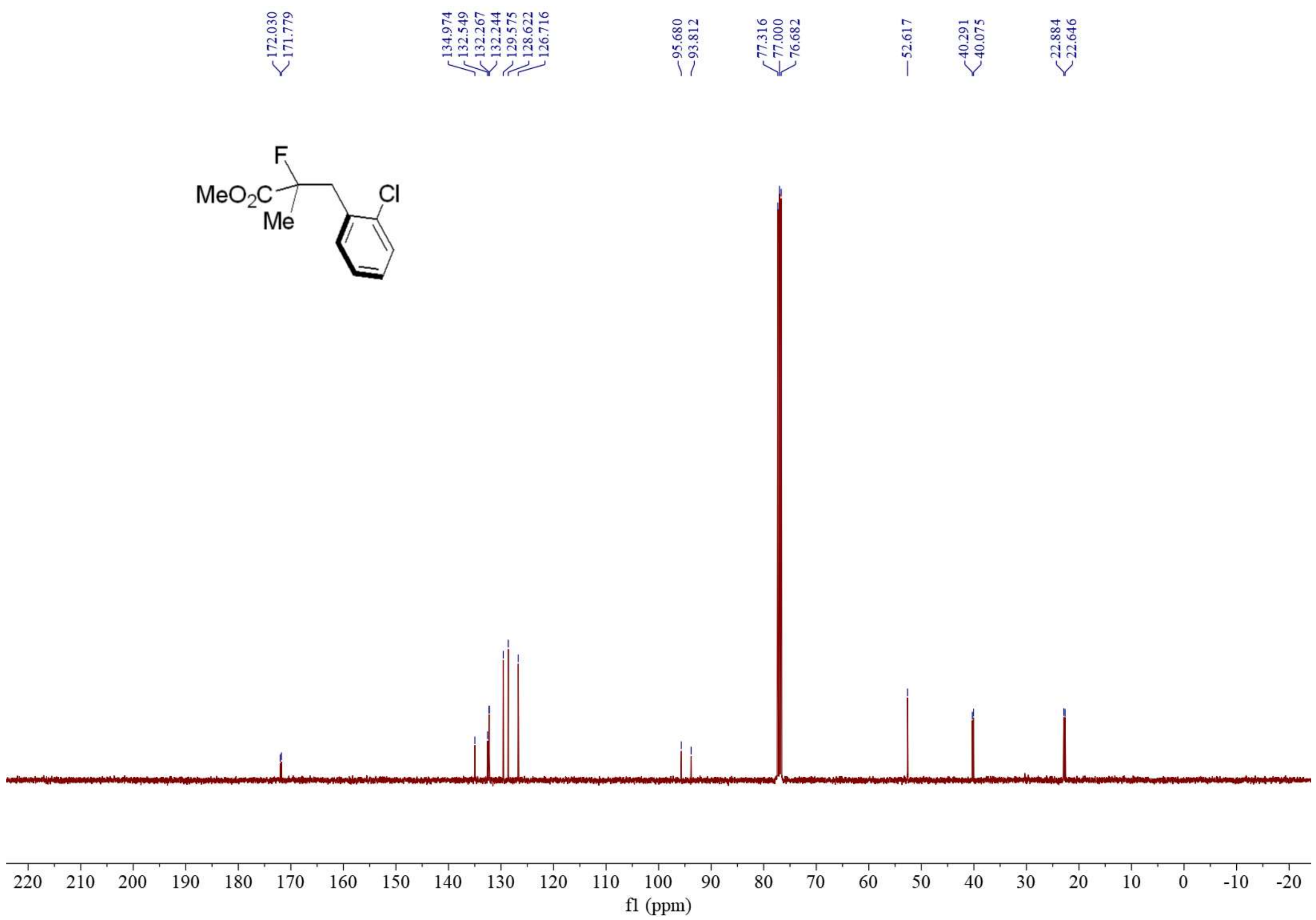

$^{19}F\{^1H\}$ NMR spectrum of **6k** (377 MHz, $CDCl_3$)

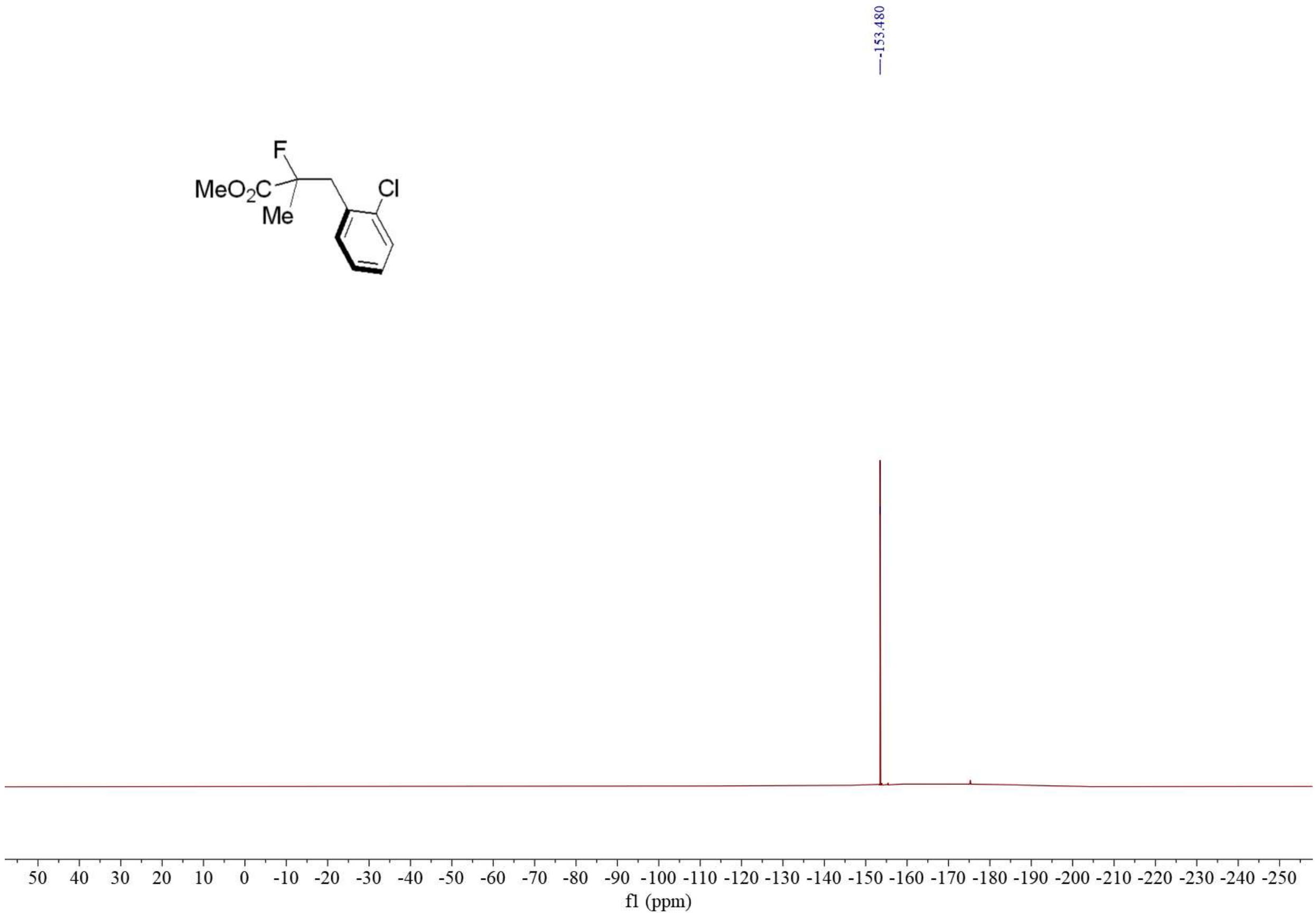

^{19}F NMR spectrum of **6k** (377 MHz, $CDCl_3$)

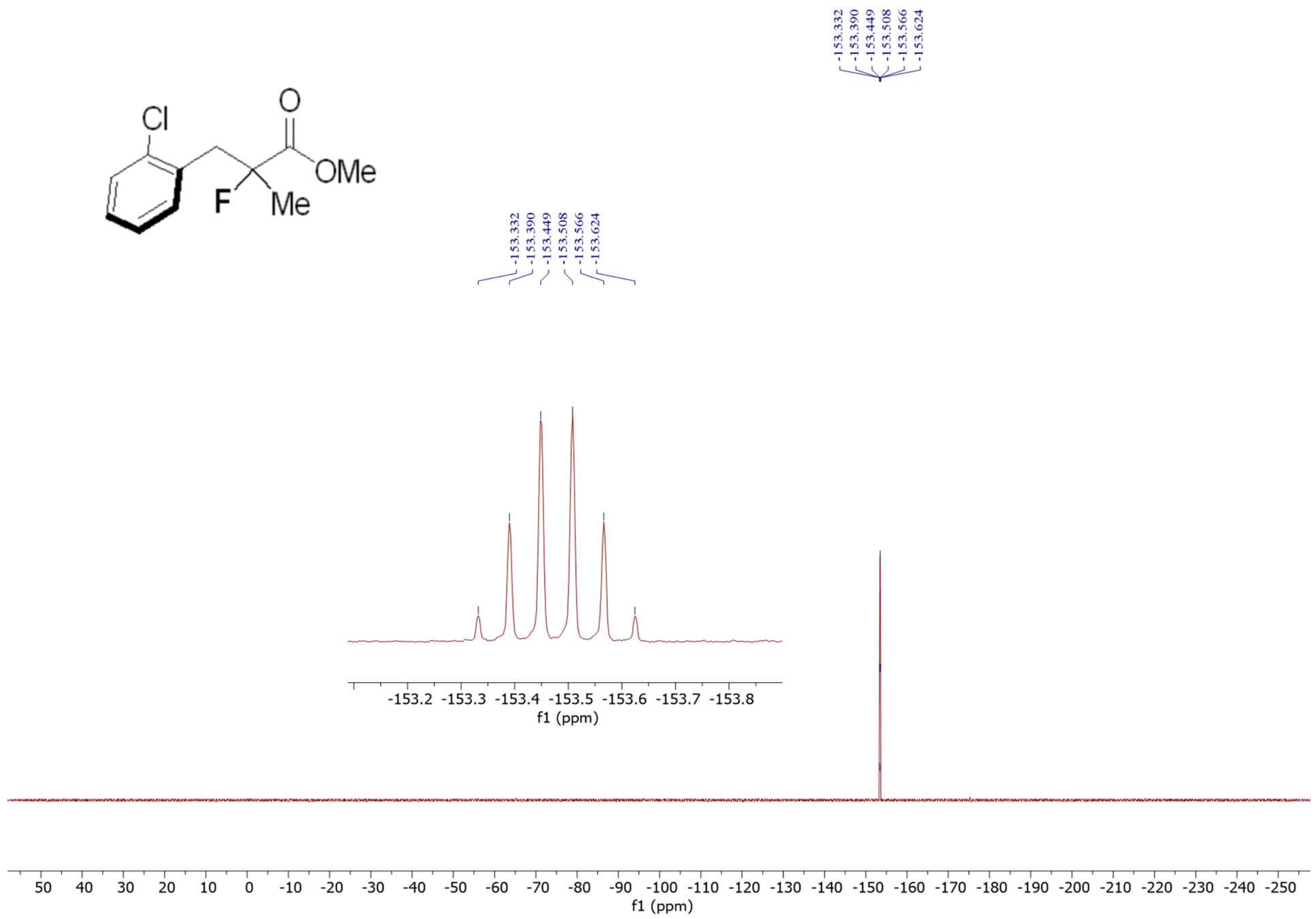

^{1}H NMR spectrum of **6l** (400 MHz, $CDCl_3$)

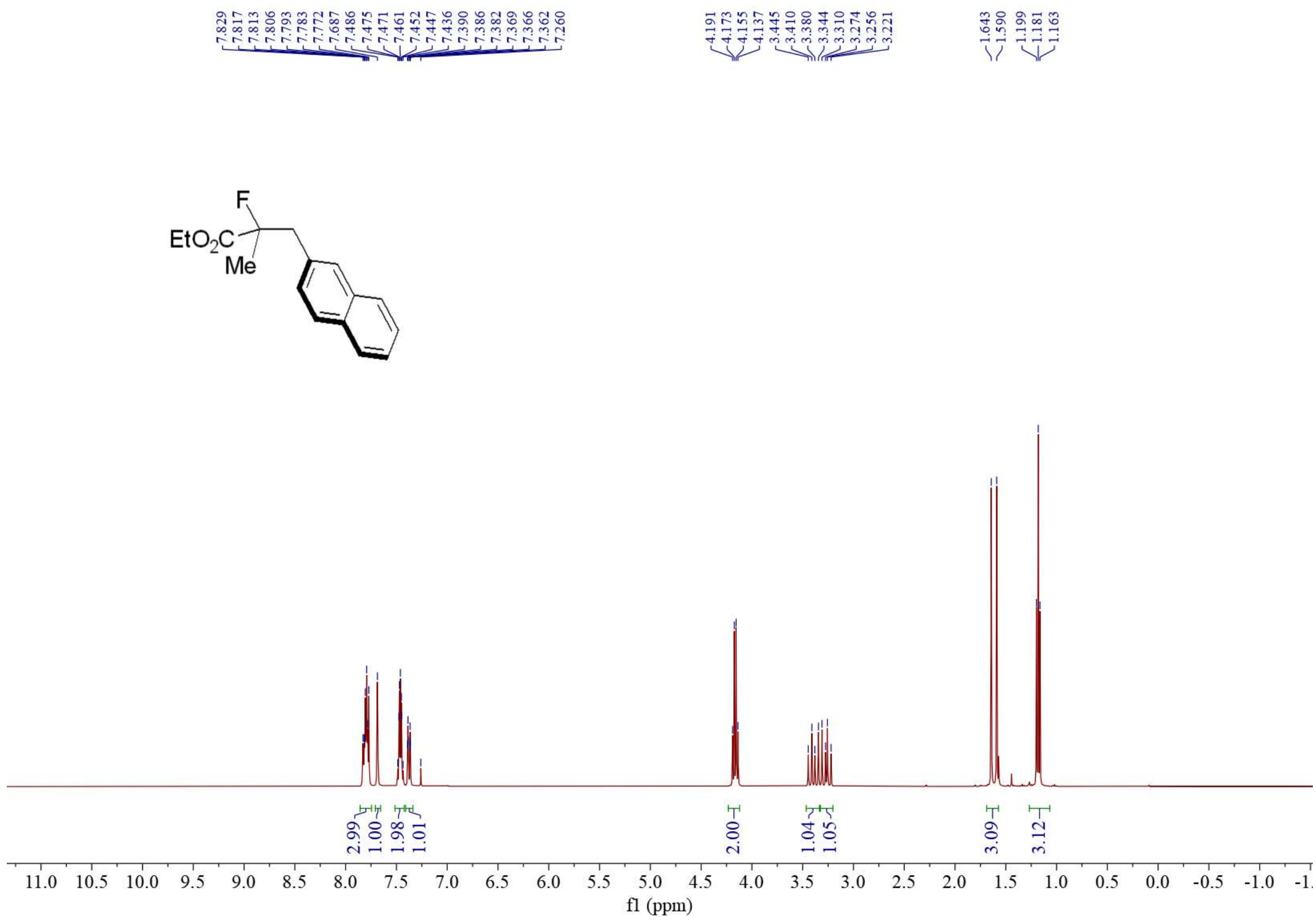

^{13}C NMR spectrum of **6l** (101 MHz, $CDCl_3$)

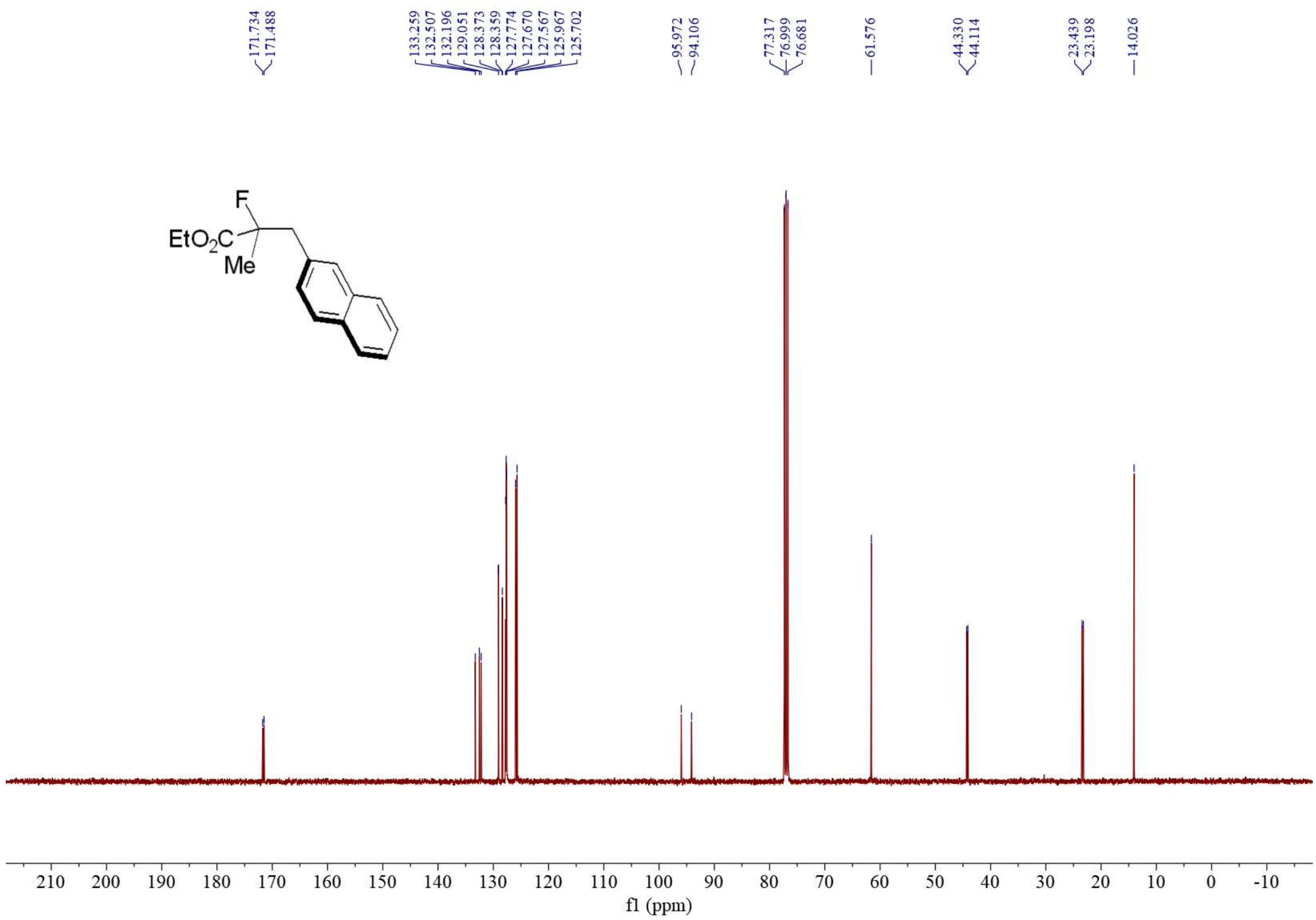

$^{19}F\{^{1}H\}$ NMR spectrum of **6l** (377 MHz, $CDCl_3$)

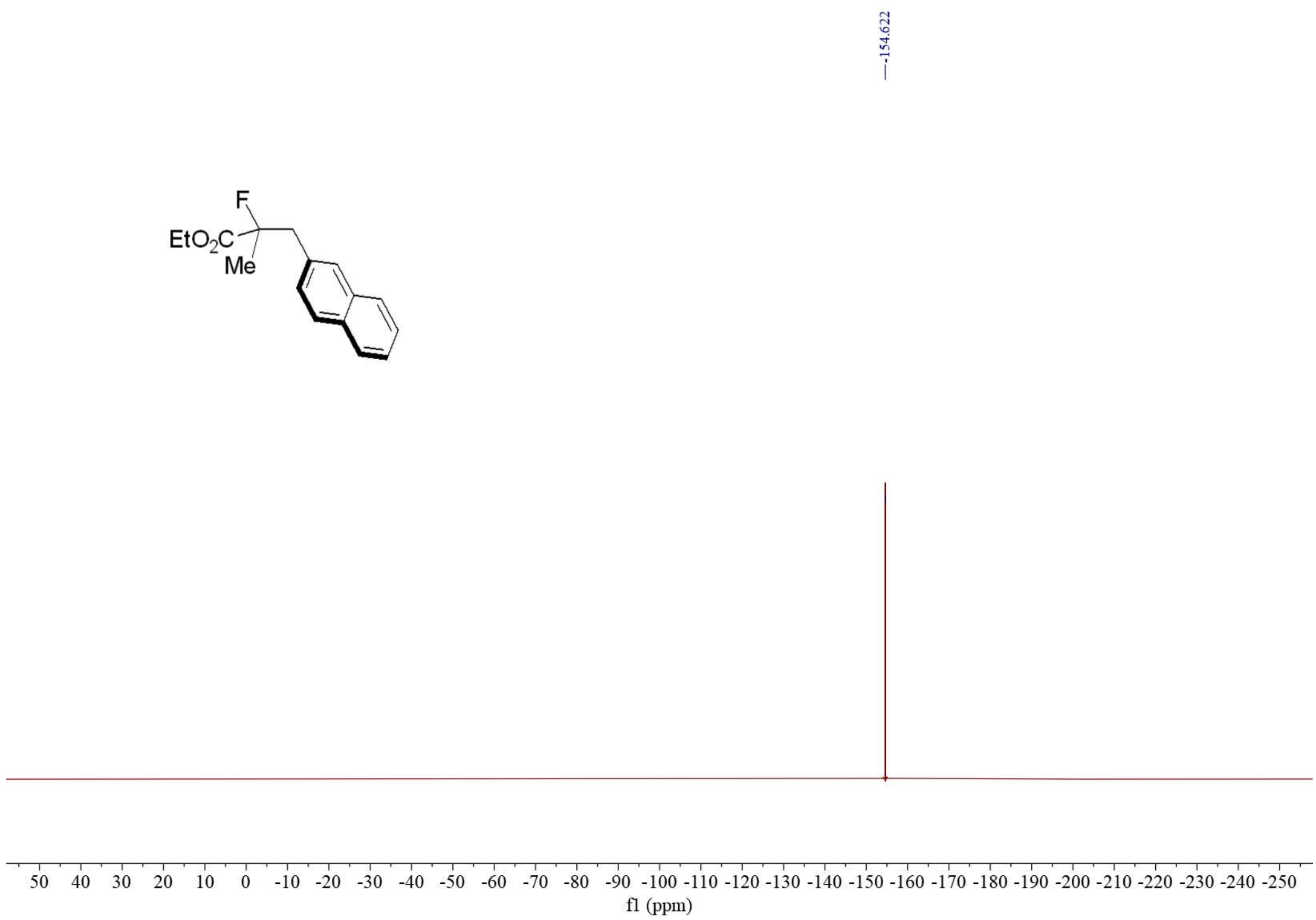

^{19}F NMR spectrum of **6l** (377 MHz, $CDCl_3$)

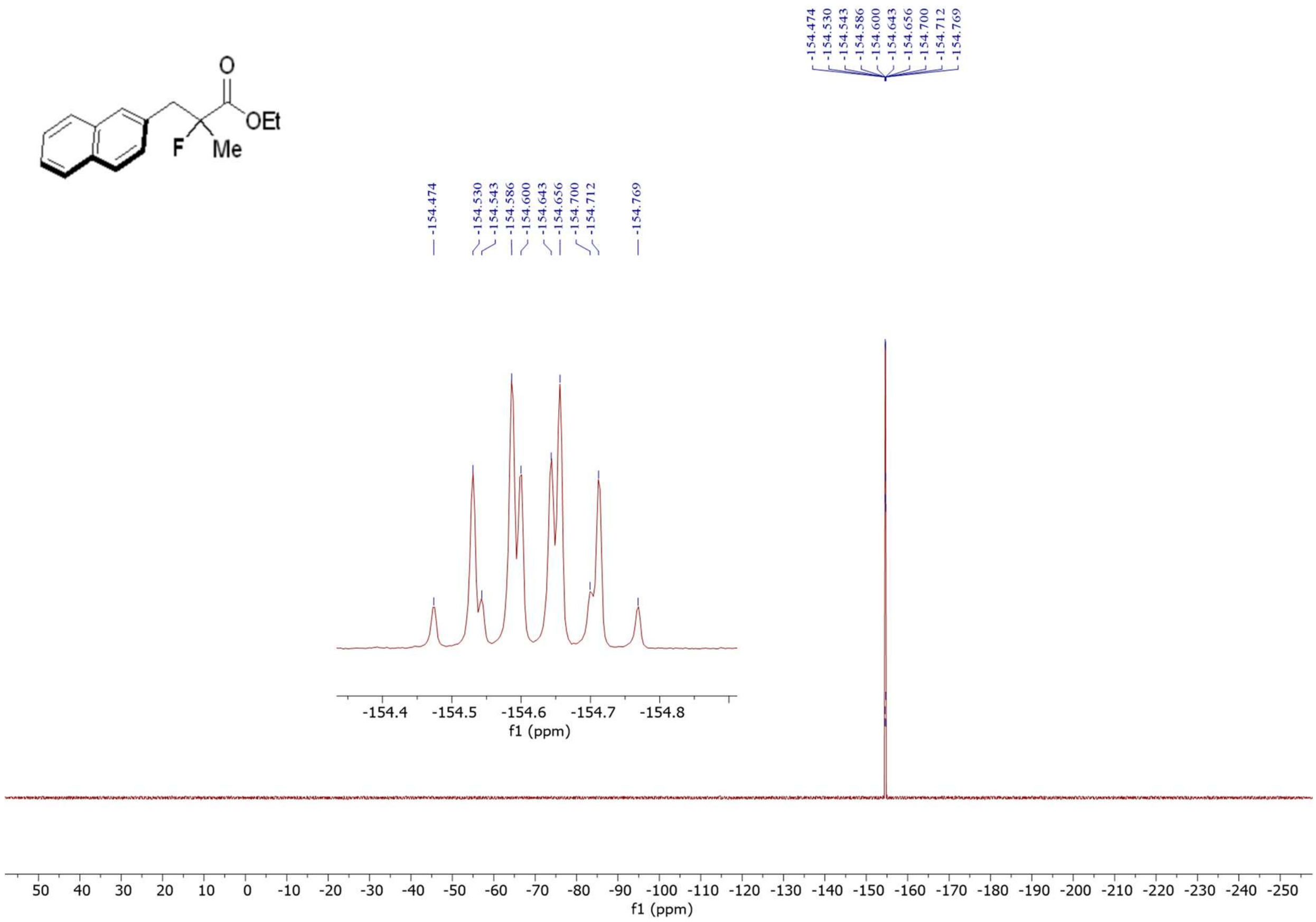

^{1}H NMR spectrum of **6m** (400 MHz, $CDCl_3$)

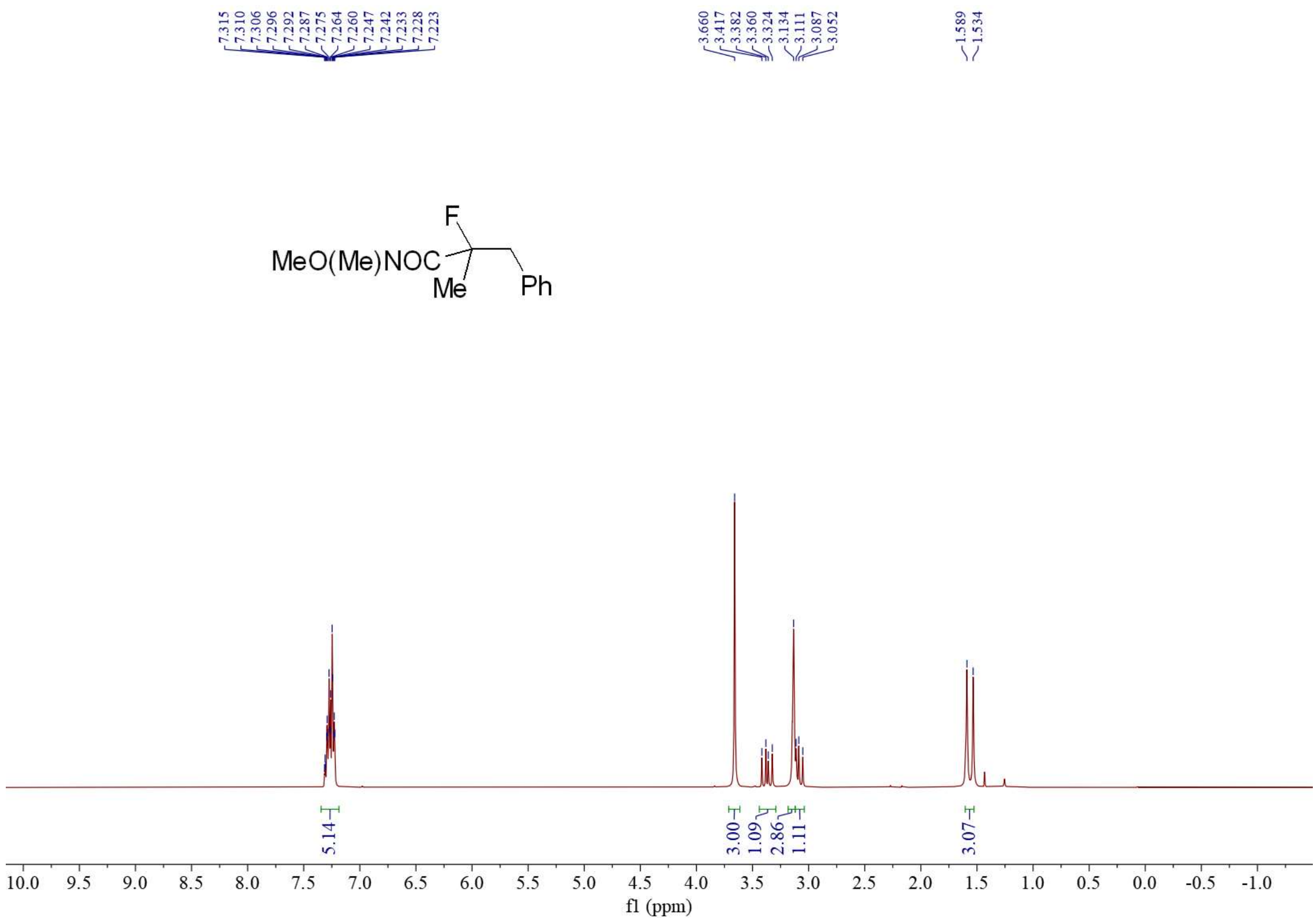

^{19}F{^{1}H} NMR spectrum of **6m** (377 MHz, $CDCl_3$)

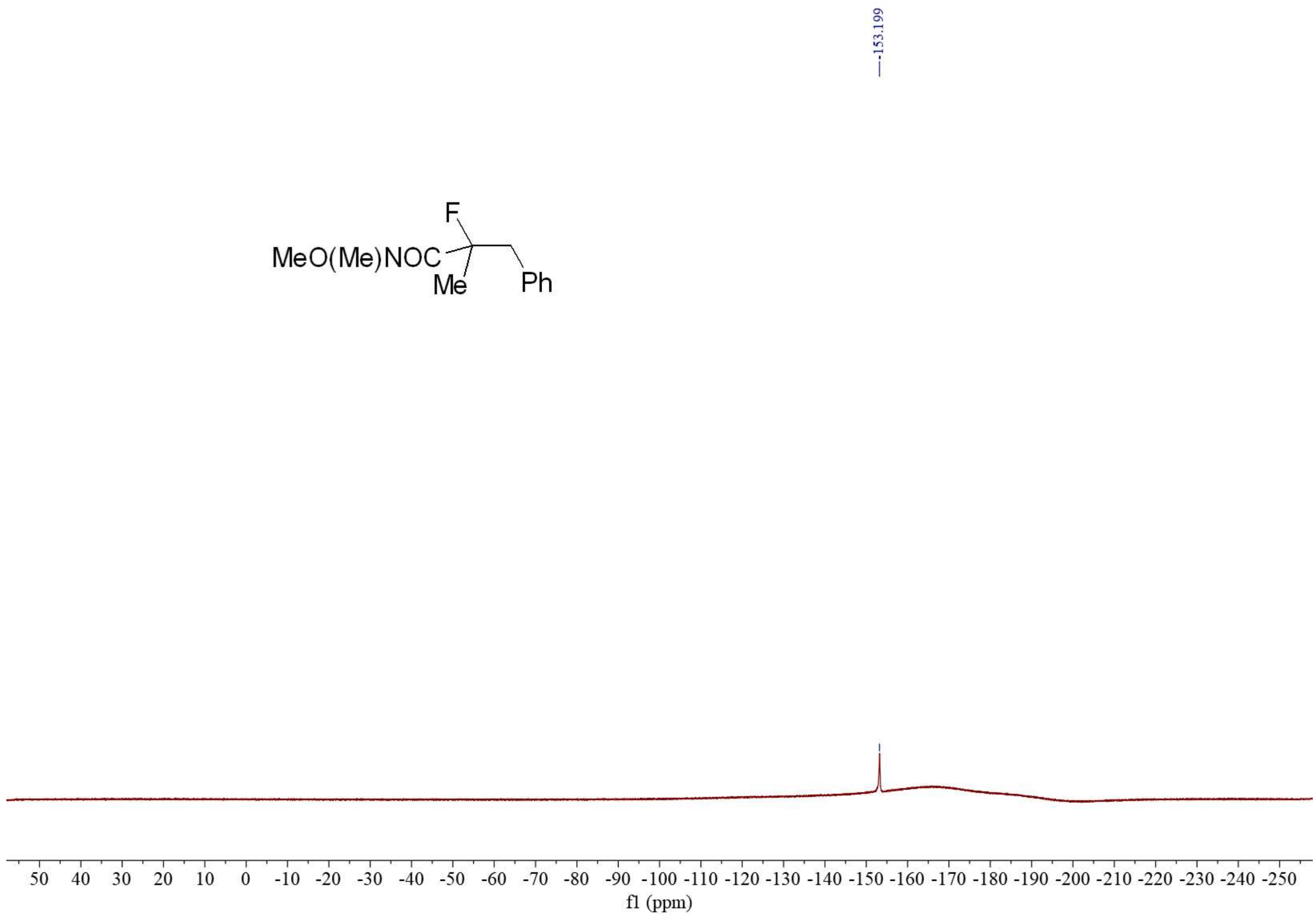

^{19}F NMR spectrum of **6m** (377 MHz, $CDCl_3$)

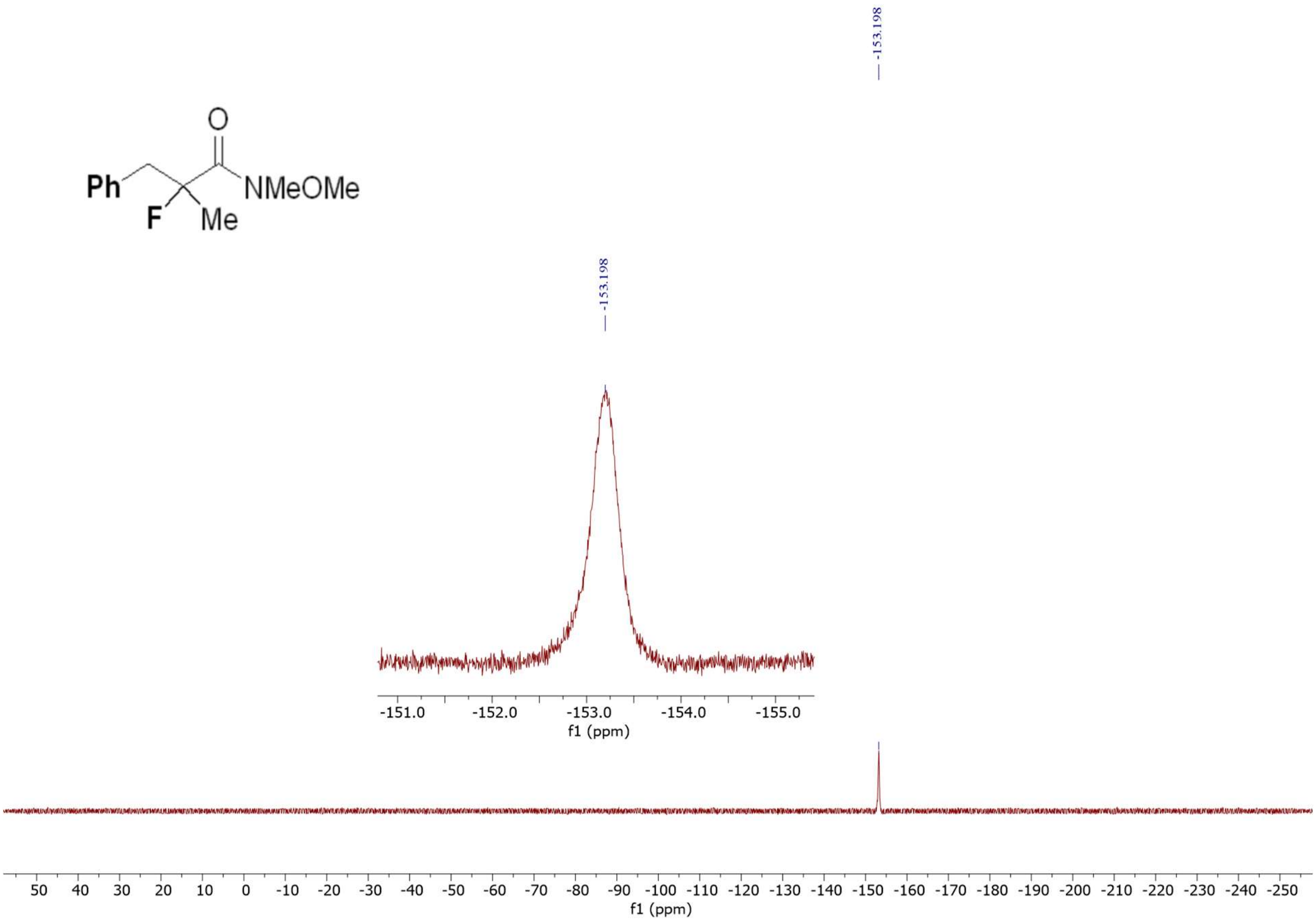

^{1}H NMR spectrum of **6n** (500 MHz, $CDCl_3$)

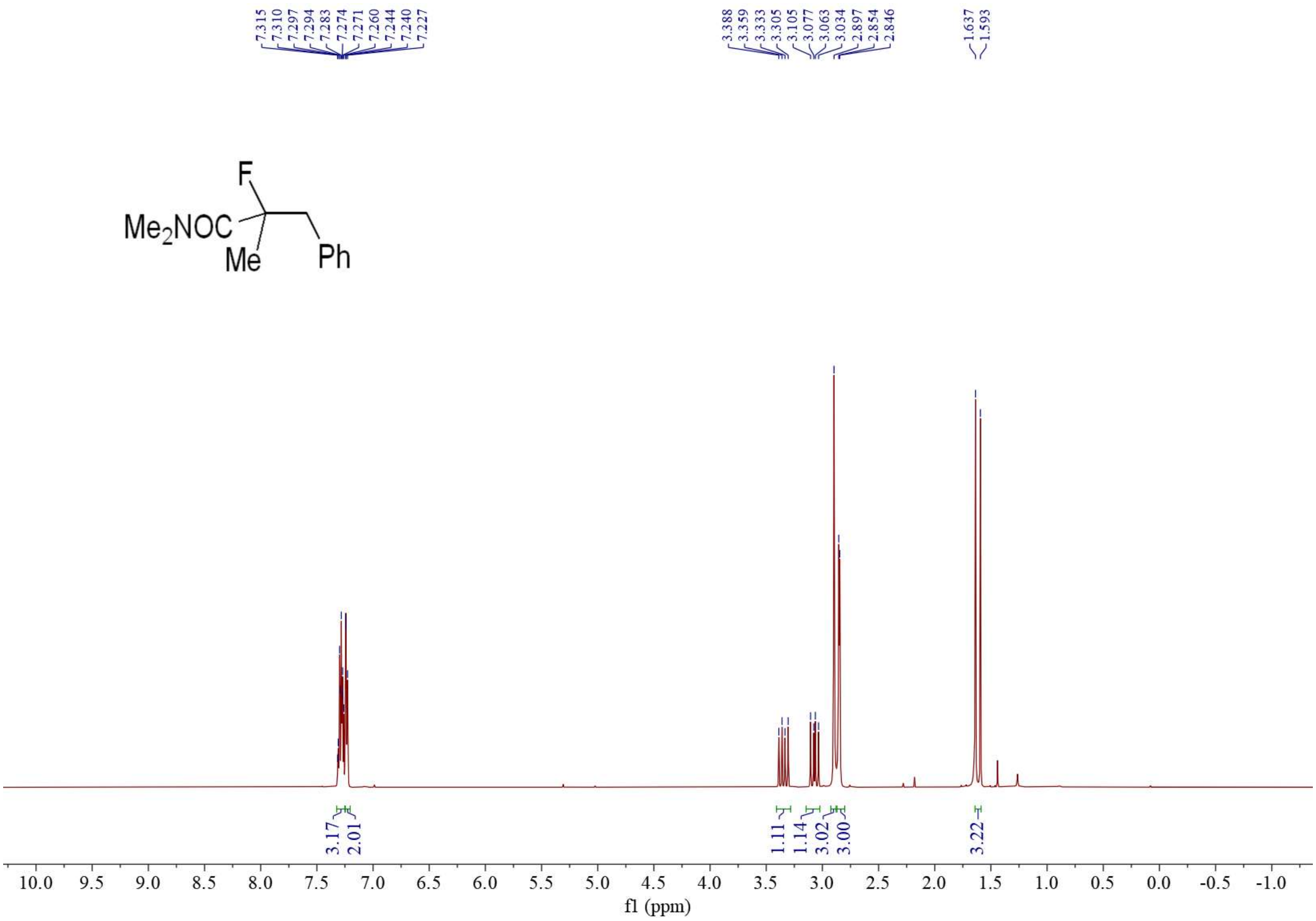

^{13}C NMR spectrum of **6n** (126 MHz, $CDCl_3$)

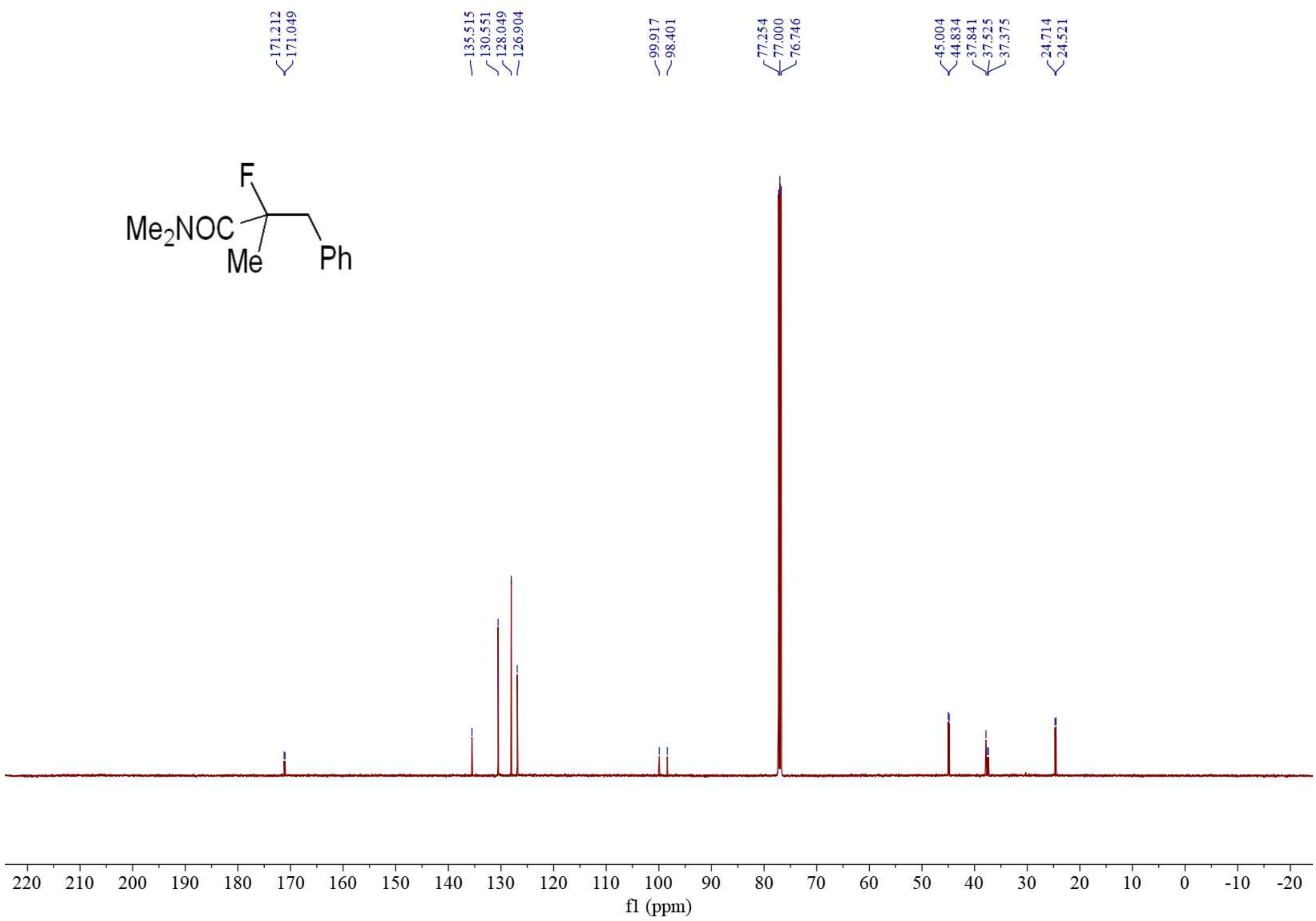

$^{19}F\{^{1}H\}$ NMR spectrum of **6n** (377 MHz, $CDCl_3$)

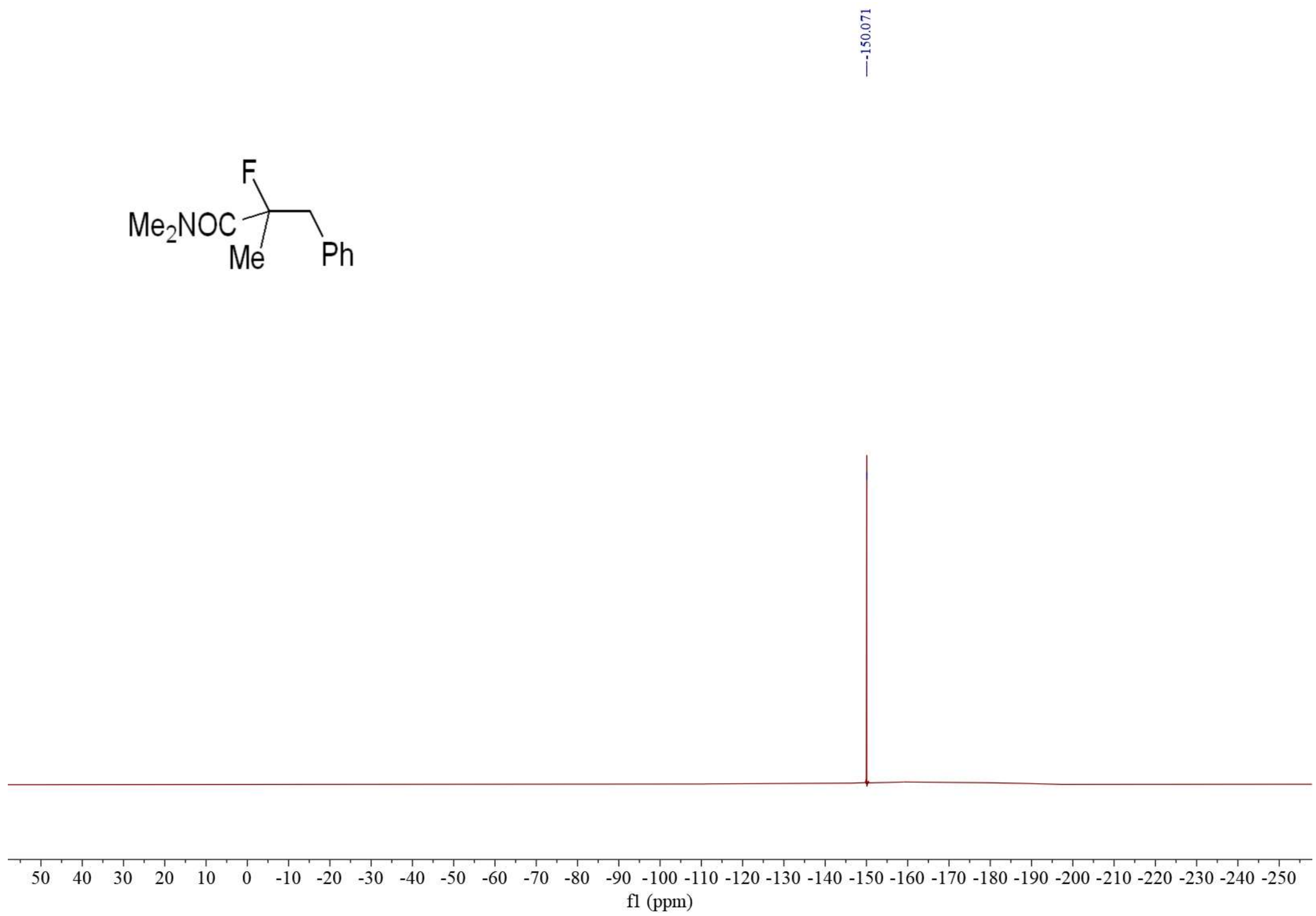

^{19}F NMR spectrum of **6n** (377 MHz, CDCl3)

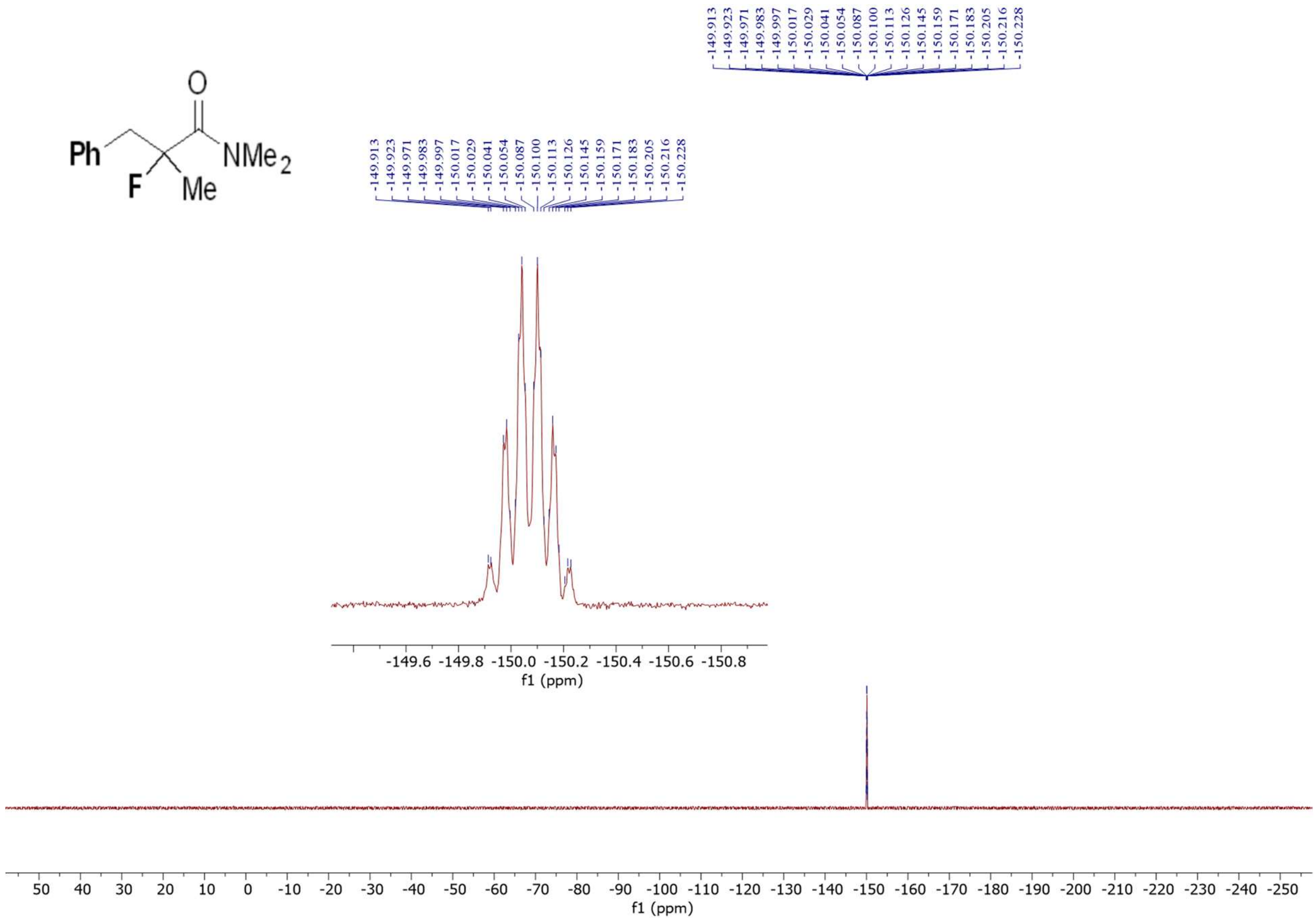

^{1}H NMR spectrum of **6o** (400 MHz, CDCl3)

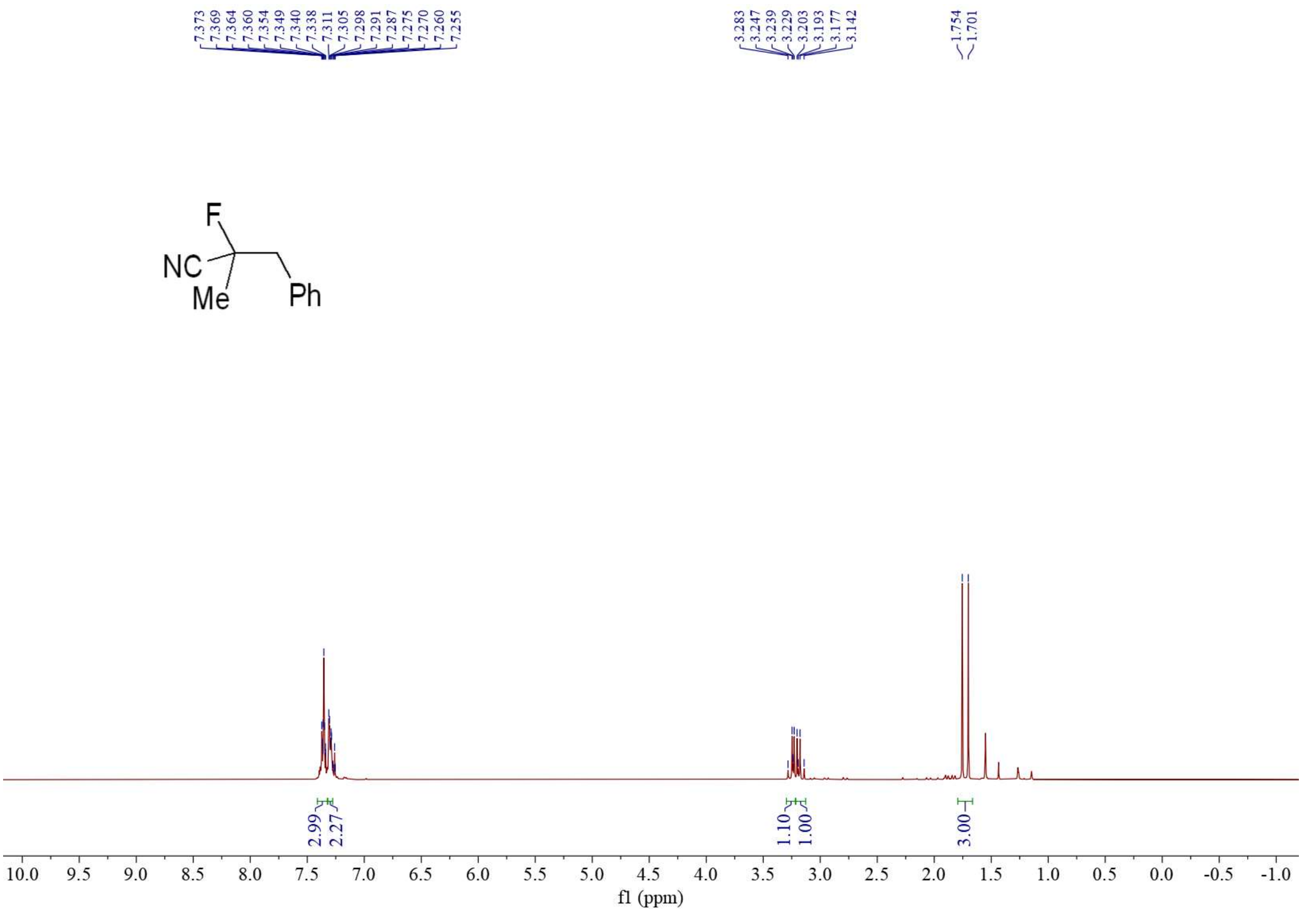

^{13}C NMR spectrum of **6o** (101 MHz, $CDCl_3$)

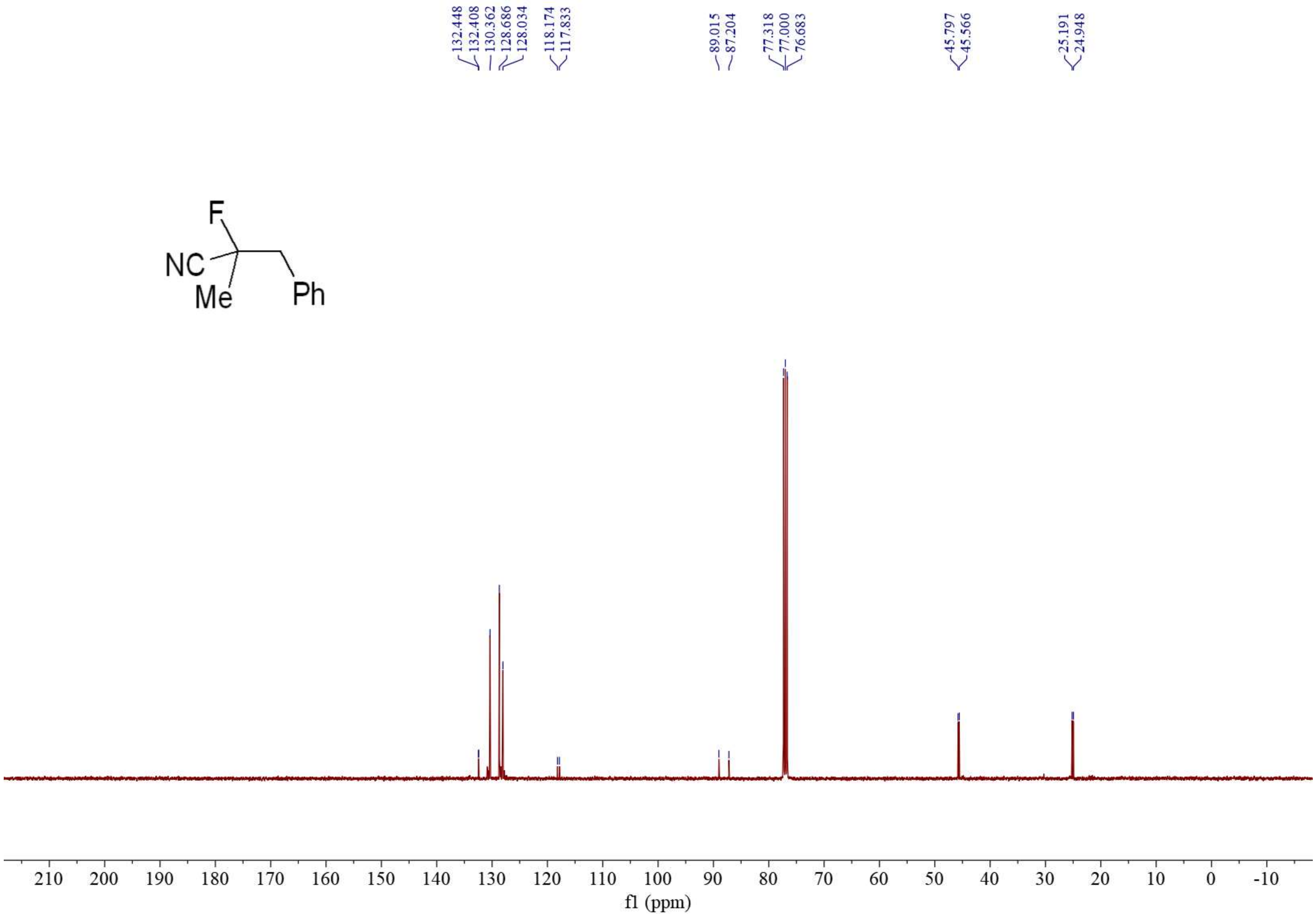

^{19}F{^{1}H} NMR spectrum of **6o** (377 MHz, $CDCl_3$)

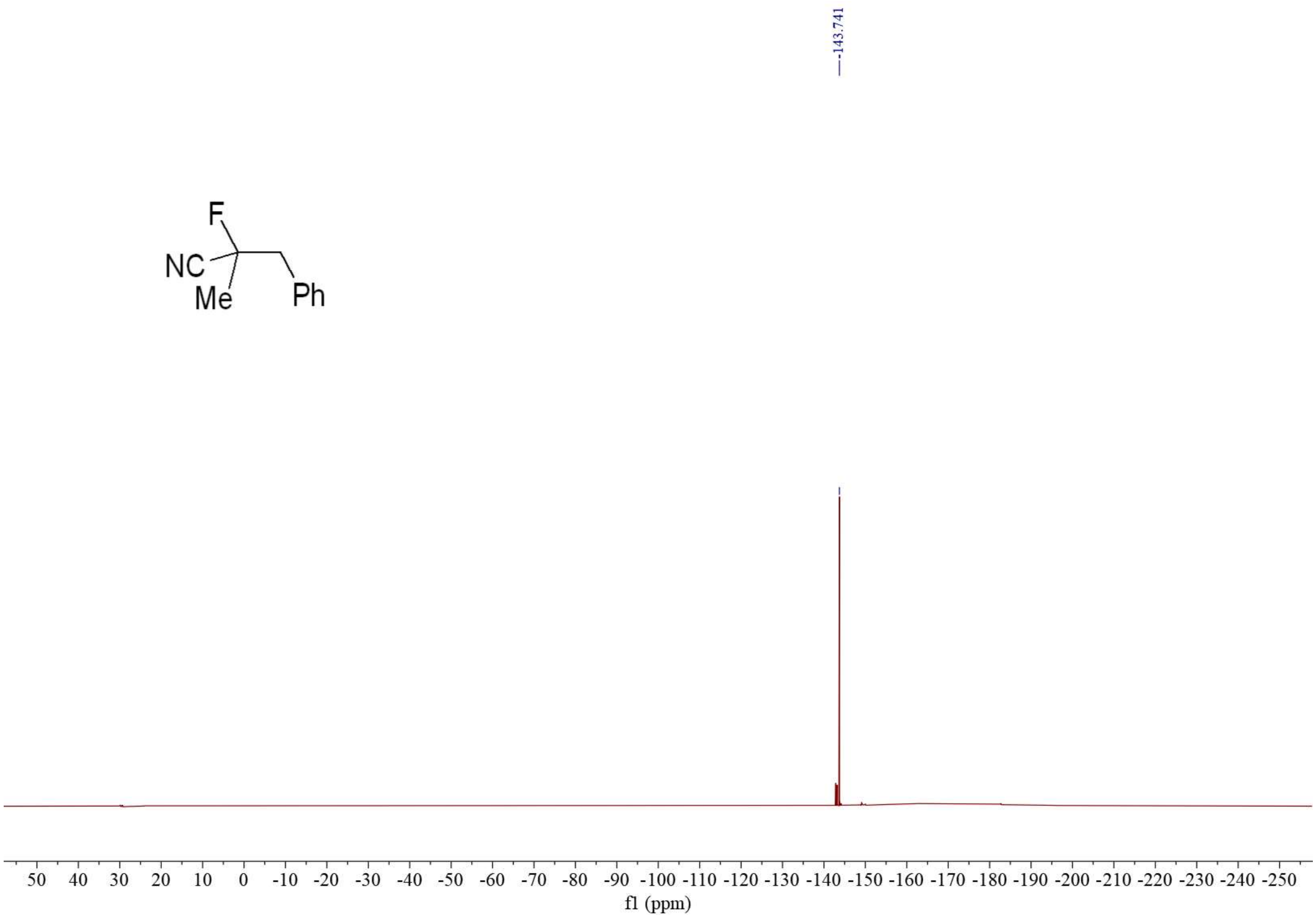

^{19}F NMR spectrum of **6o** (377 MHz, $CDCl_3$)

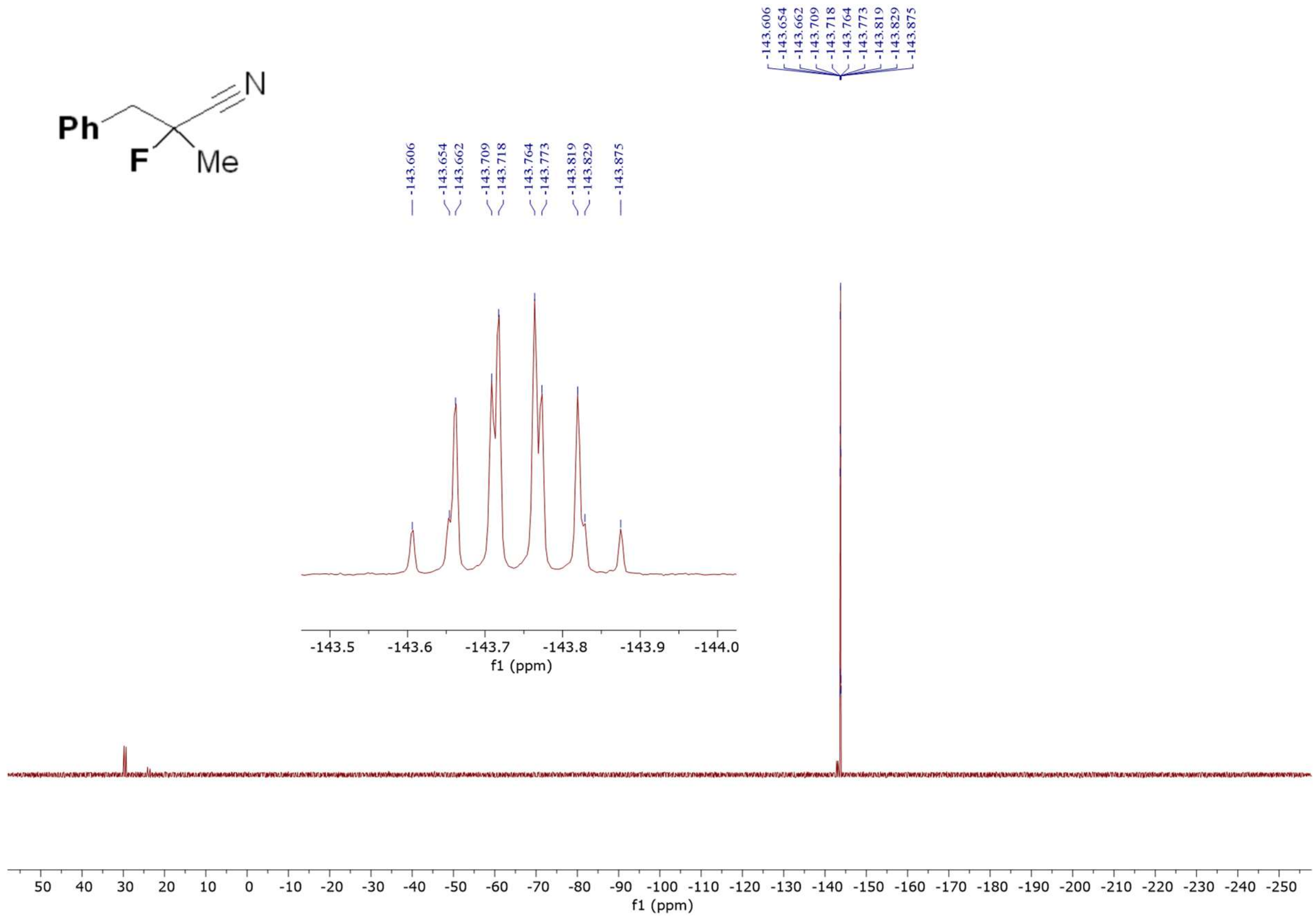

^{1}H NMR spectrum of **6p** (400 MHz, $CDCl_3$)

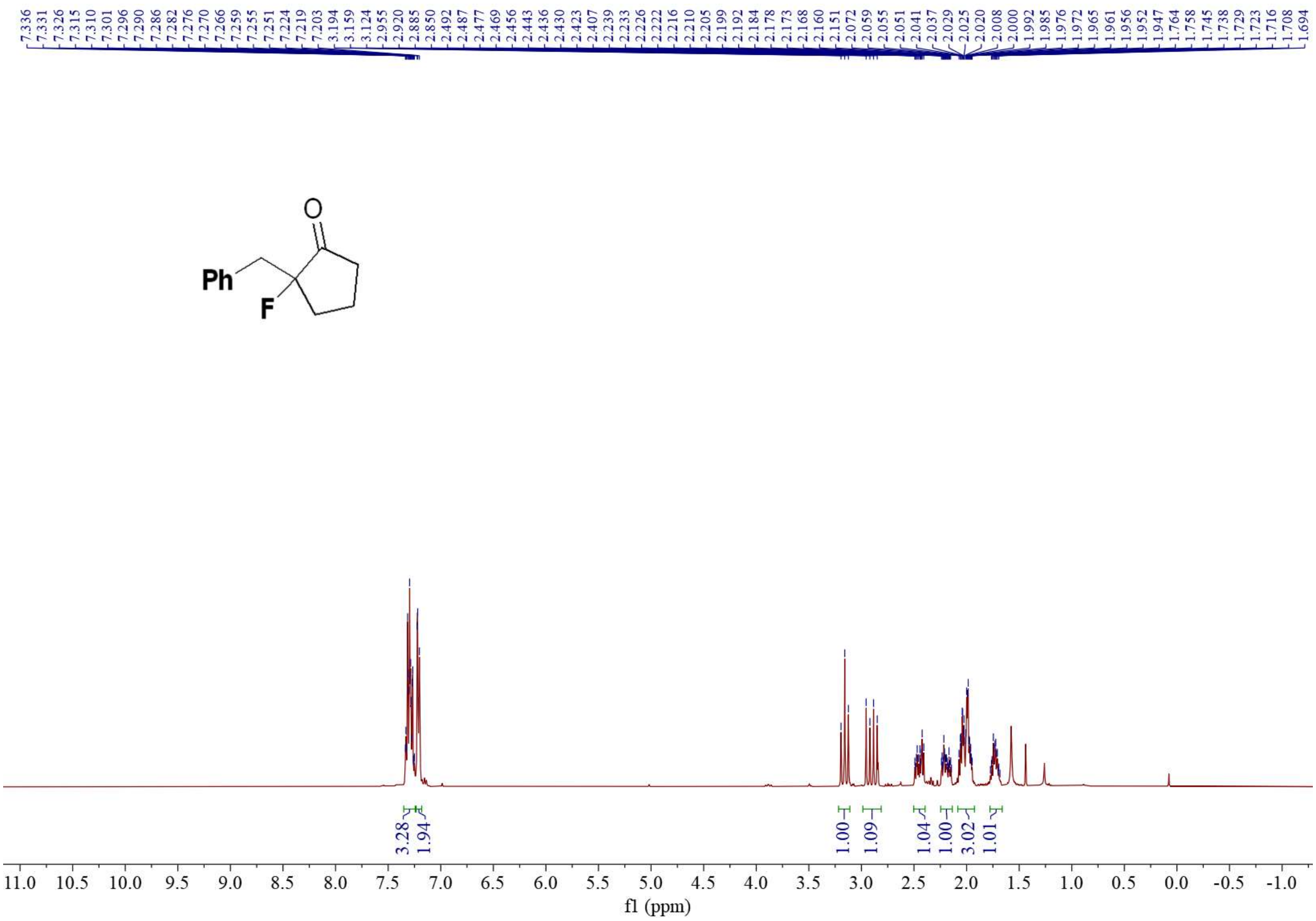

^{13}C NMR spectrum of **6p** (101 MHz, $CDCl_3$)

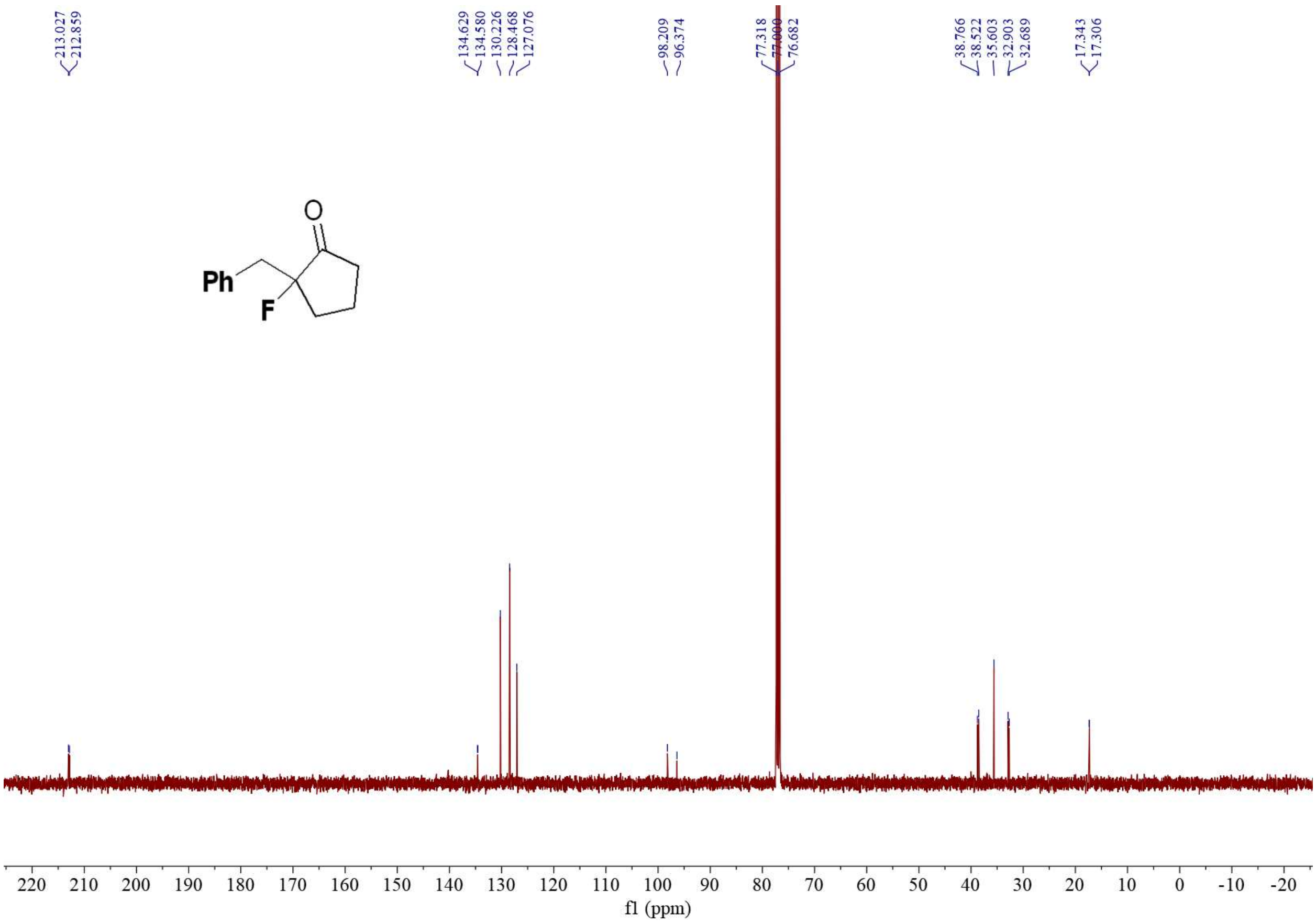

$^{19}F\{^1H\}$ NMR spectrum of **6p** (377 MHz, $CDCl_3$)

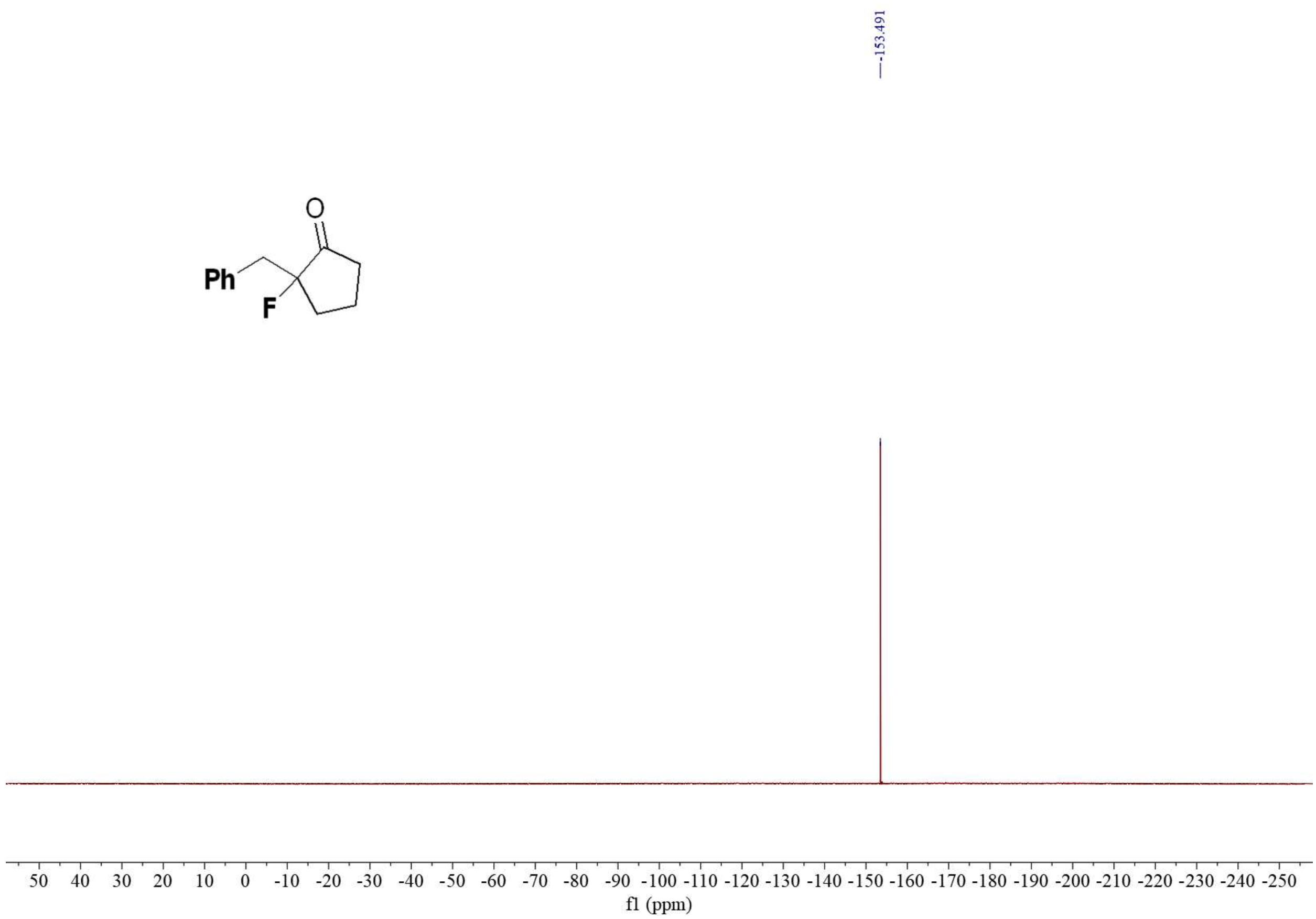

^{19}F NMR spectrum of **6p** (377 MHz, $CDCl_3$)

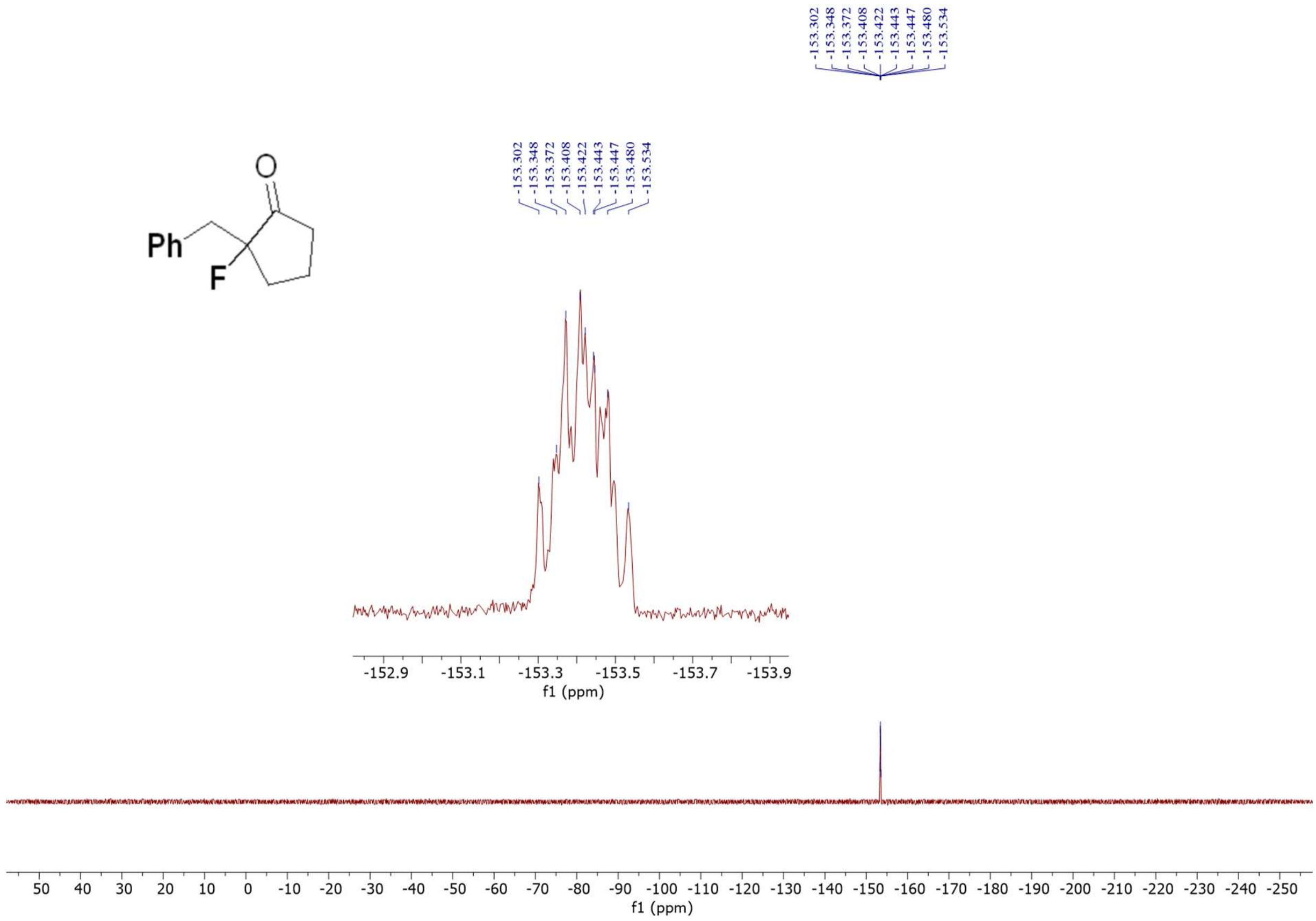

^{1}H NMR spectrum of **6q** (600 MHz, $CDCl_3$)

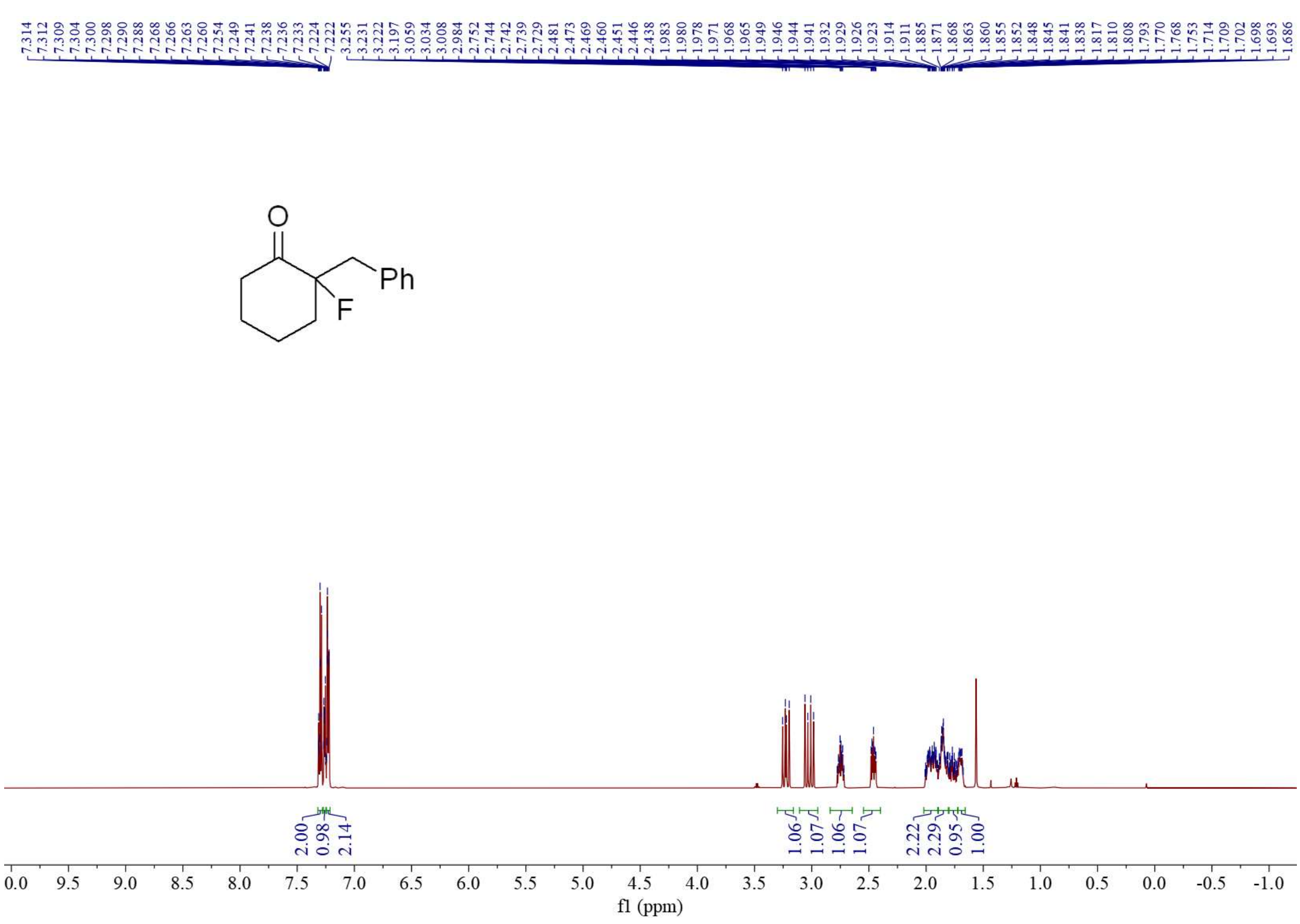

^{13}C NMR spectrum of **6q** (151 MHz, $CDCl_3$)

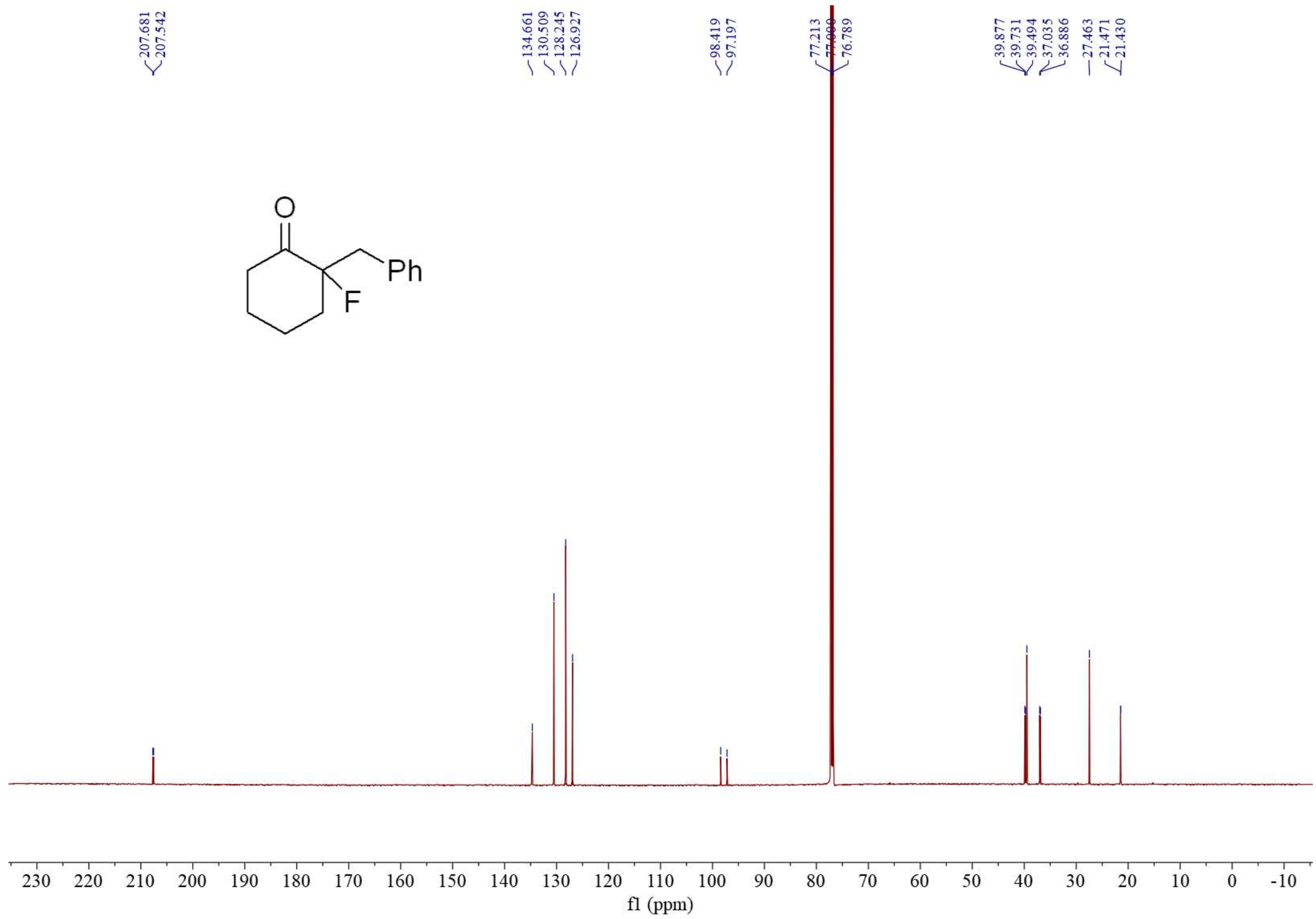

$^{19}F\{^1H\}$ NMR spectrum of **6q** (377 MHz, $CDCl_3$)

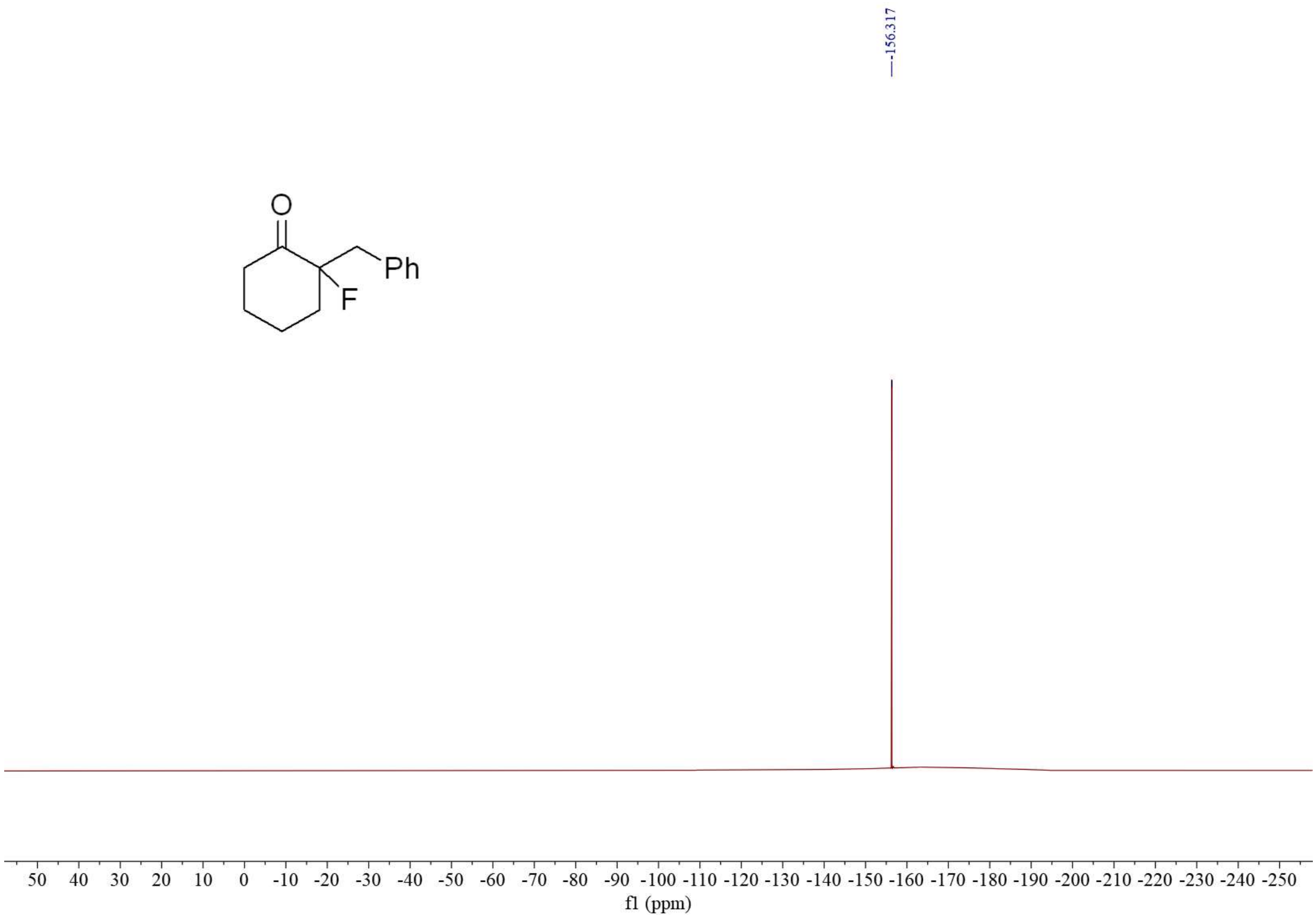

^{19}F NMR spectrum of **6q** (377 MHz, $CDCl_3$)

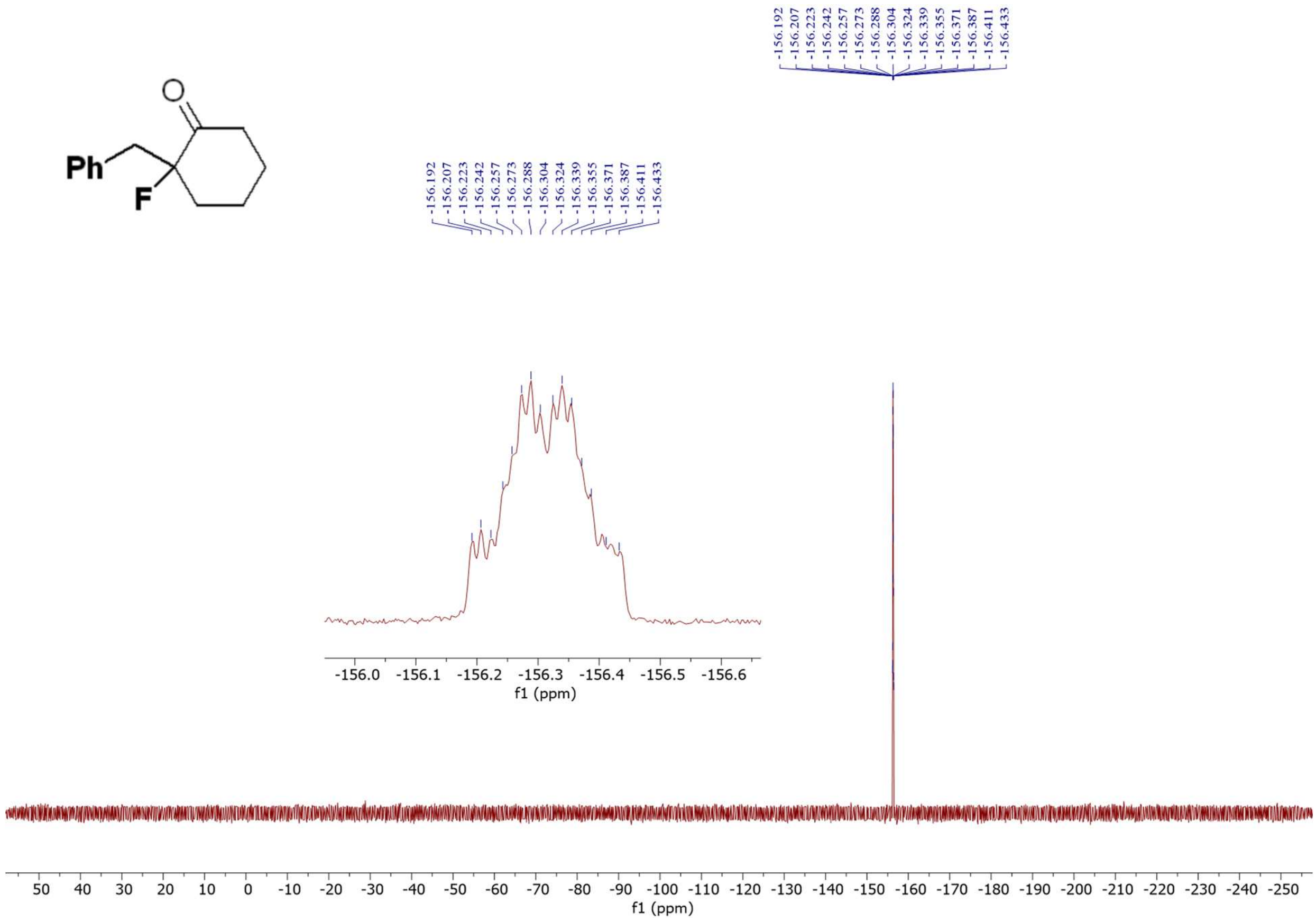

^{1}H NMR spectrum of **6r** (400 MHz, $CDCl_3$)

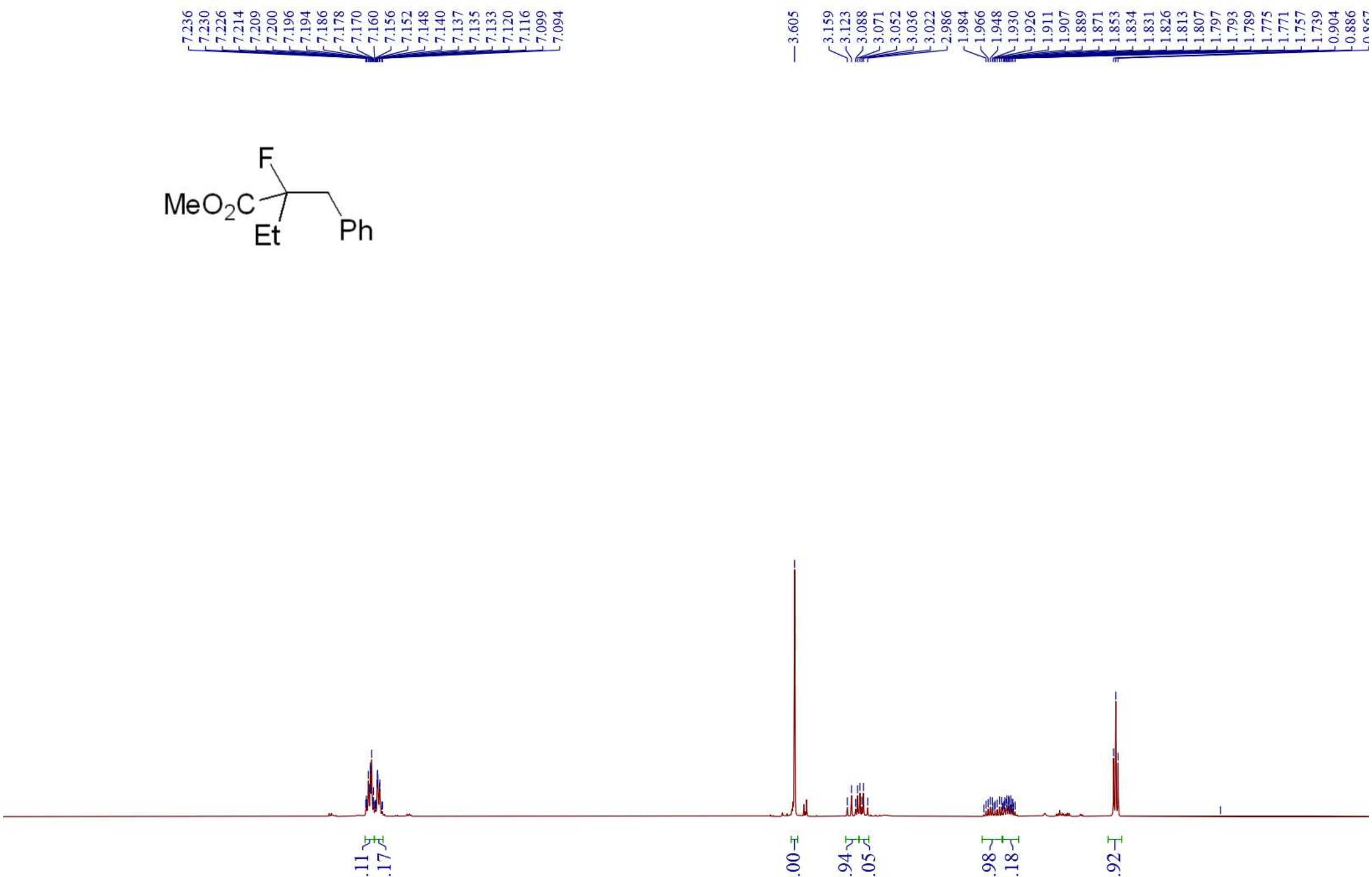

^{13}C NMR spectrum of **6r** (101 MHz, $CDCl_3$)

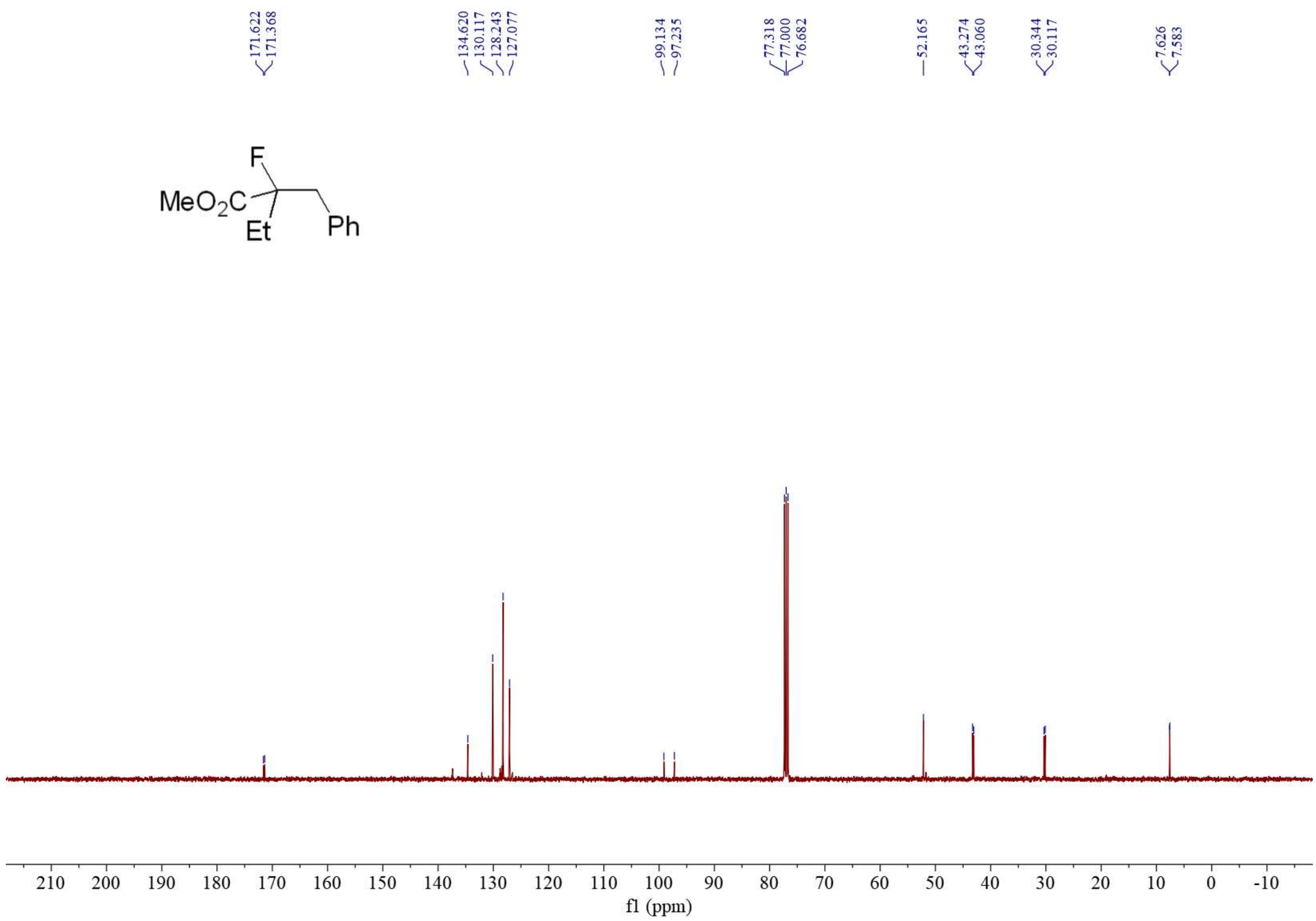

$^{19}F\{^1H\}$ NMR spectrum of **6r** (377 MHz, $CDCl_3$)

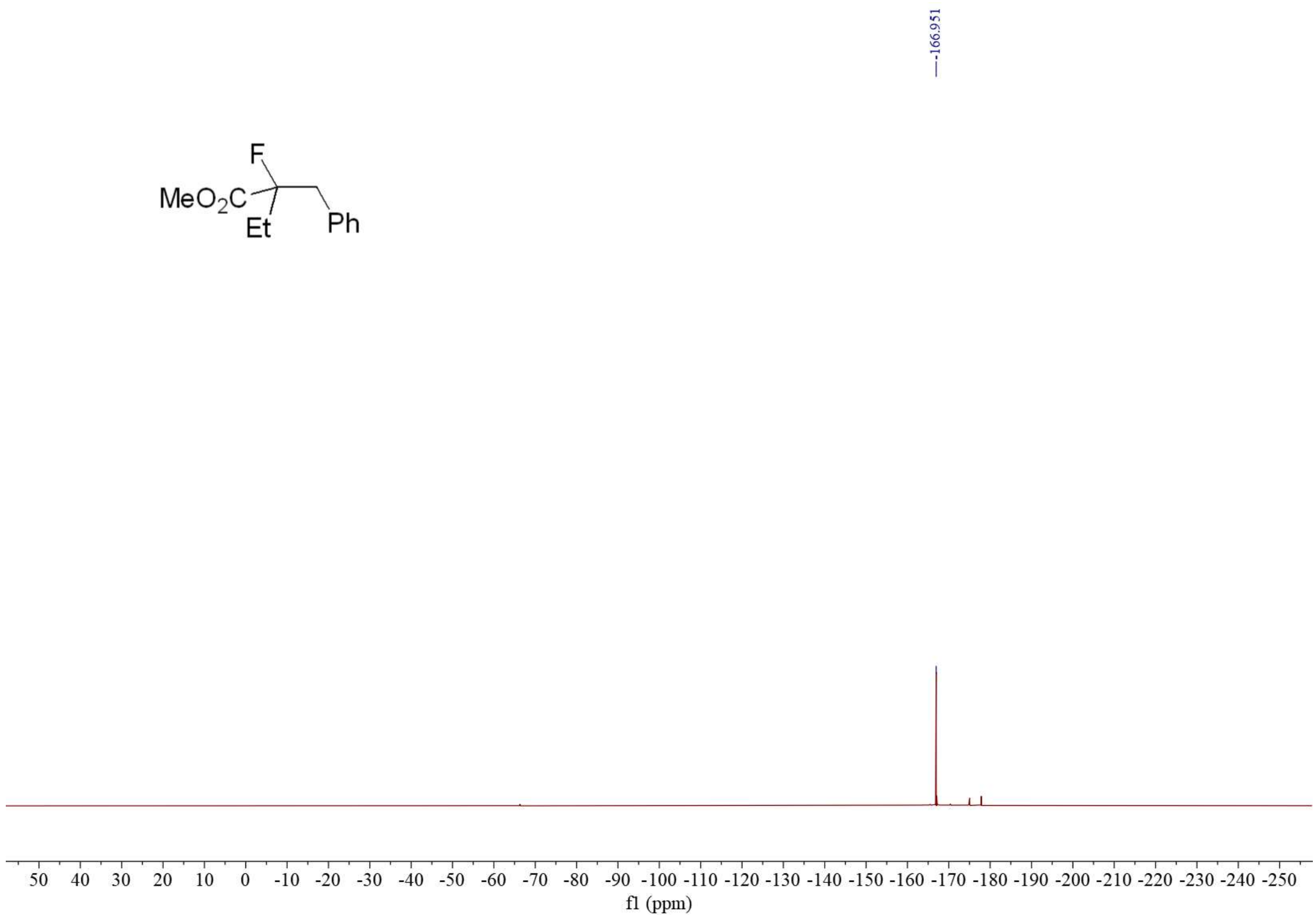

^{19}F NMR spectrum of **6r** (377 MHz, $CDCl_3$)

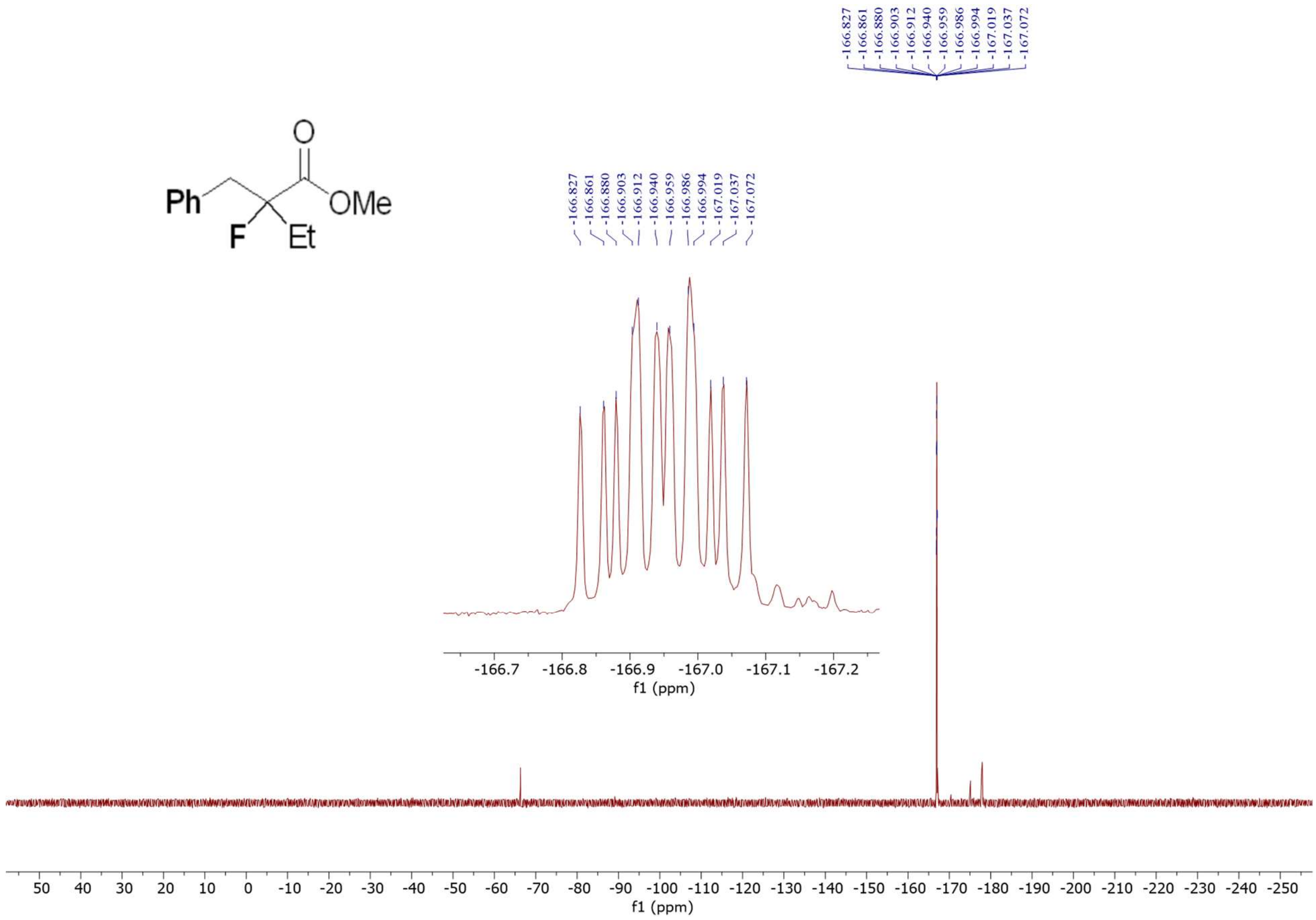

^{1}H NMR spectrum of **6s** (400 MHz, $CDCl_3$)

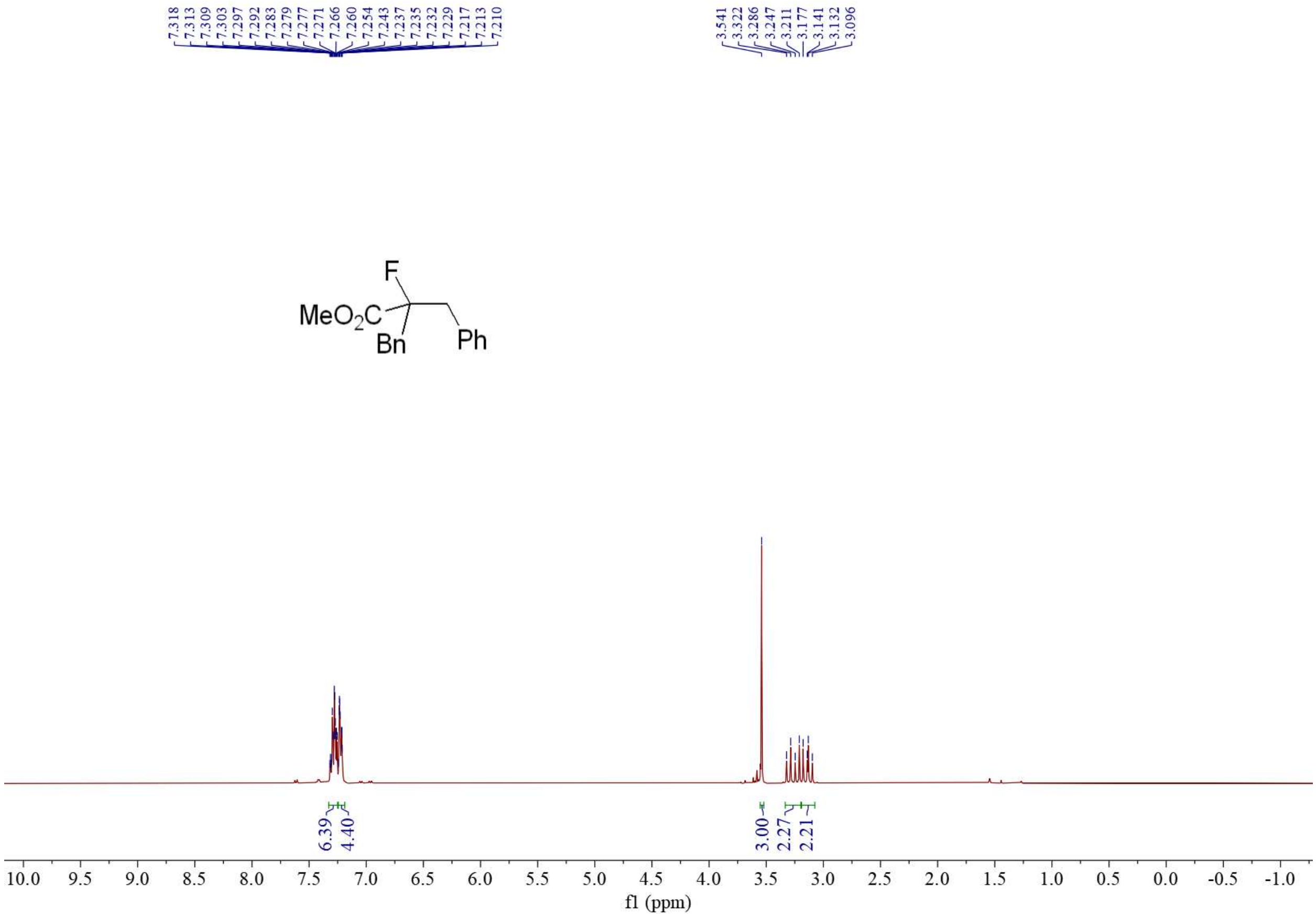

^{13}C NMR spectrum of **6s** (101 MHz, $CDCl_3$)

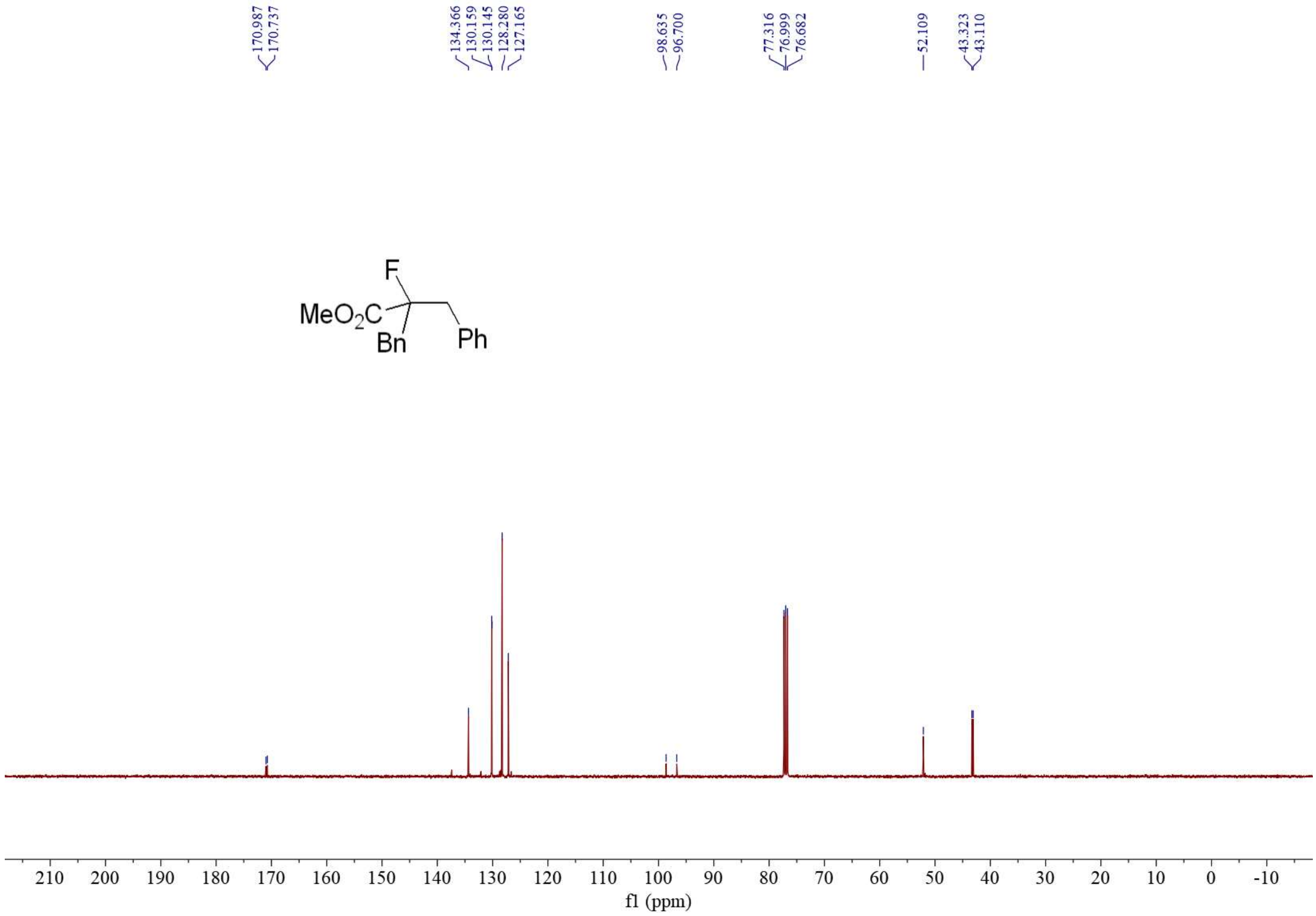

$^{19}F\{^{1}H\}$ NMR spectrum of **6s** (377 MHz, $CDCl_3$)

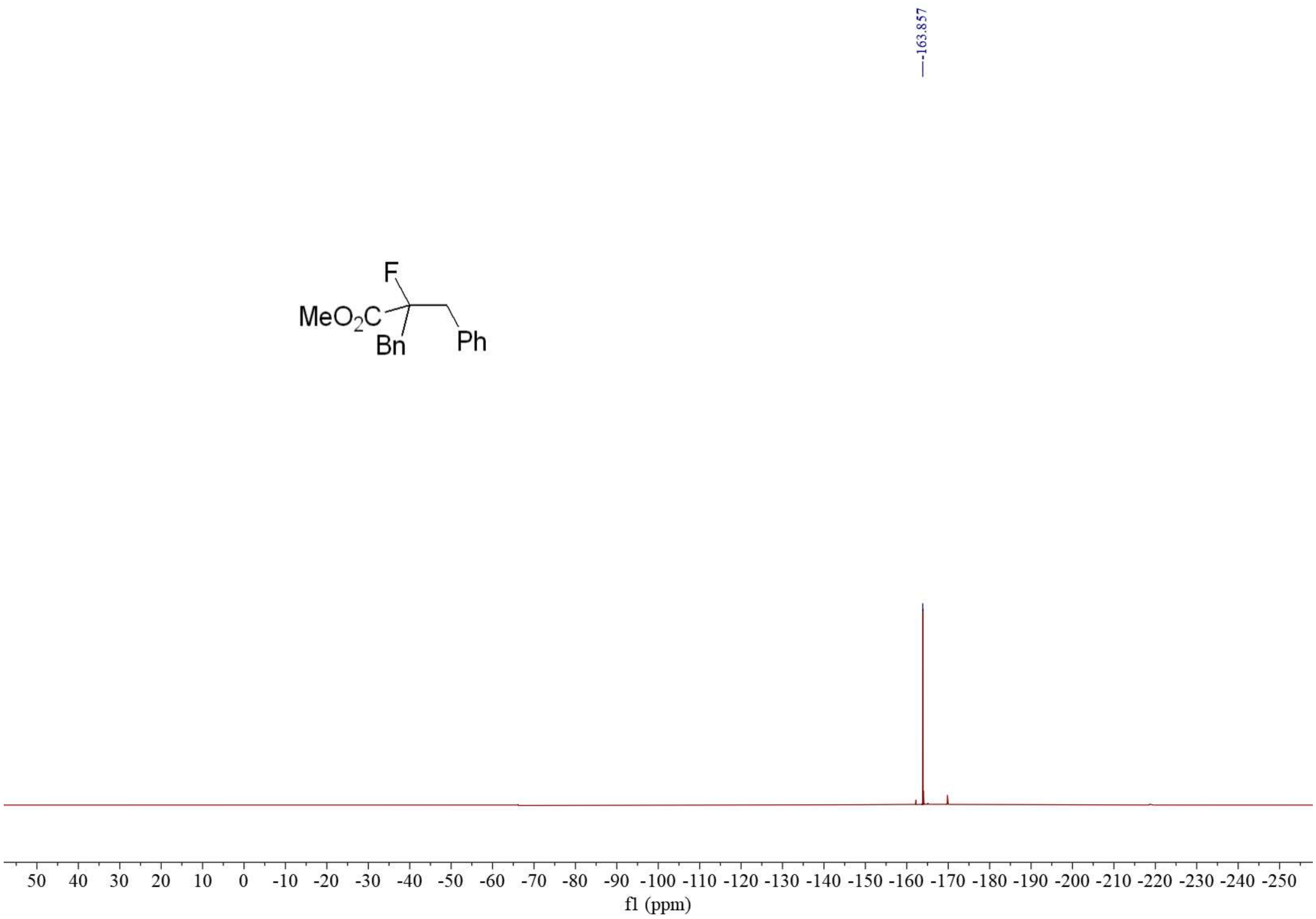

^{19}F NMR spectrum of **6s** (377 MHz, $CDCl_3$)

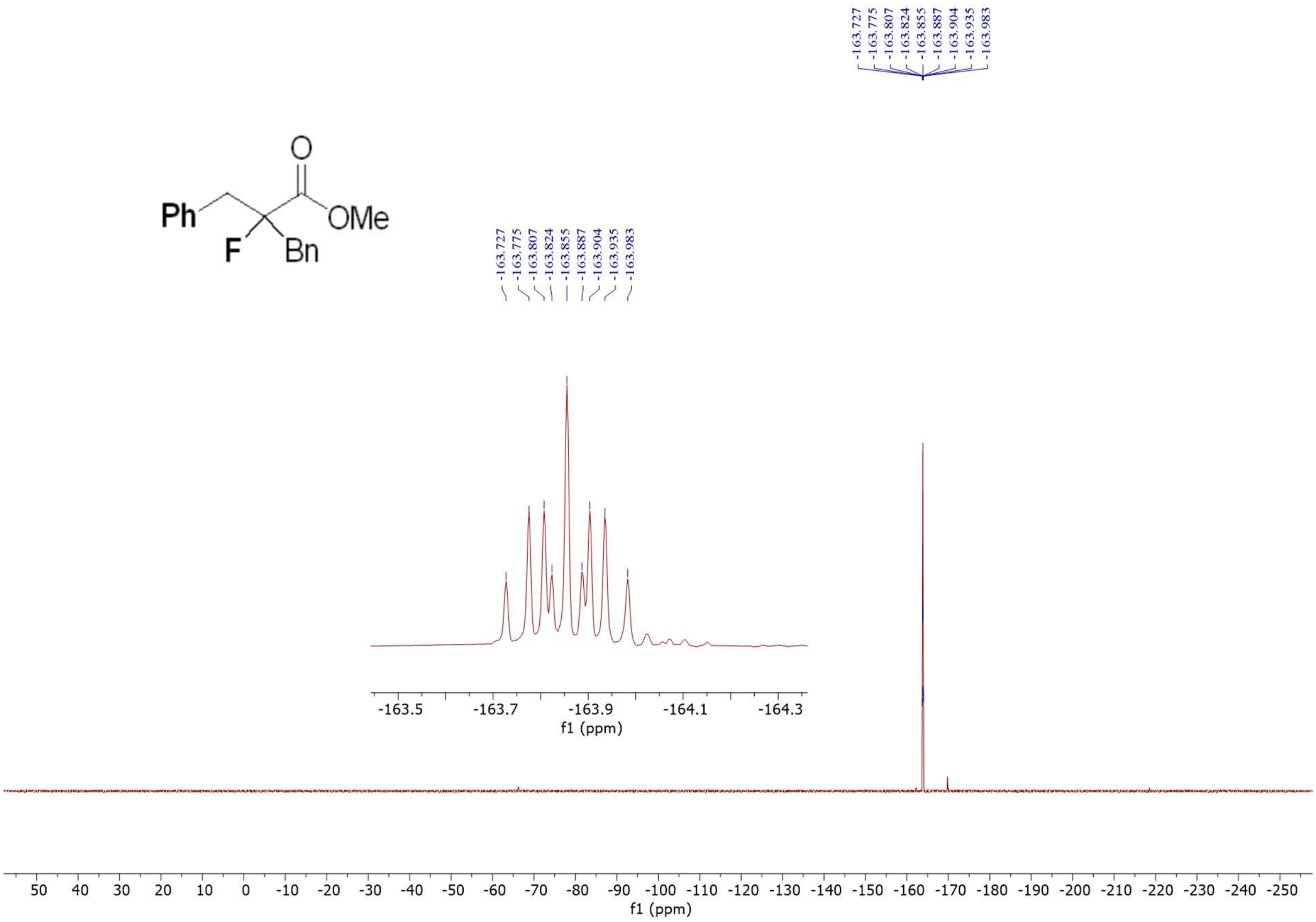

^{1}H NMR spectrum of **6t** (400 MHz, $CDCl_3$)

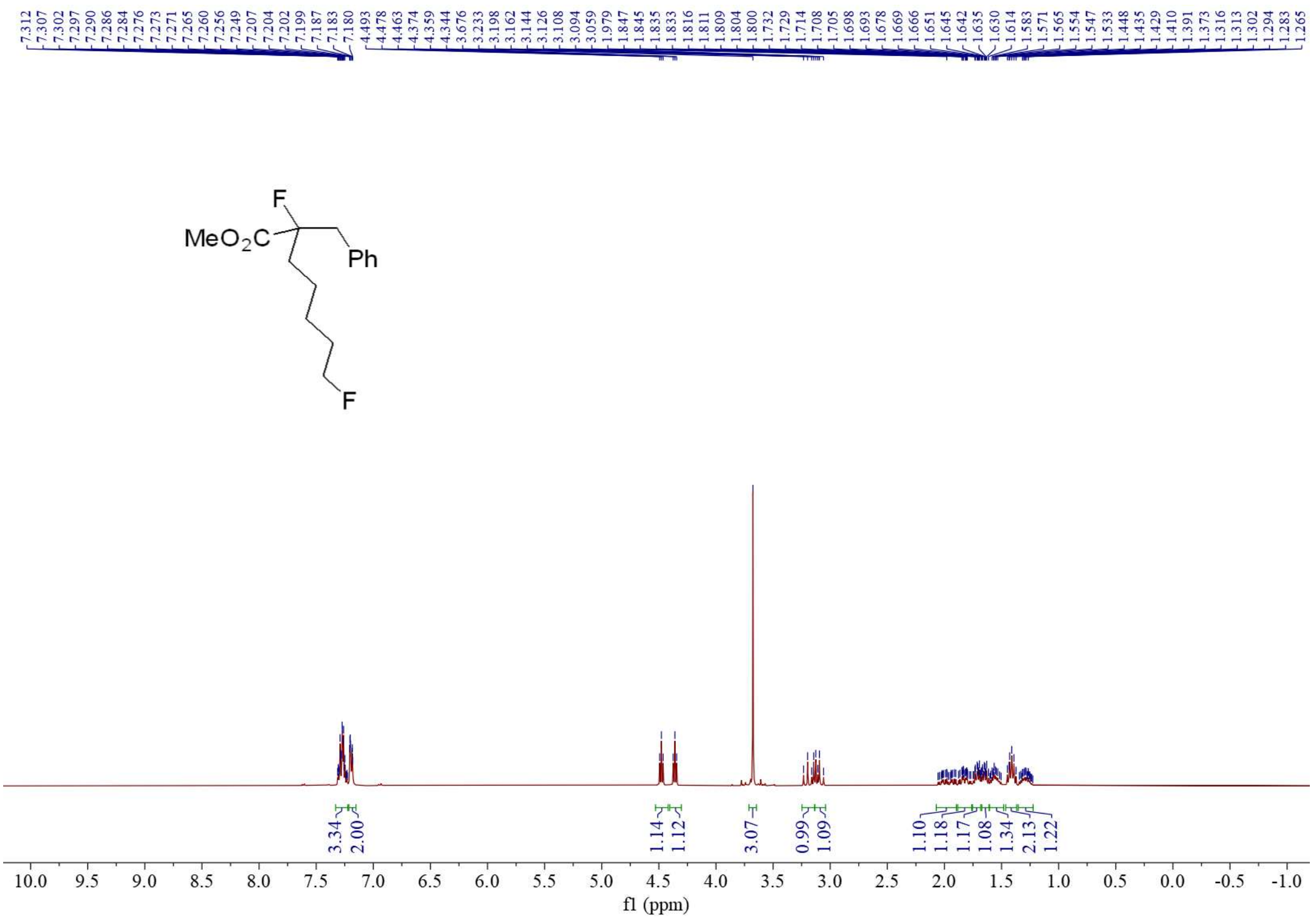

^{13}C NMR spectrum of **6t** (101 MHz, $CDCl_3$)

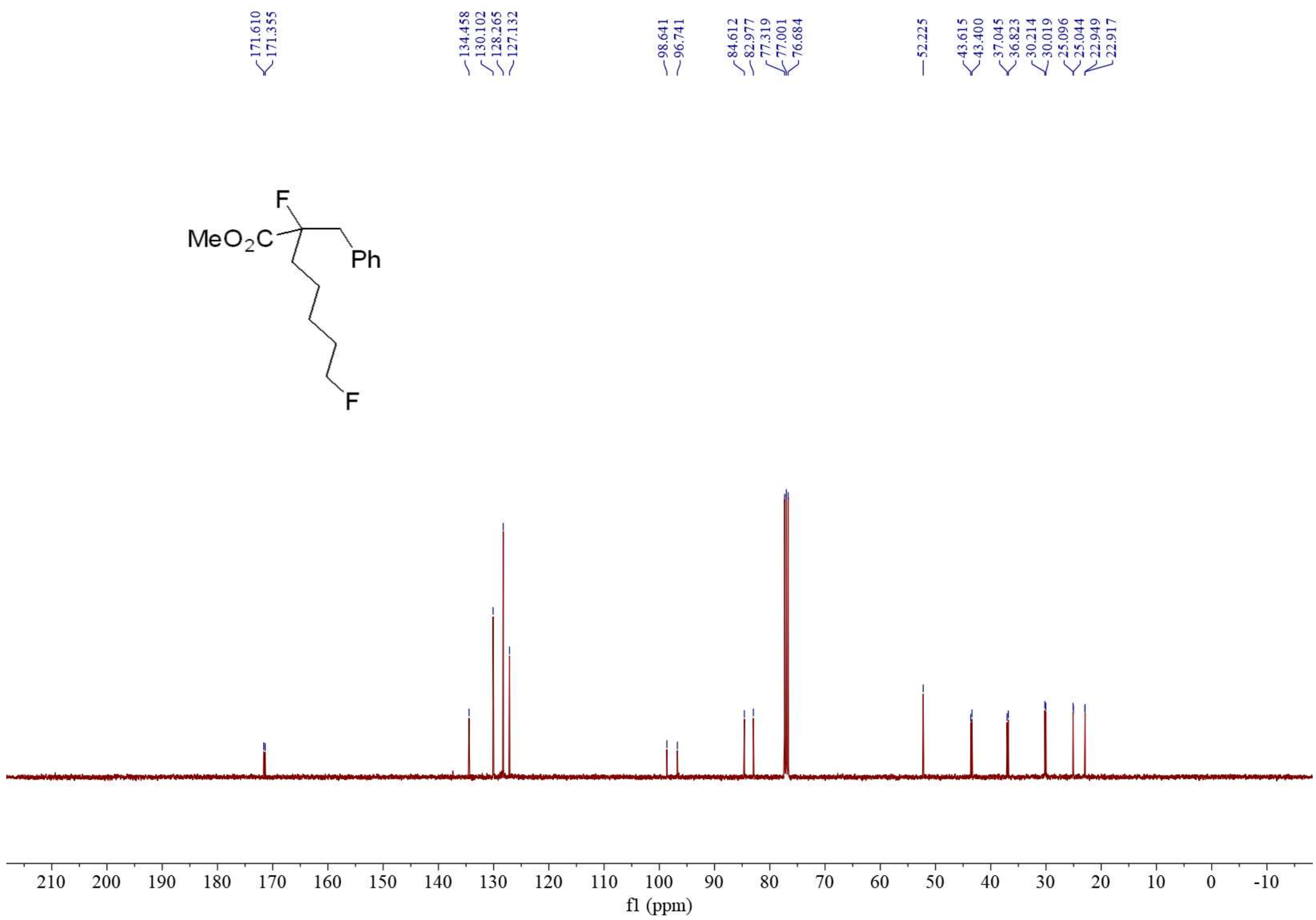

^{19}F{^{1}H} NMR spectrum of **6t** (377 MHz, $CDCl_3$)

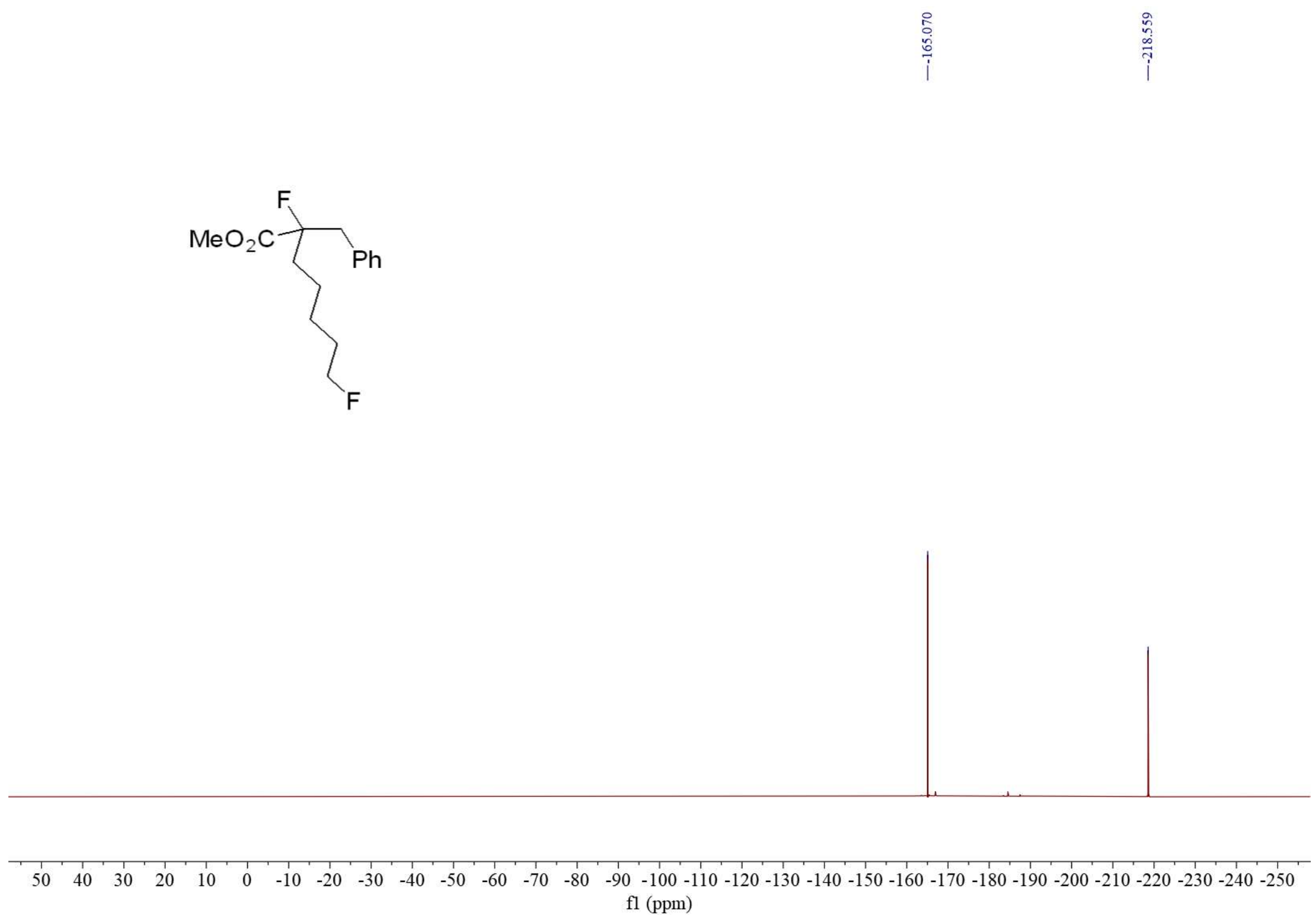

^{19}F NMR spectrum of **6t** (377 MHz, $CDCl_3$)

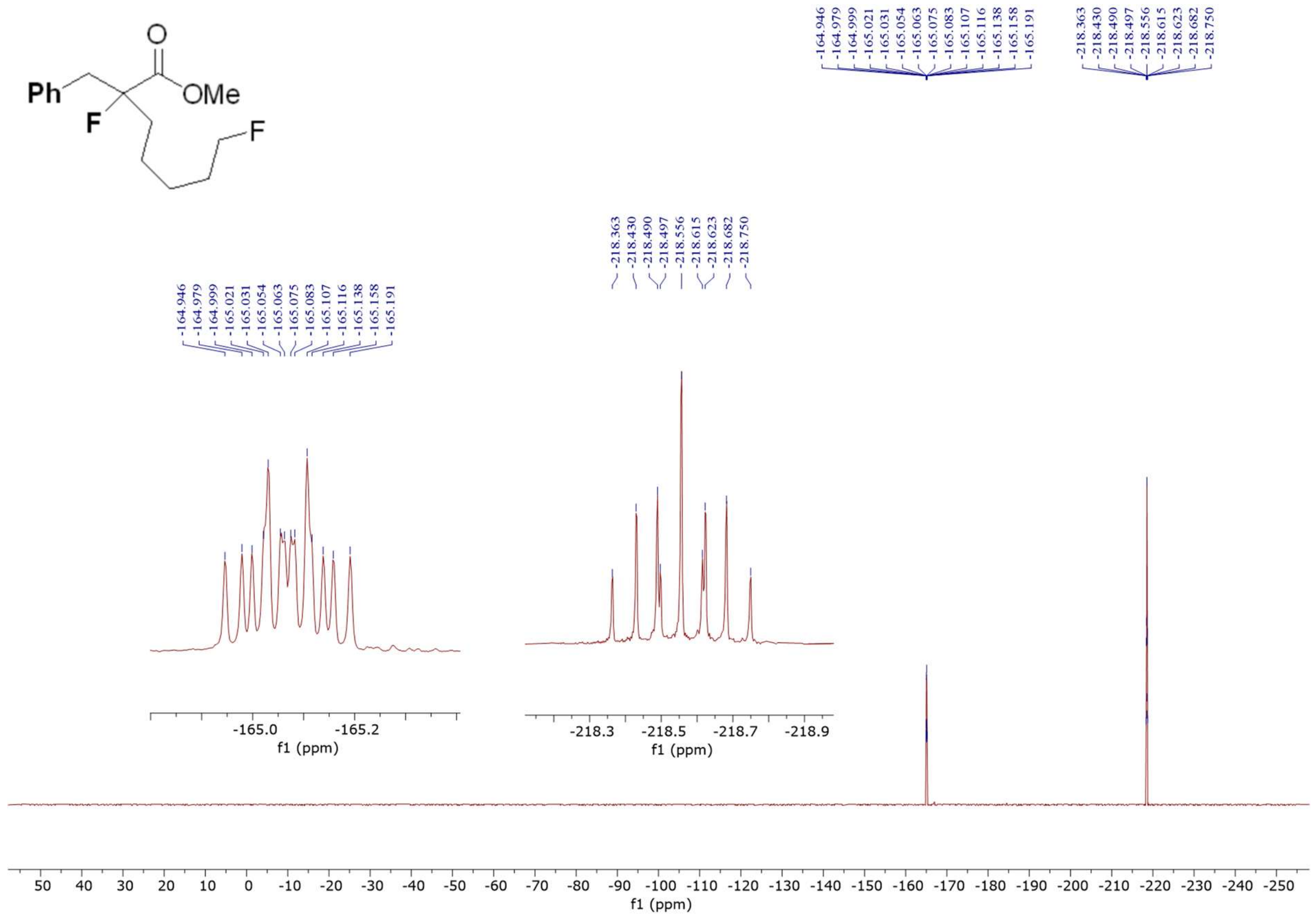

^{1}H NMR spectrum of **6u** (400 MHz, $CDCl_3$)

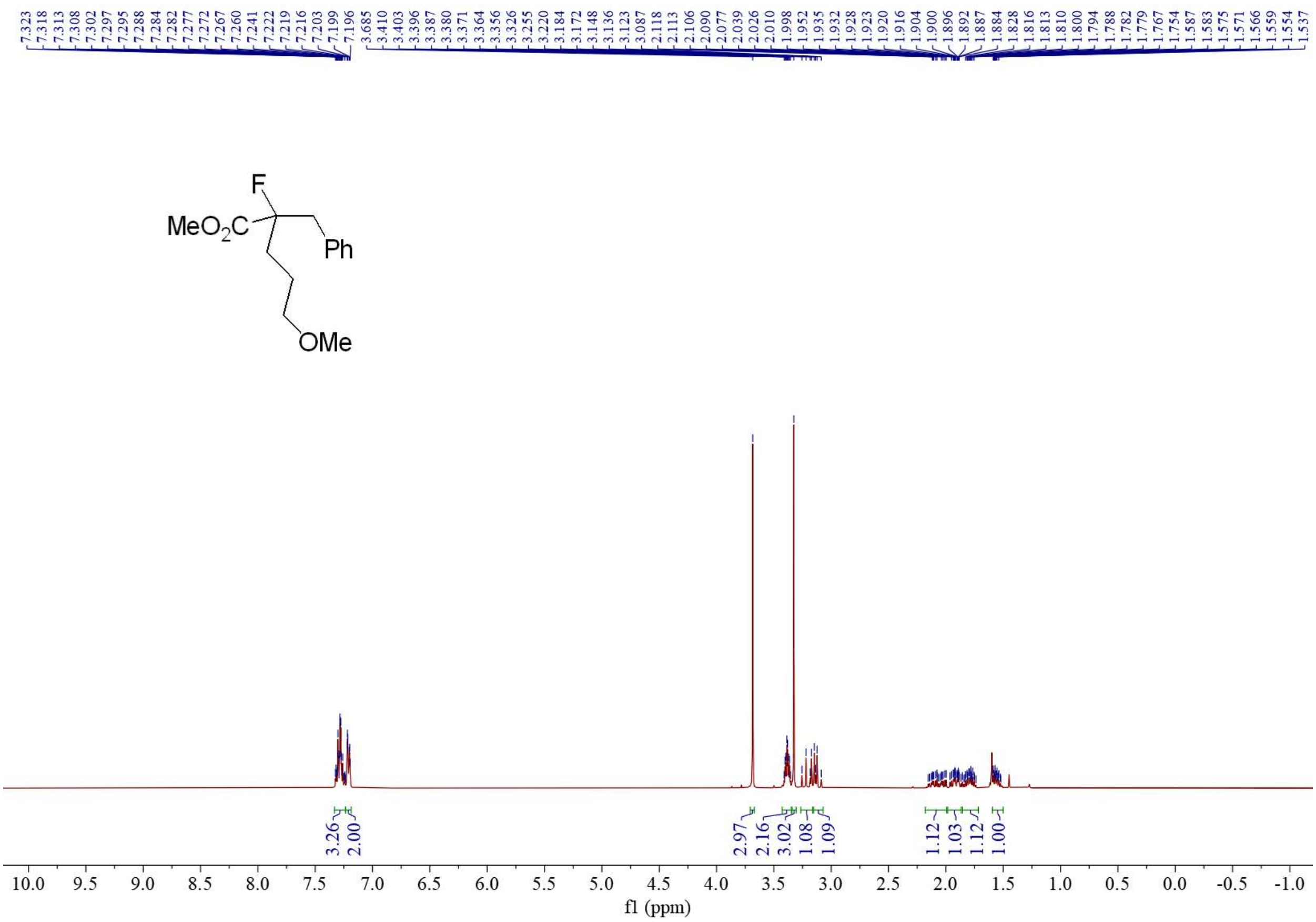

^{13}C NMR spectrum of **6u** (101 MHz, $CDCl_3$)

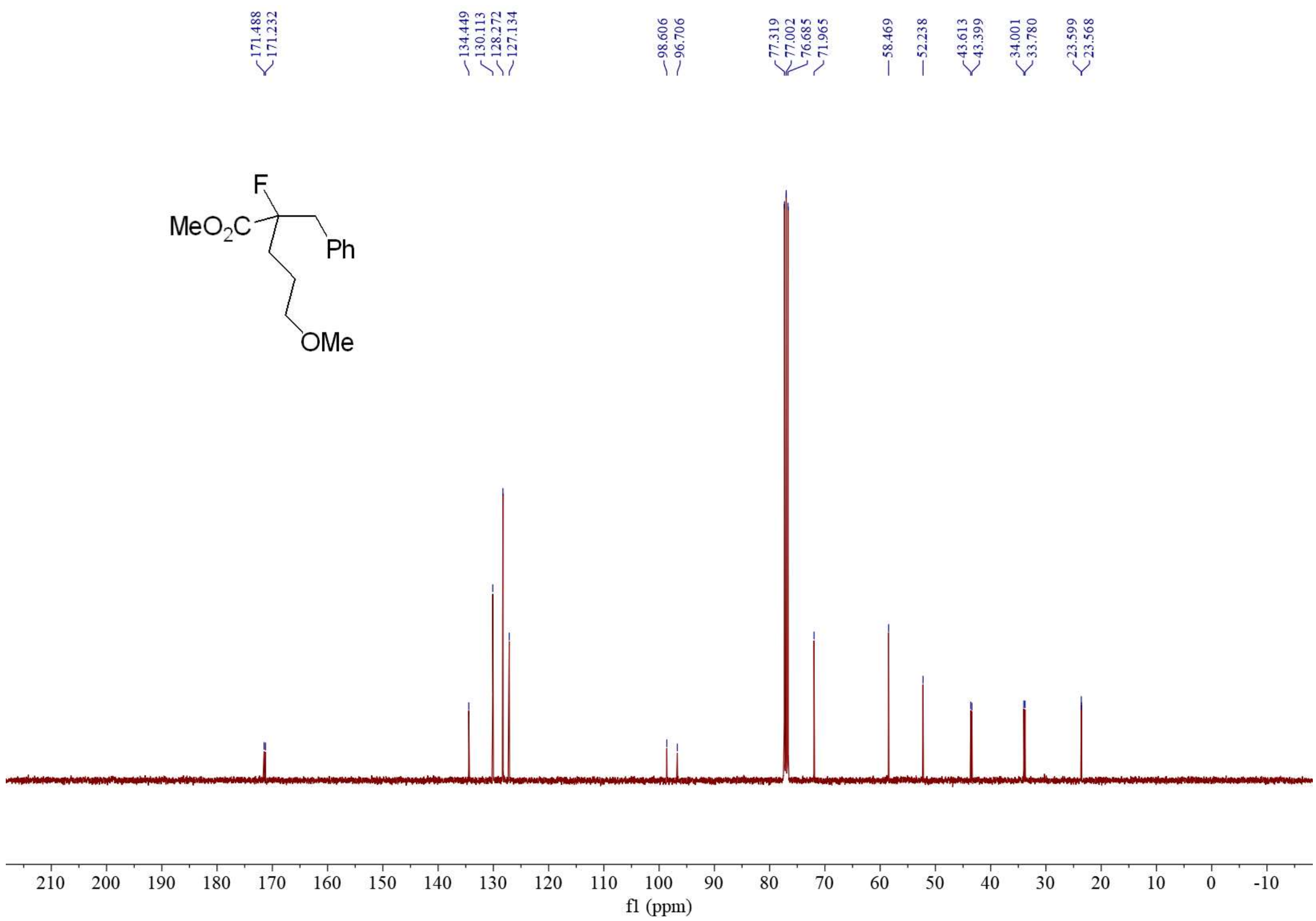

$^{19}F\{^{1}H\}$ NMR spectrum of **6u** (377 MHz, $CDCl_3$)

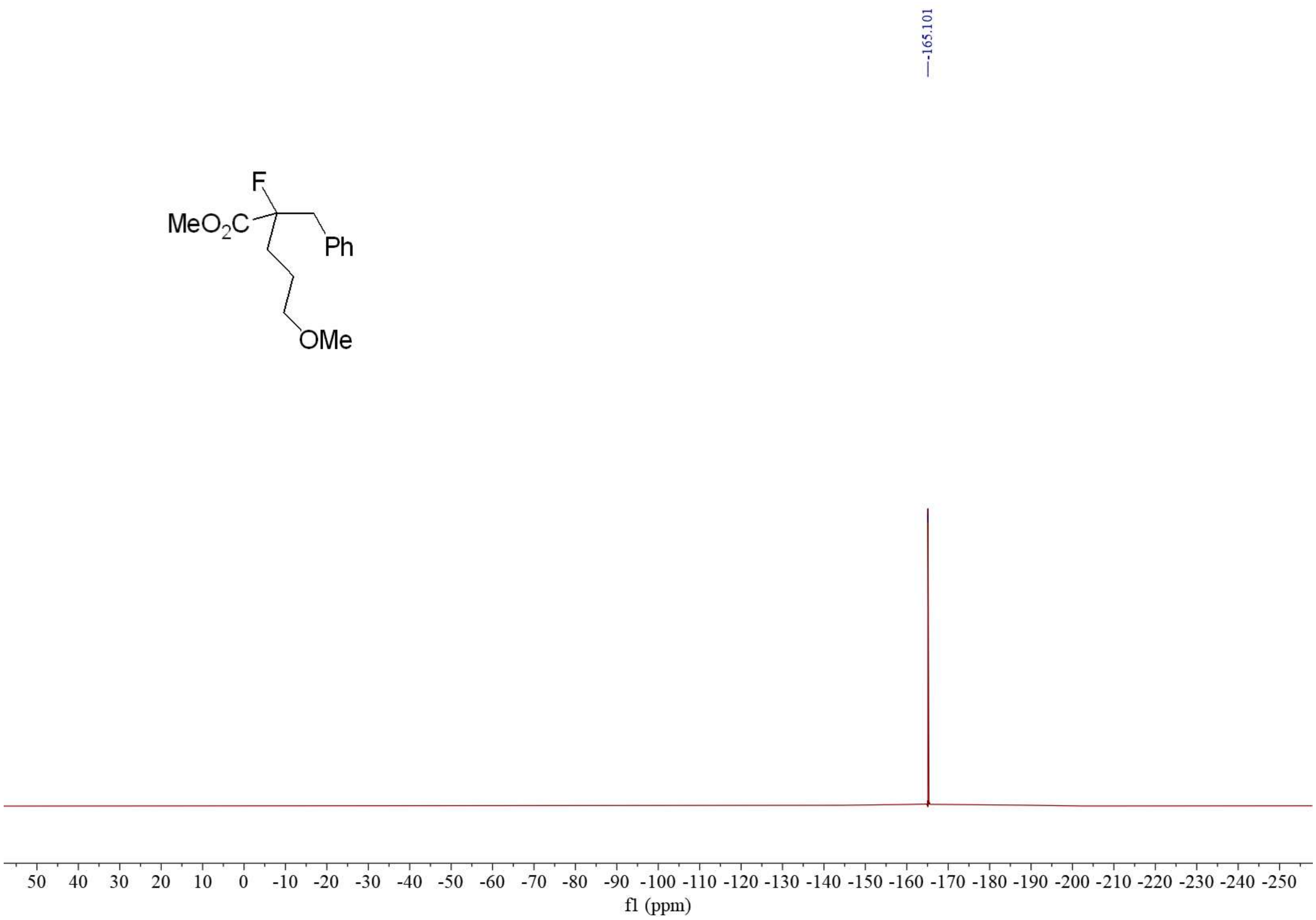

^{19}F NMR spectrum of **6u** (377 MHz, $CDCl_3$)

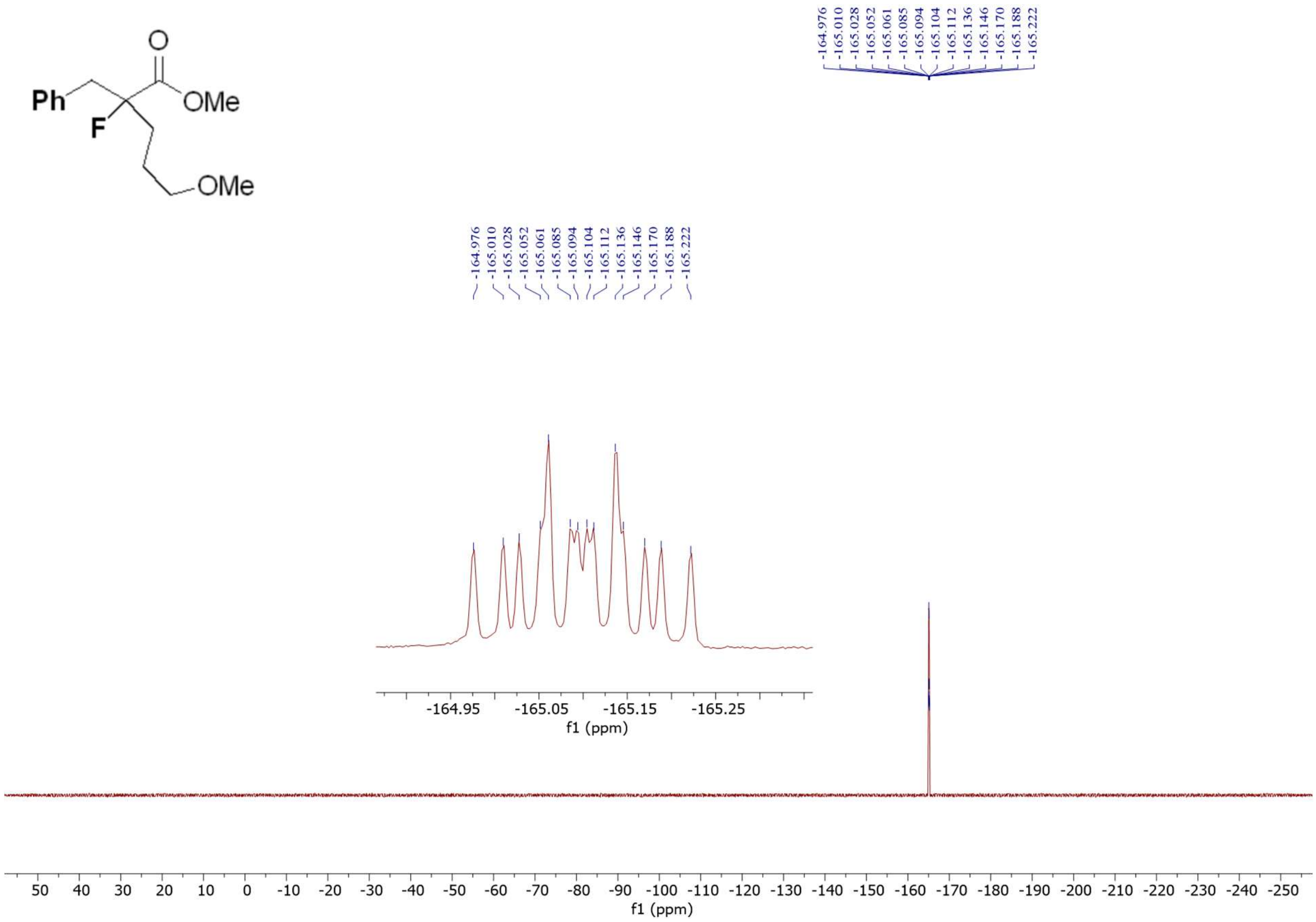

^{1}H NMR spectrum of **6v** (500 MHz, $CDCl_3$)

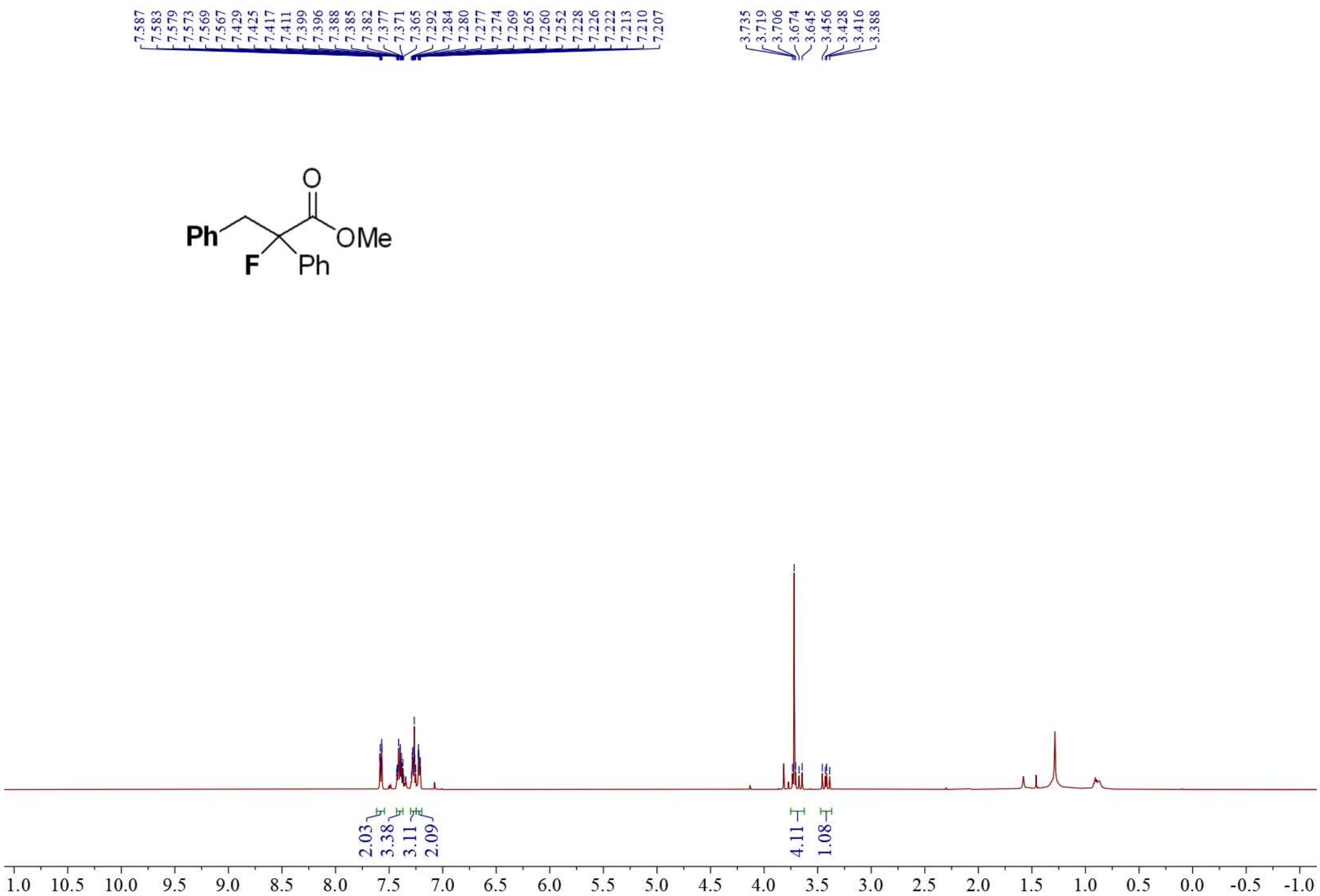

^{13}C NMR spectrum of **6v** (126 MHz, $CDCl_3$)

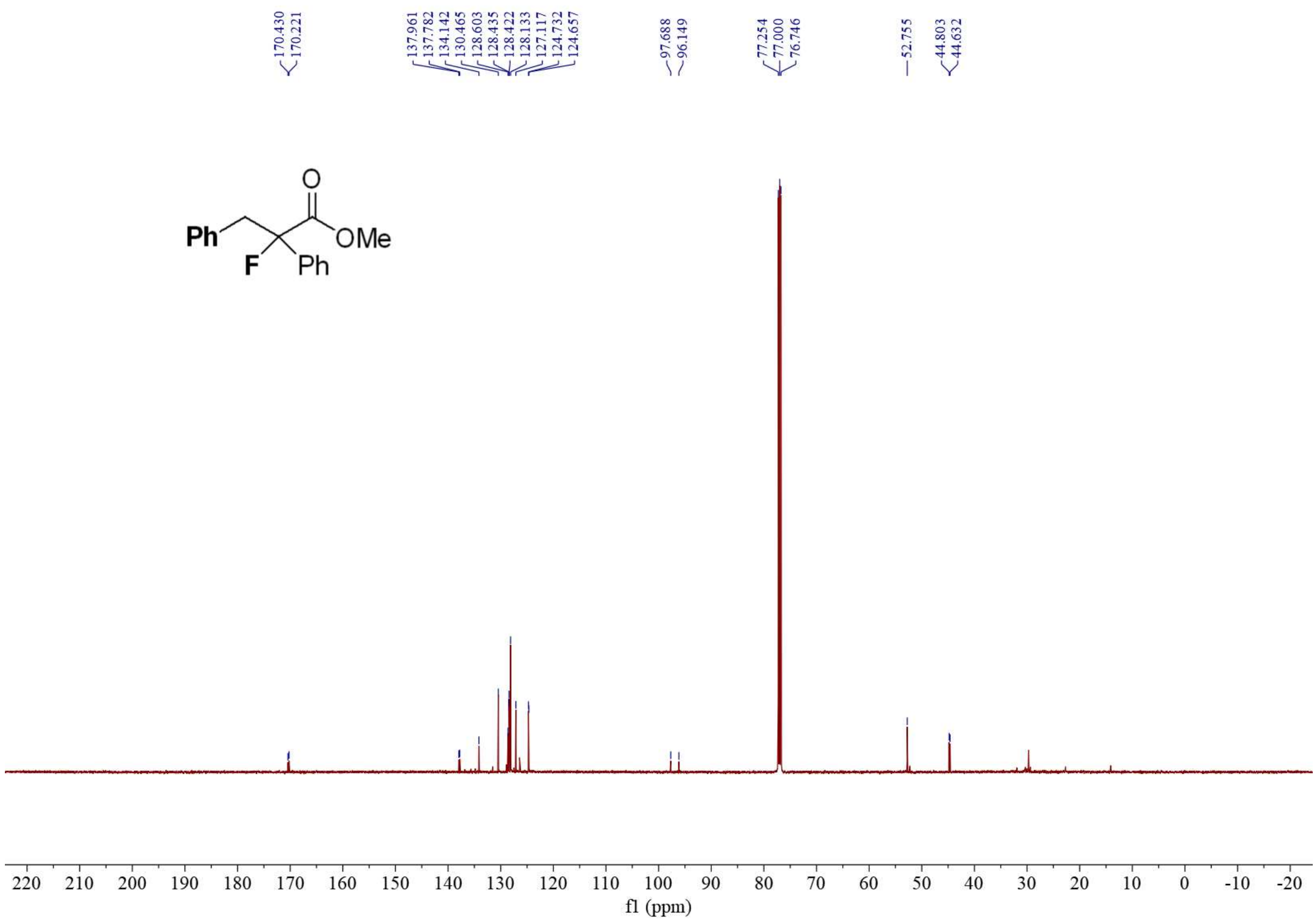

$^{19}F\{^1H\}$ NMR spectrum of **6v** (377 MHz, $CDCl_3$)

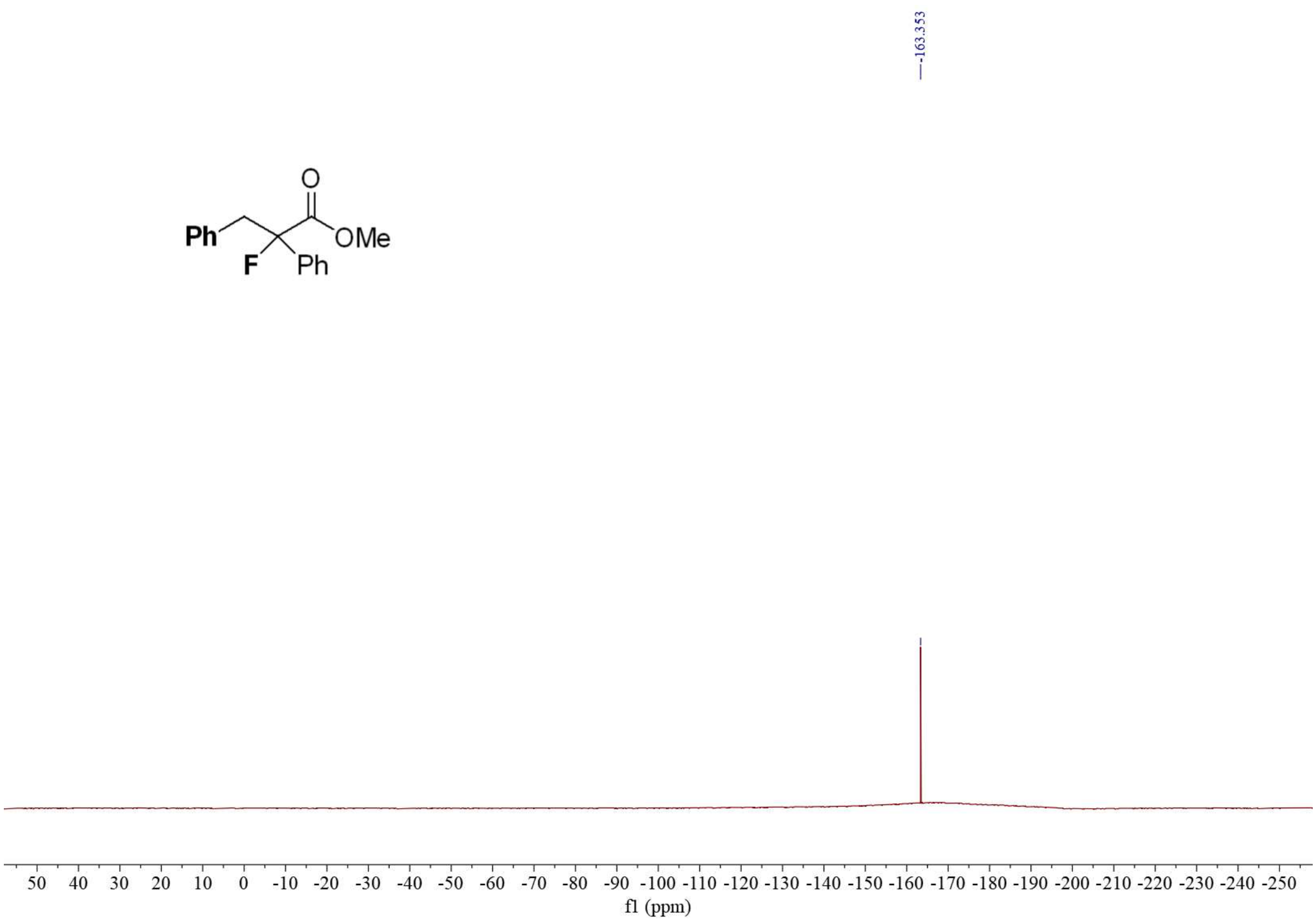

^{19}F NMR spectrum of **6v** (377 MHz, $CDCl_3$)

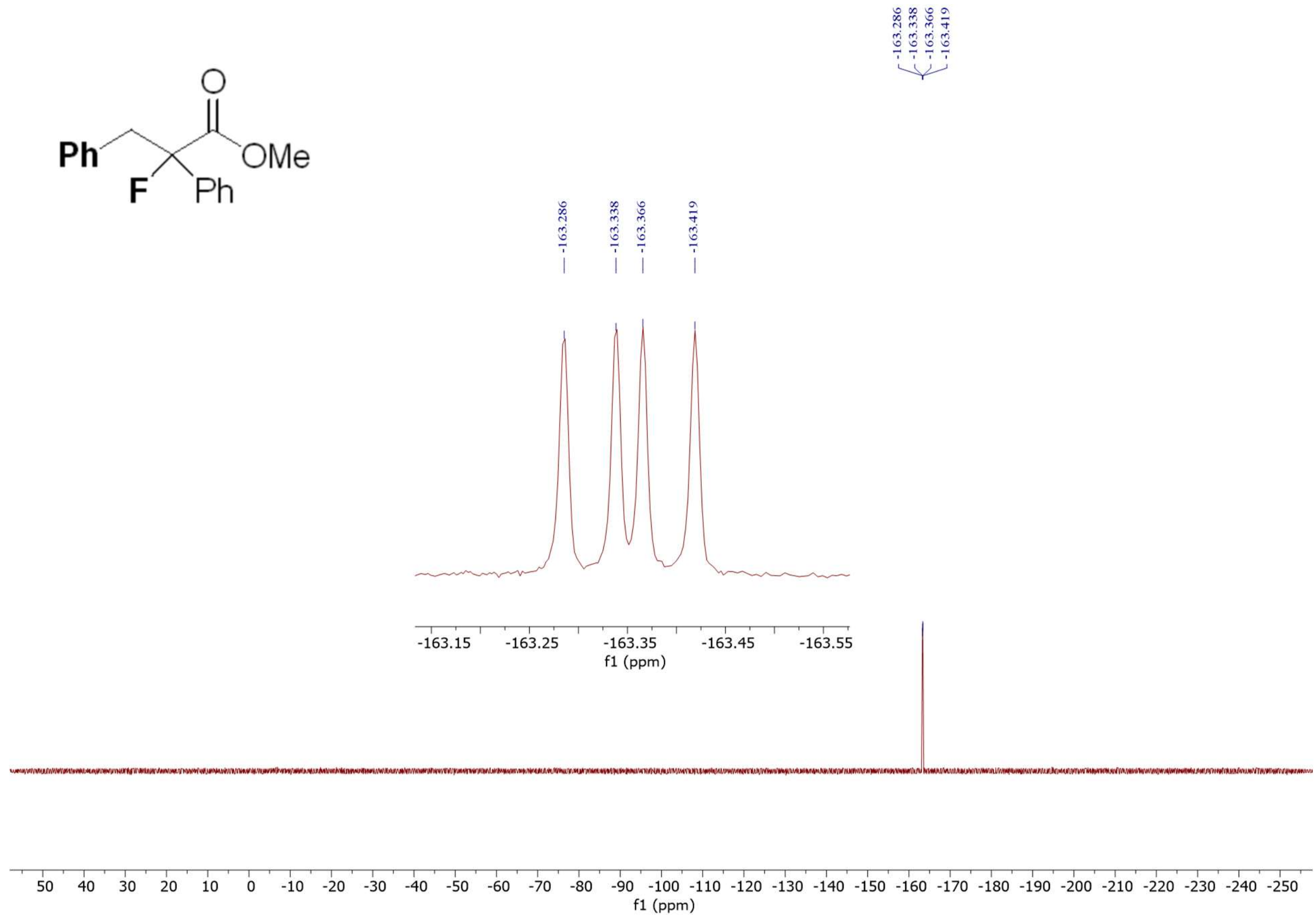

^{1}H NMR spectrum of **6w** (500 MHz, $CDCl_3$)

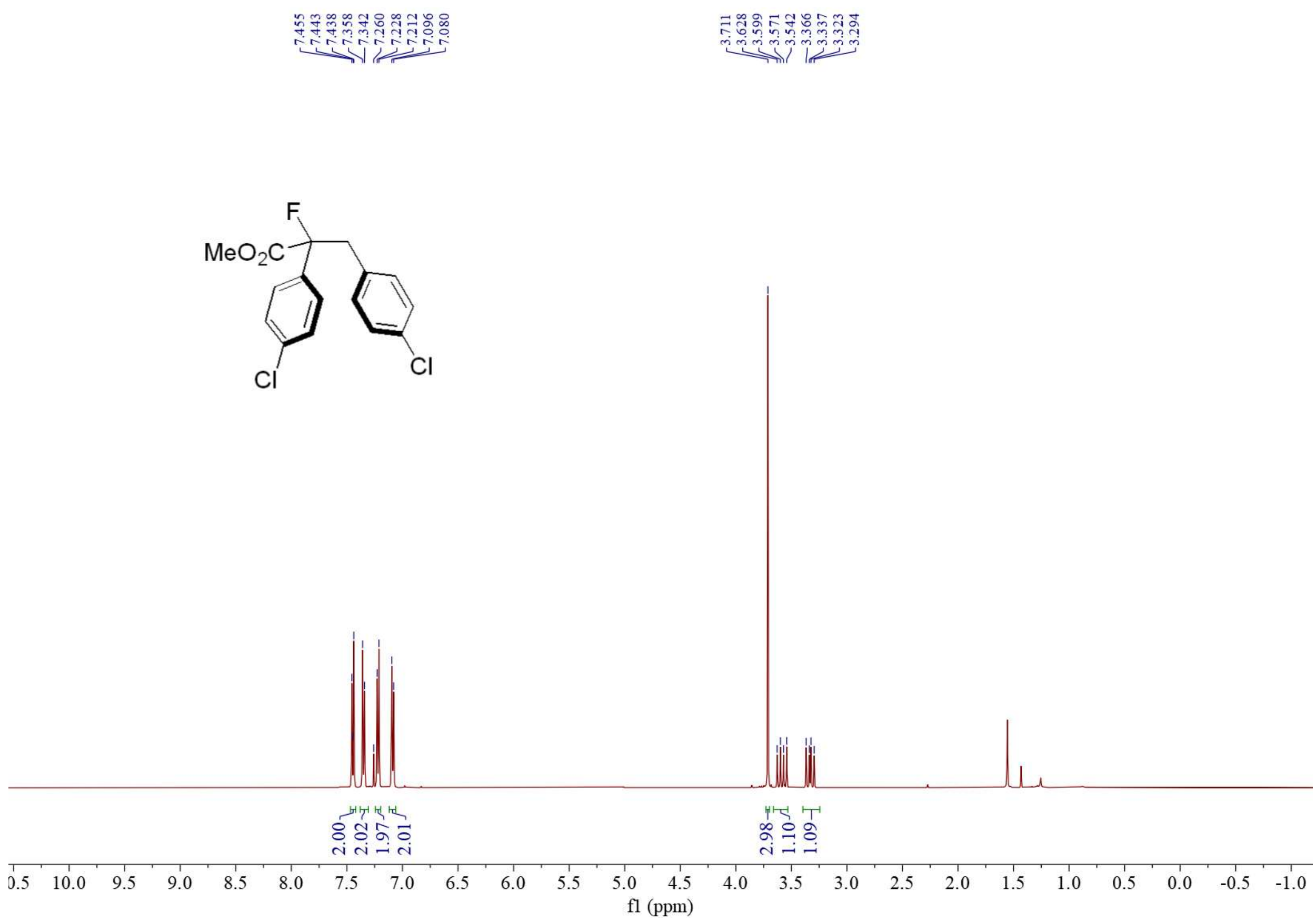

^{13}C NMR spectrum of **6w** (126 MHz, $CDCl_3$)

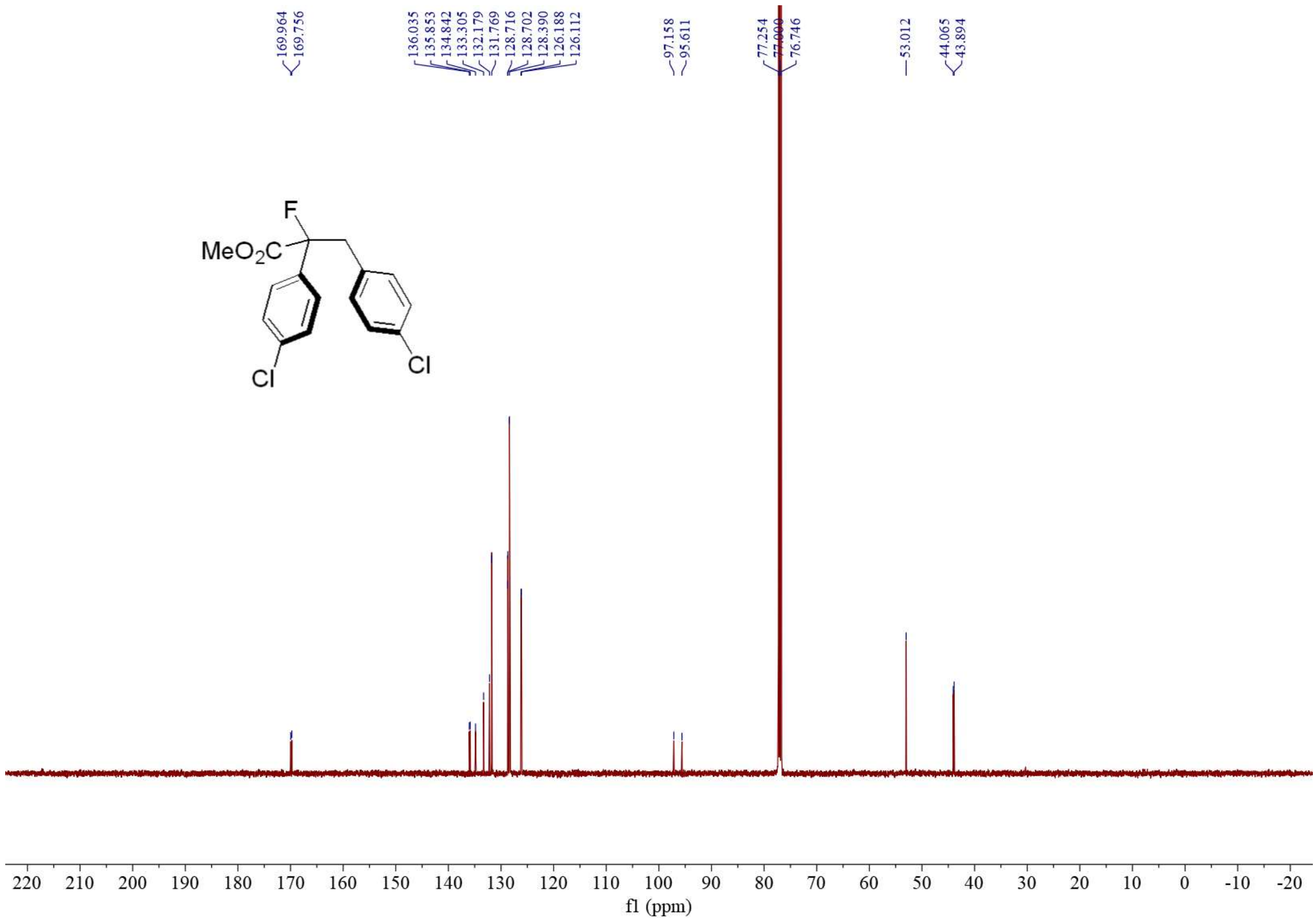

^{19}F{^{1}H} NMR spectrum of **6w** (377 MHz, $CDCl_3$)

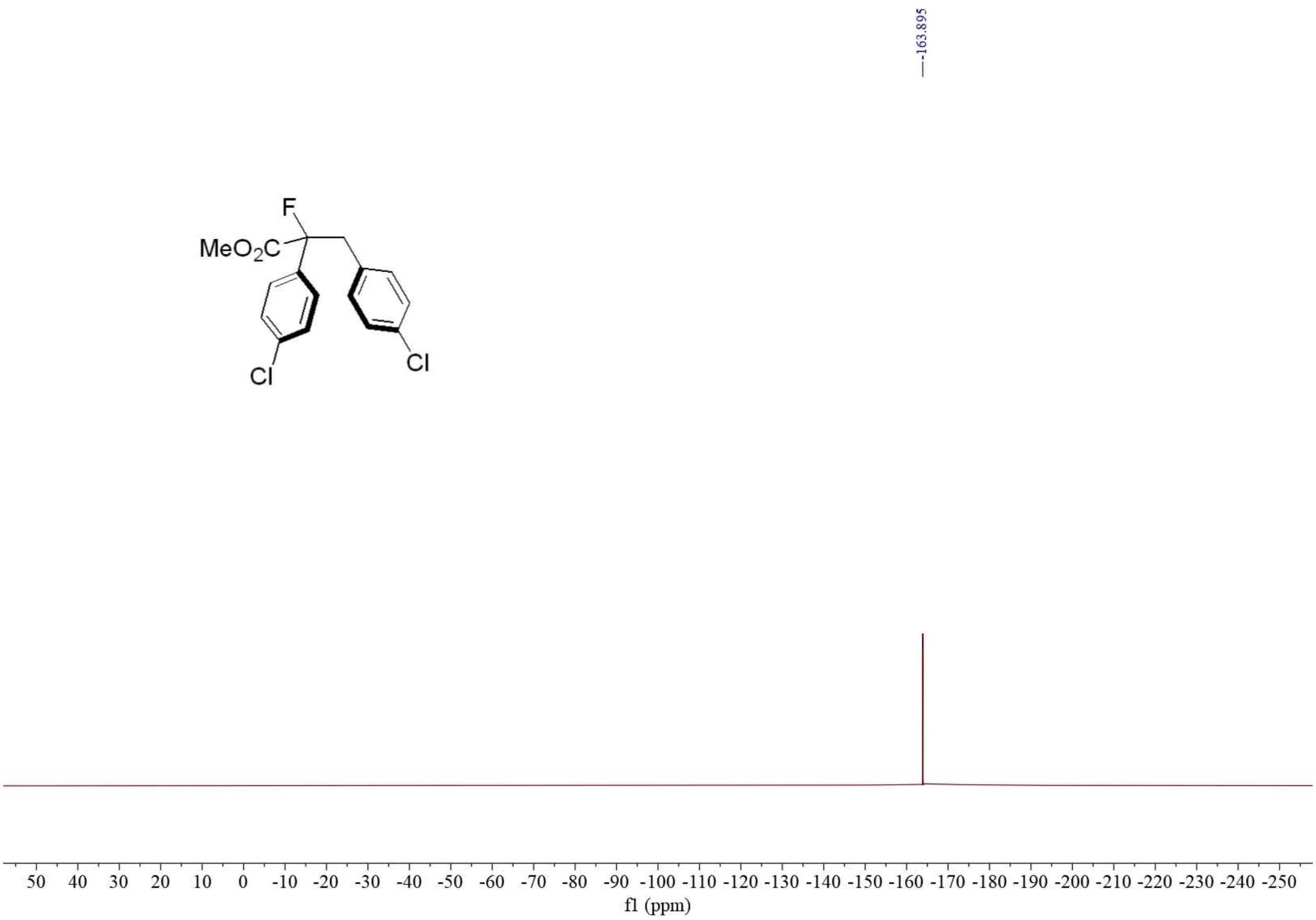

^{19}F NMR spectrum of **6w** (377 MHz, $CDCl_3$)

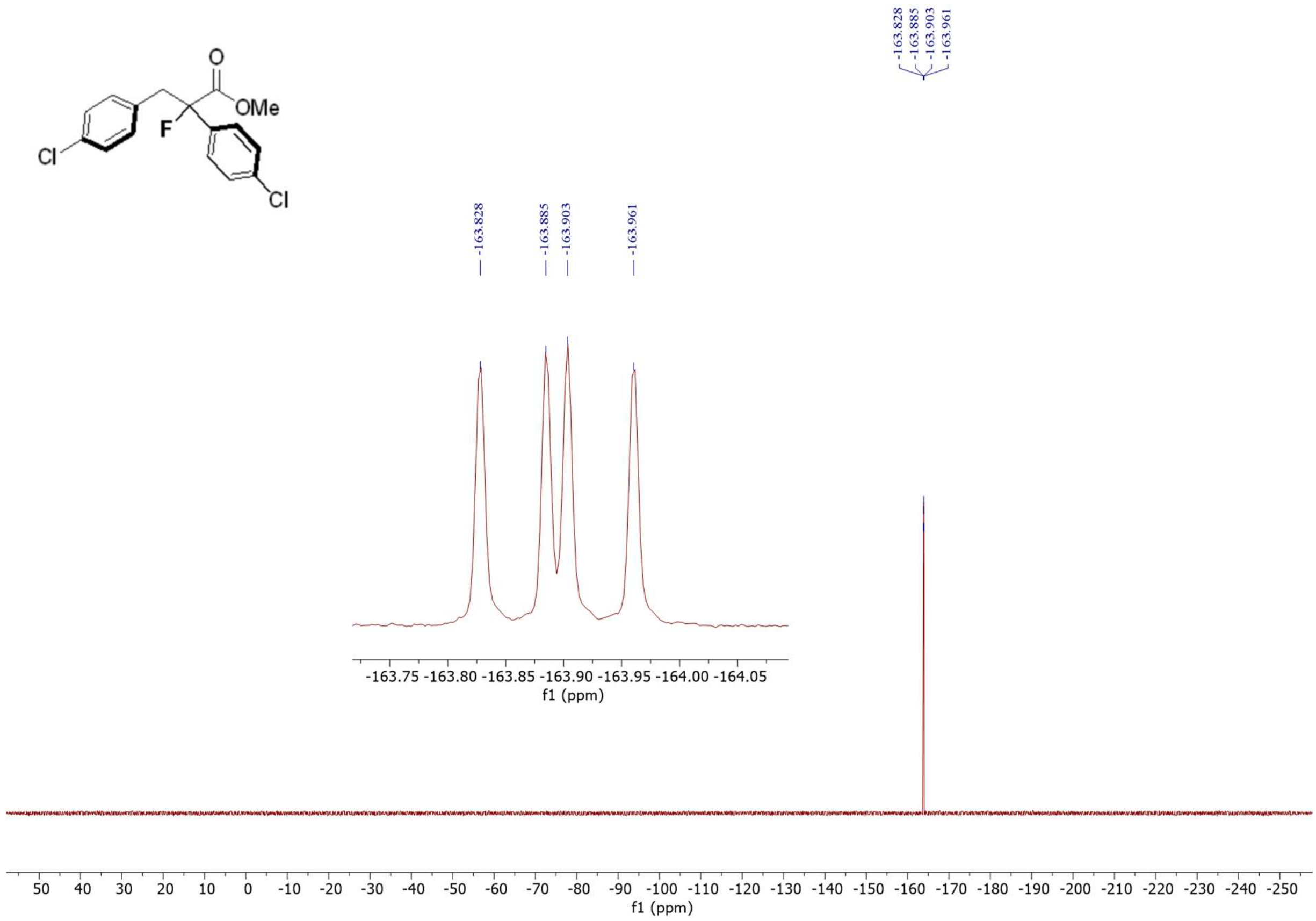

^{1}H NMR spectrum of **6x** (600 MHz, $CDCl_3$)

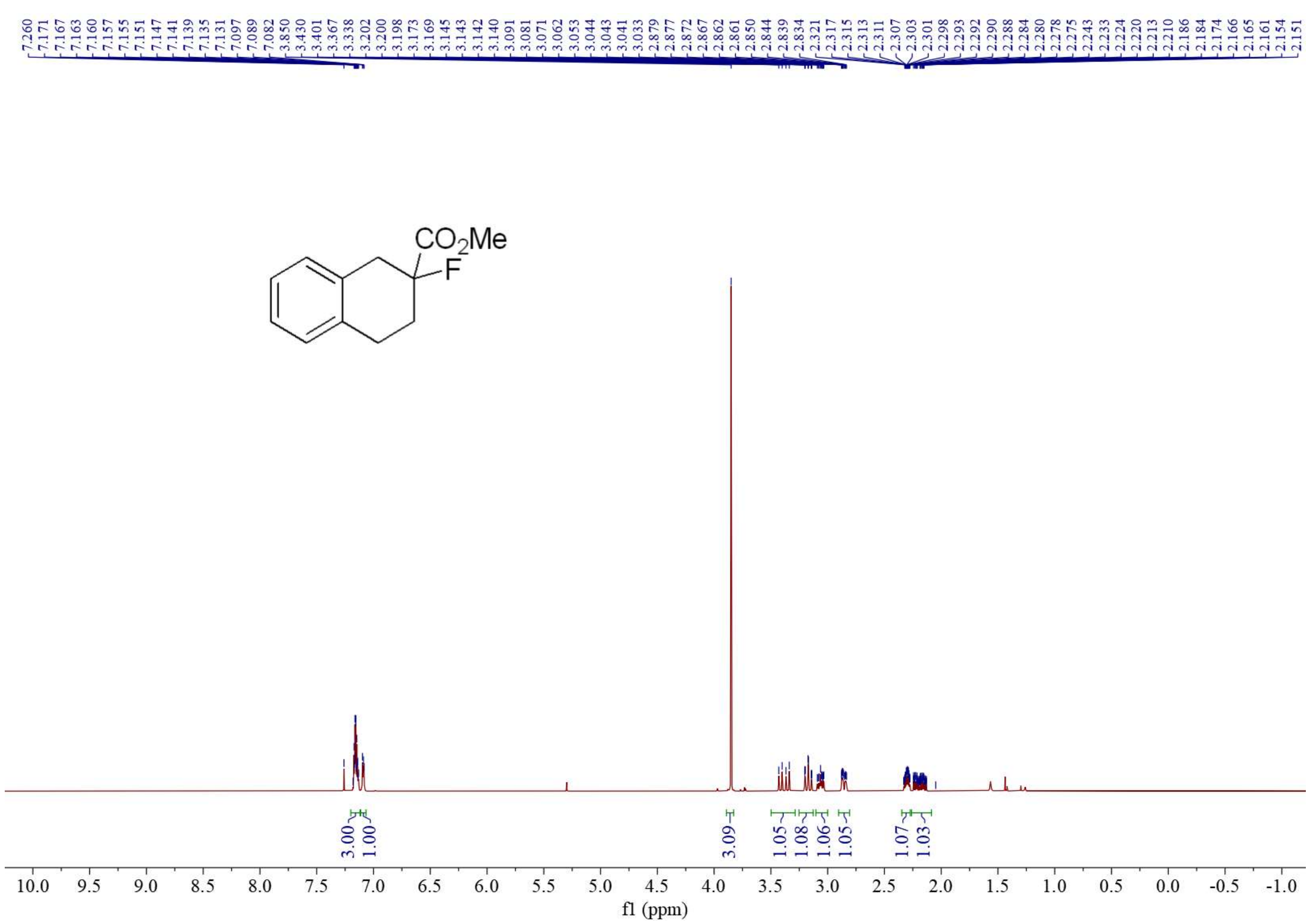

^{13}C NMR spectrum of **6x** (151 MHz, $CDCl_3$)

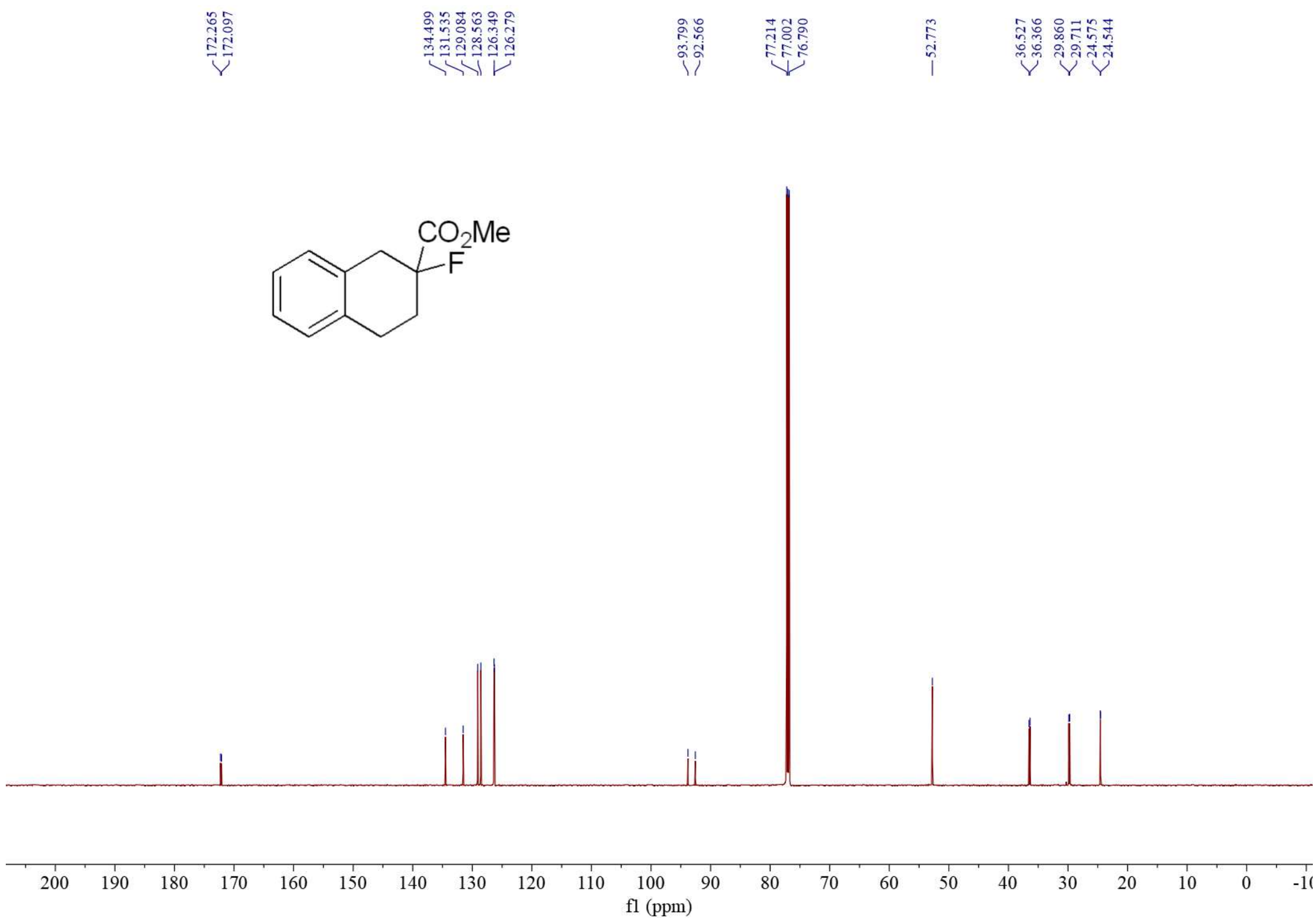

^{19}F{^{1}H} NMR spectrum of **6x** (377 MHz, $CDCl_3$)

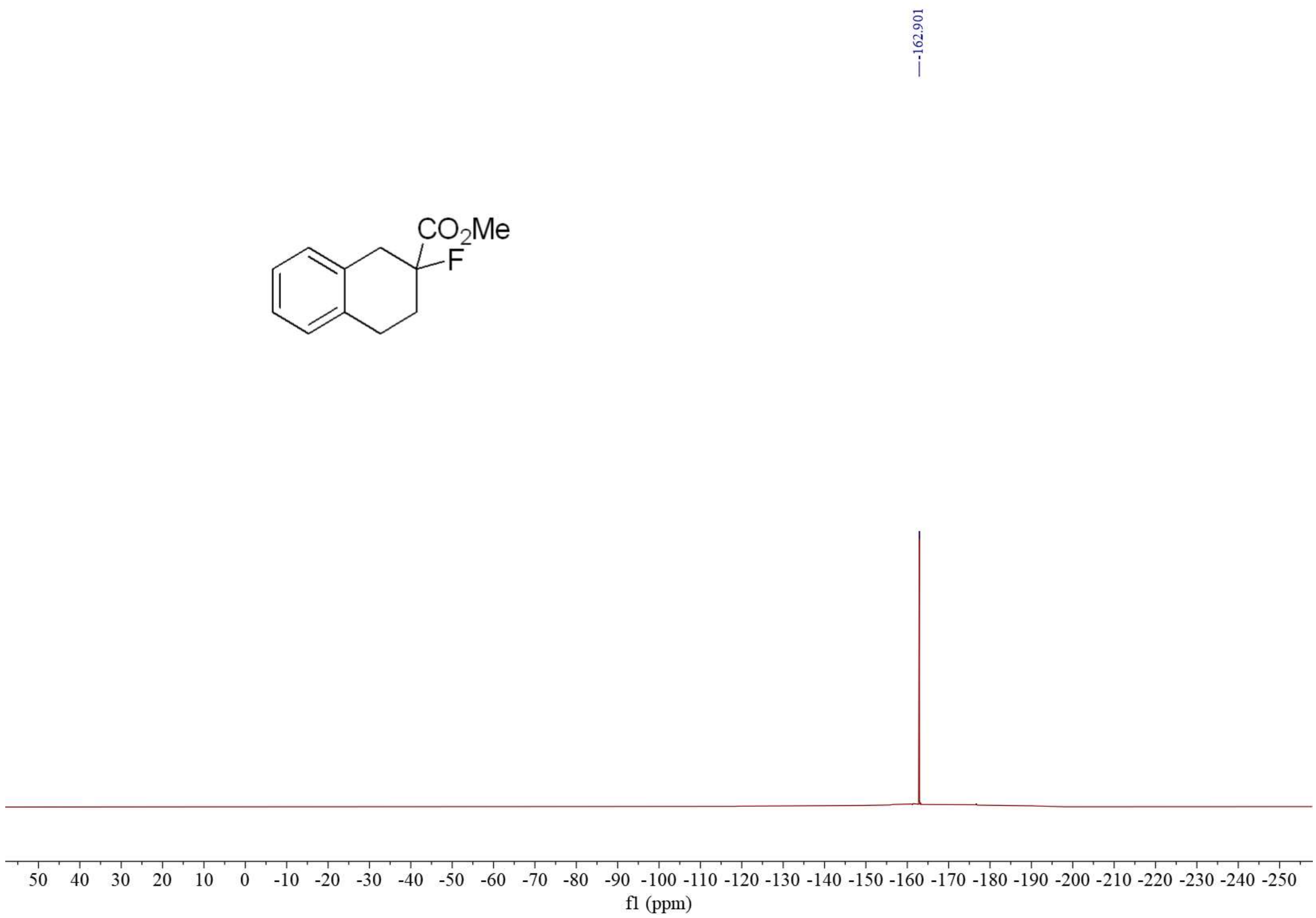

^{19}F NMR spectrum of **6x** (377 MHz, $CDCl_3$)

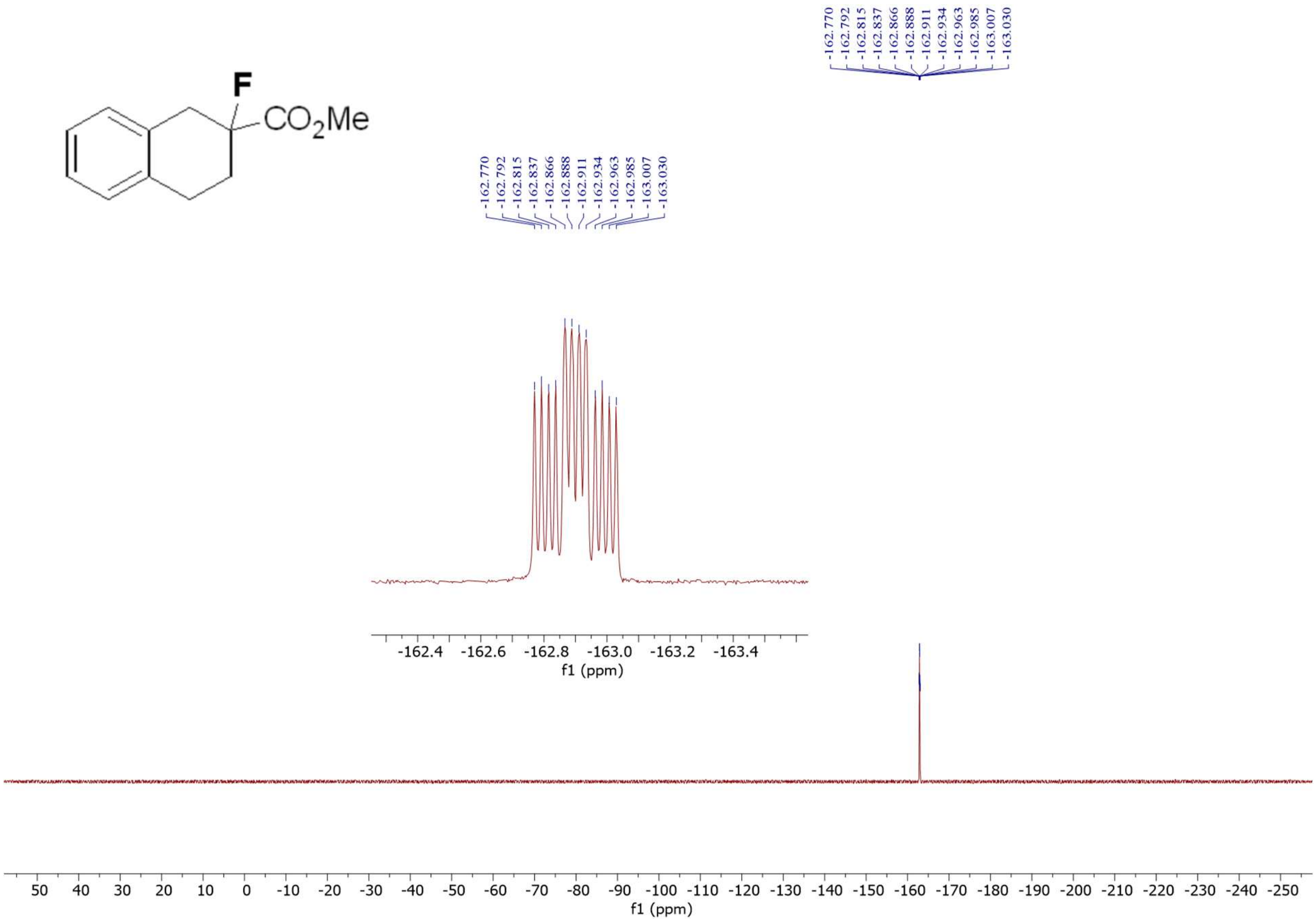

^{1}H NMR spectrum of **6y** (400 MHz, $CDCl_3$)

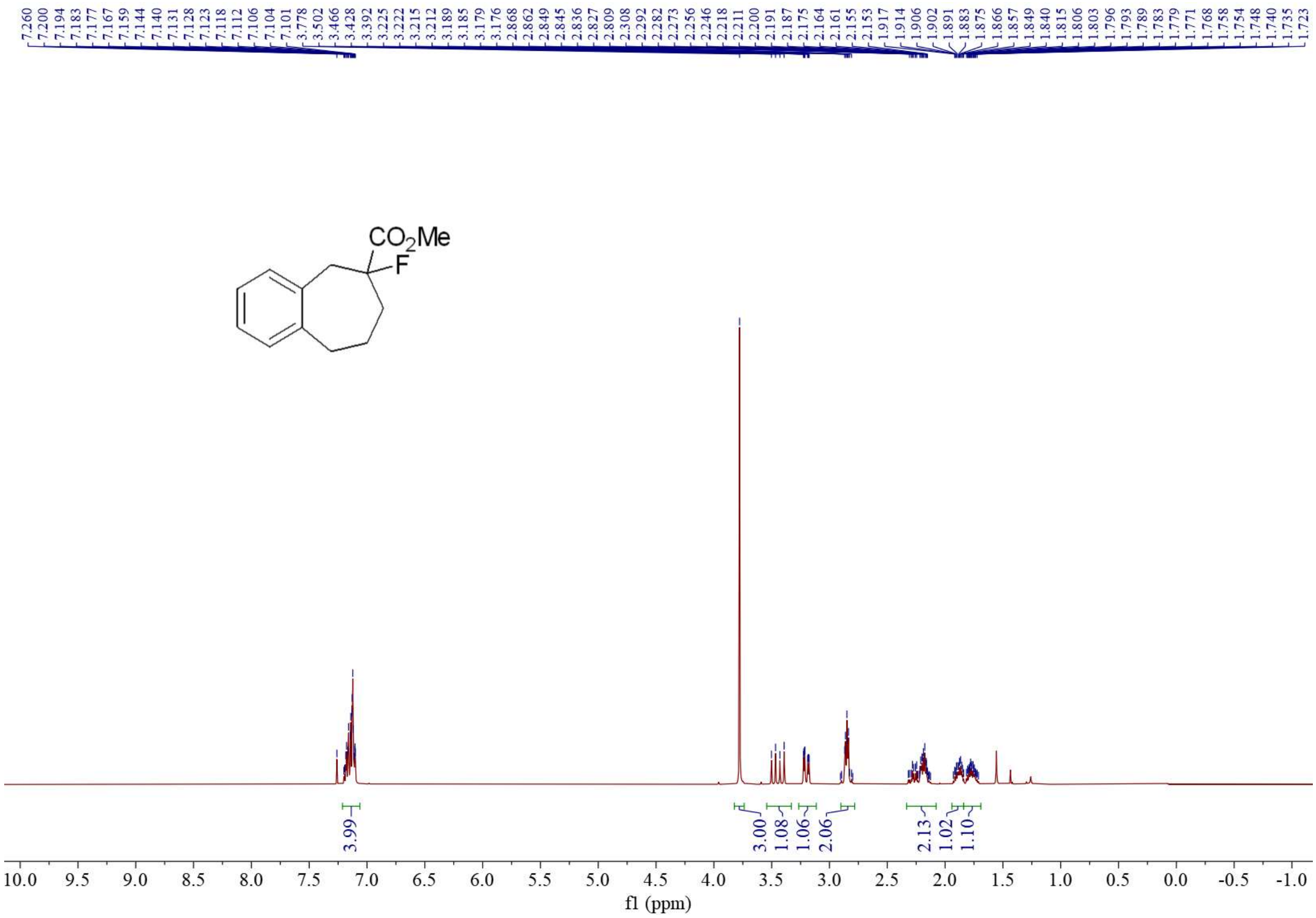

^{13}C NMR spectrum of **6y** (101 MHz, $CDCl_3$)

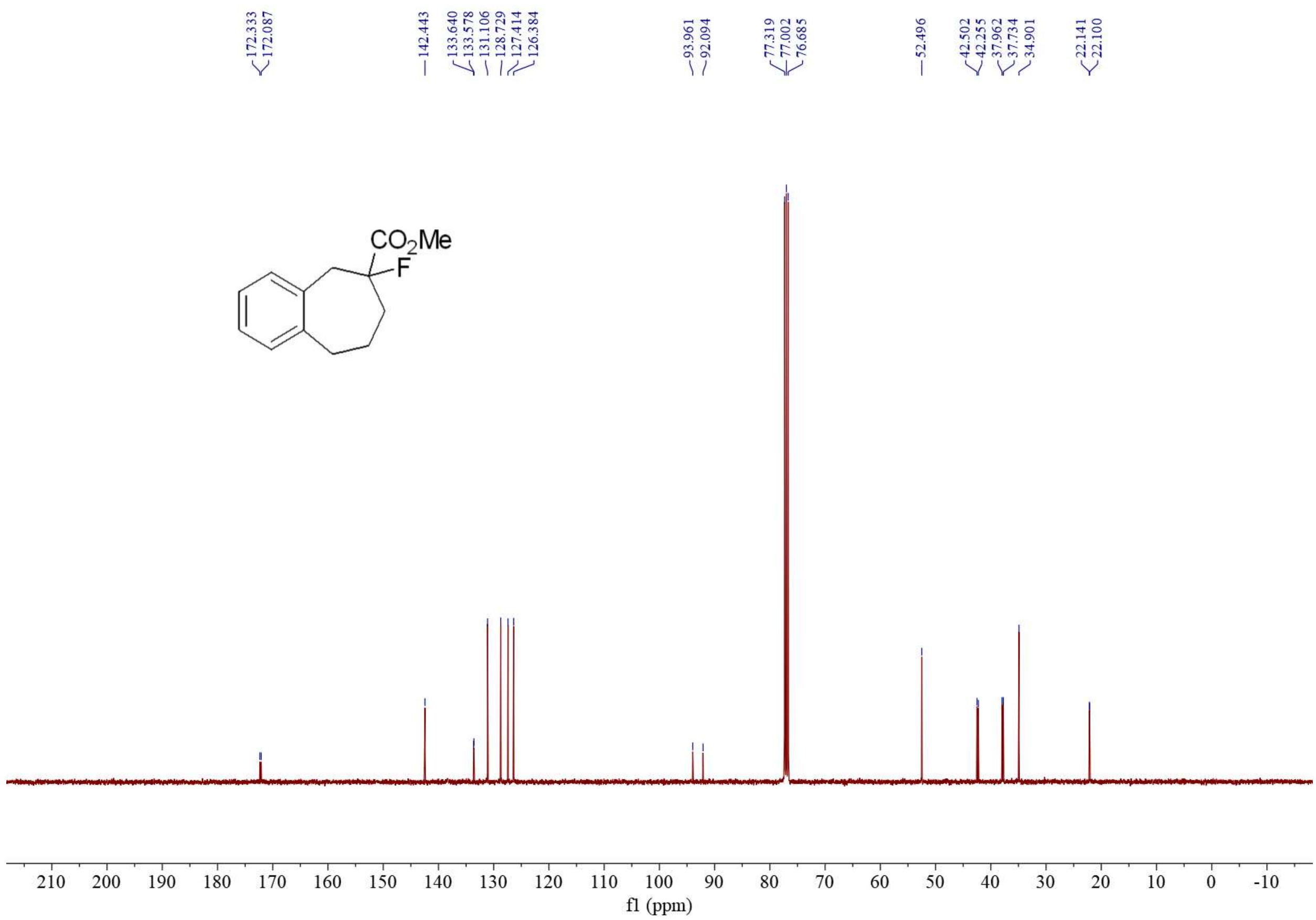

^{19}F{^{1}H} NMR spectrum of **6y** (377 MHz, $CDCl_3$)

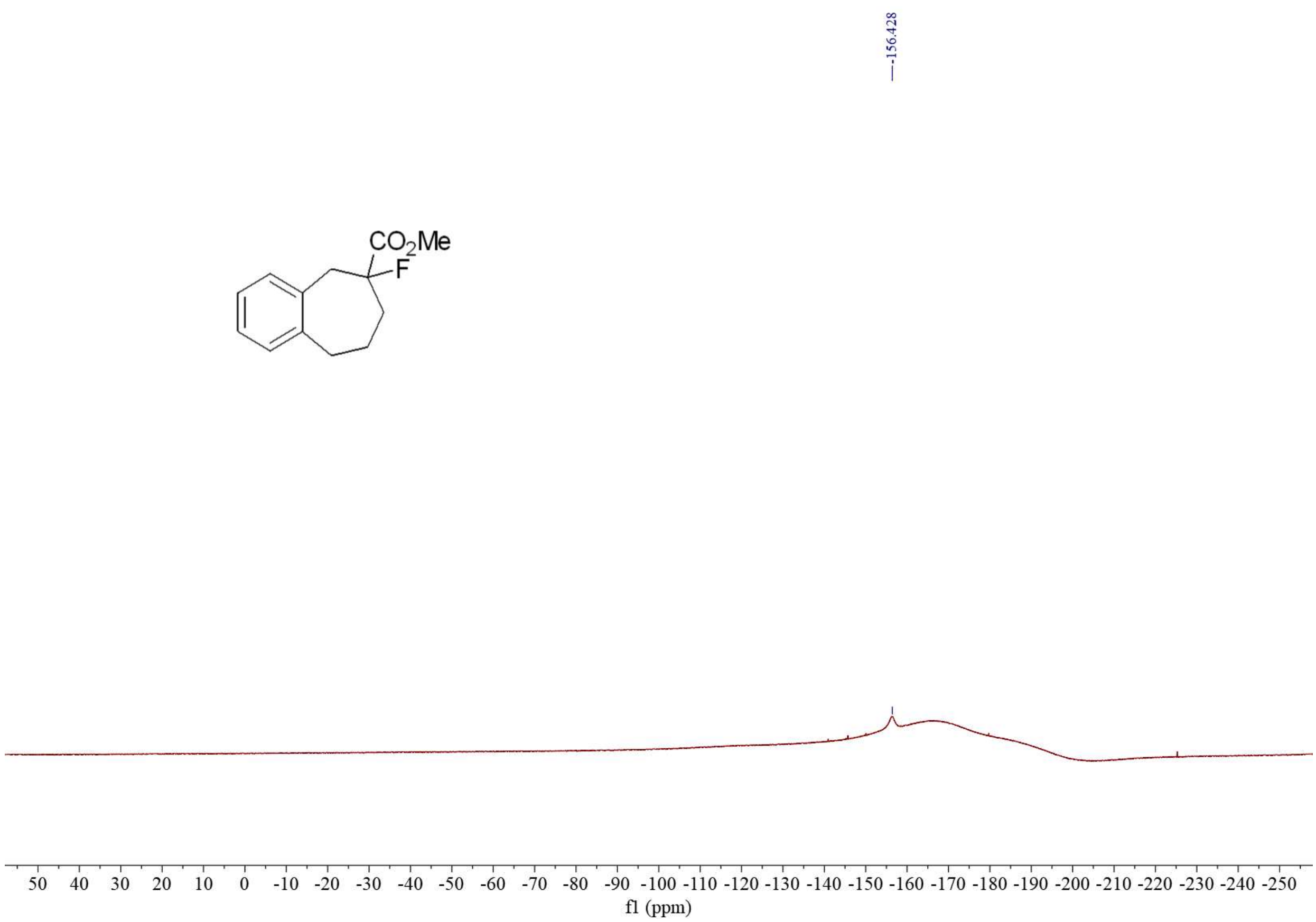

^{19}F NMR spectrum of **6y** (377 MHz, $CDCl_3$)

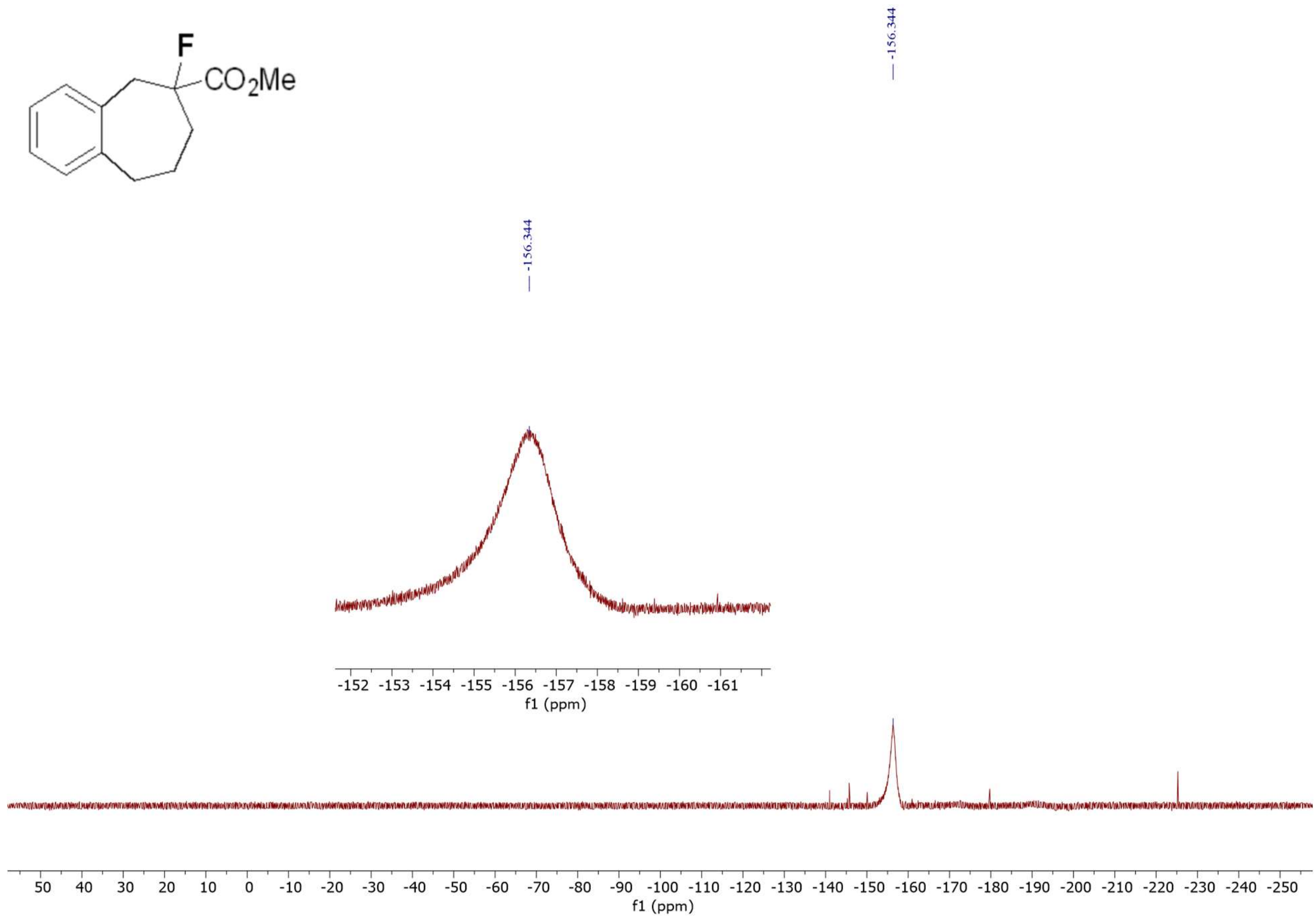

^{1}H NMR spectrum of **6z** (400 MHz, $CDCl_3$)

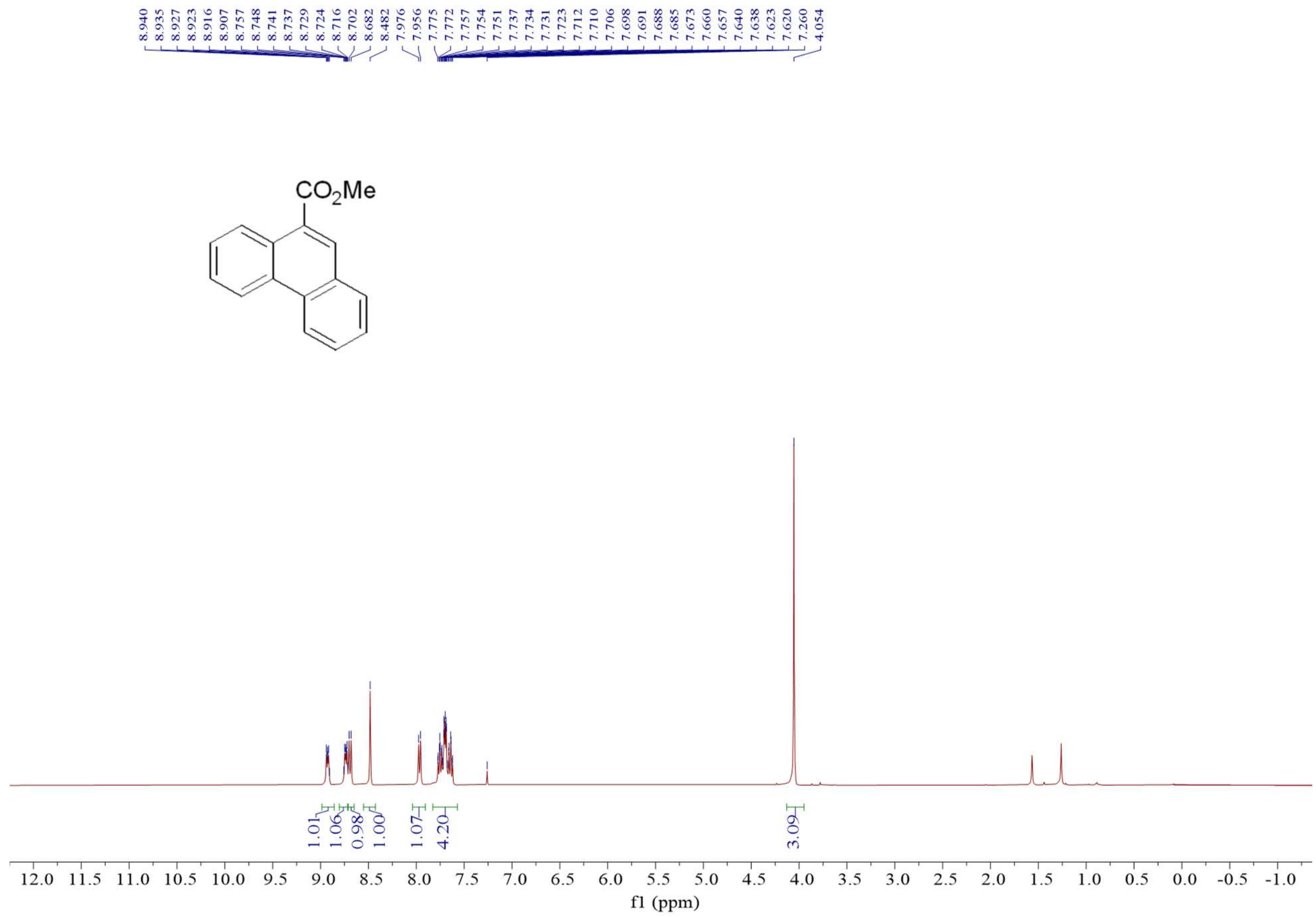

^{13}C NMR spectrum of **6z** (101 MHz, $CDCl_3$)

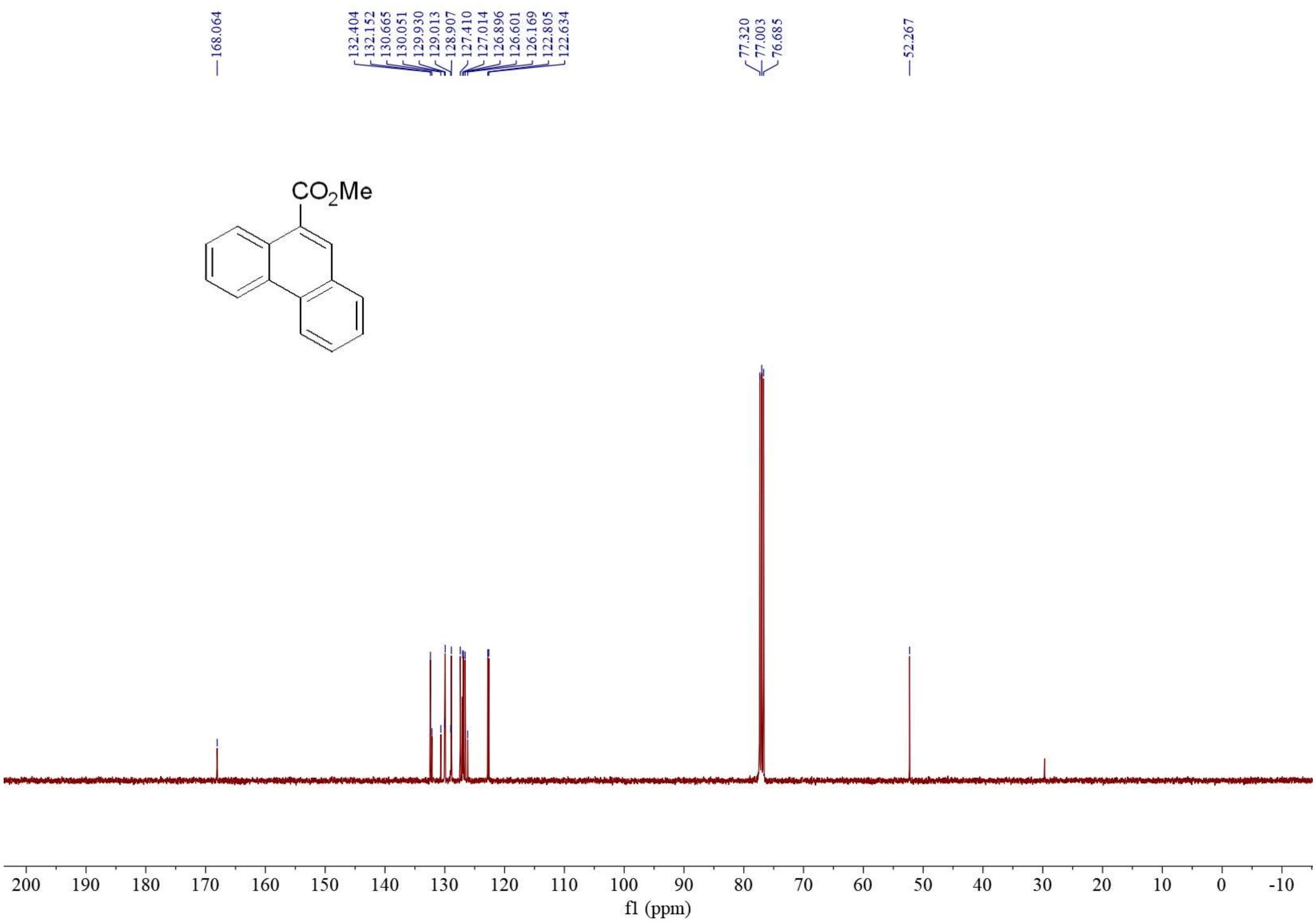

^{1}H NMR spectrum of **6aa** (500 MHz, $CDCl_3$)

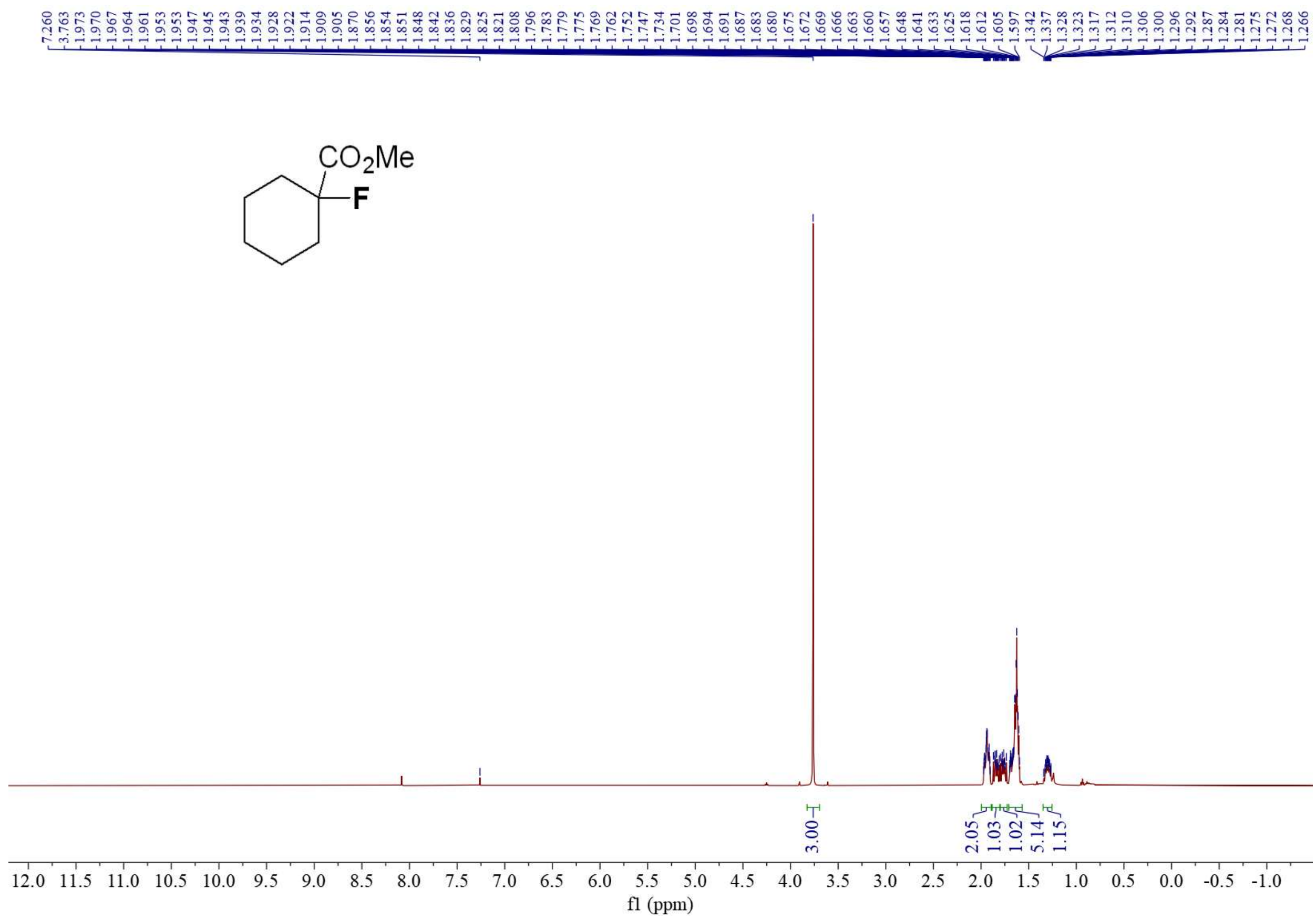

^{13}C NMR spectrum of **6aa** (101 MHz, $CDCl_3$)

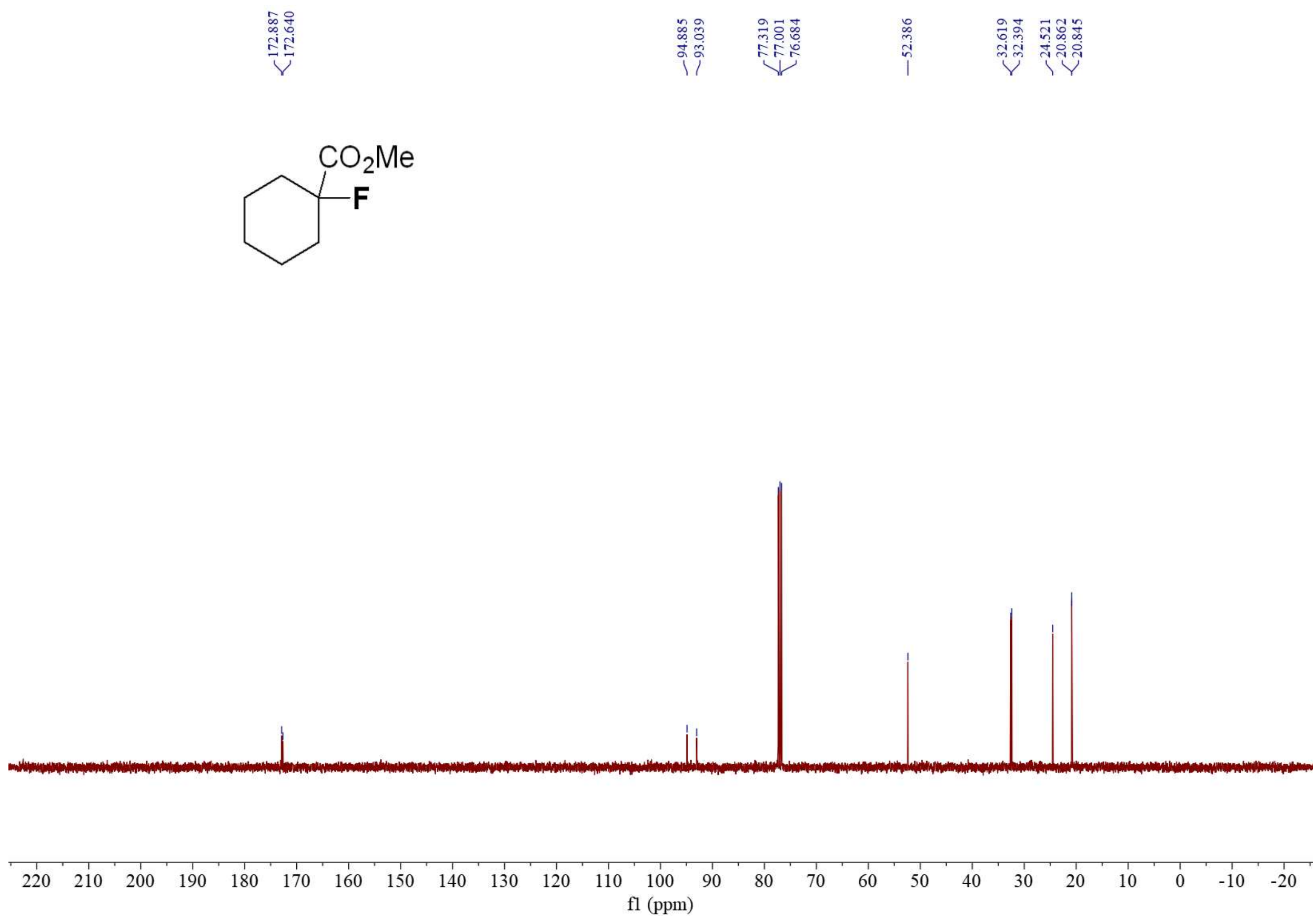

$^{19}F\{^{1}H\}$ NMR spectrum of **6aa** (377 MHz, $CDCl_3$)

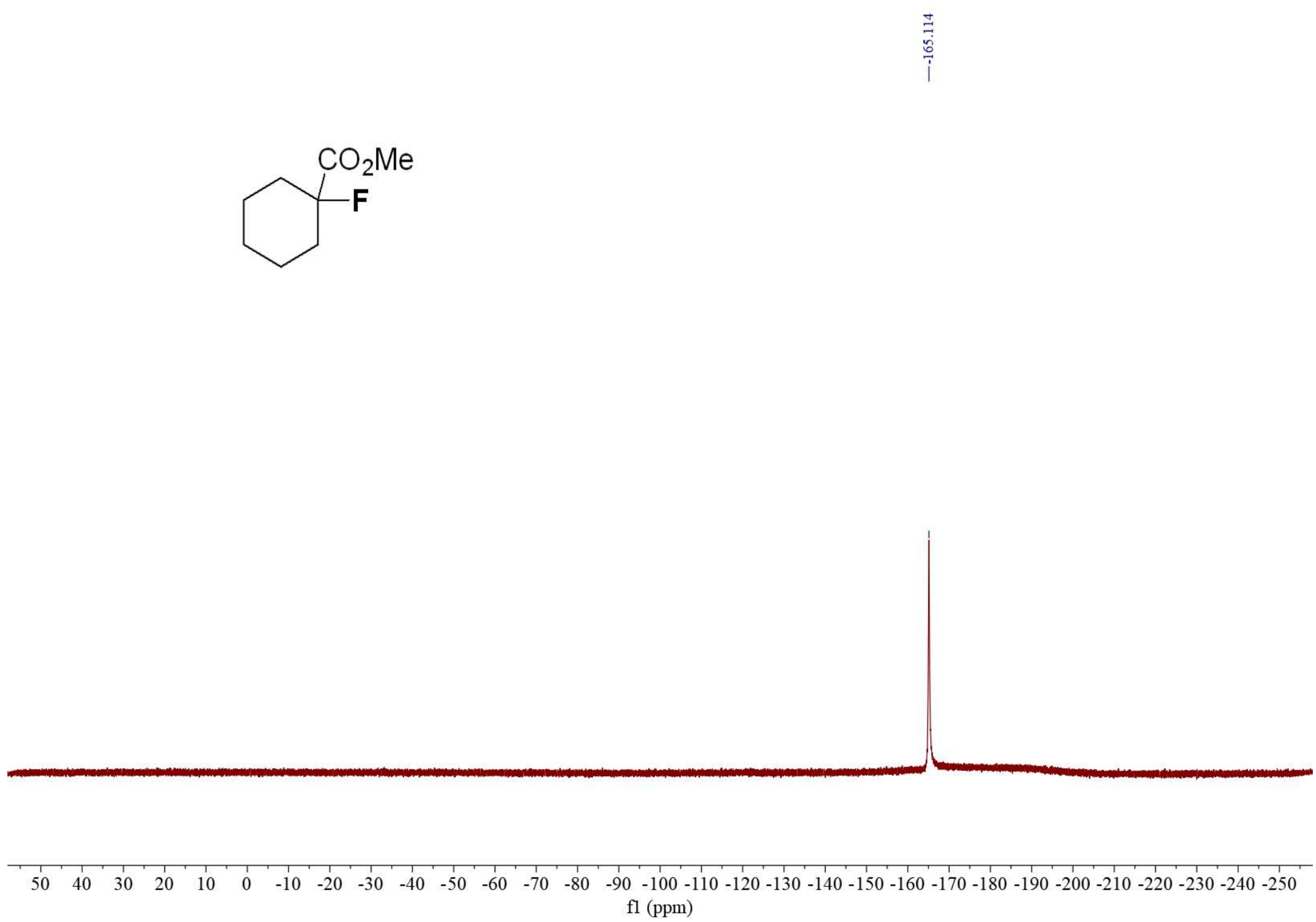

^{19}F NMR spectrum of **6aa** (377 MHz, $CDCl_3$)

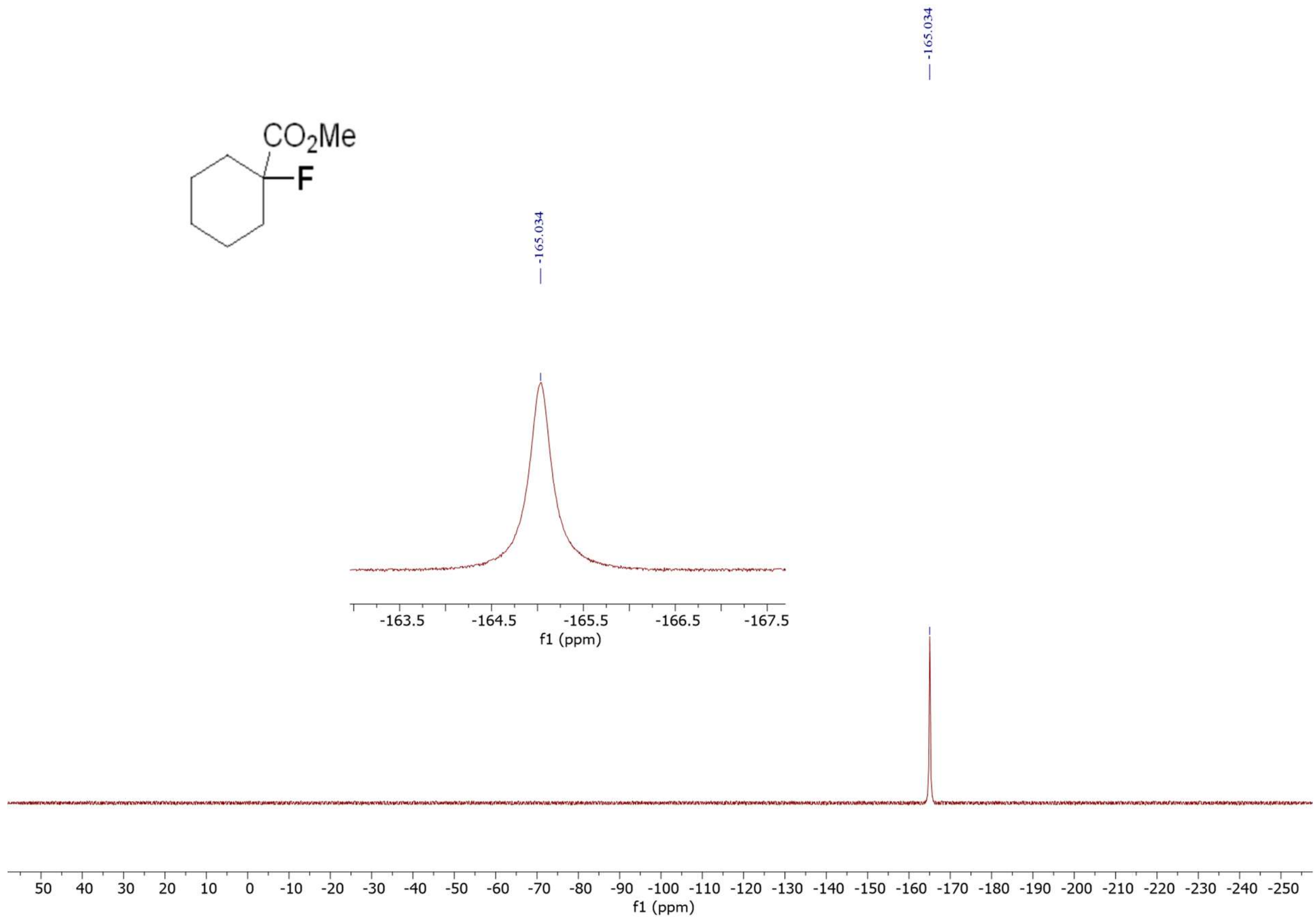

^{1}H NMR spectrum of **8a** (500 MHz, $CDCl_3$)

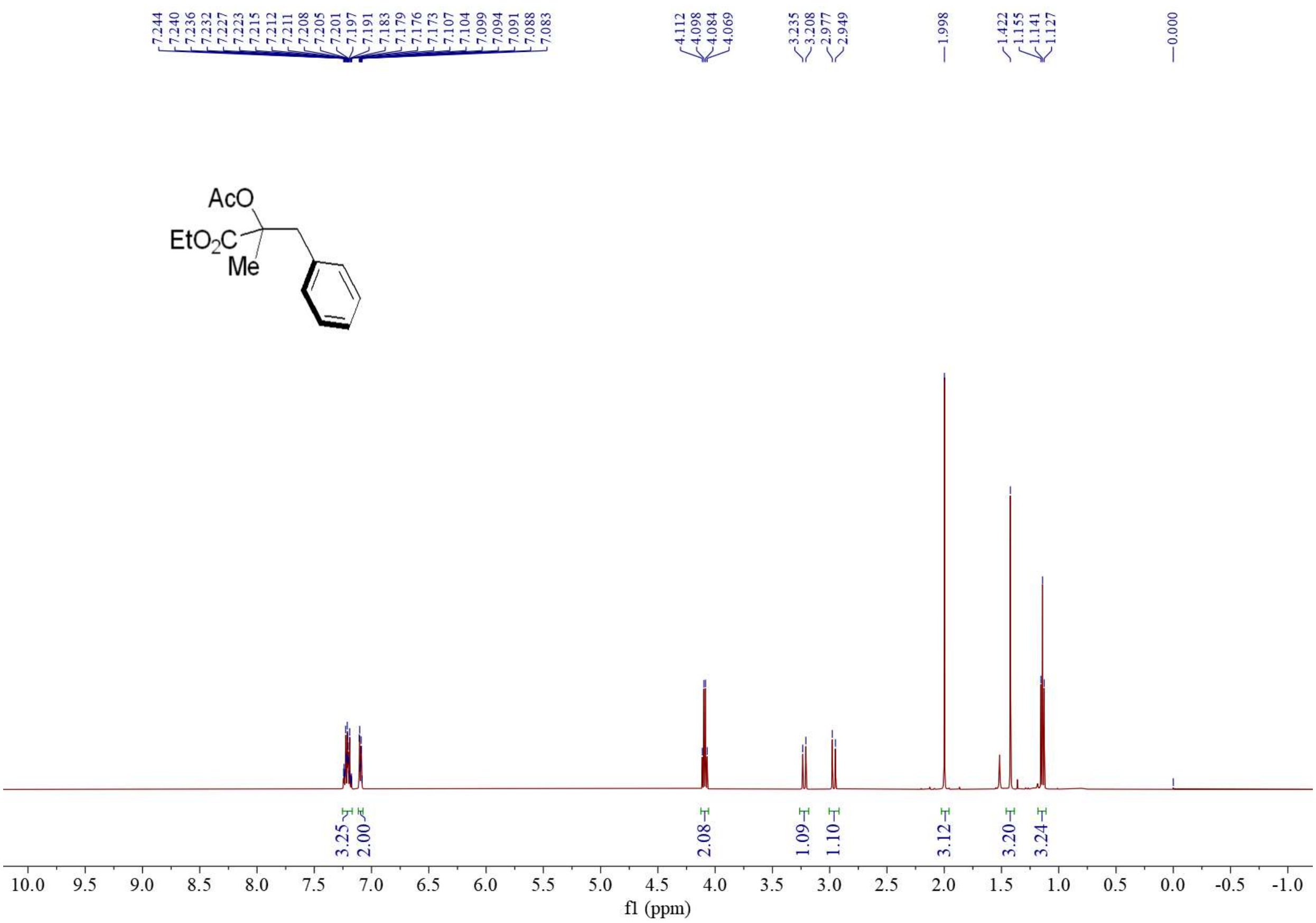

^{13}C NMR spectrum of **8a** (126 MHz, $CDCl_3$)

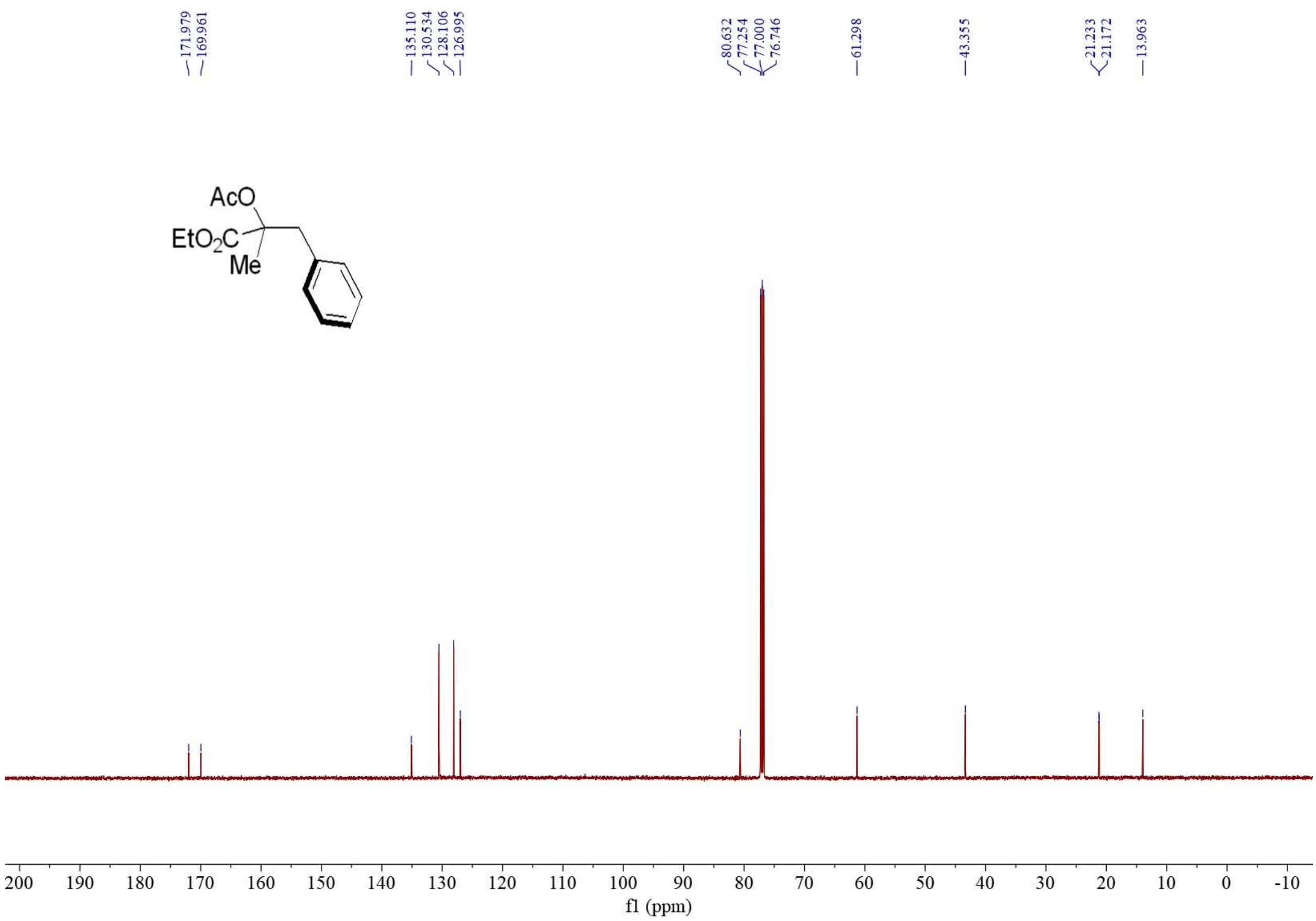

^{1}H NMR spectrum of **8b** (600 MHz, $CDCl_3$)

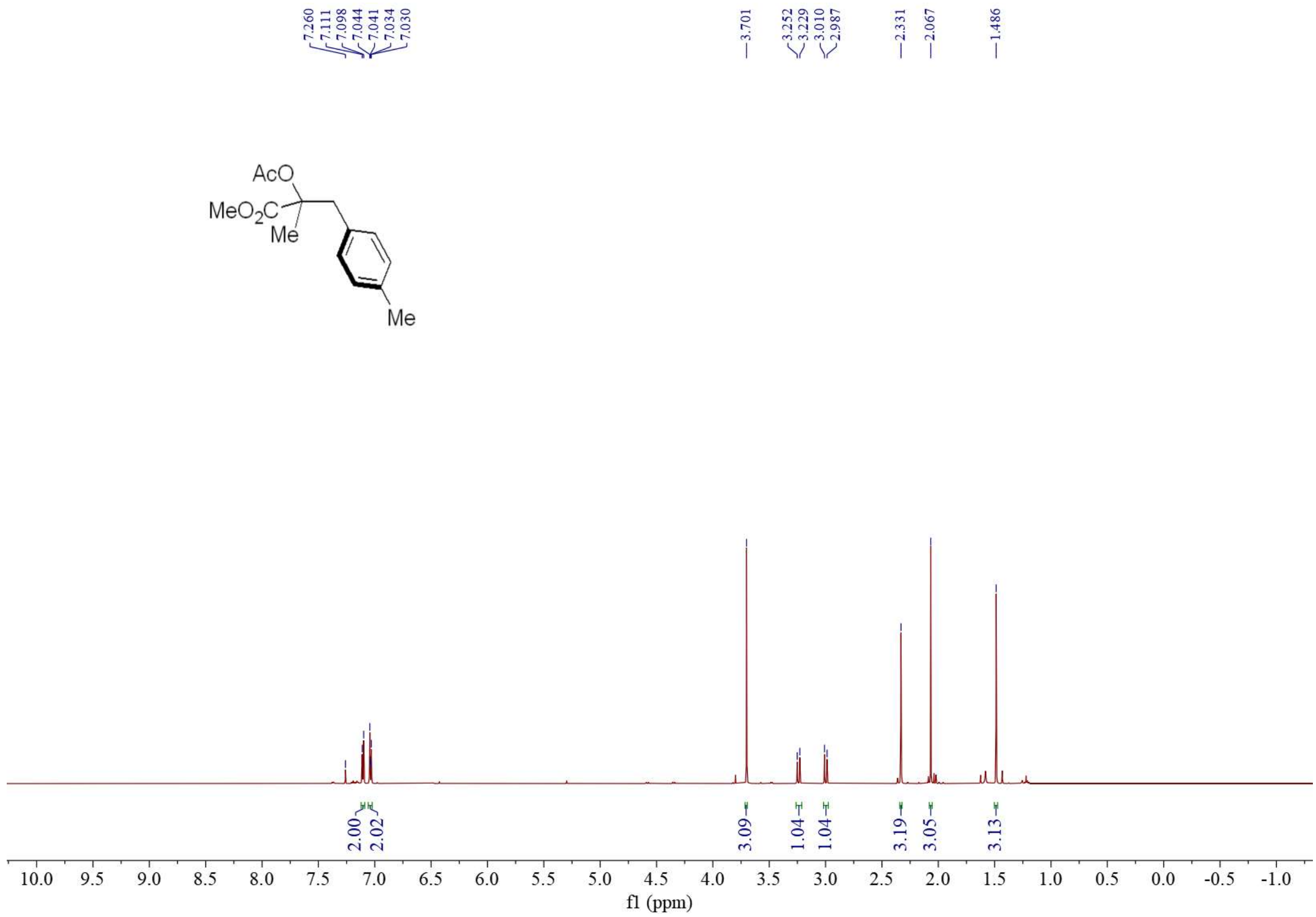

^{13}C NMR spectrum of **8b** (151 MHz, $CDCl_3$)

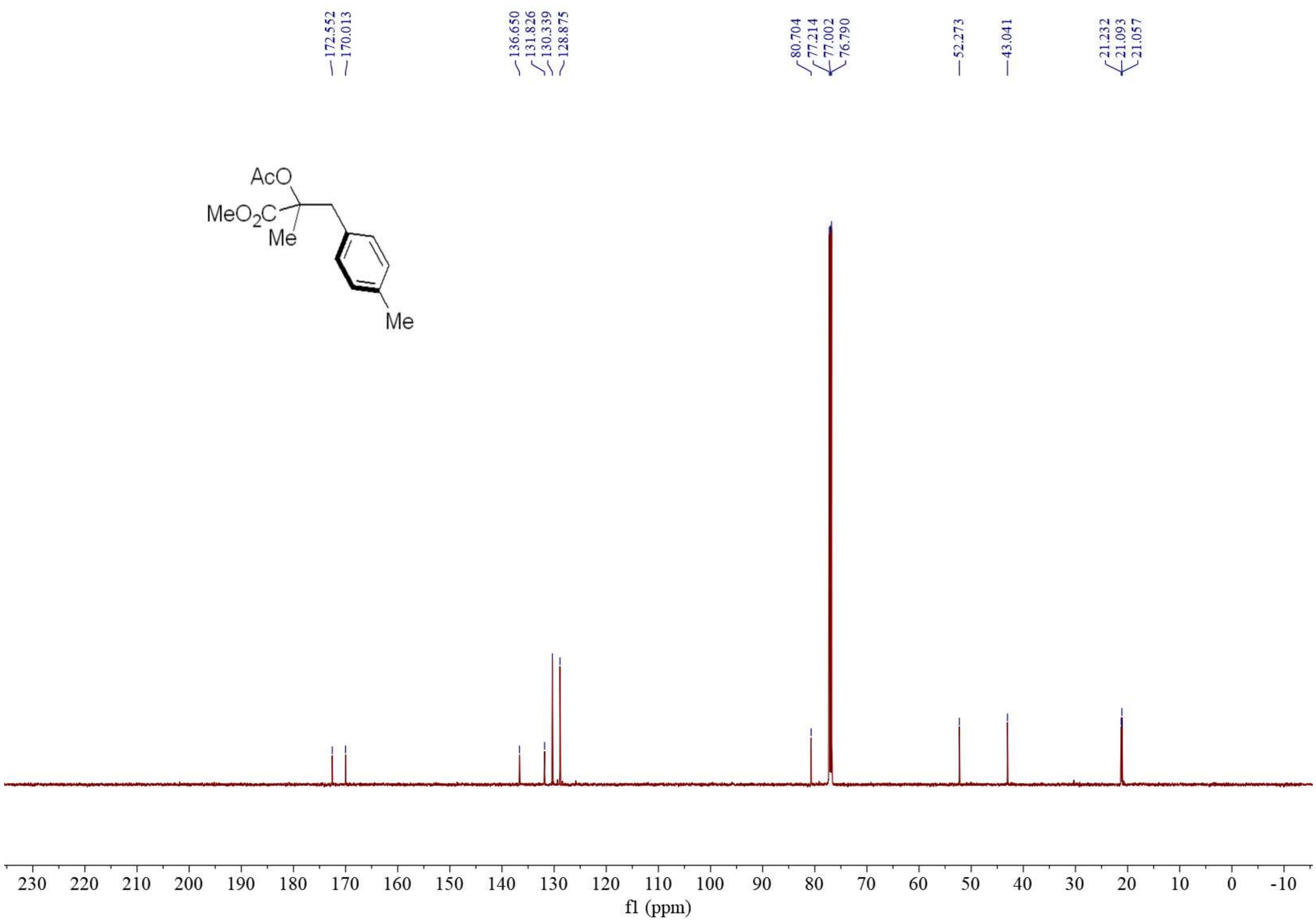

^{1}H NMR spectrum of **8c** (500 MHz, $CDCl_3$)

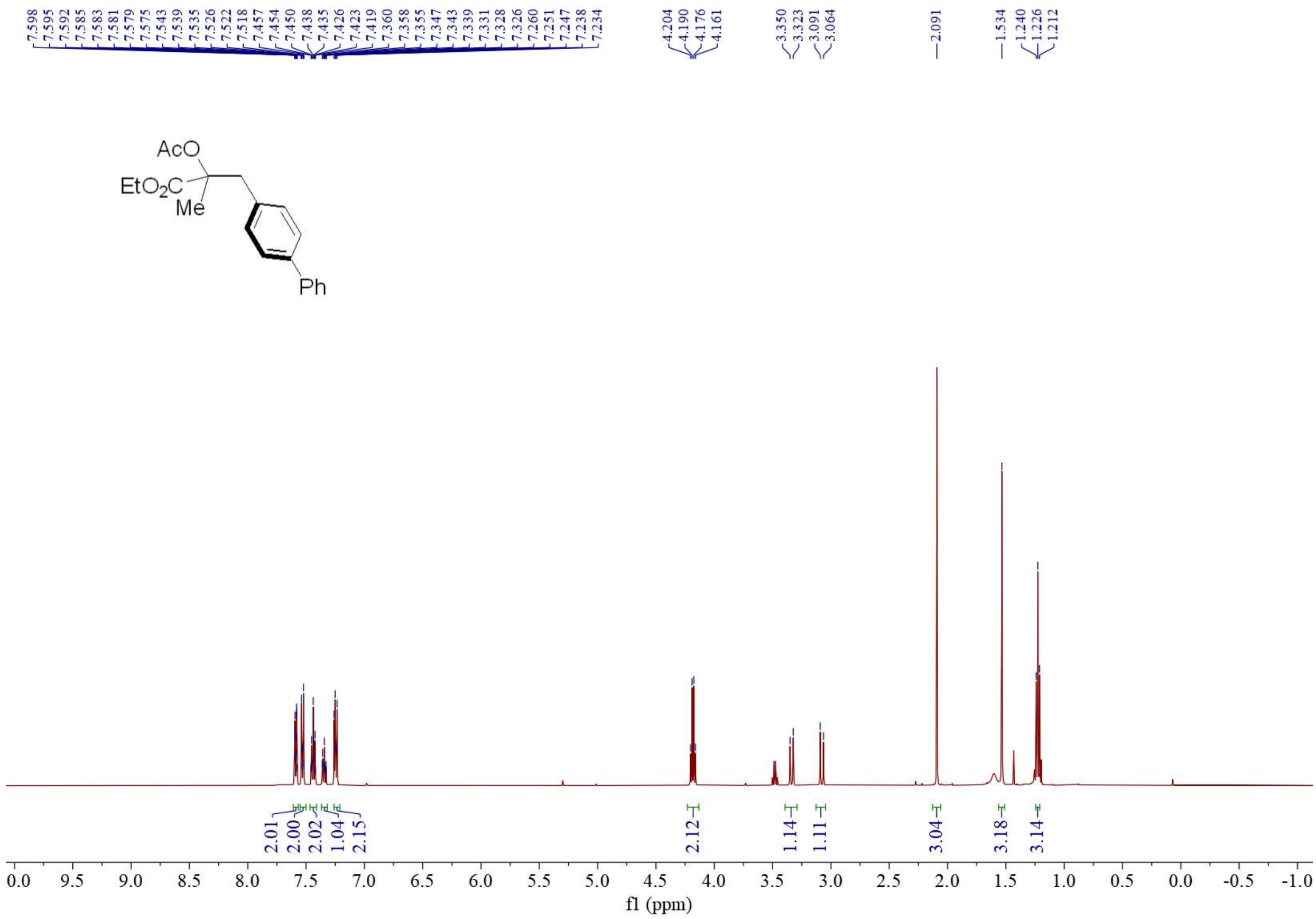

^{13}C NMR spectrum of **8c** (126 MHz, $CDCl_3$)

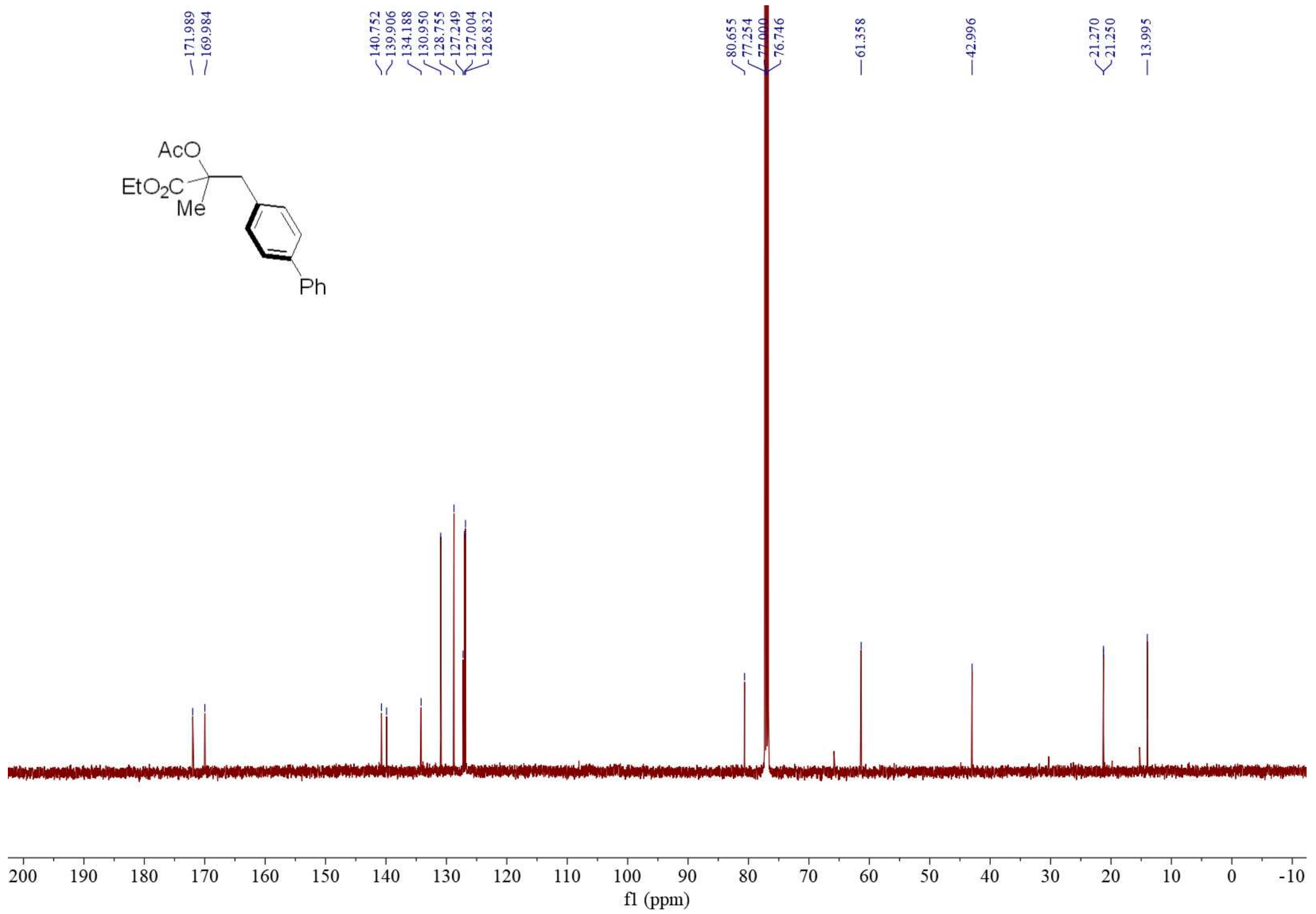

^{1}H NMR spectrum of **8d** (600 MHz, $CDCl_3$)

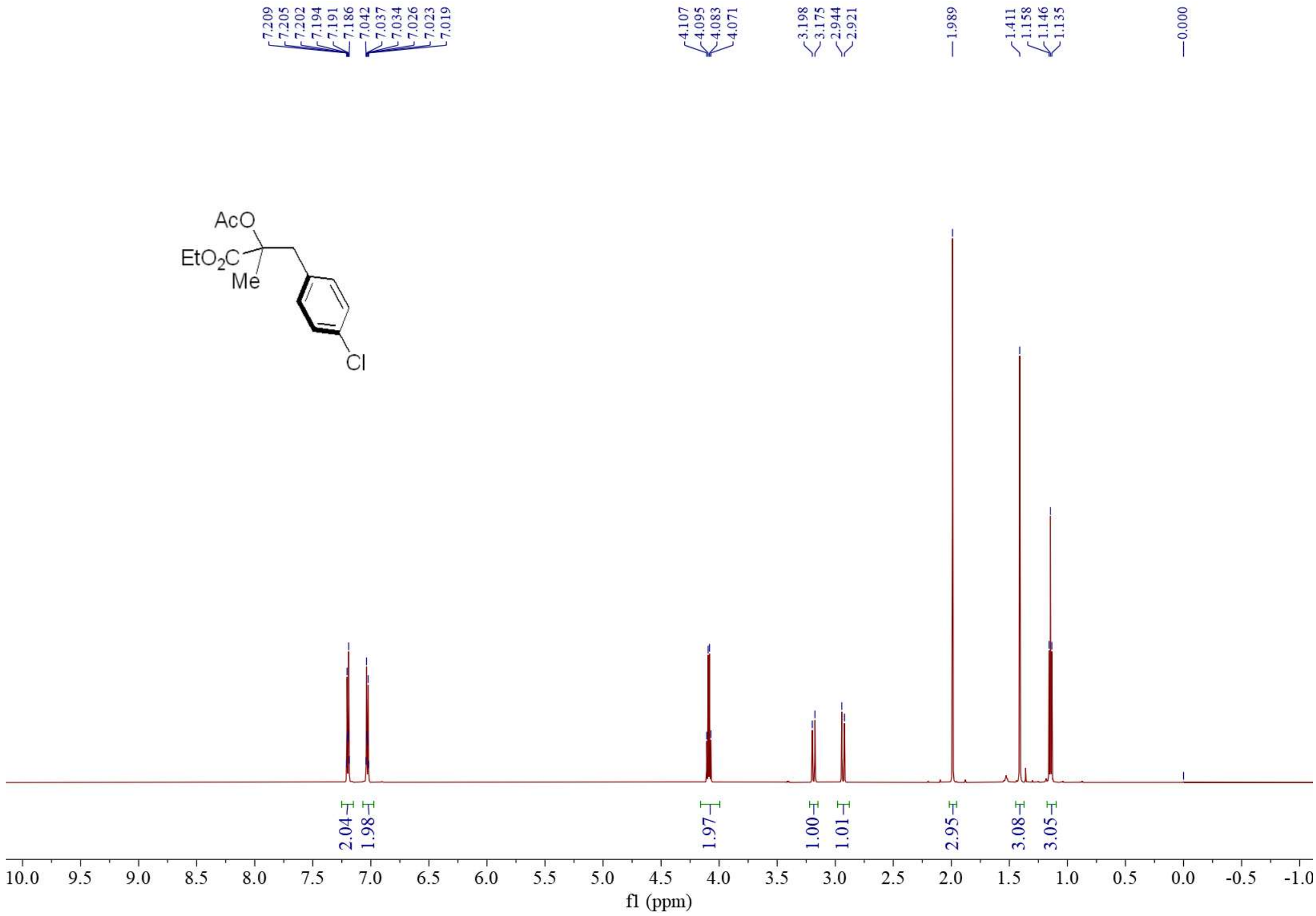

^{13}C NMR spectrum of **8d** (151 MHz, $CDCl_3$)

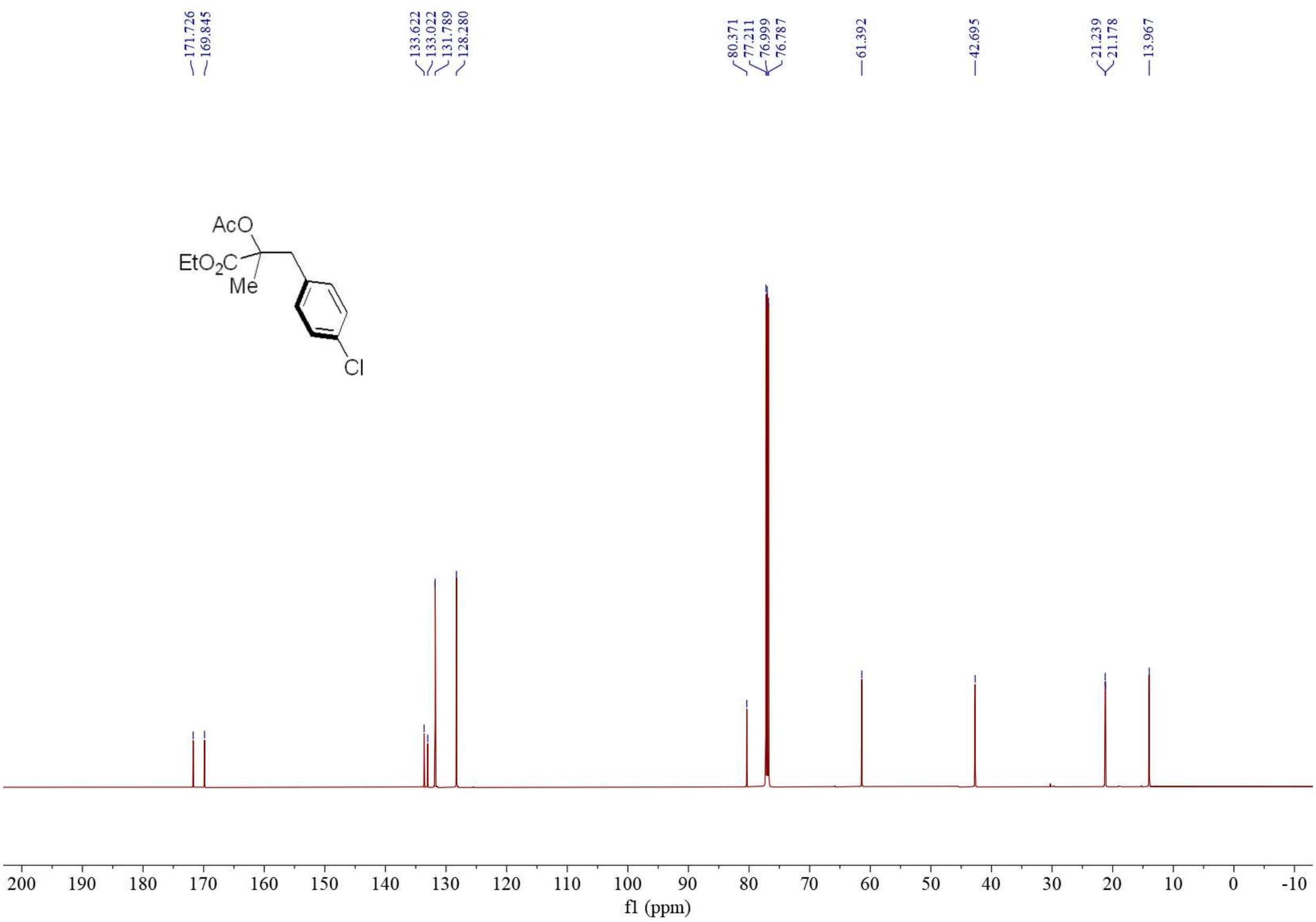

^{1}H NMR spectrum of **8e** (400 MHz, $CDCl_3$)

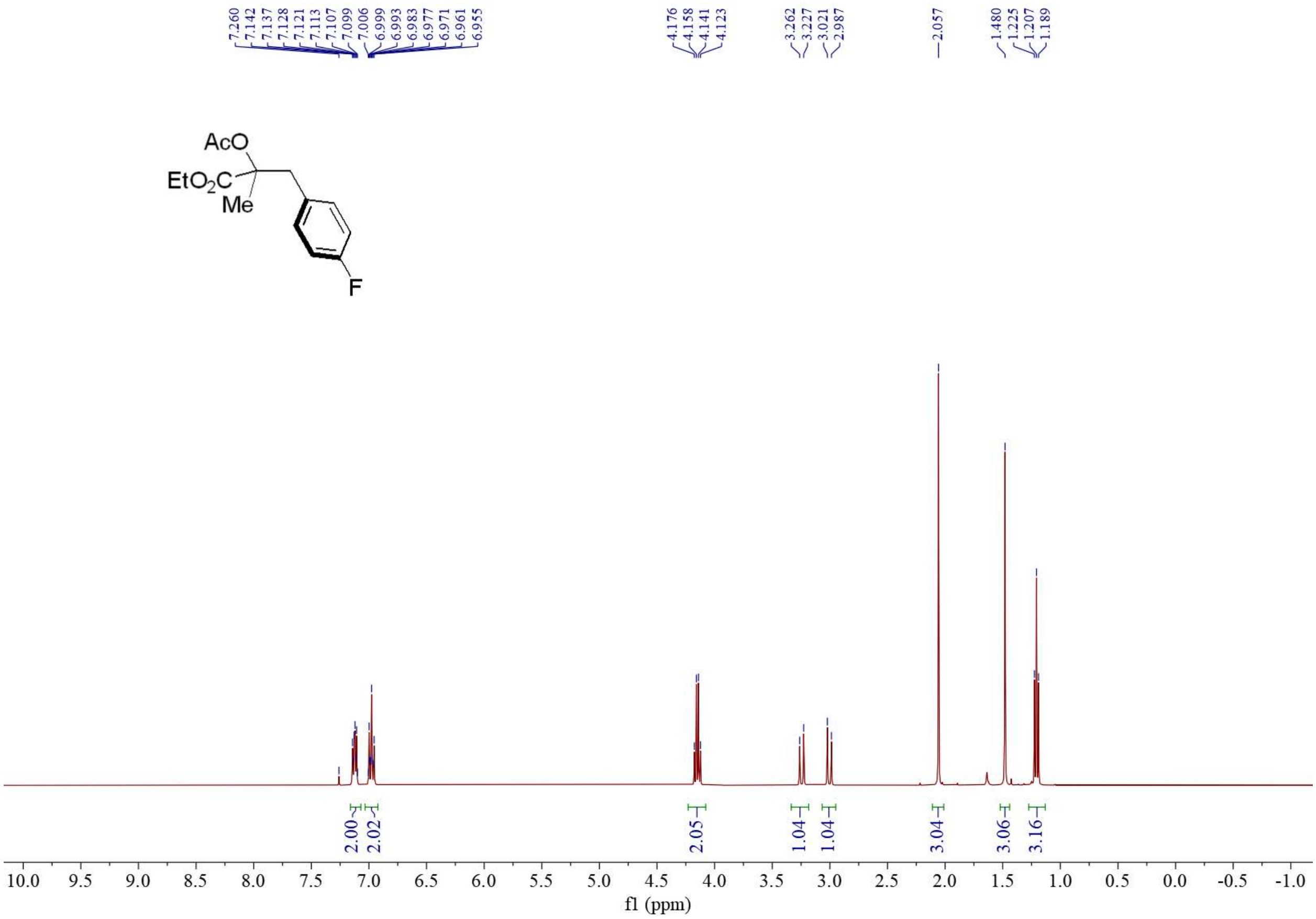

^{13}C NMR spectrum of **8e** (101 MHz, $CDCl_3$)

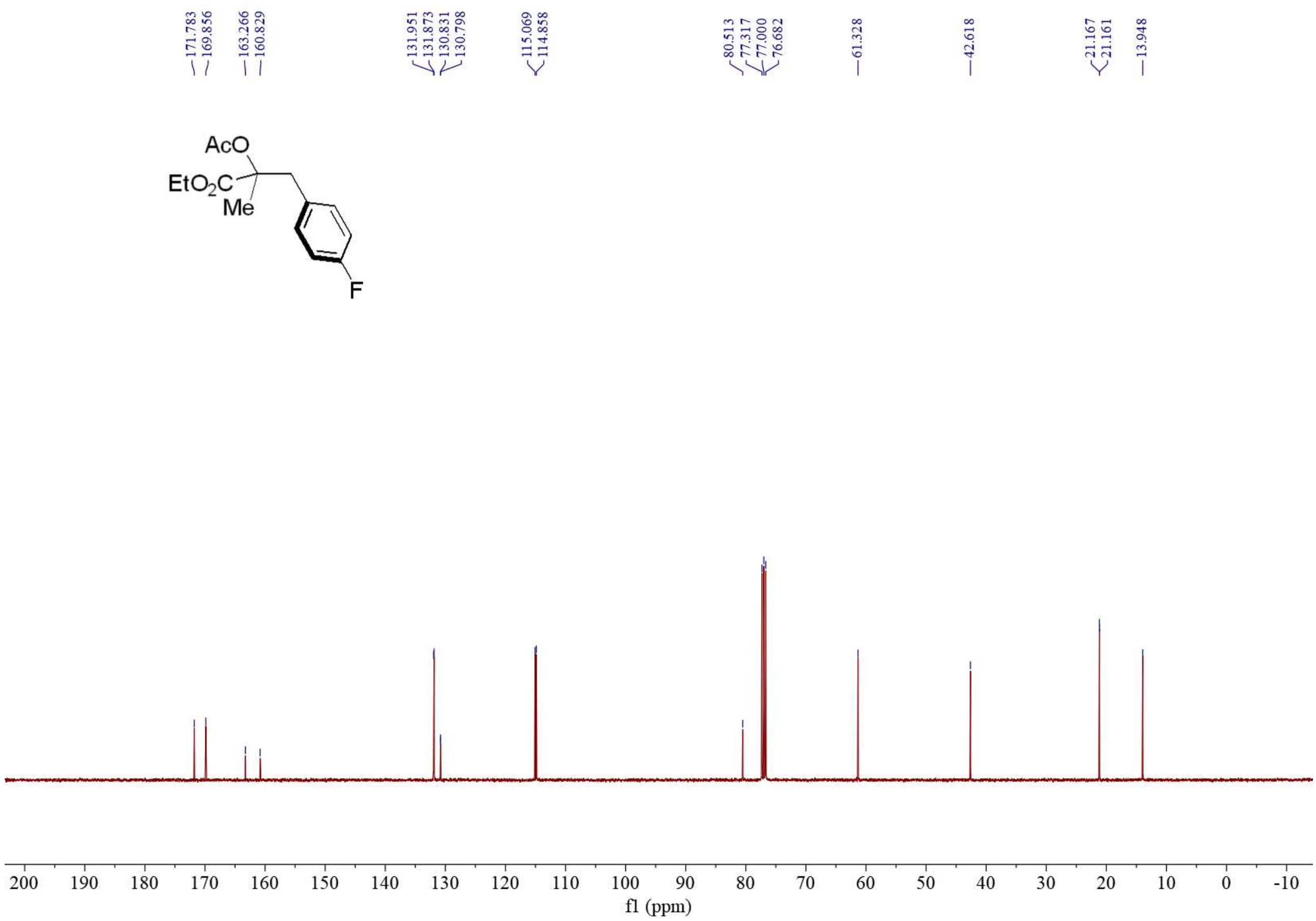

$^{19}F\{^{1}H\}$ NMR spectrum of **8e** (377 MHz, $CDCl_3$)

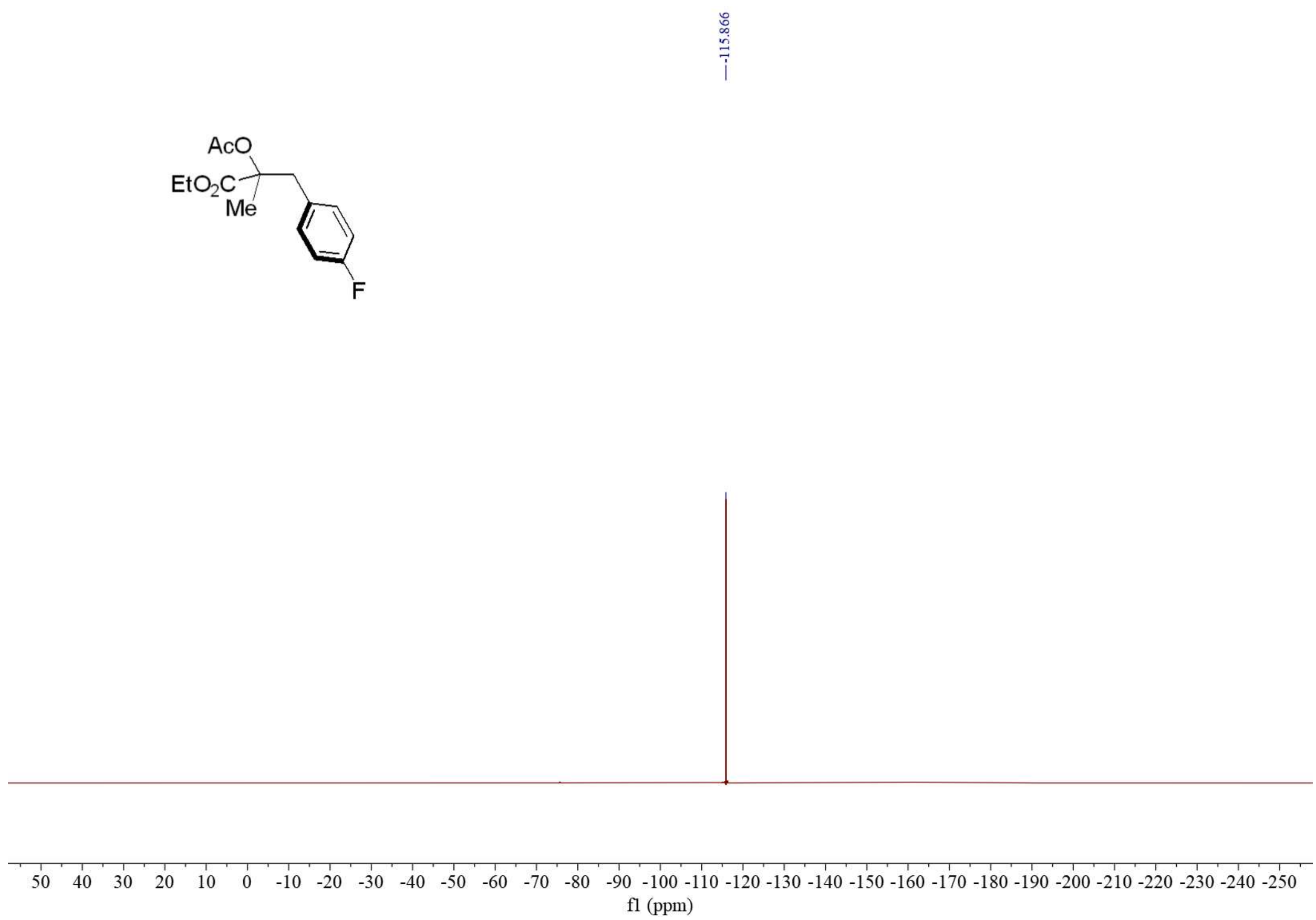

^{19}F NMR spectrum of **8e** (377 MHz, $CDCl_3$)

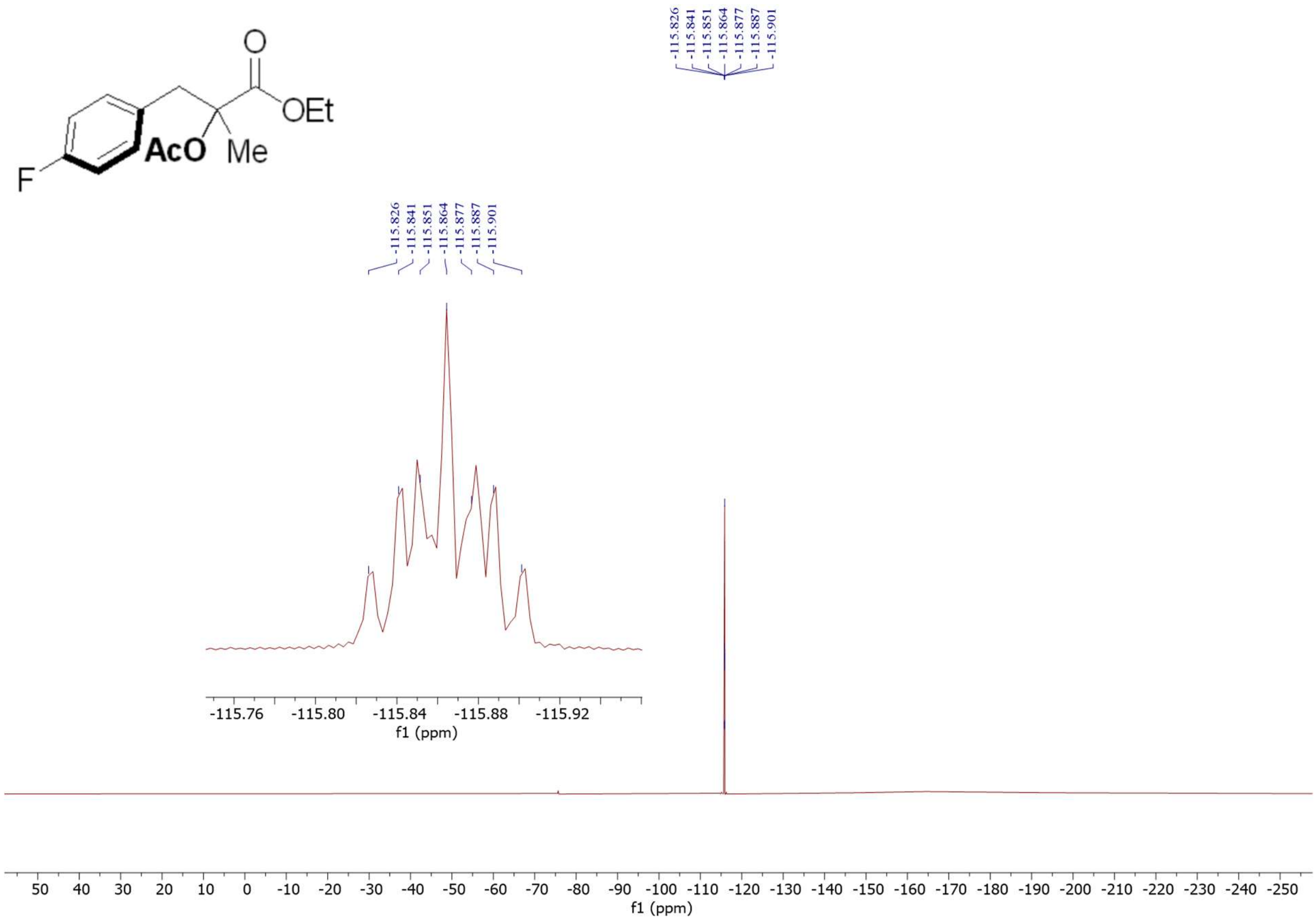

^{1}H NMR spectrum of **8f** (400 MHz, $CDCl_3$)

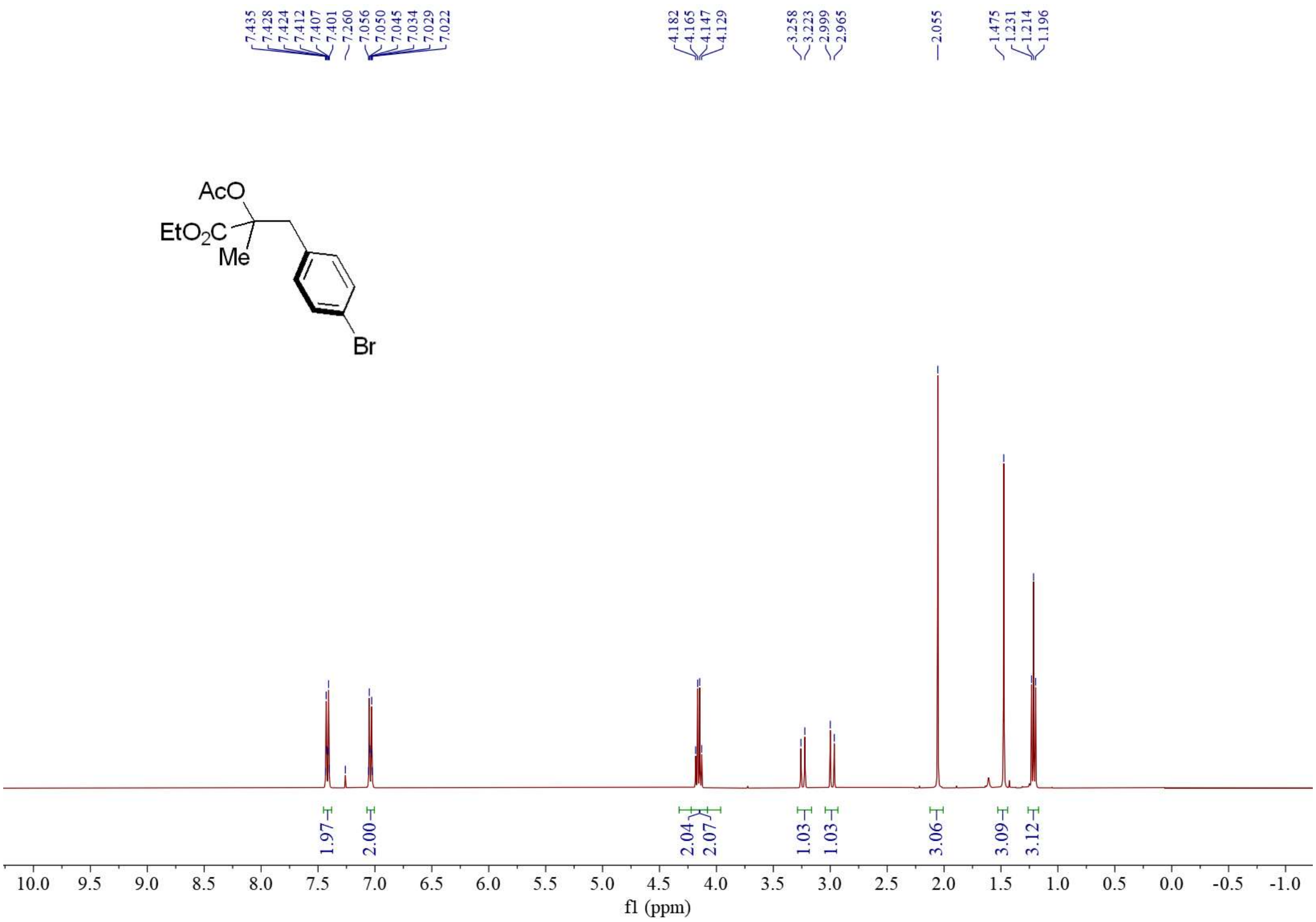

^{13}C NMR spectrum of **8f** (101 MHz, $CDCl_3$)

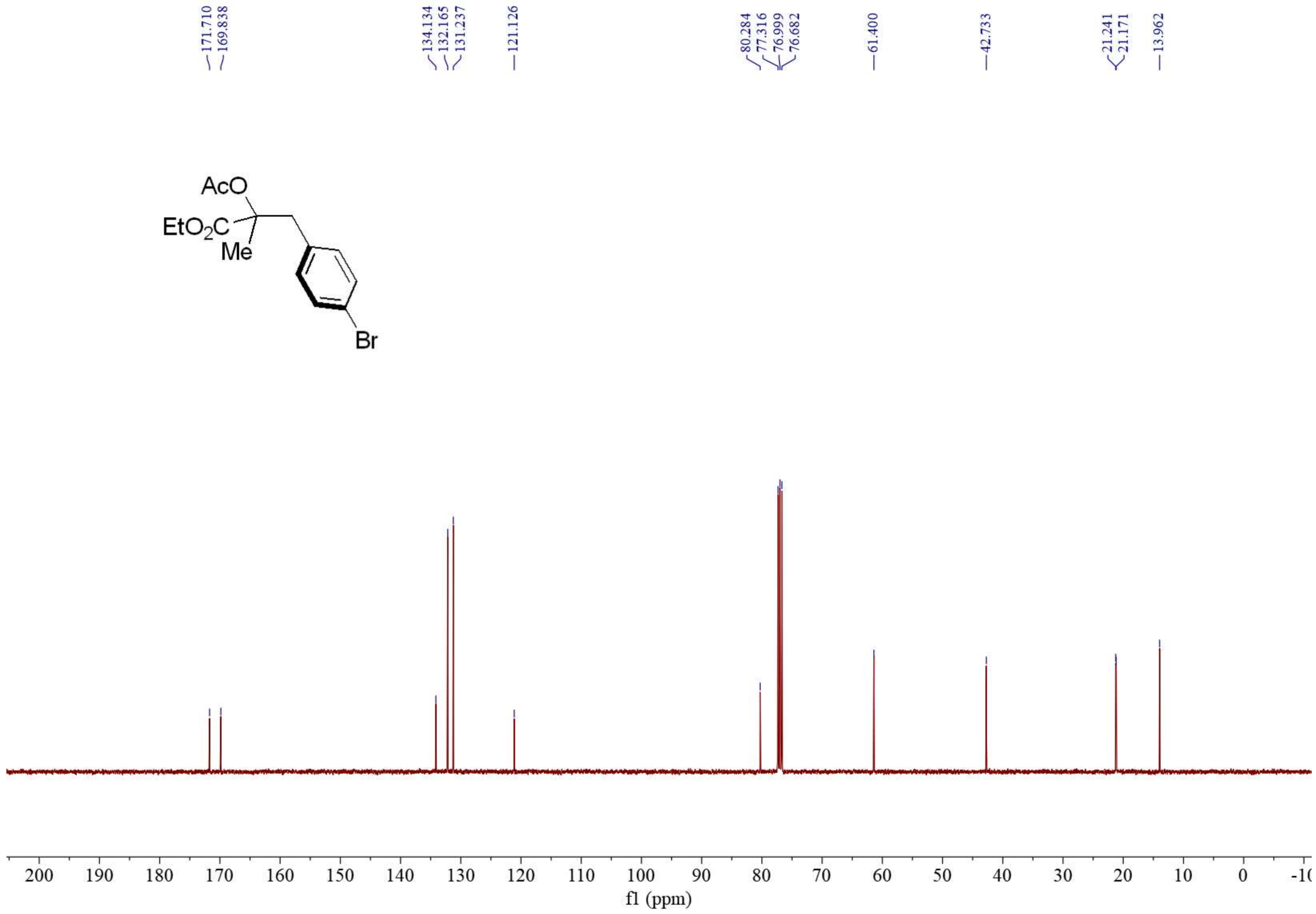

^{1}H NMR spectrum of **8g** (400 MHz, $CDCl_3$)

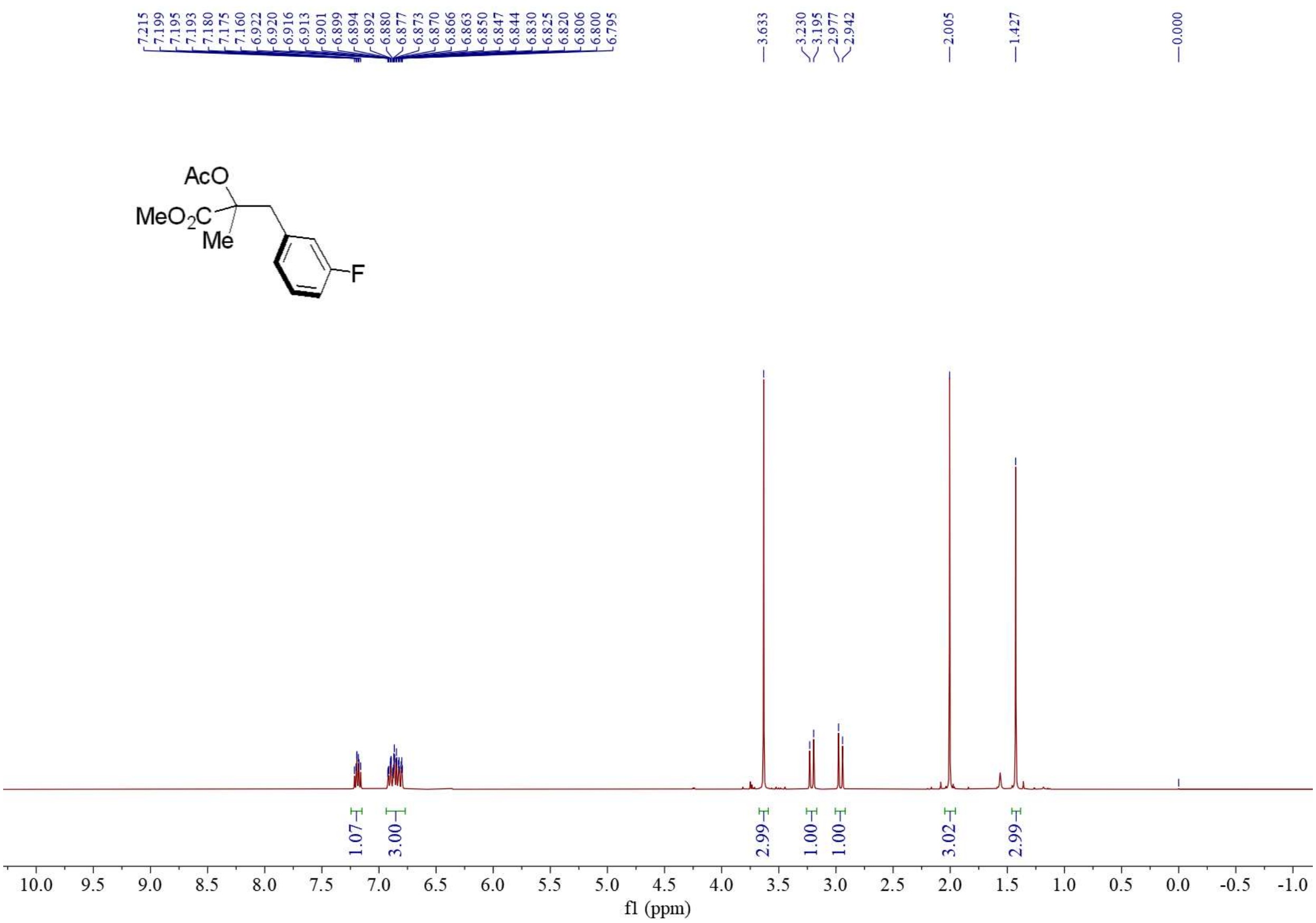

^{13}C NMR spectrum of **8g** (101 MHz, $CDCl_3$)

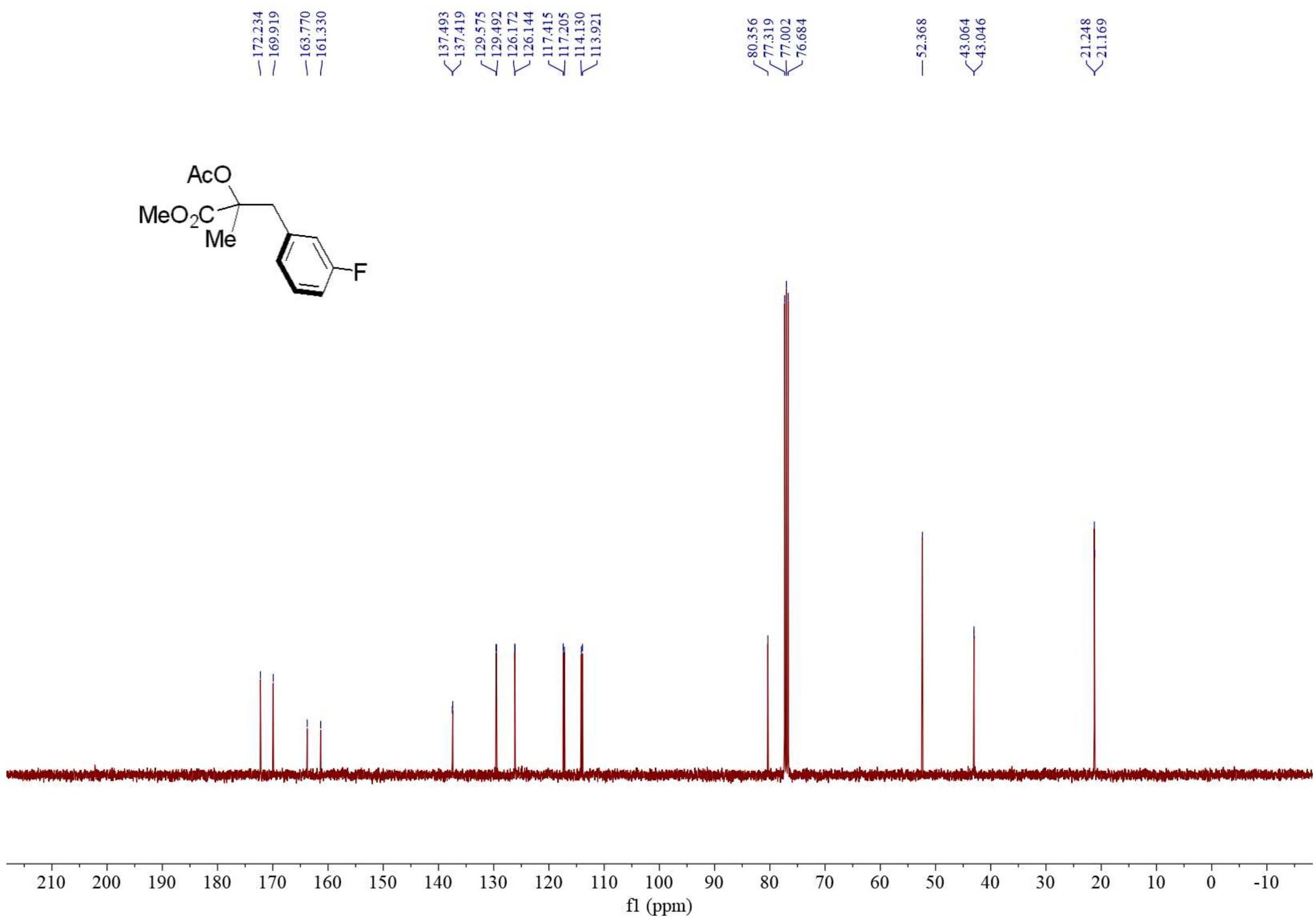

$^{19}F\{^1H\}$ NMR spectrum of **8g** (377 MHz, $CDCl_3$)

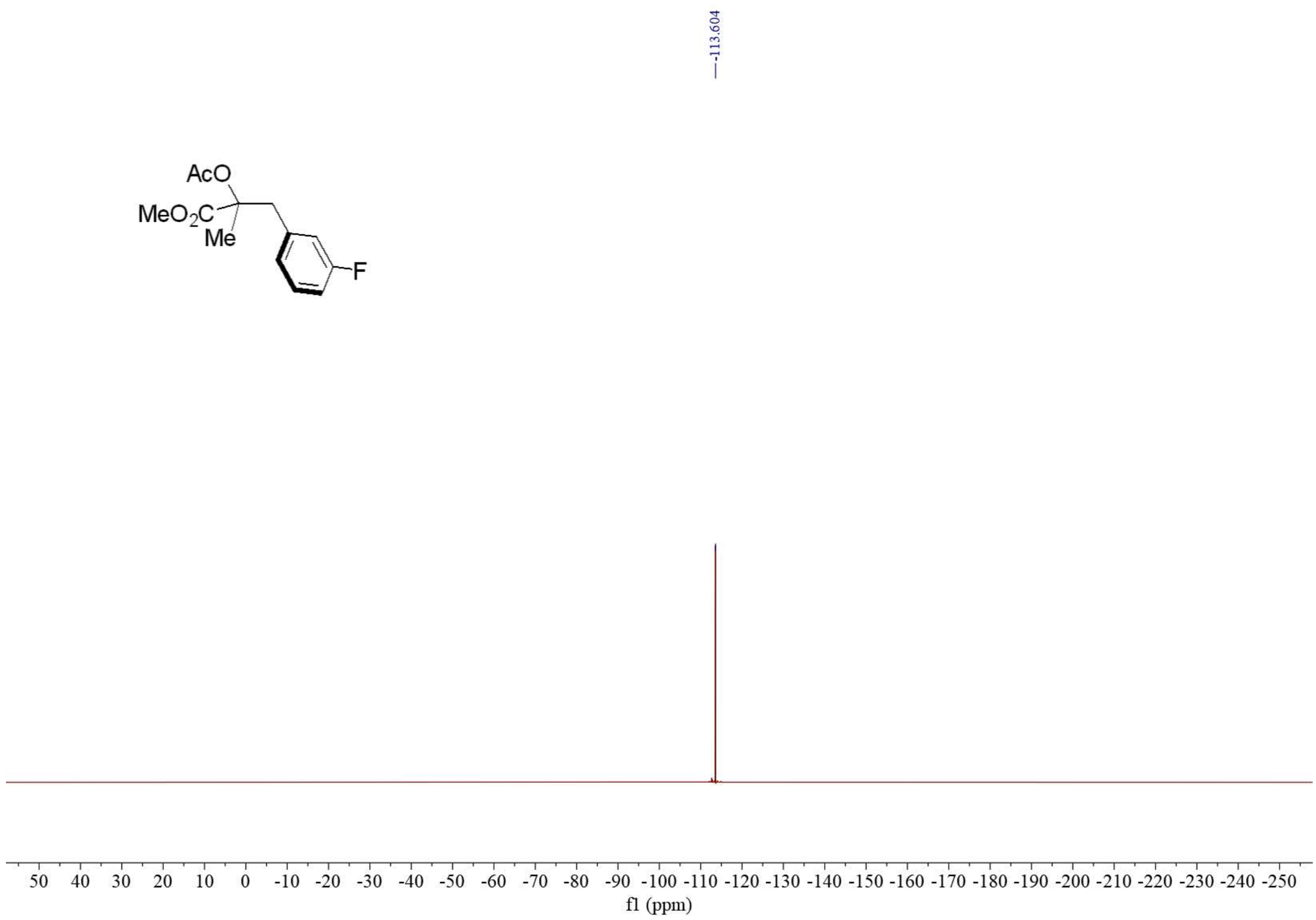

^{19}F NMR spectrum of **8g** (377 MHz, $CDCl_3$)

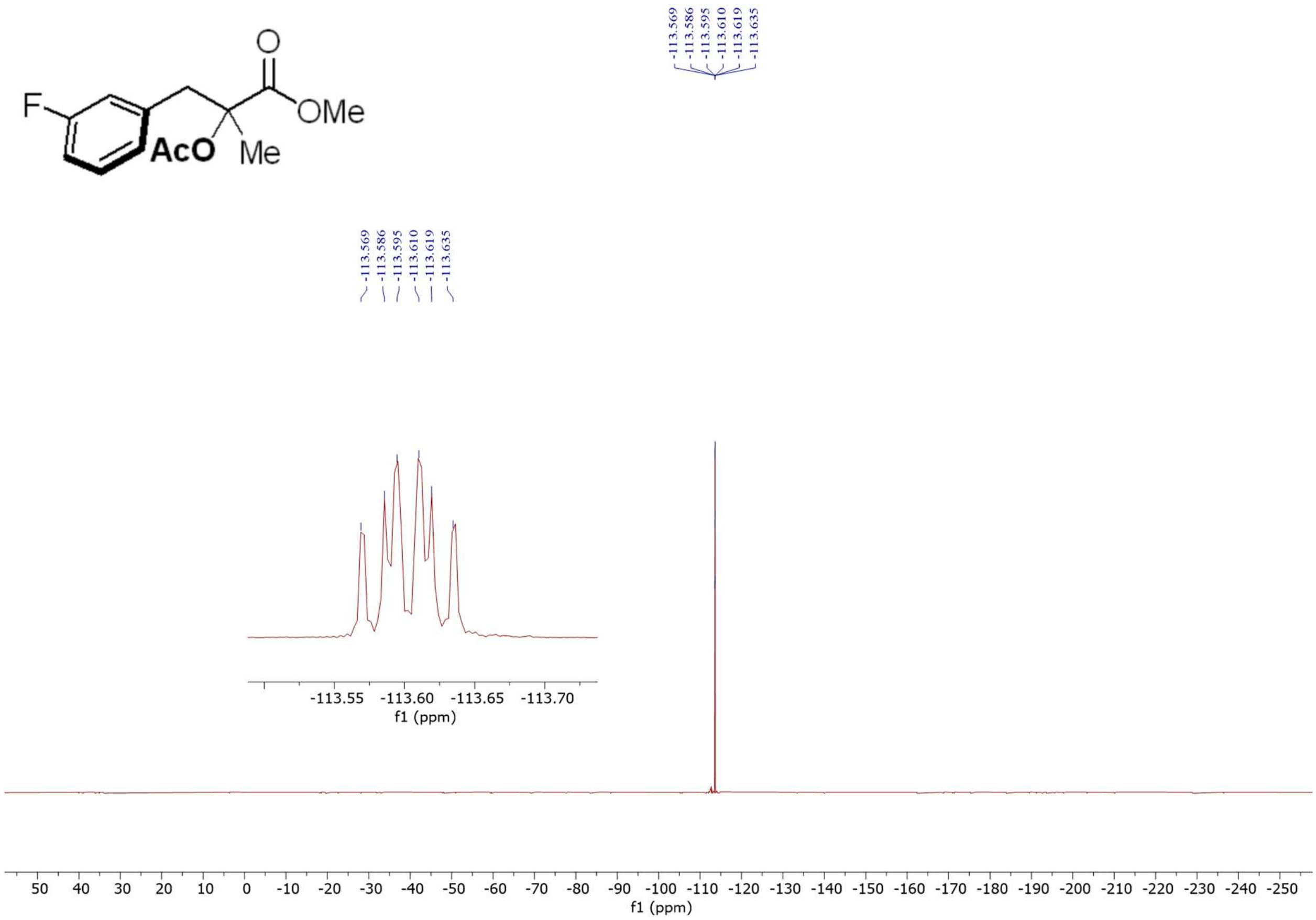

^{1}H NMR spectrum of **8h** (600 MHz, $CDCl_3$)

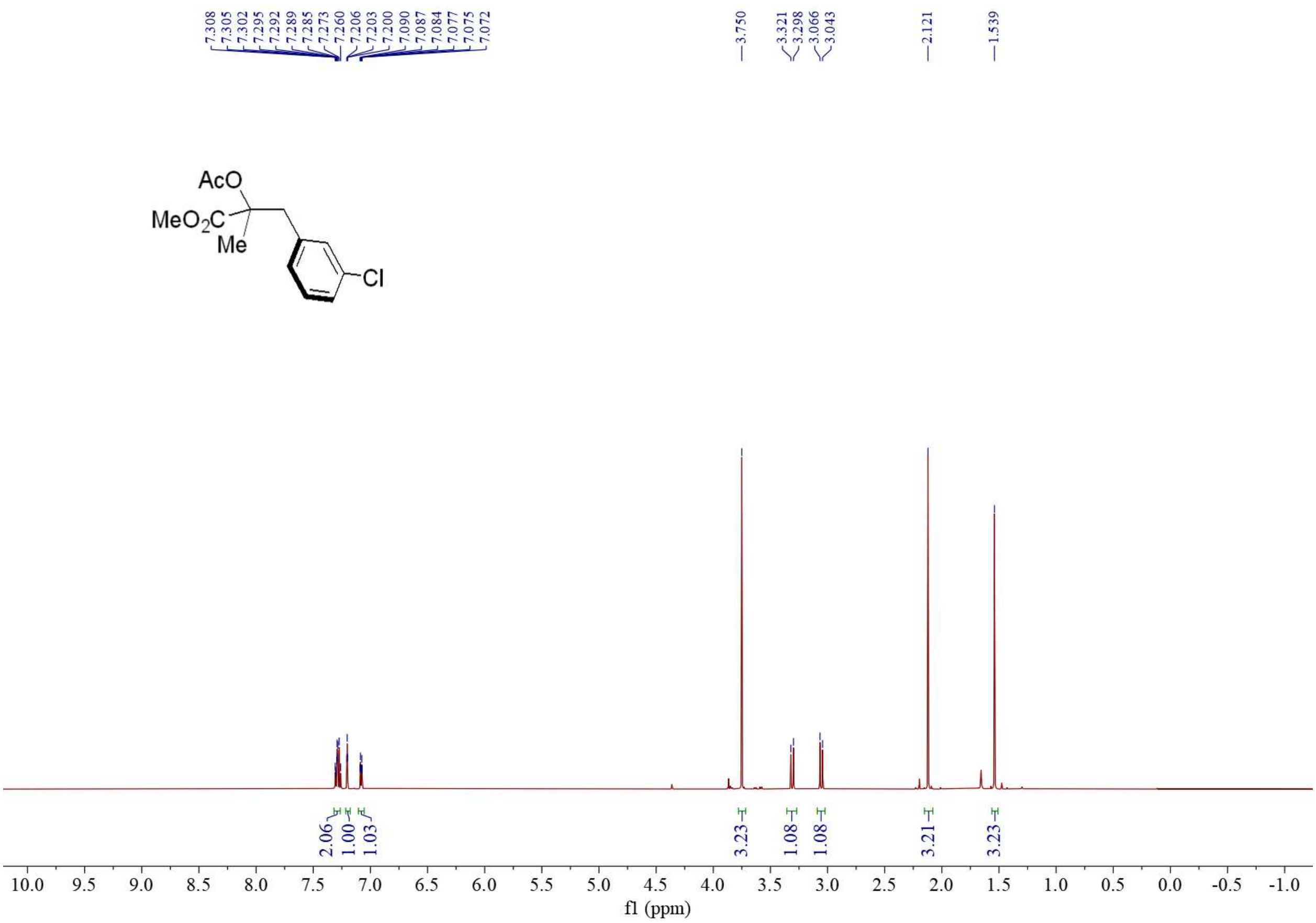

^{13}C NMR spectrum of **8h** (151 MHz, $CDCl_3$)

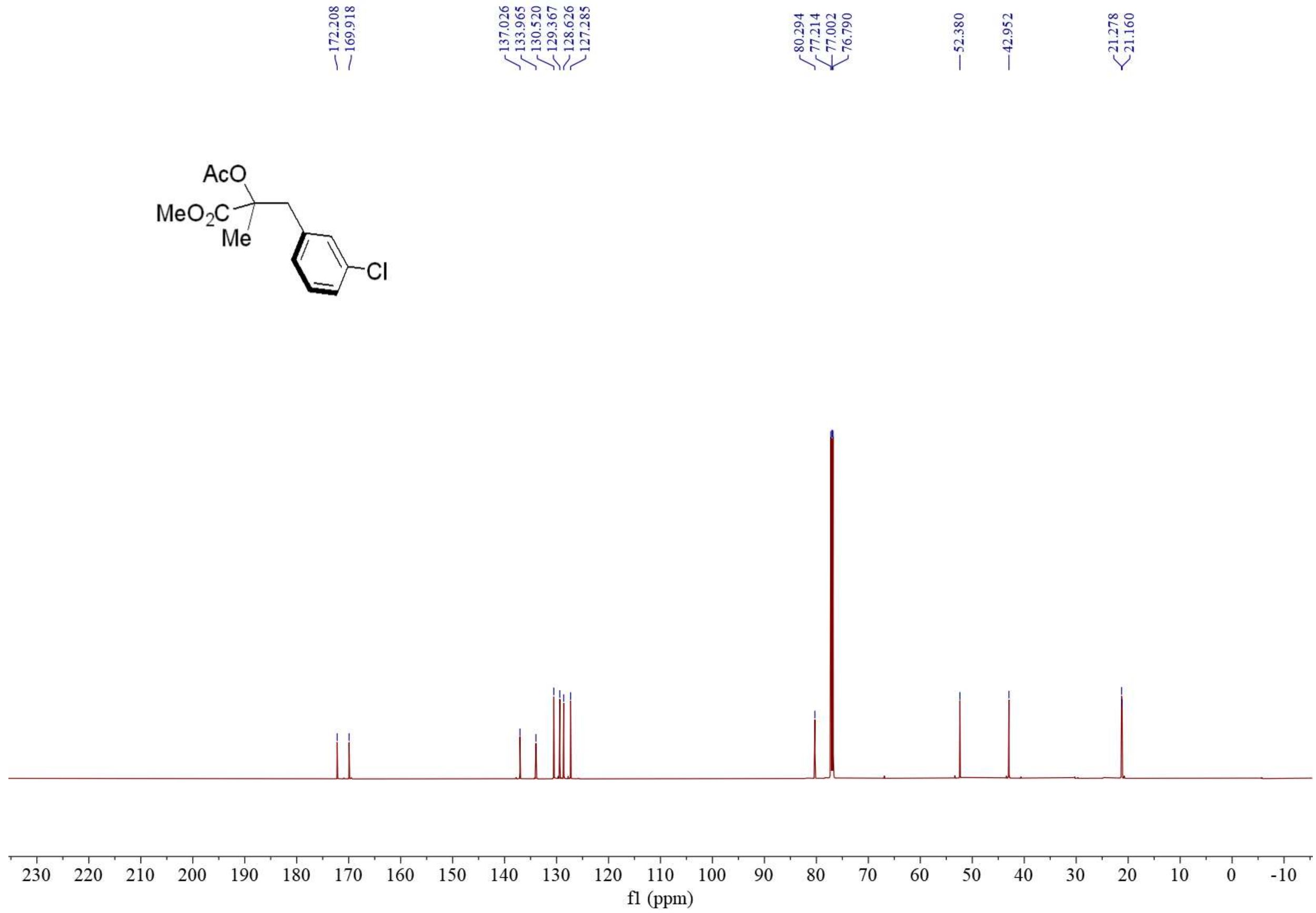

^{1}H NMR spectrum of **8i** (500 MHz, $CDCl_3$)

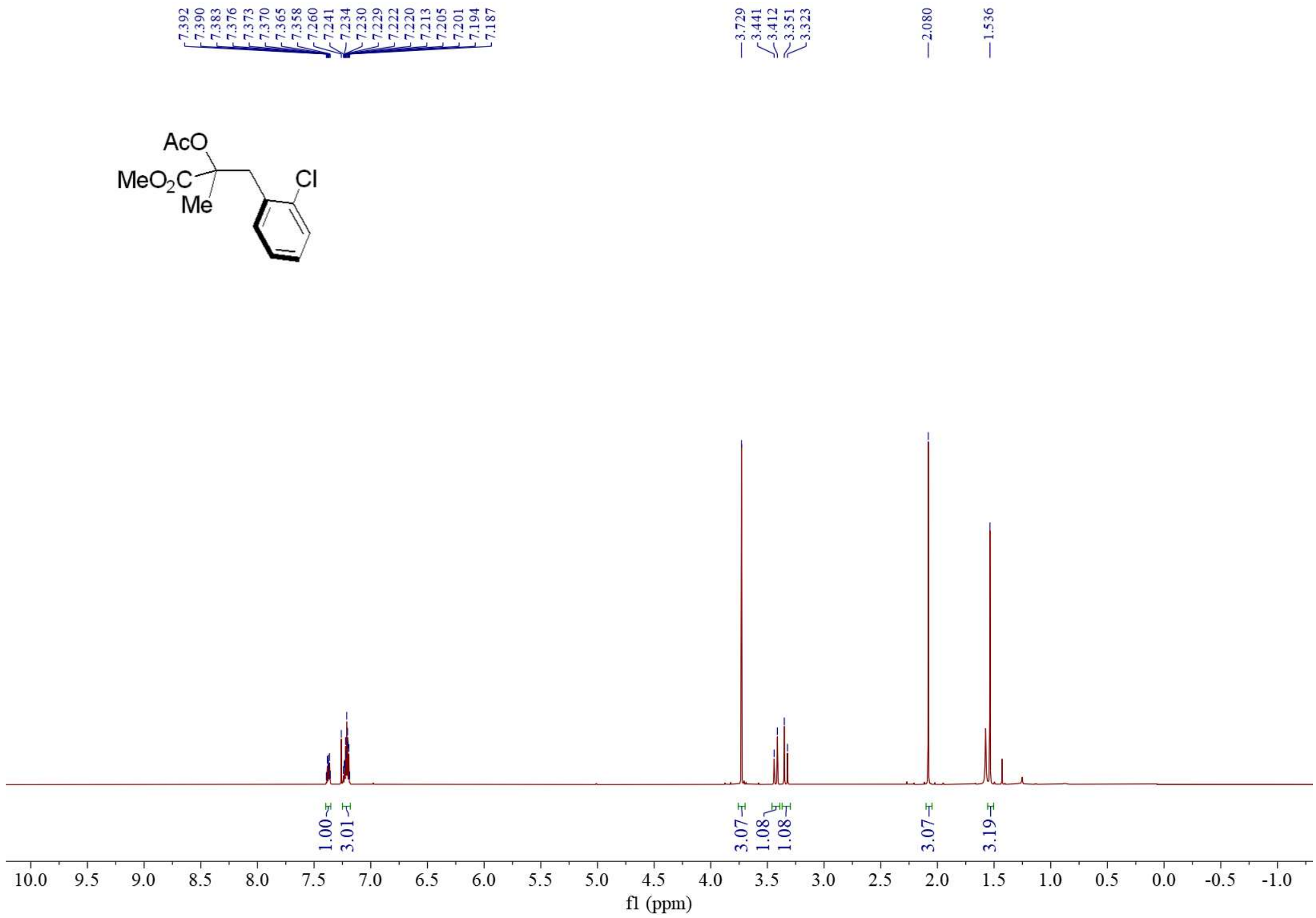

^{13}C NMR spectrum of **8i** (126 MHz, $CDCl_3$)

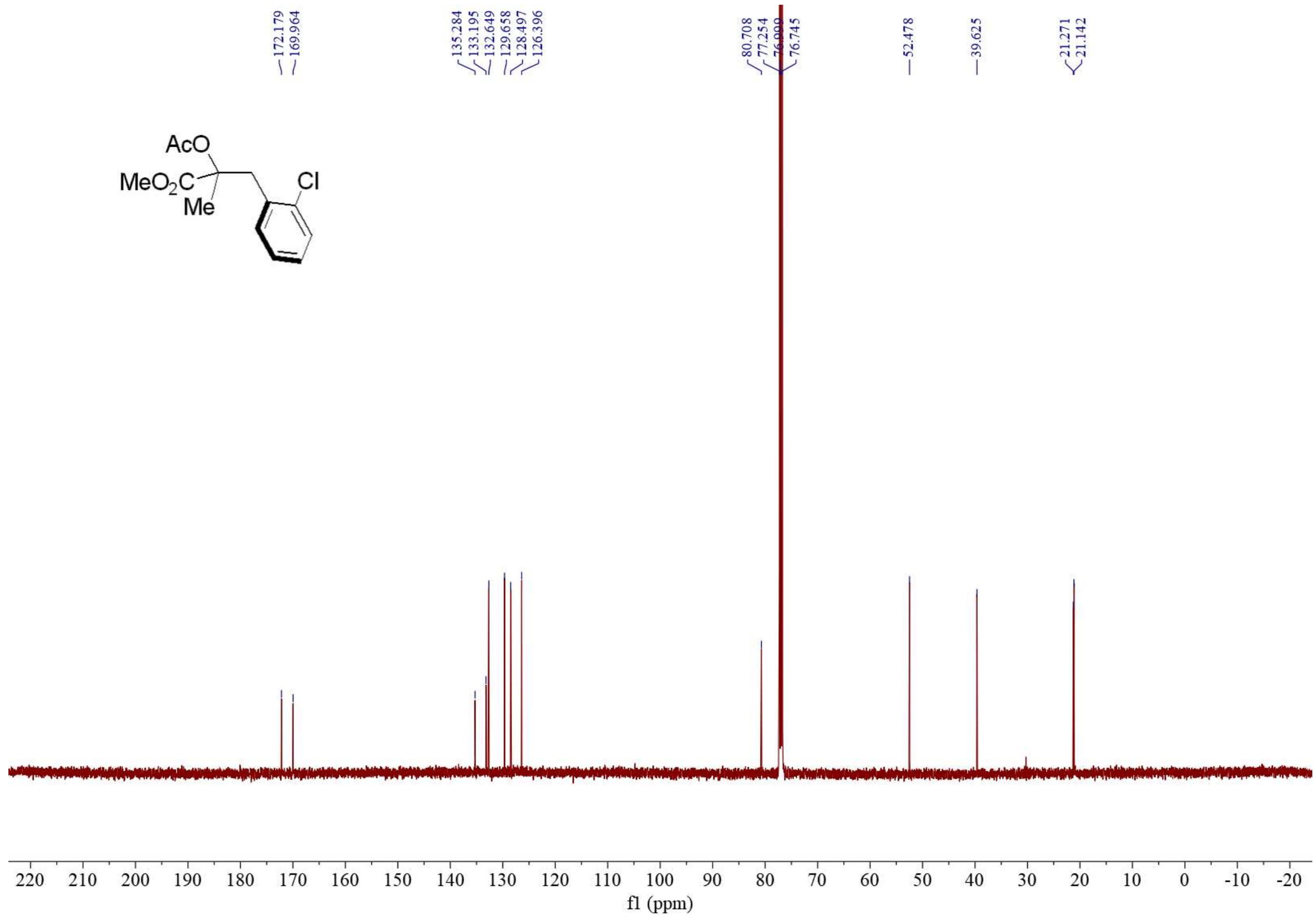

^{1}H NMR spectrum of **8j** (500 MHz, $CDCl_3$)

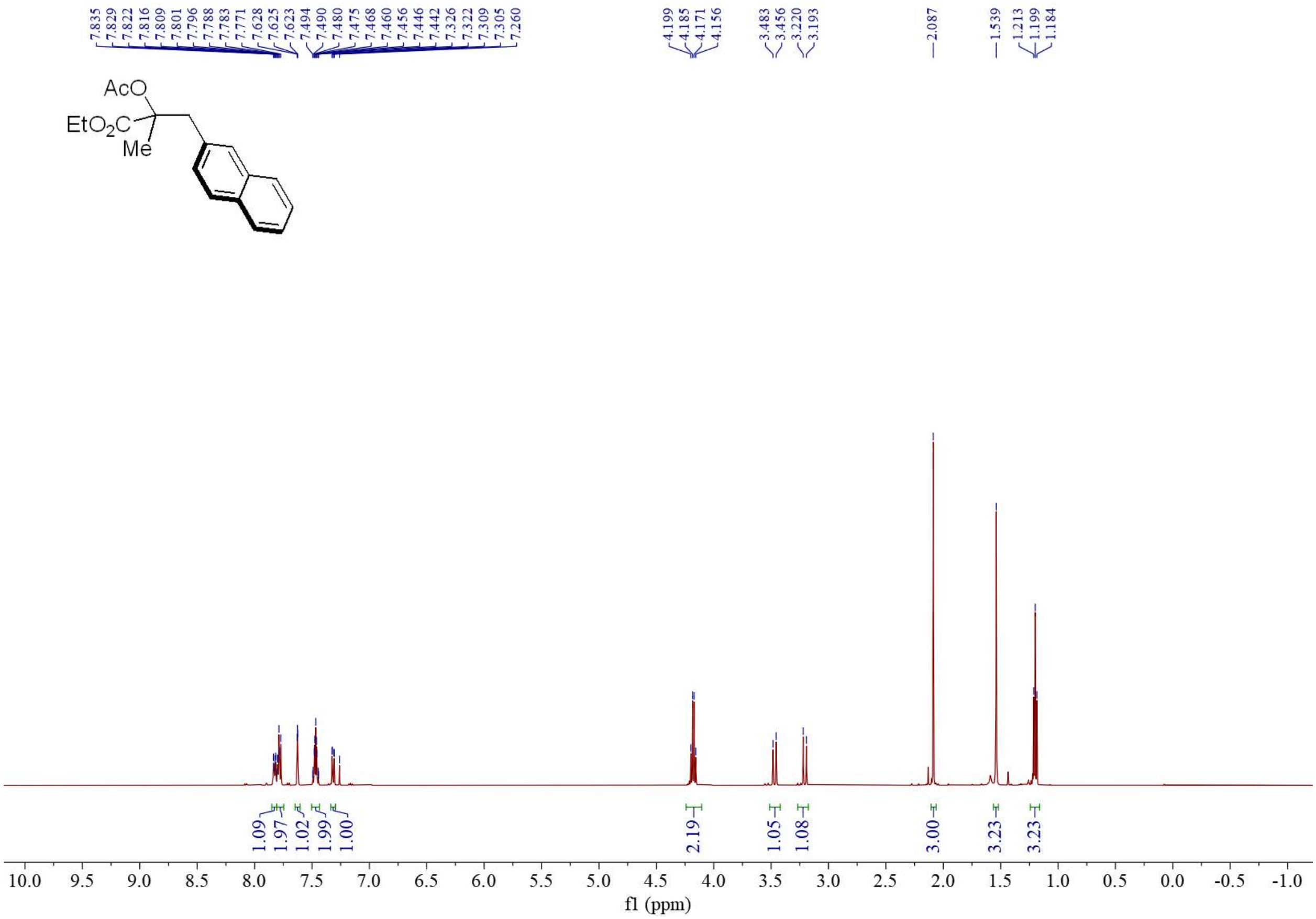

^{13}C NMR spectrum of **8j** (126 MHz, $CDCl_3$)

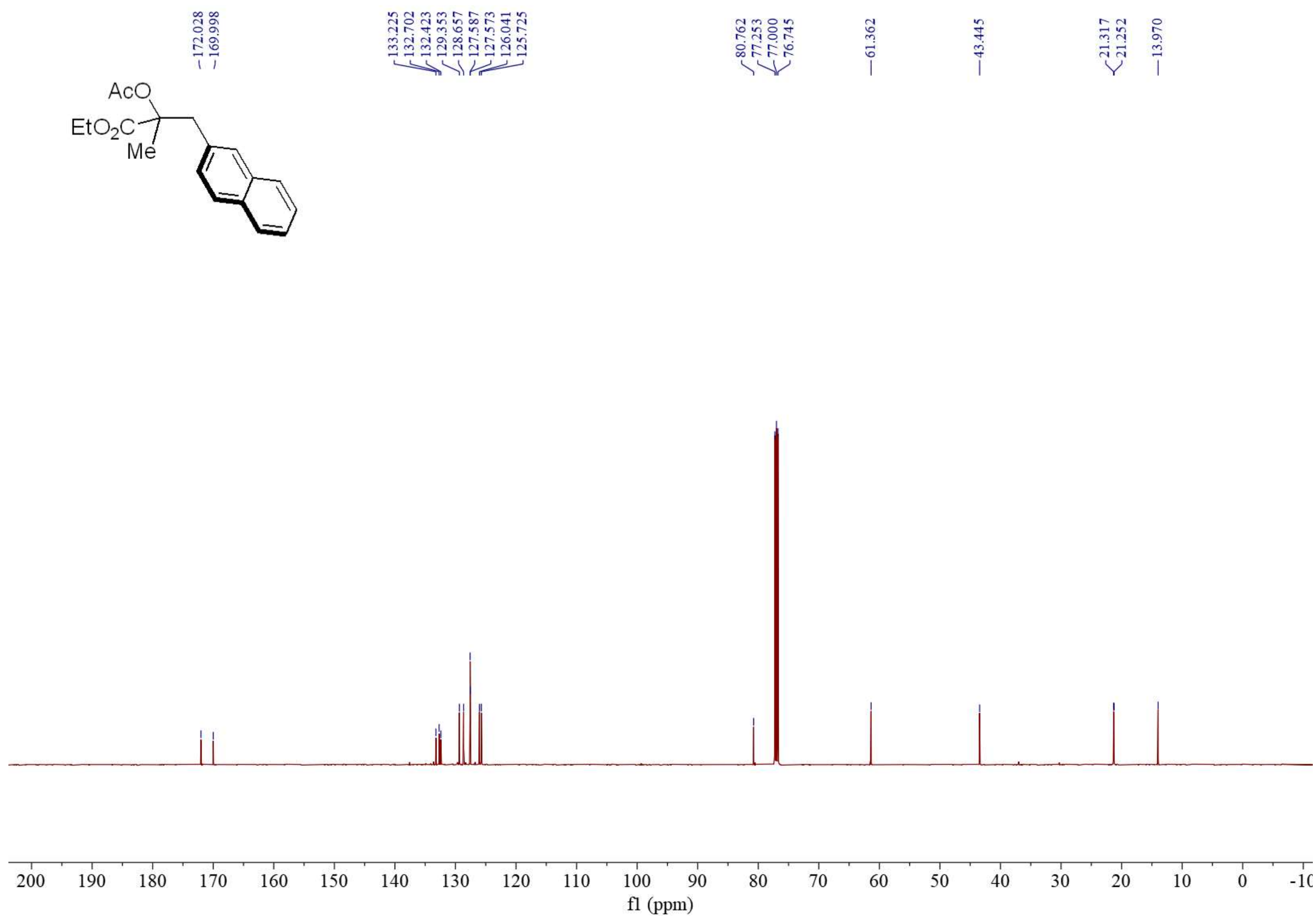

^{1}H NMR spectrum of **8k** (500 MHz, $CDCl_3$)

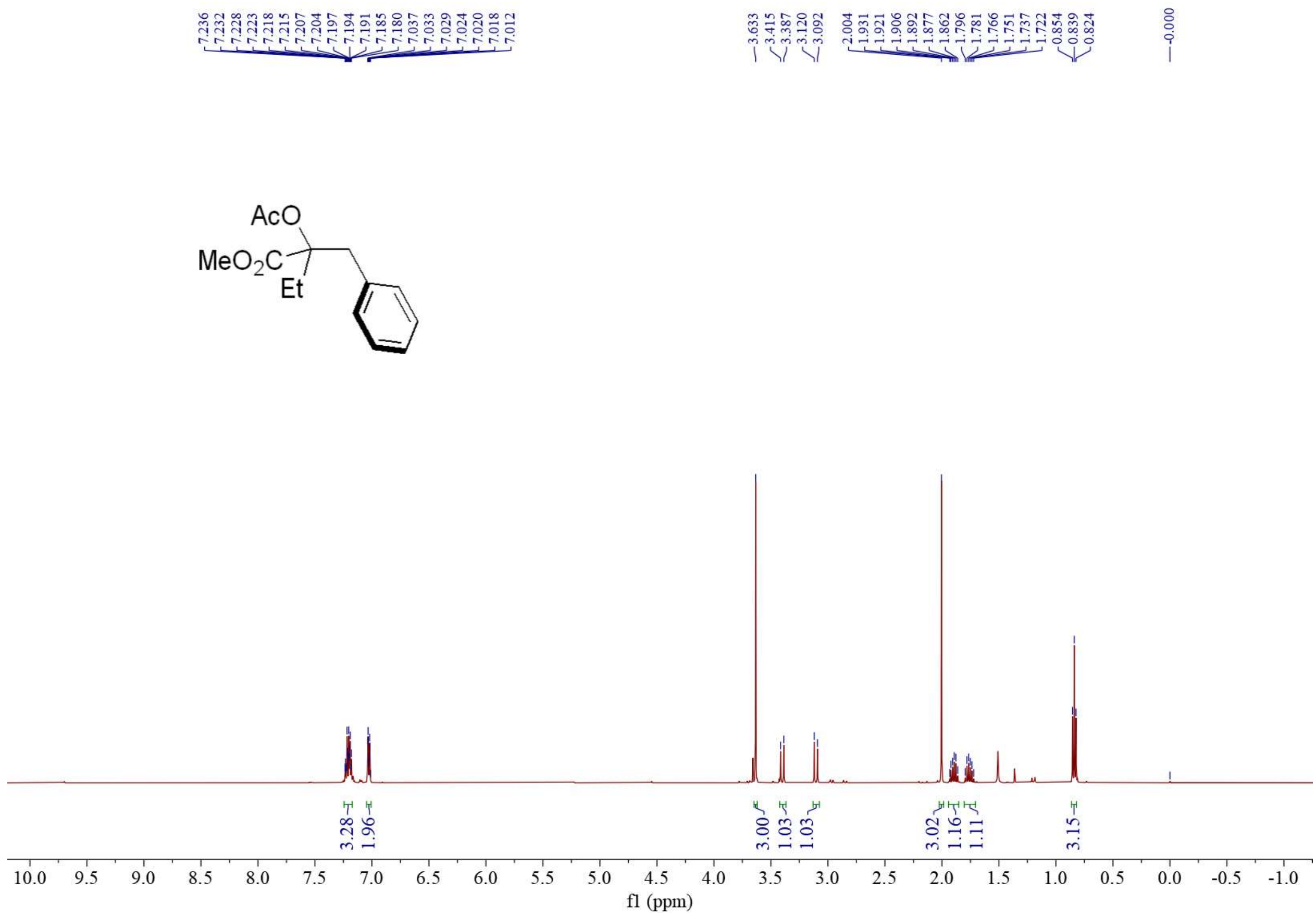

^{13}C NMR spectrum of **8k** (126 MHz, $CDCl_3$)

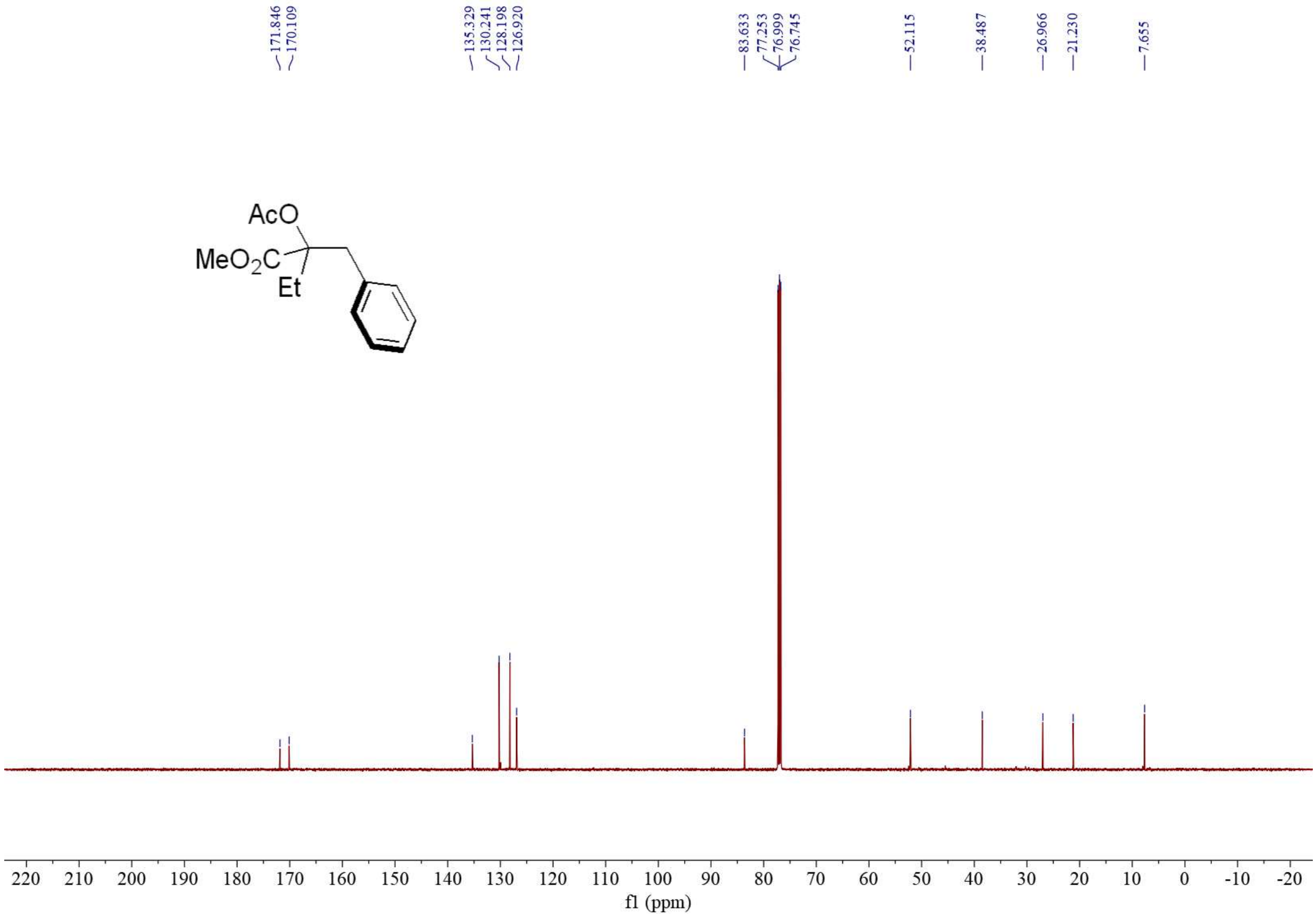

^{1}H NMR spectrum of **8l** (500 MHz, $CDCl_3$)

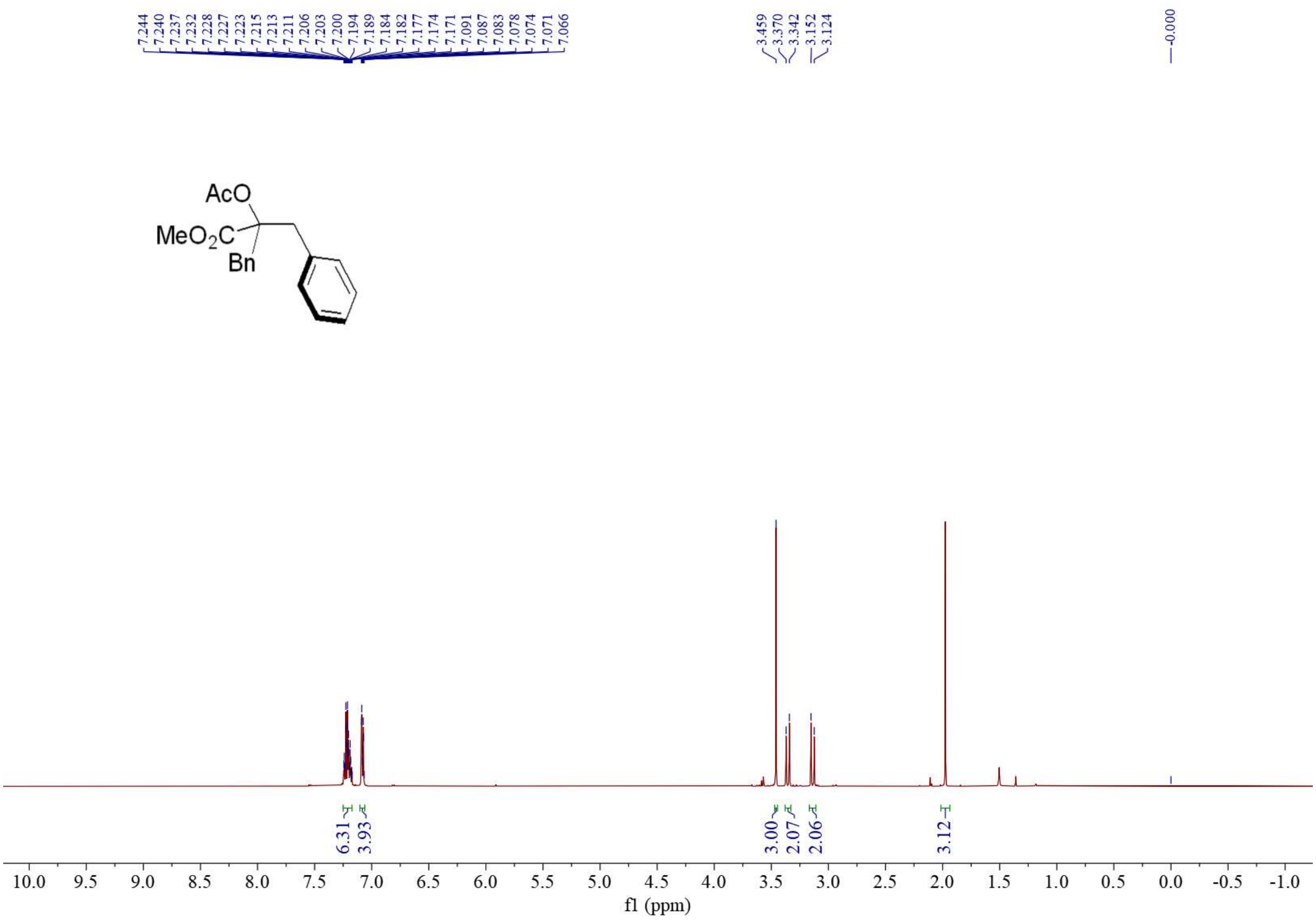

^{13}C NMR spectrum of **8l** (126 MHz, $CDCl_3$)

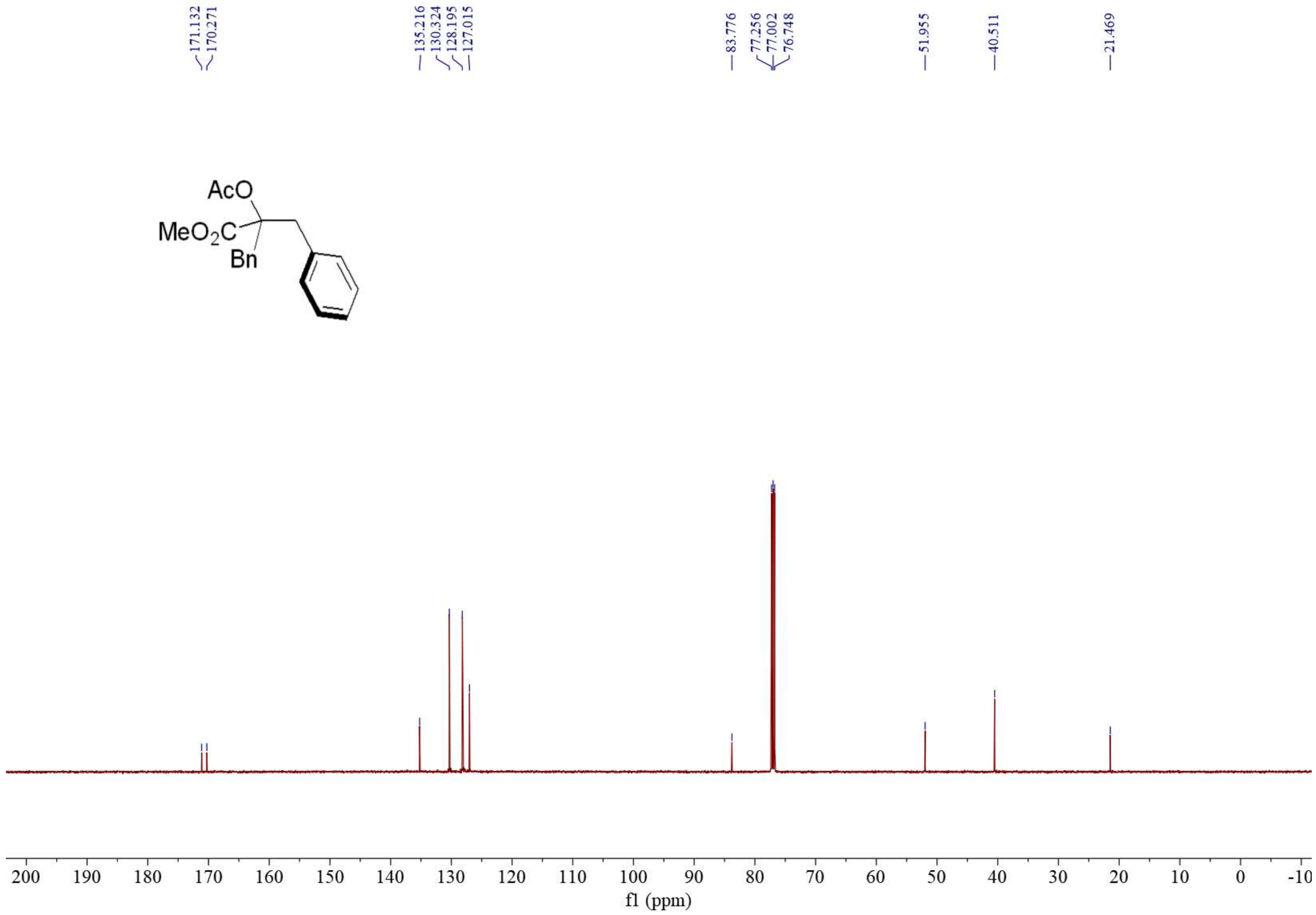

^{1}H NMR spectrum of **8m** (600 MHz, $CDCl_3$)

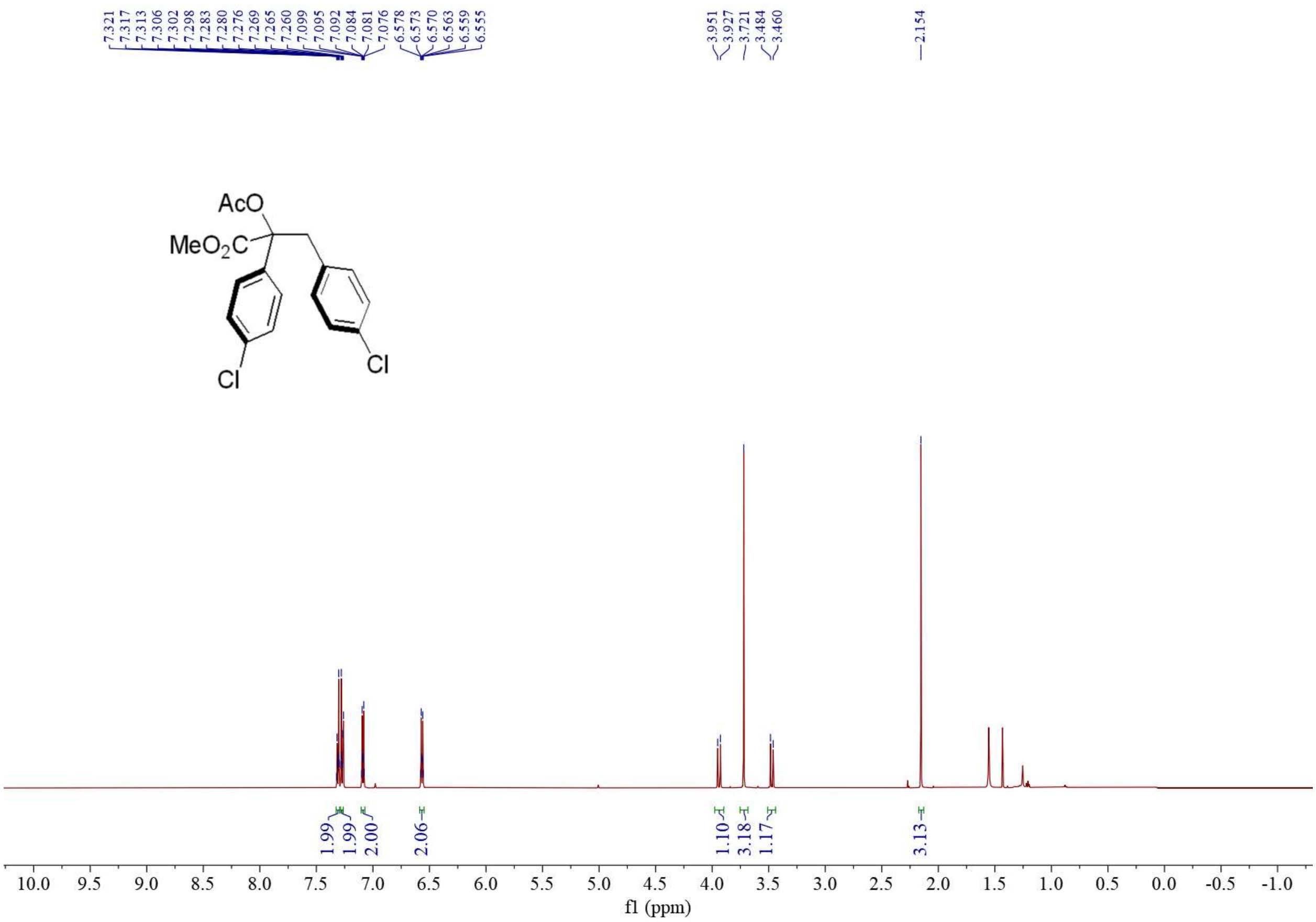

^{13}C NMR spectrum of **8m** (151 MHz, $CDCl_3$)

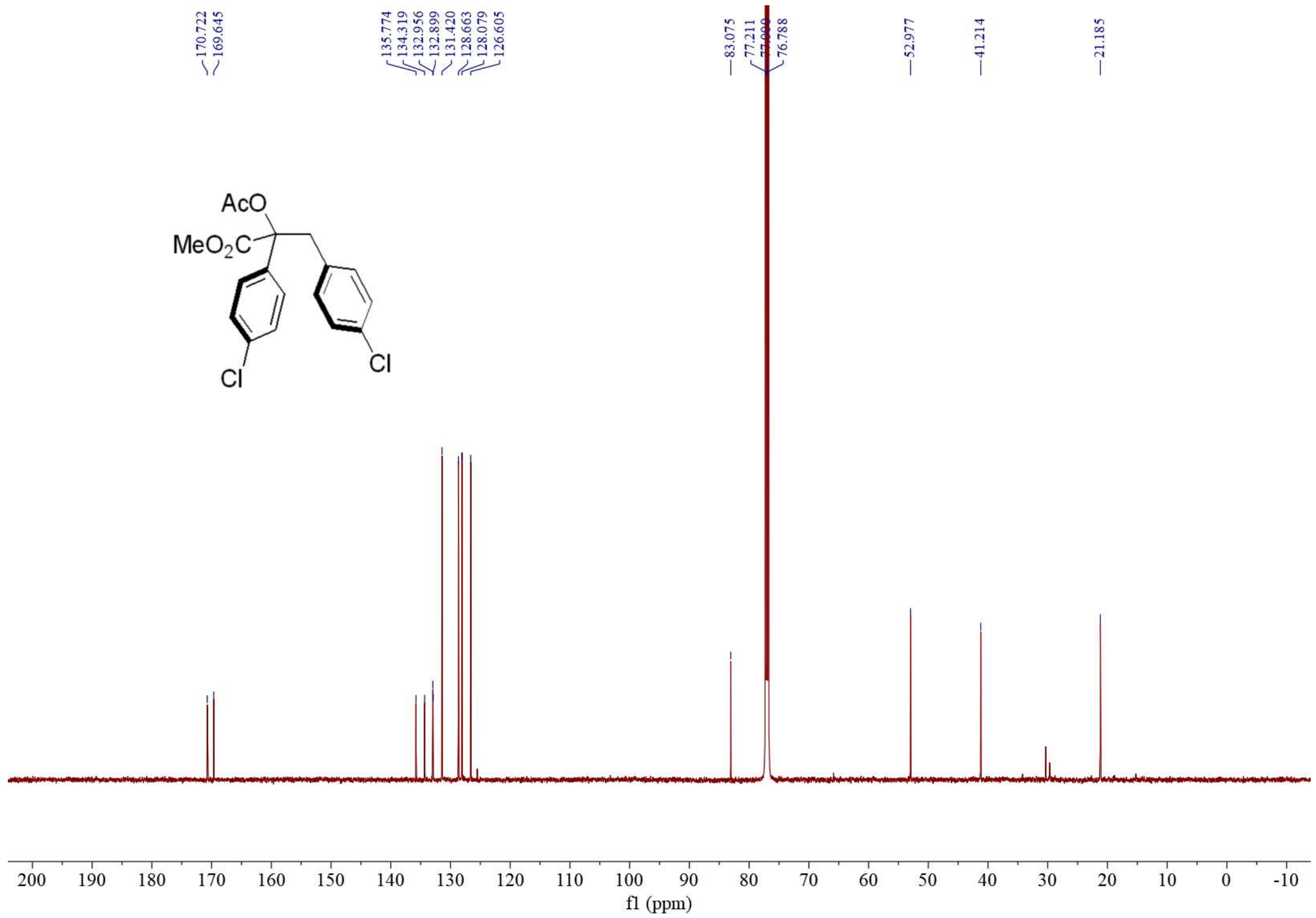

^1H NMR spectrum of **8n** (400 MHz, $CDCl_3$)

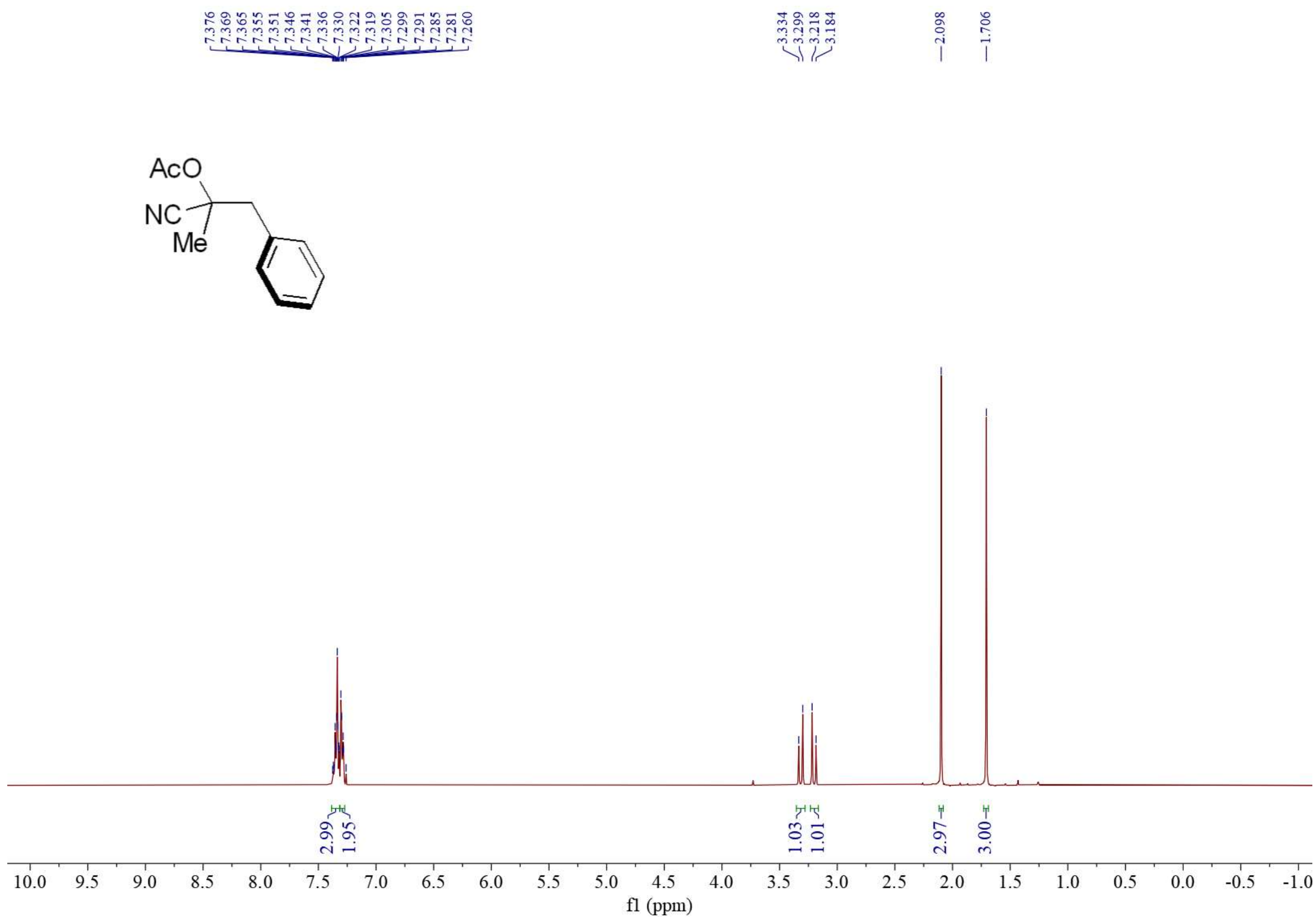

^{13}C NMR spectrum of **8n** (101 MHz, $CDCl_3$)

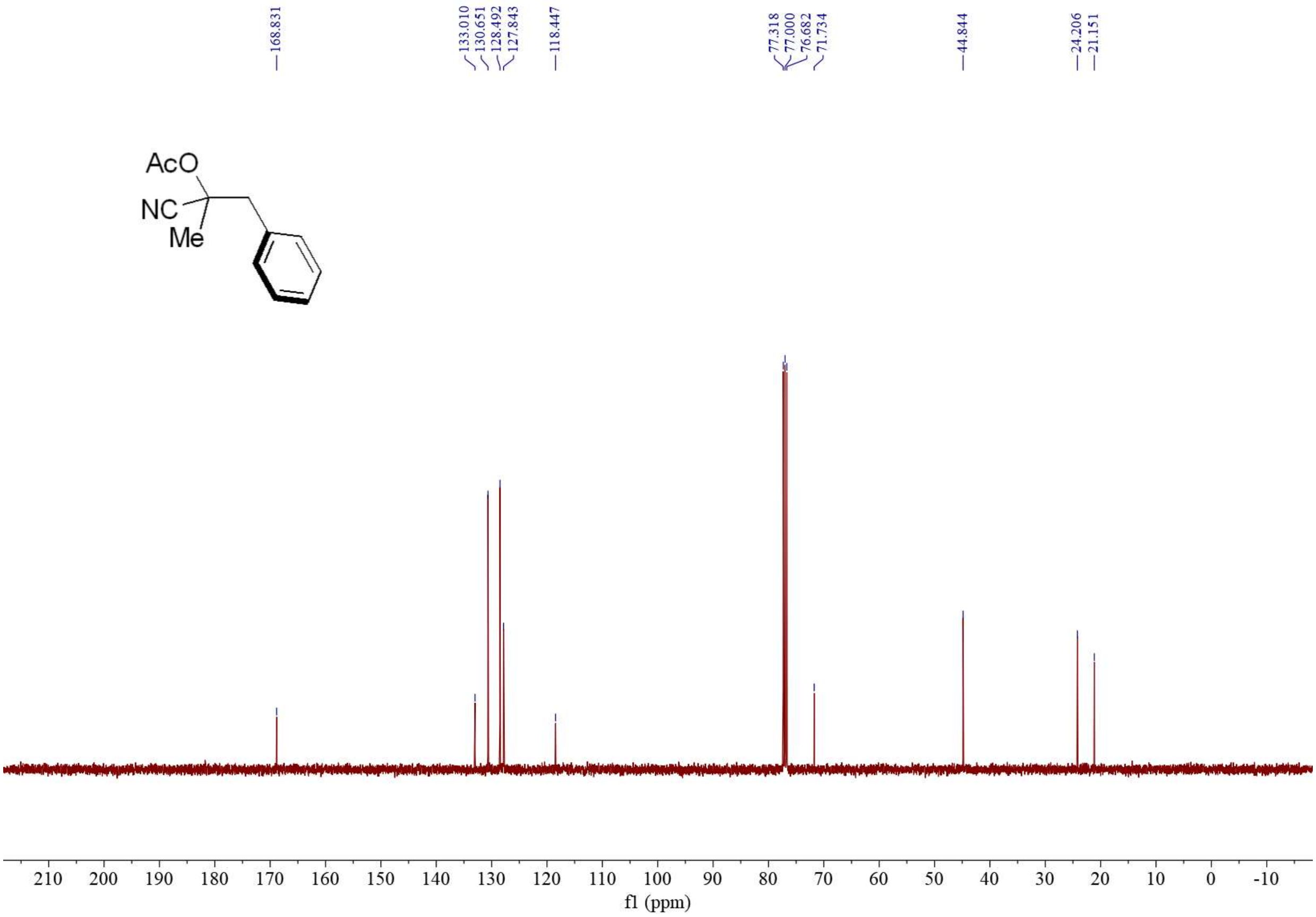

^{1}H NMR spectrum of **8o** (600 MHz, $CDCl_3$)

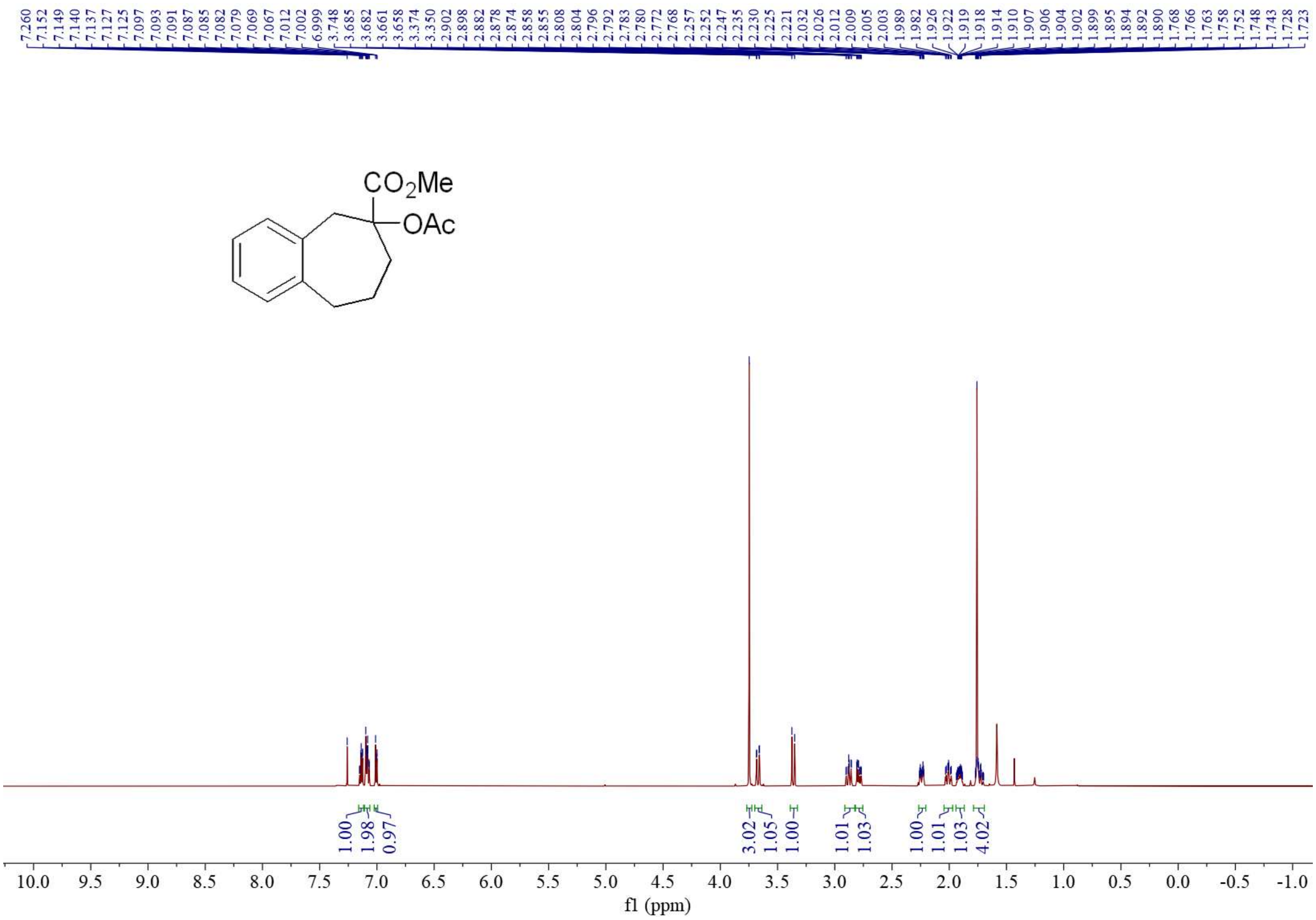

^{13}C NMR spectrum of **8o** (151 MHz, $CDCl_3$)

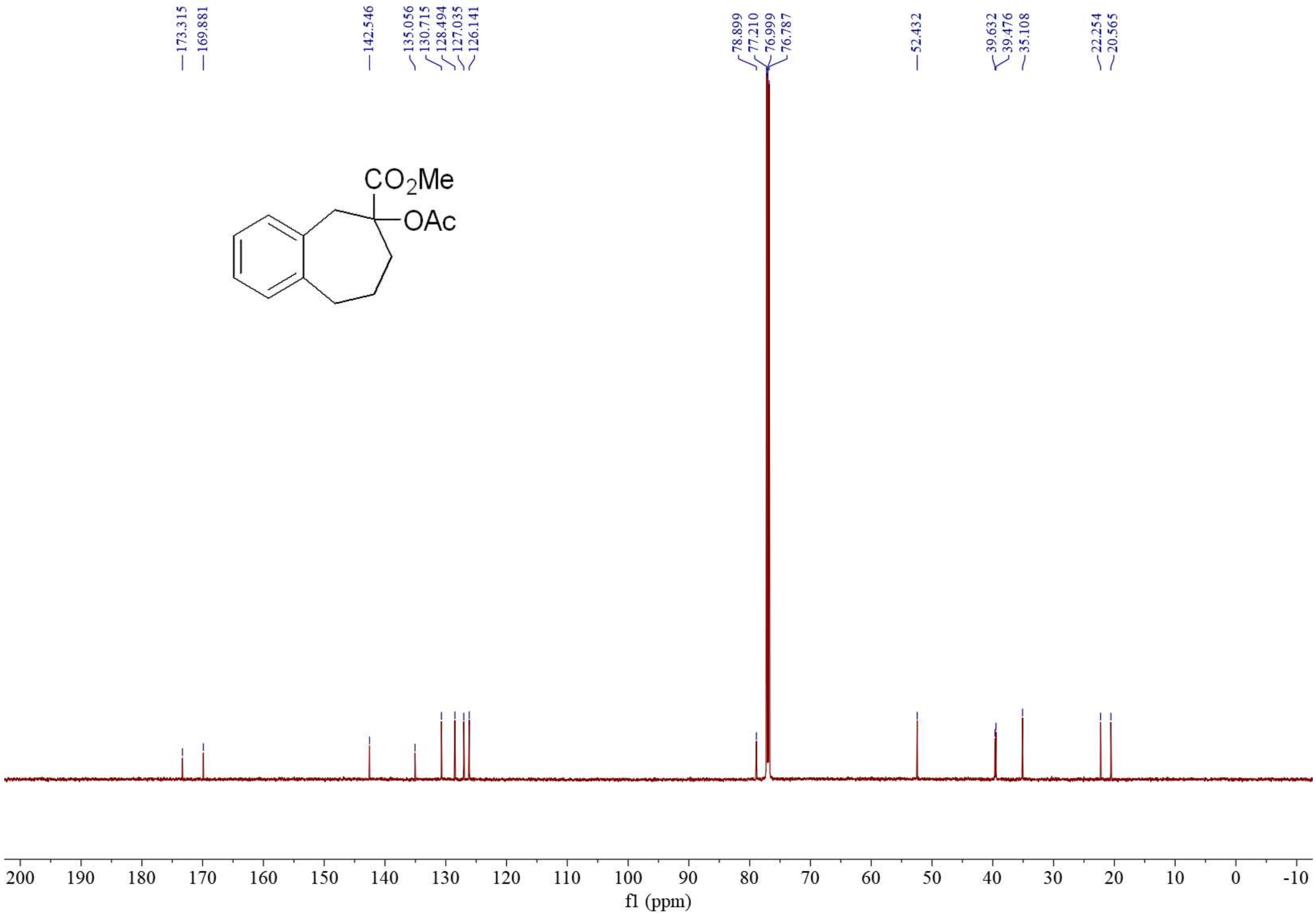

^{1}H NMR spectrum of **8p** (600 MHz, $CDCl_3$)

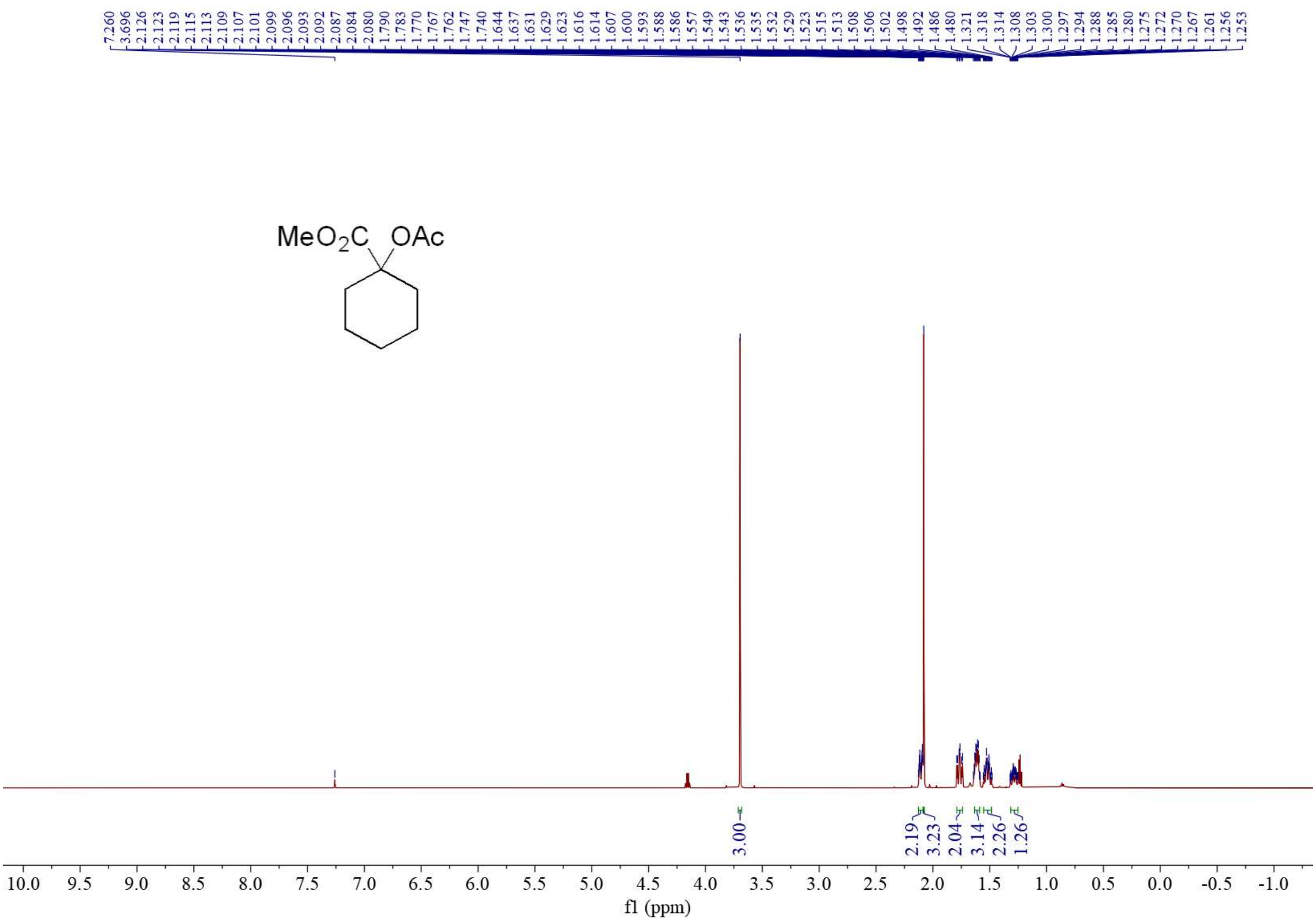

^{13}C NMR spectrum of **8p** (151 MHz, $CDCl_3$)

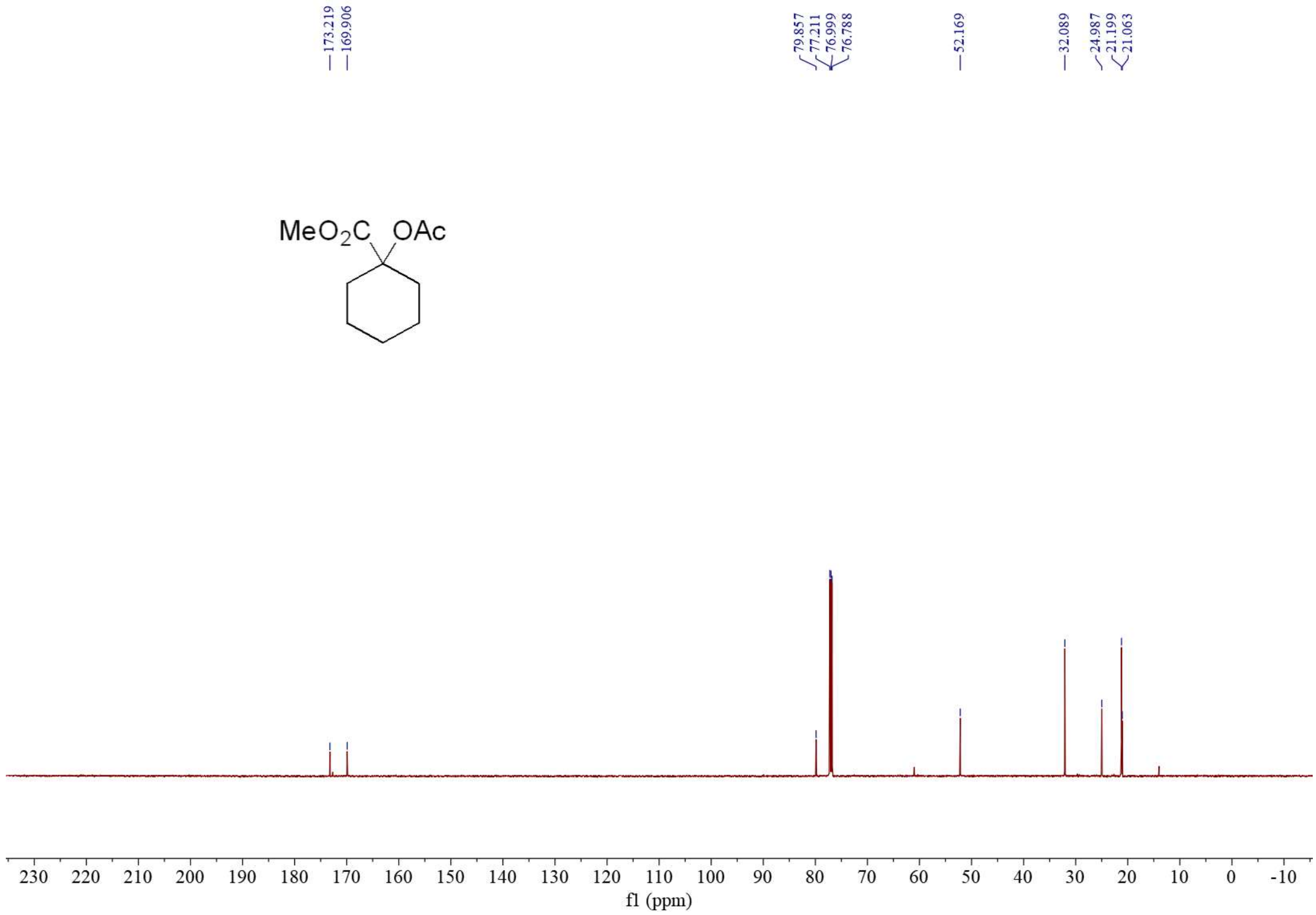

^{1}H NMR spectrum of **8q** (400 MHz, $CDCl_3$)

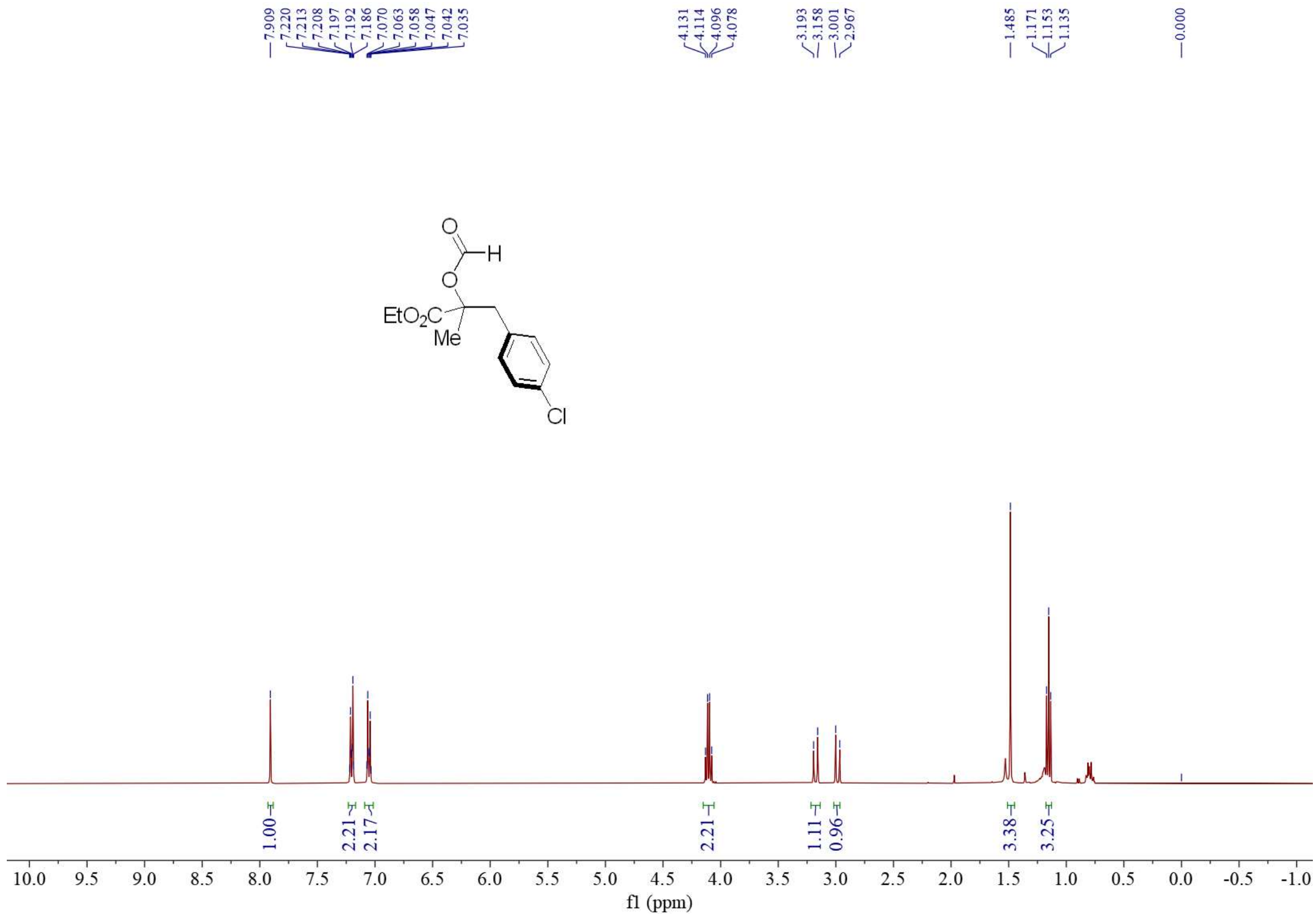

^{13}C NMR spectrum of **8q** (101 MHz, $CDCl_3$)

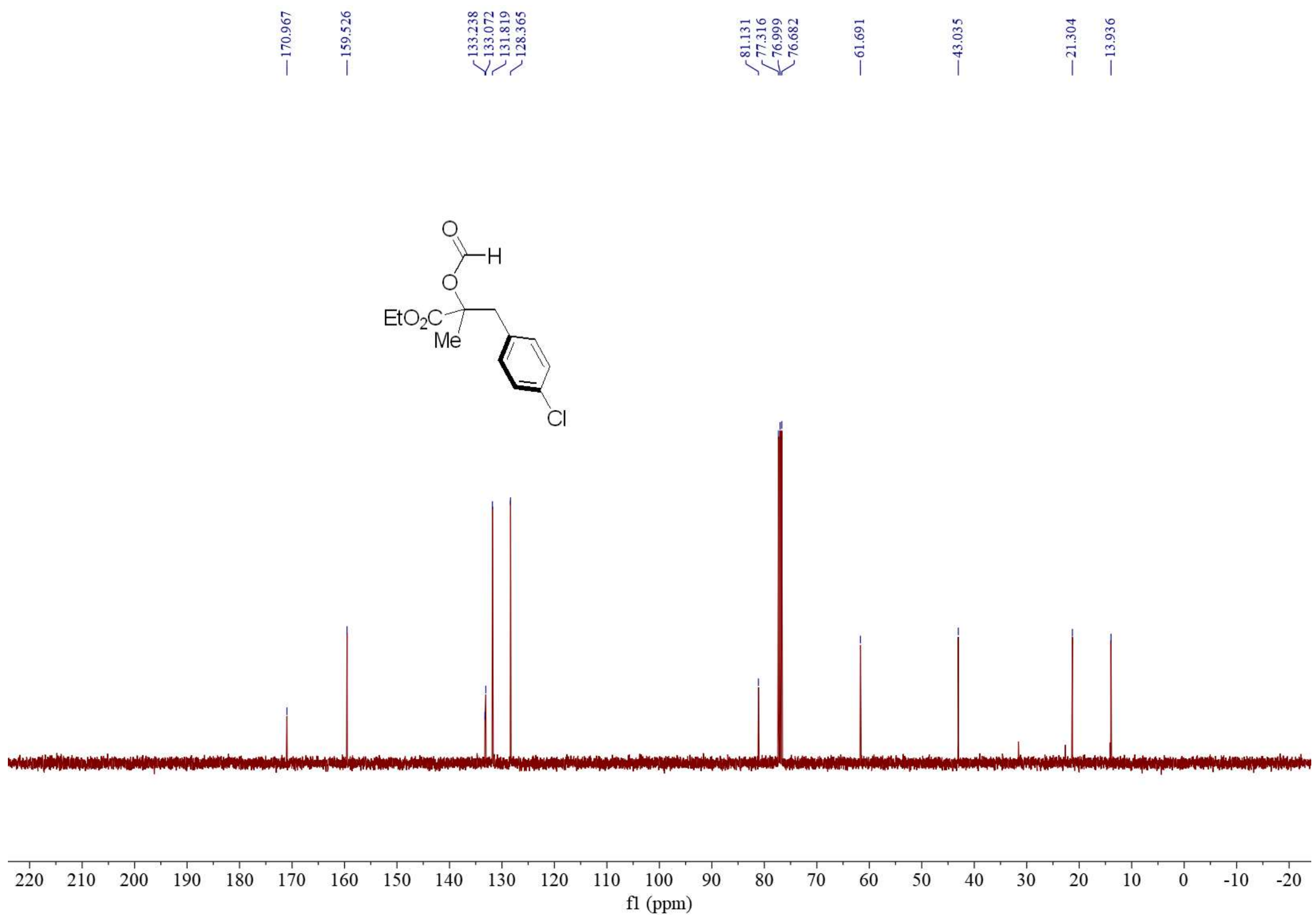

^{1}H NMR spectrum of **8r** (400 MHz, $CDCl_3$)

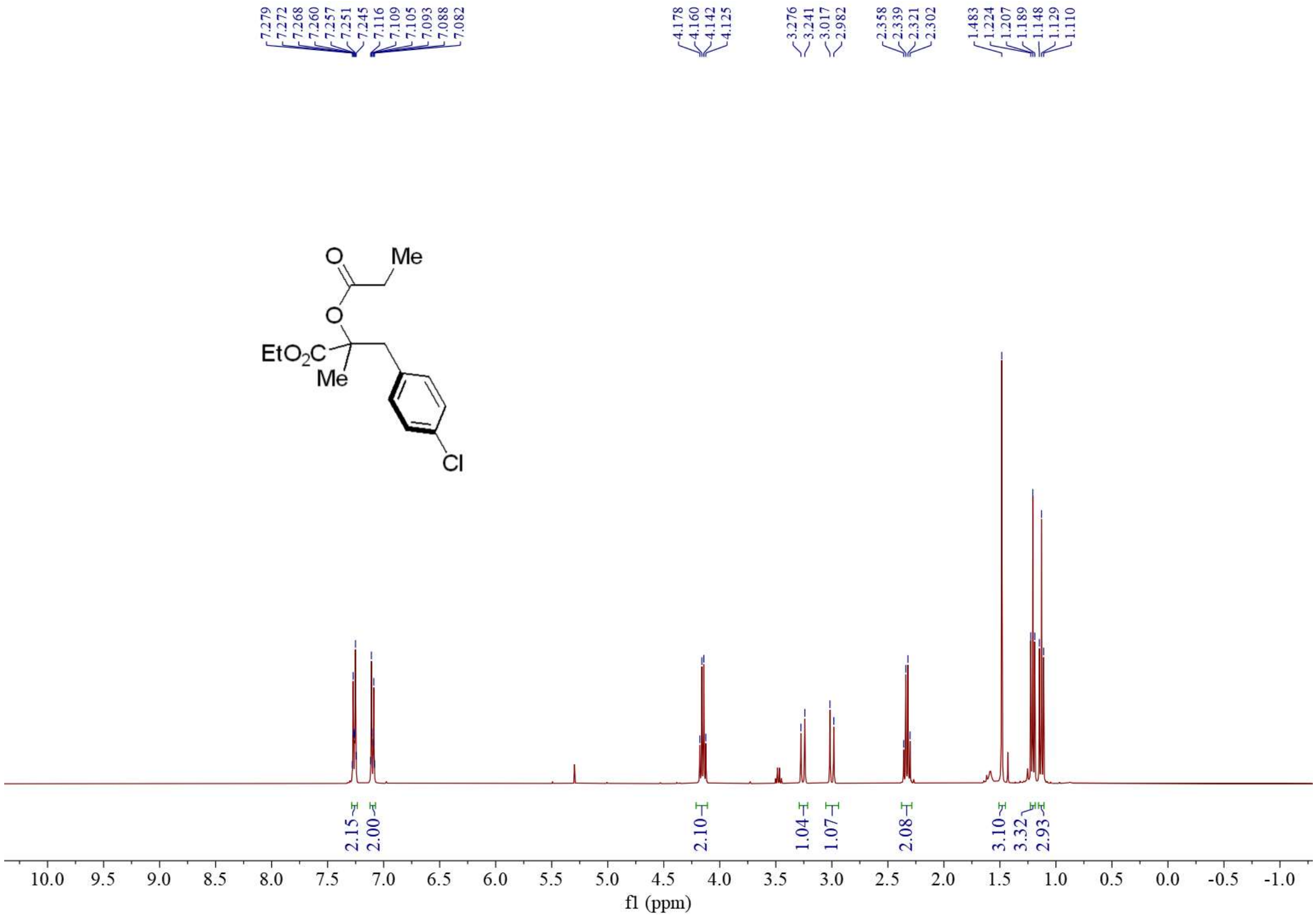

^{13}C NMR spectrum of **8r** (101 MHz, $CDCl_3$)

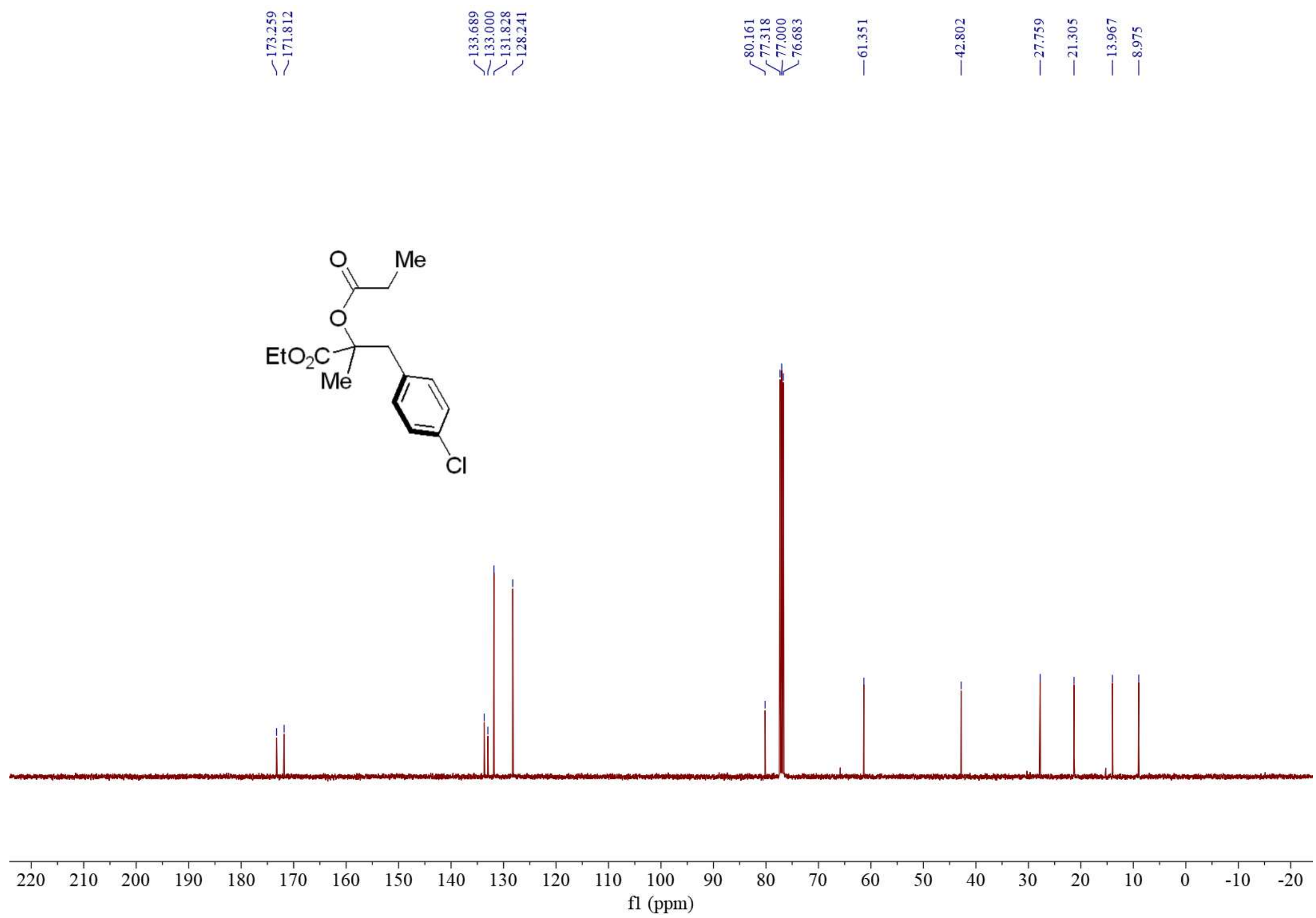

^{1}H NMR spectrum of **8s** (500 MHz, $CDCl_3$)

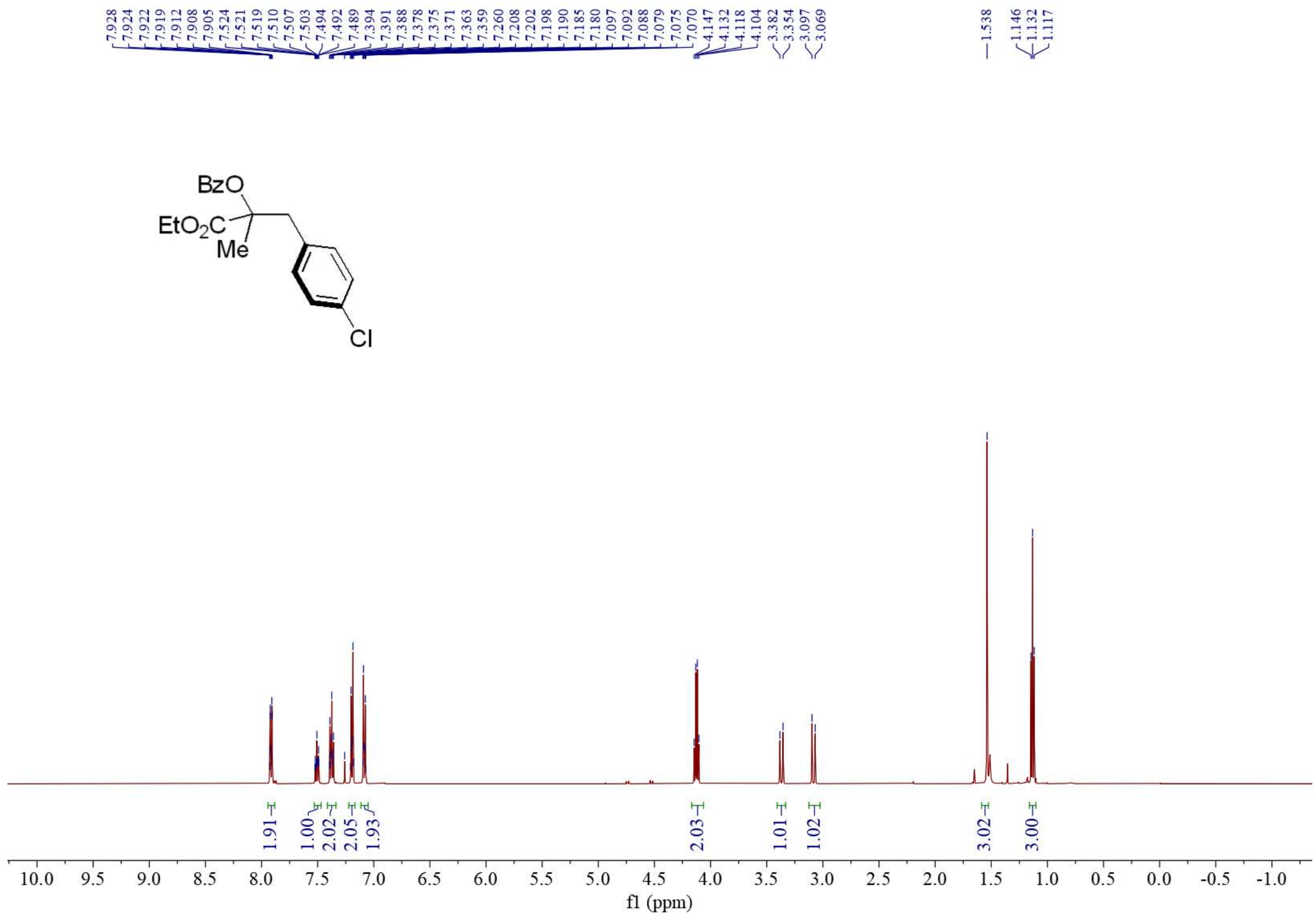

^{13}C NMR spectrum of **8s** (126 MHz, $CDCl_3$)

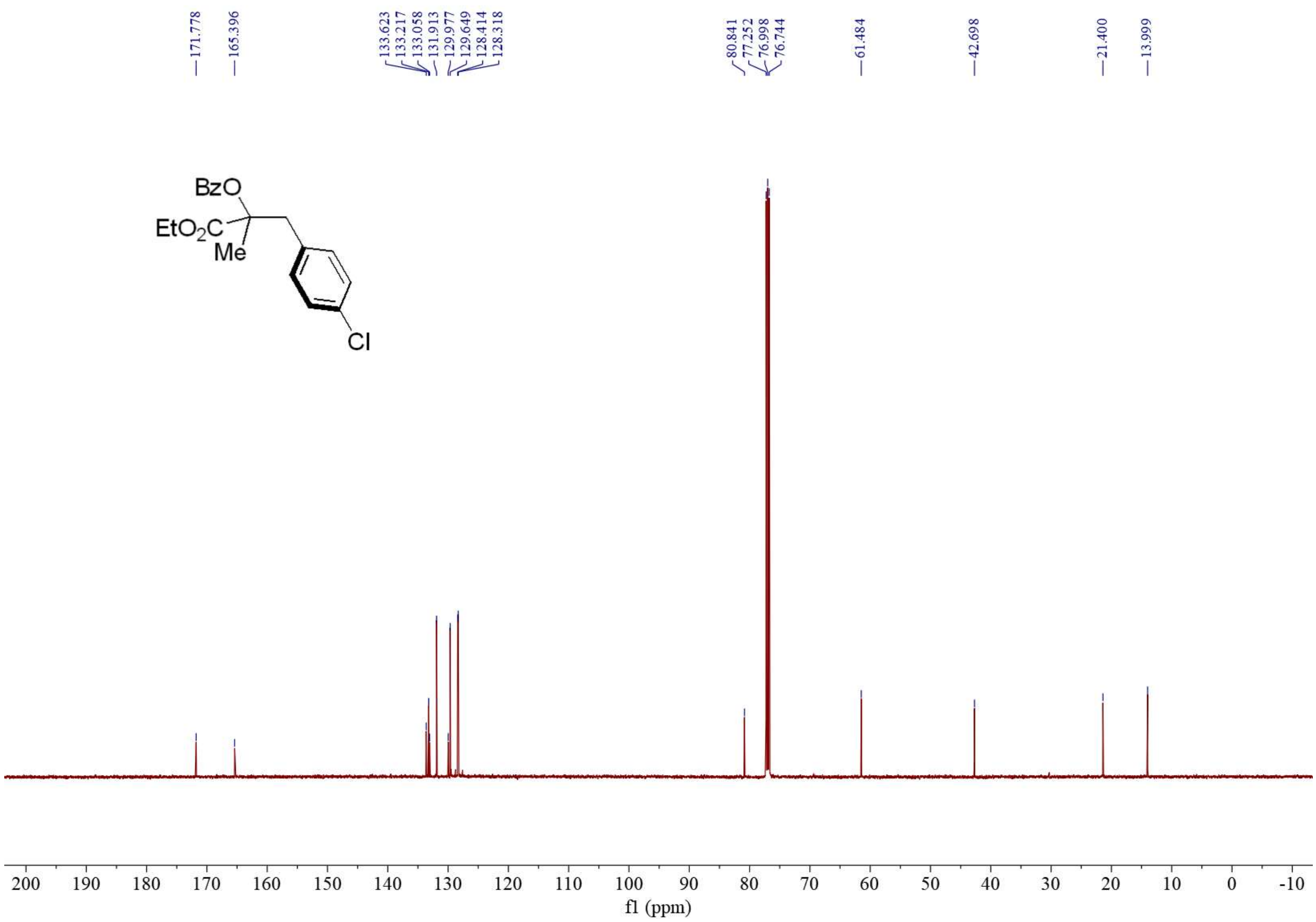

^{1}H NMR spectrum of **8t** (500 MHz, $CDCl_3$)

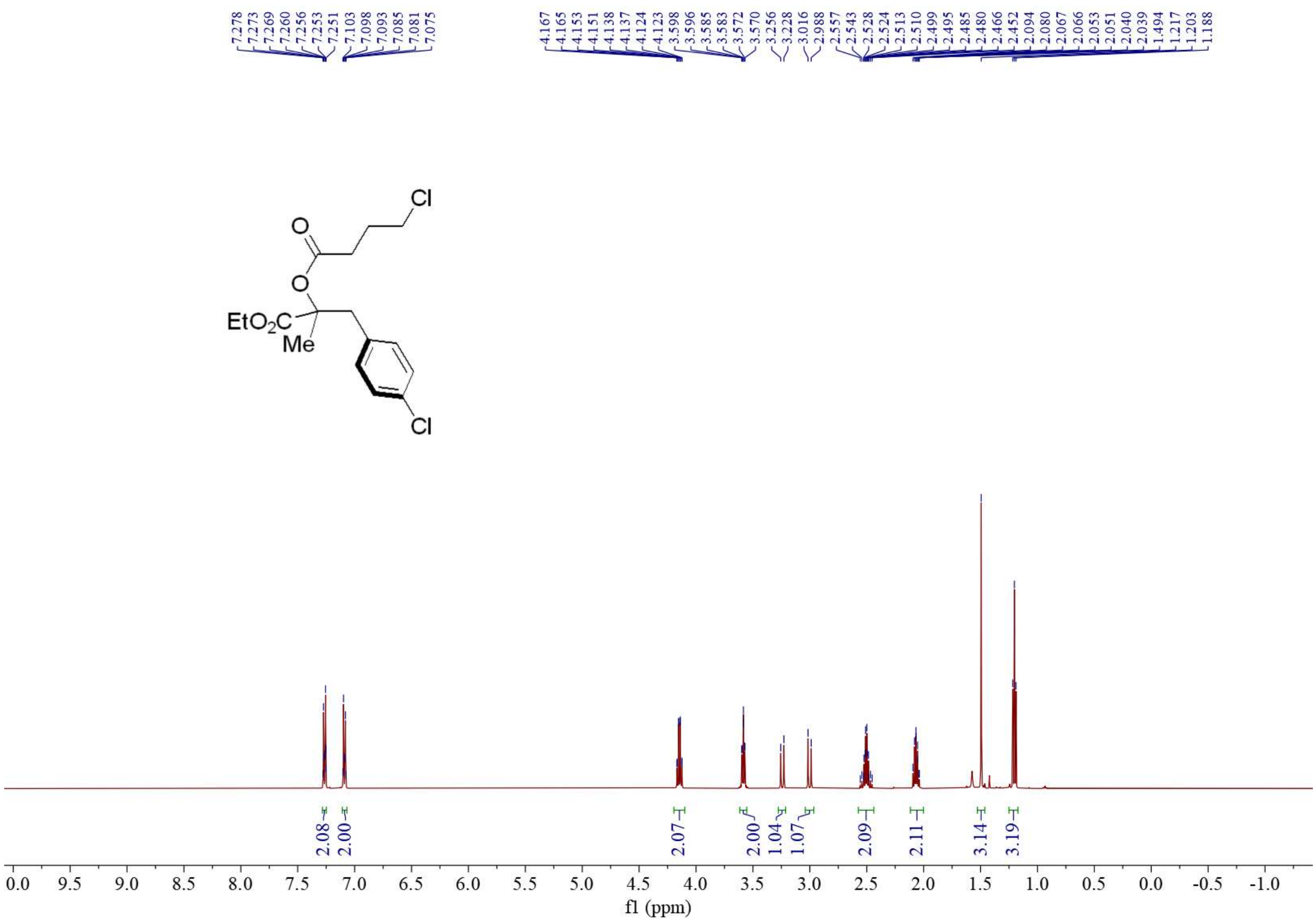

^{13}C NMR spectrum of **8t** (126 MHz, $CDCl_3$)

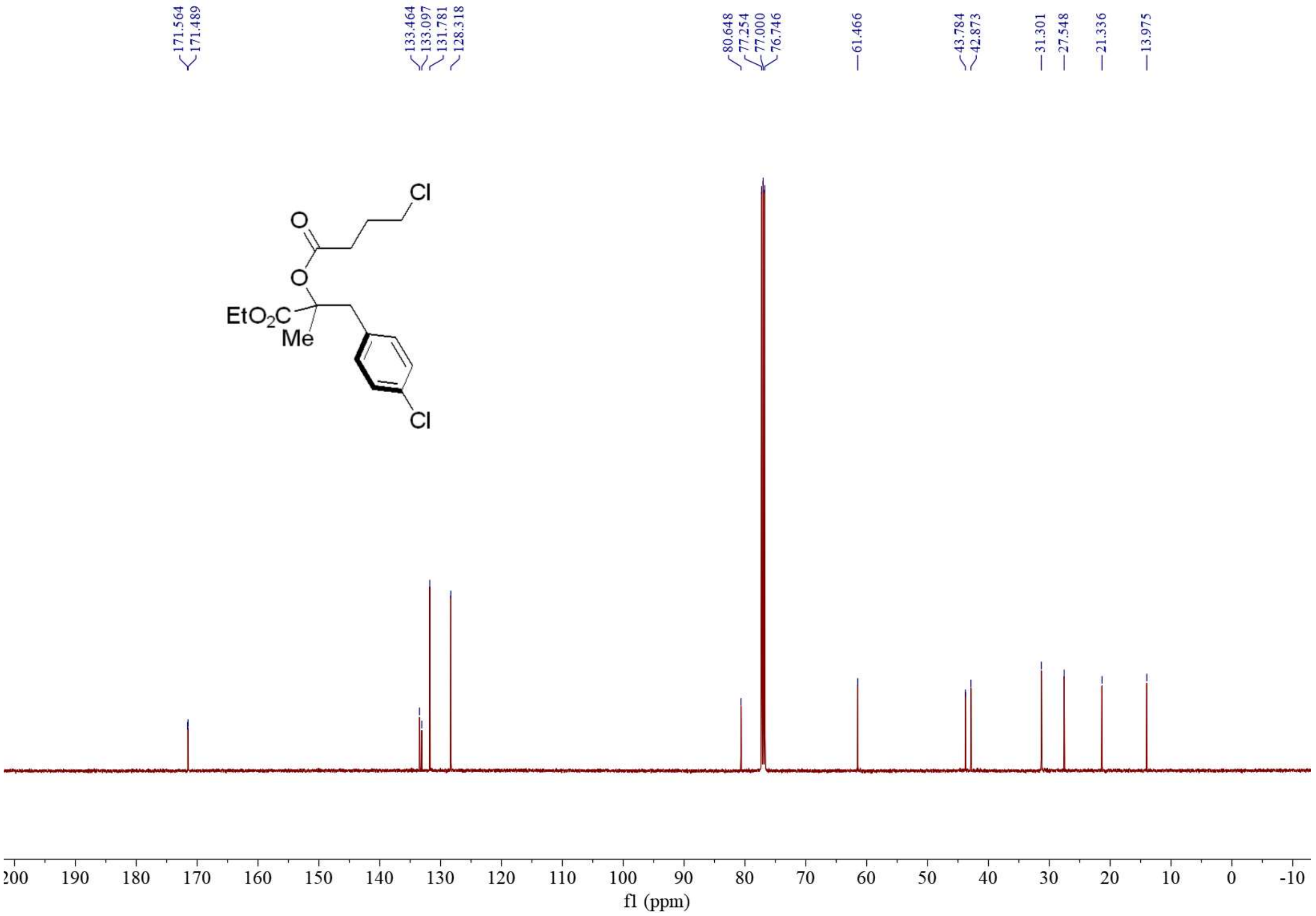

^{1}H NMR spectrum of **8u** (600 MHz, $CDCl_3$)

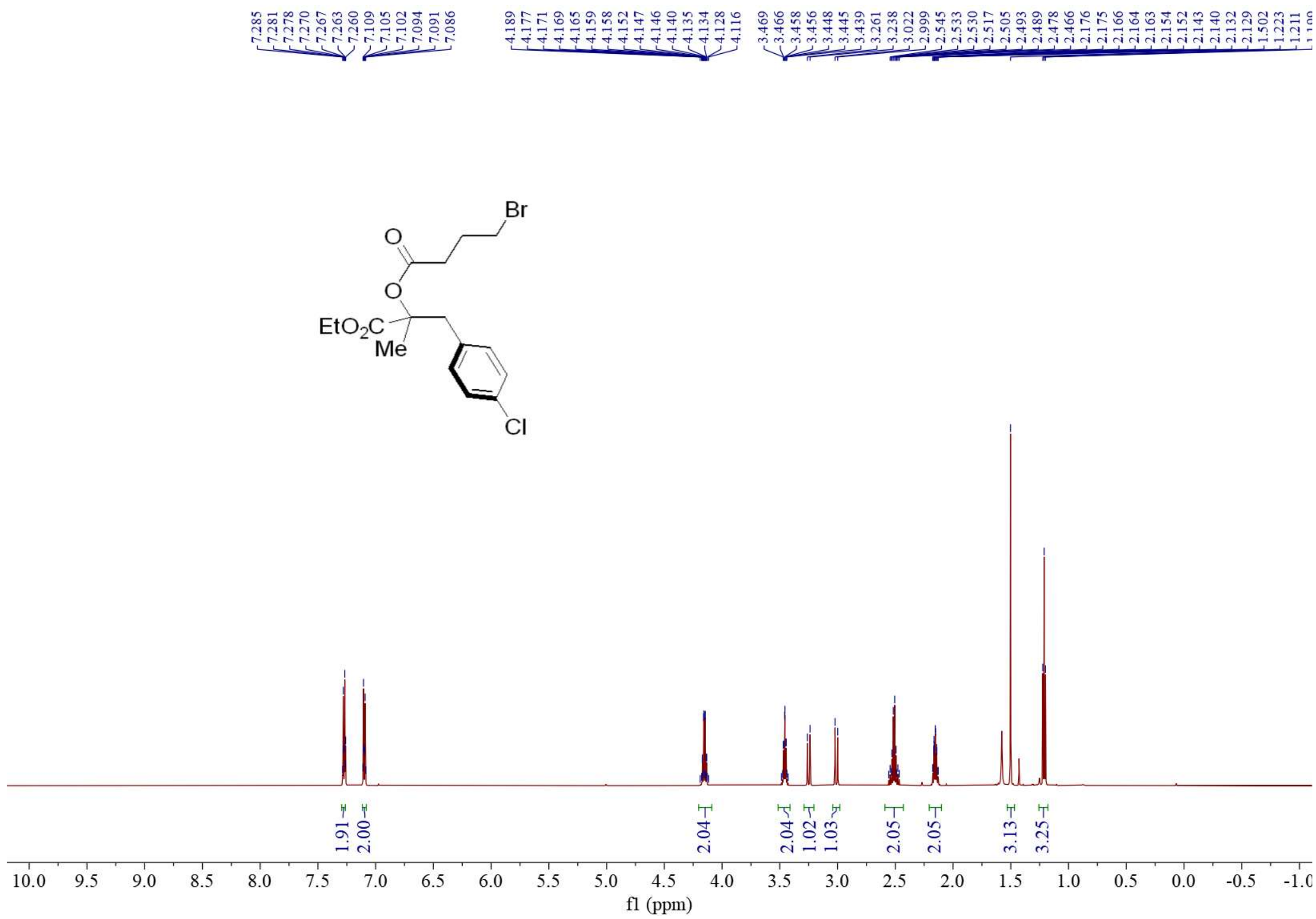

^{13}C NMR spectrum of **8u** (151 MHz, $CDCl_3$)

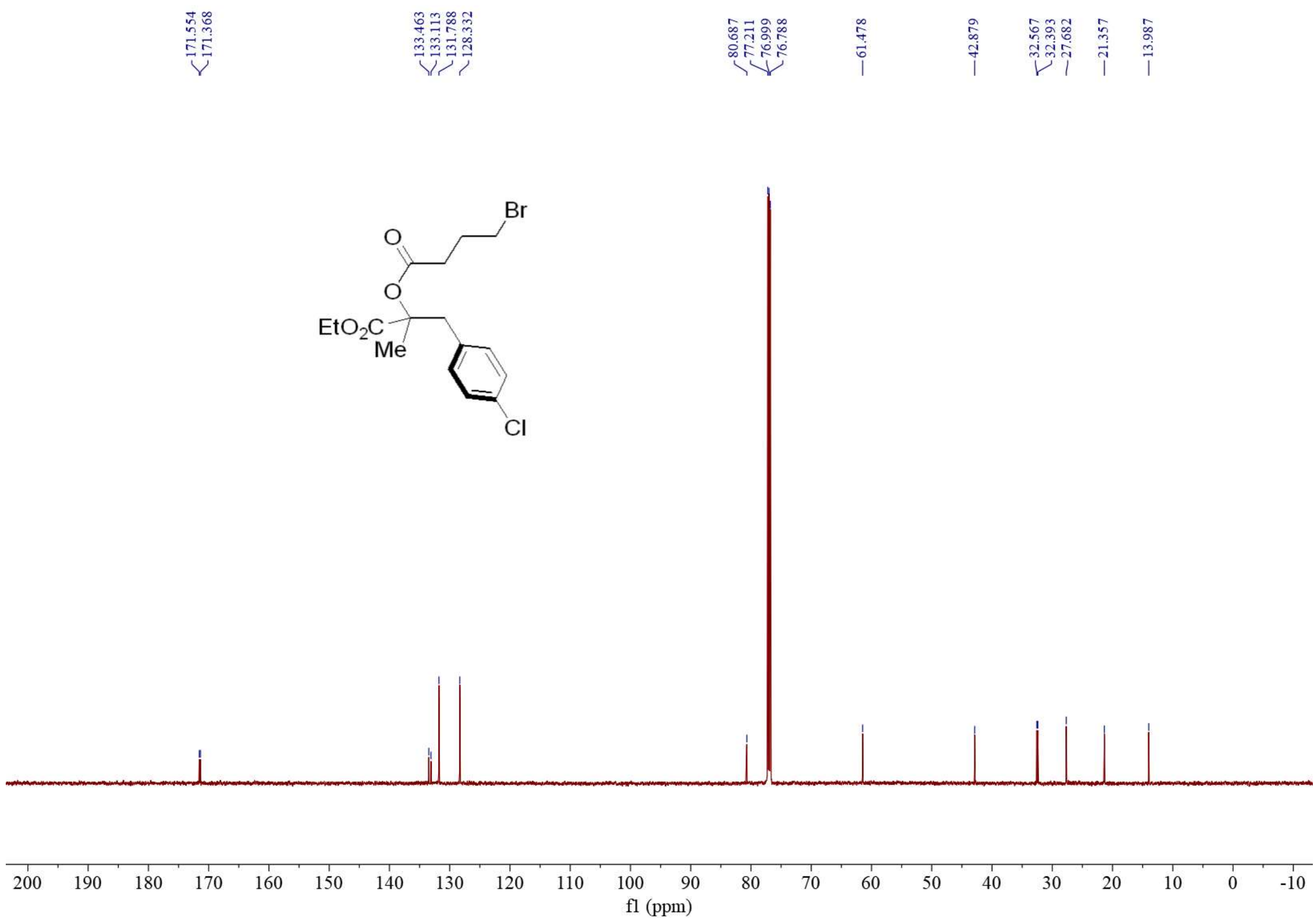

^{1}H NMR spectrum of **8v** (600 MHz, $CDCl_3$)

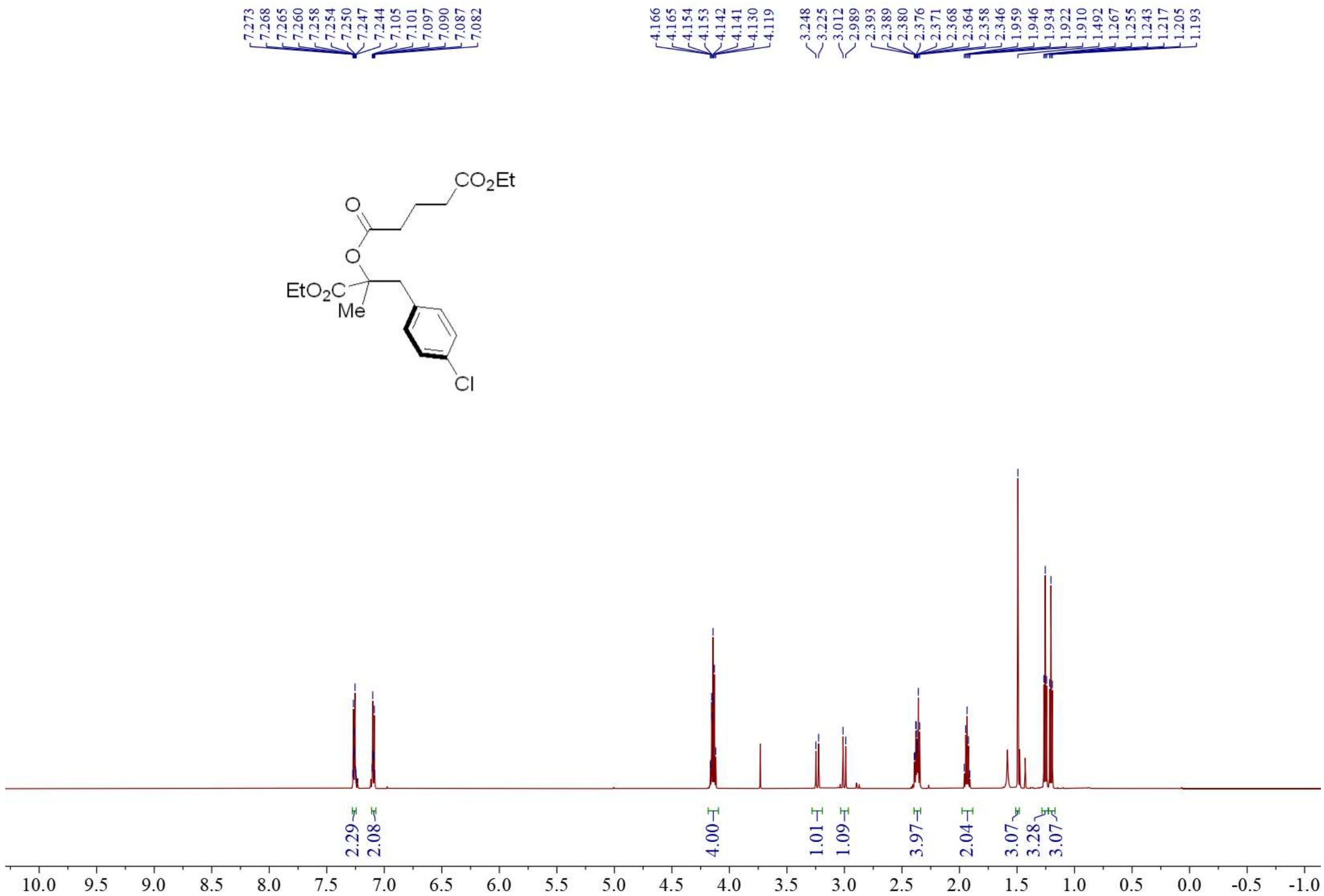

^{13}C NMR spectrum of **8v** (151 MHz, $CDCl_3$)

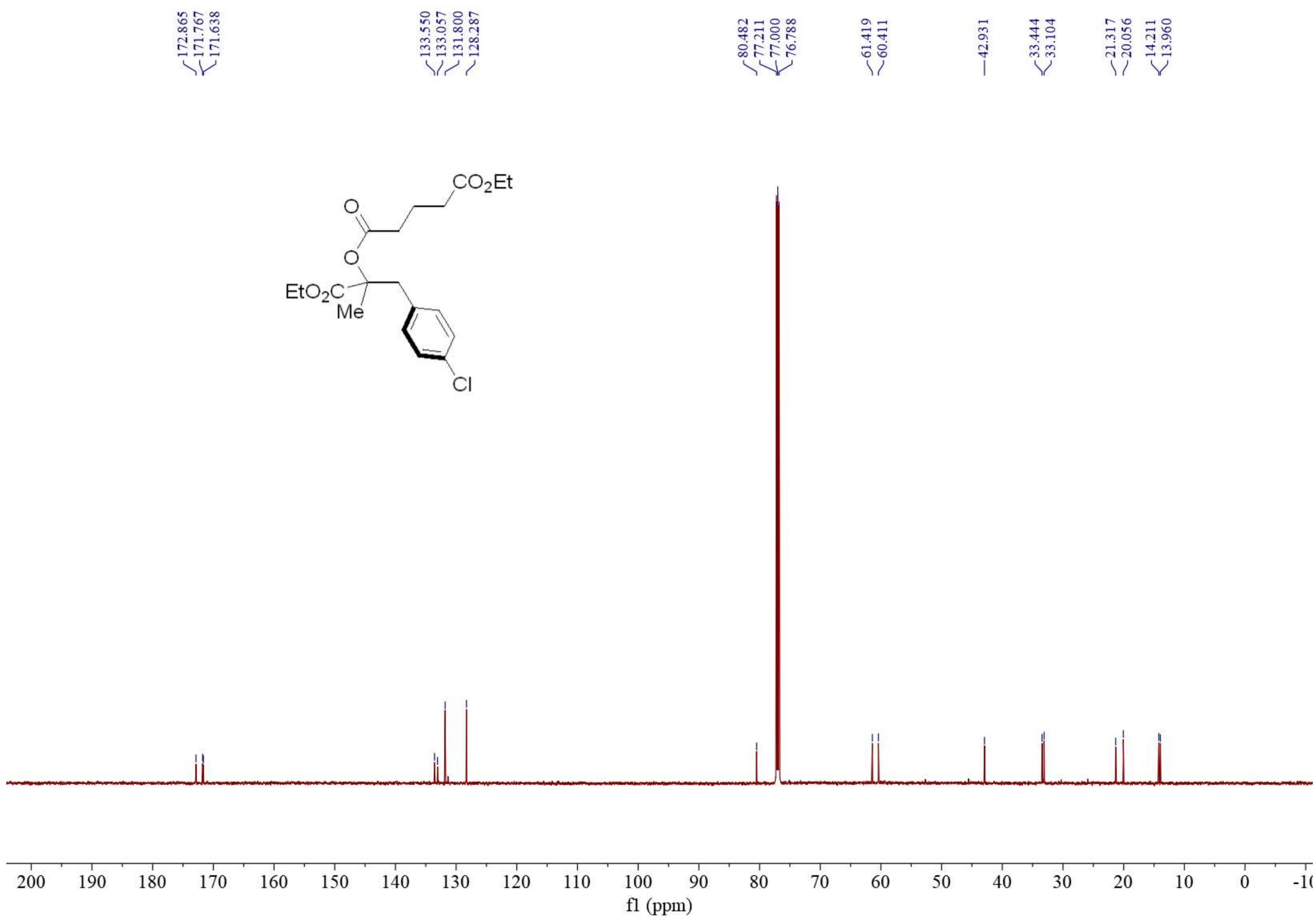

^{1}H NMR spectrum of **8w** (600 MHz, $CDCl_3$)

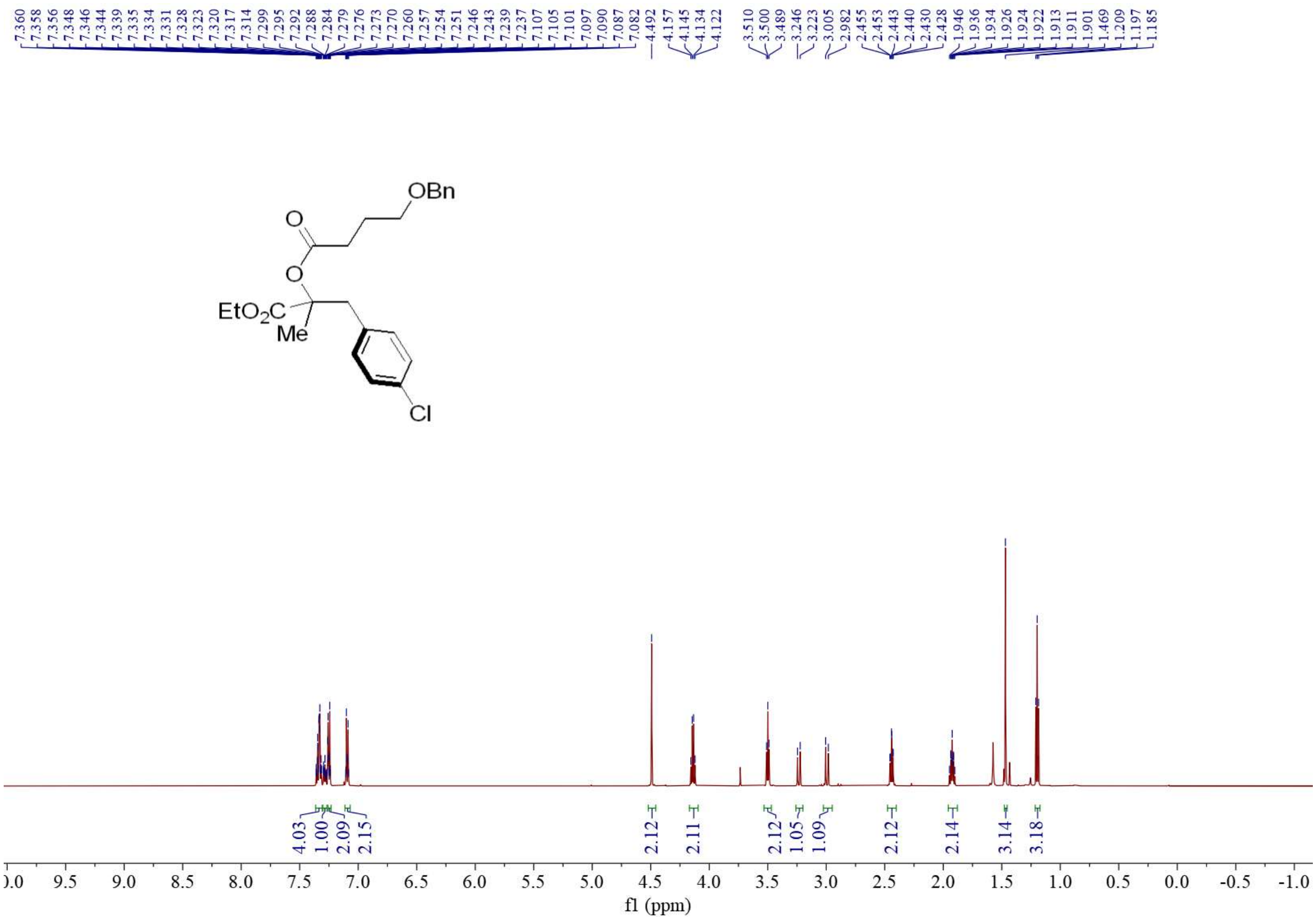

^{13}C NMR spectrum of **8w** (151 MHz, $CDCl_3$)

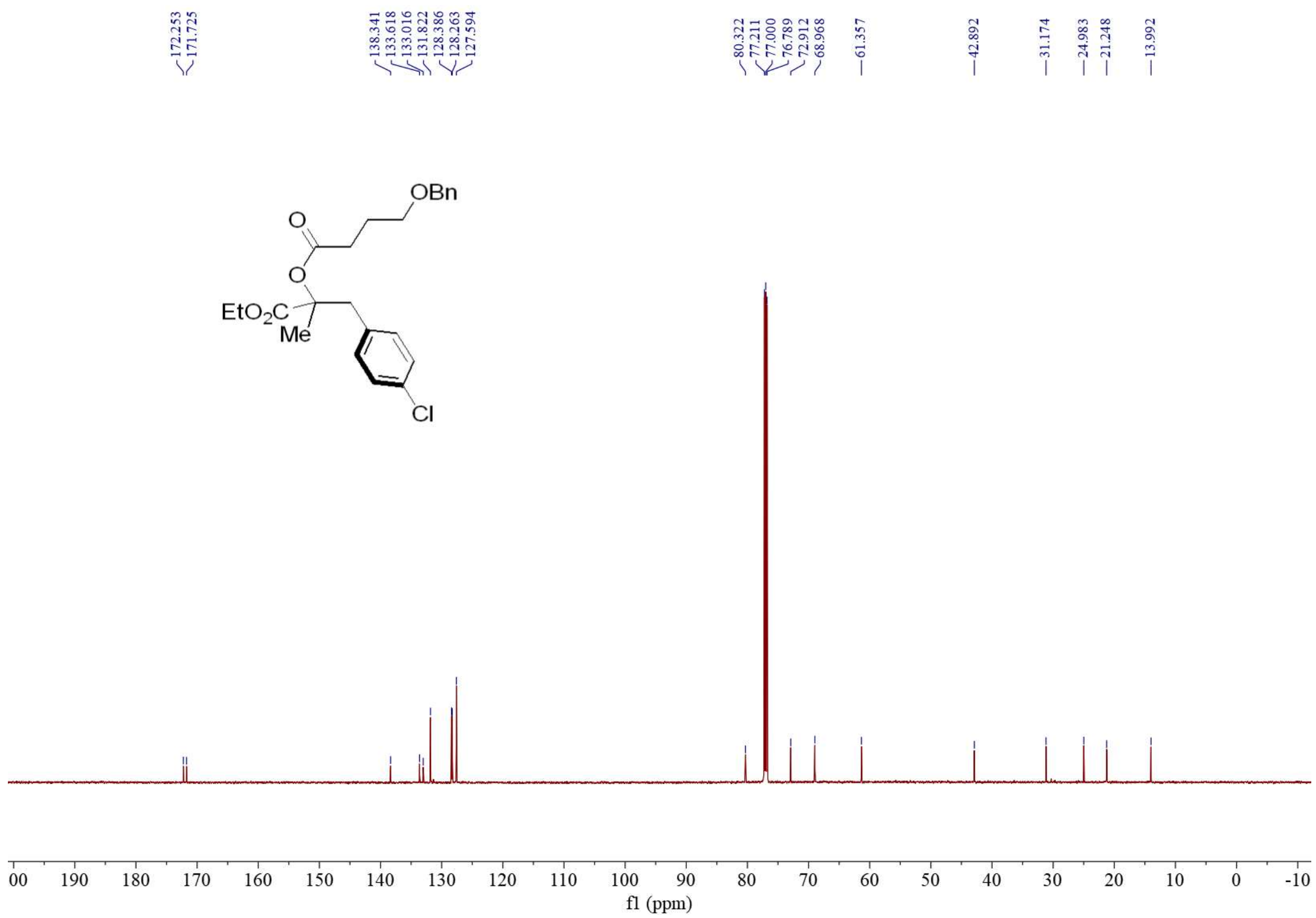

^1H NMR spectrum of **8x** (600 MHz, $CDCl_3$)

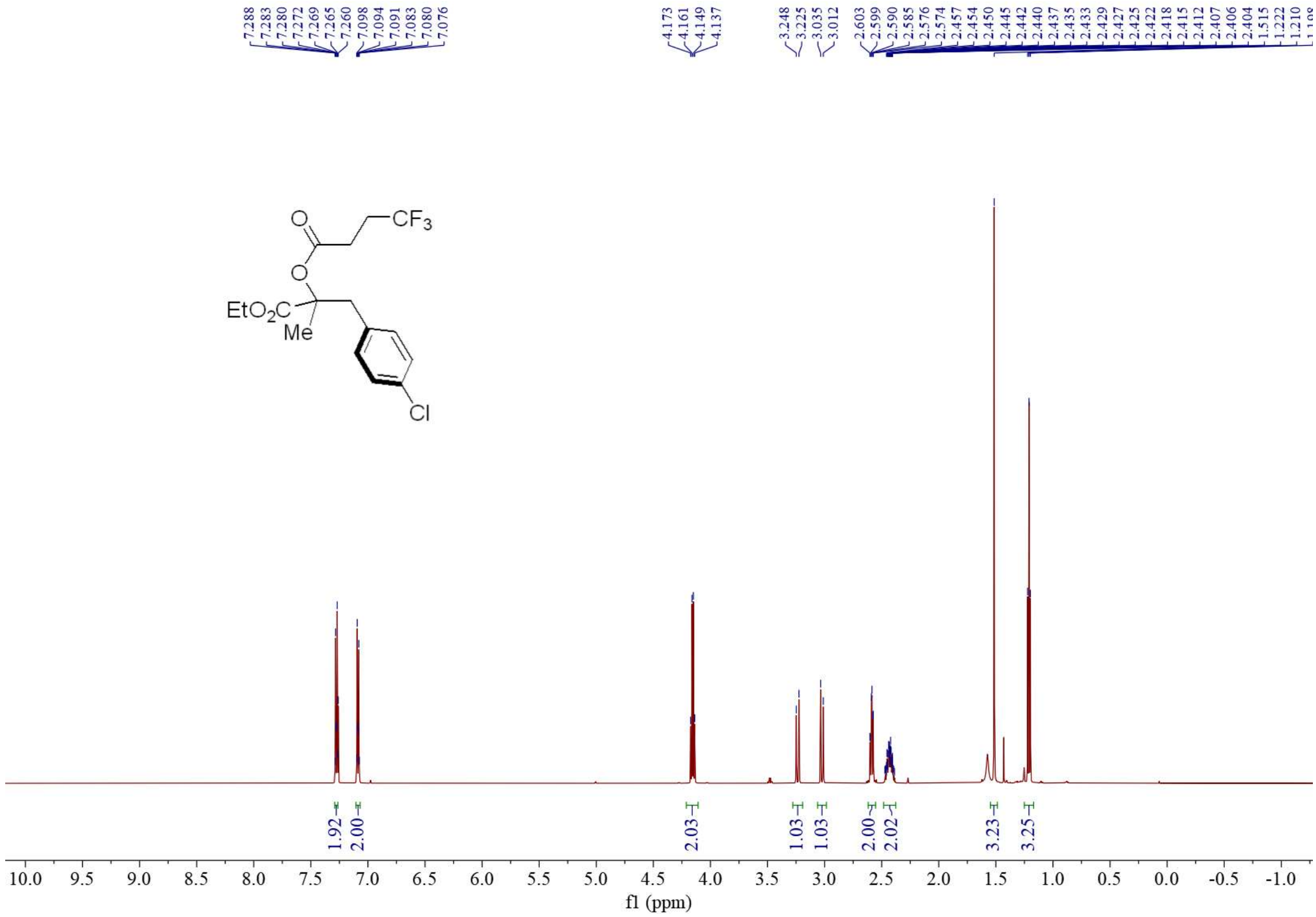

^{13}C NMR spectrum of **8x** (151 MHz, $CDCl_3$)

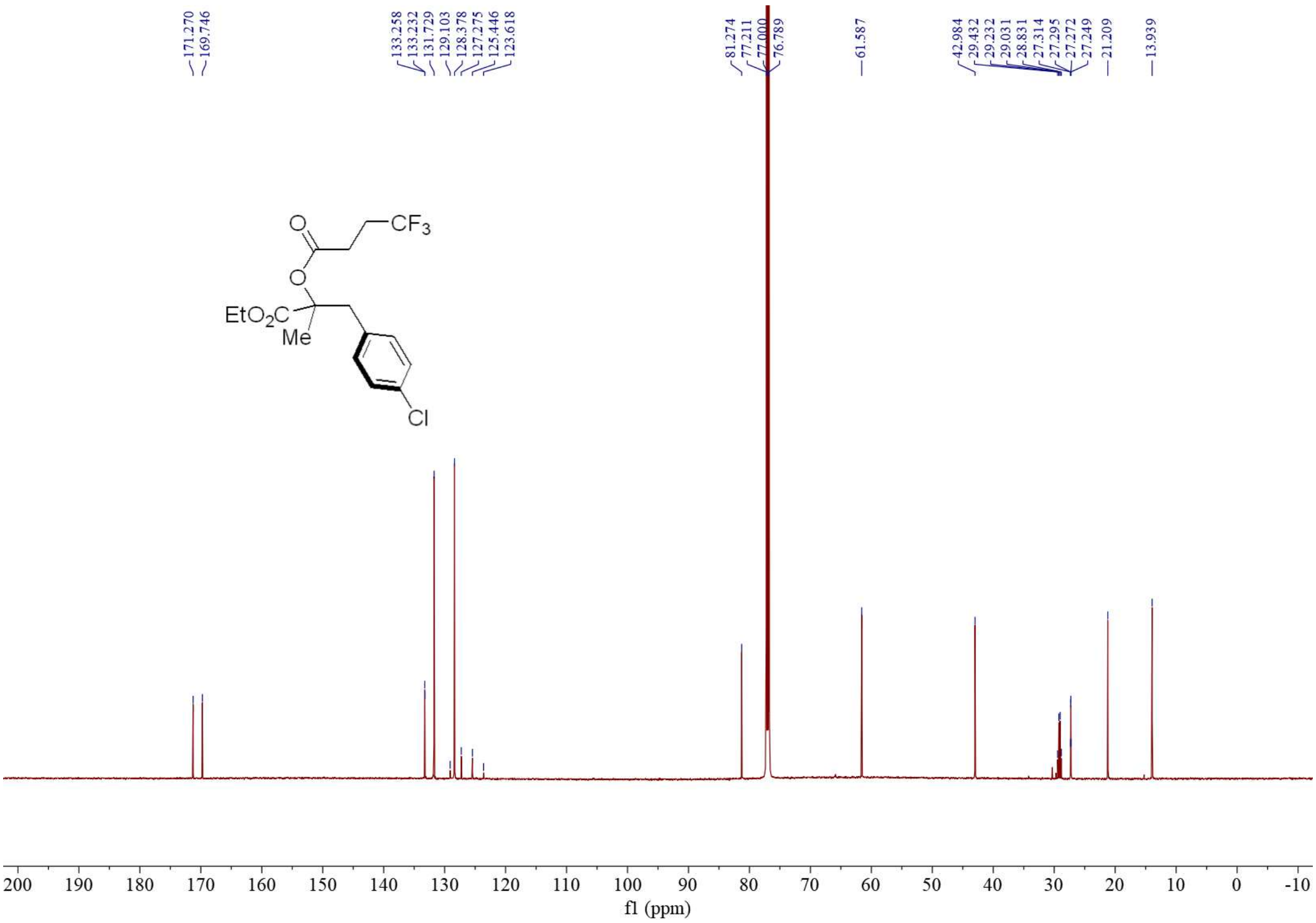

$^{19}F\{^1H\}$ NMR spectrum of **8x** (377 MHz, $CDCl_3$)

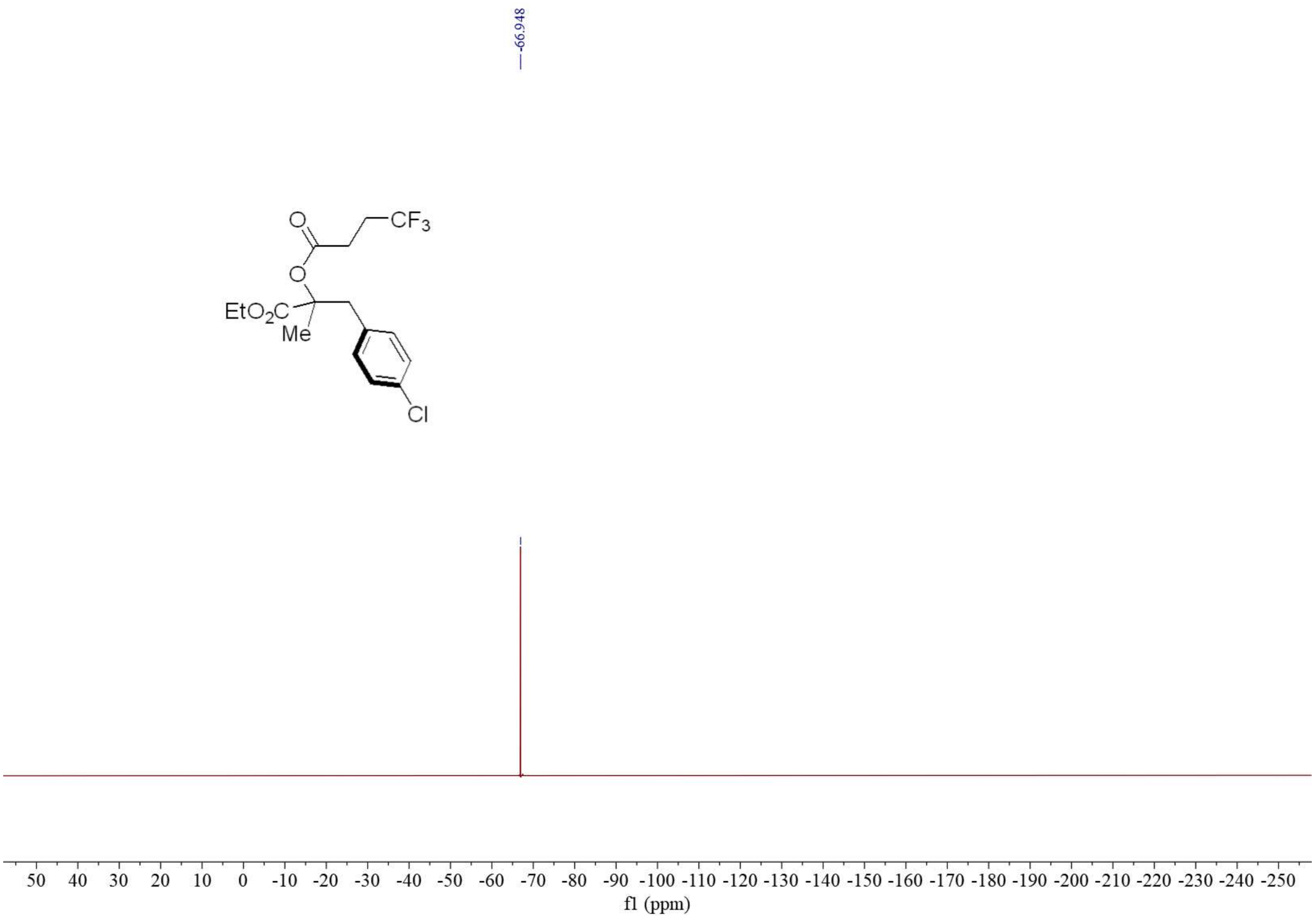

^{19}F NMR spectrum of **8x** (377 MHz, $CDCl_3$)

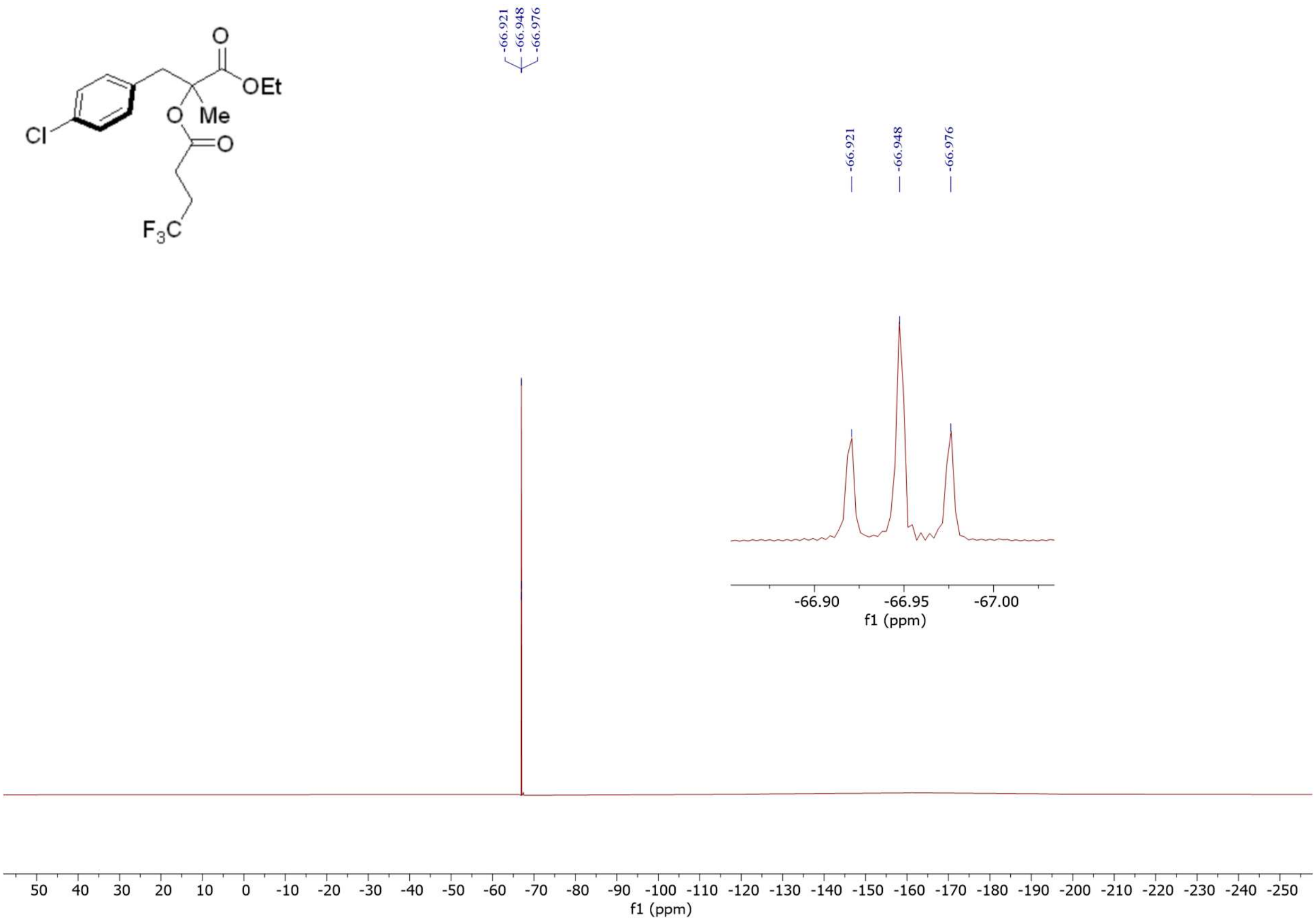

^{1}H NMR spectrum of **8y** (600 MHz, $CDCl_3$)

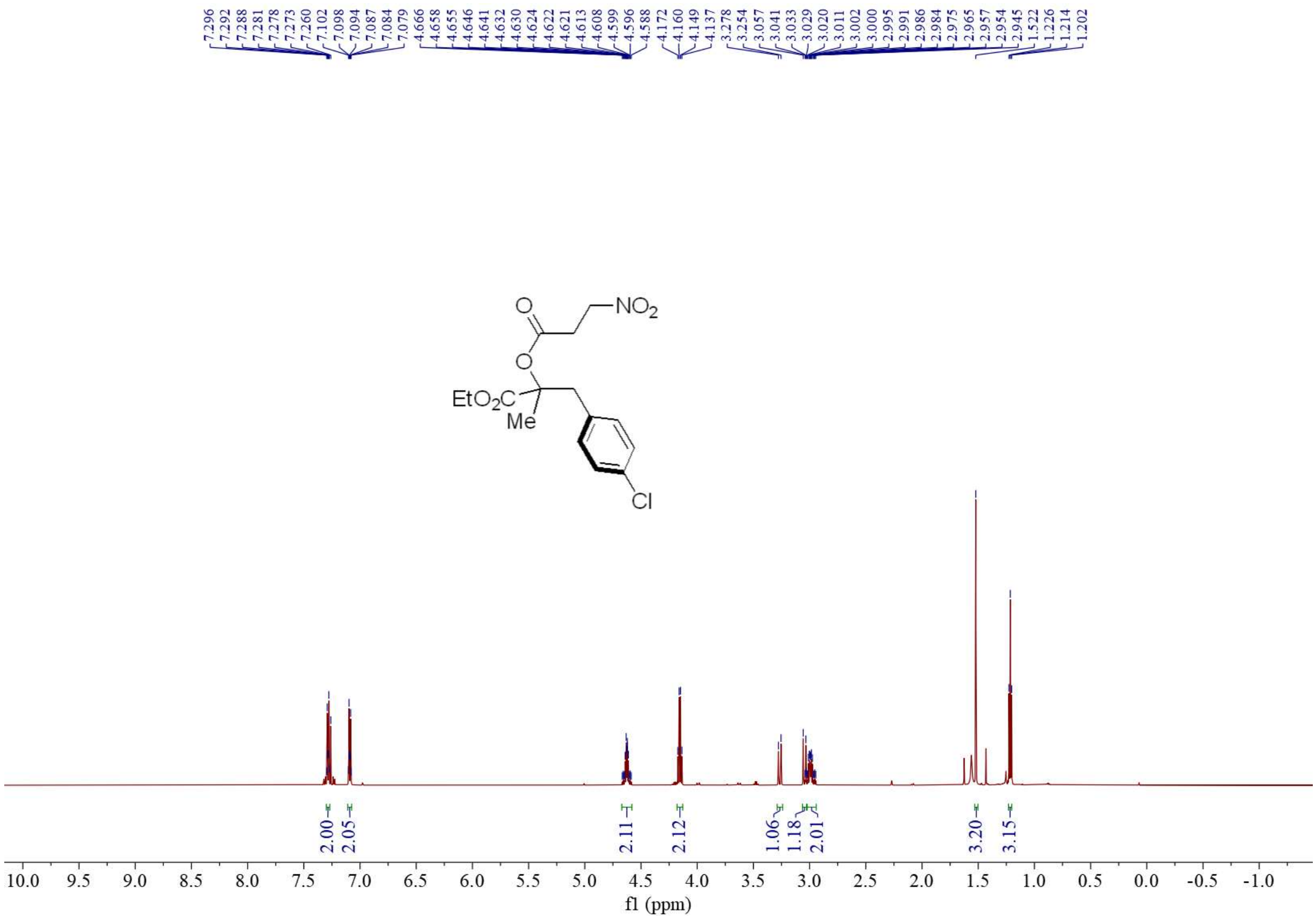

^{13}C NMR spectrum of **8y** (151 MHz, $CDCl_3$)

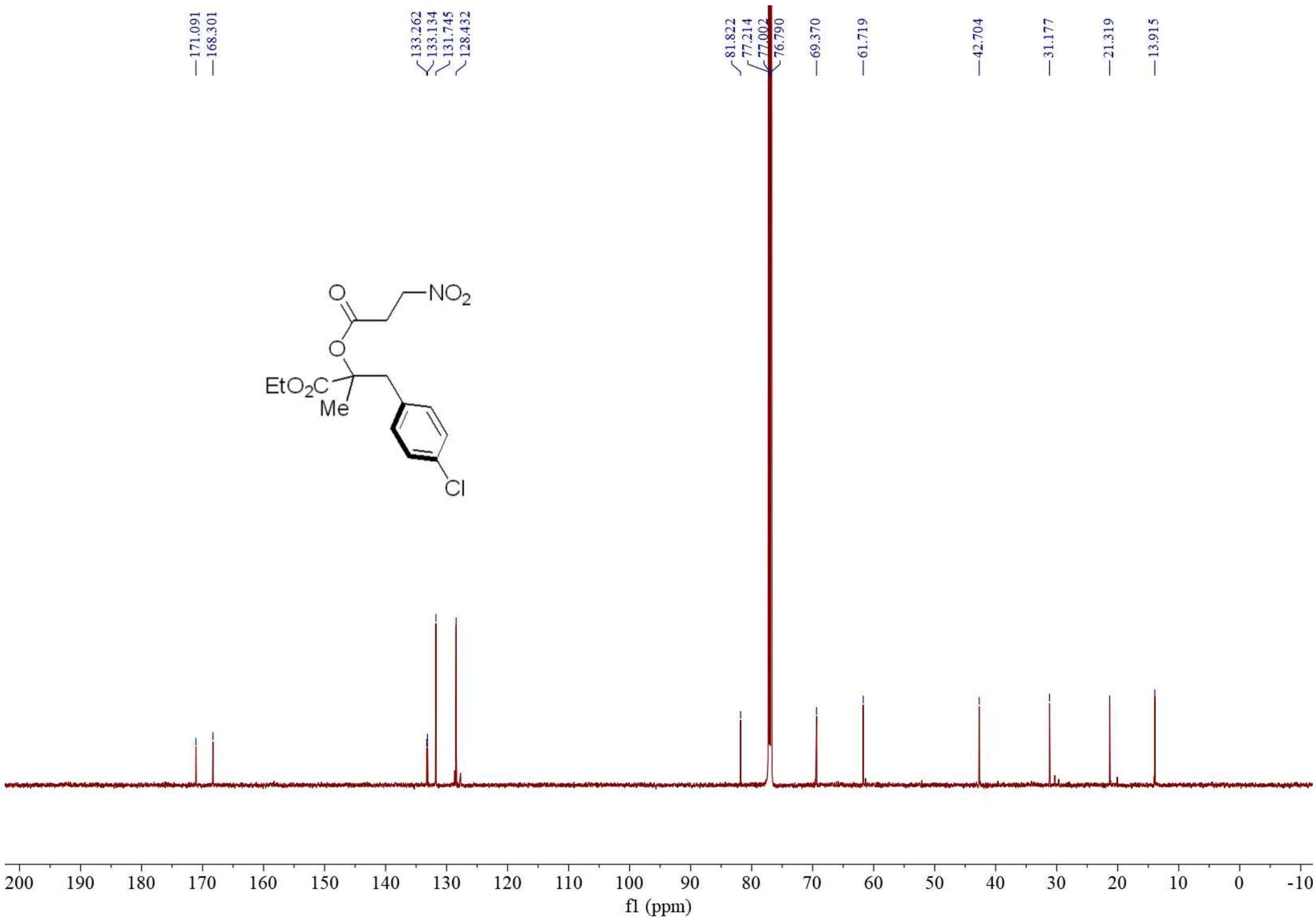

^{1}H NMR spectrum of **8z** (600 MHz, $CDCl_3$)

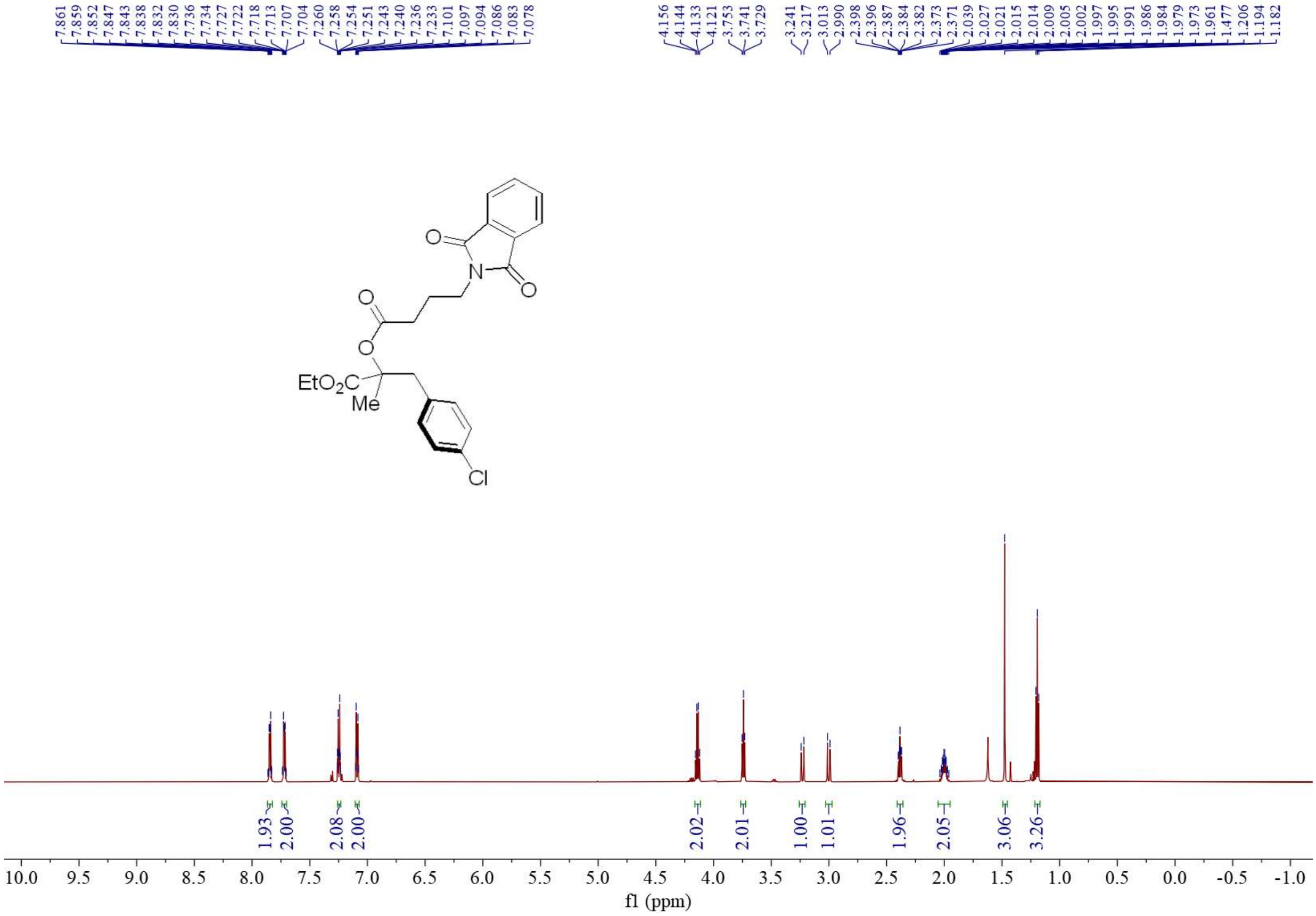

^{13}C NMR spectrum of **8z** (151 MHz, $CDCl_3$)

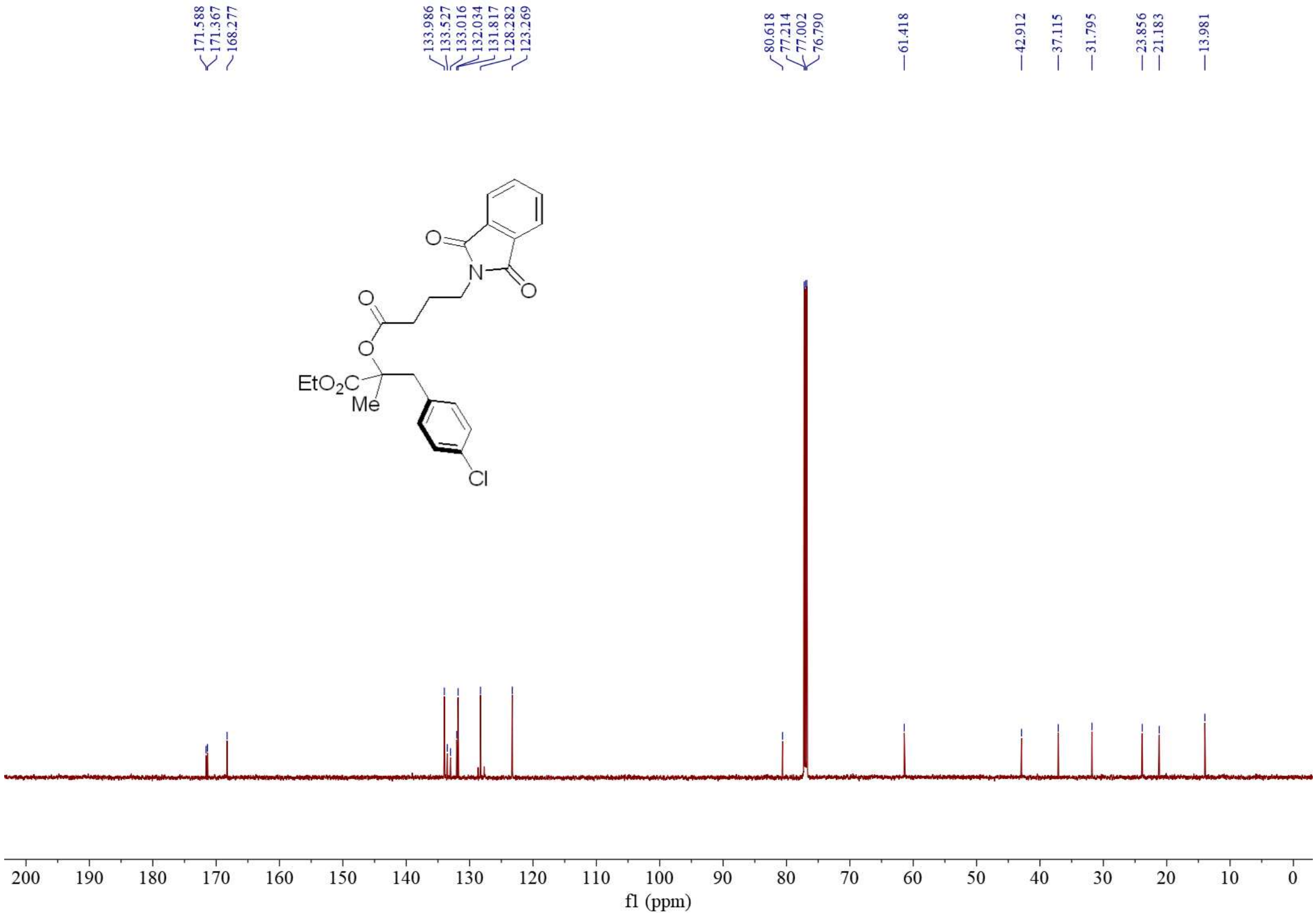

^{1}H NMR spectrum of **8aa** (400 MHz, $CDCl_3$)

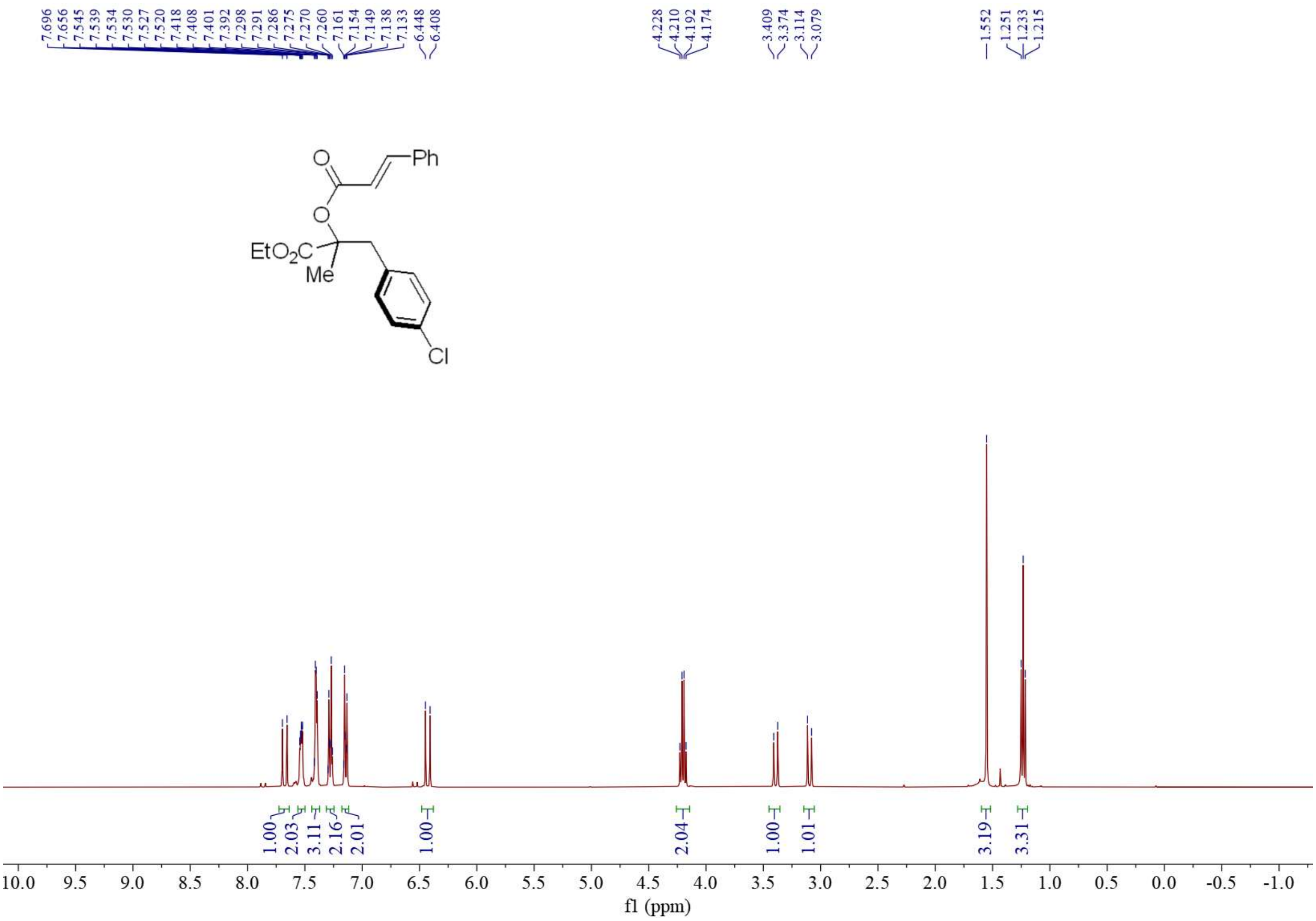

^{13}C NMR spectrum of **8aa** (101 MHz, $CDCl_3$)

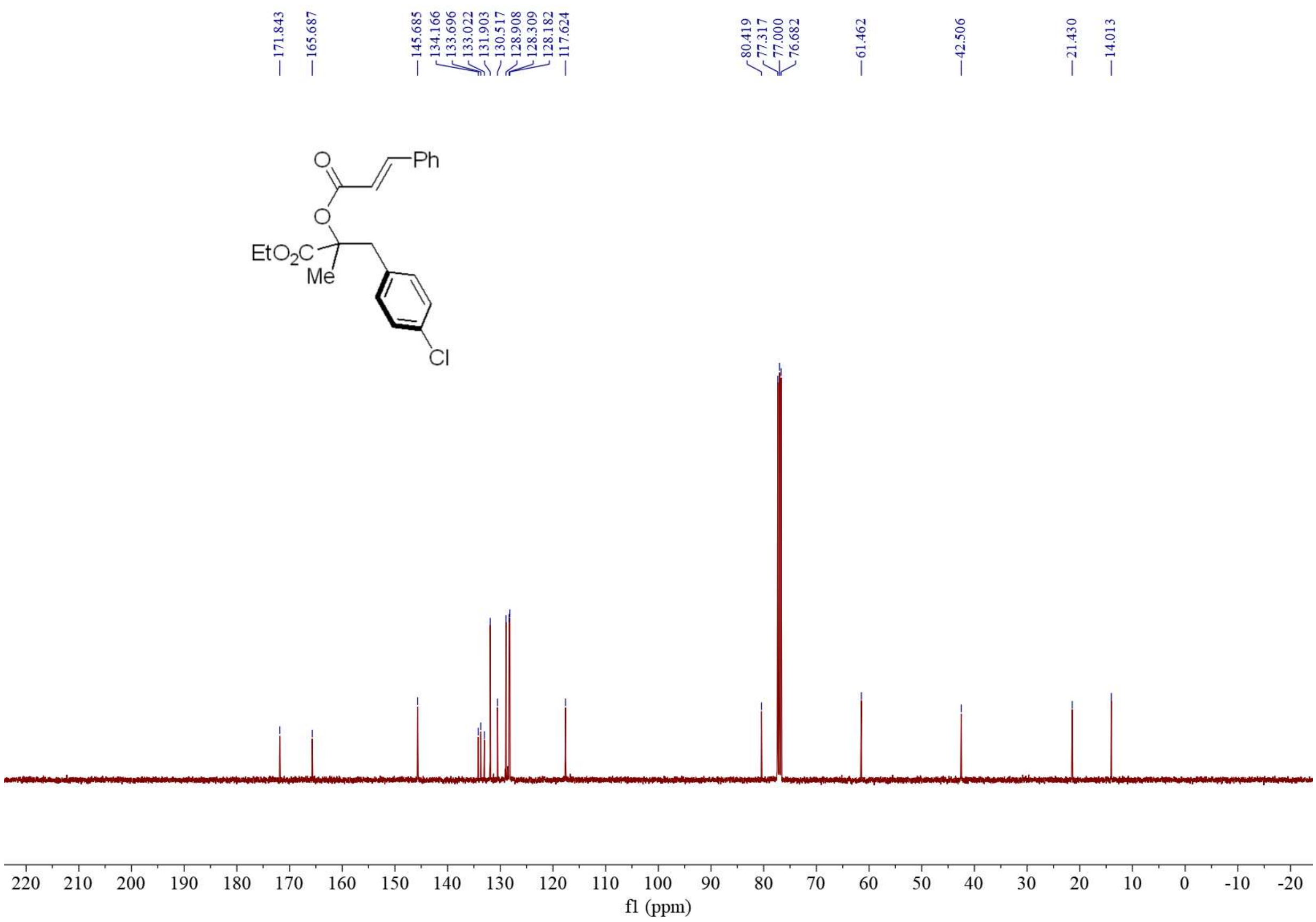

^{1}H NMR spectrum of **8ab** (600 MHz, $CDCl_3$)

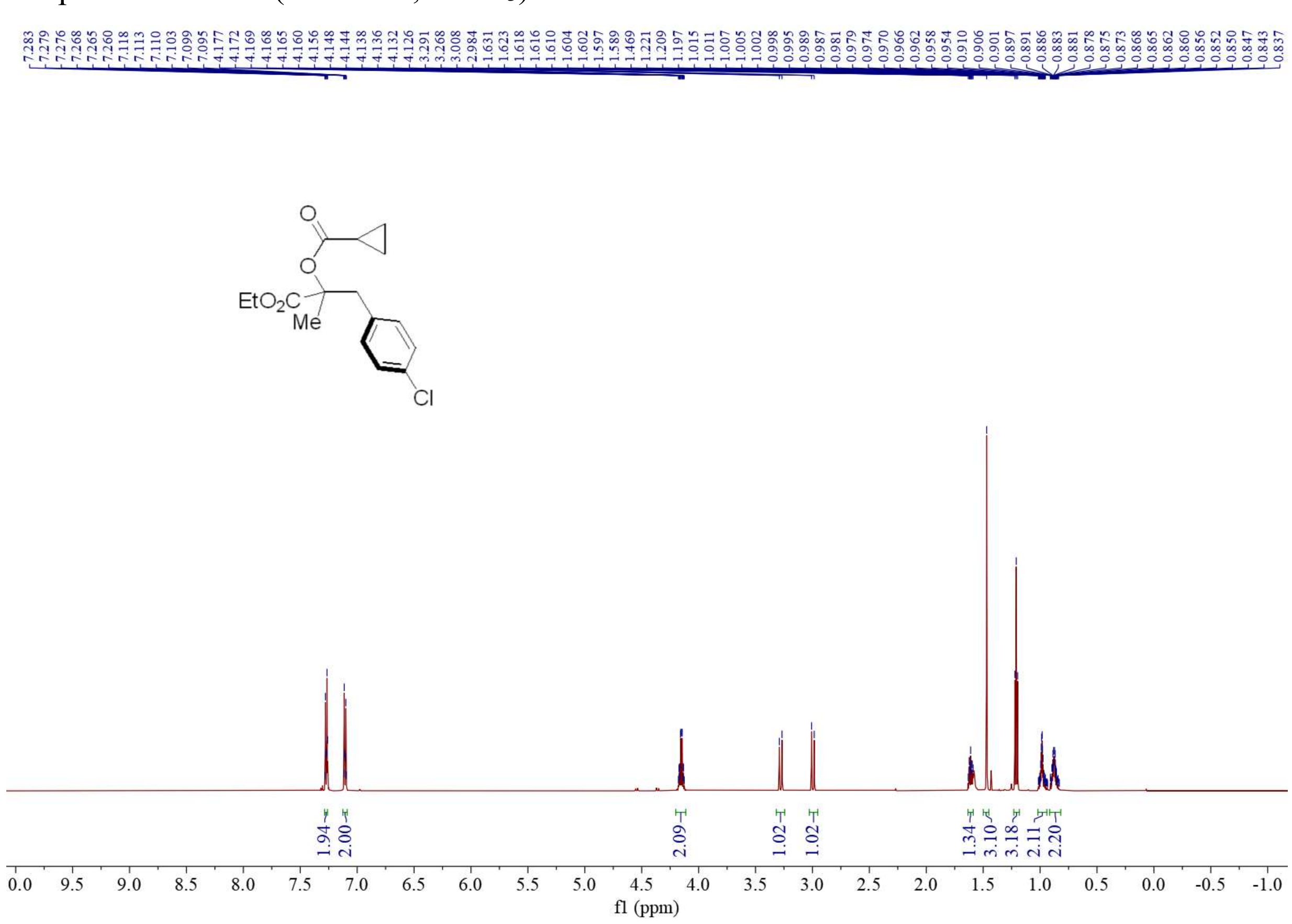

^{13}C NMR spectrum of **8ab** (151 MHz, $CDCl_3$)

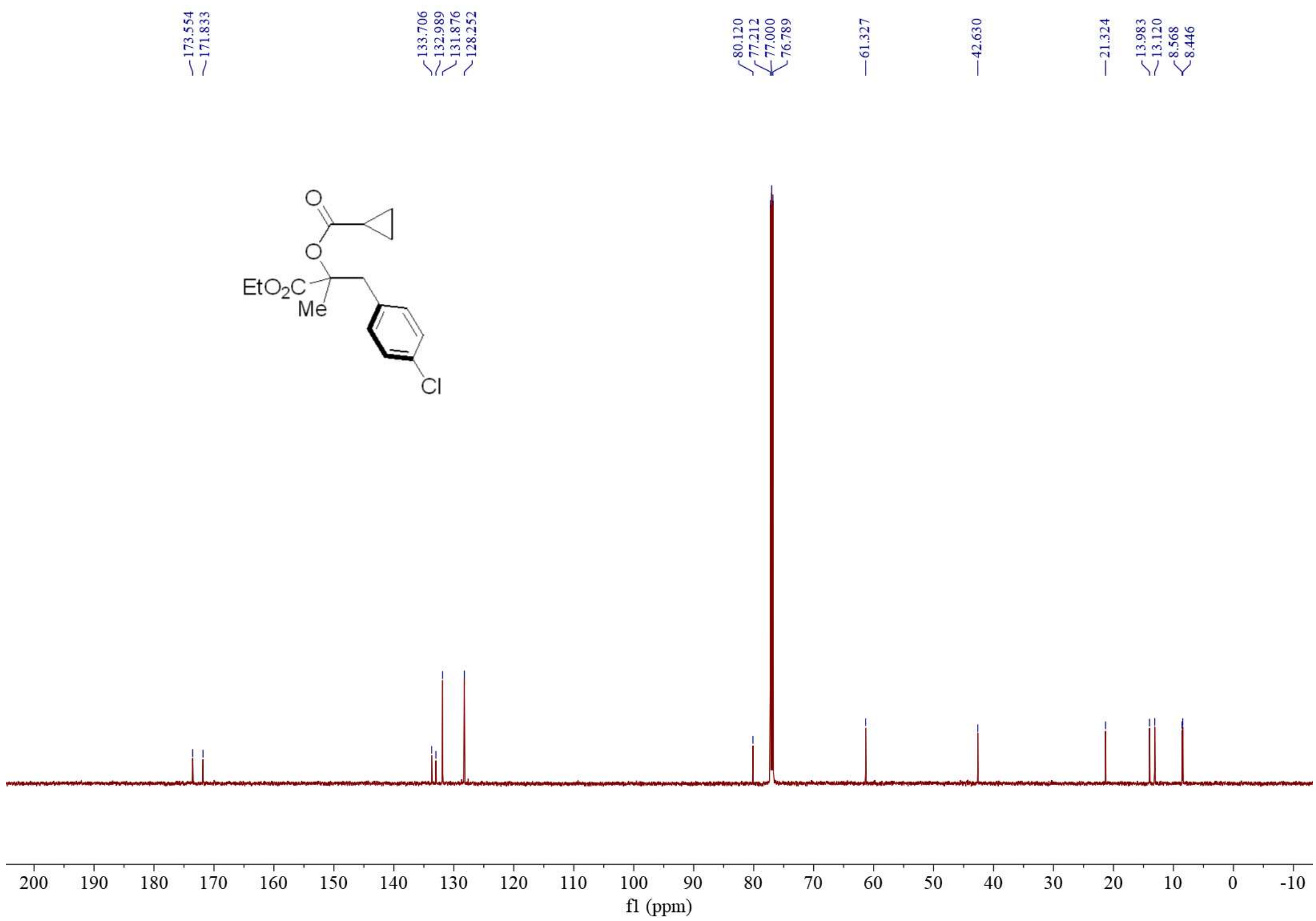

^{1}H NMR spectrum of **8ac** (500 MHz, $CDCl_3$)

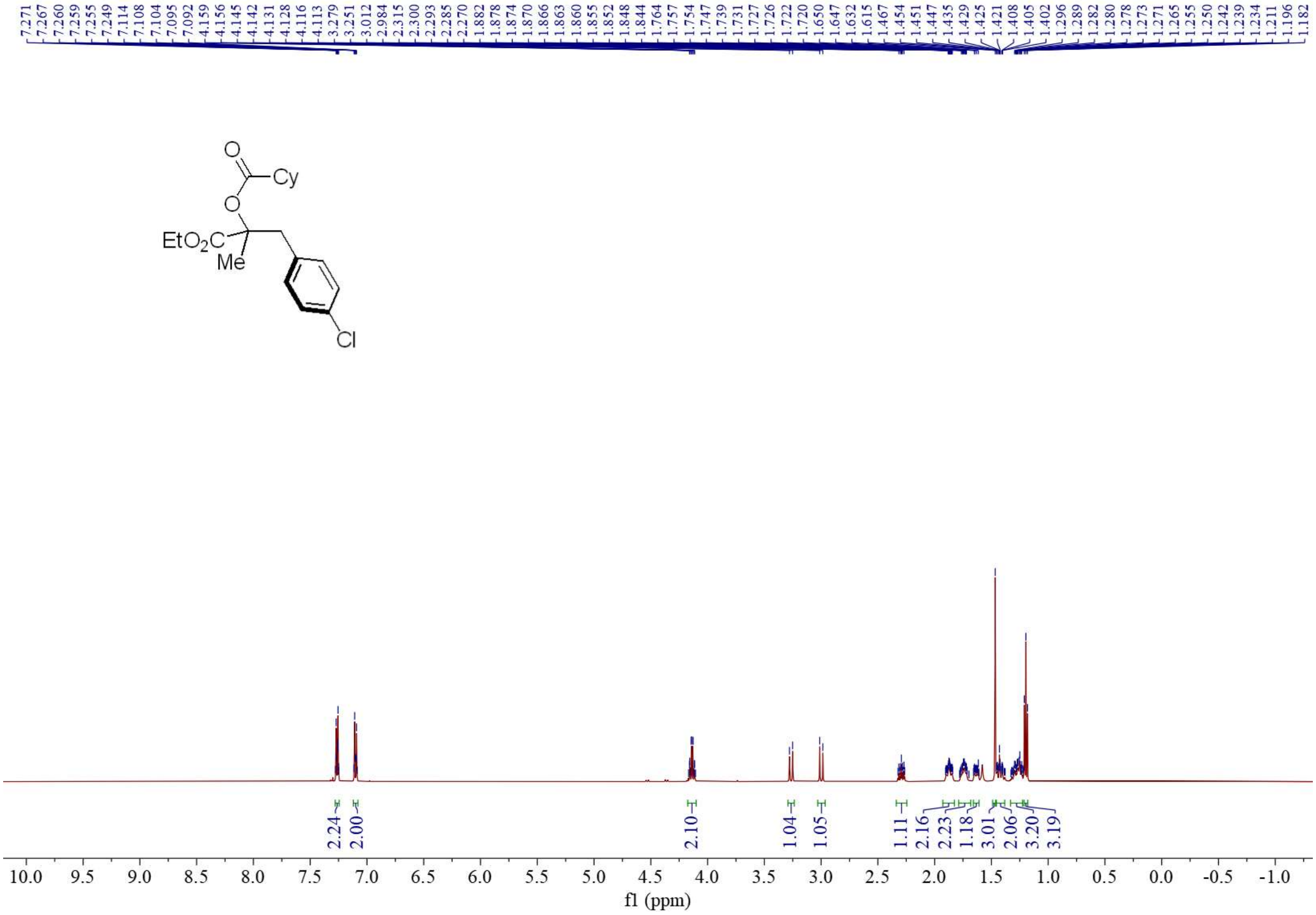

^{13}C NMR spectrum of **8ac** (126 MHz, $CDCl_3$)

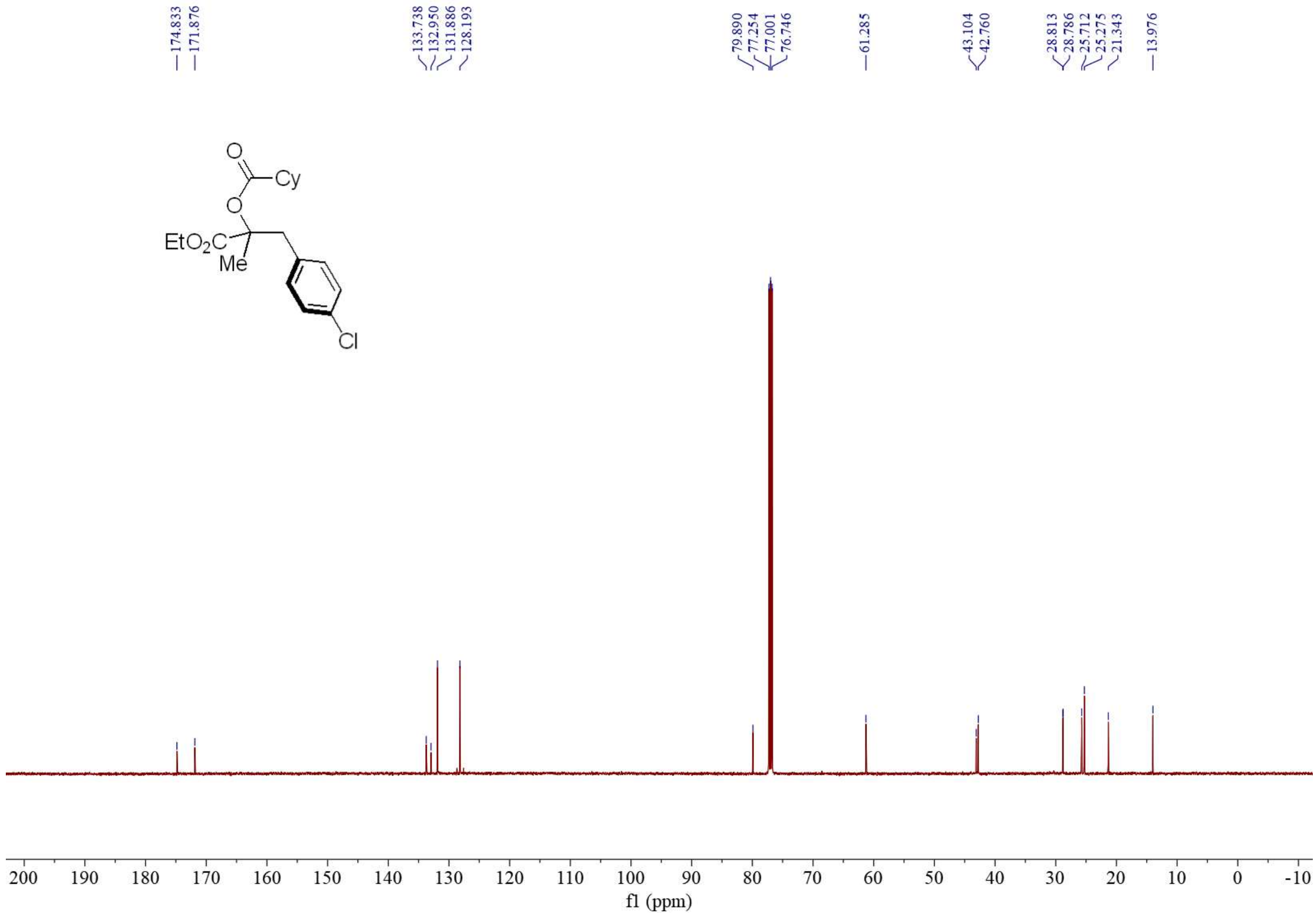

^{1}H NMR spectrum of **8ad** (600 MHz, $CDCl_3$)

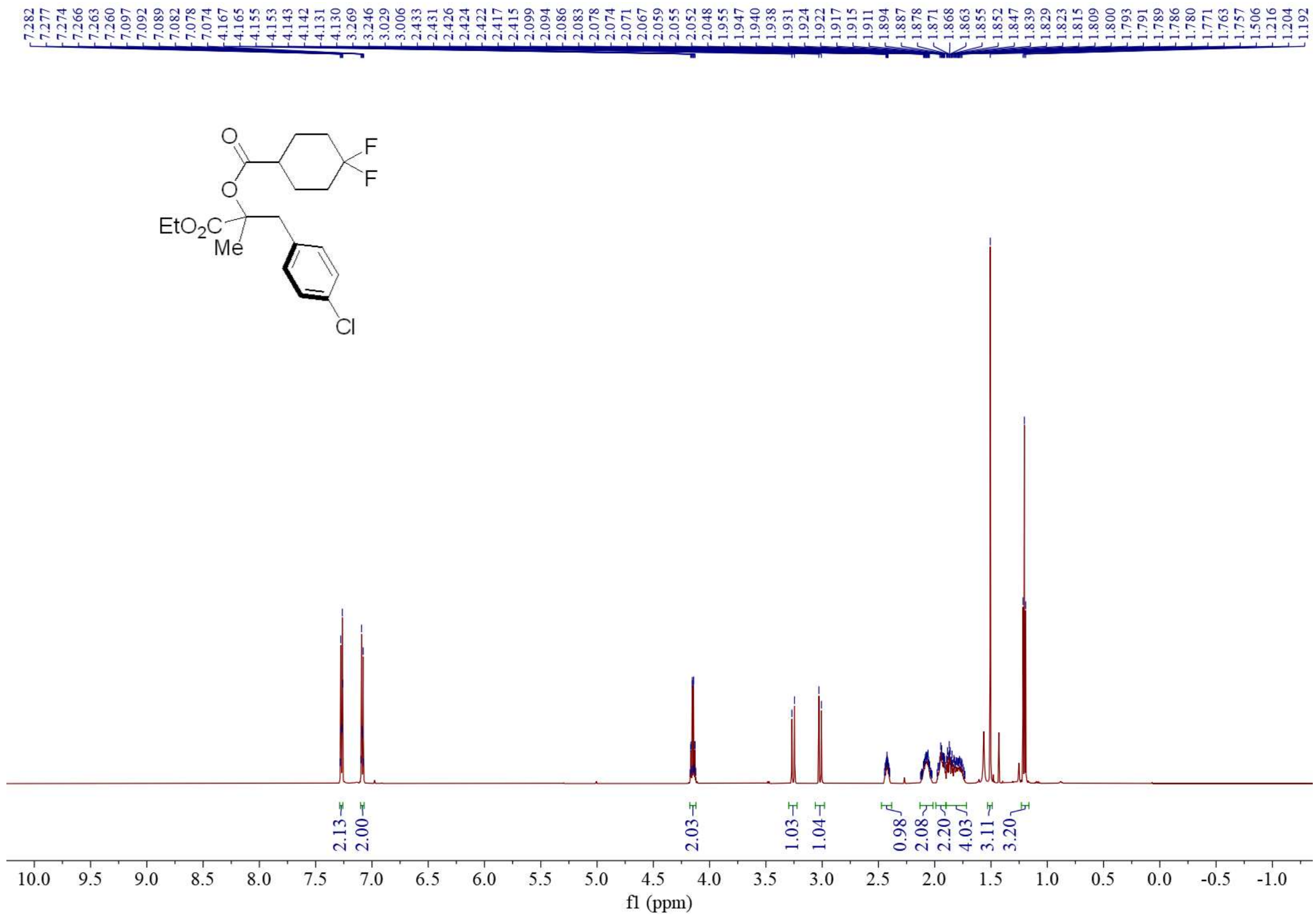

^{13}C NMR spectrum of **8ad** (151 MHz, $CDCl_3$)

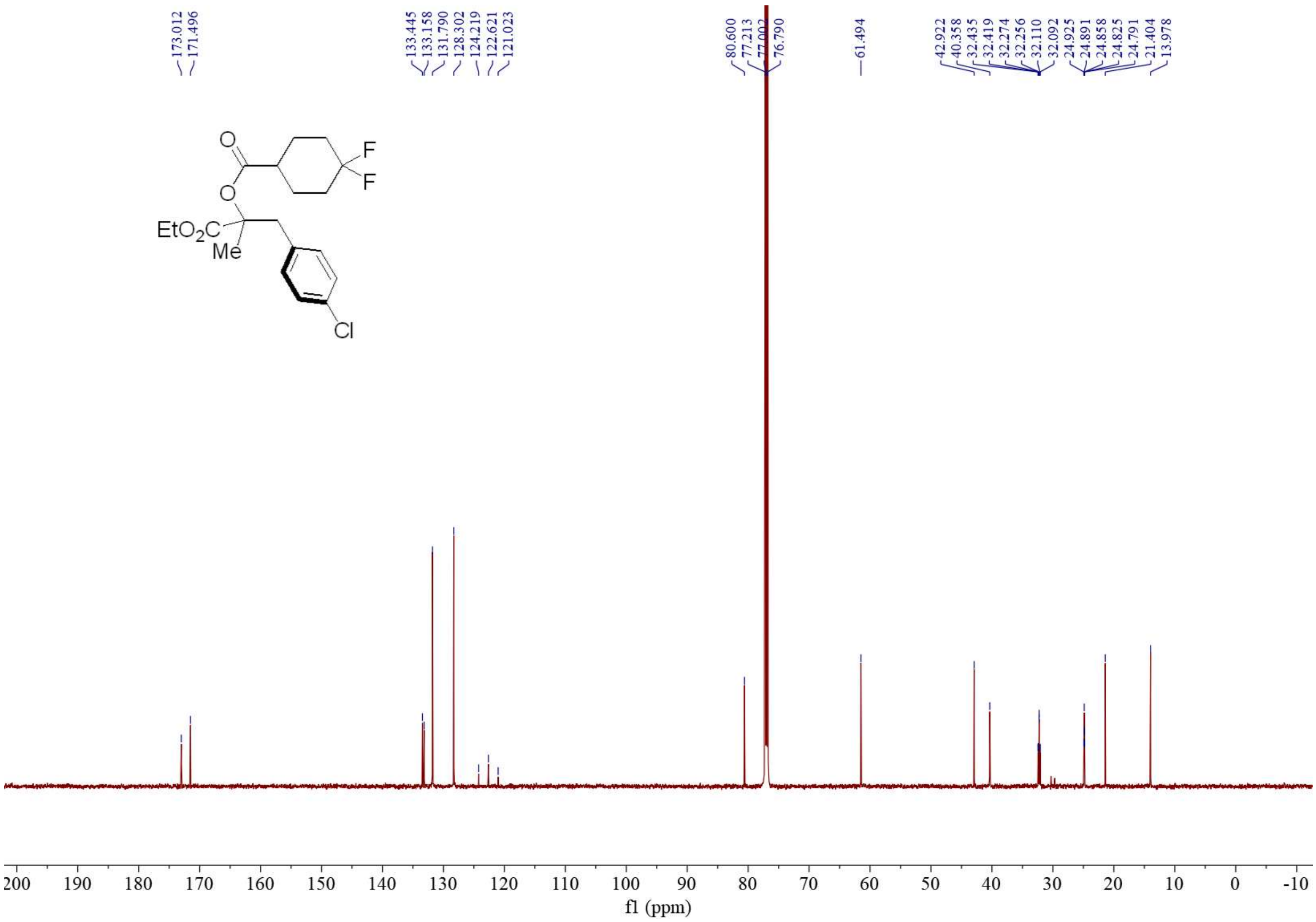

$^{19}F\{^1H\}$ NMR spectrum of **8ad** (377 MHz, $CDCl_3$)

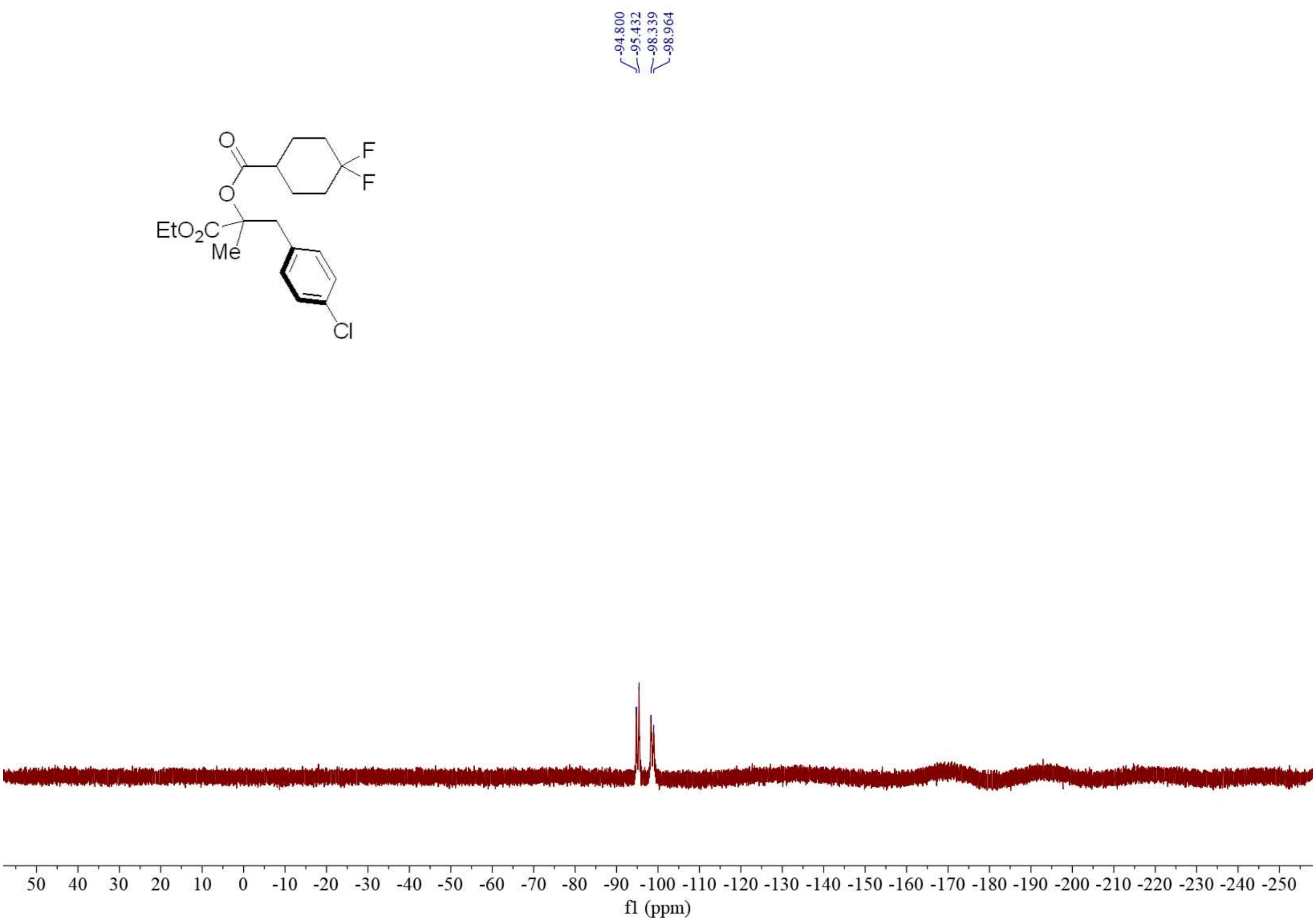

^{19}F NMR spectrum of **8ad** (377 MHz, $CDCl_3$)

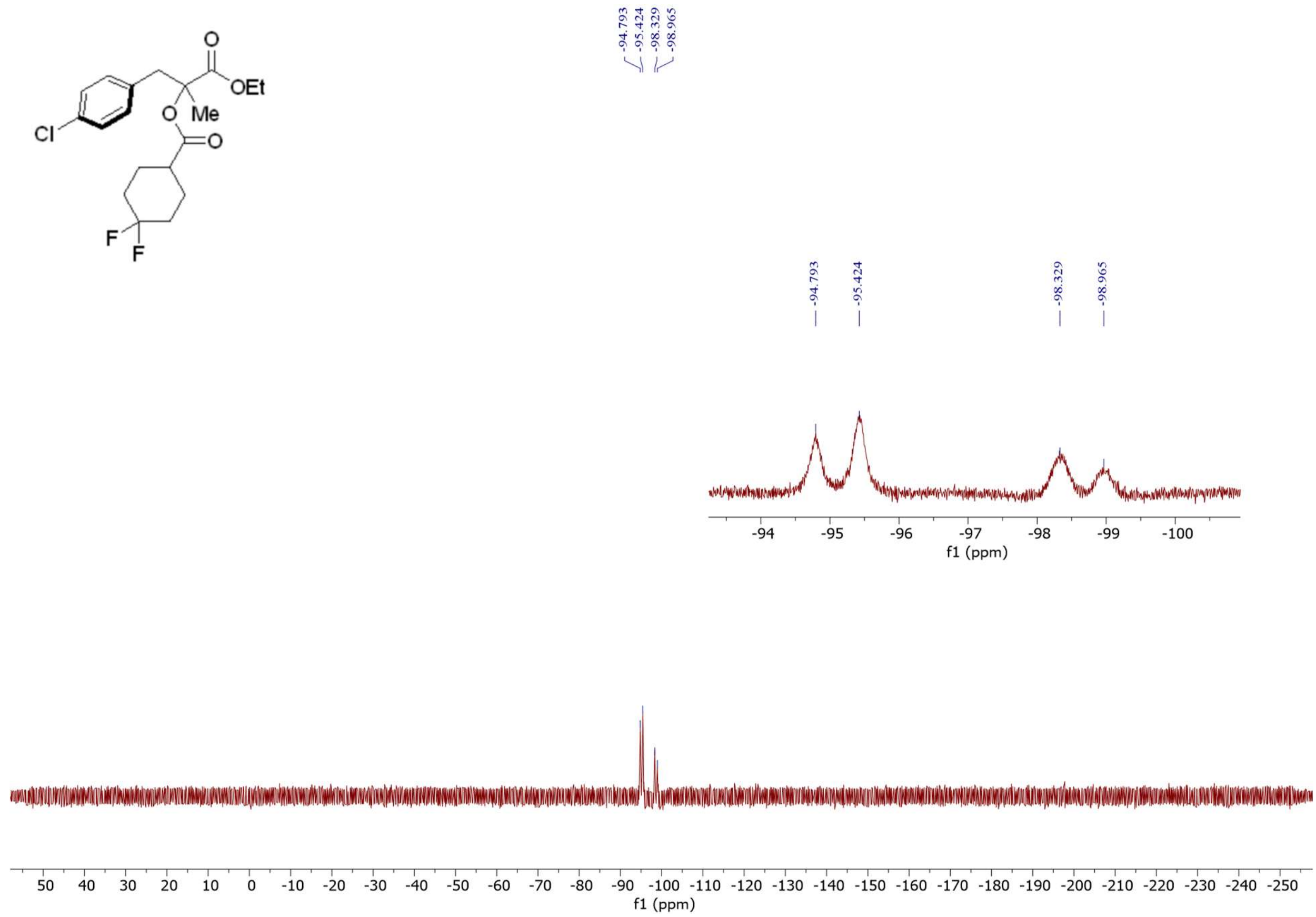

^{1}H NMR spectrum of **8ae** (600 MHz, $CDCl_3$)

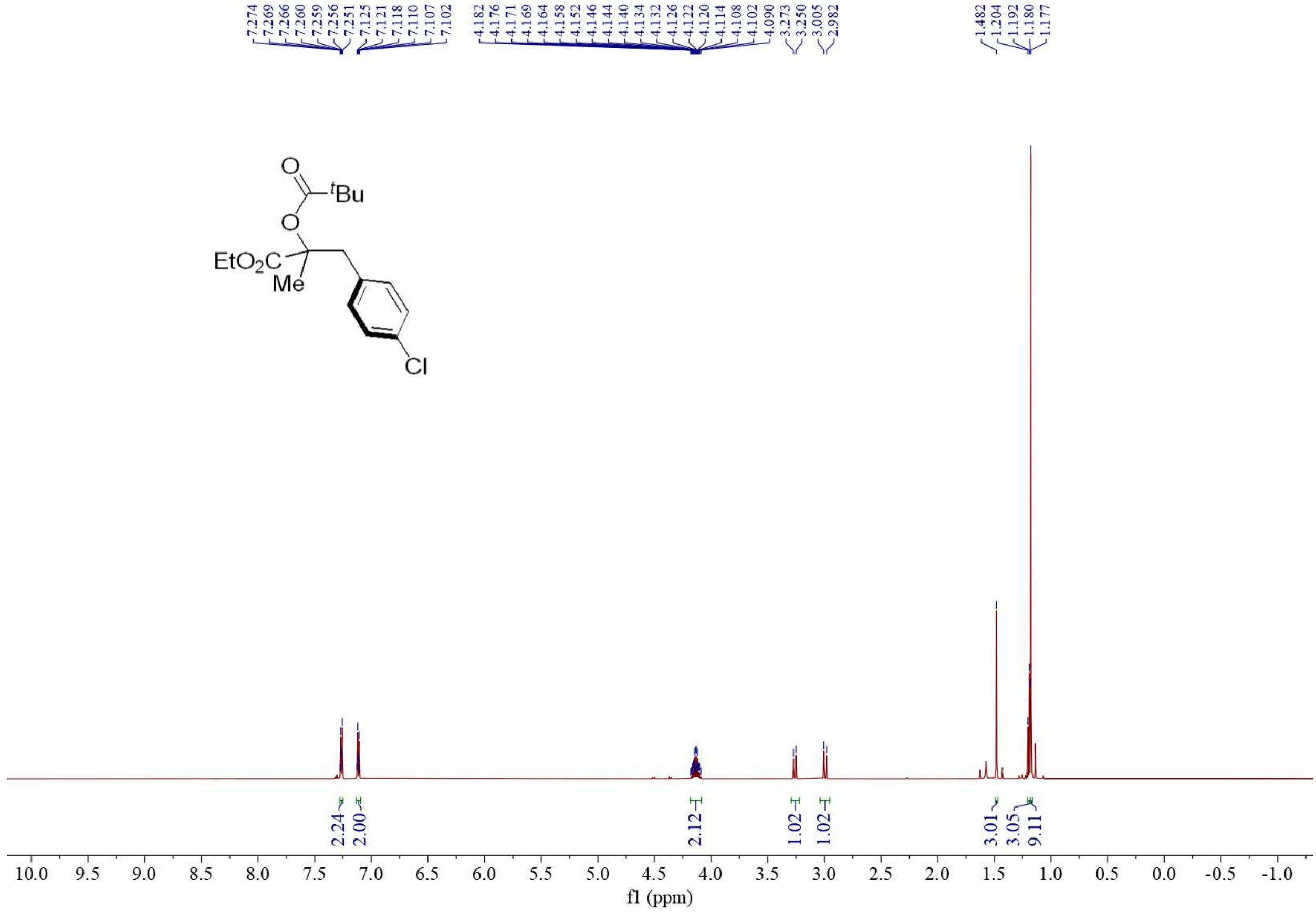

^{13}C NMR spectrum of **8ae** (151 MHz, $CDCl_3$)

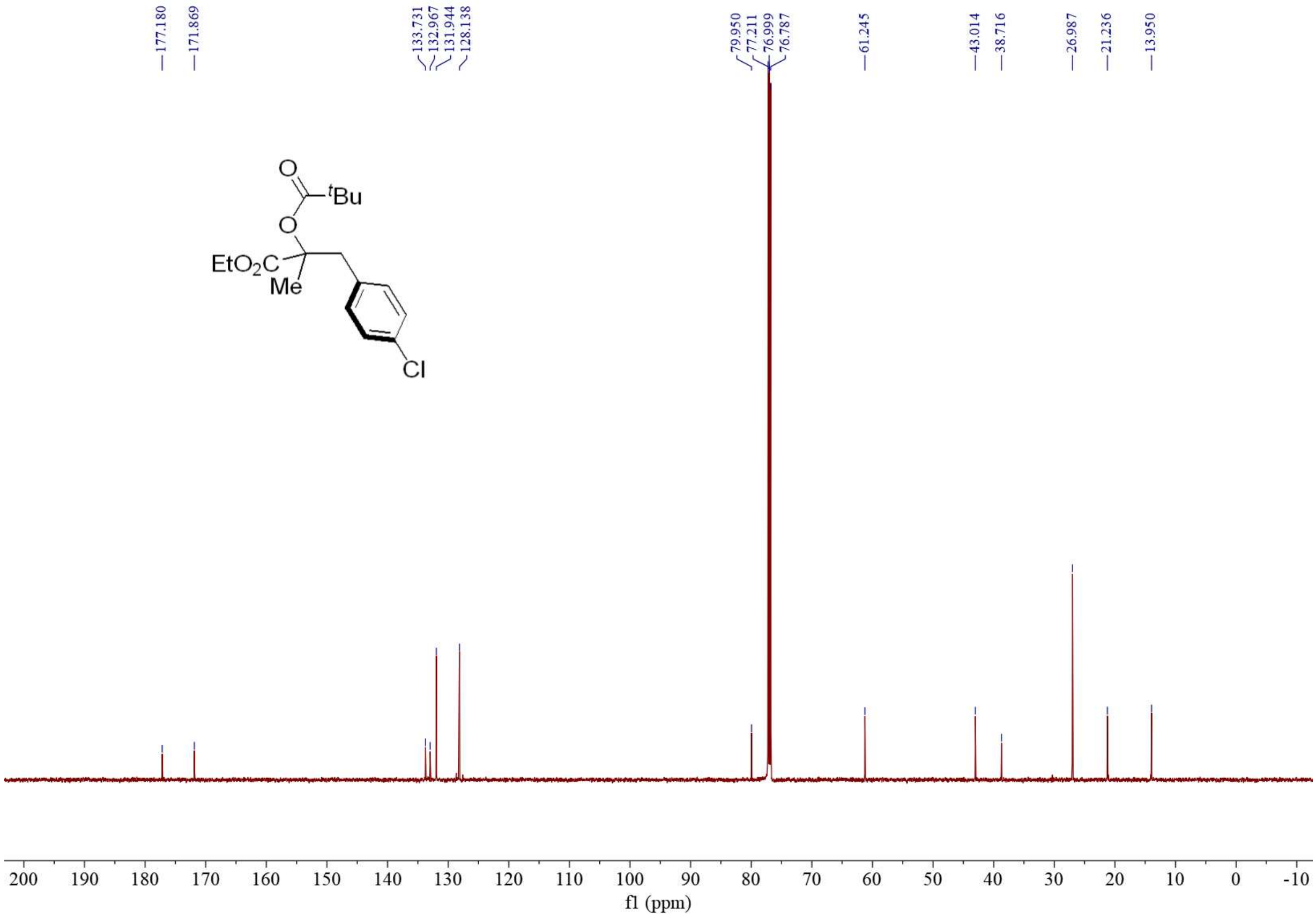

^{1}H NMR spectrum of **8af** (500 MHz, $CDCl_3$)

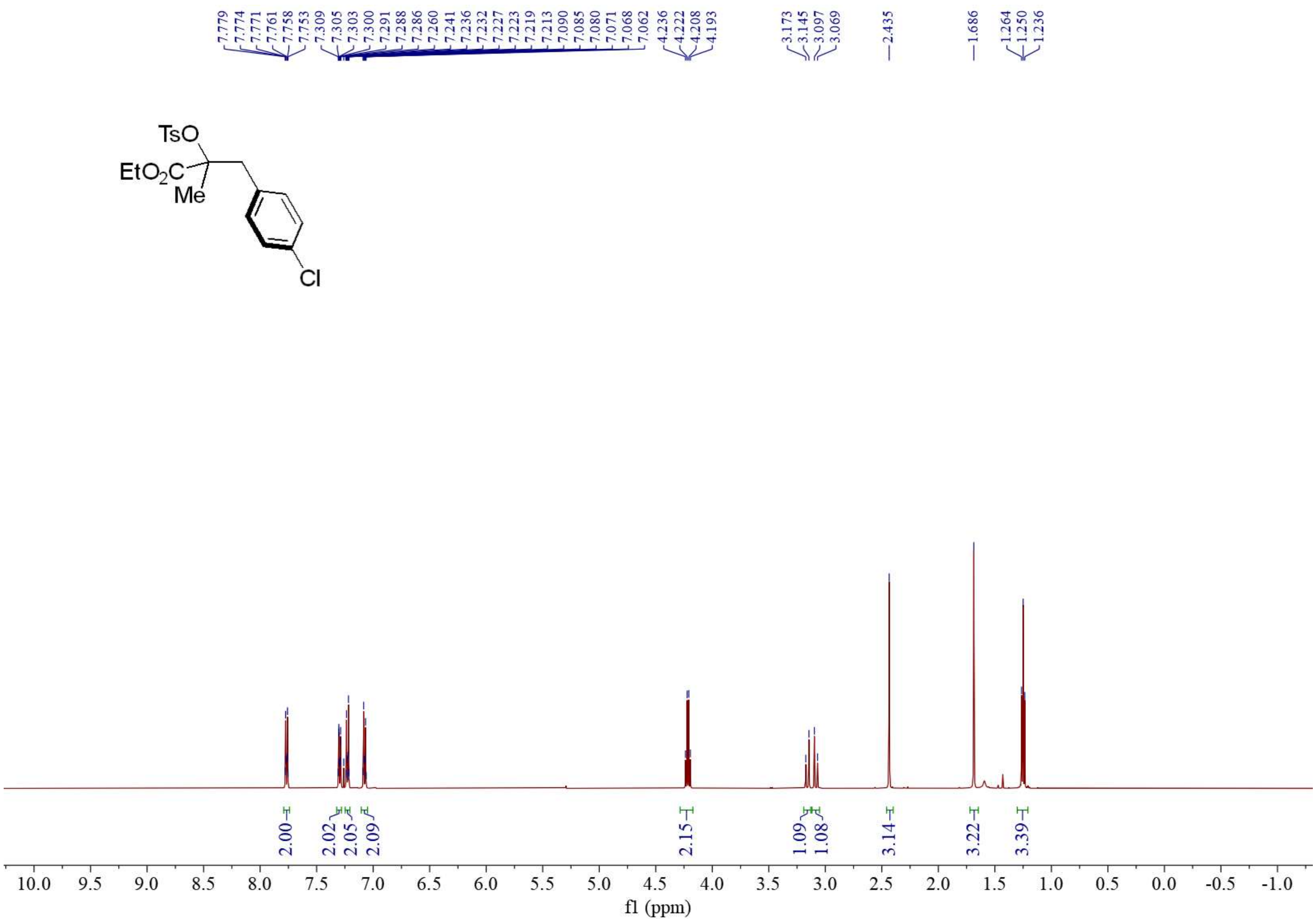

^{13}C NMR spectrum of **8af** (126 MHz, $CDCl_3$)

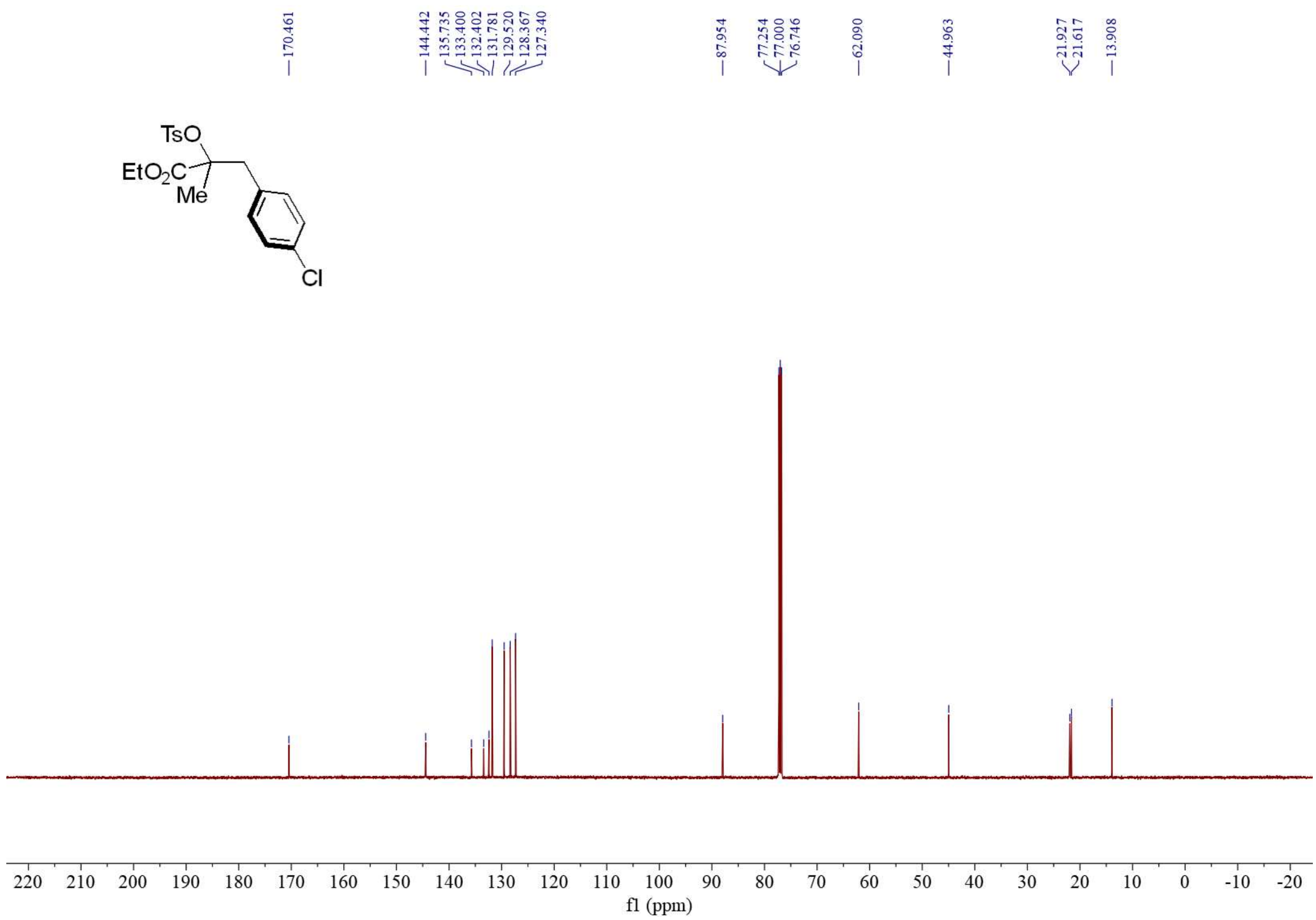

^{1}H NMR spectrum of **9a** (500 MHz, $CDCl_3$)

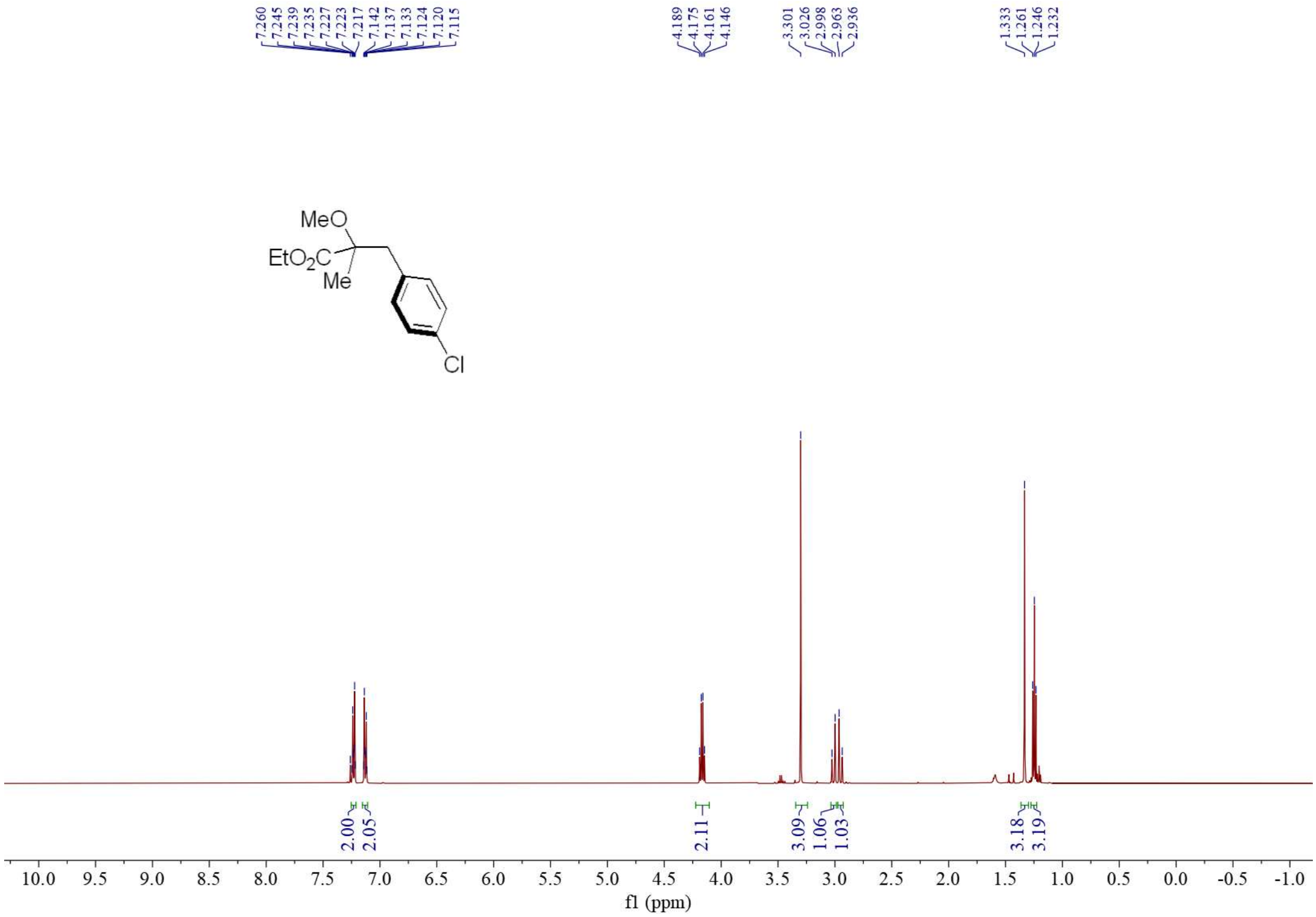

^{13}C NMR spectrum of **9a** (126 MHz, $CDCl_3$)

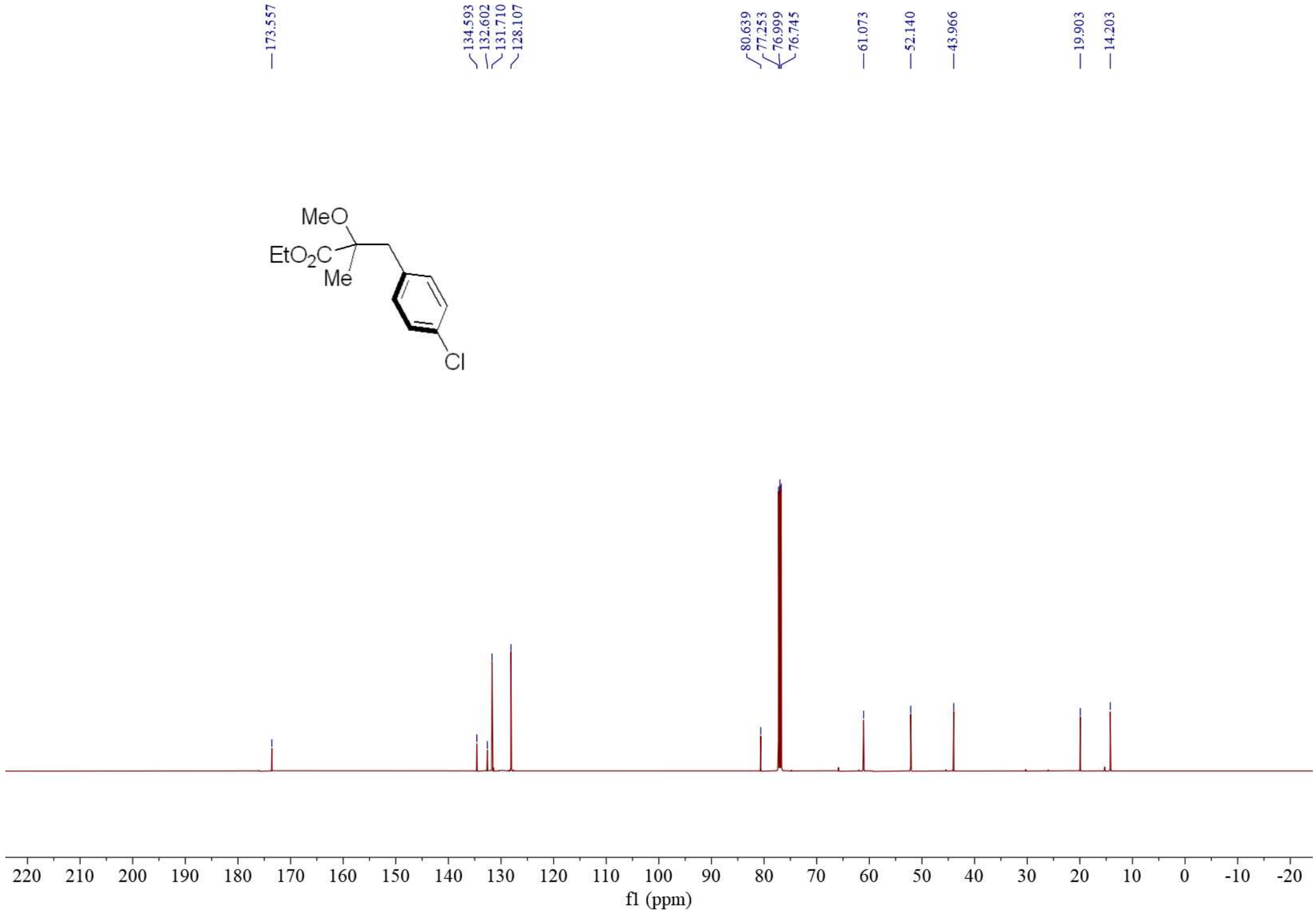

^{1}H NMR spectrum of **9b** (400 MHz, $CDCl_3$)

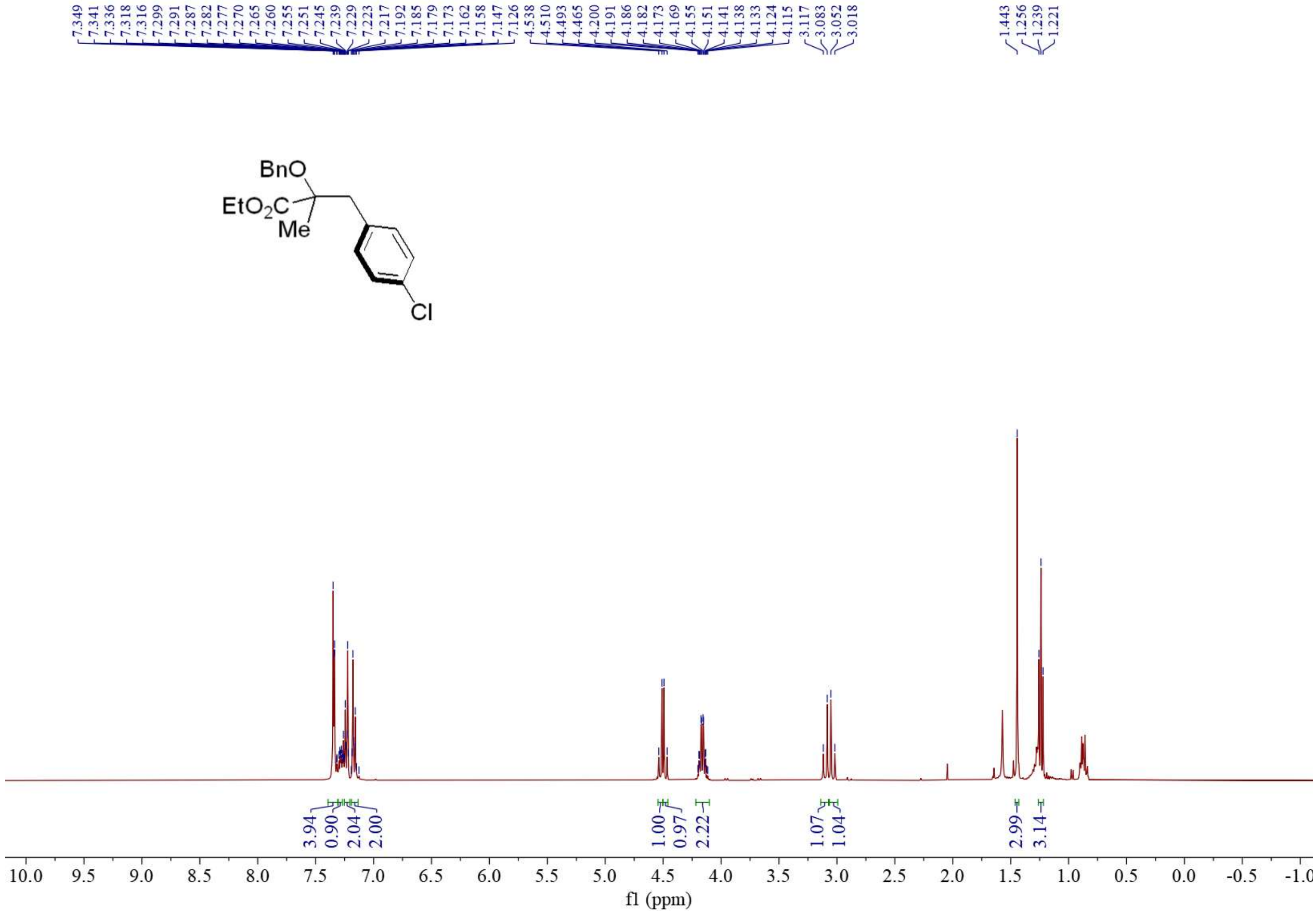

^{13}C NMR spectrum of **9b** (101 MHz, $CDCl_3$)

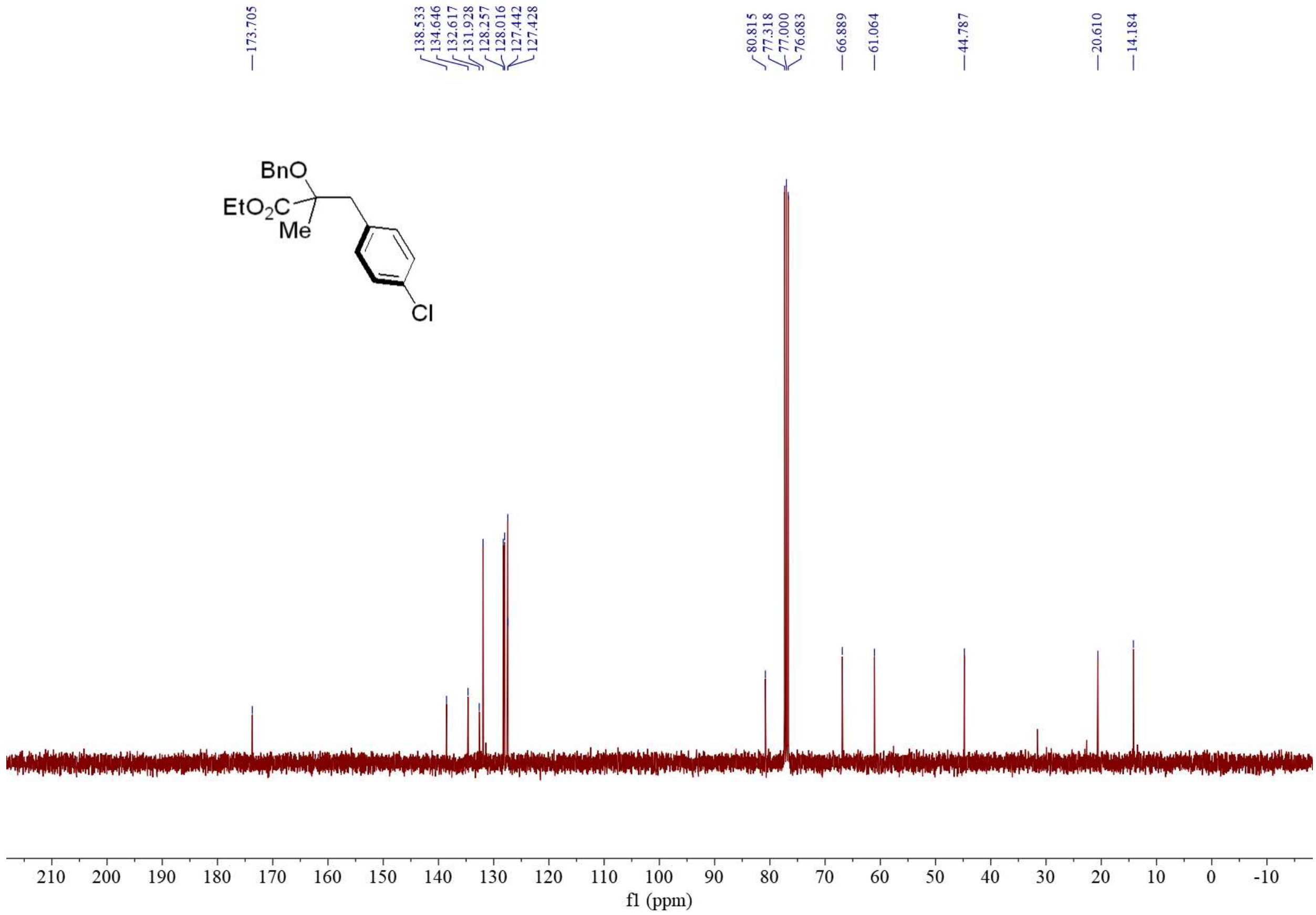

^{1}H NMR spectrum of **9c** (600 MHz, $CDCl_3$)

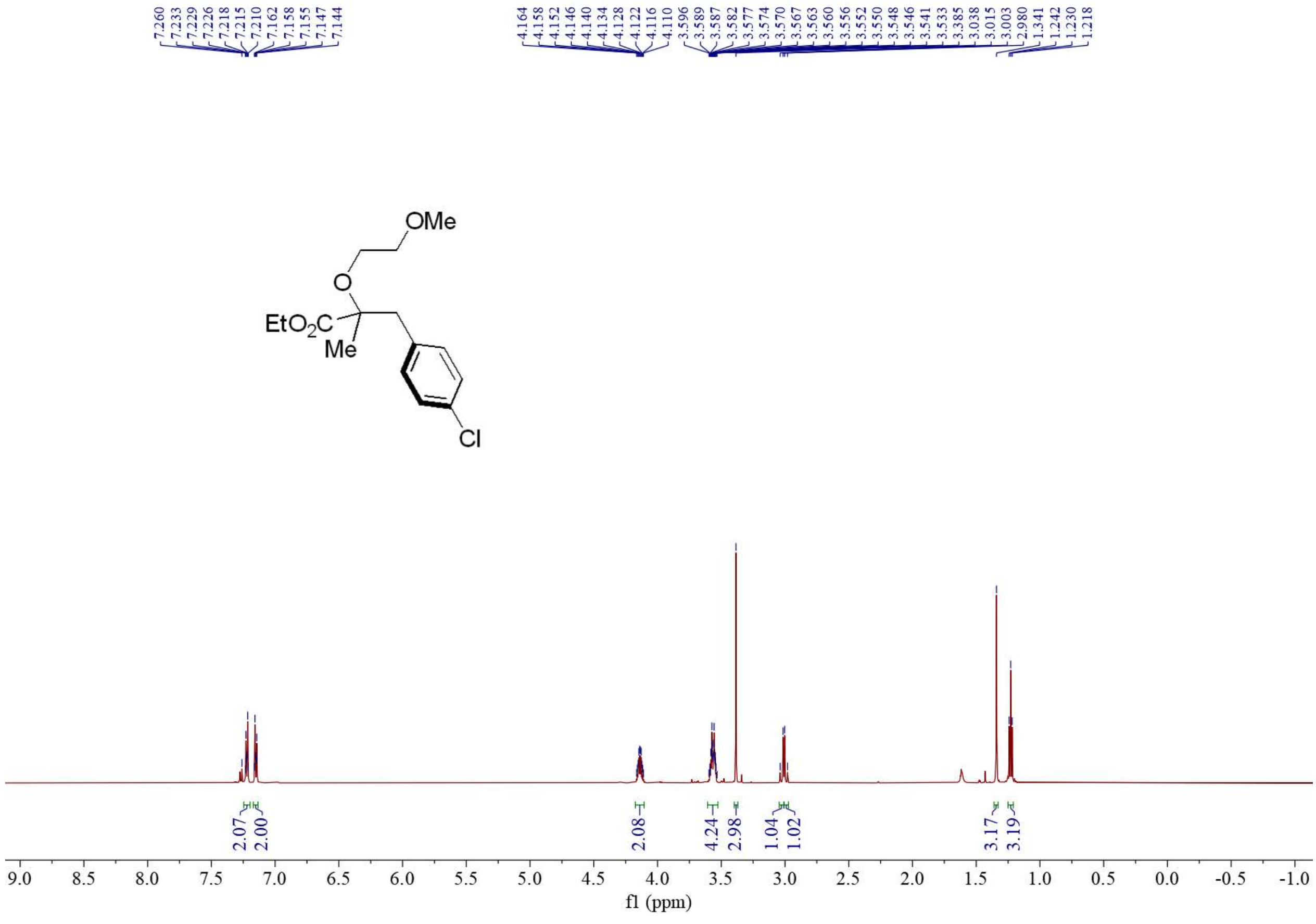

^{13}C NMR spectrum of **9c** (151 MHz, $CDCl_3$)

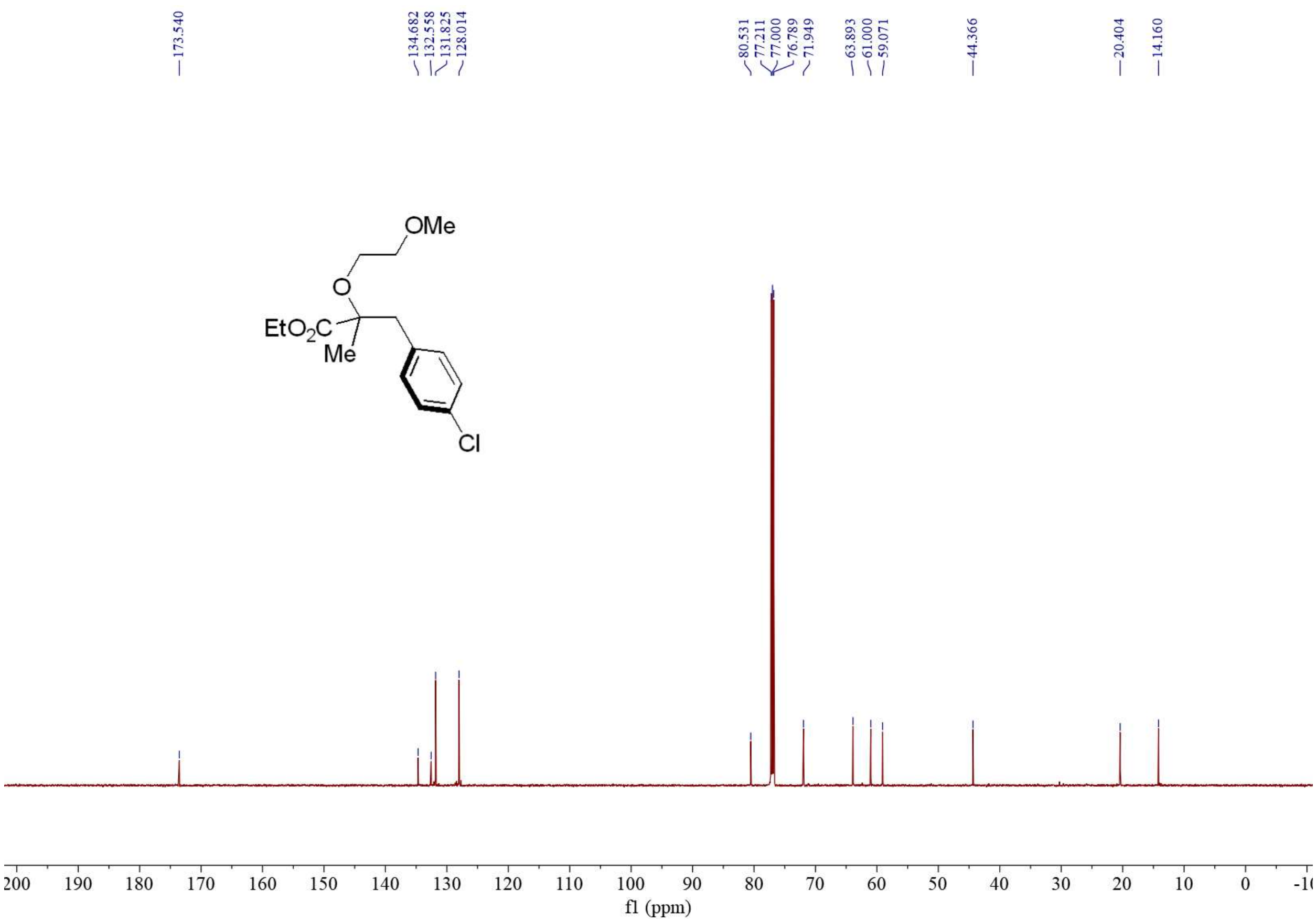

^{1}H NMR spectrum of **9d** (600 MHz, $CDCl_3$)

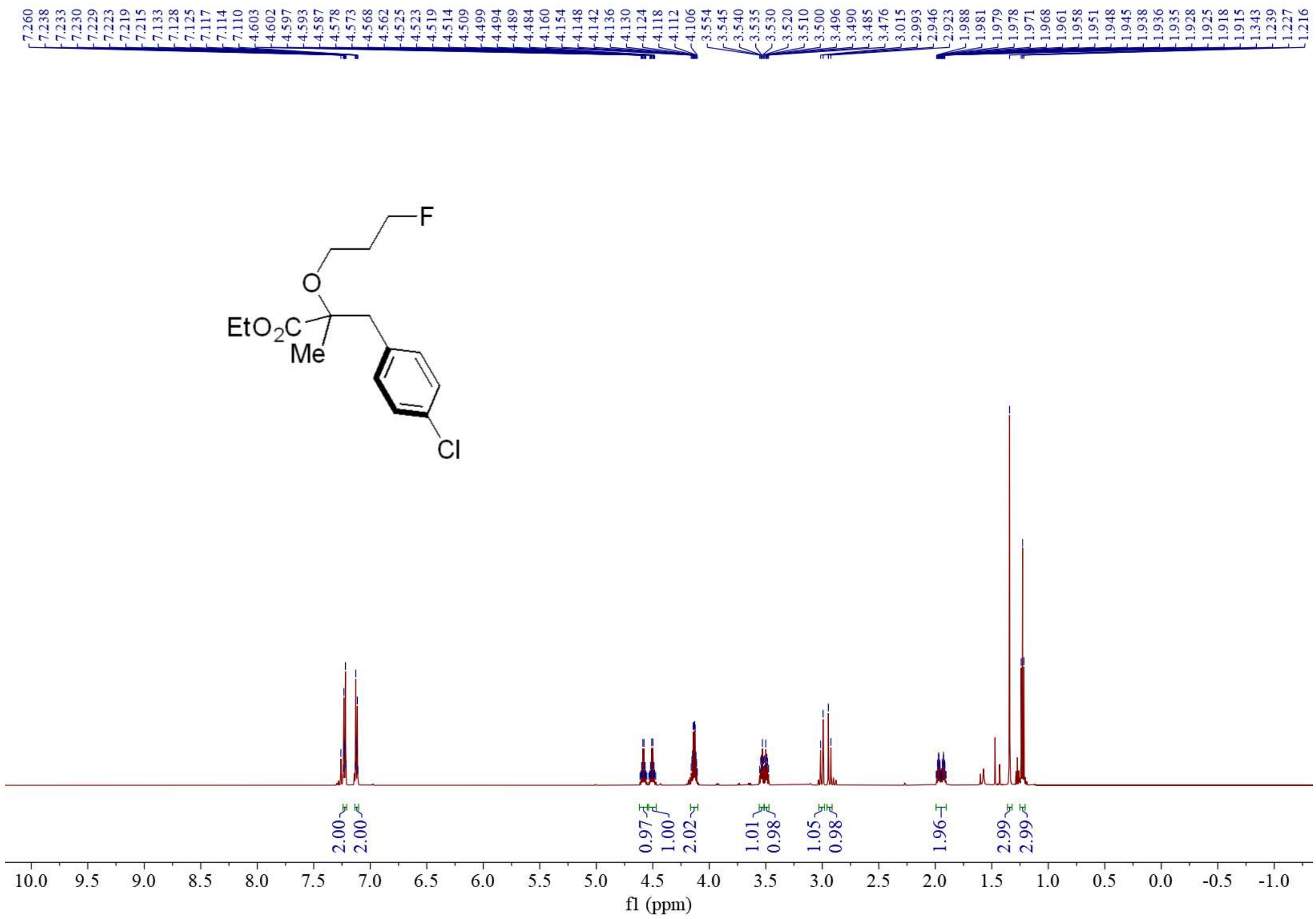

^{13}C NMR spectrum of **9d** (151 MHz, $CDCl_3$)

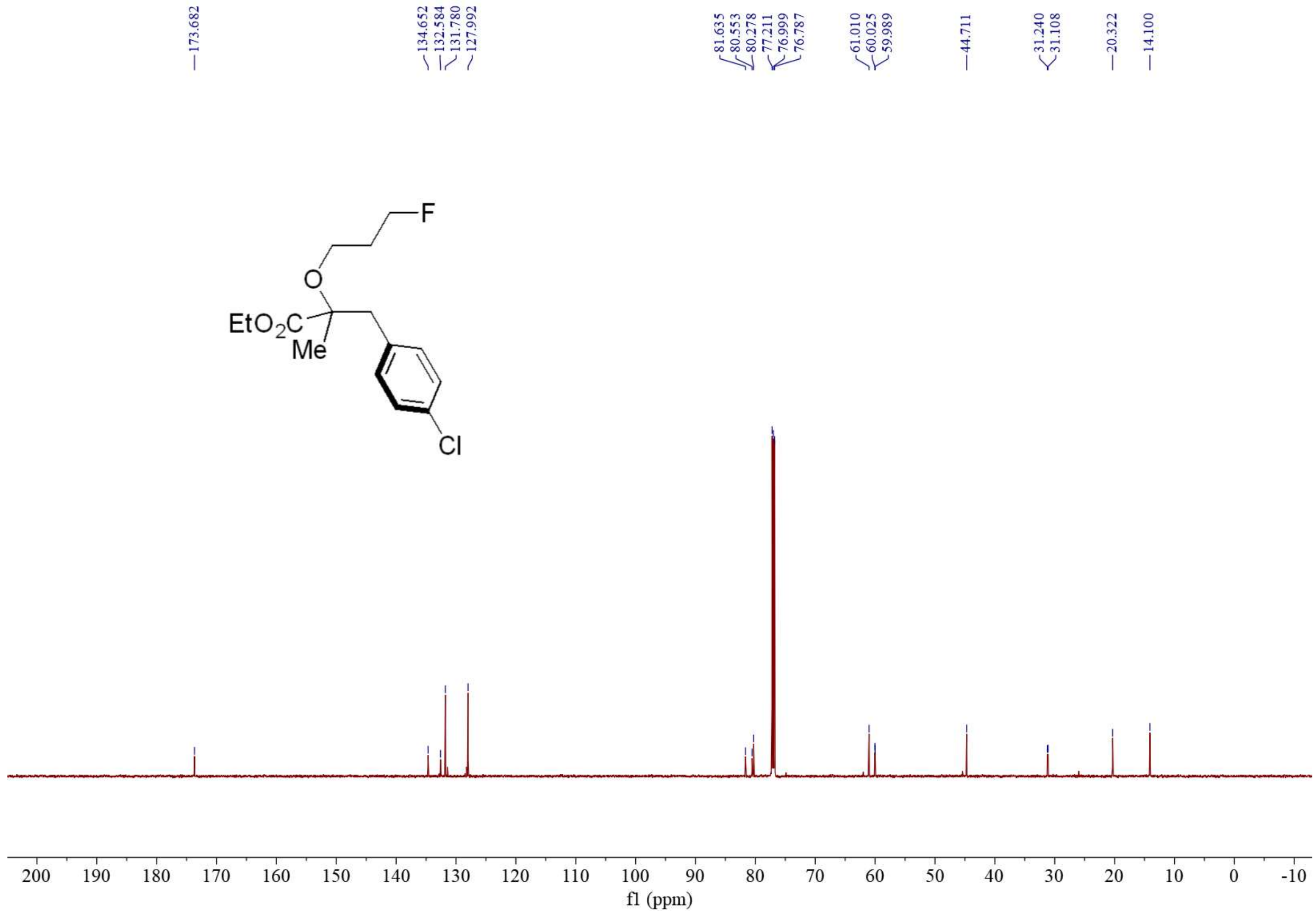

$^{19}F\{^{1}H\}$ NMR spectrum of **9d** (377 MHz, $CDCl_3$)

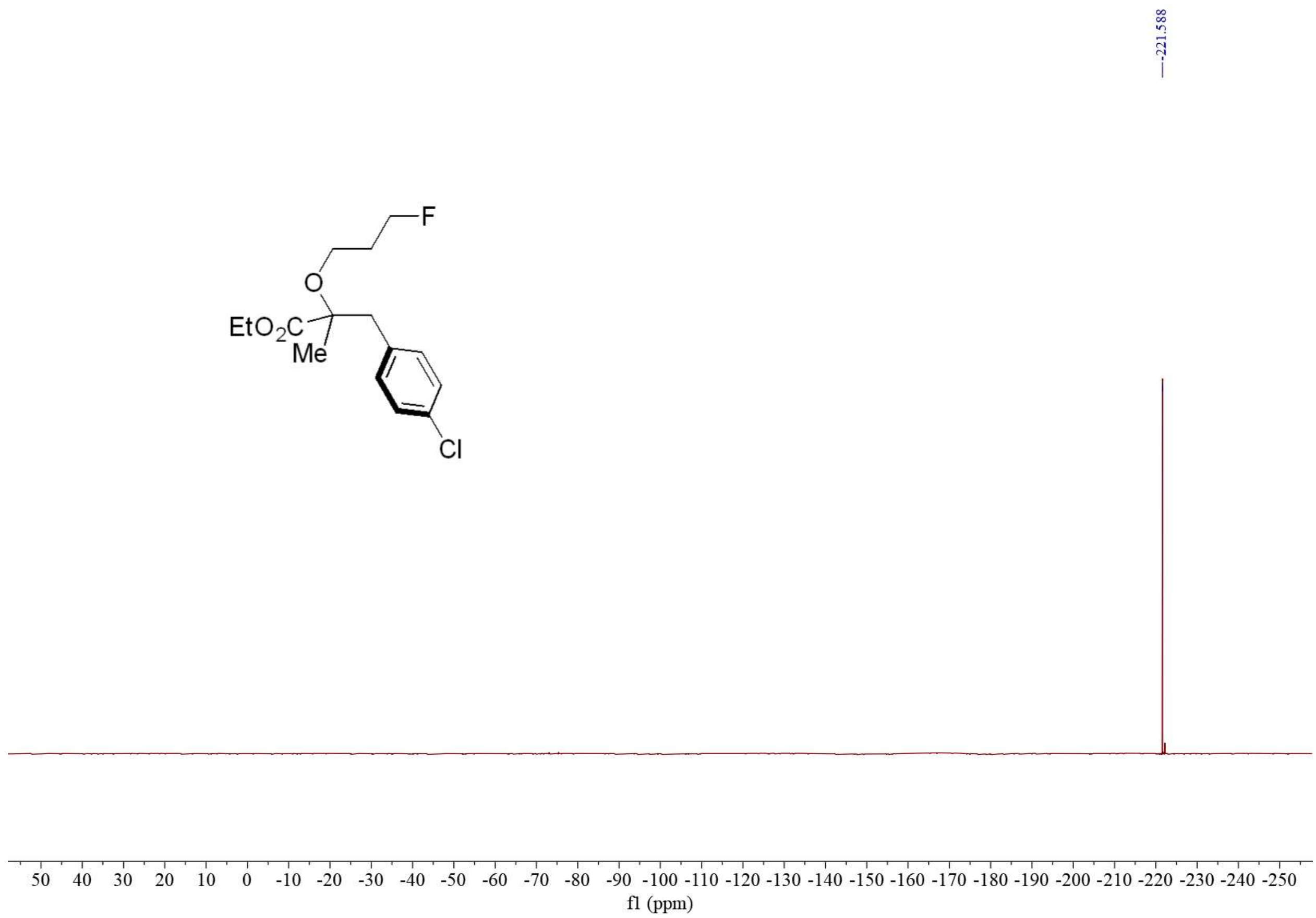

^{19}F NMR spectrum of **9d** (377 MHz, $CDCl_3$)

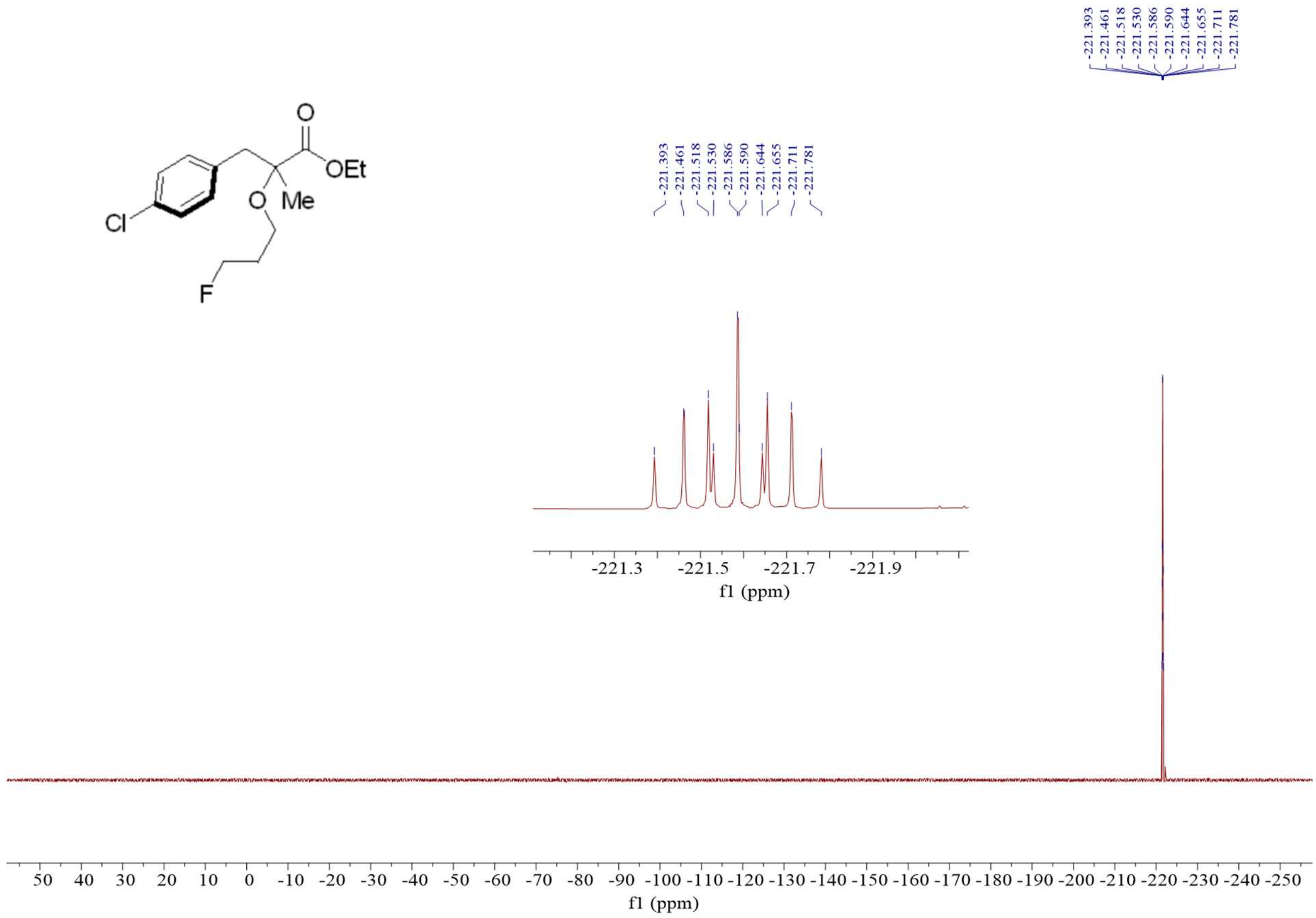

^{1}H NMR spectrum of **9e** (600 MHz, $CDCl_3$)

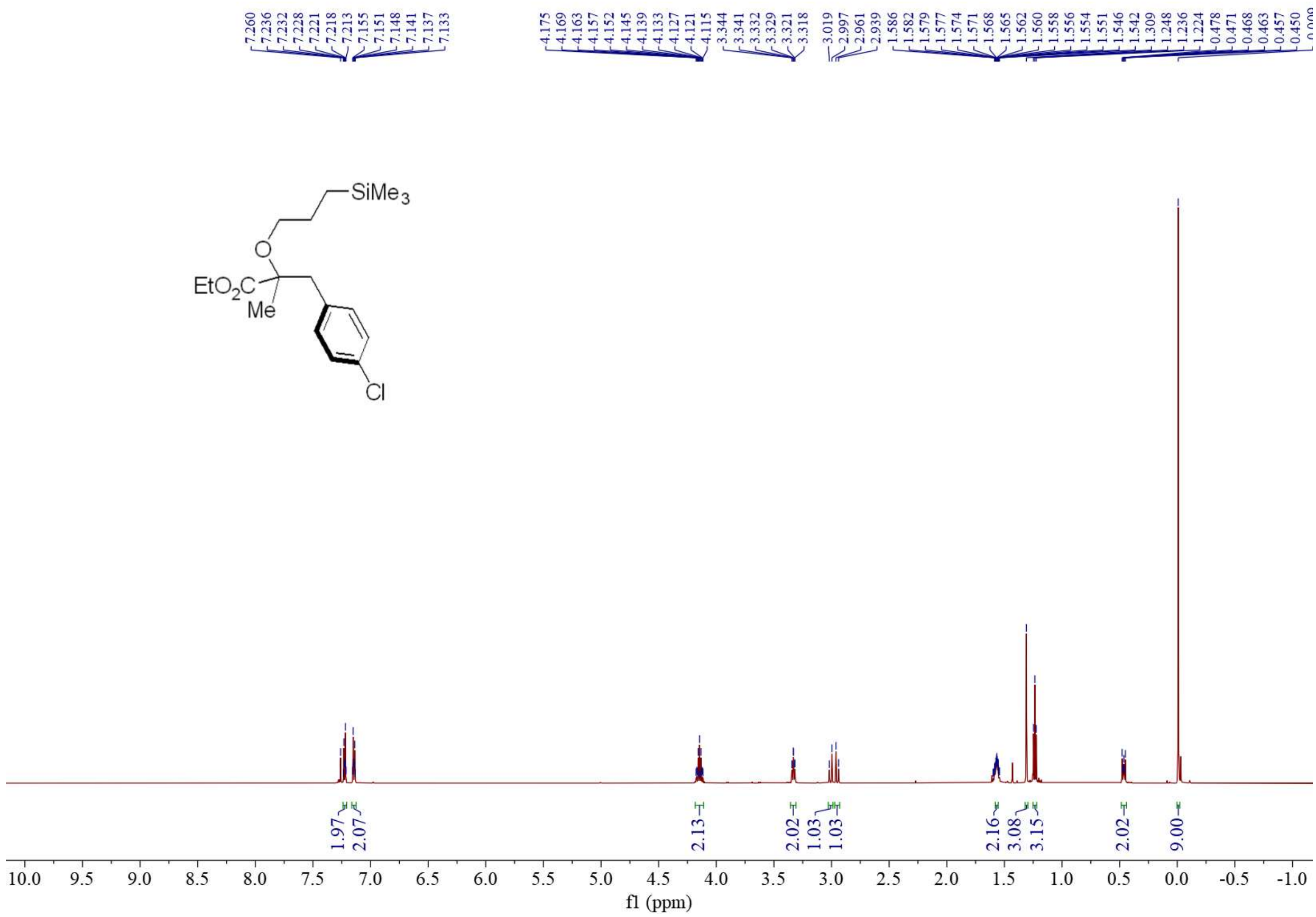

^{13}C NMR spectrum of **9e** (151 MHz, $CDCl_3$)

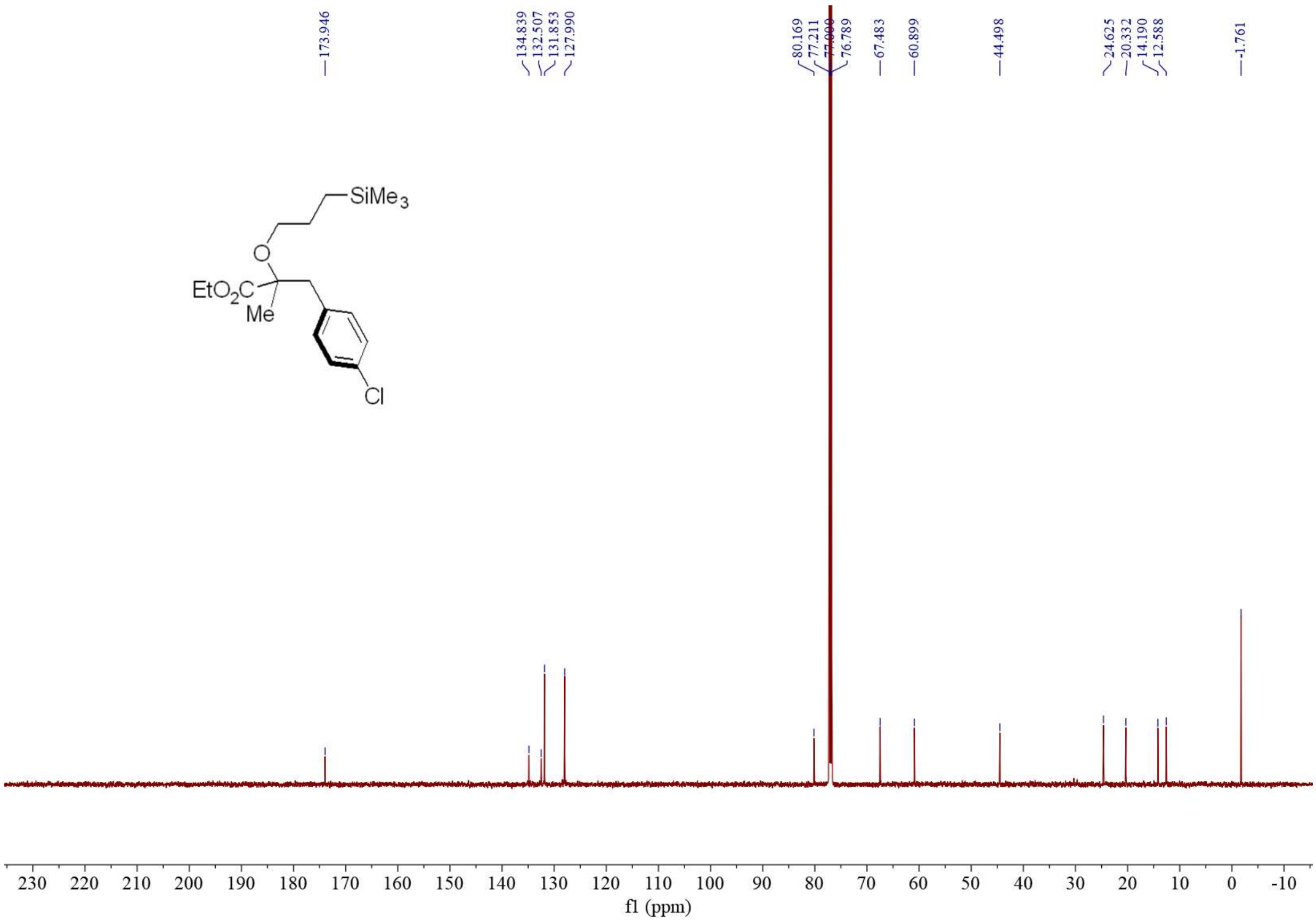

^{1}H NMR spectrum of **9f** (600 MHz, $CDCl_3$)

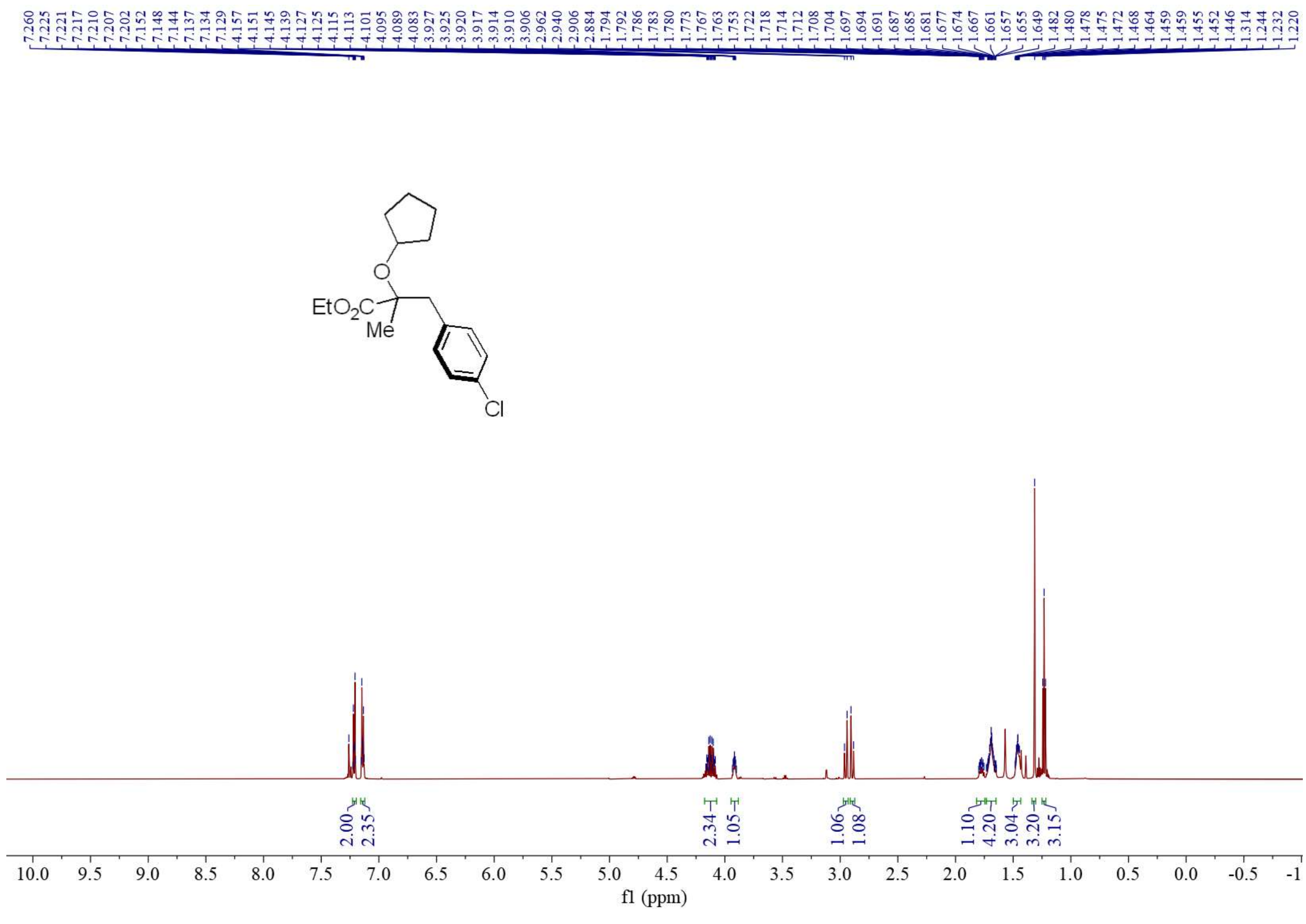

^{13}C NMR spectrum of **9f** (151 MHz, $CDCl_3$)

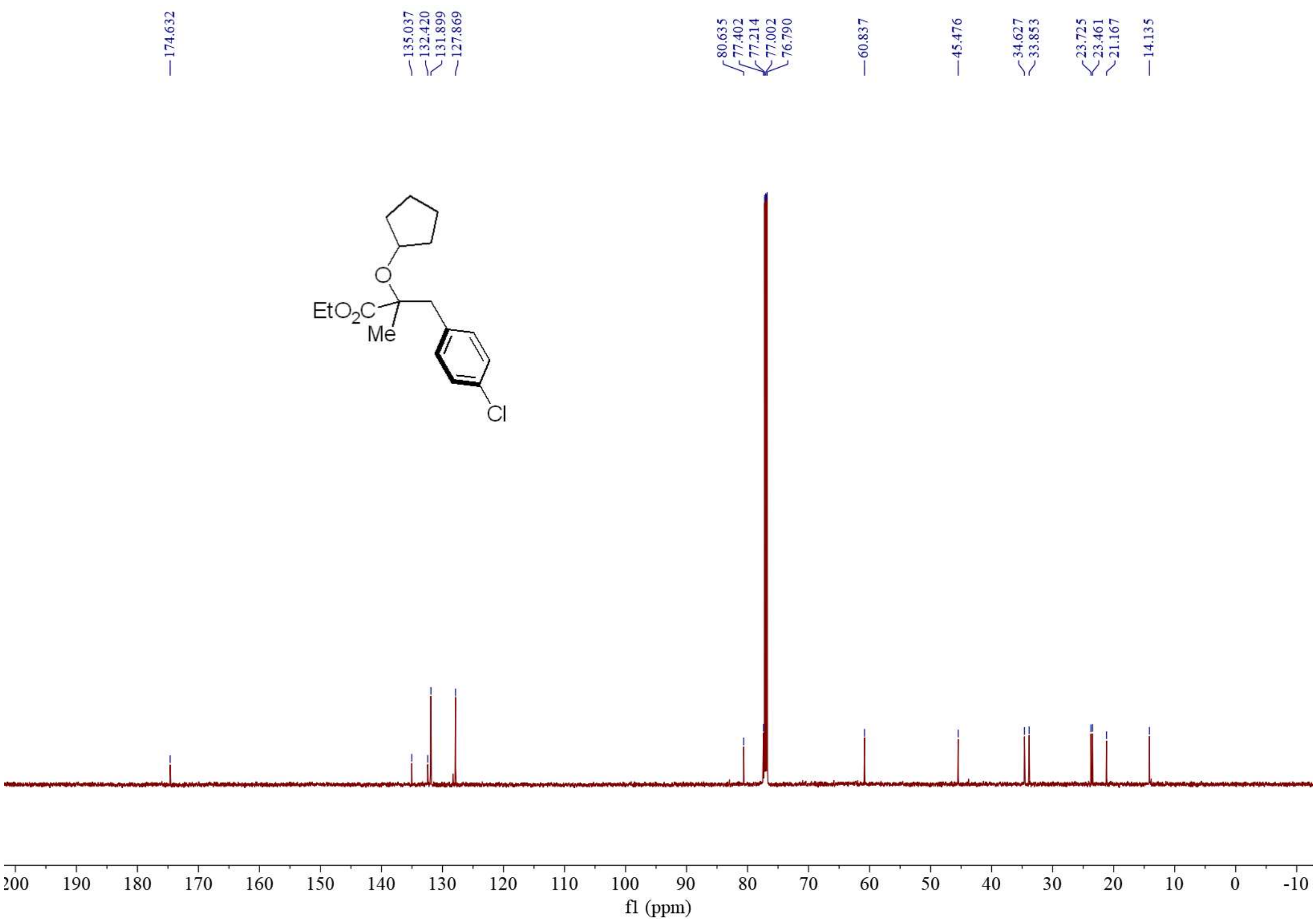

^{1}H NMR spectrum of **9g** (600 MHz, $CDCl_3$)

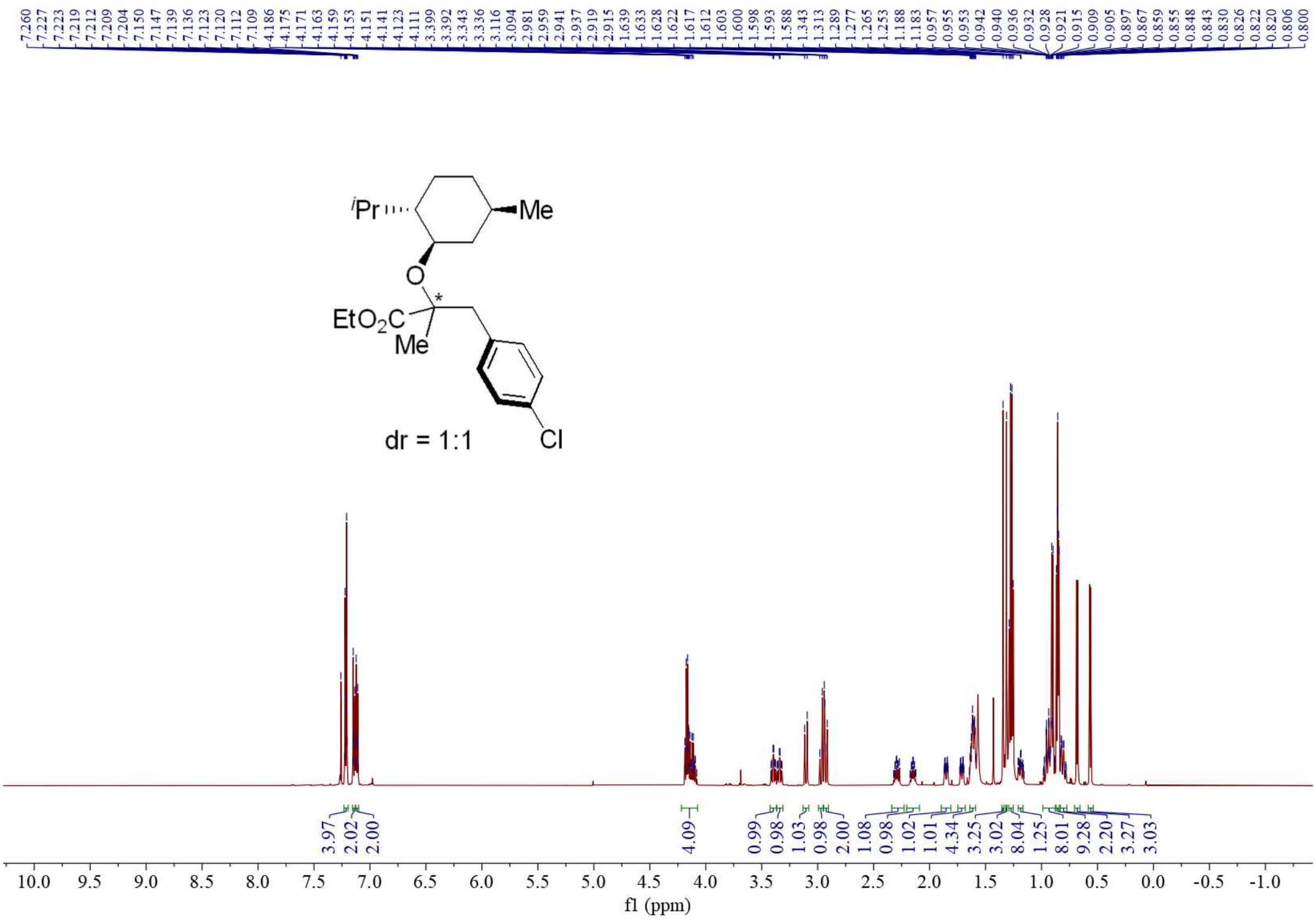

^{13}C NMR spectrum of **9g** (151 MHz, $CDCl_3$)

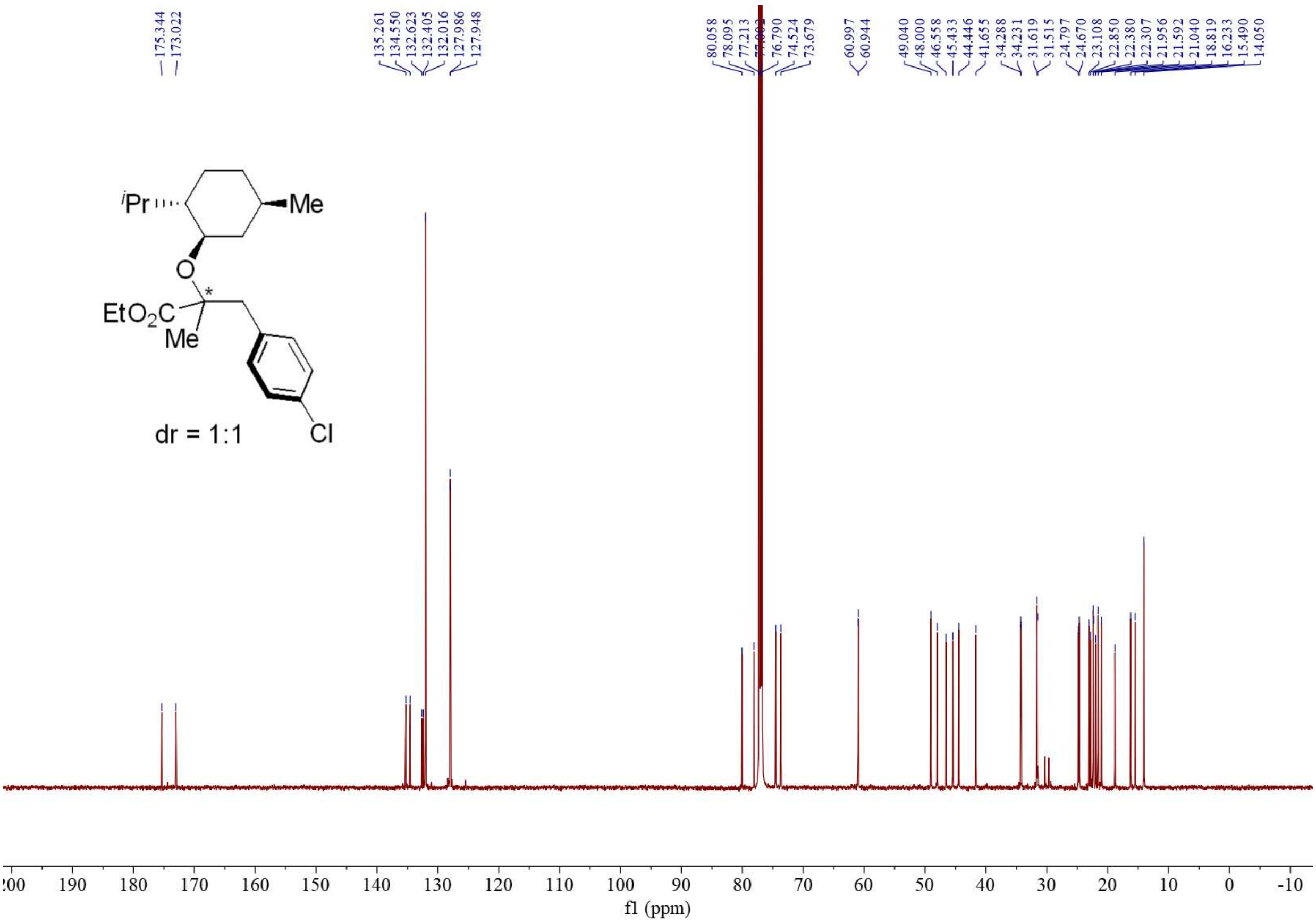

^{1}H NMR spectrum of **10** (500 MHz, $CDCl_3$)

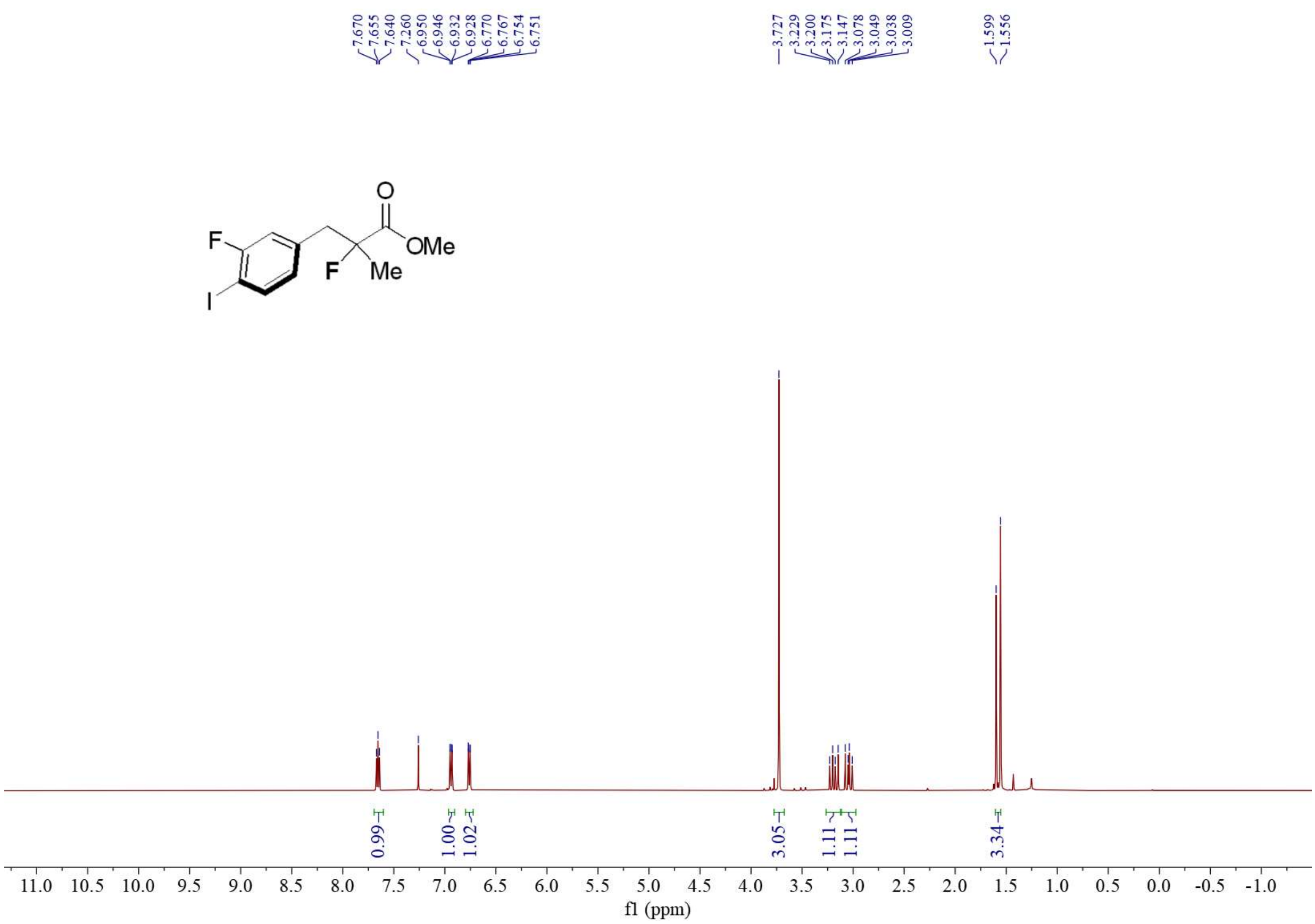

^{13}C NMR spectrum of **10** (126 MHz, $CDCl_3$)

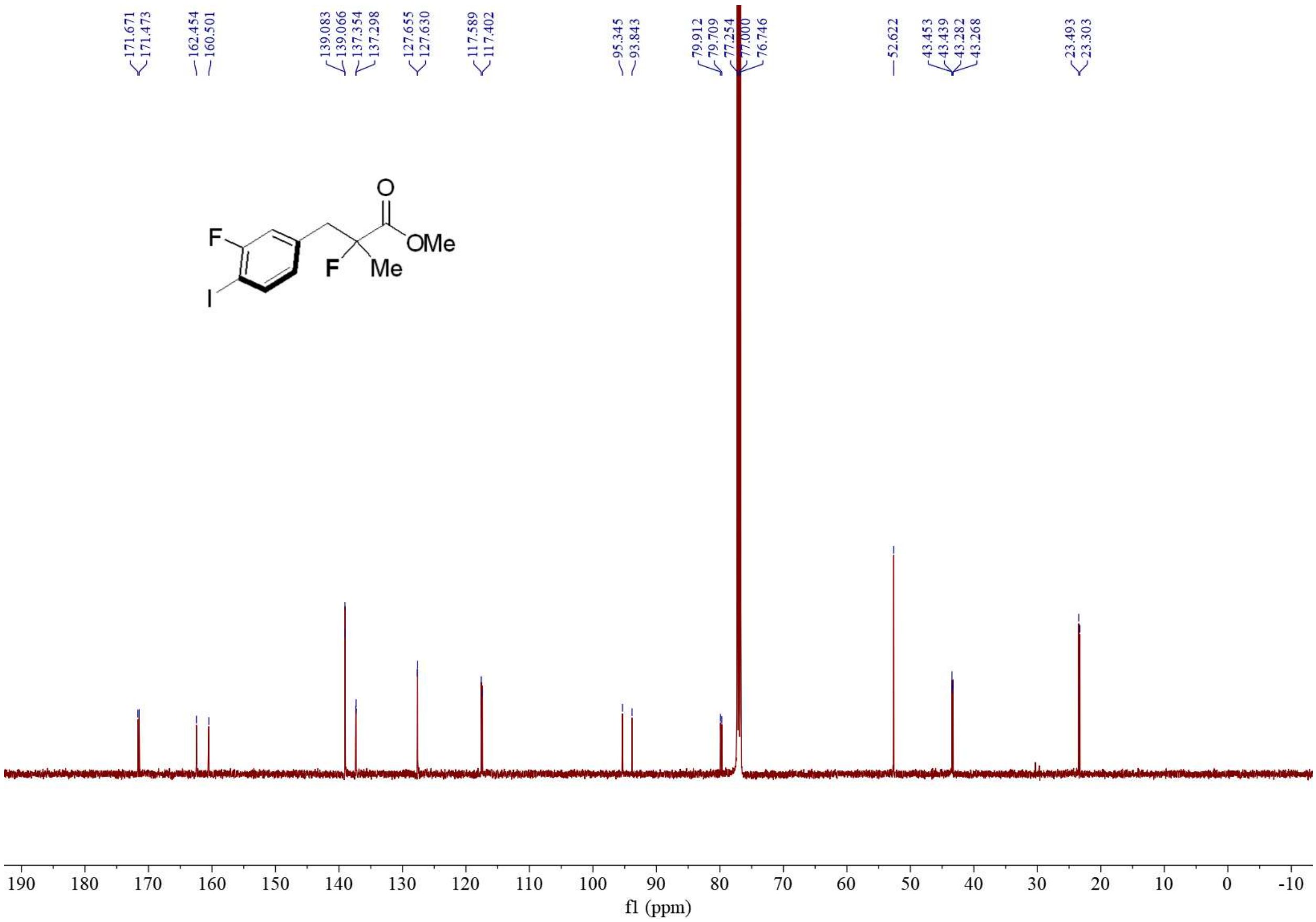

^{19}F{^{1}H} NMR spectrum of **10** (377 MHz, $CDCl_3$)

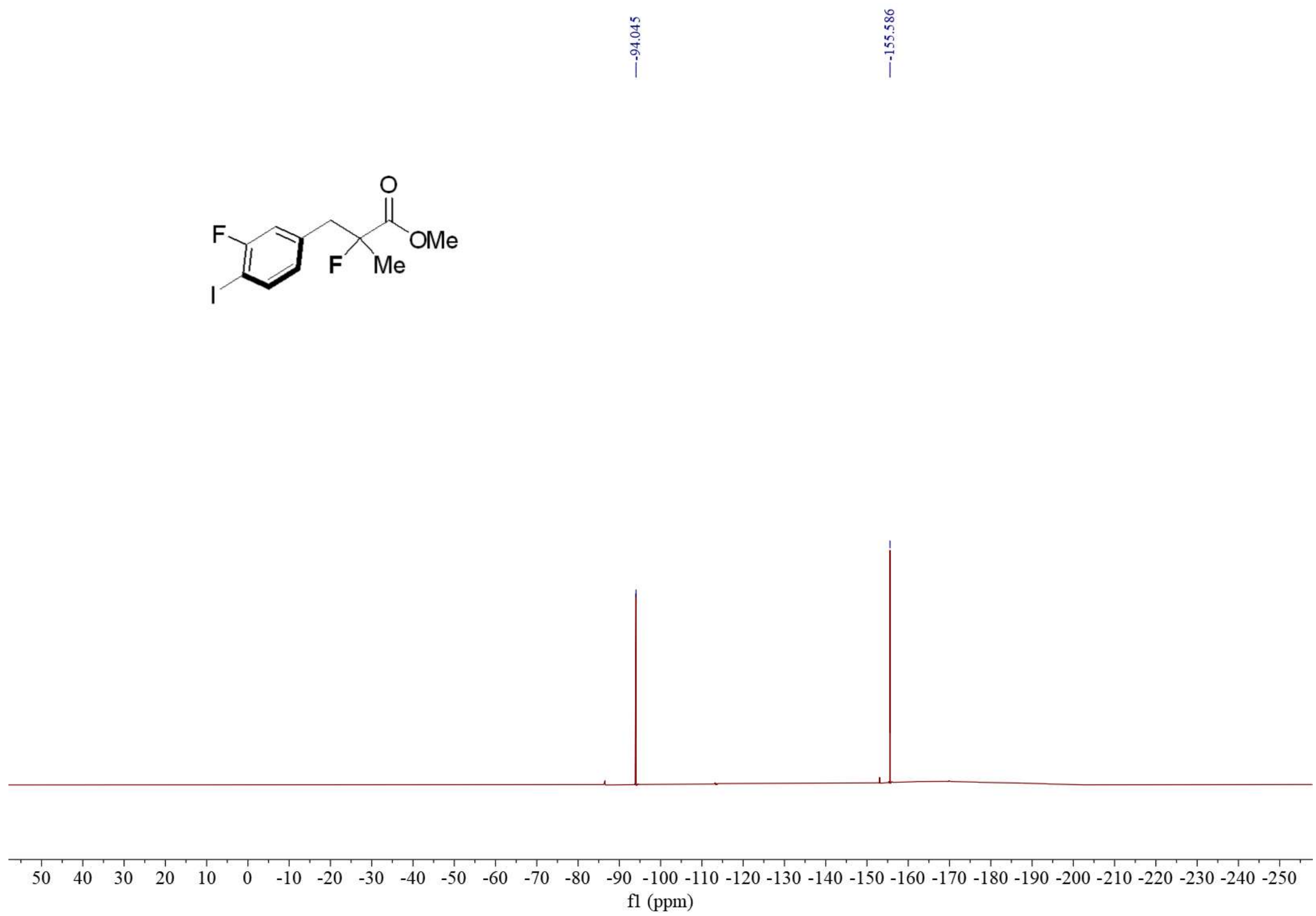

^{19}F NMR spectrum of **10** (377 MHz, $CDCl_3$)

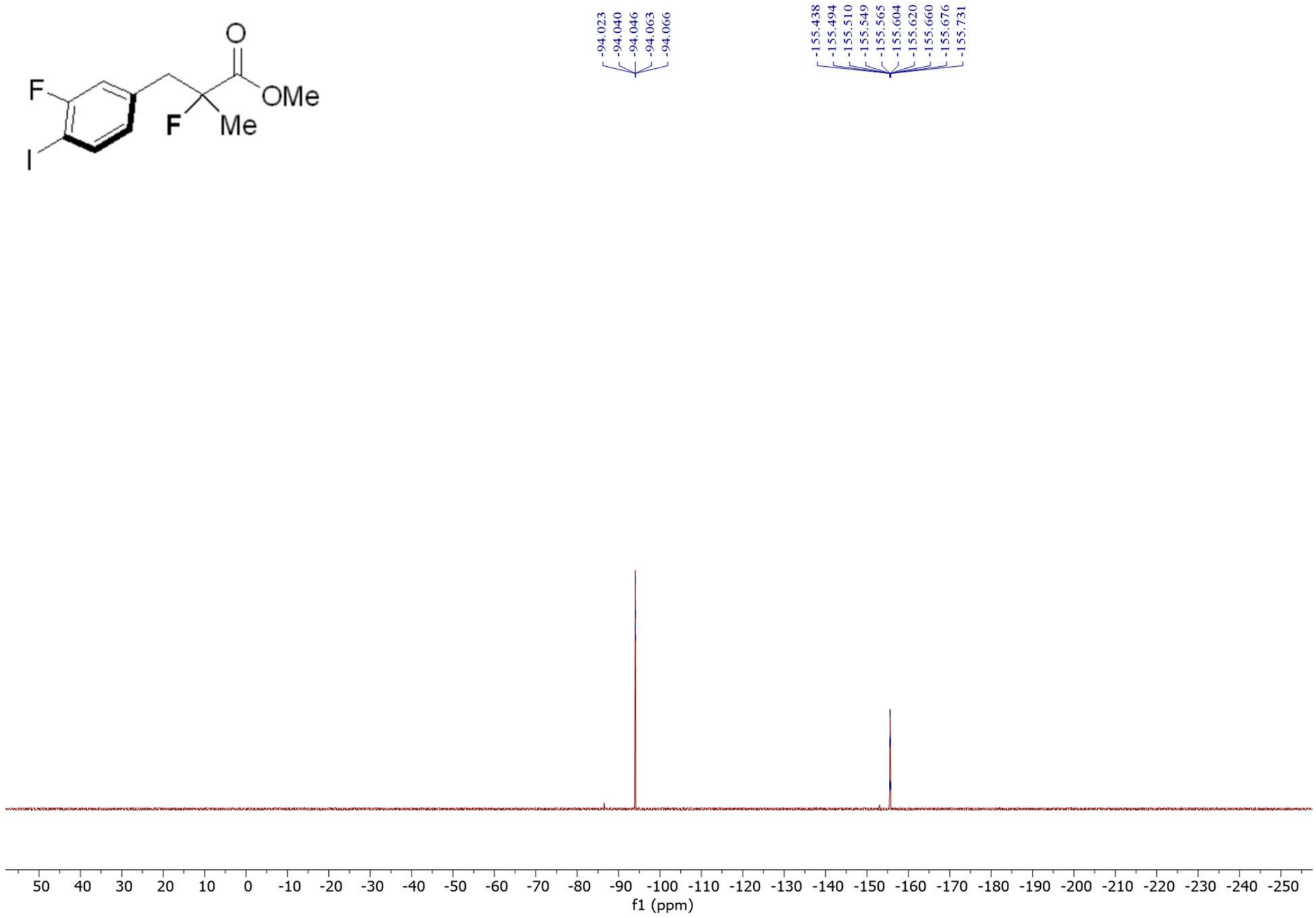

^{1}H NMR spectrum of **13** (400 MHz, $CDCl_3$)

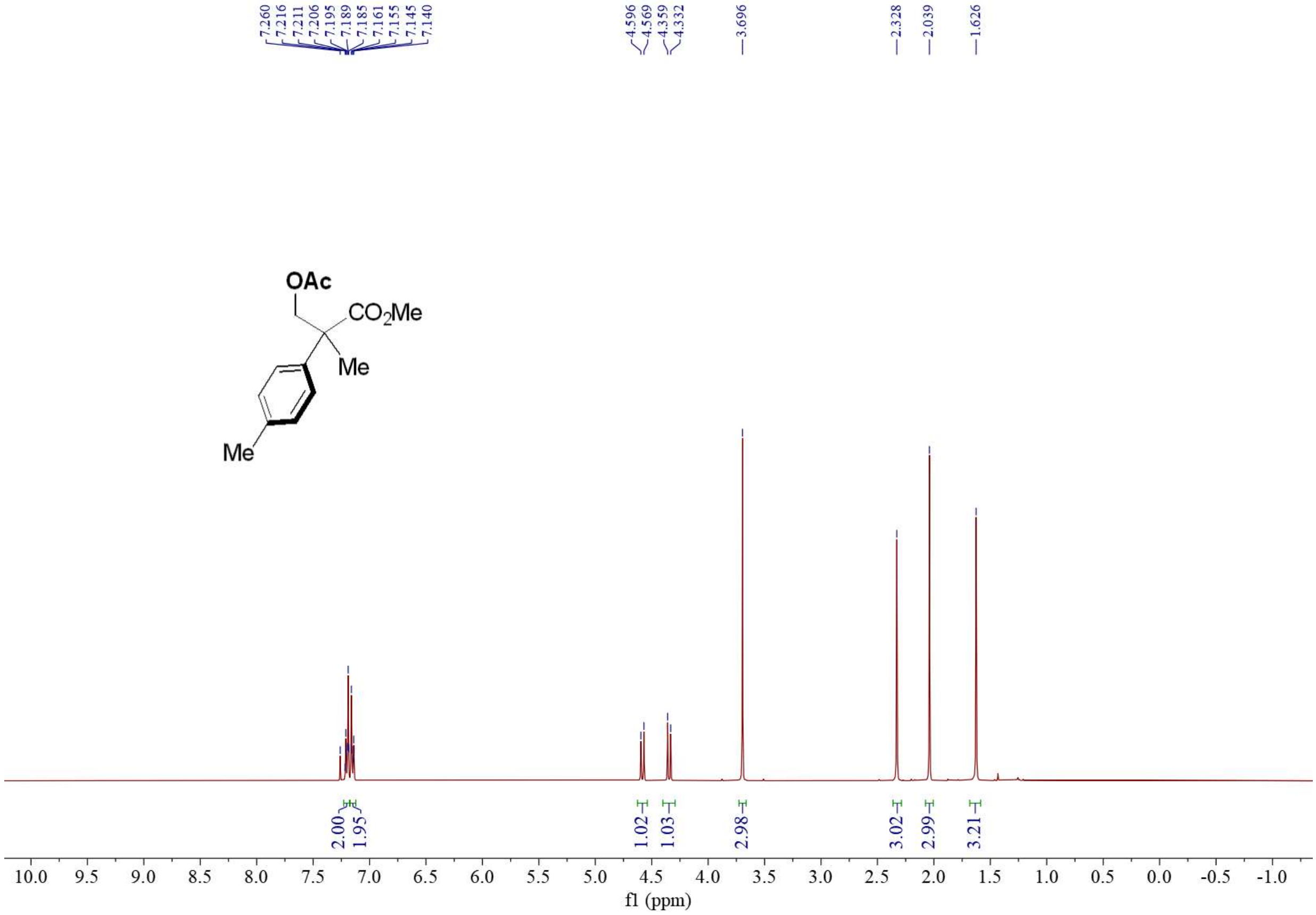

^{13}C NMR spectrum of **13** (101 MHz, $CDCl_3$)

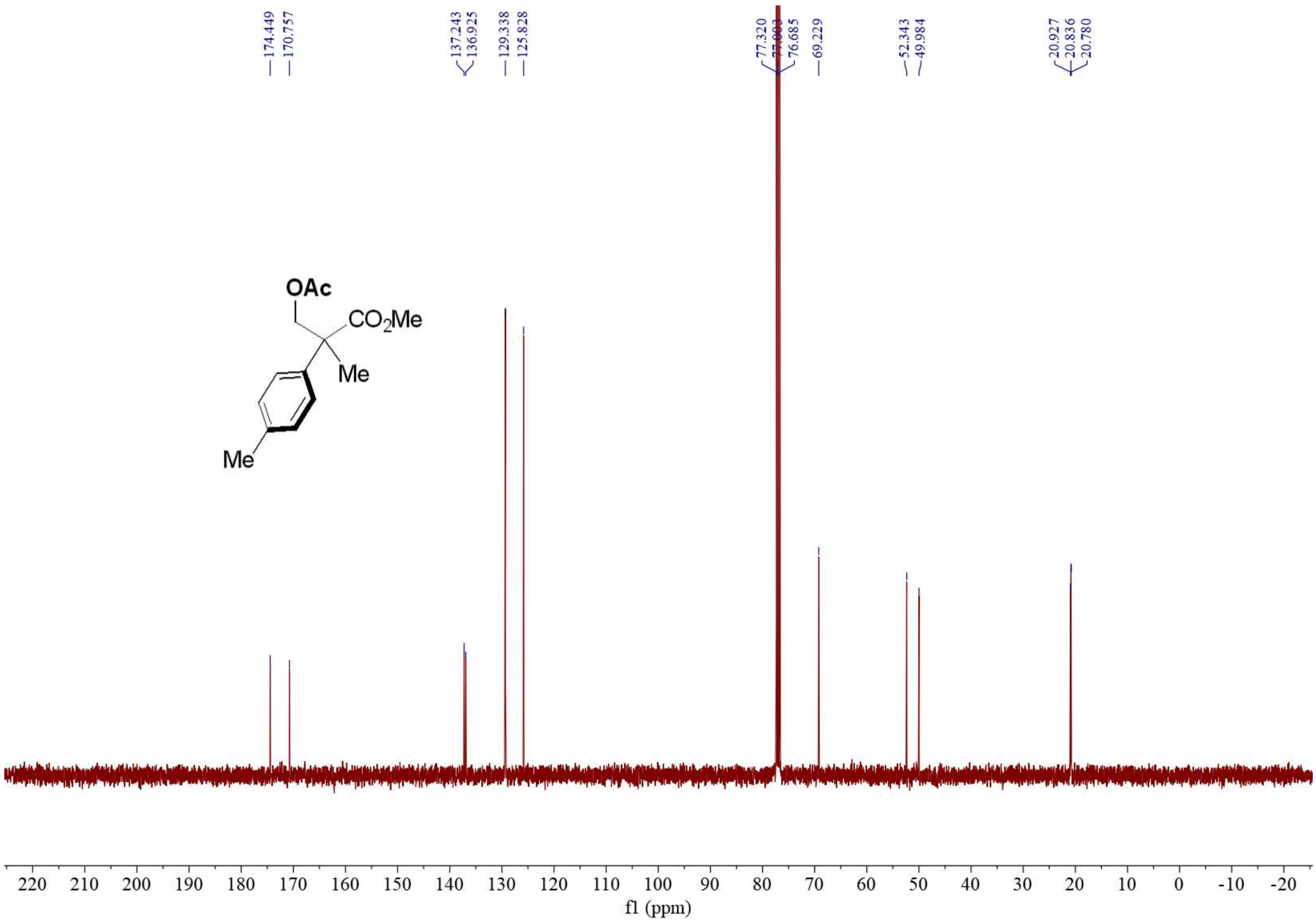

^{1}H NMR spectrum of **14** (400 MHz, $CDCl_3$)

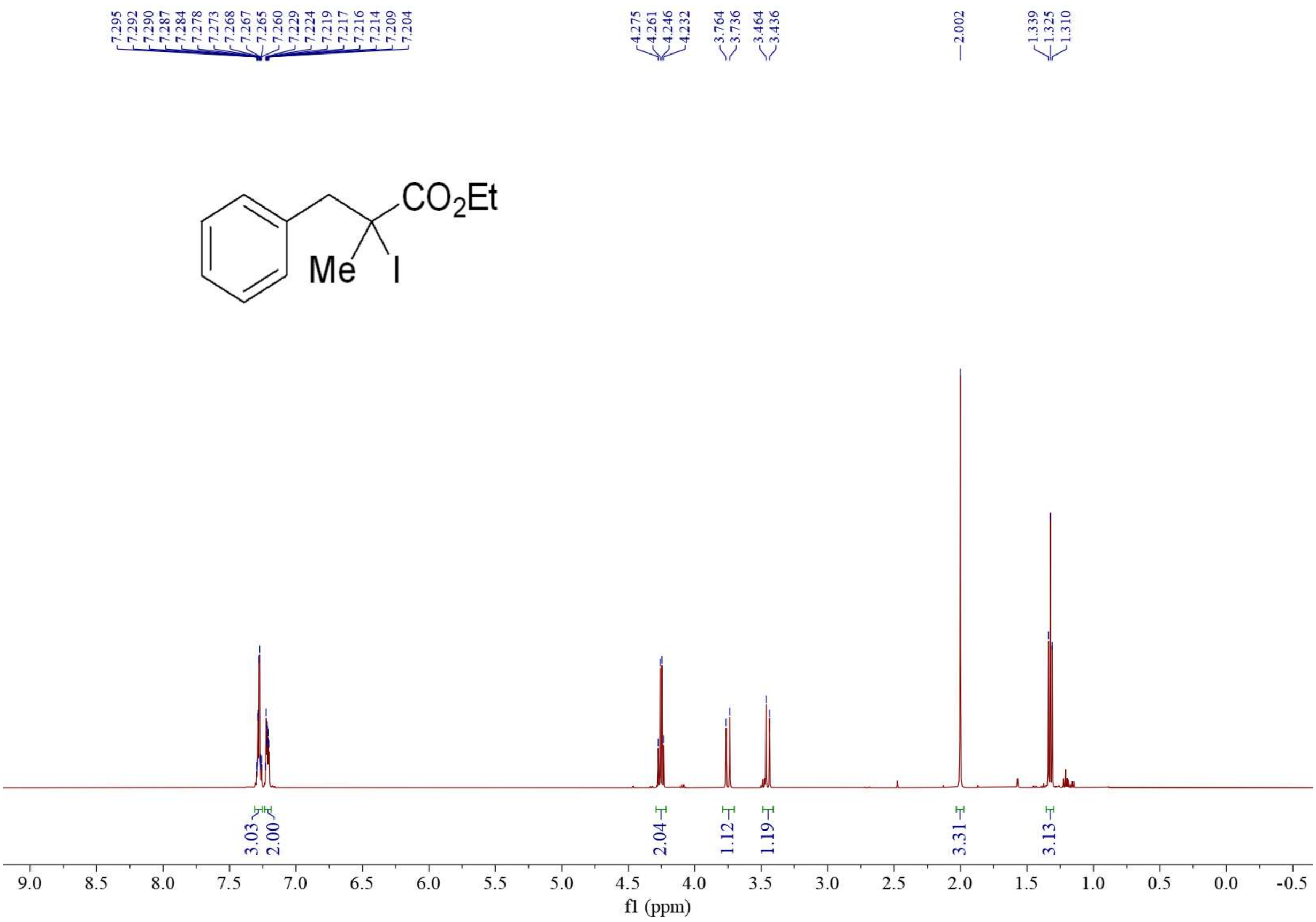

^{13}C NMR spectrum of **14** (101 MHz, $CDCl_3$)

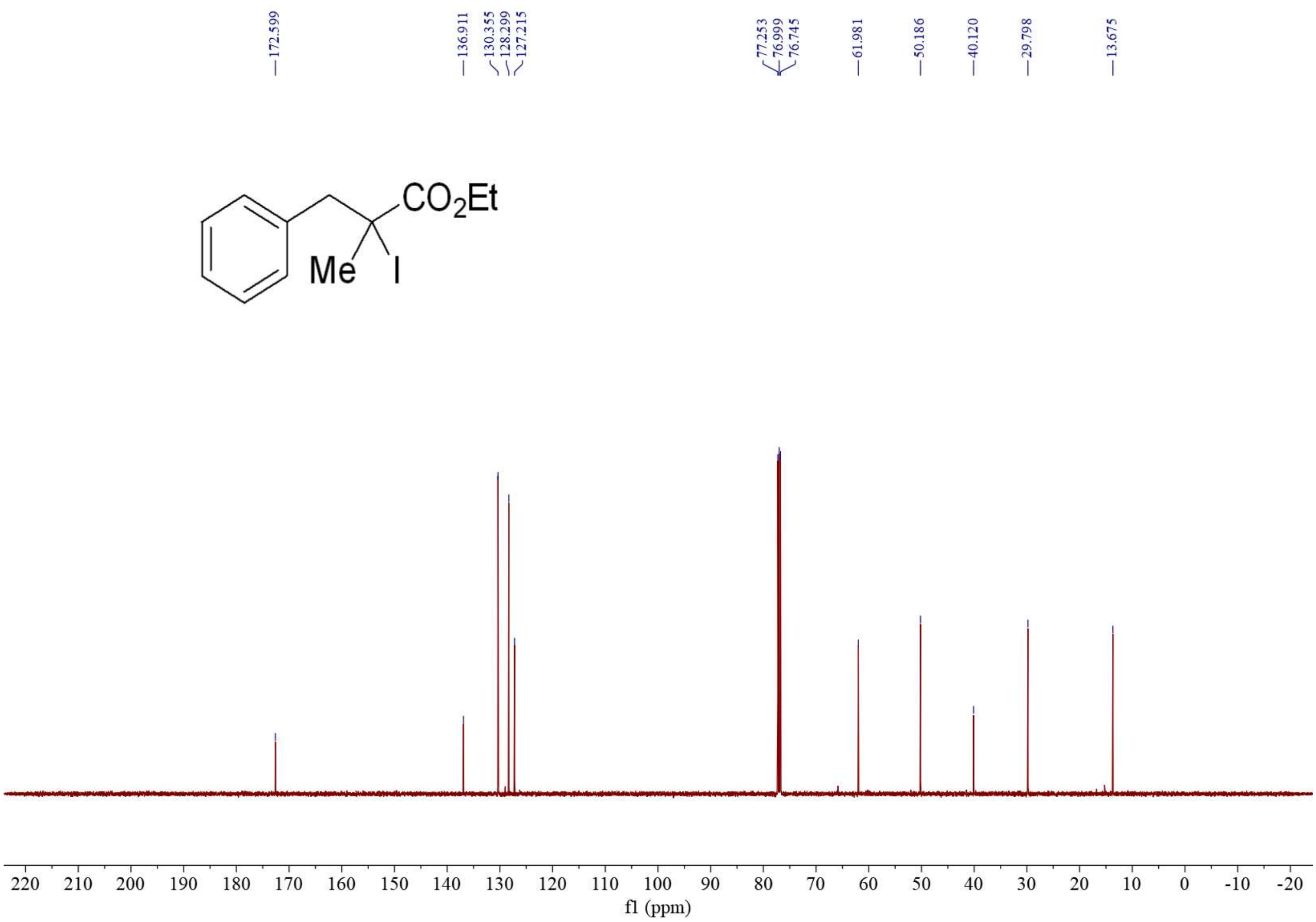

^{1}H NMR spectrum of (*R*)-**S17** (400 MHz, $CDCl_3$)

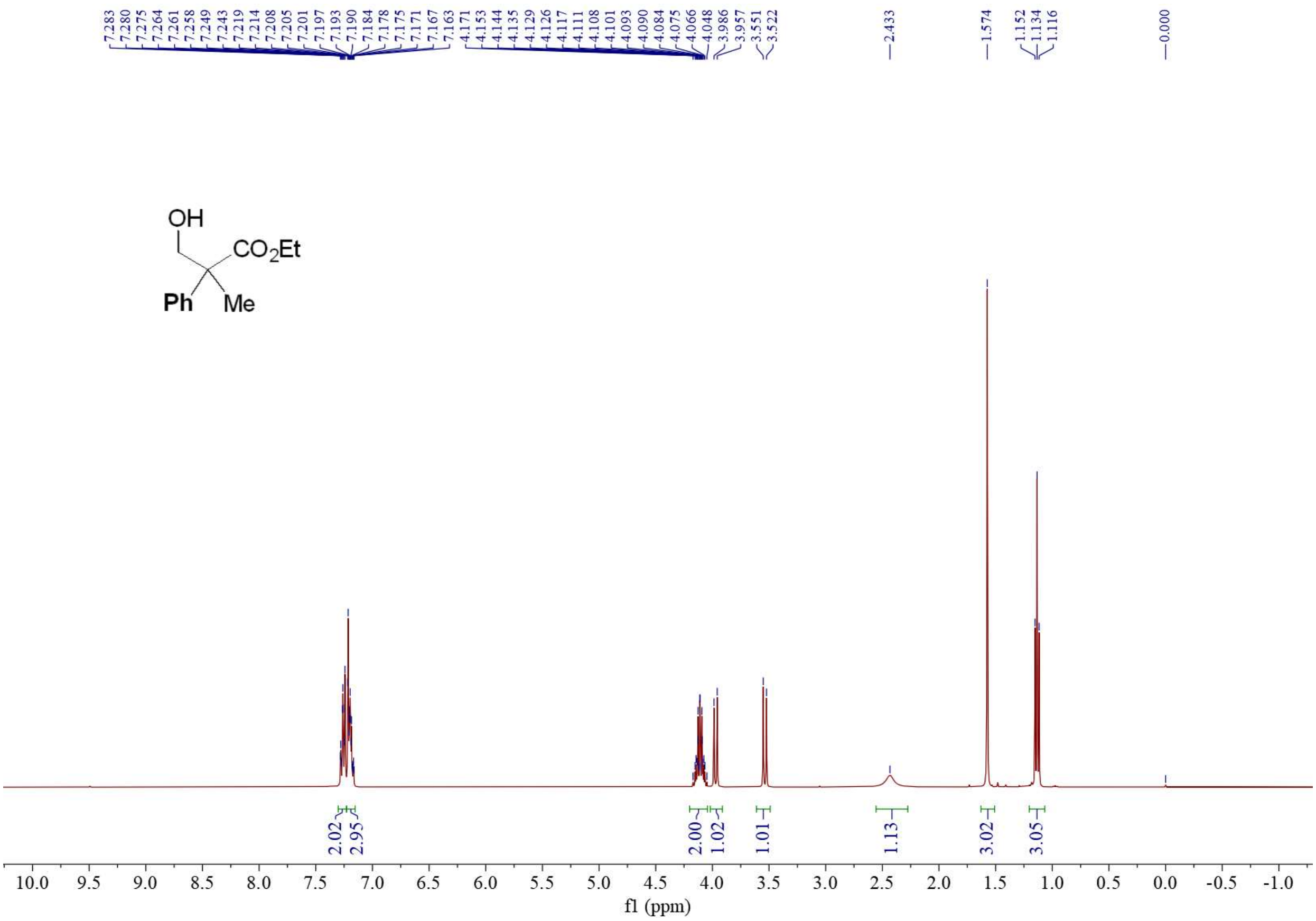

^{13}C NMR spectrum of (*R*)-**S17** (101 MHz, $CDCl_3$)

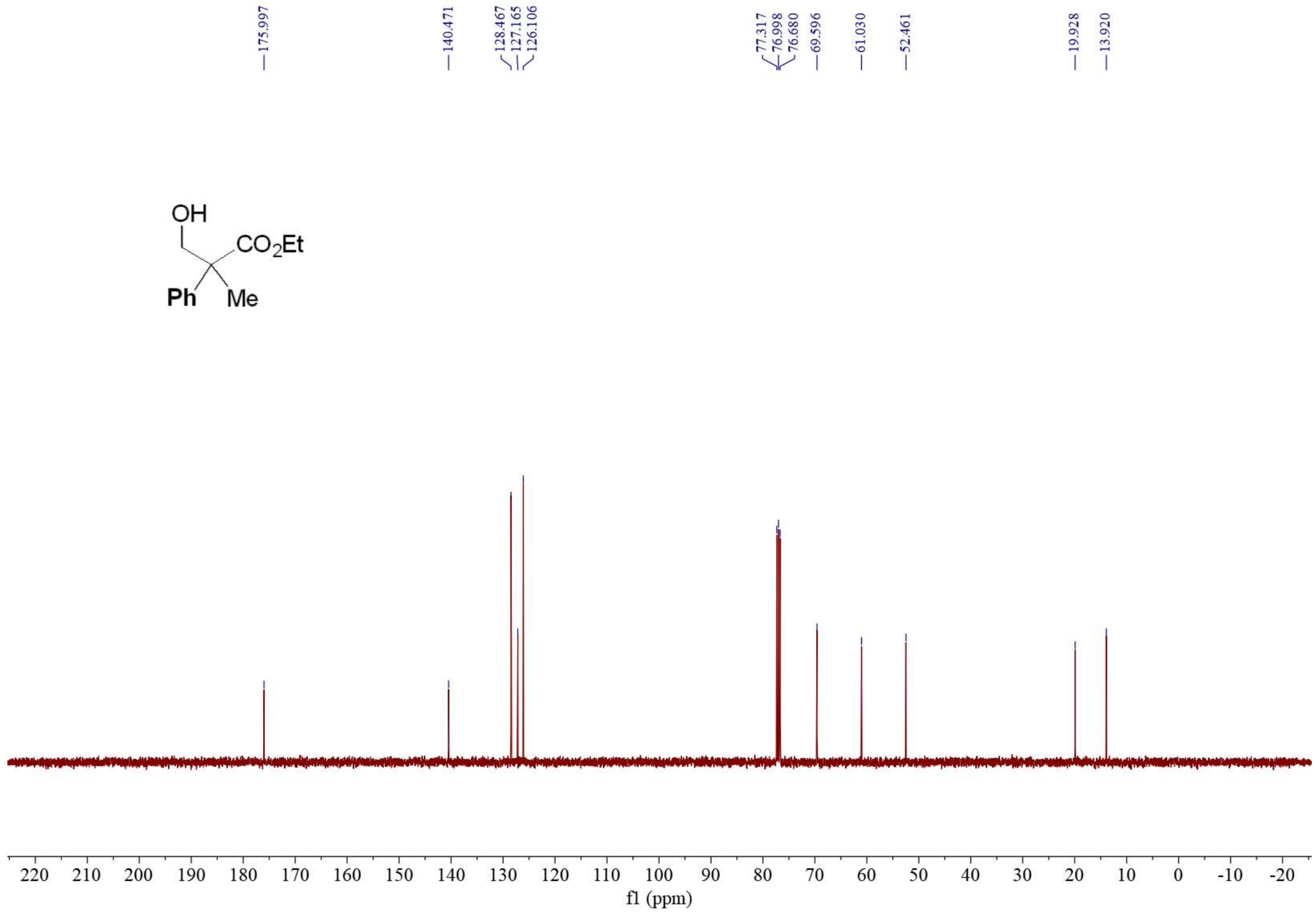

^{1}H NMR spectrum of (*R*)-**S18** (400 MHz, $CDCl_3$)

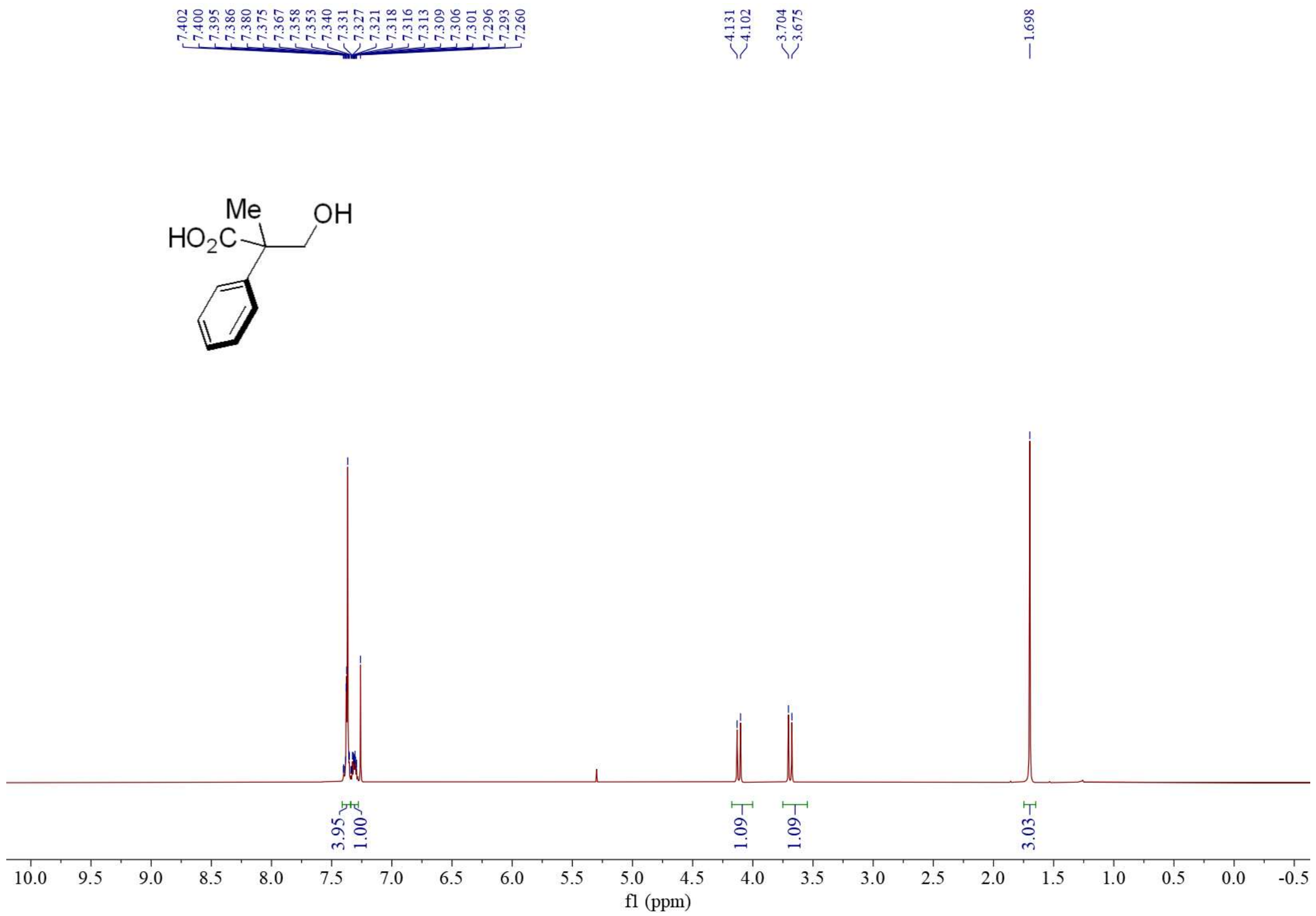

^{13}C NMR spectrum of (*R*)-**S18** (101 MHz, $CDCl_3$)

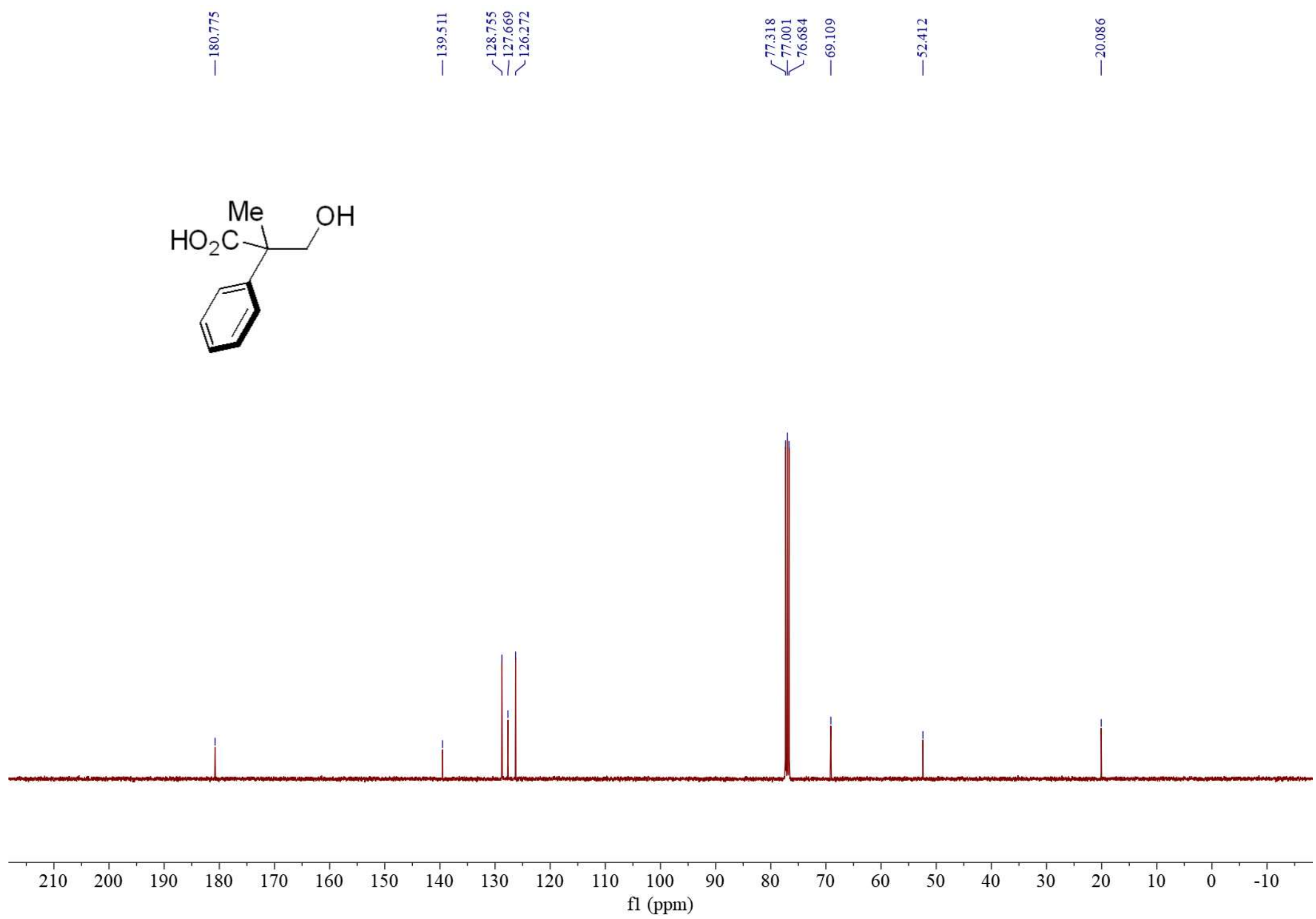

^{1}H NMR spectrum of (*S*)-**15** (400 MHz, $CDCl_3$)

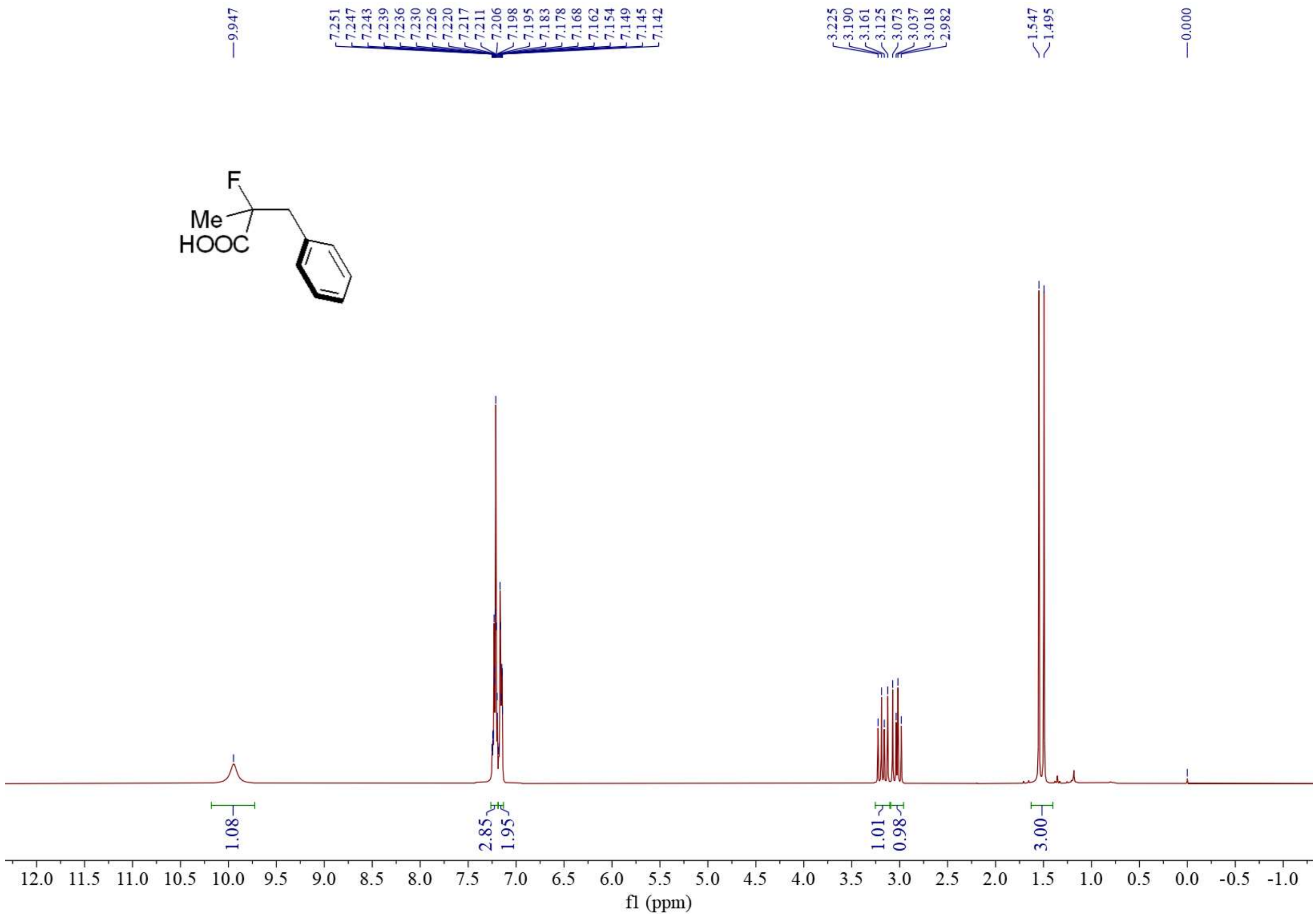

^{13}C NMR spectrum of (*S*)-**15** (101 MHz, $CDCl_3$)

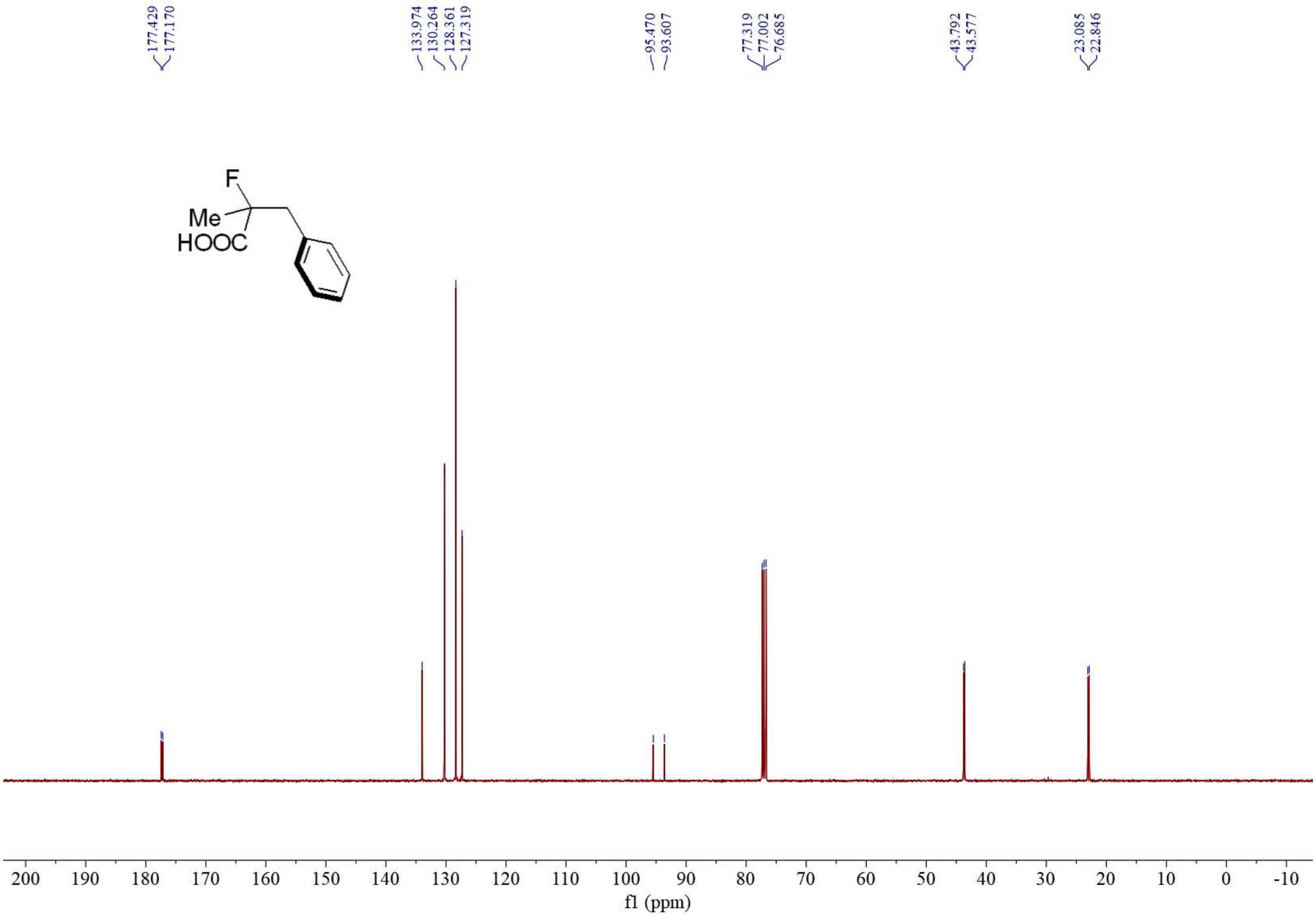

$^{19}F\{^{1}H\}$ NMR spectrum of (*S*)-**15** (377 MHz, $CDCl_3$)

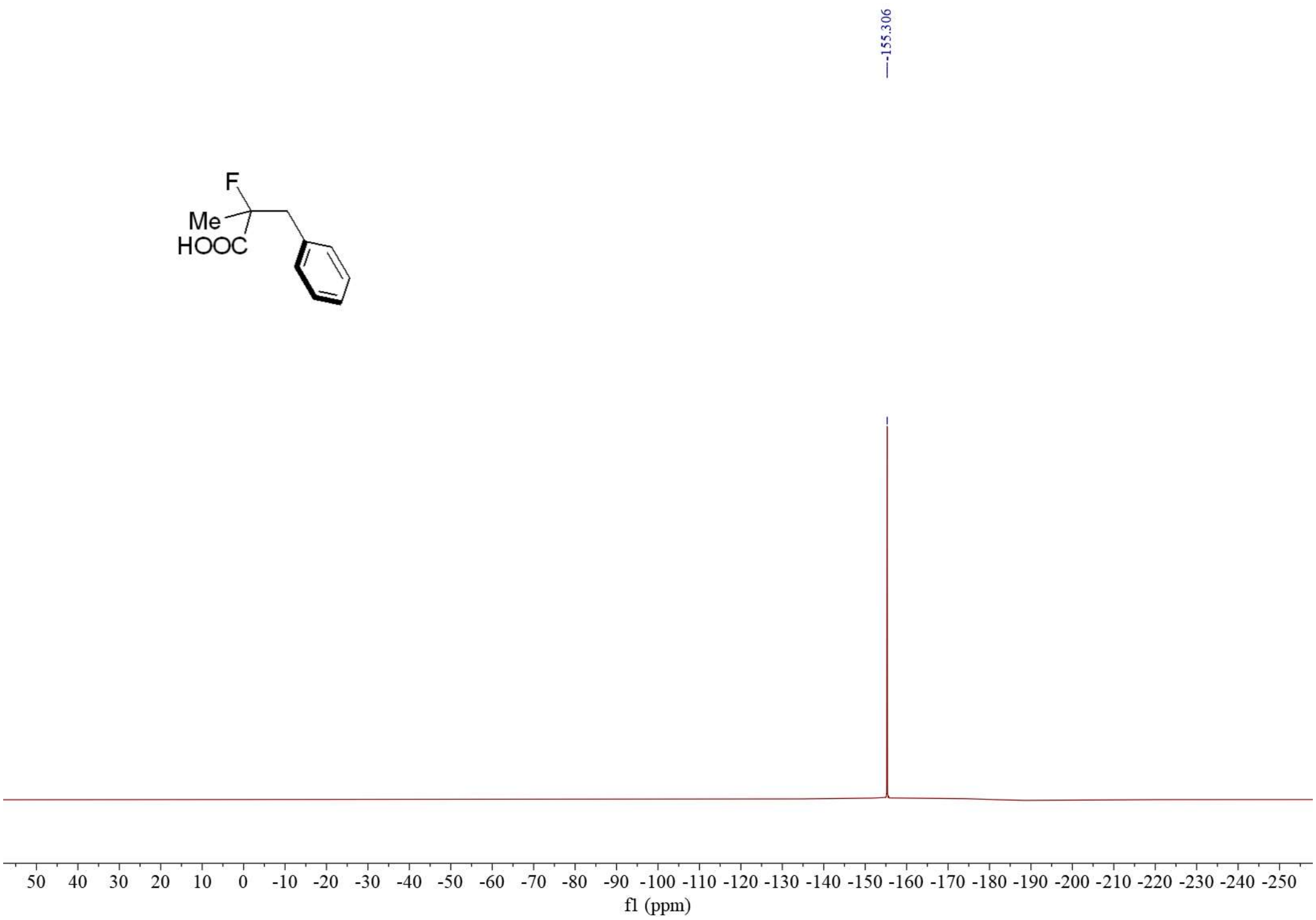

^{19}F NMR spectrum of (*S*)-**15** (377 MHz, $CDCl_3$)

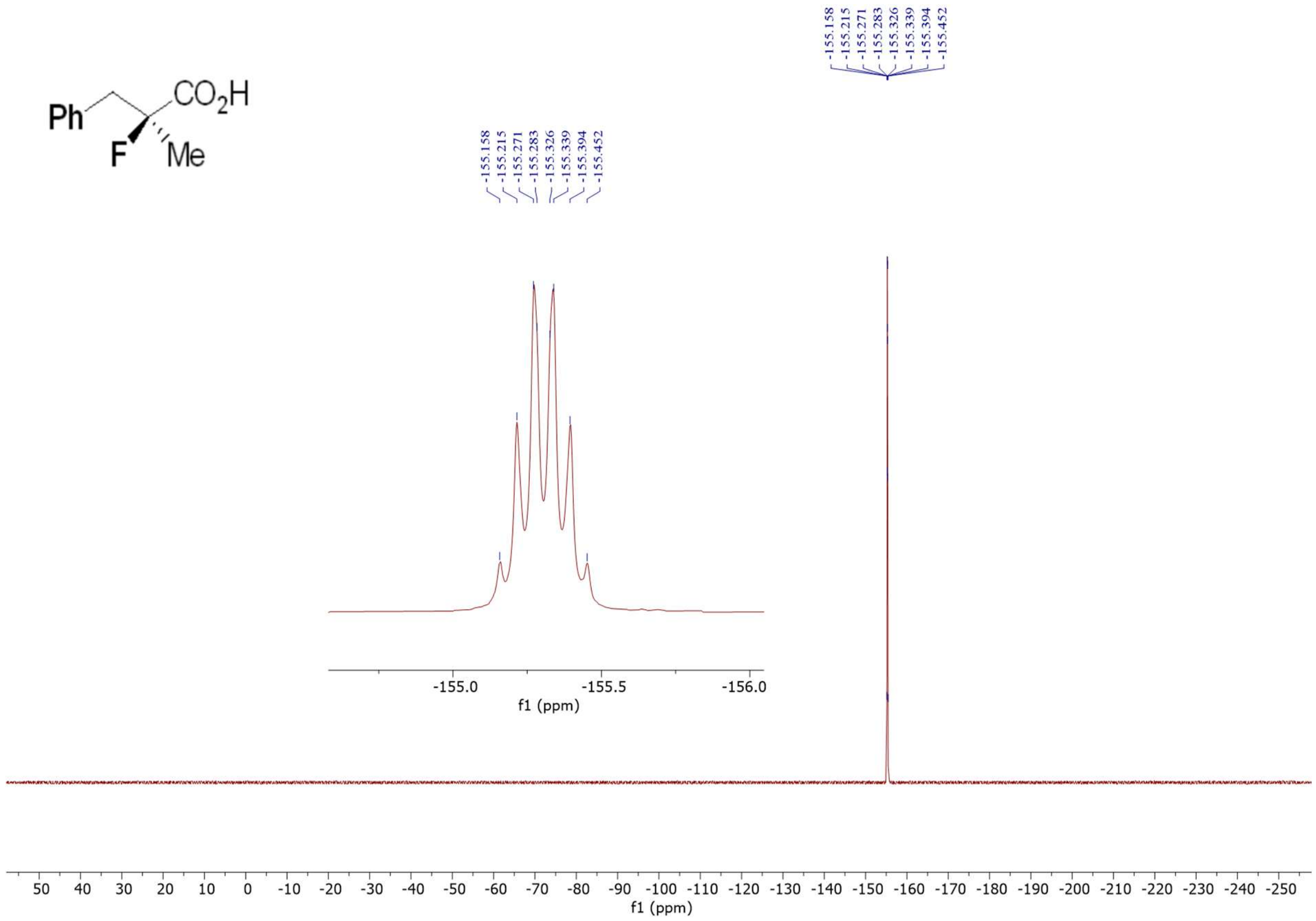

^{1}H NMR spectrum of (*S*)-**16** (400 MHz, $CDCl_3$)

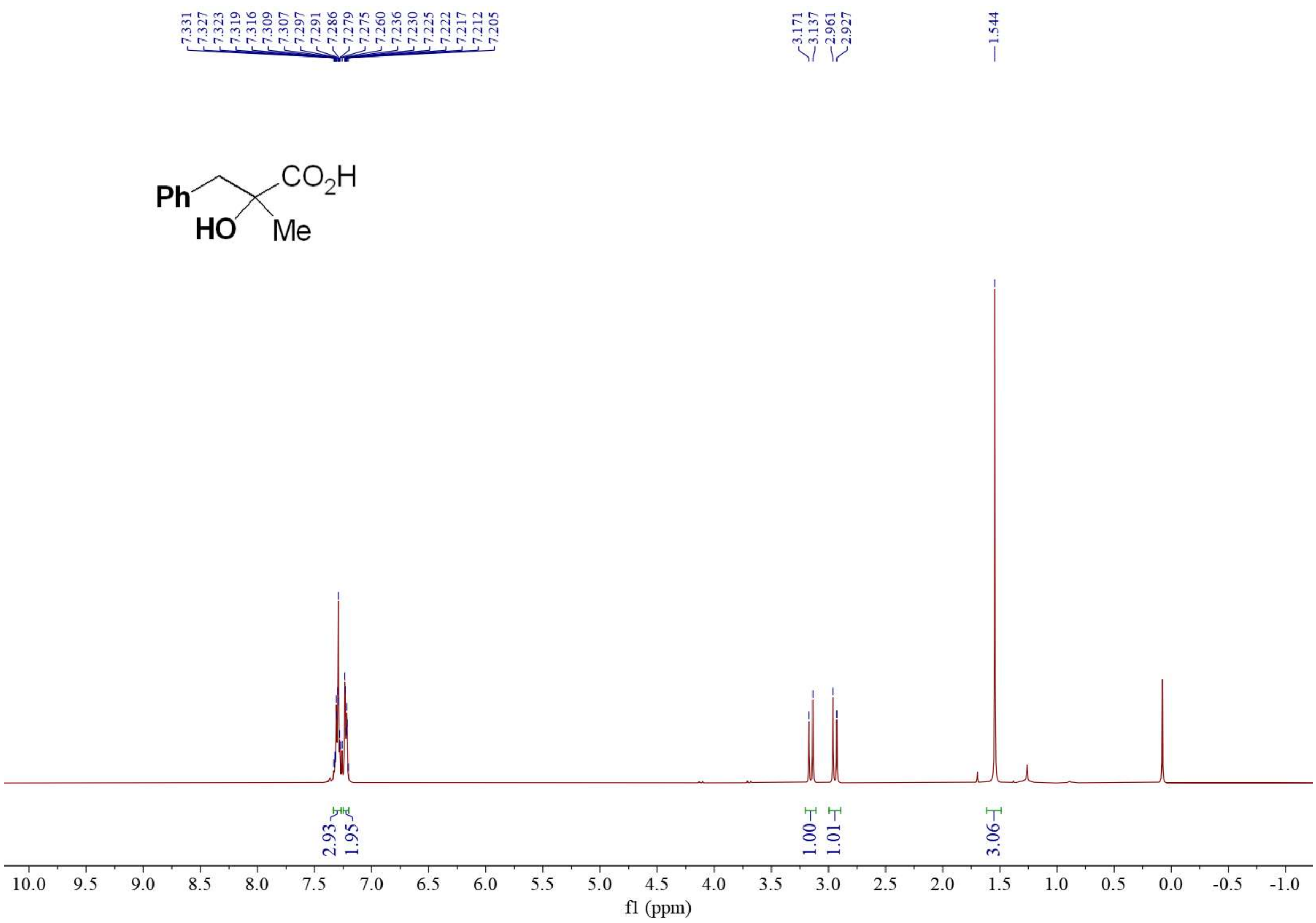

^{13}C NMR spectrum of (*S*)-**16** (101 MHz, $CDCl_3$)

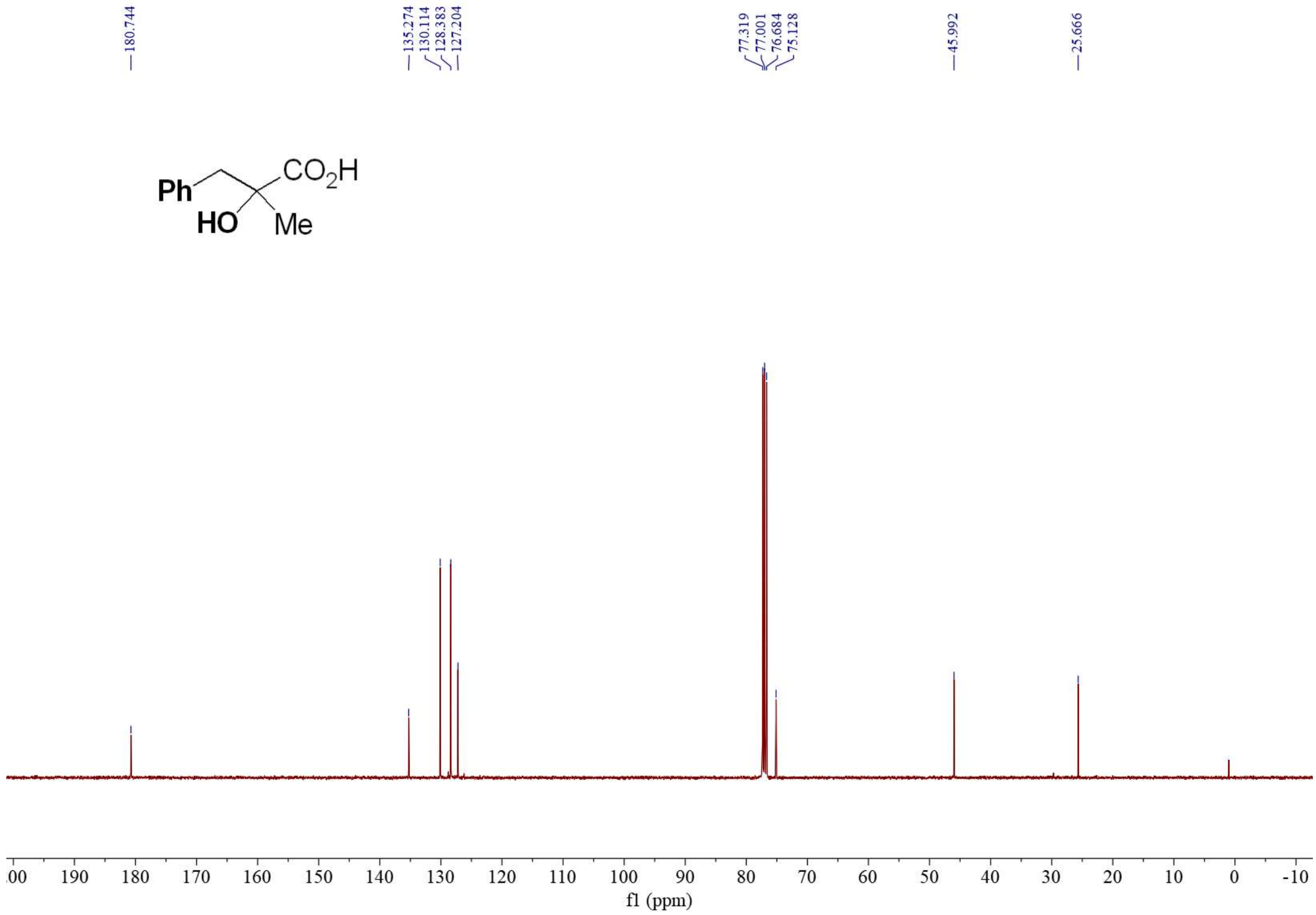

Supplement: Supplementary file 1 [file ANIE-65-e7649039-s002.pdf]
